# Combining palladium and ammonium halide catalysts for Morita–Baylis–Hillman carbonates of methyl vinyl ketone: from 1,4-carbodipoles to ion pairs†

**DOI:** 10.1039/d1sc03517g

**Published:** 2021-07-22

**Authors:** Yang Yang, Bo Zhu, Lei Zhu, Ying Jiang, Chun-Ling Guo, Jing Gu, Qin Ouyang, Wei Du, Ying-Chun Chen

**Affiliations:** Key Laboratory of Drug-Targeting and Drug Delivery System of the Ministry of Education, West China School of Pharmacy, and State Key Laboratory of Biotherapy, West China Hospital, Sichuan University Chengdu 610041 China duweiyb@scu.edu.cn ycchen@scu.edu.cn +86 28 85502609; College of Pharmacy, Third Military of Medical University Chongqing 400038 China ouyangq@tmmu.edu.cn

## Abstract

Here we report that Morita–Baylis–Hillman carbonates from diverse aldehydes and methyl vinyl ketones can be directly utilised as palladium-trimethylenemethane 1,4-carbodipole-type precursors, and both reactivity and enantioselectivity are finely regulated by adding a chiral ammonium halide as the ion-pair catalyst. The newly assembled intermediates, proposed to contain an electronically neutral π-allylpalladium halide complex and a reactive compact ion pair, efficiently undergo asymmetric [4 + 2] annulations with diverse activated alkenes or isatins, generally with high regio-, diastereo- and enantio-selectivity, and even switchable regiodivergent or diastereodivergent annulations can be well realised by tuning the substrate or catalyst assemblies. An array of control experiments, including UV/Vis absorption study and density functional theory calculations, are conducted to rationalise this new double activation mode combining a palladium complex and an ammonium halide as an ion-pair catalyst.

## Abstract 2

A double activation catalytic system combining a palladium complex and an ammonium halide was developed to promote the asymmetric [4 + 2] annulations of Morita–Baylis–Hillman carbonates of methyl vinyl ketone.

## Graphical abstract

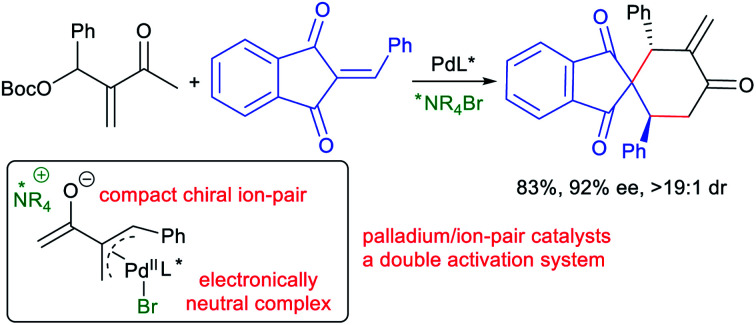

## Introduction

**Scheme 1:**
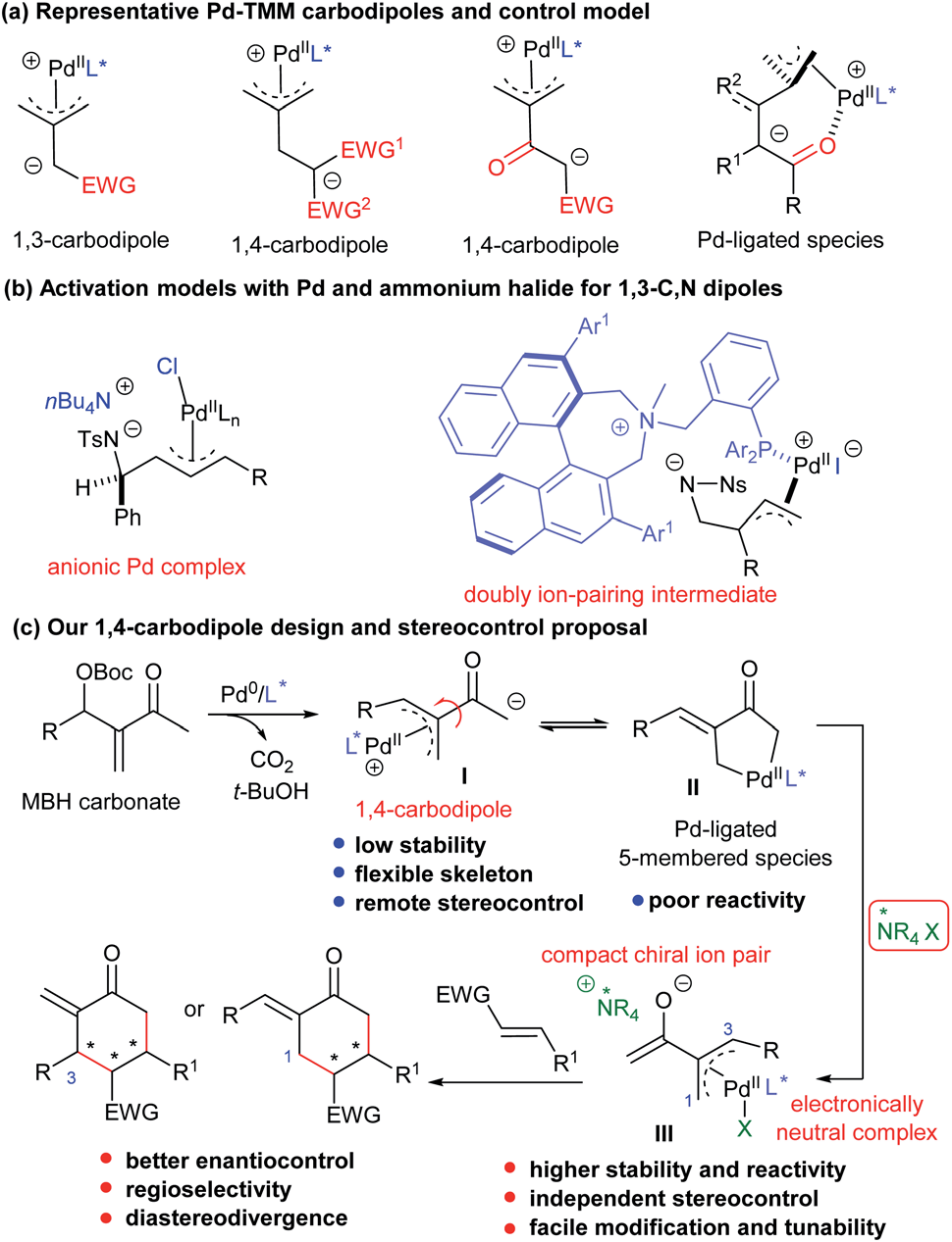
Diverse catalytic strategies involved in various Pd-based dipole species.

With the development of transition metal catalysis, zwitterionic species possessing a π-allylmetal complex moiety, which could be generated from allylic alcohol derivatives *in situ*, have been extensively employed for the construction of enantioenriched cyclic frameworks.^1^ The corresponding hetero 1,*n*-dipoles can be readily generated from various allylic alcohol-derived carbonate, carbamate, or ester derivatives.^2^ In addition, all-carbon-based dipoles have also been well explored,^3^ and those having a palladium-trimethylenemethane (Pd-TMM) motif received special attention.^4^ While fruitful scaffolds have been constructed by using Pd-TMM 1,3-carbodipoles,^5^ 1,4-carbodiploes from allylic carbonates, generally with two electron-withdrawing groups at the homoallylic position, could be efficiently utilised (Scheme 1a).^6^ However, despite the significant progress in this field, the substitution patterns of the TMM moiety are not expandable, and the 1,4-carbodipole precursors are generally limited to those possessing a gem-diactivated carbon centre to meet the required C–H acidity for deprotonation and stabilisation for anion intermediates. In addition, the stereocontrol for the related annulations usually relies on the selection of properly designed chiral P-centred ligands, generally with crowded architectures. Moreover, for more flexible 1,4-dipoles, a Pd(ii)-coordination site (including carbonyl, ester, and pyridine) embedding in the substrates is necessary for achieving high levels of enantioselectivity, by forming six- or seven-membered Pd(ii)-ligated intermediates (Scheme 1a). As a result, the development of an alternative catalytic system, rather than a sole Pd-based chiral complex, would be desirable for the reactions of some challenging substrates.

It has been documented that adding *n*Bu_4_Cl (TBAC) could significantly enhance the [3 + 2] annulation reaction of Pd(ii)-based 1,3-C,N-dipoles and activated alkenes, by possibly forming anionic Pd complexes (Scheme 1b, left).^7^ Ooi even designed a chiral BINOL-derived ammonium–phosphine hybrid ligand, which enabled fine stereocontrol in the construction of vicinal quaternary stereogenic centres in the annulation reaction of similar dipoles from oxazolidinones, *via* proposed doubly ion-pairing intermediates (Scheme 1b, right).^8^ Nevertheless, combining the double activation catalysis of ammonium halides with Pd-TMM carbodipole chemistry and deeply understanding the relevant activation mechanism still remain to be elucidated.

Morita–Baylis–Hillman (MBH) adducts, easily accessible by condensation between activated alkenes and carbonyl compounds, have been explored in asymmetric allylic alkylation reactions by forming π-allylpalladium intermediates.^9^ Typical MBH adducts from β-keto esters have been recently employed as Pd-TMM-type 1,4-carbodipole precursors in asymmetric [4 + 2] annulations *via* the deprotonation strategy;^6*e*^ nevertheless, the common analogues derived from simple enones, such as methyl vinyl ketone (MVK), and with broader substitution patterns (Scheme 1c), are much more challenging for similar transformations, and no success has been achieved yet. It was proposed that the corresponding intermediates, either 1,4-carbodipoles **I** or Pd-ligated five-membered species **II**^10^ after the oxidative addition/deprotonation process, might encounter several disadvantages, such as low stability or poor reactivity; especially, the stereocontrol would be quite challenging because of the flexibility of the skeleton together with the long distance between the nucleophilic site and the chiral ligand. Therefore, we envisaged that the addition of ammonium halide salts, as ion-pair catalysts (IPCs),^11^ would result in the formation of ion-pairing intermediates **III** from unstable 1,4-carbodipole species **I**, which would be potentially helpful for the subsequent reaction: (1) the cationic π-allylpalladium moiety would be neutralised by the halide of the IPC, especially when Cl^−^ or Br^−^ is used as a strong coordination ligand;^12^ (2) the formation of a compact chiral ion-pair would not only stabilise the labile enolate but also guarantee better reactivity and enantioselectivity in the initial key addition process; (3) the assembly and application of an individual chiral IPC and chiral ligand would be easily tunable, thus providing better synergistic stereocontrol and potentially realising diastereodivergent synthesis in the annulations; (4) such a double activation catalytic strategy would be different from the previously reported cooperative catalytic mode combining palladium and phase-transfer catalysts (Scheme 1c).^13^

## Results and discussion

### Condition optimization

Based on the above considerations, we conducted the reaction of the MBH carbonate **1a** condensed from MVK and benzaldehyde with 2-benzylidene-1*H*-indene-1,3(2*H*)-dione **2a** under the catalysis of palladium. After extensive screening, it was found that the expected [4 + 2] spirocyclic product^14^**3a** with an *exo*-methylene group could be efficiently obtained with exclusive regio- and diastereo-selectivity, by employing Pd(OAc)_2_ and a bisphosphine ligand, such as BINAP **L1** or SKP **L2**,^9*c*^ suggesting that 1,4-carbodipole **I** could be generated.^6*e*^ Unfortunately, very low enantiocontrol was observed, probably due to the remote reaction site (Table 1, entries 1 and 2). Phosphoramidite (*R*)-**L3** was tested as well, but failed to afford any product (entry 3). We also tried the bifunctional ligand **L4** bearing an ammonium salt,^8*a*^ without success either (entry 4). In contrast, when cinchonine-derived ammonium salt **C1** was added into the previously inert palladium/achiral phosphoramidite **L5** catalytic system, high reactivity, albeit with low enantiocontrol, was observed, suggesting the potential formation of a reactive ion-pairing intermediate (entry 5). Chiral salts had apparent effects on both reactivity and enantioselectivity (entries 6–10), and **C4** with a bulky substituent gave a higher ee value (entry 8).^15^ It should be noted that no reaction occurred without the phosphoramidite ligand even in the presence of **C4** (entry 11). In contrast, the combination of a bisphosphine ligand **L1** or **L2** and **C4** proved to be unsuccessful (entries 12 and 13). These results indicated that adding an ammonium halide would apparently change the previously formed Pd(ii) species of **1a**.^10^ Subsequently, a few chiral phosphoramidites were explored with chiral IPC **C4**. As a mismatch was observed for ligand (*R*)-**L3** (entry 14), the enantioselectivity was slightly improved with (*S*)-**L3** (entry 15). Moreover, better enantiocontrol was achieved by changing the *N*-substituent of the phosphoramidite to a bulkier one (entry 16), and the optimal results were obtained with **L7** (entry 17). Meanwhile, the significance of IPC was further confirmed, since no reaction occurred without **C4** (entry 18). Pleasingly, the reactivity and stereoselectivity were almost retained with lower loadings of the dipole precursor, chiral ligand and IPC (entry 19).^10^

**Table 1:** Screening conditions of asymmetric [4 + 2] annulation between MBH carbonate **1a** and activated alkene **2a**^a^

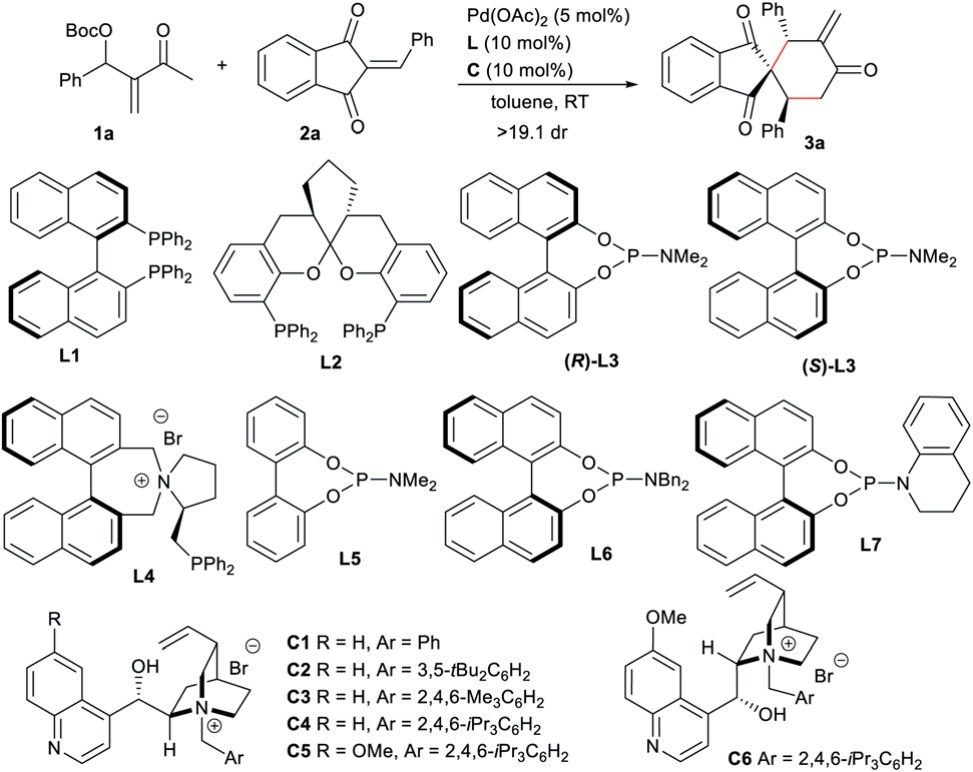					
Entry	Ligand	C	Time (h)	Yield^b^ (%)	ee^c^ (%)
1	**L1**	—	24	86	−3
2	**L2**	—	24	77	6
3	(*R*)-**L3**	—	24	NR	—
4	**L4**	—	24	NR	—
5	**L5**	**C1**	39	95	−2
6	**L5**	**C2**	39	27	−9
7	**L5**	**C3**	39	74	39
8	**L5**	**C4**	18	70	81
9	**L5**	**C5**	39	87	50
10	**L5**	**C6**	39	<10	−17
11	—	**C4**	24	NR	—
12	**L1**	**C4**	24	NR	—
13	**L2**	**C4**	24	<5	—
14	(*R*)-**L3**	**C4**	6	46	70
15	(*S*)-**L3**	**C4**	6	63	86
16	**L6**	**C4**	22	63	91
17	**L7**	**C4**	36	86	93
18	**L7**	—	36	NR	—
19^d^	**L7**	**C4**	36	83	92

a Unless otherwise noted, reactions were carried out with MBH carbonate **1a** (0.075 mmol), acceptor **2a** (0.05 mmol), Pd(OAc)_2_ (5 mol%), **L** (10 mol%) and **C** (10 mol%) in toluene (1.0 mL) under Ar.

b Isolated yield.

c Determined by HPLC analysis on a chiral stationary phase.

d With **1a** (0.06 mmol), **L7** (7.5 mol%) and **C1** (7.5 mol%).

### Substrate scope of regioselective [4 + 2] annulations

With the optimal conditions in hand, we next explored the substrate scope of the asymmetric [4 + 2] annulations of MBH carbonates **1** and 2-alkylidene indene-1,3-diones **2** under the double activation catalysis of Pd(OAc)_2_, ligand **L7** and IPC **C4**. The results are summarised in Table 2. For MBH carbonates **1** derived from different aromatic aldehydes or cinnamaldehyde and MVK, good reactivity was observed even with 2.5 mol% loadings of a palladium catalyst in some cases, and the corresponding products **3a–3p** were smoothly produced in high yields with excellent stereoselectivity (Table 2, entries 1–16). It should be noted that the halide substituents on the aryl ring could be well-tolerated. When acceptors **2** with a different aryl group were employed, similarly good results were generally obtained (entries 17–25). An alkyl-substituted substrate was also applicable albeit with moderate enantioselectivity (entry 26). In addition, product **3aa** with an additional stereogenic centre was afforded with good diastereoselectivity from a perillaldehyde-derived enone (entry 27).^16^ In some cases, using Pd(OAc)_2_ showed low reactivity, and thus other palladium sources were applied for better results (entries 17, 26 and 27).

**Table 2:** Substrate scope of asymmetric [4 + 2] annulations between MBH carbonates **1** and alkenes **2**^a^

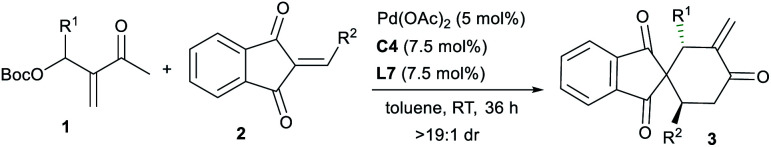				
Entry	R^1^	R^2^	Yield^b^ (%)	ee^c^ (%)
1	Ph	Ph	**3a**, 83	92
2^d^	3-CH_3_C_6_H_4_	Ph	**3b**, 76	93
3^d^	4-CH_3_C_6_H_4_	Ph	**3c**, 78	93
4	4-PhC_6_H_4_	Ph	**3d**, 80	92
5	4-FC_6_H_4_	Ph	**3e**, 93	94
6^e^	3,4-Cl_2_C_6_H_3_	Ph	**3f**, 70	93
7^d^	2-BrC_6_H_4_	Ph	**3g**, 94	92
8	3-BrC_6_H_4_	Ph	**3h**, 90	92
9^e^	4-BrC_6_H_4_	Ph	**3i**, 89	89
10	2-NO_2_C_6_H_4_	Ph	**3j**, 80	88
11	4-NO_2_C_6_H_4_	Ph	**3k**, 98	95
12	4-CNC_6_H_4_	Ph	**3l**, 83	95
13	2-Naphthyl	Ph	**3m**, 84	92
14^e^	2-Furyl	Ph	**3n**, 81	90
15^e^	3-Furyl	Ph	**3o**, 88	91
16	2-Styryl	Ph	**3p**, 75	92
17^f^	Ph	2-CH_3_C_6_H_4_	**3q**, 96	94
18^e^	Ph	4-CH_3_C_6_H_4_	**3r**, 90	93
19	Ph	4-*t*BuC_6_H_4_	**3s**, 90	93^g^
20	Ph	3-MeOC_6_H_4_	**3t**, 82	96
21	Ph	2-ClC_6_H_4_	**3u**, 56	81
22	Ph	4-FC_6_H_4_	**3v**, 80	92
23	Ph	4-BrC_6_H_4_	**3w**, 77	95
24	Ph	3-NO_2_C_6_H_4_	**3x**, 61	92
25	Ph	3-Furyl	**3y**, 93	85
26^h^	Ph	Cyclopropanyl	**3z**, 96	61^i^
27^h^	Ph	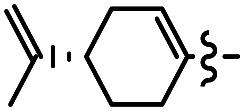	**3aa**, 73	—j

a Unless otherwise noted, reactions were carried out with MBH carbonate **1** (0.12 mmol), alkene **2** (0.10 mmol), Pd(OAc)_2_ (5 mol%), **C4** (7.5 mol%) and **L7** (7.5 mol%) in toluene (1.0 mL) at rt for 36 h under Ar.

b Isolated yields.

c Determined by HPLC analysis on a chiral stationary phase; generally dr > 19 : 1 by ^1^H NMR analysis.

d For 72 h.

e With Pd(OAc)_2_ (2.5 mol%), **C4** (3.75 mol%) and **L7** (3.75 mol%) in toluene (1.0 mL).

f With Pd_2_(dba)_3_ (2.5 mol%).

g The absolute configuration of enantiopure **3s** was determined by X-ray analysis. The other products were assigned by analogy.

h With PdCp(η^3^-allyl) (5 mol%).

i Dr = 15 : 1.

j Dr = 8 : 1.

**Scheme 2:**
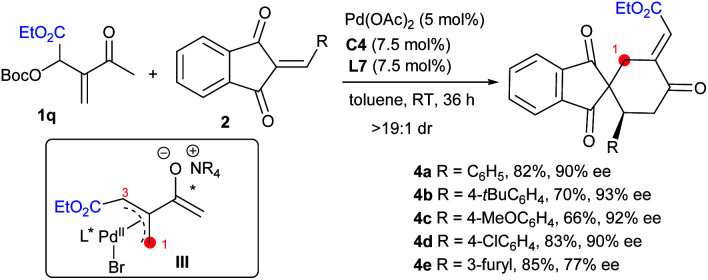
Regiodivergent [4 + 2] annulations between MBH carbonate **1q** and alkenes **2**.

Remarkably, the MBH carbonate **1q** derived from ethyl glyoxalate and MVK was compatible as well in the reactions with alkenes **2** under the standard conditions, and other types of [4 + 2] products **4a–4e** with different regioselectivity were furnished, by switching the allylic alkylation step from **C3** to **C1** (Scheme 2, intermediate **III**), and probably the electronic effect of the substituents played a key role in the regioselectivity.^10^

### Substrate scope of asymmetric diastereodivergent [4 + 2] annulations

**Scheme 3:**
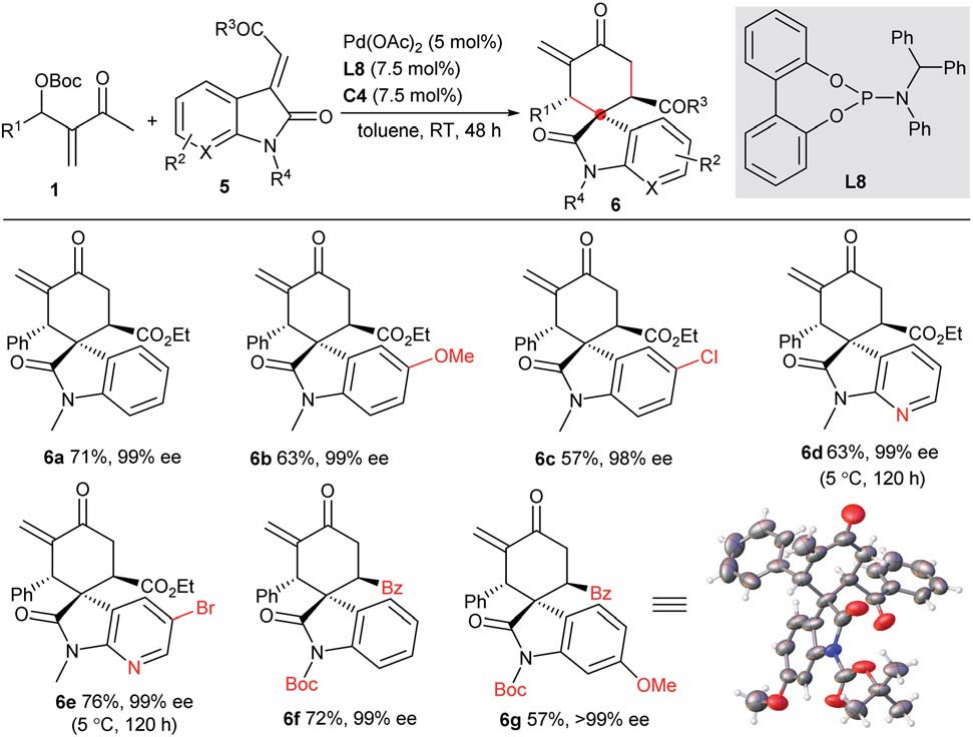
Asymmetric diastereodivergent [4 + 2] annulations between MBH carbonates **1** and 3-vinyl oxindoles **5**.

It is interesting but challenging to switch the diastereoselectivity of the reactions by simply tuning the conditions, as chiral compound libraries with higher stereodiversity could be efficiently obtained from identical starting materials.^17^ Considering that two independent chiral sources were involved in the current catalytic system, we assumed that the diastereodivergent [4 + 2] annulations of MBH carbonates **1** might be realised. Although we failed to construct different diastereomers with 2-alkylidene indene-1,3-diones **2**, 3-vinyl oxindoles **5** possessing an ester or a benzoyl group could successfully act as the counterparts in the switchable diastereodivergent [4 + 2] annulations. Under the catalysis of Pd(OAc)_2_, achiral ligand **L8** and IPC **C4**, the major diastereomers **6a–6g**, having three contiguous stereogenic centres, were generally separated directly in moderate yields with excellent enantioselectivity, as summarised in Scheme 3, though fair to moderate diastereocontrol was observed.^10^

**Scheme 4:**
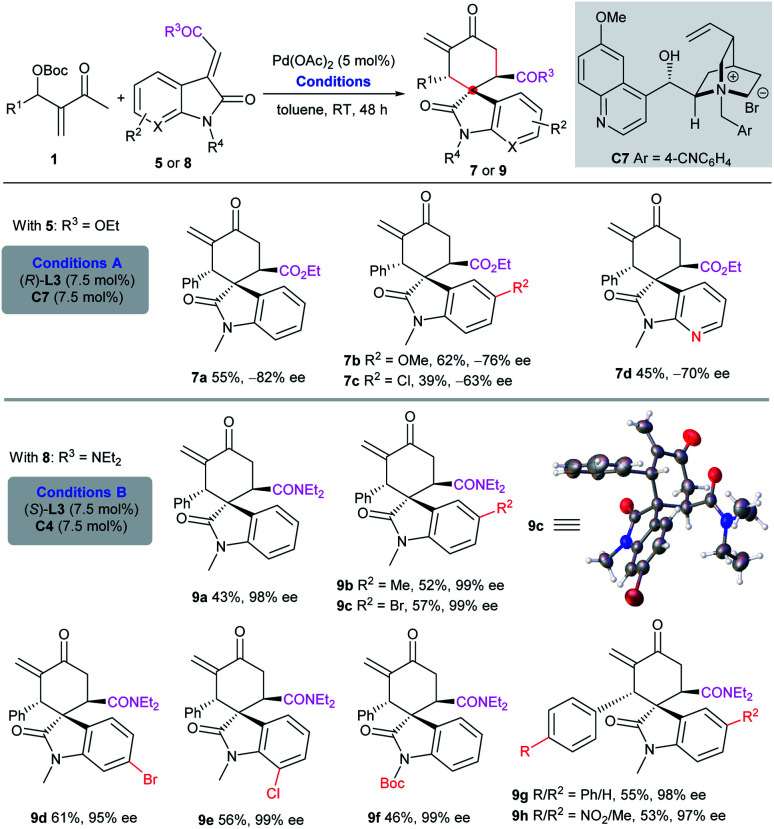
Asymmetric diastereodivergent [4 + 2] annulation by tuning the catalytic system or substrate.

In contrast, the assembly of Pd(OAc)_2_, ligand (*R*)-**L3** and chiral IPC C7 could switch the diastereoselectivity, and afford separable diastereomers **7a–7d** in moderate yields and enantioselectivity (Scheme 4, conditions A). Although we could not achieve more satisfactory results by screening diverse catalyst combinations,^10^ it was found that the substitution pattern of the acceptors also had a dramatic effect on the outcome. Almost enantiopure diastereomers **9a–h** were effectively isolated in moderate yields, by employing amide substrates **8** under the catalysis of Pd(OAc)_2_, ligand (*S*)-**L3** and chiral IPC **C4**, also demonstrating the flexibility and tunability of the current double activation catalytic system (Scheme 4, conditions B).^10^

### Substrate scope of asymmetric oxa-[4 + 2] annulations

Besides activated alkenes, activated ketones, such as *N*-protected isatins **10**,^18^ were applicable as two-atom partners in the asymmetric oxa-[4 + 2] annulations with MBH carbonate **1a**. As summarised in Table 3, the assembly of Pd(OAc)_2_, ligand **L7** and IPC **C8** proved to be a reliable catalytic system, giving spiro[tetrahydropyran-3,3′-oxindole] **11a–11h** in moderate to good yields with good to high enantioselectivity (Table 3, entries 1–8), even on a 1.0 mmol scale (entry 5).^10^

**Table 3:** Asymmetric oxa-[4 + 2] annulations between MBH carbonate **1a** and isatins **10**^a^

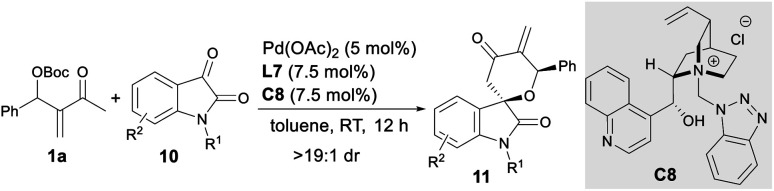				
Entry	R^1^	R^2^	Yield^b^ (%)	ee^c^ (%)
1	Me	H	**11a**, 62	87
2	Me	5-Me	**11b**, 72	90
3	Me	5-Cl	**11c**, 85	84
4	Me	6-Br	**11d**, 62	84
5	Me	7-Me	**11e**, 81 (72)	92^d^ (91)
6	Me	7-Cl	**11f**, 67	80
7	Bn	H	**11g**, 78	80
8	Bn	5-Cl	**11h**, 65	80

a Unless otherwise noted, reactions were carried out with MBH carbonate **1** (0.12 mmol), isatin **10** (0.10 mmol), Pd(OAc)_2_ (5 mol%), **C8** (7.5 mol%) and **L7** (7.5 mol%) in toluene (1.0 mL) at rt for 12 h under Ar.

b Isolated yields.

c Determined by HPLC analysis on a chiral stationary phase; dr > 19 : 1 by ^1^H NMR analysis.

d Data in parentheses were obtained on a 1.0 mmol scale. The absolute configuration of **11e** was determined by X-ray analysis after conversion to enantiopure **14** (Scheme 5). The other products were assigned by analogy.

### Transformations of the [4 + 2] annulation products

The enone moiety in the products enables further transformations to access the products with higher molecular complexity. As illustrated in Scheme 5, the *in situ* generated 1,3-dipole from *N*-phenylbenzohydrazonoyl chloride underwent highly diastereoselective [3 + 2] cycloaddition with **3a**,^19^ affording the spirocyclic framework **12** in an excellent yield. In addition, the heterocycle **13** could be efficiently constructed with a retained ee value from **3a***via* a [4 + 2] annulation with malononitrile. In addition, an interesting dimerisation of **11e***via* an oxa-Diels–Alder reaction gave polycyclic product **14** with excellent diastereoselectivity and enantioselectivity.^20^

**Scheme 5:**
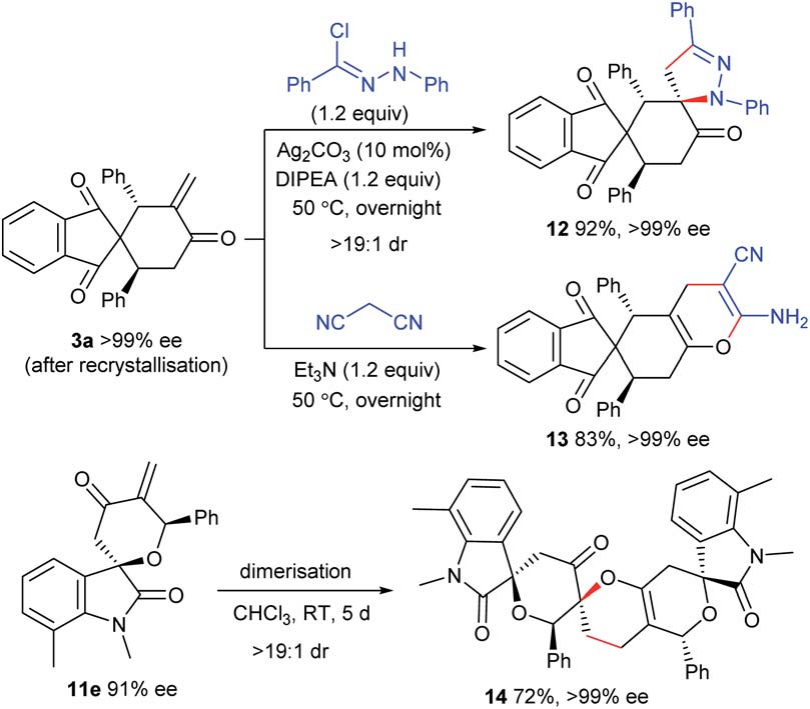
Synthetic transformations of the products.

### Mechanism studies

**Scheme 6:**
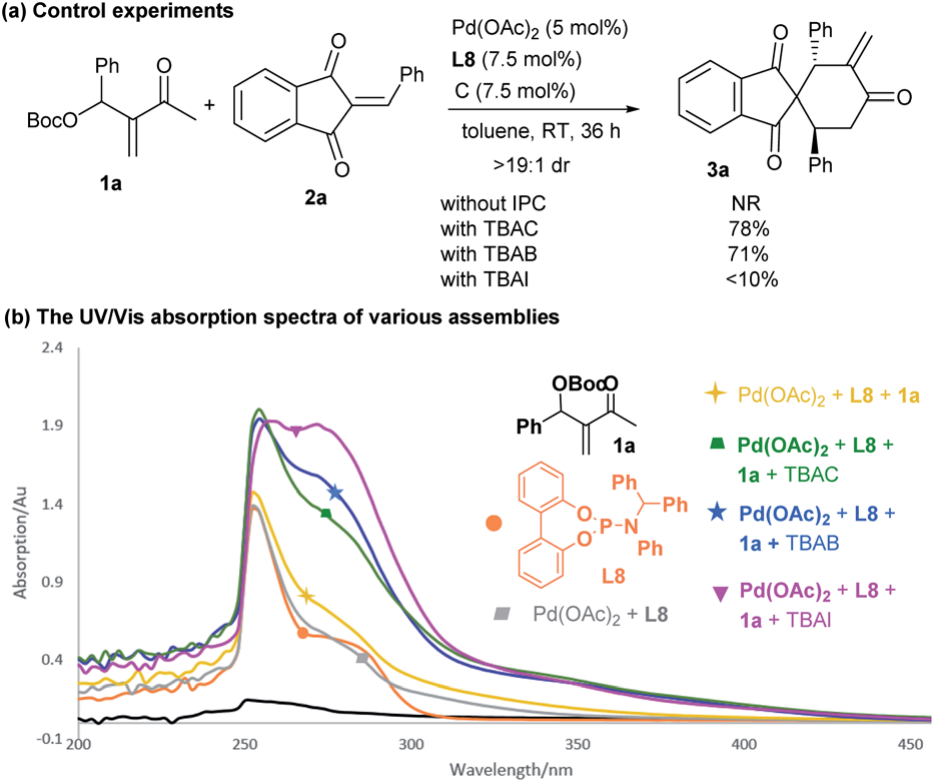
Control experiments with diverse IPCs and UV-Vis absorption analysis.

To gain some insight into the catalytic mechanism, several control experiments were further conducted. As summarised in Scheme 6a, the combination of Pd(OAc)_2_ and achiral phosphoramidite **L8** failed to promoted the reaction of **1a** and **2a** in the absence of an IPC. In contrast, adding either TBAC or TBAB significantly enhanced the desired annulation reaction, whereas the iodide salt (TBAI) was less efficient, probably due to the coordinating effects of different halides.^12,21^ While we were not able to obtain some valuable information by ^1^H NMR or mass spectrometry analysis, we carried out UV-Vis absorption experiments to monitor the complexation process.^22^ As outlined in Scheme 6b, a slightly different absorption spectrum was observed after adding MBH carbonate **1a** into the mixture of Pd(OAc)_2_ and **L8**. In contrast, apparent absorption changes were detected by further adding diverse ammonium halides. In particular, much stronger absorption in the 320–380 nm area was observed, indicating that some new complex species would be generated from the previously formed dipole **I** or ligated intermediate **II** (Scheme 1c) and an ammonium halide.

**Scheme 7:**
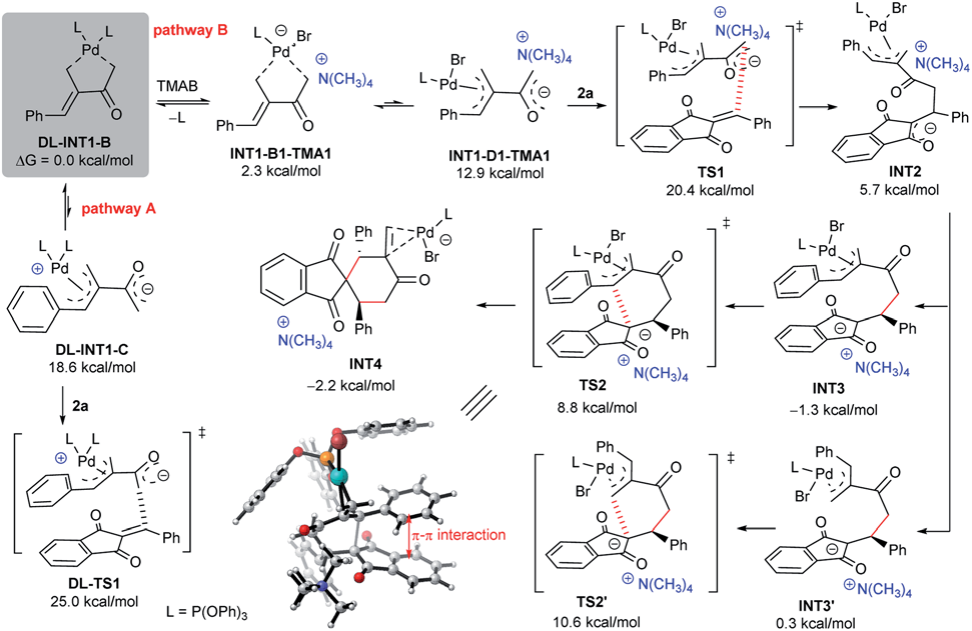
DFT calculations on the proposed catalytic mechanism, at the B3LYP-D3/6-311++G(d,p)//B3LYP-D3/6-31(d) SDD for the Pd (toluene) level.

In order to further rationalise the role of ammonium halides, the mechanism of the catalytic reaction was studied by density functional theory (DFT) calculations. To simplify the process, P(OPh)_3_ was selected as the ligand, since it gave similar results to those of phosphoramidite **L8**,^10^ and simple Me_4_NBr (TMAB) was employed as the PTC partner. As outlined in Scheme 7, after the oxidative insertion by Pd(0) and deprotonation, the five-membered ligated intermediate **DL-INT1-B** was found to be the more stable intermediate.^10^ The reactive 1,4-carbodipole **DL-INT1-C** had a much higher energy (18.6 kcal mol^−1^), and the subsequent addition to acceptor **2a***via***DL-TS1** had an energy barrier of 25.0 kcal mol^−1^, which was found to be inert at room temperature (pathway A). In contrast, a ligand exchange of **DL-INT1-B** would easily occur after adding TMAB, giving intermediate **INT1-B1-TMA1** (2.3 kcal mol^−1^) (pathway B).^23^ Moreover, after isomerisation to active ion-pair **INT1-D1-TMA1** (12.9 kcal mol^−1^) having an electronically neutral Pd(ii) complex, the subsequent Michael addition to **2a***via***TS1** had an energy barrier of 20.4 kcal mol^−1^, which was significantly lower than that of **DL-TS1**. The resultant **INT2** would be easily converted to the ion-pairing intermediate **INT3**, which would undergo allylic substitution *via***TS2** with an energy barrier of 8.8 kcal mol^−1^, finally affording the intermediate **INT4**. We also compared the possible formation of a regioisomer *via***TS2′** from ion-pair **INT3′**, which had a higher energy barrier of 10.6 kcal mol^−1^, also in good accordance with a predicted ratio (21 : 1). An obvious π–π interaction between the aromatic rings of **1a** and **2a** was observed in **TS2** (Scheme 7), which might be one of the reasons for the reduction of the energy and the formation of regioisomer **3a**.^10^

## Conclusions

We have developed a new palladium/ammonium halide double activation catalytic system, which could facilitate the conversion of possible π-allylpalladium complex-based 1,4-carbodipoles from Morita–Baylis–Hillman (MBH) carbonates of vinyl methyl ketone to more reactive compact ion-pair species. Both palladium complex and ammonium halide components were easily tunable, which would be helpful for enhancing reactivity and stereocontrol on both nucleophilic and electrophilic sites of the newly formed intermediates in a cooperative manner. The catalytic efficacy of such a transition metal and organic ion-pair system has been well demonstrated in asymmetric [4 + 2] annulations of substantial MBH carbonates and 2-alkylidene indene-1,3-diones, affording densely functionalised spirocyclic compounds^16^ in moderate to good yields with excellent stereoselectivity, and even regioselective annulations could be switched by tuning the substitutions of the MBH carbonates. Furthermore, switchable diastereodivergent [4 + 2] annulations between MBH carbonates and 3-olefinic oxindoles have also been realised by modifying the flexible assembly of the palladium complex and ion-pairing agent. The catalytic system has been further utilised in asymmetric oxa-[4 + 2] annulations between MBH carbonates and isatins, affording an array of spiro[tetrahydropyran-3,3′-oxindole] architectures. Moreover, UV-Vis absorption experiments and DFT calculations have been conducted to elucidate the catalytic mechanism, especially for the possible role of ammonium halides. We believe that the flexible double activation system combining a palladium complex and an ion-pair catalyst would find more application in diverse asymmetric reactions.

## Data availability

All experimental procedures, characterization, computational data, copies of NMR and HRMS spectra for all new compounds and HPLC chromatograms related to this study, can be found in the ESI.

## Author contributions

The manuscript was written through contributions of all authors. All authors have given approval to the final version of the manuscript.

## Conflicts of interest

There are no conflicts to declare.

## Supplementary Material

SC-012-D1SC03517G-s001

SC-012-D1SC03517G-s002
