# Supplementary material for: Combining palladium and ammonium halide catalysts for Morita–Baylis–Hillman carbonates of methyl vinyl ketone: from 1,4-carbodipoles to ion pairs
Source: Chem Sci. 2021 Jul 22;12(34):11399–405. doi: 10.1039/d1sc03517g (PMC8447884; doi:10.1039/d1sc03517g)
Supplement: SC-012-D1SC03517G-s001 [file SC-012-D1SC03517G-s001.pdf]

Electronic Supplementary Material (ESI) for Chemical Science.
This journal is © The Royal Society of Chemistry 2021

Combining palladium and ammonium halide catalysts for Morita–Baylis–Hillman carbonates of methyl vinyl ketone: From 1,4-carbodipoles to ion pairs

Yang Yang,[a] Bo Zhu,[a] Lei Zhu,[b] Ying Jiang,[a] Chun-Ling Guo,[b] Jing Gu,[b] Qin Ouyang,*[b] Wei Du,*[a] and Ying-Chun Chen*[a,b]

[a]Key Laboratory of Drug-Targeting and Drug Delivery System of the Ministry of Education and Sichuan Research Center for Drug Precision Industrial Technology, West China School of Pharmacy, Sichuan University, Chengdu 610041, China

[b]College of Pharmacy, Third Military Medical University, Shapingba, Chongqing 400038, China

E-mail: ouyangq@tmmu.edu.cn; duweiyb@scu.edu.cn; ycchen@scu.edu.cn.

Supplementary Information

Table of Contents

1. General methods S3
2. Procedures for the synthesis of IPC **C4** and bifunctional ligand **L4** S4
3. Procedures for the synthesis of (*E*)-N,N-diethyl-2-(1-methyl-2-oxoindolin-3-ylidene)acetamides S6
4. Condition optimisations S8
   4.1 Diverse phosphine ligands investigated in the asymmetric [4+2] annulation with MBH carbonate 1a and α-benzylidene-1*H*-indene-1,3(2*H*)-dione **2a** S8
   4.2 Investigation of bifunctional ligands containing an ammonium moiety S9
   4.3 Diverse control experiments S10
   4.4 Screenings with diverse phosphoramidite ligands S11
   4.5 Solvent screenings S12
   4.6 Screening conditions for asymmetric [4+2] annulations involving 3-olefinic oxindoles S13
   4.7 Screening conditions for asymmetric oxa-[4+2] annulation S15
5. General procedure for asymmetric [4+2] annulations S16
   5.1 Asymmetric [4+2] annulations involving α-alkylidene-1*H*-indene-1,3(2*H*)-diones S16
   5.2 Regiodivergent asymmetric [4+2] annulations with MBH carbonate **1q** S32
   5.3 Diastereodivergent asymmetric [4+2] annulations involving 3-olefinic oxindoles S36
   5.4 Asymmetric [4+2] annulations involving isatins S50

5.5 Transformations of the [4+2] annulation product **3a** S55
5.6 Dimerisation of 11e S56
6. Crystal data for enantiopure products **3s**, **6g**, **9c** and **14** S57
7. More unsuccessful attempts S63
8. Bioactivity test S64
8.1 Biological results S64
8.2 Biological evaluation methods S65
9. Mechanism study S66
9.1 Control experiments S66
9.2 UV-Vis absorption analysis S67
10. DFT calculations S85
10.1 Coordination model S85
10.2 Reaction process S88
10.3 The regioselectivity S90
12. NMR, HRMS spectra and HPLC chromatograms S93
13. Computational methods and data S269
14. References S346

1. General methods

When the reactions required heating, the heat source was oil bath. ^{1}H NMR (400 or 600 MHz), ^{13}C NMR (100 or 150 MHz) spectra were recorded on Varian INOVA-400/54, Agilent DD2-600/54 or Bruker Ascend^{TM} 400 instruments (Chemical shifts were reported in ppm from tetramethylsilane with the solvent resonance as the internal standard in $CDCl_3$ solution, unless otherwise noted). The following abbreviations were used to explain the multiplicities: s = singlet, d = doublet, t = triplet, dd = double doublet, dt = double triplet; td = triple doublet; m = multiplet, br = broad, and coupling constants (*J*) are reported in Hertz (Hz). High resolution mass spectra (HRMS) were recorded on a Waters SYNAPT G2, Agilent G1969-85000 or Shimadzu LCMS-IT-TOF using a time-of-flight mass spectrometer equipped with electrospray ionization (ESI) source. X-ray diffraction experiments were carried out on an Agilent Gemini or Bruker APEX-II CCD diffractometer. Ultraviolet-visible spectra were recorded on a GENESYS 180 using ethyl acetate as the solvent. In each case, diastereomeric ratio was determined by ^{1}H NMR or HPLC analysis and enantiomeric excess was determined by HPLC analysis on a chiral stationary phase, using a Daicel Chiralpak IA Column (250 × 4.6 mm), Chiralpak Column IB (250 × 4.6 mm), Chiralpak Column IC (250 × 4.6 mm), Chiralpak Column ID (250 × 4.6 mm), Chiralpak Column IE (250 × 4.6 mm), Chiralpak Column IF (250 × 4.6 mm) or Chiralpak AD-H Column (250 × 4.6 mm), Chiralcel OD-H Column (250 × 4.6 mm). UV detection was monitored at 254 nm. Optical rotation was measured in CH_2Cl_2 or $CHCl_3$ solution at 25 °C. Column chromatography was performed on silica gel (200-300 mesh) eluting with ethyl acetate (EtOAc) or acetone and petroleum ether. TLC was performed on glass-backed silica plates. UV light, I_2, solution of potassium permanganate were used to visualize products or the starting materials. All chemicals were used without purification as commercially available unless otherwise noted. Petroleum ether and EtOAc were distilled. Methyl vinyl ketone derived MBH carbonates **1**,[1] 2-alkylidene-1*H*-indene-1,3(2*H*)-diones **2**,[2] 3-olefinic oxindoles **5**,[3] (*E*)-N,N-diethyl-2-(1-methyl-2-oxoindolin-3-ylidene)acetamides **8**[4] were prepared according to the literature procedures.

2. Procedures for the synthesis of IPC C4 and bifunctional ligand L4

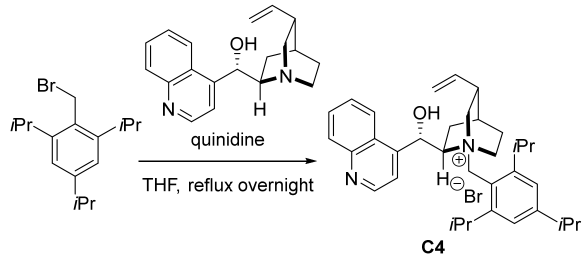

C4
8% yield

Benzyl bromide (0.71 g, 2.4 mmol) and cinchonine (0.59 g, 2.0 mmol) were dissolved in dry THF (8 mL). The mixture was refluxed overnight under Ar, and then cooled to room temperature. It was concentrated in vacuum, and purified by column chromatography (DCM/MeOH = 80/1–33/1) to give the desired ion-pair catalyst **C4**, as a white solid in 8% yield (95 mg). *The yield is low, mainly due to the steric hindrance of 2,4,6-triisopropyl benzyl bromide reagent which might make the formation of quaternary ammonium salt difficult. In addition, the attack by the N atom of quinoline ring also might occur, and a few by-products were obviously observed.*

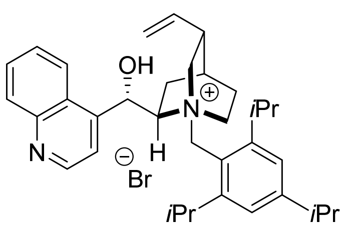

C4

(1*R*,2*R*,4*S*,5*R*)-2-((*S*)-Hydroxy(quinolin-4-yl)methyl)-1-(2,4,6-triisopropylbenzyl)-5-vinylquinuclidin-1-ium bromide (C4): White solid, mp: 133–135 °C; $[\alpha]_D^{25}$ = +80.0 (*c* = 0.08, $CHCl_3$); **^{1}H NMR** (400 MHz, $CDCl_3$): δ (ppm) 8.96 (d, *J* = 4.5 Hz, 1H), 8.24–8.12 (m, 1H), 8.07 (d, *J* = 8.4 Hz, 1H), 7.96 (d, *J* = 4.5 Hz, 1H), 7.74 (ddd, *J* = 8.2, 6.8, 1.2 Hz, 1H), 7.64 (ddd, *J* = 8.2, 6.8, 1.4 Hz, 1H), 7.20 (d, *J* = 1.9 Hz, 1H), 7.13 (d, *J* = 1.9 Hz, 1H), 7.04–7.02 (m, 1H), 6.97 (d, *J* = 5.8 Hz, 1H), 6.15 (d, *J* = 14.0 Hz, 1H), 6.11–6.03 (m, 1H), 5.28 (d, *J* = 10.3 Hz, 1H), 5.22 (d, *J* = 17.1 Hz, 1H), 5.01 (d, *J* = 13.8 Hz, 1H), 4.73–4.64 (m, 1H), 3.81 (t, *J* = 9.4 Hz, 1H), 3.73–3.66 (m, 1H), 3.56–3.26 (m, 3H), 3.00–2.90 (m, 1H), 2.81–2.73 (m, 1H), 2.66–2.54 (m, 1H), 2.51–2.39 (m, 1H), 1.94 (s, 1H), 1.88–1.70 (m, 2H), 1.41 (d, *J* = 6.6 Hz, 3H), 1.37 (d, *J* = 6.6 Hz, 3H), 1.34 (d, *J* = 6.5 Hz, 3H), 1.30–1.27 (m, 9H), 1.10–0.99 (m, 1H); **^{13}C NMR** (100 MHz, $CDCl_3$): δ (ppm) 152.5, 151.9, 150.5, 150.1, 148.1, 144.9, 135.7, 131.0, 129.2, 127.2, 124.6, 123.7, 122.4, 122.1, 120.6, 118.5, 117.8, 69.8, 64.4, 56.8, 55.2, 55.1, 38.9, 34.2, 31.7, 30.6, 28.0, 26.75, 26.68, 24.6, 23.8, 23.7, 22.9, 22.4, 21.7; **HRMS** (ESI-TOF) m/z: $[M - Br]^+$ Calcd for $C_{35}H_{47}N_2O^+$ 511.3683; Found 511.3686.

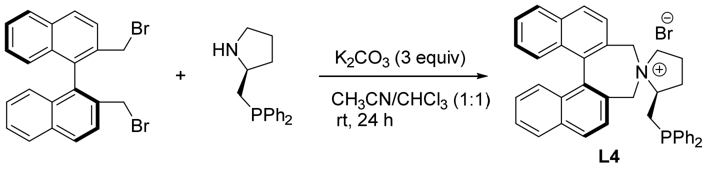

To a mixture of (*S*)-2-((diphenylphosphanyl)methyl)pyrrolidine (54 mg, 0.20 mmol) and K_2CO_3 (83 mg, 0.60 mmol) in CH_3CN (1 mL) and $CHCl_3$ (1 mL) was added (*S*)-2,2'-bis(bromomethyl)-1,1'-binaphthalene (88 mg, 0.20 mmol) at room temperature under Ar. After 24 h, the solution was concentrated and was purified by column chromatography on silica gel ($CHCl_3$/MeOH = 20/1 as eluent) to afford the desired product **L4** (89 mg, 71% yield) as a white solid.

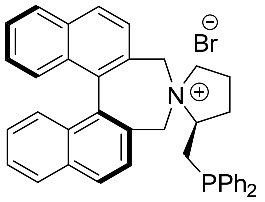

(2'*S*,11*bS*)-2'-((Diphenylphosphanyl)methyl)-3,5-dihydrospiro [dinaphtho[2,1-*c*:1',2'-e]azepine-4,1'-pyrrolidin]-4-ium bromide (L4). White solid, mp: 133–135 °C; $[\alpha]_D^{25}$ = +93.3 (*c* = 0.21, $CHCl_3$); **^{1}H NMR** (600 MHz, $CDCl_3$): δ (ppm) 8.08–8.00 (m, 2H), 8.00 (d, *J* = 3.0 Hz, 1H), 7.99 (d, *J* = 3.6 Hz, 1H), 7.95 (d, *J* = 8.4 Hz, 1H), 7.90 (d, *J* = 8.4 Hz, 1H), 7.59 (t, *J* = 7.4 Hz, 1H), 7.54–7.51 (m, 1H), 7.43–7.29 (m, 7H), 7.29–7.16 (m, 7H), 5.11 (d, *J* = 14.0 Hz, 1H), 4.80 (d, *J* = 12.5 Hz, 1H), 4.40 (q, *J* = 10.4 Hz, 1H), 4.20 (d, *J* = 12.5 Hz, 1H), 4.00–3.93 (m, 1H), 3.61 (d, *J* = 14.0 Hz, 1H), 3.26 (t, *J* = 9.9 Hz, 1H), 2.77 (t, *J* = 12.2 Hz, 1H), 2.54–2.49 (m, 1H), 2.40 (dd, *J* = 12.2, 2.6 Hz, 1H), 2.38–2.33 (m, 1H), 2.28–2.22 (m, 1H), 2.14–2.06 (m, 1H); **^{13}C NMR** (150 MHz, $CDCl_3$): δ (ppm) 136.9, 136.6 (d, *J* = 12.1 Hz), 136.1 (d, *J* = 13.3 Hz), 135.1, 134.3, 134.0, 132.8, 132.7, 132.5, 131.3, 131.0, 130.5, 129.6, 129.4, 129.3, 128.93, 128.88, 128.8, 128.7, 128.6, 128.5, 128.4, 127.9, 127.8, 127.7, 127.6, 127.33, 127.30, 127.1, 127.00, 126.98, 75.2 (d, *J* = 25.7 Hz), 65.2, 60.8, 58.9, 30.7 (d, *J* = 18.6 Hz), 28.5 (d, *J* = 7.0 Hz), 19.1; **HRMS** (ESI-TOF) m/z: $[M - Br]^+$ Calcd for $C_{39}H_{35}NP^+$ 548.2502 (^{31}P) and 549.2535 (^{32}P); Found 548.2503 (^{31}P) and 549.2543 (^{32}P).

3. Procedures for the synthesis of (*E*)-N,N-diethyl-2-(1-methyl-2-oxoindolin-3-ylidene)acetamides

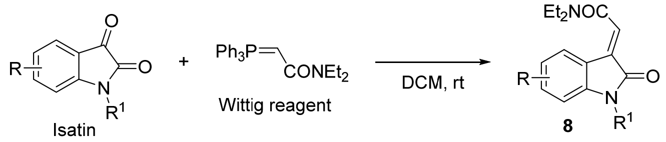

A solution of isatin (2.0 mmol) and Wittig reagent in dry DCM (8 mL) was stirred at room temperature for 1–2 h. After completion, the solvent was concentrated in vacuum, and the residue was purified by column chromatography on silica gel (petroleum ether/EtOAc = 6/1–3/1) to give the desired product.

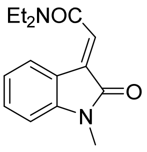

(*E*)-N,N-Diethyl-2-(1-methyl-2-oxoindolin-3-ylidene)acetamide (8a): Yellow solid, mp: 91–92 °C; **^{1}H NMR** (400 MHz, $CDCl_3$): δ (ppm) 7.3 (J = 7.7 Hz, 1H), 7.31 (td, J = 7.7, 1.1 Hz, 1H), 7.22 (s, 1H), 7.00 (td, J = 7.7, 1.1 Hz, 1H), 6.79 (d, J = 7.7 Hz, 1H), 3.56 (q, J = 7.1 Hz, 2H), 3.40 (q, J = 7.1 Hz, 2H), 3.24 (s, 3H), 1.26 (t, J = 7.1 Hz, 3H), 1.17 (t, J = 7.1 Hz, 3H); **^{13}C NMR** (100 MHz, $CDCl_3$): δ (ppm) 167.6, 165.3, 144.7, 132.6, 131.1, 126.1, 125.7, 122.7, 120.1, 108.1, 42.8, 39.8, 26.2, 14.6, 13.1; **HRMS** (ESI-TOF) m/z: $[M + Na]^+$ Calcd for $C_{15}H_{18}N_2O_2Na^+$ 281.1260; Found 281.1260.

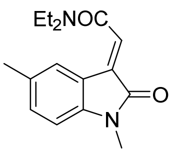

(*E*)-2-(1,5-Dimethyl-2-oxoindolin-3-ylidene)-N,N-diethylacetamide (8b): Yellow solid, mp: 75–76 °C; **^{1}H NMR** (400 MHz, $CDCl_3$): δ (ppm) 7.64(s, 1H), 7.19 (s, 1H), 7.11 (dd, J = 7.8, 0.9 Hz, 1H), 6.68 (d, J = 7.8 Hz, 1H), 3.57 (q, J = 7.1 Hz, 2H), 3.40 (q, J = 7.1 Hz, 2H), 3.21 (s, 3H), 2.30 (s, 3H), 1.27 (t, J = 7.1 Hz, 3H), 1.17 (t, J = 7.1 Hz, 3H); **^{13}C NMR** (100 MHz, $CDCl_3$): δ (ppm) 167.6, 165.4, 142.5, 132.8, 132.1, 131.4, 126.3, 125.8, 120.1, 107.9, 42.8, 39.8, 26.2, 21.1, 14.6, 13.0; **HRMS** (ESI-TOF) m/z: $[M + Na]^+$ Calcd for $C_{16}H_{21}N_2O_2Na^+$ 295.1417; Found 295.1420.

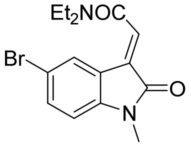

(*E*)-2-(5-Bromo-1-methyl-2-oxoindolin-3-ylidene)-N,N-diethylacetamide (8c): Yellow solid, mp: 105–107 °C; **^{1}H NMR** (400 MHz, $CDCl_3$): δ (ppm) 7.81 (d, J = 8.1 Hz, 1H), 7.25 (s, 1H), 7.14 (dd, J = 8.1, 1.8 Hz, 1H), 6.95 (d, J = 1.8 Hz, 1H),

3.54 (q, *J* = 7.1 Hz, 2H), 3.41 (q, *J* = 7.1 Hz, 2H), 3.22 (s, 3H), 1.25 (t, *J* = 7.1 Hz, 3H), 1.18 (t, *J* = 7.1 Hz, 3H); **^{13}C NMR** (100 MHz, $CDCl_3$): *δ* (ppm) 167.5, 164.9, 145.9, 132.2, 127.3, 126.4, 125.5, 125.2, 118.9, 111.7, 42.9, 40.1, 26.3, 14.7, 13.1; **HRMS** (ESI-TOF) m/z: $[M + H]^+$ Calcd for $C_{15}H_{18}N_2O_2Br^+$ 337.0546 (^{79}Br) and 339.0526 (^{81}Br); Found 337.0545 (^{79}Br) and 339.0525 (^{81}Br).

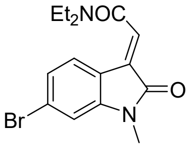

(*E*)-2-(6-Bromo-1-methyl-2-oxoindolin-3-ylidene)-N,N-diethylacetamide (8d): Yellow solid, mp: 105−107 °C; **^{1}H NMR** (400 MHz, $CDCl_3$): *δ* (ppm) 7.80 (d, *J* = 8.1 Hz, 1H), 7.25 (s, 1H), 7.14 (dd, *J* = 8.1, 1.7 Hz, 1H), 6.95 (d, *J* = 1.7 Hz, 1H), 3.54 (q, *J* = 7.1 Hz, 2H), 3.41 (q, *J* = 7.1 Hz, 2H), 3.22 (s, 3H), 1.24 (t, *J* = 7.1 Hz, 3H), 1.18 (t, *J* = 7.1 Hz, 3H); **^{13}C NMR** (100 MHz, $CDCl_3$): *δ* (ppm) 167.5, 164.9, 145.9, 132.2, 127.3, 126.4, 125.6, 125.2, 118.9, 111.7, 42.9, 40.1, 26.3, 14.7, 13.1; **HRMS** (ESI-TOF) m/z: $[M + Na]^+$ Calcd for $C_{15}H_{17}N_2O_2NaBr^+$ 359.0366 (^{79}Br) and 361.0345 (^{81}Br); Found 359.0371 (^{79}Br) and 361.0352 (^{81}Br).

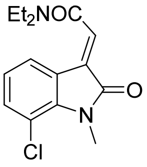

(*E*)-2-(7-Chloro-1-methyl-2-oxoindolin-3-ylidene)-N,N-diethylacetamide (8e): Yellow solid, mp: 95–96 °C; **^{1}H NMR** (400 MHz, $CDCl_3$): *δ* (ppm) 7.73 (dd, *J* = 7.6, 1.2 Hz, 1H), 7.26 (s, 1H), 7.23 (dd, *J* = 8.2, 1.2 Hz, 1H), 6.90 (dd, *J* = 8.2, 7.6 Hz, 1H), 3.62 (s, 3H), 3.55 (q, *J* = 7.1 Hz, 2H), 3.38 (q, *J* = 7.1 Hz, 2H), 1.25 (t, *J* = 7.1 Hz, 3H), 1.15 (t, *J* = 7.1 Hz, 3H); **^{13}C NMR** (100 MHz, $CDCl_3$): *δ* (ppm) 167.8, 165.0, 140.4, 133.1, 131.2, 127.6, 124.0, 123.4, 122.7, 115.7, 42.8, 39.8, 29.7, 14.6, 13.0; **HRMS** (ESI-TOF) m/z: $[M + Na]^+$ Calcd for $C_{15}H_{17}N_2O_2NaCl^+$ 315.0871 (^{35}Cl) and 317.0841 (^{37}Cl); Found 315.0883 (^{35}Cl) and 317.0861 (^{37}Cl).

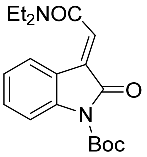

***tert*-Butyl (*E*)-3-(2-(diethylamino)-2-oxoethylidene)-2-oxoindoline-1-carboxylate (8f):** Yellow solid, mp: 56–57 °C; **^{1}H NMR** (400 MHz, $CDCl_3$): *δ* (ppm) 7.91–7.85 (m, 2H), 7.36 (td, *J* = 8.1, 1.2 Hz, 1H), 7.23 (s, 1H), 7.13 (td, *J* = 7.7, 1.2 Hz, 1H), 3.56 (q, *J* = 7.1 Hz, 2H), 3.37 (q, *J* = 7.1 Hz, 2H), 1.65 (s, 9H), 1.26 (t, *J* = 7.1 Hz, 3H), 1.15 (t, *J* = 7.1 Hz, 3H); **^{13}C NMR** (100 MHz, $CDCl_3$): *δ* (ppm) 165.9, 165.0, 149.1, 140.6, 131.3, 131.1, 127.2, 125.2, 124.5, 120.2, 115.1, 84.6, 42.8, 39.8, 28.1, 14.6, 13.0; **HRMS** (ESI-TOF) m/z: $[M + H]^+$ Calcd for $C_{19}H_{25}N_2O_4^+$ 345.1809; Found 345.1811.

4. Condition optimisations

4.1 Diverse phosphine ligands investigated in the asymmetric [4+2] annulation with MBH carbonate 1a and α-benzylidene-1*H*-indene-1,3(2*H*)-dione 2a

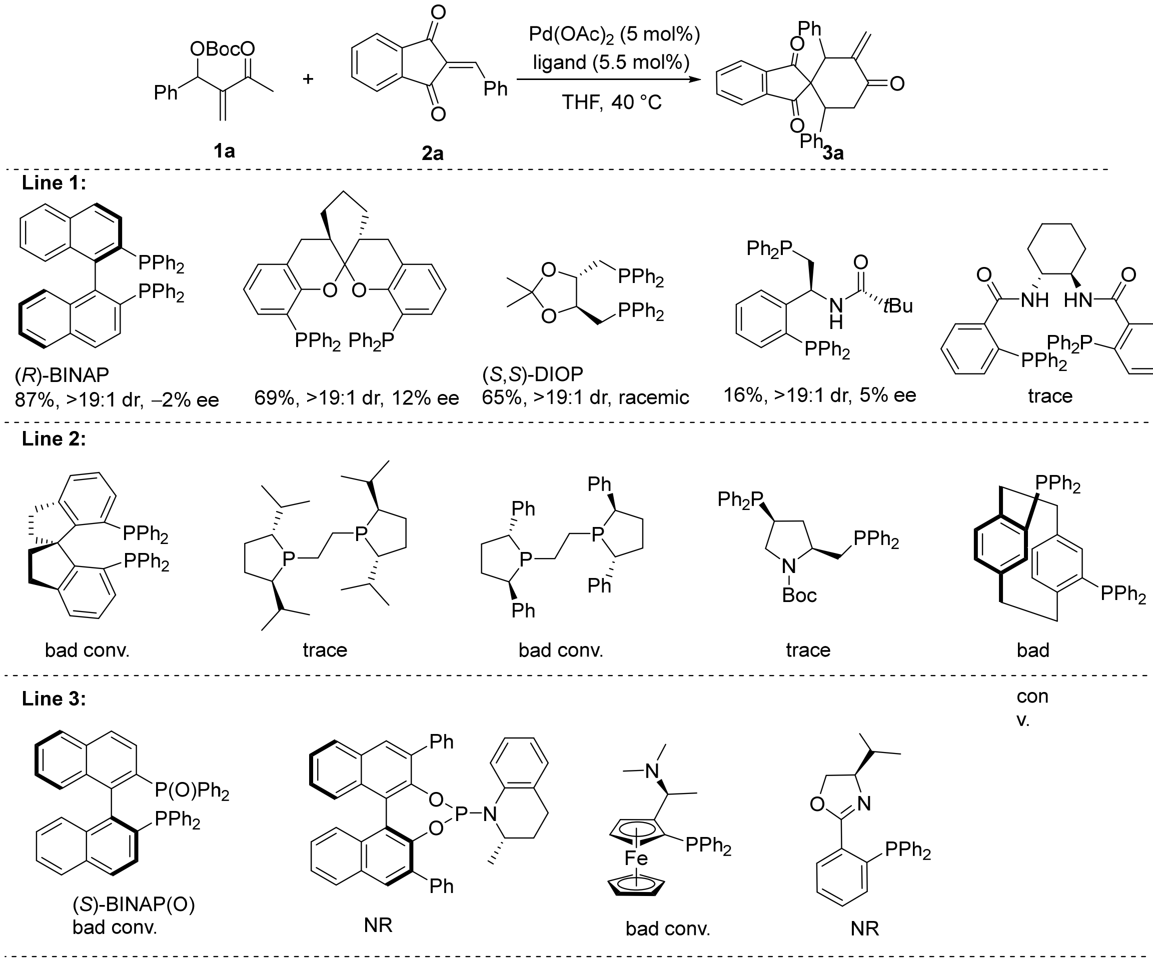

Scheme S1 Diverse phosphine ligands screened

The reaction was conducted with MBH carbonate **1a** (0.75 mmol), enone **2a** (0.05 mmol), $Pd(OAc)_2$ (0.0025 mmol) and ligand (0.00275 mmol) in dry THF (0.5 mL) under Ar, and the mixture was stirred at 40 °C for 36 h.

As outlined in Scheme **S1**, several chiral phosphine ligands were investigated. Most ligands could not promote this [4+2] annulation reaction in combination with $Pd(OAc)_2$. A few bisphosphine ligands, such as BINAP, showed good catalytic activity, but the enantioselectivity was very poor, indicating that the chiral Pd-complex could not well control the selectivity of the remote addition reaction.

4.2 Investigation of bifunctional ligands containing an ammonium moiety

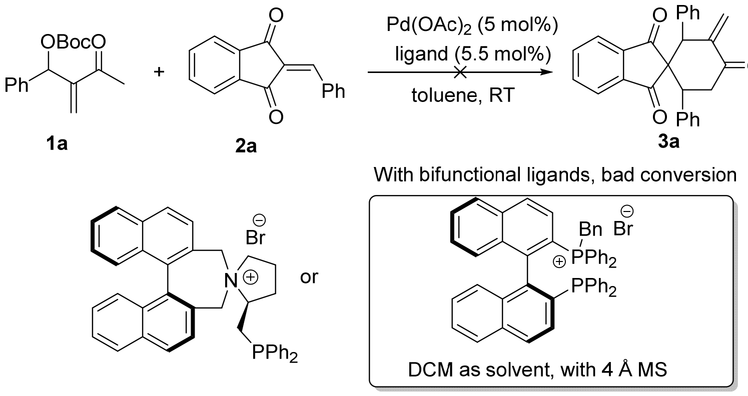

Scheme S2 Bifunctional phosphine ligands screened

The reaction was conducted with MBH carbonate **1a** (0.06 mmol), enone **2a** (0.05 mmol), $Pd(OAc)_2$ (0.0025 mmol) and bifunctional ligand (0.00275 mmol) in dry toluene (0.5 mL) under Ar, and the mixture was stirred at room temperature.

As outlined in Scheme **S2**, the phosphine ligands containing an ammonium or a phosphonium moiety, similar to the ligand developed by Ooi (*Nat. Chem.* **2014**, *6*, 47), were investigated. Unfortunately, no reaction occurred, probably due to the improper assembly of the substrate and the corresponding Pd-complex.

4.3 Diverse control experiments

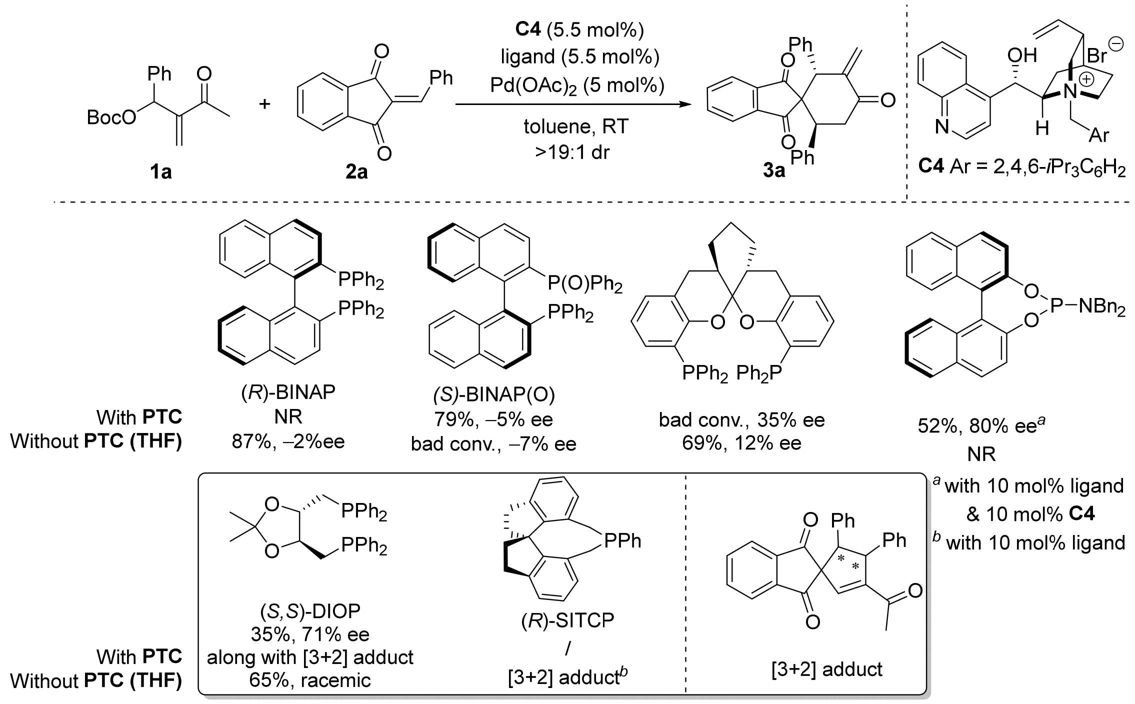

Scheme S3 The effect of IPCs

Unless other noted, the reaction was conducted with MBH carbonate **1a** (0.6mmol), enone **2a** (0.05 mmol), $Pd(OAc)_2$ (0.0025 mmol), **C4** (0.00275 mmol)and ligand (0.00275 mmol) in dry toluene (0.5 mL) under Ar, and the mixture was stirred at room temperature.

To verify the effect of IPCs, the bulky cinchonine derived ammonium salt **C4** was added into the reaction system. It was found that the addition of salt **C4** prohibited the reaction when BINAP was used (*in fact, adding simple TBAB also prohibited the reaction*). In contrast, while the phosphoramidite-Pd complex was inert, adding **C4** significantly promoted the conversion, and moderate yield and good enantioselectivity were obtained. Notably, the traditional phosphine-catalyzed [3+2] annulation product (shown in the Scheme **S3**) was also detected when nucleophilic (*S,S*)-DIOP or (*R*)-SITCP was used.

4.4 Screenings with diverse phosphoramidite ligands

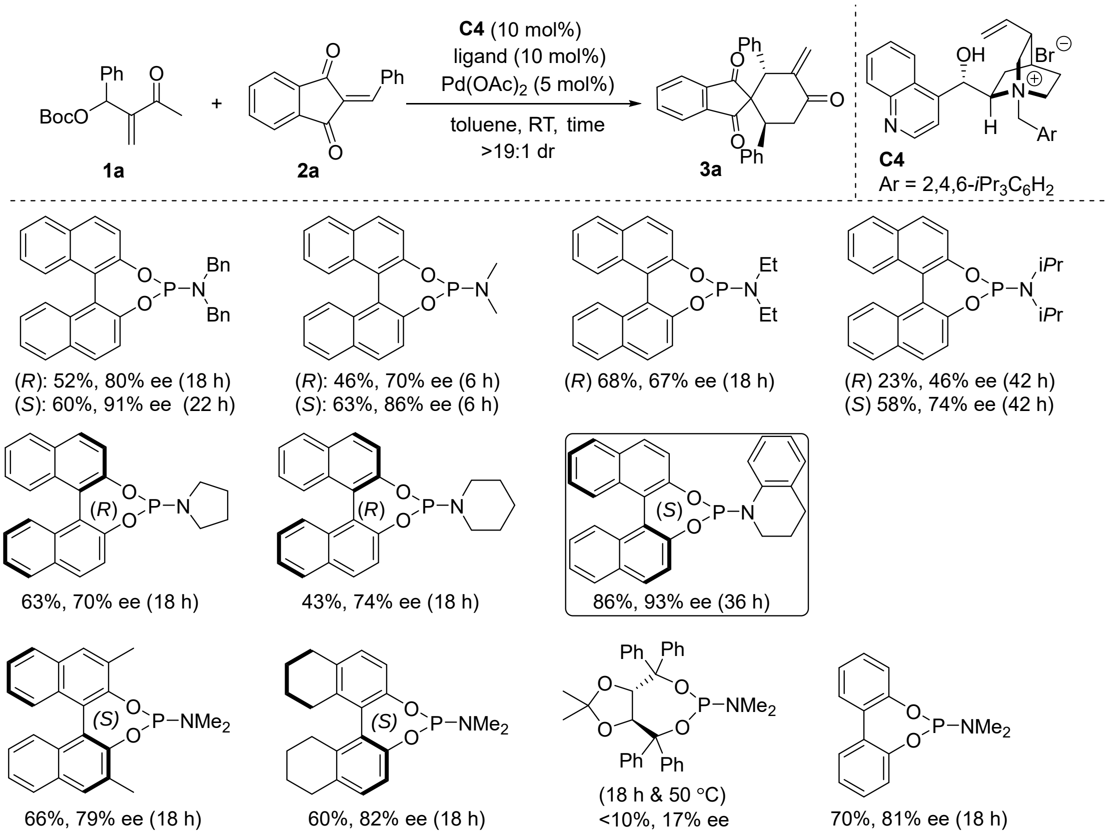

Scheme S4 More screening results of phosphoramidite ligands

Unless other noted, the reaction was conducted with MBH carbonate **1a** (0.6mmol), enone **2a** (0.05 mmol), $Pd(OAc)_2$ (0.0025 mmol), **C4** (0.005 mmol) and phosphoramidite ligand (0.005 mmol) in dry toluene (0.5 mL) under Ar, and the mixture was stirred at room temperature.

To improve the stereocontrol of this [4+2] annulation, we tested a number of phosphoramidite ligands in combination with bulky IPC **C4**. As outlined in Scheme **S4**, apparent match and mismatch effects for the chiral ligands were observed. In general, the enantioselectivity was dominantly controlled by chiral IPC **C4**, since a good ee value could be obtained when an achiral ligand was used.

4.5 Solvent screenings

Table S1 Solvent screenings for the model reaction

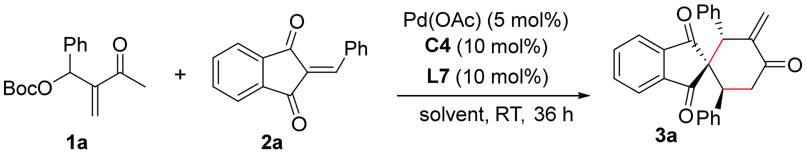

Entry[a]	Solvent	Time (h)	Yield (%)[b]	ee (%)[c]
1	Toluene	36	86	93
2	Xylene	36	60	92
3	Ethyl Acetate	36	78	85
4	CH_2Cl_2	36	ND	35
5	DCE	36	ND	37

[a]Unless otherwise noted, the reactions were conducted with **1a** (0.075 mmol), **2a** (0.05 mmol), $Pd(OAc)_2$ (0.0025 mmol), **C4** (0.005 mmol) and **L7** (0.005 mmol) in dry solvent (0.5 mL) under Ar. [b]Isolated yields. [c]Determined by chiral HPLC analysis on a chiral stationary phase; >19:1 dr. ND = not determined.

4.6 Screening conditions for asymmetric [4+2] annulations involving 3-olefinic oxindoles

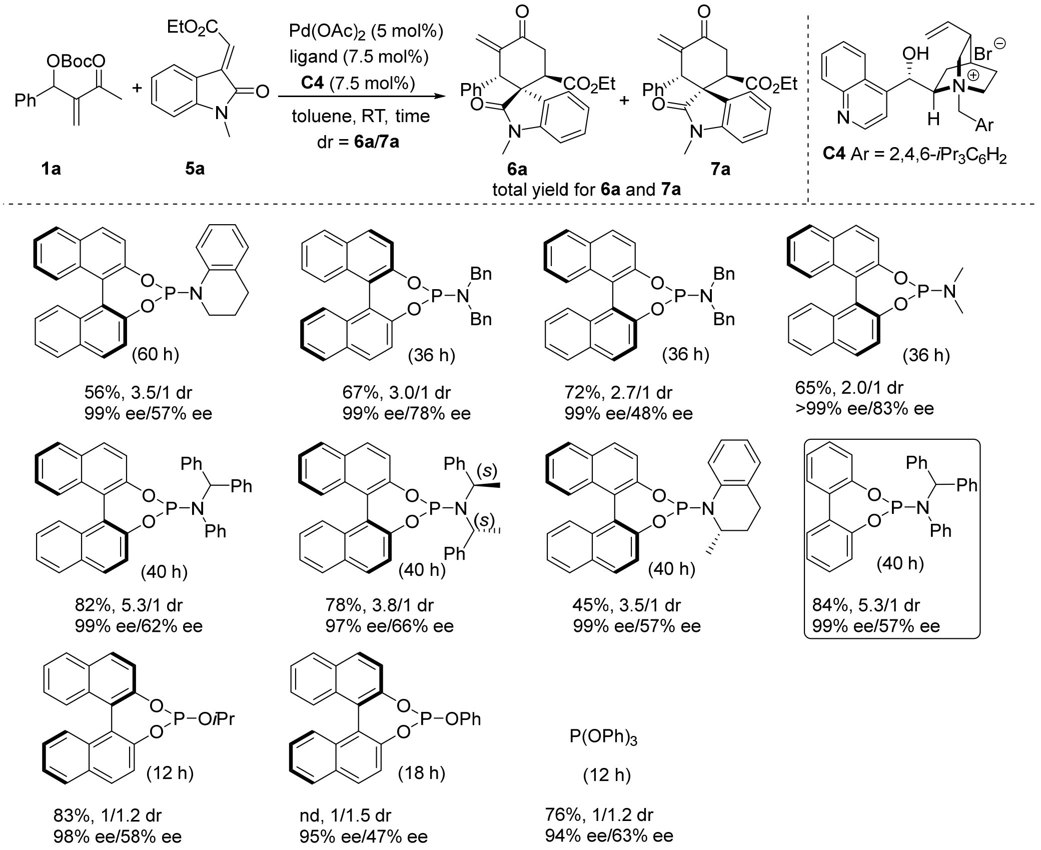

Scheme S5 Screening results of phosphoramidite ligands

The reactions were conducted with MBH carbonate **1a** (0.06 mmol), 3-olefinic oxindole **5a** (0.05 mmol), $Pd(OAc)_2$ (0.0025 mmol), **C4** (0.00375 mmol) and ligand (0.00375 mmol) in dry toluene (0.5 mL) under Ar.

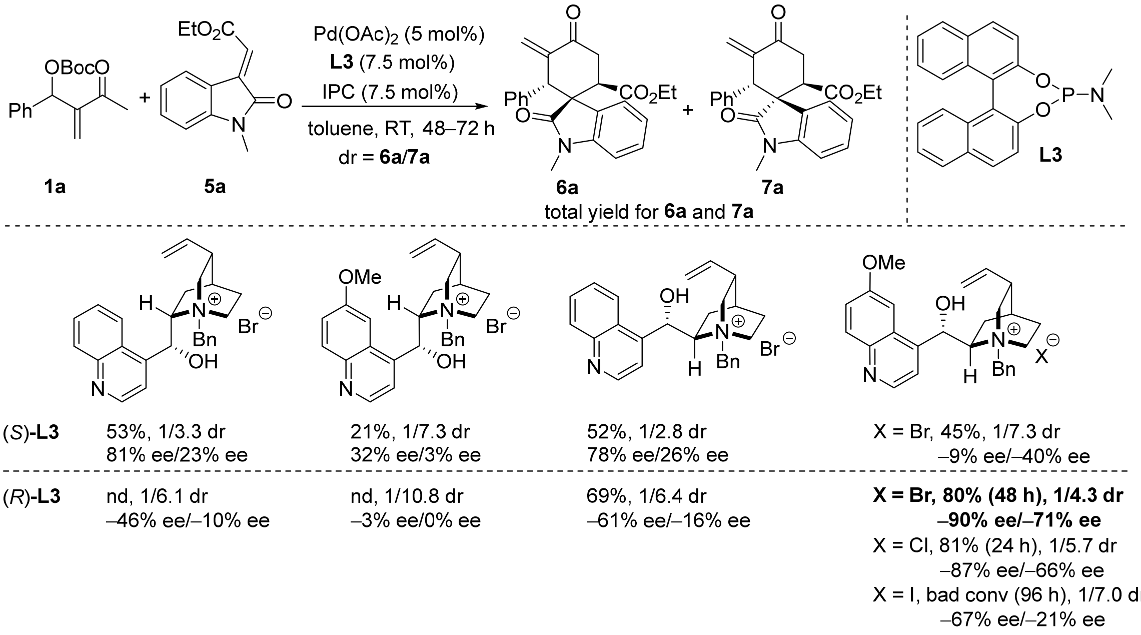

Scheme S6 Screening results by using various ammonium salts

The reactions were conducted with MBH carbonate **1a** (0.06 mmol), 3-olefinic oxindole **5a** (0.05 mmol), $Pd(OAc)_2$ (0.0025 mmol), **IPC** (0.00375 mmol) and **L3** (0.00375 mmol) in dry toluene (0.5 mL) under Ar.

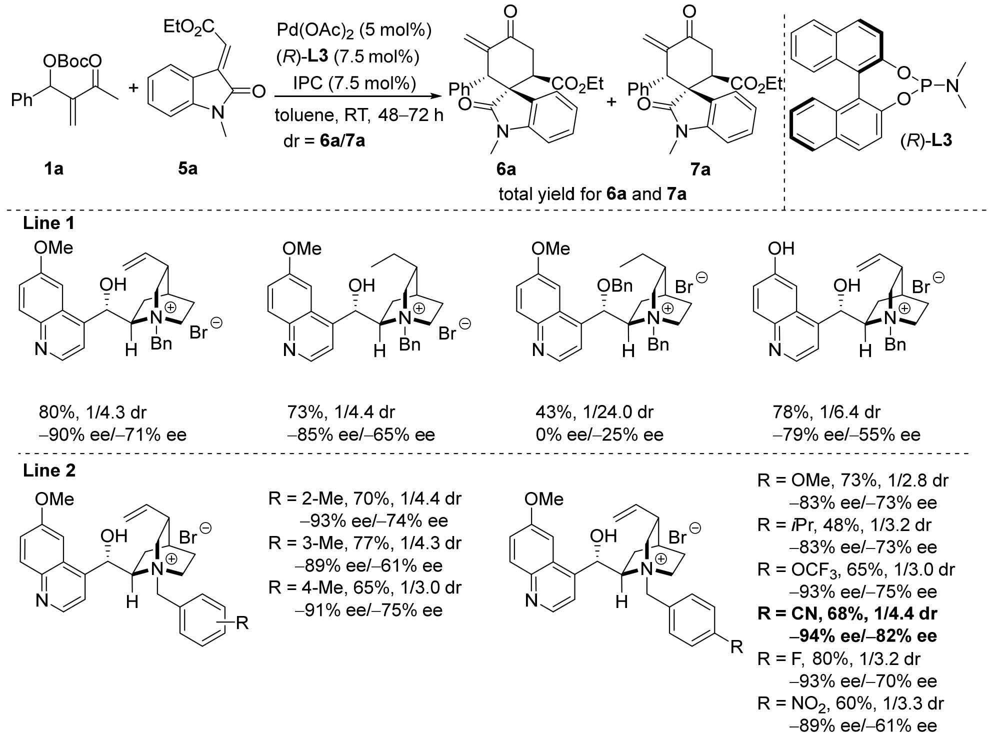

Scheme S7 Diastereoselective results by screening IPCs

The reactions were conducted with MBH carbonate **1a** (0.06 mmol), 3-olefinic oxindole **5a** (0.05 mmol), $Pd(OAc)_2$ (0.0025 mmol), **IPC** (0.00375 mmol) and (*R*)-**L3** (0.00375 mmol) in dry toluene (0.5 mL) under Ar.

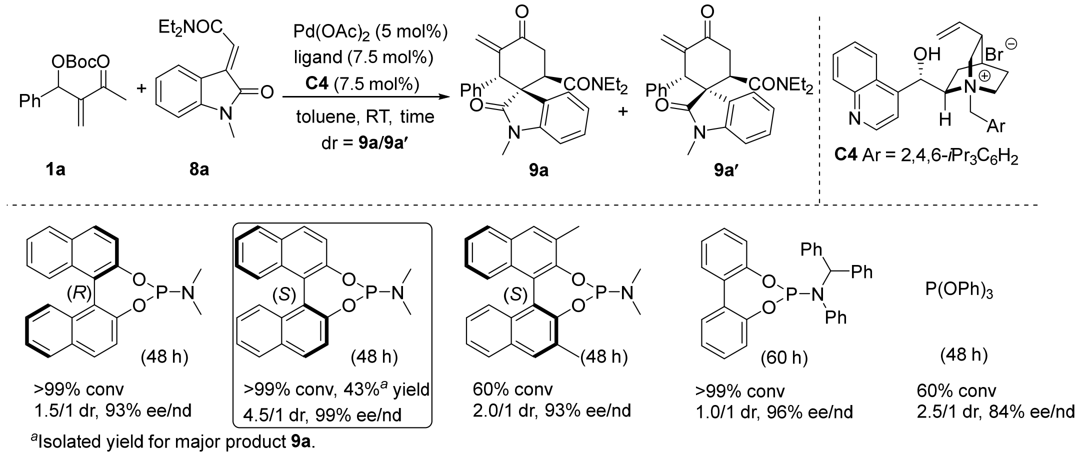

Scheme S8 Screening results of phosphoramidite ligands

The reactions were conducted with MBH carbonate **1a** (0.06 mmol), (*E*)-N,N-diethyl-2-(1-methyl-2-oxoindolin-3-ylidene)acetamide **8a** (0.05 mmol), $Pd(OAc)_2$ (0.0025 mmol), **C4** (0.00375 mmol) and ligand (0.00375 mmol) in dry toluene (0.5 mL) under Ar.

4.7 Screening conditions for asymmetric oxa-[4+2] annulation

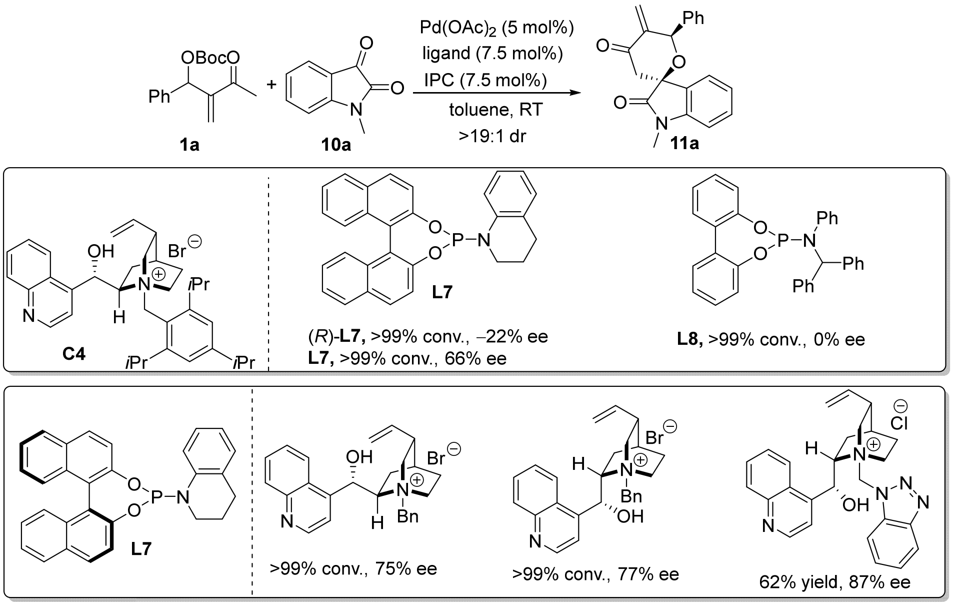

Scheme S9 Screening results of oxa-[4+2] annulation

The reactions were conducted with MBH carbonate **1a** (0.06 mmol), isatin **10a** (0.05 mmol), $Pd(OAc)_2$ (0.0025 mmol), IPC (0.00375 mmol) and ligand (0.00375 mmol) in dry toluene (0.5 mL) under Ar.

5. General procedure for asymmetric [4+2] annulations

5.1 Asymmetric [4+2] annulations involving α-alkylidene-1*H*-indene-1,3(2*H*)-diones

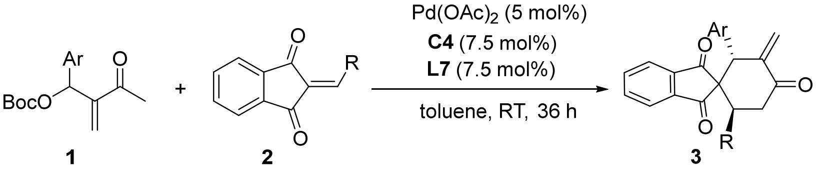

1 **2** **3**

The reaction was conducted with MBH carbonate **1** (0.12 mmol), enone **2** (0.10 mmol), $Pd(OAc)_2$ (0.0050 mmol), **C4** (0.0075 mmol) and **L7** (0.0075 mmol) in dry toluene (1.0 mL) under Ar, and the mixture was stirred at room temperature for 36 h. After completion, the product was obtained by flash chromatography on silica gel (EtOAc/petroleum ether = 1/15–1/10). The racemates were obtained similarly by using the combination of achiral tetrabutylammonium bromide (TBAB) and triphenyl phosphite.

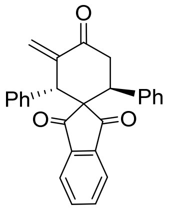

(2*R*,6*S*)-3-Methylene-2,6-diphenylspiro[cyclohexane-1,2'-indene]-1',3',4-trione (3a): *tert*-Butyl (2-methylene-3-oxo-1-phenylbutyl)carbonate **1a** (33.1 mg, 0.120 mmol), 2-benzylidene-1*H*-indene-1,3(2*H*)-dione **2a** (23.4 mg, 0.100 mmol), $Pd(OAc)_2$ (1.1 mg, 0.0050 mmol), **C4** (4.4 mg, 0.0075 mmol) and **L7** (3.4 mg, 0.0075 mmol) were added into a test tube under Ar, followed by the addition of dry toluene (1.0 mL). The mixture was stirred at rt for 36 h, and monitored by TLC. Product **3a** was obtained as a white solid by flash chromatography on silica gel (EtOAc/petroleum ether = 1/10–1/8), 32.7 mg, 83% yield; mp: 158−161 °C; >19:1 dr; 92% ee, determined by HPLC analysis [Daicel Chiral AD-H Column, *i*PrOH/*n*-hexane = 40/60, 1.0 mL/min, λ = 254 nm, t (major) = 11.15 min, t (minor) = 8.16 min]; $[\alpha]^{25}_{D}$ = −25.8 (*c* = 0.31, $CHCl_3$); **^{1}H NMR** (400 MHz, $CDCl_3$): *δ* (ppm) 7.74–7.71 (m, 1H), 7.68–7.58 (m, 3H), 7.19–7.05 (m, 6H), 7.05–7.00 (m, 2H), 7.00–6.95 (m, 2H), 6.44 (s, 1H), 5.33 (s, 1H), 4.44 (s, 1H), 3.84 (dd, *J* = 11.6, 4.8 Hz, 1H), 3.59 (dd, *J* = 17.0, 11.6 Hz, 1H), 3.02 (dd, *J* = 17.0, 4.8 Hz, 1H); **^{13}C NMR** (100 MHz, $CDCl_3$): *δ* (ppm) 201.4, 201.3, 198.6, 143.3, 141.8, 141.4, 138.1, 136.7, 135.65, 135.56, 130.3, 128.7, 128.33, 128.30, 127.7, 127.5, 125.2, 122.99, 122.97, 62.0, 50.5, 43.0, 41.6; **HRMS** (ESI-TOF) m/z: $[M + Na]^+$ Calcd for $C_{27}H_{20}O_3Na^+$ 415.1305; Found 415.1308.

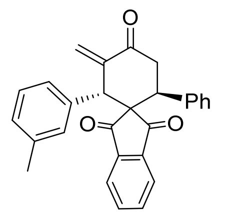

(2*R*,6*S*)-3-Methylene-6-phenyl-2-(*m*-tolyl)spiro[cyclohexane-1,2'-indene]-1',3',4-trione (3b): *tert*-Butyl (2-methylene-3-oxo-1-(*m*-tolyl)butyl)carbonate **1b** (34.8 mg, 0.120 mmol), 2-benzylidene-1*H*-indene-1,3(2*H*)-dione **2a** (23.4 mg, 0.100 mmol), $Pd(OAc)_2$ (1.1 mg, 0.0050 mmol), **C4** (4.4 mg, 0.0075 mmol) and **L7** (3.4 mg, 0.0075 mmol) were added into a test tube under Ar, followed by the addition of dry toluene (1.0 mL). The mixture was stirred at rt for 72 h, and monitored by TLC. Product **3b** was obtained as a white solid by flash chromatography on silica gel (EtOAc/petroleum ether = 1/10–1/8), 30.9 mg, 76% yield; mp: 88–89 °C; >19:1 dr; 93% ee, determined by HPLC analysis [Daicel Chiral AD-H Column, *i*PrOH/*n*-hexane = 20/80, 1.0 mL/min, λ = 254 nm, t (major) = 13.61 min, t (minor) = 10.30 min]; $[\alpha]_D^{25}$ = −15.4 (*c* = 0.33, $CHCl_3$); **^{1}H NMR** (400 MHz, $CDCl_3$): *δ* (ppm) 7.77–7.71 (m, 1H), 7.70–7.56 (m, 3H), 7.13–7.00 (m, 4H), 6.98–6.96 (m, 2H), 6.92 (d, *J* = 7.6 Hz, 1H), 6.82–6.80 (m, 2H), 6.42 (s, 1H), 5.32 (s, 1H), 4.38 (s, 1H), 3.84 (dd, *J* = 11.5, 4.9 Hz, 1H), 3.58 (dd, *J* = 17.0, 11.5 Hz, 1H), 3.02 (dd, *J* = 17.0, 4.9 Hz, 1H), 2.20 (s, 3H); **^{13}C NMR** (100 MHz, $CDCl_3$): *δ* (ppm) 201.4, 201.3, 198.7, 143.4, 141.9, 141.4, 138.2, 137.9, 136.6, 135.6, 135.5, 131.0, 128.7, 128.4, 128.3, 128.2, 127.5, 127.3, 125.2, 123.0, 122.9, 62.0, 50.5, 42.9, 41.7, 21.3; **HRMS** (ESI-TOF) m/z: $[M + Na]^+$ Calcd for $C_{28}H_{22}O_3Na^+$ 429.1461; Found 429.1465.

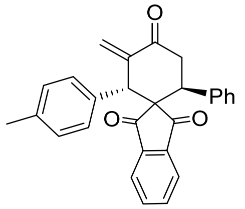

(2*R*,6*S*)-3-Methylene-6-phenyl-2-(*p*-tolyl)spiro[cyclohexane-1,2'-indene]-1',3',4-trione (3c): *tert*-Butyl (2-methylene-3-oxo-1-(*p*-tolyl)butyl)carbonate **1c** (34.8 mg, 0.120 mmol), 2-benzylidene-1*H*-indene-1,3(2*H*)-dione **2a** (23.4 mg, 0.100 mmol), $Pd(OAc)_2$ (1.1 mg, 0.0050 mmol), **C4** (4.4 mg, 0.0075 mmol) and **L7** (3.4 mg, 0.0075 mmol) were added into a test tube under Ar, followed by the addition of dry toluene (1.0 mL). The mixture was stirred at rt for 72 h, and monitored by TLC. Product **3c** was obtained as a white solid by flash chromatography on silica gel (EtOAc/petroleum ether = 1/10–1/8), 31.7 mg, 78% yield; mp: 197–199 °C; >19:1 dr; 93% ee, determined by HPLC analysis [Daicel Chiral AD-H Column, *i*PrOH/*n*-hexane = 20/80, 1.0 mL/min, λ = 254 nm, t (major) = 13.89 min, t (minor) = 12.53 min]; $[\alpha]_D^{25}$ = −50.8 (*c* = 0.37, $CHCl_3$); **^{1}H NMR** (400 MHz, $CDCl_3$): *δ* (ppm) 7.79–7.71 (m, 1H), 7.71–7.58 (m, 3H), 7.14–7.01 (m, 3H), 6.98–6.94 (m, 4H), 6.93–6.82 (m, 2H), 6.41 (t, *J* = 1.4 Hz, 1H), 5.31 (dd, *J* = 2.0, 1.4 Hz, 1H), 4.38 (t, *J* = 2.0 Hz, 1H), 3.84 (dd, *J* = 11.7, 4.9 Hz, 1H), 3.58 (dd, *J* = 17.1, 11.7 Hz, 1H), 3.01 (dd, *J* = 17.1, 4.9 Hz, 1H), 2.21 (s, 3H); **^{13}C NMR** (100 MHz,

$CDCl_3$): δ (ppm) 201.6, 201.2, 198.8, 143.5, 141.8, 141.3, 138.2, 137.3, 135.7, 135.6, 133.8, 130.1, 129.1, 128.7, 128.3, 127.5, 125.2, 123.04, 123.01, 62.0, 50.2, 42.9, 41.7, 21.0; **HRMS** (ESI-TOF) m/z: $[M + Na]^+$ Calcd for $C_{28}H_{22}O_3Na^+$ 429.1461; Found 429.1465.

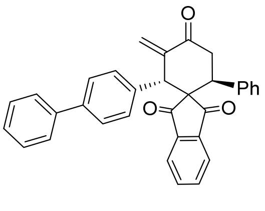

(2*R*,6*S*)-2-([1,1'-Biphenyl]-4-yl)-3-methylene-6-phenylspiro[cyclohexane-1,2'-indene]-1',3',4-trione (3d): 1-([1,1'-Biphenyl]-4-yl)-2-methylene-3-oxobutyl *tert*-butyl carbonate **1d** (42.2 mg, 0.120 mmol), 2-benzylidene-1*H*-indene-1,3(2*H*)-dione **2a** (23.4 mg, 0.100 mmol), $Pd(OAc)_2$ (1.1 mg, 0.0050 mmol), **C4** (4.4 mg, 0.0075 mmol) and **L7** (3.4 mg, 0.0075 mmol) were added into a test tube under Ar, followed by the addition of dry toluene (1.0 mL). The mixture was stirred at rt for 36 h, and monitored by TLC. Product **3d** was obtained as a white solid by flash chromatography on silica gel (EtOAc/petroleum ether = 1/10–1/8), 37.3 mg, 80% yield; mp: 194–195 °C; >19:1 dr; 92% ee, determined by HPLC analysis [Daicel Chiral IA Column, *i*PrOH/*n*-hexane = 40/60, 1.0 mL/min, λ = 254 nm, t (major) = 17.50 min, t (minor) = 14.64 min]; $[\alpha]_D^{25} = -112.5$ (*c* = 0.24, $CHCl_3$); **^{1}H NMR** (400 MHz, $CDCl_3$): δ (ppm) 7.78–7.72 (m, 1H), 7.70–7.66 (m, 1H), 7.65–7.60 (m, 2H), 7.51–7.45 (m, 2H), 7.43–7.35 (m, 4H), 7.33–7.27 (m, 1H), 7.16–7.07 (m, 5H), 7.01–6.94 (m, 2H), 6.48 (s, 1H), 5.38 (s, 1H), 4.50 (s, 1H), 3.86 (dd, *J* = 11.4, 4.8 Hz, 1H), 3.59 (dd, *J* = 17.0, 11.4 Hz, 1H), 3.05 (dd, *J* = 17.0, 4.8 Hz, 1H); **^{13}C NMR** (100 MHz, $CDCl_3$): δ (ppm) 201.41, 201.40, 198.6, 143.3, 141.8, 141.4, 140.3, 140.2, 138.1, 135.74, 135.71, 135.67, 130.7, 128.74, 128.73, 128.3, 127.5, 127.4, 126.92, 126.91, 125.4, 123.1, 123.0, 62.1, 50.0, 43.2, 41.6; **HRMS** (ESI-TOF) m/z: $[M + Na]^+$ Calcd for $C_{33}H_{24}O_3Na^+$ 491.1618; Found 491.1620.

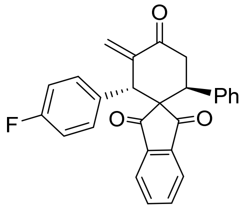

(2*R*,6*S*)-2-(4-Fluorophenyl)-3-methylene-6-phenylspiro[cyclohexane-1,2'-indene]-1',3',4-trione (3e): *tert*-Butyl 1-(4-fluorophenyl)-2-methylene-3-oxobutyl carbonate **1e** (35.3 mg, 0.120 mmol), 2-benzylidene-1*H*-indene-1,3(2*H*)-dione **2a** (23.4 mg, 0.100 mmol), $Pd(OAc)_2$ (1.1 mg, 0.0050 mmol), **C4** (4.4 mg, 0.0075 mmol) and **L7** (3.4 mg, 0.0075 mmol) were added into a test tube under Ar, followed by the addition of dry toluene (1.0 mL). The mixture was stirred at rt for 36 h, and monitored by TLC. Product **3e** was obtained as a pale yellow solid by flash chromatography on silica gel (EtOAc/petroleum ether = 1/10–1/8), 38.3 mg, 93% yield; mp: 57–60 °C; >19:1 dr; 94% ee,

determined by HPLC analysis [Daicel Chiral AD-H Column, *i*PrOH/*n*-hexane = 20/80, 1.0 mL/min, λ = 254 nm, t (major) = 17.77 min, t (minor) = 13.38 min]; $[\alpha]_D^{25}$ = –29.4 (*c* = 0.77, $CHCl_3$); **^{1}H NMR** (400 MHz, $CDCl_3$): *δ* (ppm) 7.80–7.72 (m, 1H), 7.70–7.59 (m, 3H), 7.14–7.04 (m, 3H), 7.03–6.97 (m, 2H), 6.97–6.91 (m, 2H), 6.87–6.77 (m, 2H), 6.47 (dd, *J* = 2.2, 1.1 Hz, 1H), 5.30 (dd, *J* = 2.2, 1.1 Hz, 1H), 4.47 (t, *J* = 2.2 Hz, 1H), 3.78 (dd, *J* = 11.0, 4.8 Hz, 1H), 3.54 (dd, *J* = 17.0, 11.0 Hz, 1H), 3.02 (dd, *J* = 17.0, 4.8 Hz, 1H); **^{13}C NMR** (100 MHz, $CDCl_3$): *δ* (ppm) 201.6, 201.2, 198.4, 162.0 (d, *J* = 247.3 Hz), 143.2, 141.7, 141.4, 137.9, 135.83, 135.80, 132.40, 132.37, 132.0 (d, *J* = 8.1 Hz), 128.7, 128.3, 127.6, 125.3, 123.0 (d, *J* = 3.0 Hz), 115.3 (d, *J* = 21.4 Hz), 62.1, 49.2, 43.3, 41.5; **^{19}F NMR** (376 MHz, $CDCl_3$): *δ* (ppm) −114.2; **HRMS** (ESI-TOF) m/z: $[M + Na]^+$ Calcd for $C_{27}H_{19}FNaO_3^+$ 433.1210; Found 433.1207.

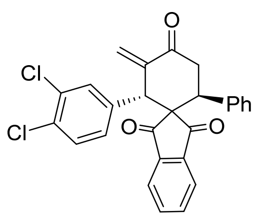

(2*R*,6*S*)-2-(3,4-Dichlorophenyl)-3-methylene-6-phenylspiro[cyclohexane-1,2'-indene]-1',3',4-trione (3f): *tert*-Butyl (1-(3,4-dichlorophenyl)-2-methylene-3-oxobutyl)carbonate **1f** (41.4 mg, 0.120 mmol), 2-benzylidene-1*H*-indene-1,3(2*H*)-dione **2a** (23.4 mg, 0.100 mmol), $Pd(OAc)_2$ (0.60 mg, 0.0025 mmol), **C4** (2.2 mg, 0.0038 mmol) and **L7** (1.7 mg, 0.0038 mmol) were added into a test tube under Ar, followed by the addition of dry toluene (1.0 mL). The mixture was stirred at rt for 36 h, and monitored by TLC. Product **3f** was obtained as a pale yellow solid by flash chromatography on silica gel (EtOAc/petroleum ether = 1/10–1/8), 32.6 mg, 70% yield; mp: 166–167 °C; >19:1 dr; 93% ee, determined by HPLC analysis [Daicel Chiral IA Column, *i*PrOH/*n*-hexane = 20/80, 1.0 mL/min, λ = 254 nm, t (major) = 14.69 min, t (minor) = 13.28 min]; $[\alpha]_D^{25}$ = –63.0 (*c* = 0.24, $CHCl_3$); **^{1}H NMR** (400 MHz, $CDCl_3$): *δ* (ppm) 7.83–7.76 (m, 1H), 7.75–7.65 (m, 3H), 7.23 (d, *J* = 8.3 Hz, 1H), 7.16–7.07 (m, 4H), 6.96–6.86 (m, 3H), 6.49 (dd, *J* = 2.2, 1.0 Hz, 1H), 5.26 (dd, *J* = 2.2, 1.0 Hz, 1H), 4.41 (t, *J* = 2.2 Hz, 1H), 3.74 (dd, *J* = 10.2, 5.2 Hz, 1H), 3.46 (dd, *J* = 17.4, 10.2 Hz, 1H), 3.06 (dd, *J* = 17.4, 5.2 Hz, 1H); **^{13}C NMR** (100 MHz, $CDCl_3$): *δ* (ppm) 201.1, 200.5, 197.7, 142.7, 141.4, 141.3, 137.7, 137.2, 136.08, 136.06, 132.5, 132.3, 132.0, 130.3, 129.6, 128.7, 128.4, 127.8, 125.8, 123.3, 123.2, 61.6, 48.5, 43.5, 41.4; **HRMS** (ESI-TOF) m/z: $[M + H]^+$ Calcd for $C_{27}H_{19}Cl_2O_3^+$ 461.0706 (^{35}Cl*2) and 463.0676 (^{35}Cl + ^{37}Cl); Found 461.0700 (^{35}Cl*2) and 463.0675 (^{35}Cl + ^{37}Cl).

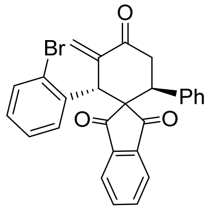

(2*R*,6*S*)-2-(2-Bromophenyl)-3-methylene-6-phenylspiro[cyclohexane-1,2'-indene]-1',3',4-trione (3g): 1-(2-Bromophenyl)-2-methylene-3-oxobutyl *tert*-butyl carbonate **1g** (42.6 mg, 0.120 mmol), 2-benzylidene-1*H*-indene-1,3(2*H*)-dione **2a** (23.4 mg, 0.100 mmol), $Pd(OAc)_2$ (1.1 mg, 0.0050 mmol), **C4** (4.4 mg, 0.0075 mmol) and **L7** (3.4 mg, 0.0075 mmol) were added into a test tube under Ar, followed by the addition of dry toluene (1.0 mL). The mixture was stirred at rt for 72 h, and monitored by TLC. Product **3g** was obtained as a white solid by flash chromatography on silica gel (EtOAc/petroleum ether = 1/10–1/8), 44.0 mg, 94% yield; mp: 198–199 °C; >19:1 dr; 92% ee, determined by HPLC analysis [Daicel Chiral IC Column, *i*PrOH/*n*-hexane = 40/60, 1.0 mL/min, λ = 254 nm, t (major) = 20.76 min, t (minor) = 17.54 min]; $[\alpha]_D^{25}$ = +30.7 (*c* = 0.22, $CHCl_3$); **^{1}H NMR** (400 MHz, $CDCl_3$): *δ* (ppm) 7.89 (dt, *J* = 7.5, 1.2 Hz, 1H), 7.74–7.70 (m, 1H), 7.69–7.64 (m, 2H), 7.51 (dd, *J* = 7.8, 1.4 Hz, 1H), 7.34 (td, *J* = 7.8, 1.4 Hz, 1H), 7.18–7.12 (m, 2H), 7.08–6.91 (m, 5H), 6.34 (t, *J* = 1.2 Hz, 1H), 5.27 (t, *J* = 1.2 Hz, 1H), 4.89 (t, *J* = 1.2 Hz, 1H), 3.97 (dd, *J* = 13.2, 5.4 Hz, 1H), 3.66 (dd, *J* = 17.9, 13.2 Hz, 1H), 3.00 (dd, *J* = 17.9, 5.4 Hz, 1H); **^{13}C NMR** (100 MHz, $CDCl_3$): *δ* (ppm) 202.0, 198.7, 198.6, 143.6, 142.5, 140.7, 139.0, 137.6, 135.9, 135.5, 132.9, 130.7, 129.1, 128.8, 128.3, 127.6, 127.5, 126.4, 126.2, 123.2, 59.5, 50.0, 42.3, 41.9; **HRMS** (ESI-TOF) m/z: $[M + Na]^+$ Calcd for $C_{27}H_{19}BrO_3Na^+$ 493.0410 (^{79}Br) and 495.0389 (^{81}Br); Found 493.0414 (^{79}Br) and 495.0399 (^{81}Br).

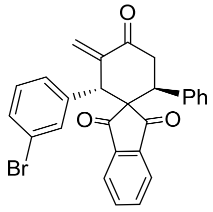

(2*R*,6*S*)-2-(3-Bromophenyl)-3-methylene-6-phenylspiro[cyclohexane-1,2'-indene]-1',3',4-trione (3h): 1-(3-Bromophenyl)-2-methylene-3-oxobutyl *tert*-butyl carbonate **1h** (42.6 mg, 0.120 mmol), 2-benzylidene-1*H*-indene-1,3(2*H*)-dione **2a** (23.4 mg, 0.100 mmol), $Pd(OAc)_2$ (1.1 mg, 0.0050 mmol), **C4** (4.4 mg, 0.0075 mmol) and **L7** (3.4 mg, 0.0075 mmol) were added into a test tube under Ar, followed by the addition of dry toluene (1.0 mL). The mixture was stirred at rt for 36 h, and monitored by TLC. Product **3h** was obtained as a white solid by flash chromatography on silica gel (EtOAc/petroleum ether = 1/10–1/8), 42.3 mg, 90% yield; mp: 171–172 °C; >19:1 dr; 92% ee, determined by HPLC analysis [Daicel Chiral IC Column, *i*PrOH/*n*-hexane = 10/90, 1.0 mL/min, λ = 254 nm, t (major) = 20.25 min, t (minor) = 17.75 min]; $[\alpha]_D^{25}$ = −19.5 (*c* = 0.43, $CHCl_3$); **^{1}H NMR** (400 MHz, $CDCl_3$): *δ* (ppm) 8.05–7.63 (m, 4H), 7.36 (t, *J* = 4.0 Hz, 1H), 7.24–6.95 (m, 8H), 6.57 (s, 1H), 5.39 (s, 1H), 4.49 (s, 1H), 3.88 (dd, *J* = 10.8, 5.0 Hz, 1H), 3.61 (dd, *J* = 17.2, 10.8 Hz, 1H), 3.15 (dd, *J* = 17.2, 5.0 Hz,

1H); **^{13}C NMR** (150 MHz, $CDCl_3$): δ (ppm) 201.4, 201.2, 198.4, 143.1, 142.0, 141.6, 139.5, 138.2, 136.2, 136.2, 133.6, 131.2, 130.2, 129.2, 129.0, 128.7, 128.0, 126.0, 123.5, 123.4, 122.7, 62.1, 49.9, 43.5, 41.8; **HRMS** (ESI-TOF) m/z: $[M + Na]^+$ Calcd for $C_{27}H_{19}O_3BrNa^+$ 493.0410 (^{79}Br) and 495.0389 (^{81}Br); Found 493.0408 (^{79}Br) and 495.0395 (^{81}Br).

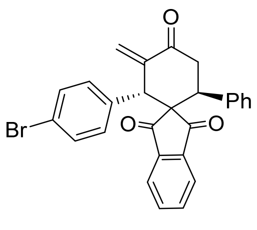

(2*R*,6*S*)-2-(4-Bromophenyl)-3-methylene-6-phenylspiro[cyclohexane-1,2'-indene]-1',3',4-trione (3i): 1-(4-Bromophenyl)-2-methylene-3-oxobutyl *tert*-butyl carbonate **1i** (42.6 mg, 0.120 mmol), 2-benzylidene-1*H*-indene-1,3(2*H*)-dione **2a** (23.4 mg, 0.100 mmol), $Pd(OAc)_2$ (0.60 mg, 0.0025 mmol), **C4** (2.2 mg, 0.0038 mmol) and **L7** (1.7 mg, 0.0038 mmol) were added into a test tube under Ar, followed by the addition of dry toluene (1.0 mL). The mixture was stirred at rt for 36 h, and monitored by TLC. Product **3i** was obtained as a white solid by flash chromatography on silica gel (EtOAc/petroleum ether = 1/10–1/8), 41.8 mg, 89% yield; mp: 179–181 °C; >19:1 dr; 89% ee, determined by HPLC analysis [Daicel Chiral IA Column, *i*PrOH/*n*-hexane = 20/80, 1.0 mL/min, λ = 254 nm, t (major) = 16.13 min, t (minor) = 12.07 min]; $[\alpha]_D^{25} = -66.3$ (*c* = 0.18, $CHCl_3$); **^{1}H NMR** (400 MHz, $CDCl_3$): δ (ppm) 7.80–7.73 (m, 1H), 7.73–7.64 (m, 3H), 7.32–7.25 (m, 2H), 7.12–7.08 (m, 3H), 6.98–6.88 (m, 4H), 6.46 (dd, *J* = 2.2, 1.0 Hz, 1H), 5.28 (dd, *J* = 2.2, 1.0 Hz, 1H), 4.43 (t, *J* = 2.2 Hz, 1H), 3.76 (dd, *J* = 10.7, 5.0 Hz, 1H), 3.50 (dd, *J* = 17.2, 10.7 Hz, 1H), 3.04 (dd, *J* = 17.2, 5.0 Hz, 1H); **^{13}C NMR** (100 MHz, $CDCl_3$): δ (ppm) 201.3, 200.9, 198.1, 143.0, 141.6, 141.4, 137.9, 135.9, 132.0, 131.5, 128.7, 128.3, 127.6, 125.4, 123.12, 123.09, 121.8, 61.9, 49.2, 43.4, 41.5; **HRMS** (ESI-TOF) m/z: $[M + H]^+$ Calcd for $C_{27}H_{20}BrO_3^+$ 471.0590 (^{79}Br) and 473.0570 (^{81}Br) Found 471.0584 (^{79}Br) and 473.0568 (^{81}Br).

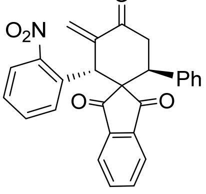

(2*R*,6*S*)-3-Methylene-2-(2-nitrophenyl)-6-phenylspiro[cyclohexane-1,2'-indene]-1',3',4-trione (3j): *tert*-Butyl (2-methylene-1-(2-nitrophenyl)-3-oxobutyl)carbonate **1j** (38.5 mg, 0.120 mmol), 2-benzylidene-1*H*-indene-1,3(2*H*)-dione **2a** (23.4 mg, 0.100 mmol), $Pd(OAc)_2$ (1.1 mg, 0.0050 mmol), **C4** (4.4 mg, 0.0075 mmol) and **L7** (3.4 mg, 0.0075 mmol) were added into a test tube under Ar, followed by the addition of dry toluene (1.0 mL). The mixture was stirred at rt for 36 h, and monitored by TLC. Product **3j** was obtained as a white solid by flash chromatography on silica gel (EtOAc/petroleum

ether = 1/10–1/8), 35.4 mg, 80% yield; mp: 178−181 °C; >19:1 dr; 88% ee, determined by HPLC analysis [Daicel Chiral IA Column, *i*PrOH/*n*-hexane = 20/80, 1.0 mL/min, λ = 254 nm, t (major) = 10.46 min, t (minor) = 19.78 min]; $[\alpha]_D^{25}$ = −36.5 (*c* = 0.34, $CHCl_3$); **^{1}H NMR** (400 MHz, $CDCl_3$): *δ* (ppm) 8.01 (dd, *J* = 8.3, 1.4 Hz, 1H), 7.90 (d, *J* = 7.8 Hz, 1H), 7.76–7.60 (m, 3H), 7.57–7.46 (m, 2H), 7.30 (dd, *J* = 7.8, 1.4 Hz, 1H), 7.04–6.94 (m, 3H), 6.94–6.85 (m, 2H), 6.37 (s, 1H), 5.28 (s, 1H), 5.11 (s, 1H), 3.95 (dd, *J* = 13.4, 5.3 Hz, 1H), 3.63 (dd, *J* = 17.9, 13.4 Hz, 1H), 2.99 (dd, *J* = 17.9, 5.3 Hz, 1H); **^{13}C NMR** (100 MHz, $CDCl_3$): *δ* (ppm) 202.1, 199.0, 198.5, 148.4, 143.7, 142.6, 140.7, 137.0, 136.2, 136.0, 135.5, 133.4, 131.6, 128.7, 128.5, 128.3, 127.7, 127.5, 125.5, 123.0, 122.9, 59.0, 45.8, 42.6, 42.1; **HRMS** (ESI-TOF) m/z: $[M + Na]^+$ Calcd for $C_{27}H_{19}NO_5Na^+$ 460.1155; Found 460.1154.

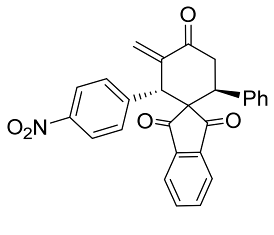

(2R,6S)-3-Methylene-2-(4-nitrophenyl)-6-phenylspiro[cyclohexane-1,2'-indene]-1',3',4-trione (3k): *tert*-Butyl (2-methylene-1-(4-nitrophenyl)-3-oxobutyl)carbonate **1k** (38.5 mg, 0.120 mmol), 2-benzylidene-1*H*-indene-1,3(2*H*)-dione **2a** (23.4 mg, 0.100 mmol), $Pd(OAc)_2$ (1.1 mg, 0.0050 mmol), **C4** (4.4 mg, 0.0075 mmol) and **L7** (3.4 mg, 0.0075 mmol) were added into a test tube under Ar, followed by the addition of dry toluene (1.0 mL). The mixture was stirred at rt for 36 h, and monitored by TLC. Product **3k** was obtained as a white solid by flash chromatography on silica gel (EtOAc/petroleum ether = 1/10–1/8), 42.7 mg, 98% yield; mp: 153−156 °C;>19:1 dr; 95% ee, determined by HPLC analysis [Daicel Chiral IC Column, *i*PrOH/*n*-hexane = 40/60, 1.0 mL/min, λ = 254 nm, t (major) = 23.19 min, t (minor) = 19.11 min]; $[\alpha]_D^{25}$ = −69.8 (*c* = 0.11, $CHCl_3$); **^{1}H NMR** (400 MHz, $CDCl_3$): *δ* (ppm) 8.08–7.98 (m, 2H), 7.82–7.61 (m, 4H), 7.24 (d, *J* = 8.8 Hz, 2H), 7.16–7.11 (m, 3H), 6.95–6.92 (m, 2H), 6.52 (s, 1H), 5.25 (s, 1H), 4.56 (s, 1H), 3.76 (dd, *J* = 10.1, 5.3 Hz, 1H), 3.48 (dd, *J* = 17.5, 10.1 Hz, 1H), 3.11 (dd, *J* = 17.5, 5.3 Hz, 1H); **^{13}C NMR** (100 MHz, $CDCl_3$): *δ* (ppm) 200.9, 200.3, 197.5, 147.2, 144.7, 142.4, 141.3, 141.2, 137.5, 136.22, 136.17, 131.3, 128.7, 128.5, 127.9, 126.0, 123.5, 123.32, 123.28, 61.6, 49.1, 43.5, 41.4; **HRMS** (ESI-TOF) m/z: $[M + Na]^+$ Calcd for $C_{27}H_{19}NO_5Na^+$ 460.1155; Found 460.1152.

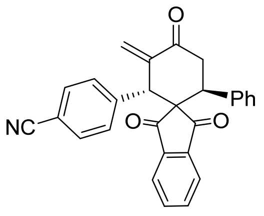

4-((2*S*,6*R*)-5-Methylene-1',3',4-trioxo-2-phenyl-1',3'-dihydrospiro[cyclohexane-1,2'-inden]-6-yl)benzonitrile (3l): *tert*-Butyl (2-methylene-1-(4-cycnophenyl)-3-oxobutyl)carbonate **1l** (36.1 mg, 0.120 mmol), 2-benzylidene-1*H*-indene-1,3(2*H*)-dione **2a** (23.4 mg, 0.100 mmol), $Pd(OAc)_2$ (1.1 mg, 0.0050 mmol), **C4** (4.4 mg, 0.0075 mmol) and **L7** (3.4 mg, 0.0075 mmol) were added into a test tube under Ar, followed by the addition of dry toluene (1.0 mL). The mixture was stirred at rt for 36 h, and monitored by TLC. Product **3l** was obtained as a white solid by flash chromatography on silica gel (EtOAc/petroleum ether = 1/10–1/8), 34.8 mg, 83% yield; mp: 173–174 °C; >19:1 dr; 95% ee, determined by HPLC analysis [Daicel Chiral AD-H Column, *i*PrOH/*n*-hexane = 20/80, 1.0 mL/min, λ = 254 nm, t (major) = 42.23 min, t (minor) = 26.22 min]; $[\alpha]_D^{25}$ = –87.0 (*c* = 0.20, $CHCl_3$); **^{1}H NMR** (400 MHz, $CDCl_3$): *δ* (ppm) 7.77–7.74 (m, 1H), 7.74–7.66 (m, 3H), 7.51–7.42 (m, 2H), 7.17–7.15 (m, 2H), 7.13–7.10 (m, 3H), 6.97–6.88 (m, 2H), 6.49 (dd, *J* = 2.2, 0.9 Hz, 1H), 5.24 (dd, *J* = 2.2, 0.9 Hz, 1H), 4.49 (t, *J* = 2.2 Hz, 1H), 3.76 (dd, *J* = 10.4, 5.2 Hz, 1H), 3.49 (dd, *J* = 17.4, 10.4 Hz, 1H), 3.08 (dd, *J* = 17.4, 5.2 Hz, 1H); **^{13}C NMR** (100 MHz, $CDCl_3$): *δ* (ppm) 200.9, 200.5, 197.6, 142.6, 142.4, 141.3, 141.2, 137.6, 136.2, 136.1, 132.1, 131.1, 128.7, 128.4, 127.8, 125.9, 123.3, 123.2, 118.3, 111.7, 61.6, 49.6, 43.4, 41.4; **HRMS** (ESI-TOF) m/z: $[M + Na]^+$ Calcd for $C_{28}H_{19}NO_3Na^+$ 440.1257; Found 440.1257.

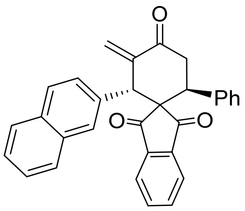

(2*R*,6*S*)-3-Methylene-2-(naphthalen-2-yl)-6-phenylspiro[cyclohexane-1,2'-indene]-1',3',4-trione (3m): *tert*-Butyl (2-methylene-1-(naphthalen-2-yl)-3-oxobutyl)carbonate **1m** (39.1 mg, 0.120 mmol), 2-benzylidene-1*H*-indene-1,3(2*H*)-dione **2a** (23.4 mg, 0.100 mmol), $Pd(OAc)_2$ (1.1 mg, 0.0050 mmol), **C4** (4.4 mg, 0.0075 mmol) and **L7** (3.4 mg, 0.0075 mmol) were added into a test tube under Ar, followed by the addition of dry toluene (1.0 mL). The mixture was stirred at rt for 36 h, and monitored by TLC. Product **3m** was obtained as a white solid by flash chromatography on silica gel (EtOAc/petroleum ether = 1/10–1/8), 37.4 mg, 84% yield; mp: 186–187 °C; >19:1 dr; 92% ee, determined by HPLC analysis [Daicel Chiral IE Column, *i*PrOH/*n*-hexane = 40/60, 1.0 mL/min, λ = 254 nm, t (major) = 16.82 min, t (minor) = 12.47 min]; $[\alpha]_D^{25}$ = –70.0 (*c* = 0.20, $CHCl_3$); **^{1}H NMR** (400 MHz, $CDCl_3$): *δ* (ppm) 7.75–7.72 (m, 1H), 7.72–7.67 (m, 2H), 7.65–7.53 (m, 4H), 7.49 (d, *J* = 1.8 Hz, 1H), 7.44–7.35 (m, 2H), 7.19 (dd, *J* = 8.6, 1.8 Hz, 1H), 7.17–7.04 (m, 3H), 7.01–6.93 (m, 2H), 6.49 (dd, *J* = 2.2, 1.2

Hz, 1H), 5.33 (dd, *J* = 2.2, 1.2 Hz, 1H), 4.65 (t, *J* = 2.2 Hz, 1H), 3.86 (dd, *J* = 10.7, 5.0 Hz, 1H), 3.57 (dd, *J* = 17.2, 10.7 Hz, 1H), 3.11 (dd, *J* = 17.2, 5.0 Hz, 1H); **^{13}C NMR** (100 MHz, $CDCl_3$): δ (ppm) 201.6, 201.2, 198.5, 143.5, 141.7, 141.3, 138.1, 135.71, 135.67, 134.3, 133.0, 132.6, 129.8, 128.8, 128.3, 128.0, 127.8, 127.6, 127.5, 126.2, 126.1, 125.6, 123.1, 123.0, 62.1, 50.0, 43.5, 41.6; **HRMS** (ESI-TOF) m/z: $[M + Na]^+$ Calcd for $C_{31}H_{22}O_3Na^+$ 465.1461; Found 465.1465.

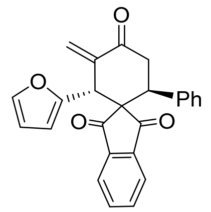

(2*R*,6*S*)-3-Methylene-2-(4-nitrophenyl)-6-phenylspiro[cyclohexane-1,2'-indene]-1',3',4-trione (3n): *tert*-Butyl (1-(furan-2-yl)-2-methylene-3-oxobutyl) carbonate **1n** (31.9 mg, 0.120 mmol), 2-benzylidene-1*H*-indene-1,3(2*H*)-dione **2a** (23.4 mg, 0.100 mmol), $Pd(OAc)_2$ (0.60 mg, 0.0025 mmol), **C4** (2.2 mg, 0.0038 mmol) and **L7** (1.7 mg, 0.0038 mmol) were added into a test tube under Ar, followed by the addition of dry toluene (1.0 mL). The mixture was stirred at rt for 36 h, and monitored by TLC. Product **3n** was obtained as a white solid by flash chromatography on silica gel (EtOAc/petroleum ether = 1/10–1/8), 31.0 mg, 81% yield; mp: 68–69 °C; >19:1 dr; 90% ee, determined by HPLC analysis [Daicel Chiral IC Column, *i*PrOH/*n*-hexane = 20/80, 1.0 mL/min, λ = 254 nm, t (major) = 14.83 min, t (minor) = 13.22 min]; $[\alpha]_D^{25}$ = –62.0 (*c* = 0.60, $CHCl_3$); **^{1}H NMR** (400 MHz, $CDCl_3$): δ (ppm) 7.86–7.62 (m, 4H), 7.26 (s, 1H), 7.12–6.95 (m, 5H), 6.30 (s, 1H), 6.22 (dd, *J* = 3.3, 1.9 Hz, 1H), 6.03 (d, *J* = 3.3 Hz, 1H), 5.31 (s, 1H), 4.43 (s, 1H), 4.00 (dd, *J* = 12.5, 4.8 Hz, 1H), 3.58 (dd, *J* = 17.1, 12.5 Hz, 1H), 2.95 (dd, *J* = 17.1, 4.8 Hz, 1H); **^{13}C NMR** (150 MHz, $CDCl_3$): δ (ppm) 201.6, 200.1, 198.9, 150.8, 142.9, 142.1, 142.0, 141.5, 138.2, 136.1, 135.9, 129.0, 128.7, 127.9, 124.8, 123.5, 123.4, 110.7, 109.7, 60.8, 45.2, 43.2, 42.2; **HRMS** (ESI-TOF) m/z: $[M + H]^+$ Calcd for $C_{25}H_{19}O_4^+$ 383.1278; Found 383.1277.

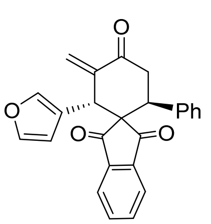

(2*R*,6*S*)-2-(Furan-3-yl)-3-methylene-6-phenylspiro[cyclohexane-1,2'-indene]-1',3',4-trione (3o): *tert*-Butyl (1-(furan-3-yl)-2-methylene-3-oxobutyl)carbonate **1o** (31.9 mg, 0.120 mmol), 2-benzylidene-1*H*-indene-1,3(2*H*)-dione **2a** (23.4 mg, 0.100 mmol), $Pd(OAc)_2$ (1.1 mg, 0.0050 mmol), **C4** (4.4 mg, 0.0075 mmol) and **L7** (3.4 mg, 0.0075 mmol) were added into a test tube under Ar, followed by the addition of dry toluene (1.0 mL). The mixture was stirred at rt for 36 h, and monitored by TLC. Product **3o** was obtained as a white solid by flash chromatography on silica gel (EtOAc/petroleum ether = 1/10–1/8), 33.9 mg, 88% yield; mp: 69–71 °C; >19:1 dr; 91% ee, determined by HPLC analysis [Daicel Chiral

IB Column, *i*PrOH/*n*-hexane = 10/90, 1.0 mL/min, λ = 254 nm, t (major) = 16.66 min, t (minor) = 15.71 min]; $[\alpha]_D^{25}$ = –32.9 (*c* = 1.67, $CHCl_3$); **^{1}H NMR** (400 MHz, $CDCl_3$): *δ* (ppm) 7.80 (d, *J* = 7.4 Hz, 1H), 7.73–7.64 (m, 1H), 7.64–7.60 (m, 2H), 7.15 (t, *J* = 1.8 Hz, 1H), 7.11–7.03 (m, 4H), 6.94–6.91 (m, 2H), 6.43 (dd, *J* = 2.4, 1.0 Hz, 1H), 6.17 (d, *J* = 1.8 Hz, 1H), 5.43 (d, *J* = 1.0 Hz 1H), 4.52 (t, *J* = 2.4 Hz, 1H), 3.73 (dd, *J* = 11.7, 4.0 Hz, 1H), 3.55 (dd, *J* = 16.5, 11.7 Hz, 1H), 2.91 (dd, *J* = 16.5, 4.0 Hz, 1H); **^{13}C NMR** (100 MHz, $CDCl_3$): *δ* (ppm) 202.3, 201.5, 198.6, 143.1, 142.9, 142.0, 141.8, 141.6, 137.7, 135.7, 128.5, 128.3, 127.6, 124.6, 122.94, 122.89, 119.5, 111.2, 61.9, 43.8, 41.3, 40.0; **HRMS** (ESI-TOF) m/z: $[M + Na]^+$ Calcd for $C_{25}H_{18}O_4Na^+$ 405.1097; Found 405.1096.

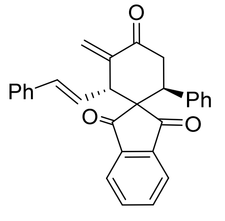

(2*R*,6*S*)-3-Methylene-6-phenyl-2-((*E*)-styryl)spiro[cyclohexane-1,2'-indene]-1',3',4-trione (3p): (*E*)-*tert*-Butyl (4-methylene-5-oxo-1-phenylhex-1-en-3-yl)carbonate **1p** (36.2 mg, 0.120 mmol), 2-benzylidene-1*H*-indene-1,3(2*H*)-dione **2a** (23.4 mg, 0.100 mmol), $Pd(OAc)_2$ (1.1 mg, 0.0050 mmol), **C4** (4.4 mg, 0.0075 mmol) and **L7** (3.4 mg, 0.0075 mmol) were added into a test tube under Ar, followed by the addition of dry toluene (1.0 mL). The mixture was stirred at rt for 36 h, and monitored by TLC. Product **3p** was obtained as a white solid by flash chromatography on silica gel (EtOAc/petroleum ether = 1/10–1/8), 30.4 mg, 75% yield; mp: 75–76 °C; >19:1 dr; 92% ee, determined by HPLC analysis [Daicel Chiral IF Column, *i*PrOH/*n*-hexane = 40/60, 1.0 mL/min, λ = 254 nm, t (major) = 11.18 min, t (minor) = 9.94 min]; $[\alpha]_D^{25}$ = –164.2 (*c* = 0.32, $CHCl_3$); **^{1}H NMR** (400 MHz, $CDCl_3$): *δ* (ppm) 7.88–7.79 (m, 1H), 7.75–7.60 (m, 3H), 7.26–7.17 (m, 5H), 7.09–6.93 (m, 5H), 6.36 (t, *J* = 1.4 Hz, 1H), 6.39–6.30 (m, 2H), 5.43 (t, *J* = 1.4 Hz, 1H), 4.01–3.91 (m, 1H), 3.81 (dd, *J* = 13.0, 4.3 Hz, 1H), 3.59 (dd, *J* = 16.9, 13.0 Hz, 1H), 2.80 (dd, *J* = 16.9, 4.3 Hz, 1H); **^{13}C NMR** (100 MHz, $CDCl_3$): *δ* (ppm) 201.9, 201.3, 199.1, 143.1, 141.82, 141.79, 137.8, 136.3, 135.84, 135.80, 134.8, 128.55, 128.48, 128.4, 128.0, 127.5, 126.6, 124.5, 123.9, 123.1, 123.0, 61.5, 47.4, 43.5, 41.5; **HRMS** (ESI-TOF) m/z: $[M + Na]^+$ Calcd for $C_{29}H_{22}O_3Na^+$ 441.1461; Found 441.1463.

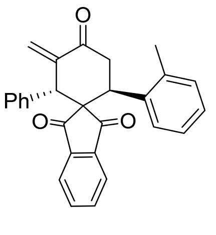

(2*R*,6*S*)-3-Methylene-2-phenyl-6-(o-tolyl)spiro[cyclohexane-1,2'-indene]-1',3',4-trione (3q): *tert*-Butyl (2-methylene-3-oxo-1-phenylbutyl)carbonate **1a** (33.2 mg, 0.120 mmol), 2-(2-methylbenzylidene)-1*H*-indene-1,3(2*H*)-dione **2b** (24.8 mg, 0.100 mmol), $Pd_2(dba)_3$ (2.3 mg, 0.0025 mmol), **C4** (4.4 mg, 0.0075

mmol) and **L7** (3.4 mg, 0.0075 mmol) were added into a test tube under Ar, followed by the addition of dry toluene (1.0 mL). The mixture was stirred at rt for 36 h, and monitored by TLC. Product **3q** was obtained as a white solid by flash chromatography on silica gel (EtOAc/petroleum ether = 1/10–1/8), 39.2 mg, 96% yield; mp: 144–147 °C; >19:1 dr; 94% ee, determined by HPLC analysis [Daicel Chiral ID Column, *i*PrOH/*n*-hexane = 20/80, 1.0 mL/min, λ = 254 nm, t (major) = 22.95 min, t (minor) = 15.39 min]; $[\alpha]_D^{25}$ = +29.8 (*c* = 0.34, $CHCl_3$); **^{1}H NMR** (400 MHz, $CDCl_3$): *δ* (ppm) 7.85–7.74 (m, 2H), 7.74–7.65 (m, 2H), 7.22–7.11 (m, 3H), 7.10–7.04 (m, 1H), 7.04–6.94 (m, 5H), 6.45 (t, *J* = 1.6 Hz, 1H), 5.27 (dd, *J* = 2.0, 1.6 Hz, 1H), 4.41 (t, *J* = 2.0 Hz, 1H), 4.14 (dd, *J* = 9.9, 6.0 Hz, 1H), 3.34 (dd, *J* = 17.8, 9.9 Hz, 1H), 3.06 (dd, *J* = 17.8, 6.0 Hz, 1H), 2.01 (s, 3H); **^{13}C NMR** (100 MHz, $CDCl_3$): *δ* (ppm) 201.1, 200.7, 198.5, 143.8, 141.4, 137.8, 137.5, 136.5, 135.81, 135.79, 130.7, 130.2, 128.2, 127.6, 127.4, 127.2, 126.3, 126.0, 123.20, 123.17, 60.9, 50.6, 42.7, 37.5, 19.9; **HRMS** (ESI-TOF) m/z: $[M + Na]^+$ Calcd for $C_{28}H_{22}O_3Na^+$ 429.1461; Found 429.1490.

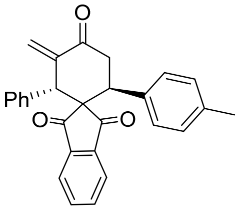

(2*R*,6*S*)-6-(4-Methylphenyl)-3-methylene-2-phenylspiro[cyclohexane-1,2'-indene]-1',3',4-trione (3r): *tert*-Butyl (2-methylene-3-oxo-1-phenylbutyl) carbonate **1a** (33.2 mg, 0.120 mmol), 2-(4-methylbenzylidene)-1*H*-indene-1,3(2*H*)-dione **2c** (24.8 mg, 0.100 mmol), $Pd(OAc)_2$ (0.60 mg, 0.0025 mmol), **C4** (2.2 mg, 0.0038 mmol) and **L7** (1.7 mg, 0.0038 mmol) were added into a test tube under Ar, followed by the addition of dry toluene (1.0 mL). The mixture was stirred at rt for 36 h, and monitored by TLC. The product **3r** was obtained as a white solid by flash chromatography on silica gel (EtOAc/petroleum ether = 1/10–1/8), 36.7 mg, 90% yield; mp: 172–173 °C; >19:1 dr; 93% ee, determined by HPLC analysis [Daicel Chiral IB Column, *i*PrOH/*n*-hexane = 20/80, 1.0 mL/min, λ = 254 nm, t (major) = 10.21 min, t (minor) = 12.20 min]; $[\alpha]_D^{25}$ = –39.5 (*c* = 0.50, $CHCl_3$); **^{1}H NMR** (400 MHz, $CDCl_3$): *δ* (ppm) 7.77–7.58 (m, 4H), 7.19–7.07 (m, 3H), 7.04–6.97 (m, 2H), 6.94–6.81 (m, 4H), 6.43 (t, *J* = 1.6 Hz, 1H), 5.30 (t, *J* = 1.6 Hz, 1H), 4.41 (t, *J* = 2.1 Hz, 1H), 3.79 (dd, *J* = 11.2, 5.0 Hz, 1H), 3.53 (dd, *J* = 17.1, 11.2 Hz, 1H), 3.02 (dd, *J* = 17.1, 5.0 Hz, 1H), 2.17 (s, 3H); **^{13}C NMR** (100 MHz, $CDCl_3$): *δ* (ppm) 201.4, 201.4, 198.7, 143.4, 141.8, 141.4, 137.1, 136.9, 135.6, 135.5, 135.1, 130.2, 129.0, 128.6, 128.3, 127.6, 125.2, 123.03, 123.00, 62.0, 50.4, 42.6, 41.8, 20.9; **HRMS** (ESI-TOF) m/z: $[M + Na]^+$ Calcd for $C_{28}H_{22}O_3Na^+$ 429.1461; Found 429.1460.

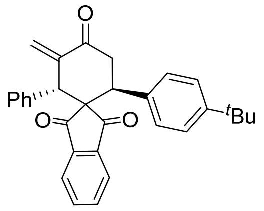

(2*R*,6*S*)-6-(4-(*tert*-Butyl)phenyl)-3-methylene-2-phenylspiro[cyclohexane-1,2'-indene]-1',3',4-trione (3s): *tert*-Butyl (2-methylene-3-oxo-1-phenyl butyl)carbonate **1a** (33.2 mg, 0.120 mmol), 2-(4-(*tert*-butyl)benzylidene)-1*H*-indene-1,3(2*H*)-dione **2d** (29.0 mg, 0.100 mmol), $Pd(OAc)_2$ (1.1 mg, 0.0050 mmol), **C4** (4.4 mg, 0.0075 mmol) and **L7** (3.4 mg, 0.0075 mmol) were added into a test tube under Ar, followed by the addition of dry toluene (1.0 mL). The mixture was stirred at rt for 36 h, and monitored by TLC. Product **3s** was obtained as a white solid by flash chromatography on silica gel (EtOAc/petroleum ether = 1/10–1/8), 38.5 mg, 90% yield; mp: 174–175 °C; >19:1 dr; 93% ee, determined by HPLC analysis [Daicel Chiral AD-H Column, *i*PrOH/*n*-hexane = 30/70, 1.0 mL/min, λ = 254 nm, t (major) = 6.92 min, t (minor) = 8.79 min]; $[\alpha]_D^{25}$ = –35.6 (*c* = 0.75, $CHCl_3$); **^{1}H NMR** (400 MHz, $CDCl_3$): *δ* (ppm) 7.81–7.71 (m, 1H), 7.66–7.58 (m, 3H), 7.20–6.98 (m, 7H), 6.92–6.75 (m, 2H), 6.43 (s, 1H), 5.32 (s, 1H), 4.45 (s, 1H), 3.79 (dd, *J* = 11.3, 4.8 Hz, 1H), 3.55 (dd, *J* = 17.1, 11.3 Hz, 1H), 3.02 (dd, *J* = 17.1, 4.8 Hz, 1H), 1.16 (s, 9H); **^{13}C NMR** (100 MHz, $CDCl_3$): *δ* (ppm) 201.5, 201.4, 198.8, 150.3, 143.4, 141.9, 141.4, 136.8, 135.5, 135.4, 134.9, 130.3, 128.34, 128.32, 127.6, 125.13, 125.11, 123.0, 62.2, 50.2, 42.8, 41.7, 34.3, 31.1; **HRMS** (ESI-TOF) m/z: $[M + Na]^+$ Calcd for $C_{31}H_{28}O_3Na^+$ 471.1931; Found 471.1944.

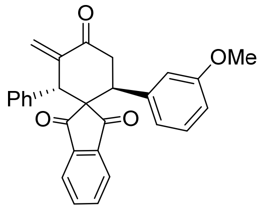

(2*R*,6*S*)-6-(3-Methoxyphenyl)-3-methylene-2-phenylspiro[cyclohexane-1,2'-indene]-1',3',4-trione (3t): *tert*-Butyl (2-methylene-3-oxo-1-phenylbutyl) carbonate **1a** (33.2 mg, 0.120 mmol), 2-(3-methoxybenzylidene)-1*H*-indene-1,3(2*H*)-dione **2e** (26.4 mg, 0.100 mmol), $Pd(OAc)_2$ (1.1 mg, 0.0050 mmol), **C4** (4.4 mg, 0.0075 mmol) and **L7** (3.4 mg, 0.0075 mmol) were added into a test tube under Ar, followed by the addition of dry toluene (1.0 mL). The mixture was stirred at rt for 36 h, and monitored by TLC. Product **3t** was obtained as a white solid by flash chromatography on silica gel (EtOAc/petroleum ether = 1/10–1/8), 35.7 mg, 82% yield; mp: 120–121 °C; >19:1 dr; 96% ee, determined by HPLC analysis [Daicel Chiral IA Column, *i*PrOH/*n*-hexane = 10/90, 1.0 mL/min, λ = 254 nm, t (major) = 23.94 min, t (minor) = 21.19 min]; $[\alpha]_D^{25}$ = –21.3 (*c* = 0.33, $CHCl_3$); **^{1}H NMR** (400 MHz, $CDCl_3$): *δ* (ppm) 7.75–7.71 (m, 1H), 7.71–7.68 (m, 1H), 7.67–7.61 (m, 2H), 7.19–7.09 (m, 3H), 7.05–6.97 (m, 3H), 6.60 (ddd, *J* = 8.0, 2.4, 0.8 Hz, 1H), 6.56 (dt, *J* = 8.0, 1.2 Hz, 1H), 6.50 (t, *J* = 2.2 Hz, 1H), 6.43 (dd, *J* = 1.6, 1.2 Hz, 1H), 5.33 (dd, *J* = 2.2, 1.2 Hz, 1H), 4.43 (t, *J* = 2.0 Hz,

1H), 3.83 (dd, *J* = 11.8, 4.8 Hz, 1H), 3.63 (s, 3H), 3.58 (dd, *J* = 17.0, 11.8 Hz, 1H), 3.01 (dd, *J* = 17.0, 4.8 Hz, 1H); **^{13}C NMR** (100 MHz, $CDCl_3$): *δ* (ppm) 201.5, 201.2, 198.6, 159.3, 143.2, 141.8, 141.4, 139.6, 136.8, 135.7, 135.6, 130.2, 129.3, 128.3, 127.7, 125.3, 123.04, 122.99, 121.0, 114.4, 113.1, 61.9, 55.1, 50.6, 43.0, 41.7; **HRMS** (ESI-TOF) m/z: $[M + Na]^+$ Calcd for $C_{28}H_{22}O_4Na^+$ 445.1410; Found 445.1419.

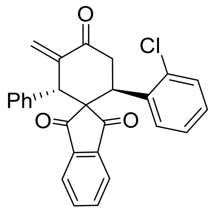

(2*R*,6*S*)-6-(2-Chlorophenyl)-3-methylene-2-phenylspiro[cyclohexane-1,2'-indene]-1',3',4-trione (3u): *tert*-Butyl (2-methylene-3-oxo-1-phenylbutyl) carbonate **1a** (33.2 mg, 0.120 mmol), 2-(2-chlorobenzylidene)-1*H*-indene-1,3(2*H*)-dione **2f** (26.9 mg, 0.100 mmol), $Pd(OAc)_2$ (1.1 mg, 0.0050 mmol), **C4** (4.4 mg, 0.0075 mmol) and **L7** (3.4 mg, 0.0075 mmol) were added into a test tube under Ar, followed by the addition of dry toluene (1.0 mL). The mixture was stirred at rt for 36 h, and monitored by TLC. Product **3u** was obtained as a white solid by flash chromatography on silica gel (EtOAc/petroleum ether = 1/10–1/8), 24.0 mg, 56% yield; mp: 167–168 °C; >19:1 dr; 81% ee, determined by HPLC analysis [Daicel Chiral AD-H Column, *i*PrOH/*n*-hexane = 10/90, 1.0 mL/min, λ = 254 nm, t (major) = 15.64 min, t (minor) = 14.71 min]; $[\alpha]_D^{25}$ = +39.2 (*c* = 0.26, $CHCl_3$); **^{1}H NMR** (400 MHz, $CDCl_3$): *δ* (ppm) 7.90 (dt, *J* = 7.5, 1.1 Hz, 1H), 7.78–7.64 (m, 3H), 7.35–7.27 (m, 2H), 7.25–7.15 (m, 2H), 7.12–6.94 (m, 5H), 6.53 (dd, *J* = 2.8, 1.3 Hz, 1H), 5.19 (dd, *J* = 2.8, 1.3 Hz, 1H), 4.48 (t, *J* = 2.8 Hz, 1H), 4.25 (dd, *J* = 7.2, 4.8 Hz, 1H), 3.42 (dd, *J* = 18.1, 7.2 Hz, 1H), 2.99 (dd, *J* = 18.1, 4.8 Hz, 1H); **^{13}C NMR** (100 MHz, $CDCl_3$): *δ* (ppm) 201.7, 199.0, 197.8, 144.1, 141.9, 141.0, 137.2, 136.8, 135.9, 135.6, 135.0, 130.6, 129.7, 129.3, 128.9, 128.3, 127.6, 127.2, 126.4, 123.3, 123.2, 60.0, 48.2, 41.6, 39.1; **HRMS** (ESI-TOF) m/z: $[M + Na]^+$ Calcd for $C_{27}H_{19}O_3ClNa^+$ 449.0915 (^{35}Cl) and 451.0885 (^{37}Cl); Found 449.0916 (^{35}Cl) and 451.0895 (^{37}Cl).

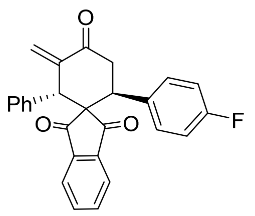

(2*R*,6*S*)-6-(4-Fluorophenyl)-3-methylene-2-phenylspiro[cyclohexane-1,2'-indene]-1',3',4-trione (3v): *tert*-Butyl (2-methylene-3-oxo-1-phenylbutyl) carbonate **1a** (33.2 mg, 0.120 mmol), 2-(4-fluorobenzylidene)-1*H*-indene-1,3(2*H*)-dione **2g** (25.2 mg, 0.100 mmol), $Pd(OAc)_2$ (1.1 mg, 0.0050 mmol), **C4** (4.4 mg, 0.0075 mmol) and **L7** (3.4 mg, 0.0075 mmol) were added into a test tube under Ar, followed by the addition of dry toluene (1.0 mL). The mixture was stirred at rt for 36 h, and monitored

by TLC. Product **3v** was obtained as a white solid by flash chromatography on silica gel (EtOAc/petroleum ether = 1/10–1/8), 33.1 mg, 80% yield; mp: 124–125 °C; >19:1 dr; 92% ee, determined by HPLC analysis [Daicel Chiral IA Column, *i*PrOH/*n*-hexane = 40/60, 0.8 mL/min, λ = 254 nm, t (major) = 9.98 min, t (minor) = 9.33 min]; $[\alpha]_D^{25}$ = −18.5 (*c* = 0.26, $CHCl_3$); **^{1}H NMR** (400 MHz, $CDCl_3$): *δ* (ppm) 7.76–7.57 (m, 4H), 7.22–7.10 (m, 3H), 7.04–6.90 (m, 4H), 6.80–6.74 (m, 2H), 6.43 (s, 1H), 5.34 (s, 1H), 4.39 (s, 1H), 3.85 (dd, *J* = 12.0, 4.7 Hz, 1H), 3.58 (dd, *J* = 17.0, 12.0 Hz, 1H), 2.98 (dd, *J* = 17.0, 4.7 Hz, 1H); **^{13}C NMR** (100 MHz, $CDCl_3$): *δ* (ppm) 201.5, 201.1, 198.3, 161.9 (d, *J* = 246.7 Hz), 143.1, 141.9, 141.3, 136.6, 135.8, 135.7, 133.9 (d, *J* = 3.4 Hz), 130.3 (d, *J* = 8.1 Hz), 130.2, 128.4, 127.8, 125.4, 123.0, 123.0, 115.2 (d, *J* = 21.4 Hz), 62.0, 50.7, 42.1, 41.8; **^{19}F NMR** (376 MHz, $CDCl_3$): δ (ppm) −114.5; **HRMS** (ESI-TOF) m/z: $[M + Na]^+$ Calcd for $C_{27}H_{19}O_3FNa^+$ 433.1210; Found 433.1237.

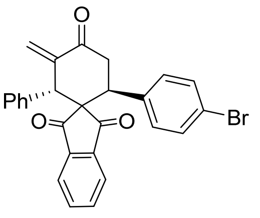

(2*R*,6*S*)-6-(4-Bromophenyl)-3-methylene-2-phenylspiro[cyclohexane-1,2'-indene]-1',3',4-trione (3w): *tert*-Butyl (2-methylene-3-oxo-1-phenylbutyl) carbonate **1a** (33.2 mg, 0.120 mmol), 2-(4-bromobenzylidene)-1*H*-indene-1,3(2*H*)-dione **2h** (31.3 mg, 0.100 mmol), $Pd(OAc)_2$ (1.1 mg, 0.0050 mmol), **C4** (4.4 mg, 0.0075 mmol) and **L7** (3.4 mg, 0.0075 mmol) were added into a test tube under Ar, followed by the addition of dry toluene (1.0 mL). The mixture was stirred at rt for 36 h, and monitored by TLC. Product **3w** was obtained as a pale yellow solid by flash chromatography on silica gel (EtOAc/petroleum ether = 1/10–1/8), 36.4 mg, 77% yield; mp: 153–154 °C; >19:1 dr; 95% ee, determined by HPLC analysis [Daicel Chiral IA Column, *i*PrOH/*n*-hexane = 20/80, 1.0 mL/min, λ = 254 nm, t (major) = 14.20 min, t (minor) = 13.50 min]; $[\alpha]_D^{25}$ = –35.1 (*c* = 0.29, $CHCl_3$); **^{1}H NMR** (400 MHz, $CDCl_3$): *δ* (ppm) 7.79–7.61 (m, 4H), 7.24–7.09 (m, 5H), 7.04–6.97 (m, 2H), 6.90–6.81 (m, 2H), 6.43 (s, 1H), 5.33 (s, 1H), 4.37 (s, 1H), 3.83 (dd, *J* = 11.8, 4.8 Hz, 1H), 3.56 (dd, *J* = 17.1, 11.8 Hz, 1H), 2.99 (dd, *J* = 17.1, 4.8 Hz, 1H); **^{13}C NMR** (100 MHz, $CDCl_3$): *δ* (ppm) 201.4, 200.8, 198.2, 143.0, 141.7, 141.2, 137.2, 136.6, 136.0, 135.8, 131.5, 130.4, 130.1, 128.4, 127.8, 125.6, 123.2, 123.1, 121.6, 61.7, 50.9, 42.0, 41.5; **HRMS** (ESI-TOF) m/z: $[M + K]^+$ Calcd for $C_{27}H_{19}O_3BrK^+$ 509.1049 (^{79}Br) and 511.0129 (^{81}Br); Found 509.1048 (^{79}Br) and 511.0138 (^{81}Br).

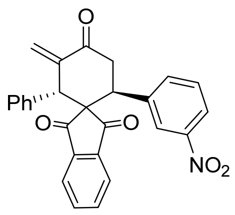

(2*R*,6*S*)-3-Methylene-6-(3-nitrophenyl)-2-phenylspiro[cyclohexane-1,2'-indene]-1',3',4-trione (3x): *tert*-Butyl (2-methylene-3-oxo-1-phenylbutyl) carbonate **1a** (33.2 mg, 0.120 mmol), 2-(3-nitrobenzylidene)-1*H*-indene-1,3(2*H*)-dione **2i** (31.3 mg, 0.100 mmol), $Pd(OAc)_2$ (1.1 mg, 0.0050 mmol), **C4** (4.4 mg, 0.0075 mmol) and **L7** (3.4 mg, 0.0075 mmol) were added into a test tube under Ar, followed by the addition of dry toluene (1.0 mL). The mixture was stirred at rt for 36 h, and monitored by TLC. Product **3x** was obtained as a white solid by flash chromatography on silica gel (EtOAc/petroleum ether = 1/10–1/8), 26.7 mg, 61% yield; mp: 86–88 °C; >19:1 dr; 92% ee, determined by HPLC analysis [Daicel Chiral IC Column, *i*PrOH/*n*-hexane = 40/60, 1.0 mL/min, λ = 254 nm, t (major) = 19.43 min, t (minor) = 15.94 min]; $[\alpha]_D^{25} = -16.5$ (*c* = 0.40, $CHCl_3$); **^{1}H NMR** (400 MHz, $CDCl_3$): *δ* (ppm) 7.96 (dt, *J* = 7.9, 1.9 Hz, 1H), 7.86 (t, *J* = 1.9 Hz, 1H), 7.77–7.66 (m, 4H), 7.40–7.28 (m, 2H), 7.22–7.09 (m, 3H), 7.00–6.96 (m, 2H), 6.46 (s, 1H), 5.37 (s, 1H), 4.38 (s, 1H), 3.98 (dd, *J* = 11.6, 5.1 Hz, 1H), 3.61 (dd, *J* = 17.2, 11.6 Hz, 1H), 3.07 (dd, *J* = 17.2, 5.1 Hz, 1H); **^{13}C NMR** (100 MHz, $CDCl_3$): *δ* (ppm) 201.0, 200.4, 197.5, 148.0, 142.7, 141.7, 141.0, 140.5, 136.4, 136.2, 136.0, 134.7, 130.1, 129.4, 128.5, 128.0, 126.1, 123.9, 123.31, 123.26, 122.7, 61.5, 51.0, 42.0, 41.3; **HRMS** (ESI-TOF) m/z: $[M + Na]^+$ Calcd for $C_{27}H_{19}NO_5Na^+$ 460.1155; Found 460.1153.

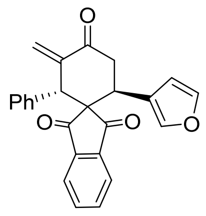

(2*R*,6*S*)-6-(Furan-3-yl)-3-methylene-2-phenylspiro[cyclohexane-1,2'-indene]-1',3',4-trione (3y): *tert*-Butyl (2-methylene-3-oxo-1-phenylbutyl) carbonate **1a** (33.2 mg, 0.120 mmol), 2-(furan-3-ylmethylene)-1*H*-indene-1,3(2*H*)-dione **2j** (22.4 mg, 0.100 mmol), $Pd(OAc)_2$ (1.1 mg, 0.0050 mmol), **C4** (4.4 mg, 0.0075 mmol) and **L7** (3.4 mg, 0.0075 mmol) were added into a test tube under Ar, followed by the addition of dry toluene (1.0 mL). The mixture was stirred at rt for 36 h, and monitored by TLC. Product **3y** was obtained as a white solid by flash chromatography on silica gel (EtOAc/petroleum ether = 1/10–1/8), 35.9 mg, 93% yield; mp: 139–140 °C; >19:1 dr; 85% ee, determined by HPLC analysis [Daicel Chiral AD-H Column, *i*PrOH/*n*-hexane = 20/80, 1.0 mL/min, λ = 254 nm, t (major) = 13.44 min, t (minor) = 10.93 min]; $[\alpha]_D^{25} = -12.7$ (*c* = 0.22, $CHCl_3$); **^{1}H NMR** (400 MHz, $CDCl_3$): *δ* (ppm) 7.84–7.78 (m, 2H), 7.76–7.69 (m, 2H), 7.23–7.14 (m, 3H), 7.13 (t, *J* = 1.6 Hz, 1H), 7.07–7.06 (m, 1H), 7.02–6.95 (m, 2H), 6.36 (t, *J* = 1.3 Hz, 1H), 6.05 (dd, *J* = 2.0, 0.8 Hz, 1H), 5.28 (dd, *J* = 2.0, 1.3 Hz, 1H), 4.30 (t, *J* = 1.6 Hz, 1H), 3.82 (dd, *J* = 11.3, 5.3 Hz, 1H), 3.41 (dd, *J* = 17.4, 11.3 Hz, 1H), 3.04

(dd, J = 17.4, 5.3 Hz, 1H); **^{13}C NMR** (100 MHz, $CDCl_3$): δ (ppm) 201.4, 200.7, 198.5, 143.3, 143.0, 141.8, 141.2, 140.5, 137.5, 135.9, 135.8, 129.9, 128.4, 127.7, 125.4, 123.3, 123.2, 122.6, 110.2, 61.1, 50.9, 41.9, 33.2; **HRMS** (ESI-TOF) m/z: $[M + Na]^+$ Calcd for $C_{25}H_{14}O_4Na^+$ 405.1097; Found 405.1099.

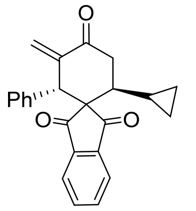

(2*R*,6*S*)-6-Cyclopropyl-3-methylene-2-phenylspiro[cyclohexane-1,2'-indene]-1',3',4-trione (3z): *tert*-Butyl (2-methylene-3-oxo-1-phenylbutyl)carbonate **1a** (33.2 mg, 0.120 mmol), 2-(cyclopropylmethylene)-1*H*-indene-1,3(2*H*)-dione **2k** (19.8 mg, 0.100 mmol), Pd(η^3-allyl)Cp (1.1 mg, 0.0050 mmol), **C4** (4.4 mg, 0.0075 mmol) and **L7** (3.4 mg, 0.0075 mmol) were added into a test tube under Ar followed by the addition of dry toluene (1.0 mL). The mixture was stirred at rt for 36 h, and monitored by TLC. Product **3z** was obtained as a white solid by flash chromatography on silica gel (EtOAc/petroleum ether = 1/10–1/8), 34.6 mg, 96% yield; mp: 109−110 °C; 15:1 dr; 61% ee, determined by HPLC analysis [Daicel Chiral AD-H Column, *i*PrOH/*n*-hexane = 10/90, 1.0 mL/min, λ = 254 nm, t (major) = 12.60 min, t (minor) = 8.12 min]; $[\alpha]_D^{25}$ = +14.7 (c = 0.43, $CHCl_3$); **^{1}H NMR** (400 MHz, $CDCl_3$): δ (ppm) 7.86–7.80 (m, 2H), 7.76–7.67 (m, 2H), 7.18–7.04 (m, 3H), 7.03–6.96 (m, 2H), 6.34 (dd, J = 2.3, 1.1 Hz, 1H), 5.23 (dd, J = 2.3, 1.1 Hz, 1H), 4.48 (t, J = 2.1 Hz, 1H), 3.08 (dd, J = 16.6, 10.6 Hz, 1H), 2.91 (dd, J = 16.6, 4.8 Hz, 1H), 1.76 (td, J = 10.6, 4.8 Hz, 1H), 0.85–0.76 (m, 1H), 0.49–0.39 (m, 1H), 0.21–0.09 (m, 1H), 0.09–0.00 (m, 1H), –0.12—0.27 (m, 1H); **^{13}C NMR** (100 MHz, $CDCl_3$): δ (ppm) 202.7, 202.0, 198.9, 143.3, 142.2, 141.6, 136.3, 135.7, 135.6, 130.2, 128.3, 127.6, 124.5, 123.0, 61.3, 50.3, 43.3, 42.1, 13.4, 5.8, 4.0; **HRMS** (ESI-TOF) m/z: $[M + Na]^+$ Calcd for $C_{24}H_{20}O_3Na^+$ 379.1305; Found 379.1324.

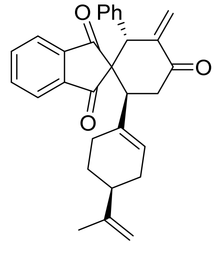

(2*R*,6*S*)-6-((*S*)-Perill)-3-methylene-2-phenylspiro[cyclohexane-1,2'-indene]-1',3',4-trione (3aa): *tert*-Butyl (2-methylene-3-oxo-1-phenylbutyl)carbonate **1a** (33.2 mg, 0.120 mmol), (*S*)-2-((4-(prop-1-en-2-yl)cyclohex-1-en-1-yl)methylene)-1*H*-indene-1,3(2*H*)-dione **2l** (27.8 mg, 0.100 mmol), Pd(η^3-allyl)Cp (1.1 mg, 0.0050 mmol), **C4** (4.4 mg, 0.0075 mmol) and **L7** (3.4 mg, 0.0075 mmol) were added into a test tube under Ar, followed by the addition of dry toluene (1.0 mL). The mixture was stirred at rt for 36 h, and monitored by TLC. Product **3aa** was obtained as a semi-solid by flash

chromatography on silica gel (EtOAc/petroleum ether = 1/10–1/8), 34.1 mg, 73% yield; 8:1 dr by ^{1}H NMR analysis; $[\alpha]_D^{25}$ = +8.6 (*c* = 0.07, $CHCl_3$); **^{1}H NMR** (400 MHz, $CDCl_3$): δ (ppm) 7.96–7.78 (m, 2H), 7.78–7.67 (m, 2H), 7.16–7.06 (m, 3H), 7.06–6.93 (m, 2H), 6.37–6.35 (m, 1H), 5.53–5.32 (m, 1H), 5.32–5.20 (m, 1H), 4.61 (t, *J* = 1.7 Hz, 1H), 4.53 (s, 1H), 4.46 (d, *J* = 2.3 Hz, 1H), 3.17 (dd, *J* = 16.3, 10.3 Hz, 1H), 3.07 (dd, *J* = 10.3, 4.6 Hz, 1H), 2.86 (dd, *J* = 16.3, 4.6 Hz, 1H), 2.01–1.66 (m, 5H), 1.61 (s, 3H), 1.59–1.54 (m, 1H), 1.20–1.04 (m, 1H); **^{13}C NMR** (100 MHz, $CDCl_3$): δ (ppm) 201.8, 201.4, 199.2, 149.2, 143.5, 141.7, 141.5, 136.8, 135.71, 135.65, 135.3, 130.4, 128.3, 127.6, 126.9, 124.8, 123.13, 123.08, 108.7, 61.1, 50.1, 44.5, 41.3, 40.3, 30.6, 28.8, 27.4, 20.7; **HRMS** (ESI-TOF) m/z: $[M + Na]^+$ Calcd for $C_{30}H_{28}O_3Na^+$ 459.1931; Found 459.1932.

5.2 Regiodivergent asymmetric [4+2] annulations with MBH carbonate 1q

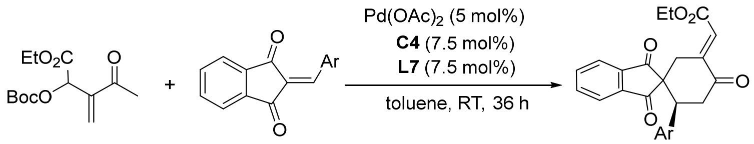

The reaction was conducted with MBH carbonate **1q** (0.12 mmol), enone **2** (0.10 mmol), $Pd(OAc)_2$ (0.005 mmol), **C4** (0.0075 mmol) and **L7** (0.0075 mmol) in dry toluene (1.0 mL) under Ar, and the mixture was stirred at room temperature for 36 h. After completion, the product was obtained by flash chromatography on silica gel (THF/petroleum ether = 1/15–1/10). The racemates were obtained similarly by using the combination of achiral tetrabutylammonium bromide (TBAB) and triphenyl phosphite.

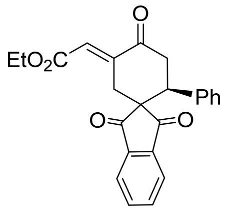

Ethyl (*S*,*E*)-2-(2-(4-phenyl)-1',3',4-trioxo-1',3'-dihydrospiro[cyclohexane-1,2'-inden]-5-ylidene)acetate (4a): Ethyl 2-((*tert*-butoxycarbonyl)oxy)-3-methylene-4-oxopentanoate **1q** (32.6 mg, 0.120 mmol), 2-benzylidene-1*H*-indene-1,3(2*H*)-dione **2a** (23.4 mg, 0.100 mmol), $Pd(OAc)_2$ (1.1 mg, 0.0050 mmol), **C4** (4.4 mg, 0.0075 mmol) and **L7** (3.4 mg, 0.0075 mmol) were added into a test tube under Ar, followed by the addition of dry toluene (1.0 mL). The mixture was stirred at rt for 36 h, and monitored by TLC. Product **4a** was obtained as a colorless oil, 30.2 mg, 82% yield; 90% ee, determined by HPLC analysis [Daicel Chiral AD-H Column, *i*PrOH/*n*-hexane = 40/60, 1.0 mL/min, λ = 254 nm, t (major) = 27.87 min, t (minor) = 9.01 min]; $[\alpha]_D^{25}$ = +76.0 (*c* = 0.10, $CHCl_3$); **^{1}H NMR** (400 MHz, $CDCl_3$): δ (ppm) 7.87–7.77 (m, 1H), 7.77–7.55 (m, 3H), 7.07–6.96 (m, 5H), 6.76 (dd, *J*

= 3.2, 1.4 Hz, 1H), 4.19–4.10 (m, 2H), 3.88 (d, *J* = 18.0 Hz, 1H), 3.82 (dd, *J* = 14.0, 5.0 Hz, 1H), 3.62 (dd, *J* = 17.6, 14.0 Hz, 1H), 3.14 (dd, *J* = 18.0, 3.2 Hz, 1H), 2.87 (dd, *J* = 17.6, 5.0 Hz, 1H), 1.26 (t, *J* = 7.1 Hz, 3H); ^{13}**C NMR** (100 MHz, $CDCl_3$): *δ* (ppm) 202.3, 201.0, 198.0, 165.8, 146.5, 141.5, 141.3, 137.4, 135.9, 128.5, 127.9, 127.8, 124.4, 123.2, 123.0, 60.7, 56.7, 45.7, 41.8, 32.6, 14.1; **HRMS** (ESI-TOF) m/z: $[M + Na]^+$ Calcd for $C_{24}H_{20}O_5Na^+$ 411.1203; Found 411.1206.

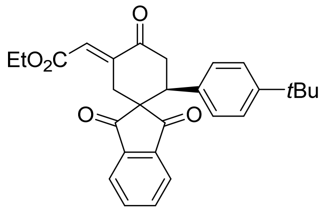

Ethyl (*S*,*E*)-2-(2-(4-(*tert*-butyl)phenyl)-1',3',4-trioxo-1',3'-dihydro spiro[cyclohexane-1,2'-inden]-5-ylidene)acetate (4b): Ethyl 2-((*tert*-butoxycarbonyl)oxy)-3-methylene-4-oxopentanoate **1q** (32.6 mg, 0.120 mmol), 2-(4-(*tert*-butyl)benzylidene)-1*H*-indene-1,3(2*H*)-dione **2d** (29.0 mg, 0.100 mmol), $Pd(OAc)_2$ (1.1 mg, 0.0050 mmol), **C4** (4.4 mg, 0.0075 mmol) and **L7** (3.4 mg, 0.0075 mmol) were added into a test tube under Ar, followed by the addition of dry toluene (1.0 mL). The mixture was stirred at rt for 36 h, and monitored by TLC. Product **4b** was obtained as a white solid, 31.2 mg, 70% yield; mp: 111−113 °C; 93% ee, determined by HPLC analysis [Daicel Chiral IF Column, *i*PrOH/*n*-hexane = 40/60, 1.0 mL/min, λ = 254 nm, t (major) = 13.74 min, t (minor) = 12.78 min]; $[\alpha]_D^{25}$ = +61.1 (*c* = 0.36, $CHCl_3$); ^{1}**H NMR** (400 MHz, $CDCl_3$): *δ* (ppm) 7.81 (dt, *J* = 7.0, 1.4 Hz, 1H), 7.74–7.57 (m, 3H), 7.02 (d, *J* = 8.5 Hz, 2H), 6.95–6.83 (m, 2H), 6.76 (dd, *J* = 3.2, 1.4 Hz, 1H), 4.21−4.09 (m, 2H), 3.87 (dd, *J* = 17.9, 1.4 Hz, 1H), 3.79 (dd, *J* = 14.0, 4.9 Hz, 1H), 3.59 (dd, *J* = 17.7, 14.0 Hz, 1H), 3.14 (dd, *J* = 17.9, 3.2 Hz, 1H), 2.86 (dd, *J* = 17.7, 4.9 Hz, 1H), 1.26 (t, *J* = 7.0 Hz, 3H), 1.09 (s, 9H); ^{13}**C NMR** (150 MHz, $CDCl_3$): *δ* (ppm) 202.3, 201.1, 198.1, 165.7, 150.6, 146.6, 141.5, 141.3, 135.6, 135.5, 134.1, 127.4, 125.2, 124.3, 123.1, 122.9, 60.6, 56.9, 45.4, 41.7, 34.2, 32.2, 30.9, 14.0; **HRMS** (ESI-TOF) m/z: $[M + Na]^+$ Calcd for $C_{28}H_{28}O_5Na^+$ 467.1829; Found 467.1827.

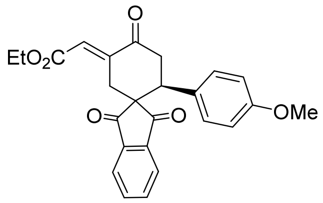

Ethyl (*S*,*E*)-2-(2-(4-(methoxyl)phenyl)-1',3',4-trioxo-1',3'-dihydro spiro[cyclohexane-1,2'-inden]-5-ylidene)acetate (4c): Ethyl 2-((*tert*-butoxycarbonyl)oxy)-3-methylene-4-oxopentanoate **1q** (32.6 mg, 0.120 mmol), 2-(3-methoxybenzylidene)-1*H*-indene-1,3(2*H*)-dione **2e** (26.4 mg, 0.100 mmol), $Pd(OAc)_2$ (1.1 mg, 0.0050 mmol), **C4** (4.4 mg, 0.0075 mmol) and **L7** (3.4 mg, 0.0075 mmol) were added into a test tube under Ar, followed by the addition of dry toluene (1.0 mL).

The mixture was stirred at rt for 36 h, and monitored by TLC. Product **4c** was obtained as a colorless oil, 27.6 mg, 66% yield; 92% ee, determined by HPLC analysis [Daicel Chiral AD-H Column, *i*PrOH/*n*-hexane = 40/60, 1.0 mL/min, λ = 254 nm, t (major) = 26.62 min, t (minor) = 10.60 min]; $[\alpha]_D^{25}$ = +45.7 (*c* = 0.21, $CHCl_3$); **^{1}H NMR** (400 MHz, $CDCl_3$): δ (ppm) 7.93–7.80 (m, 1H), 7.79–7.64 (m, 3H), 6.93–6.91 (m, 2H), 6.75 (dd, *J* = 3.2, 1.4 Hz, 1H), 6.58–6.55 (m, 2H), 4.18–4.10 (m, 2H), 3.87 (dd, *J* = 17.8, 1.4 Hz, 1H), 3.77 (dd, *J* = 14.1, 5.0 Hz, 1H), 3.62 (s, 3H), 3.59–3.54 (m, 1H), 3.10 (dd, *J* = 17.8, 3.2 Hz, 1H), 2.84 (dd, *J* = 17.8, 5.0 Hz, 1H), 1.25 (t, *J* = 7.1 Hz, 3H); **^{13}C NMR** (100 MHz, $CDCl_3$): δ 202.6, 201.3, 198.2, 165.8, 158.8, 146.7, 141.5, 141.4, 135.94, 135.92, 129.5, 129.0, 124.4, 123.3, 123.1, 113.8, 60.7, 56.9, 55.1, 44.9, 42.2, 32.5, 14.1; **HRMS** (ESI-TOF) m/z: $[M + Na]^+$ Calcd for $C_{25}H_{22}O_6Na^+$ 441.1309; Found 441.1306.

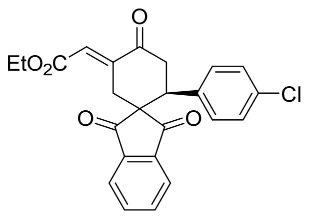

Ethyl (*S*,*E*)-2-(2-(4-(chloro)phenyl)-1',3',4-trioxo-1',3'-dihydrospiro [cyclohexane-1,2'-inden]-5-ylidene)acetate (4d): Ethyl 2-((*tert*-butoxycarbonyl)oxy)-3-methylene-4-oxopentanoate **1q** (32.6 mg, 0.120 mmol), 2-(4-chlorobenzylidene)-1*H*-indene-1,3(2*H*)-dione **2m** (26.9 mg, 0.100 mmol), $Pd(OAc)_2$ (1.1 mg, 0.0050 mmol), **C4** (4.4 mg, 0.0075 mmol) and **L7** (3.4 mg, 0.0075 mmol) were added into a test tube under Ar, followed by the addition of dry toluene (1.0 mL). The mixture was stirred at rt for 36 h, and monitored by TLC. Product **4d** was obtained as a colorless oil, 35.2 mg, 83% yield; 90% ee, determined by HPLC analysis [Daicel Chiral IA Column, *i*PrOH/*n*-hexane = 40/60, 1.0 mL/min, λ = 254 nm, t (major) = 17.02 min, t (minor) = 8.62 min]; $[\alpha]_D^{25}$ = +34.5 (*c* = 0.29, $CHCl_3$); **^{1}H NMR** (400 MHz, $CDCl_3$): δ (ppm) 7.86–7.83 (m, 1H), 7.80–7.67 (m, 3H), 7.06–7.00 (m, 2H), 6.99–6.90 (m, 2H), 6.76 (dd, *J* = 3.2, 1.3 Hz, 1H), 4.20–4.08 (m, 2H), 3.90 (dd, *J* = 17.9, 1.3 Hz, 1H), 3.81 (dd, *J* = 14.0, 5.2 Hz, 1H), 3.58 (dd, *J* = 17.7, 14.0 Hz, 1H), 3.10 (dd, *J* = 17.9, 3.2 Hz, 1H), 2.85 (dd, *J* = 17.7, 5.2 Hz, 1H), 1.25 (t, *J* = 7.1 Hz, 3H); **^{13}C NMR** δ (ppm) (150 MHz, $CDCl_3$): δ 202.0, 200.7, 197.5, 165.6, 146.1, 141.2, 141.1, 136.1, 135.9, 133.5, 129.2, 128.6, 124.6, 123.3, 123.1, 60.7, 56.4, 44.7, 41.7, 32.6, 14.0; **HRMS** (ESI-TOF) m/z: $[M + Na]^+$ Calcd for $C_{24}H_{19}O_5NaCl^+$ 445.0813 (^{35}Cl) and 447.0784 (^{37}Cl); Found 445.0812 (^{35}Cl) and 447.0792 (^{37}Cl).

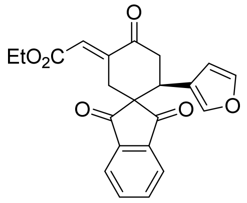

Ethyl (*S*,*E*)-2-(2-(furan-3-yl)-1',3',4-trioxo-1',3'-dihydrospiro[cyclohexane-1,2'-inden]-5-ylidene)acetate (4e): Ethyl 2-((*tert*-butoxycarbonyl)oxy)-3-methylene-4-oxopentanoate **1q** (32.6 mg, 0.120 mmol), 2-(furan-3-ylmethylene)-1*H*-indene-1,3(2*H*)-dione **2j** (22.4 mg, 0.100 mmol), $Pd(OAc)_2$ (1.1 mg, 0.0050 mmol), **C4** (4.4 mg, 0.0075 mmol) and **L7** (3.4 mg, 0.0075 mmol) were added into a test tube under Ar, followed by the addition of dry toluene (1.0 mL). The mixture was stirred at rt for 36 h, and monitored by TLC. Product **4e** was obtained as a colorless oil, 32.4 mg, 85% yield; 77% ee, determined by HPLC analysis [Daicel Chiral IE Column, *i*PrOH/*n*-hexane = 40/60, 1.0 mL/min, λ = 254 nm, t (major) = 45.49 min, t (minor) = 23.49 min]; $[\alpha]_D^{25}$ = +10.9 (*c* = 0.17, $CHCl_3$); **^{1}H NMR** (400 MHz, $CDCl_3$): δ (ppm) 8.03–7.68 (m, 4H), 7.10 (s, 1H), 7.04 (t, *J* = 1.8, 1H), 6.74 (dd, *J* = 3.3, 1.3 Hz, 1H), 6.00 (s, 1H), 4.18–4.07(m, 2H), 3.91 (dd, *J* = 17.9, 1.3 Hz, 1H), 3.79 (dd, *J* = 13.8, 5.4 Hz, 1H), 3.42 (dd, *J* = 17.9, 13.8 Hz, 1H), 3.04 (dd, *J* = 17.9, 3.3 Hz, 1H), 2.88 (dd, *J* = 17.9, 5.4 Hz, 1H), 1.25 (t, *J* = 7.1 Hz, 3H); **^{13}C NMR** (150 MHz, $CDCl_3$): δ (ppm) 202.0, 201.3, 197.4, 165.7, 146.1, 143.2, 141.3, 141.2, 140.0, 136.0, 124.4, 123.3, 123.1, 122.2, 109.1, 60.7, 56.0, 41.6, 36.0, 32.1, 14.0; **HRMS** (ESI-TOF) m/z: $[M + Na]^+$ Calcd for $C_{22}H_{18}NaO_6^+$ 401.0996; Found 401.0996.

5.3 Diastereodivergent asymmetric [4+2] annulations involving 3-olefinic oxindoles

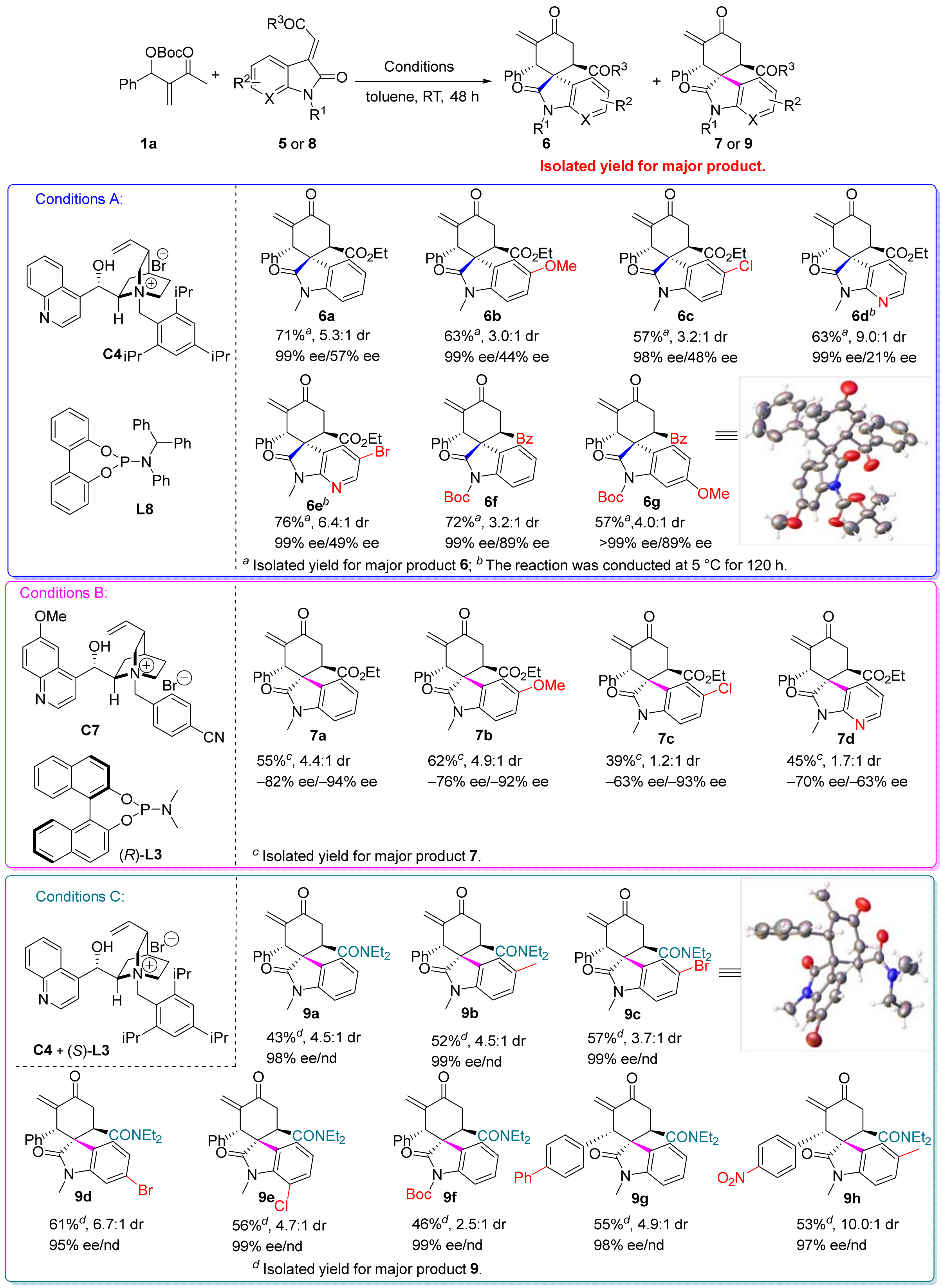

Conditions A: The reaction was conducted with MBH carbonate **1a** (0.12 mmol), 3-olefinic oxindole **5** (0.10 mmol), $Pd(OAc)_2$ (0.005 mmol), **C4** (0.0075 mmol) and **L8** (0.0075 mmol) in dry toluene (1.0 mL) under Ar, and the mixture was stirred at room temperature. After completion, the product was obtained by flash chromatography on silica gel (EtOAc/petroleum ether = 1/10–1/7). The racemates were obtained similarly by using the combination of achiral tetrabutylammonium bromide (TBAB) and triphenyl phosphite.

Conditions B: The reaction was conducted with MBH carbonate **1a** (0.12 mmol), 3-olefinic oxindole **5** (0.10 mmol), $Pd(OAc)_2$ (0.005 mmol), **C5** (0.0075 mmol) and (*R*)-**L3** (0.0075 mmol) in dry toluene (1.0 mL) under Ar, and the mixture was stirred at room temperature. After completion, the product was obtained by flash chromatography on silica gel (EtOAc/petroleum ether = 1/10–1/7). The racemates were obtained similarly by using the combination of achiral tetrabutylammonium bromide (TBAB) and triphenyl phosphite.

Conditions C: The reaction was conducted with MBH carbonate **1a** (0.12 mmol), (*E*)-N,N-diethyl-2-(1-methyl-2-oxoindolin-3-ylidene)acetamides **8** (0.10 mmol), $Pd(OAc)_2$ (0.005 mmol), **C4** (0.0075 mmol) and (*S*)-**L3** (0.0075 mmol) in dry toluene (1.0 mL) under Ar, and the mixture was stirred at room temperature. After completion, the product was obtained by flash chromatography on silica gel (EtOAc/petroleum ether = 1/5–1/3). The racemates were obtained similarly by using the combination of achiral tetrabutylammonium bromide (TBAB) and triphenyl phosphite.

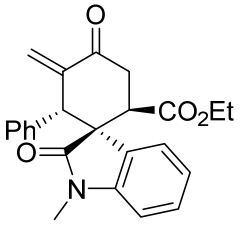

Ethyl (1*S*,2*R*,6*R*)-1'-methyl-3-methylene-2',4-dioxo-2-phenylspiro[cyclohexane-1,3'-indoline]-6-carboxylate (6a): Following **Conditions A**, a solution of *tert*-butyl (2-methylene-3-oxo-1-phenylbutyl)carbonate **1a** (41.4 mg, 0.150 mmol), ethyl (*E*)-2-(1-methyl-2-oxoindolin-3-ylidene)acetate **5a** (23.1 mg, 0.100 mmol), $Pd(OAc)_2$ (1.1 mg, 0.0050 mmol), **C4** (4.4 mg, 0.0075 mmol) and **L8** (3.5 mg, 0.0075 mmol) was stirred in dry toluene (1.0 mL) at room temperature under Ar. After completion, the major product was obtained as a colorless oil by flash chromatography on silica gel (EtOAc/petroleum ether = 1/10–1/7); 27.6 mg, 71% yield; 99% ee, determined by HPLC analysis [Daicel Chiral IE Column, *i*PrOH/*n*-hexane = 40/60, 1.0 mL/min, λ = 254 nm, t (major) = 19.64 min, t (minor) = 18.60 min]; $[\alpha]^{25}_{D}$ = +38.9 (*c* = 0.45, $CHCl_3$); **^{1}H NMR** (400 MHz, $CDCl_3$): *δ* (ppm) 7.29–7.01 (m, 4H), 6.85–6.60 (m, 4H), 6.28 (t, *J* = 1.4 Hz, 1H), 6.05 (d, *J* = 7.4 Hz, 1H), 5.18 (d, *J* = 1.4 Hz, 1H), 4.06 (s, 1H), 3.89–3.75 (m, 2H), 3.48 (dd, *J* = 11.0, 5.8 Hz, 1H), 3.39 (dd, *J* = 17.4, 11.0 Hz, 1H), 3.09 (s, 3H), 2.96 (dd, *J* = 17.4, 5.8 Hz, 1H), 0.87 (t, *J* = 7.1 Hz, 3H); **^{13}C NMR** (100 MHz, $CDCl_3$): *δ* (ppm) 197.4, 177.4, 171.5, 144.2, 143.9, 139.1, 130.5, 129.2, 128.6, 128.3, 127.9, 126.6, 125.7, 121.9, 108.3, 61.4, 53.1, 51.9, 44.6, 38.2, 26.5, 14.1; **HRMS** (ESI-TOF) m/z: $[M + Na]^+$ Calcd for $C_{24}H_{23}O_4NNa^+$ 412.1519; Found 412.1526.

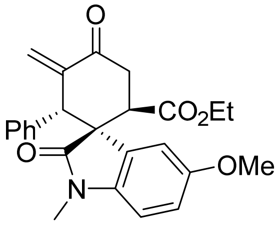

Ethyl (1*S*,2*R*,6*R*)-5'-methoxy-1'-methyl-3-methylene-2',4-dioxo-2-phenylspiro[cyclohexane-1,3'-indoline]-6-carboxylate (6b): Following **Conditions A**, *tert*-butyl (2-methylene-3-oxo-1-phenylbutyl)carbonate **1a** (41.4 mg, 0.150 mmol), ethyl (*E*)-2-(5-methoxy-1-methyl-2-oxoindolin-3-ylidene)acetate **5b** (26.1 mg, 0.100 mmol), $Pd(OAc)_2$ (1.1 mg, 0.0050 mmol), **C4** (4.4 mg, 0.0075 mmol) and **L8** (3.5 mg, 0.0075 mmol) was stirred in dry toluene (1.0 mL) at room temperature under Ar. After completion, the major product was obtained as a colorless oil by flash chromatography on silica gel (EtOAc/petroleum ether = 1/10–1/7); 26.4 mg, 63% yield; 99% ee, determined by HPLC analysis [Daicel Chiral IE Column, *i*PrOH/*n*-hexane = 40/60, 1.0 mL/min, λ = 254 nm, t (major) = 22.32 min, t (minor) = 20.15 min]; $[\alpha]^{25}_{D}$ = +34.8 (*c* = 0.47, $CHCl_3$); **^{1}H NMR** (400 MHz, $CDCl_3$): *δ* (ppm) 7.26–7.17 (m, 3H), 6.84–6.73 (m, 3H), 6.69 (d, *J* = 8.4 Hz, 1H), 6.34 (s, 1H), 5.65 (d, *J* = 2.5 Hz, 1H), 5.25 (s, 1H), 4.08 (s, 1H), 3.93–3.83 (m, 2H), 3.60–3.42 (m, 2H), 3.52 (s, 3H), 3.14 (s, 3H), 3.01 (dd, *J* = 16.6, 5.0 Hz, 1H), 0.94 (t, *J* = 7.1 Hz, 3H); **^{13}C NMR** (100 MHz, $CDCl_3$): *δ* (ppm) 197.0,

176.8, 171.0, 154.8, 143.4, 139.0, 137.4, 130.3, 129.4, 128.1, 127.6, 126.3, 113.9, 112.3, 108.3, 61.0, 55.6, 52.9, 51.7, 44.2, 37.8, 26.3, 13.8; **HRMS** (ESI-TOF) m/z: $[M + Na]^+$ Calcd for $C_{25}H_{25}O_5NNa^+$ 442.1625; Found 442.1623.

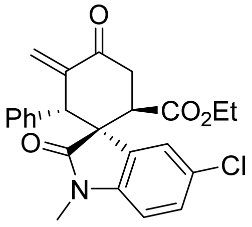

Ethyl (1*S*,2*R*,6*R*)-5'-chloro-1'-methyl-3-methylene-2',4-dioxo-2-phenyl spiro[cyclohexane-1,3'-indoline]-6-carboxylate (6c): Following **Conditions A**, *tert*-butyl (2-methylene-3-oxo-1-phenylbutyl)carbonate **1a** (41.4 mg, 0.150 mmol), ethyl (*E*)-2-(5-chloro-1-methyl-2-oxoindolin-3-ylidene) acetate **5c** (26.6 mg, 0.100 mmol), $Pd(OAc)_2$ (1.1 mg, 0.0050 mmol), **C4** (4.4 mg, 0.0075 mmol) and **L8** (3.5 mg, 0.0075 mmol) was stirred in dry toluene (1.0 mL) at room temperature under Ar. After completion, the major product was obtained as a colorless oil by flash chromatography on silica gel (EtOAc/petroleum ether = 1/10–1/7); 24.2 mg, 57% yield; 98% ee, determined by HPLC analysis [Daicel Chiral IE Column, *i*PrOH/*n*-hexane = 40/60, 1.0 mL/min, λ = 254 nm, t (major) = 16.93 min, t (minor) = 13.06 min]; $[\alpha]_D^{25}$ = +101.7 (*c* = 0.23, $CHCl_3$); **^{1}H NMR** (400 MHz, $CDCl_3$): *δ* (ppm) 7.35–7.13 (m, 4H), 6.82–6.63 (m, 3H), 6.36 (t, *J* = 1.2 Hz, 1H), 5.91 (d, *J* = 2.0 Hz, 1H), 5.27 (t, *J* = 1.2 Hz, 1H), 4.03 (s, 1H), 3.94–3.85 (m, 2H), 3.57–3.43 (m, 2H), 3.16 (s, 3H), 3.02 (dd, *J* = 17.0, 5.4 Hz, 1H), 0.96 (t, *J* = 7.1 Hz, 3H); **^{13}C NMR** (150 MHz, $CDCl_3$): *δ* (ppm) 196.5, 176.7, 170.8, 142.8, 142.4, 138.5, 129.9, 128.5, 128.1, 128.0, 127.9, 126.8, 126.6, 125.7, 108.7, 61.1, 52.8, 51.4, 43.8, 37.6, 26.2, 13.7; **HRMS** (ESI-TOF) m/z: $[M + Na]^+$ Calcd for $C_{24}H_{22}O_4NClNa^+$ 446.1130 (^{35}Cl) and 448.1100 (^{37}Cl); Found 446.1126 (^{35}Cl) and 448.1102 (^{37}Cl).

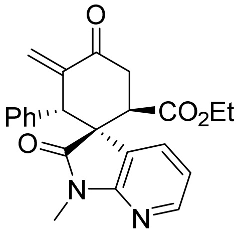

Ethyl (1*S*,2*R*,6*R*)-1'-methyl-3-methylene-2',4-dioxo-2-phenyl-1',2'-dihydro spiro[cyclohexane-1,3'-pyrrolo[2,3-*b*]pyridine]-6-carboxylate (6d): Following **Conditions A**, *tert*-butyl (2-methylene-3-oxo-1-phenylbutyl) carbonate **1a** (41.4 mg, 0.150 mmol), ethyl (*E*)-2-(1-methyl-2-oxo-1,2-dihydro-3*H*-pyrrolo[2,3-*b*]pyridin-3-ylidene)acetate **5d** (23.2 mg, 0.100 mmol), $Pd(OAc)_2$ (1.1 mg, 0.0050 mmol), **C4** (4.4 mg, 0.0075 mmol) and **L8** (3.5 mg, 0.0075 mmol) was stirred in dry toluene (1.0 mL) at 5 °C under Ar. After completion, the major product was obtained as a white solid by flash chromatography on silica gel (EtOAc/petroleum ether = 1/10–1/7); 24.6 mg, 63% yield; mp: 151–153

°C; 99% ee, determined by HPLC analysis [Daicel Chiral IE Column, *i*PrOH/*n*-hexane = 40/60, 1.0 mL/min, λ = 254 nm, t (major) = 17.02 min, t (minor) = 15.32 min]; $[\alpha]_D^{25}$ = +33.6 (*c* = 0.22, $CHCl_3$); **^{1}H NMR** (400 MHz, $CDCl_3$): *δ* (ppm) 8.16 (dd, *J* = 5.3, 1.6 Hz, 1H), 7.31–7.23 (m, 3H), 6.81–6.75 (m, 2H), 6.70 (dd, *J* = 7.4, 5.3 Hz, 2H), 6.36 (s, 1H), 6.22 (dd, *J* = 7.4, 1.6 Hz, 1H), 5.29 (s, 1H), 4.07 (s, 1H), 3.98–3.78 (m, 2H), 3.60 (dd, *J* = 11.8, 6.0 Hz, 1H), 3.48 (dd, *J* = 17.7, 11.8 Hz, 1H), 3.27 (s, 3H), 3.02 (dd, *J* = 17.7, 6.0 Hz, 1H), 0.95 (t, *J* = 7.1 Hz, 3H); **^{13}C NMR** (100 MHz, $CDCl_3$): *δ* (ppm) 196.4, 176.9, 170.7, 157.1, 147.7, 142.8, 138.9, 132.7, 130.1, 128.3, 128.0, 126.9, 123.1, 117.1, 61.3, 52.5, 51.0, 43.9, 37.8, 25.4, 13.8; **HRMS** (ESI-TOF) m/z: $[M + Na]^+$ Calcd for $C_{23}H_{22}O_4N_2Na^+$ 413.1472; Found 413.1466.

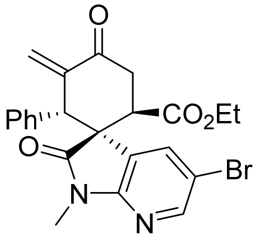

Ethyl (1*S*,2*R*,6*R*)-5'-bromo-1'-methyl-3-methylene-2',4-dioxo-2-phenyl-1',2'-dihydrospiro[cyclohexane-1,3'-pyrrolo[2,3-*b*]pyridine]-6-carboxylate (6e): Following **Conditions A**, *tert*-butyl (2-methylene-3-oxo-1-phenylbutyl)carbonate **1a** (41.4 mg, 0.15 mmol), ethyl (*E*)-2-(5-bromo-1-methyl-2-oxo-1,2-dihydro-3*H*-pyrrolo[2,3-*b*]pyridin-3-ylidene)acetate **5e** (31.2 mg, 0.10 mmol), $Pd(OAc)_2$ (1.1 mg, 0.005 mmol), **C4** (4.4 mg, 0.0075 mmol) and **L8** (3.5 mg, 0.0075 mmol) was stirred in dry toluene (1.0 mL) at 5 °C under Ar. After completion, the major product was obtained as a semi-solid by flash chromatography on silica gel (EtOAc/petroleum ether = 1/10–1/7); 35.7 mg, 76% yield; 99% ee, determined by HPLC analysis [Daicel Chiral IE Column, *i*PrOH/*n*-hexane = 40/60, 1.0 mL/min, λ = 254 nm, t (major) = 15.31 min, t (minor) = 10.94 min]; $[\alpha]_D^{25}$ = +90.0 (*c* = 0.16, $CHCl_3$); **^{1}H NMR** (400 MHz, $CDCl_3$): *δ* (ppm) 8.21 (d, *J* = 2.1 Hz, 1H), 7.36–7.28 (m, 3H), 6.83–6.69 (m, 2H), 6.37 (s, 1H), 6.14 (d, *J* = 2.1 Hz, 1H), 5.32 (d, *J* = 1.3 Hz, 1H), 3.99 (s, 1H), 3.98–3.84 (m, 2H), 3.58 (dd, *J* = 12.2, 5.8 Hz, 1H), 3.47 (dd, *J* = 17.5, 12.2 Hz, 1H), 3.26 (s, 3H), 3.03 (dd, *J* = 17.5, 5.8 Hz, 1H), 0.98 (t, *J* = 7.1 Hz, 3H); **^{13}C NMR** (100 MHz, $CDCl_3$): *δ* (ppm) 196.0, 176.4, 170.5, 155.8, 148.2, 142.3, 138.7, 135.4, 130.0, 128.4, 128.3, 127.2, 124.9, 112.4, 61.4, 52.7, 51.2, 43.6, 37.7, 25.5, 13.8; **HRMS** (ESI-TOF) m/z: $[M + Na]^+$ Calcd for $C_{23}H_{21}O_4N_2BrNa^+$ 491.0577 (^{79}Br) and 493.0556 (^{81}Br); Found 491.0577 (^{79}Br) and 493.0561 (^{81}Br).

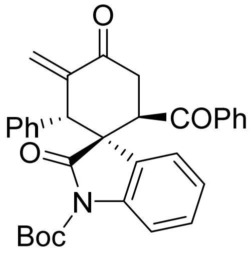

tert-Butyl (1_S_,2_R_,6_R_)-6-benzoyl-3-methylene-2',4-dioxo-2-phenylspiro [cyclohexane-1,3'-indoline]-1'-carboxylate (6f): Following **Conditions A**, _tert_-butyl (2-methylene-3-oxo-1-phenylbutyl)carbonate **1a** (41.4 mg, 0.150 mmol), _tert_-butyl (_E_)-2-oxo-3-(2-oxo-2-phenylethylidene)indoline-1-carboxylate **5f** (34.9 mg, 0.100 mmol), $Pd(OAc)_2$ (1.1 mg, 0.0050 mmol), **C4** (4.4 mg, 0.0075 mmol) and **L8** (3.5 mg, 0.0075 mmol) was stirred in dry toluene (1.0 mL) at room temperature under Ar. After completion, the major product was obtained as a white solid by flash chromatography on silica gel (EtOAc/petroleum ether = 1/10–1/7); 36.5 mg, 72% yield; mp: 154–156 °C; 99% ee, determined by HPLC analysis [Daicel Chiral ID Column, _i_PrOH/_n_-hexane = 40/60, 1.0 mL/min, λ = 254 nm, t (major) = 9.96 min, t (minor) = 8.24 min]; $[\alpha]_D^{25}$ = +49.3 (_c_ = 0.60, $CHCl_3$); **^{1}H NMR** (400 MHz, $CDCl_3$): _δ_ (ppm) 7.82 (d, _J_ = 8.4 Hz, 1H), 7.67 (d, _J_ = 8.0 Hz, 2H), 7.52 (t, _J_ = 7.6 Hz, 1H), 7.38 (t, _J_ = 7.6 Hz, 2H), 7.32–7.27 (m, 3H), 7.18 (t, _J_ = 8.0 Hz, 1H), 6.81 (d, _J_ = 6.8 Hz, 2H), 6.67 (t, _J_ = 7.6 Hz, 1H), 6.33 (s, 1H), 5.75 (d, _J_ = 7.6 Hz, 1H), 5.33 (s, 1H), 4.73 (dd, _J_ = 12.7, 6.1 Hz, 1H), 4.07 (s, 1H), 3.44 (dd, _J_ = 17.9, 12.7 Hz, 1H), 3.03 (dd, _J_ = 17.9, 6.1 Hz, 1H), 1.67 (s, 9H); **^{13}C NMR** (100 MHz, $CDCl_3$): _δ_ (ppm) 197.4, 197.1, 176.0, 149.4, 143.0, 140.0, 139.2, 135.2, 133.8, 130.2, 128.9, 128.7, 128.30, 128.26, 127.8, 127.7, 127.1, 124.2, 123.1, 114.9, 84.4, 54.0, 51.2, 46.3, 38.7, 28.2; **HRMS** (ESI-TOF) m/z: $[M + Na]^+$ Calcd for $C_{32}H_{29}O_5NNa^+$ 530.1938; Found 530.1940.

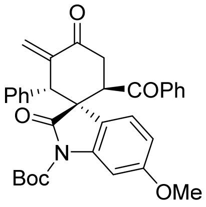

tert-Butyl (1_S_,2_R_,6_R_)-6-benzoyl-6'-methoxy-3-methylene-2',4-dioxo-2-phenylspiro[cyclohexane-1,3'-indoline]-1'-carboxylate (6g): Following **Conditions A**, _tert_-butyl (2-methylene-3-oxo-1-phenylbutyl)carbonate **1a** (41.4 mg, 0.150 mmol), _tert_-butyl (_E_)-6-methoxy-2-oxo-3-(2-oxo-2-phenylethylidene) indoline-1-carboxylate **5g** (37.9 mg, 0.100 mmol), $Pd(OAc)_2$ (1.1 mg, 0.0050 mmol), **C4** (4.4 mg, 0.0075 mmol) and **L8** (3.5 mg, 0.0075 mmol) was stirred in dry toluene (1.0 mL) at room temperature under Ar. After completion, the major product was obtained as a white solid by flash chromatography on silica gel (EtOAc/petroleum ether = 1/10–1/7); 30.5 mg, 57% yield; mp: 183–184 °C; >99% ee, determined by HPLC analysis [Daicel Chiral IF Column, 1.0 mL/min, λ = 254 nm, eluent (20% V/V isopropanol dissolved in _n_-hexane), t (major) = 13.35 min, t (minor) = 10.10 min]; $[\alpha]_D^{25}$ = +49.5 (_c_ = 0.32, $CHCl_3$); **^{1}H NMR** (400 MHz, $CDCl_3$): _δ_ (ppm) 7.72–7.63 (m, 2H), 7.56–7.49 (m, 1H), 7.47 (d, _J_ = 2.4 Hz, 1H), 7.38 (t, _J_ = 7.8 Hz, 2H), 7.31–7.28 (m, 3H), 6.86–6.80 (m, 2H), 6.32 (t, _J_ = 1.1 Hz,

1H), 6.20 (dd, J = 8.4, 2.4 Hz, 1H), 5.61 (d, J = 8.4 Hz, 1H), 5.32 (t, J = 1.1 Hz, 1H), 4.69 (dd, J = 12.8, 6.0 Hz, 1H), 4.04 (s, 1H), 3.74 (s, 3H), 3.41 (dd, J = 17.8, 12.8 Hz, 1H), 3.00 (dd, J = 17.8, 6.0 Hz, 1H), 1.67 (s, 9H); **^{13}C NMR** (100 MHz, $CDCl_3$): δ (ppm) 197.6, 197.2, 176.3, 160.0, 149.3, 143.2, 141.1, 139.4, 135.2, 133.8, 130.2, 128.9, 128.3, 128.2, 127.8, 126.9, 124.9, 119.4, 108.9, 101.4, 84.4, 55.4, 54.1, 50.8, 46.3, 38.8, 28.2; **HRMS** (ESI-TOF) m/z: $[M + Na]^+$ Calcd for $C_{32}H_{31}O_6NNa^+$ 560.2044; Found 560.2042.

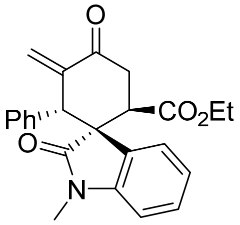

Ethyl (1*R*,2*R*,6*R*)-1'-methyl-3-methylene-2',4-dioxo-2-phenylspiro[cyclohexane-1,3'-indoline]-6-carboxylate (7a): Following **Conditions B**, *tert*-butyl (2-methylene-3-oxo-1-phenylbutyl)carbonate **1a** (41.4 mg, 0.150 mmol), ethyl (*E*)-2-(1-methyl-2-oxoindolin-3-ylidene)acetate **5a** (23.1 mg, 0.100 mmol), $Pd(OAc)_2$ (1.1 mg, 0.0050 mmol), **C7** (3.7 mg, 0.0075 mmol) and (*R*)-**L3** (2.7 mg, 0.0075 mmol) was stirred in dry toluene (1.0 mL) at room temperature under Ar. After completion, the major product was obtained as a colorless oil by flash chromatography on silica gel (EtOAc/petroleum ether = 1/10–1/7); 21.4 mg, 55% yield; −82% ee, determined by HPLC analysis [Daicel Chiral IE Column, *i*PrOH/*n*-hexane = 40/60, 1.0 mL/min, λ = 254 nm, t (major) = 12.09 min, t (minor) = 15.92 min]; $[\alpha]^{25}_{D}$ = +38.0 (c = 0.20, $CHCl_3$); **^{1}H NMR** (400 MHz, $CDCl_3$): δ (ppm) 7.19–7.13 (m, 2H), 7.07–6.96 (m, 4H), 6.94–6.89 (m, 2H), 6.52 (d, J = 7.8 Hz, 1H), 6.35 (dd, J = 2.7, 1.2 Hz, 1H), 5.10 (dd, J = 2.7, 1.2 Hz, 1H), 4.71 (t, J = 2.7 Hz, 1H), 4.15–4.02 (m, 2H), 3.64 (dd, J = 17.2, 6.4 Hz, 1H), 3.2 (dd, J = 6.4, 5.2 Hz, 1H) 2.97 (s, 3H), 2.86 (dd, J = 17.2, 5.2 Hz, 1H), 1.09 (t, J = 7.2 Hz, 3H); **^{13}C NMR** (100 MHz, $CDCl_3$): δ (ppm) 197.4, 176.6, 172.3, 143.8, 143.1, 136.1, 130.1, 129.5, 128.7, 127.6, 127.3, 124.8, 123.7, 122.3, 108.0, 61.2, 52.6, 51.8, 44.9, 37.6, 25.9, 13.9; **HRMS** (ESI-TOF) m/z: $[M + Na]^+$ Calcd for $C_{24}H_{23}O_4NNa^+$ 412.1519; Found 412.1518.

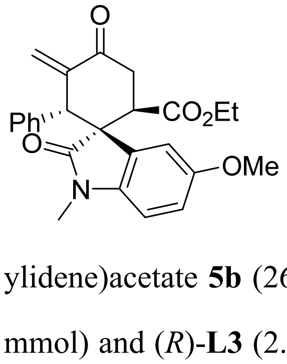

Ethyl (1*R*,2*R*,6*R*)-5'-methoxy-1'-methyl-3-methylene-2',4-dioxo-2-phenylspiro[cyclohexane-1,3'-indoline]-6-carboxylate (7b): Following **Conditions B**, *tert*-butyl (2-methylene-3-oxo-1-phenylbutyl)carbonate **1a** (41.4 mg, 0.150 mmol), ethyl (*E*)-2-(5-methoxy-1-methyl-2-oxoindolin-3-ylidene)acetate **5b** (26.1 mg, 0.100 mmol), $Pd(OAc)_2$ (1.1 mg, 0.0050 mmol), **C7** (3.7 mg, 0.0075 mmol) and (*R*)-**L3** (2.7 mg, 0.0075 mmol) was stirred in dry toluene (1.0 mL) at room temperature

under Ar. After completion, the major product was obtained as a colorless oil by flash chromatography on silica gel (EtOAc/petroleum ether = 1/10–1/7); 26.0 mg, 62% yield; −76% ee, determined by HPLC analysis [Daicel Chiral IE Column, *i*PrOH/*n*-hexane = 40/60, 1.0 mL/min, λ = 254 nm, t (major) = 13.09 min, t (minor) = 18.04 min]; $[\alpha]_D^{25}$ = +91.4 (*c* = 0.30, $CHCl_3$); **^{1}H NMR** (400 MHz, $CDCl_3$): *δ* (ppm) 7.06–7.01 (m, 3H), 6.97–6.89 (m, 2H), 6.80 (d, *J* = 2.5 Hz, 1H), 6.67 (dd, *J* = 8.5, 2.5 Hz, 1H), 6.42 (d, *J* = 8.5 Hz, 1H), 6.33 (dd, *J* = 2.8, 1.4 Hz, 1H), 5.07 (dd, *J* = 2.8, 1.4 Hz, 1H), 4.70 (t, *J* = 2.8 Hz, 1H), 4.17–4.05 (m, 2H), 3.75 (s, 3H), 3.67 (dd, *J* = 17.2, 6.5 Hz, 1H), 3.19 (dd, *J* = 6.5, 4.8 Hz, 1H), 2.94 (s, 3H), 2.82 (dd, *J* = 17.2, 4.8 Hz, 1H), 1.12 (t, *J* = 7.1 Hz, 3H); **^{13}C NMR** (100 MHz, $CDCl_3$): *δ* (ppm) 197.4, 176.2, 172.4, 155.7, 144.0, 136.6, 136.2, 130.7, 130.2, 127.7, 127.3, 124.8, 112.7, 111.7, 108.2, 61.3, 55.9, 52.8, 51.7, 45.0, 37.6, 26.0, 13.9; **HRMS** (ESI-TOF) m/z: $[M + Na]^+$ Calcd for $C_{25}H_{25}O_5NNa^+$ 442.1625; Found 442.1622.

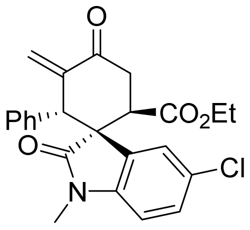

Ethyl (1*R*,2*R*,6*R*)-5'-chloro-1'-methyl-3-methylene-2',4-dioxo-2-phenylspiro[cyclohexane-1,3'-indoline]-6-carboxylate (7c): Following **Conditions B**, *tert*-butyl (2-methylene-3-oxo-1-phenylbutyl)carbonate **1a** (41.4 mg, 0.150 mmol), ethyl (*E*)-2-(5-chloro-1-methyl-2-oxoindolin-3-ylidene)acetate **5c** (26.6 mg, 0.100 mmol), $Pd(OAc)_2$ (1.1 mg, 0.0050 mmol), **C7** (3.7 mg, 0.0075 mmol) and (*R*)-**L3** (2.7 mg, 0.0075 mmol) was stirred in dry toluene (1.0 mL) at room temperature under Ar. After completion, the major product was obtained as a colorless oil by flash chromatography on silica gel (EtOAc/petroleum ether = 1/10–1/7); 16.5 mg, 39% yield; −63% ee, determined by HPLC analysis analysis [Daicel Chiral IE Column, *i*PrOH/*n*-hexane = 40/60, 1.0 mL/min, λ = 254 nm, t (major) = 9.26 min, t (minor) = 10.60 min]; $[\alpha]_D^{25}$ = +49.0 (*c* = 0.20, $CHCl_3$); **^{1}H NMR** (400 MHz, $CDCl_3$): *δ* (ppm) 7.17–7.11 (m, 2H), 7.08–7.05 (m, 3H), 6.95–6.92 (m, 2H), 6.45 (d, *J* = 8.2 Hz, 1H), 6.36–6.35 (m, 1H), 5.09–5.08 (m, 1H), 4.70 (t, *J* = 2.8 Hz, 1H), 4.29–4.04 (m, 2H), 3.64 (ddd, *J* = 17.2, 6.5, 1.0 Hz, 1H), 3.17 (dd, *J* = 6.5, 4.6 Hz, 1H), 2.96 (s, 3H), 2.82 (dd, *J* = 17.2, 4.6 Hz, 1H), 1.16 (t, *J* = 7.1 Hz, 3H); **^{13}C NMR** (100 MHz, $CDCl_3$): *δ* (ppm) 196.9, 176.1, 172.3, 143.6, 141.7, 135.9, 131.1, 130.1, 128.6, 127.9, 127.7, 127.5, 125.1, 124.3, 108.9, 61.5, 52.7, 51.5, 44.8, 37.4, 26.1, 13.9; **HRMS** (ESI-TOF) m/z: $[M + Na]^+$ Calcd for $C_{24}H_{22}O_4NClNa^+$ 446.1130 (^{35}Cl) and 448.1100 (^{37}Cl); Found 446.1129 (^{35}Cl) and 448.1109 (^{37}Cl).

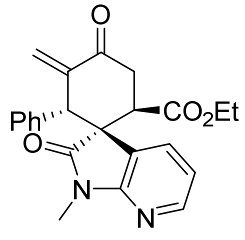

Ethyl (1*R*,2*R*,6*R*)-1'-methyl-3-methylene-2',4-dioxo-2-phenyl-1',2'-dihydrospiro[cyclohexane-1,3'-pyrrolo[2,3-b]pyridine]-6-carboxylate (7d): Following **Conditions B**, *tert*-butyl (2-methylene-3-oxo-1-phenylbutyl) carbonate **1a** (41.4 mg, 0.150 mmol), ethyl (*E*)-2-(1-methyl-2-oxo-1,2-dihydro-3*H*-pyrrolo[2,3-b]pyridin-3-ylidene)acetate **5d** (23.2 mg, 0.100 mmol), $Pd(OAc)_2$ (1.1 mg, 0.0050 mmol), **C7** (3.7 mg, 0.0075 mmol) and (*R*)-**L3** (2.7 mg, 0.0075 mmol) was stirred in dry toluene (1.0 mL) at room temperature under Ar. After completion, the major product was obtained as a colorless oil by flash chromatography on silica gel (EtOAc/petroleum ether = 1/10–1/7); 17.6 mg, 45% yield; −70% ee, determined by HPLC analysis [Daicel Chiral IE Column, *i*PrOH/*n*-hexane = 40/60, 1.0 mL/min, λ = 254 nm, t (major) = 10.86 min, t (minor) = 12.00 min]; $[\alpha]_D^{25}$ = +29.3 (*c* = 0.08, $CHCl_3$); **^{1}H NMR** (400 MHz, $CDCl_3$): *δ* (ppm) 8.05 (dd, *J* = 5.2, 1.6 Hz, 1H), 7.42 (dd, *J* = 7.4, 1.6 Hz, 1H), 7.16–7.02 (m, 3H), 6.99–6.82 (m, 3H), 6.36 (dd, *J* = 2.8, 1.2 Hz, 1H), 5.11 (dd, *J* = 2.8, 1.2 Hz, 1H), 4.71 (t, *J* = 2.8 Hz, 1H), 4.16–4.04 (m, 2H), 3.61 (dd, *J* = 17.4, 6.6 Hz, 1H), 3.24 (dd, *J* = 6.6, 5.2 Hz, 1H), 3.07 (s, 3H), 2.87 (dd, *J* = 17.4, 5.2 Hz, 1H), 1.12 (t, *J* = 7.2 Hz, 3H); **^{13}C NMR** (100 MHz, $CDCl_3$): *δ* (ppm) 196.9, 176.3, 172.1, 156.4, 147.6, 143.4, 135.7, 131.4, 130.0, 128.0, 127.8, 125.3, 124.2, 117.8, 61.4, 52.3, 51.5, 44.4, 37.6, 25.2, 13.9; **HRMS** (ESI-TOF) m/z: $[M + Na]^+$ Calcd for $C_{23}H_{22}O_4N_2Na^+$ 413.1472; Found 413.1471.

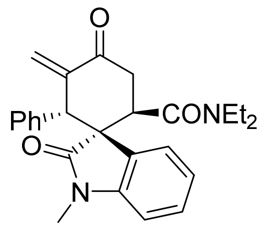

(1*R*,2*R*,6*R*)-N,N-Diethyl-1'-methyl-3-methylene-2',4-dioxo-2-phenylspiro [cyclohexane-1,3'-indoline]-6-carboxamide (9a): Following **Conditions C**, *tert*-butyl (2-methylene-3-oxo-1-phenylbutyl) carbonate **1a** (41.4 mg, 0.150 mmol), (*E*)-N,N-diethyl-2-(1-methyl-2-oxoindolin-3-ylidene)acetamide **8a** (25.8 mg, 0.100 mmol), $Pd(OAc)_2$ (1.1 mg, 0.0050 mmol), **C4** (4.4 mg, 0.0075 mmol) and (*S*)-**L3** (2.7 mg, 0.0075 mmol) was stirred in dry toluene (1.0 mL) at room temperature under Ar. After completion, the major product was obtained as a colorless oil by flash chromatography on silica gel (EtOAc/petroleum ether = 1/5–1/3); 17.9 mg, 43% yield; 98% ee, determined by HPLC analysis [Daicel Chiral AD-H Column, *i*PrOH/*n*-hexane = 20/80, 1.0 mL/min, λ = 254 nm, t (major) = 9.69 min, t (minor) = 12.21 min]; $[\alpha]_D^{25}$ = −65.6 (*c* = 0.18, $CHCl_3$); **^{1}H NMR** (400 MHz, $CDCl_3$): *δ* (ppm) 7.24 (d, *J* = 1.2 Hz, 1H), 7.12 (td, *J* = 7.8, 1.2 Hz, 1H), 7.02–6.89 (m, 6H), 6.51 (d, *J* = 7.8 Hz, 1H), 6.27 (dd, *J* = 3.0, 1.5 Hz, 1H), 5.09 (t, *J* = 3.0 Hz, 1H), 4.94 (dd, *J* = 3.0, 1.5 Hz, 1H), 3.79 (dd, *J* =

16.6, 6.5 Hz, 1H), 3.64 (dq, J = 14.1, 7.1 Hz, 1H), 3.24 (dd, J = 6.5, 2.2 Hz, 1H), 3.21–2.14 (m, 1H), 2.98 (s, 3H), 2.77–2.64 (m, 2H), 2.59 (dd, J = 16.6, 2.2 Hz, 1H), 1.19 (t, J = 7.1 Hz, 3H), 0.80 (t, J = 7.1 Hz, 3H); **^{13}C NMR** (150 MHz, $CDCl_3$): δ (ppm) 197.9, 177.1, 171.4, 145.0, 142.7, 136.9, 130.3, 129.1, 128.6, 127.4, 126.9, 124.5, 123.3, 122.0, 107.7, 52.8, 51.1, 42.2, 41.0, 40.9, 38.2, 25.8, 14.1, 12.7; **HRMS** (ESI-TOF) m/z: $[M + Na]^+$ Calcd for $C_{26}H_{28}O_3N_2Na^+$ 439.1992; Found 439.1993.

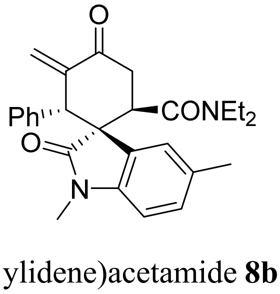

(1*R*,2*R*,6*R*)-N,N-Diethyl-1',5'-dimethyl-3-methylene-2',4-dioxo-2-phenyl spiro[cyclohexane-1,3'-indoline]-6-carboxamide (9b): Following **Conditions C**, *tert*-butyl (2-methylene-3-oxo-1-phenylbutyl)carbonate **1a** (41.4 mg, 0.150 mmol), (*E*)-N,N-diethyl-2-(1,5-dimethyl-2-oxoindolin-3-ylidene)acetamide **8b** (27.2 mg, 0.100 mmol), $Pd(OAc)_2$ (1.1 mg, 0.0050 mmol), **C4** (4.4 mg, 0.0075 mmol) and (*S*)-**L3** (2.7 mg, 0.0075 mmol) was stirred in dry toluene (1.0 mL) at room temperature under Ar. After completion, the major product was obtained as a colorless oil by flash chromatography on silica gel (EtOAc/petroleum ether = 1/5–1/3); 22.4 mg, 52% yield; 99% ee, determined by HPLC analysis [Daicel Chiral AD-H Column, *i*PrOH/*n*-hexane = 10/90, 1.0 mL/min, λ = 254 nm, t (major) = 15.19 min, t (minor) = 21.63 min]; $[\alpha]_D^{25}$ = –96.3 (c = 0.43, $CHCl_3$); **^{1}H NMR** (400 MHz, $CDCl_3$): δ (ppm) 7.07 (d, J = 1.7 Hz, 1H), 7.02–6.97 (m, 3H), 6.95–6.85 (m, 3H), 6.39 (d, J = 7.9 Hz, 1H), 6.27 (dd, J = 3.0, 1.5 Hz, 1H), 5.05 (t, J = 3.0 Hz, 1H), 4.93 (dd, J = 3.0, 1.5 Hz, 1H), 3.81 (dd, J = 16.6, 6.5 Hz, 1H), 3.77–3.69 (m, 1H), 3.23 (dd, J = 6.5, 2.1 Hz, 1H), 3.09 (dq, J = 14.0, 7.1 Hz, 1H), 2.96 (s, 3H), 2.75–2.64 (m, 2H), 2.57 (dd, J = 16.6, 2.1 Hz, 1H), 2.26 (s, 3H), 1.22 (t, J = 7.1 Hz, 3H), 0.81 (t, J = 7.1 Hz, 3H); **^{13}C NMR** (100 MHz, $CDCl_3$): δ (ppm) 198.1, 177.1, 171.7, 145.3, 140.4, 137.1, 131.6, 130.4, 129.2, 128.9, 127.5, 126.9, 125.2, 123.3, 107.6, 52.9, 51.2, 42.2, 41.2, 40.9, 38.4, 25.9, 21.1, 14.2, 13.0; **HRMS** (ESI-TOF) m/z: $[M + Na]^+$ Calcd for $C_{27}H_{30}O_3N_2Na^+$ 453.2149; Found 453.2150.

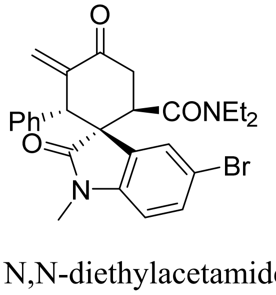

(1*R*,2*R*,6*R*)-5'-Bromo-N,N-diethyl-1'-methyl-3-methylene-2',4-dioxo-2-phenylspiro[cyclohexane-1,3'-indoline]-6-carboxamide (9c): Following **Conditions C**, *tert*-butyl (2-methylene-3-oxo-1-phenylbutyl)carbonate **1a** (41.4 mg, 0.150 mmol), (*E*)-2-(5-bromo-1-methyl-2-oxoindolin-3-ylidene)-N,N-diethylacetamide **8c** (33.7 mg, 0.100 mmol), $Pd(OAc)_2$ (1.1 mg, 0.0050 mmol), **C4** (4.4 mg,

0.0075 mmol) and (*S*)-**L3** (2.7 mg, 0.0075 mmol) was stirred in dry toluene (1.0 mL) at room temperature under Ar. After completion, the product was obtained as white solid by flash chromatography on silica gel (EtOAc/petroleum ether = 1/5–1/3); 28.3 mg, 57% yield; mp: 214–216 °C; 99% ee, determined by HPLC analysis [Daicel Chiral IE Column, *i*PrOH/*n*-hexane = 40/60, 1.0 mL/min, λ = 254 nm, t (major) = 14.35 min, t (minor) = 13.14 min]; $[\alpha]_D^{25}$ = −74.7 (*c* = 0.16, $CHCl_3$); ^{1}**H NMR** (400 MHz, $CDCl_3$): *δ* (ppm) 7.15–7.06 (m, 2H), 7.06–6.98 (m, 3H), 6.95–6.90 (m, 2H), 6.66 (d, *J* = 2.4 Hz, 1H), 6.28 (dd, *J* = 3.2, 1.6 Hz, 1H), 5.08 (t, *J* = 2.8 Hz, 1H), 4.94 (dd, *J* = 2.8, 1.6 Hz, 1H), 3.75 (dd, *J* = 16.7, 6.6 Hz, 1H), 3.63 (dq, *J* = 14.1, 7.1 Hz, 1H), 3.30–3.09 (m, 2H), 2.96 (s, 3H), 2.83–2.66 (m, 2H), 2.59 (dd, *J* = 16.7, 2.2 Hz, 1H), 1.19 (t, *J* = 7.1 Hz, 3H), 0.85 (t, *J* = 7.1 Hz, 3H); ^{13}**C NMR** (100 MHz, $CDCl_3$): *δ* (ppm) 197.6, 177.0, 171.3, 144.8, 144.2, 136.7, 130.3, 128.3, 127.7, 127.3, 125.9, 124.9, 123.7, 122.3, 111.4, 52.8, 51.2, 42.4, 41.1, 40.8, 38.2, 26.0, 14.4, 12.9; **HRMS** (ESI-TOF) m/z: $[M + Na]^+$ Calcd for $C_{27}H_{30}O_3N_2Na^+$ 517.1097 (^{79}Br) and 519.1077 (^{81}Br); Found 517.1096 (^{79}Br) and 519.1078 (^{81}Br).

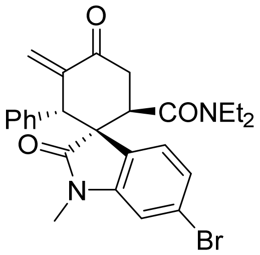

(1*R*,2*R*,6*R*)-6'-Bromo-N,N-diethyl-1'-methyl-3-methylene-2',4-dioxo-2-phenylspiro[cyclohexane-1,3'-indoline]-6-carboxamide (9d): Following **Conditions C**, *tert*-butyl (2-methylene-3-oxo-1-phenylbutyl)carbonate **1a** (41.4 mg, 0.150 mmol), (*E*)-2-(6-bromo-1-methyl-2-oxoindolin-3-ylidene)-N,N-diethylacetamide **8d** (33.7 mg, 0.100 mmol), $Pd(OAc)_2$ (1.1 mg, 0.0050 mmol), **C4** (4.4 mg, 0.0075 mmol) and (*S*)-**L3** (2.7 mg, 0.0075 mmol) was stirred in dry toluene (1.0 mL) at room temperature under Ar. After completion, the major product was obtained as a white solid by flash chromatography on silica gel (EtOAc/petroleum ether = 1/5–1/3); 30.3 mg, 61% yield; mp: 182−185 °C; 95% *ee*, determined by HPLC analysis [Daicel Chiral IE Column, *i*PrOH/*n*-hexane = 40/60, 1.0 mL/min, λ = 254 nm, t (major) = 14.14 min, t (minor) = 12.92 min]; $[\alpha]_D^{25}$ = −76.3 (*c* = 0.16, $CHCl_3$); ^{1}**H NMR** (400 MHz, $CDCl_3$): *δ* (ppm) 7.16–7.06 (m, 2H), 7.06–6.98 (m, 3H), 6.94–6.90 (m, 2H), 6.66 (d, *J* = 1.6 Hz, 1H), 6.28 (dd, *J* = 3.0, 1.4 Hz, 1H), 5.08 (t, *J* = 3.0 Hz, 1H), 4.94 (dd, *J* = 2.8, 1.4 Hz, 1H), 3.75 (dd, *J* = 16.8, 6.5 Hz, 1H), 3.63 (dq, *J* = 14.1, 7.1 Hz, 1H), 3.29–3.11 (m, 2H), 2.96 (s, 3H), 2.83–2.66 (m, 2H), 2.59 (dd, *J* = 16.8, 2.2 Hz, 1H), 1.19 (t, *J* = 7.1 Hz, 3H), 0.85 (t, *J* = 7.1 Hz, 3H); ^{13}**C NMR** (100 MHz, $CDCl_3$): *δ* (ppm) 197.6, 177.0, 171.3, 144.8, 144.2, 136.7, 130.3, 128.3, 127.7, 127.3, 125.9, 124.9, 123.7, 122.3, 111.4, 52.8, 51.2, 42.4, 41.1, 40.8, 38.2, 26.0, 14.4,

12.9; **HRMS** (ESI-TOF) m/z: $[M + Na]^+$ Calcd for $C_{27}H_{30}O_3N_2Na^+$ 517.1097 (^{79}Br) and 519.1077 (^{81}Br); Found 517.1105 (^{79}Br) and 519.1089 (^{81}Br).

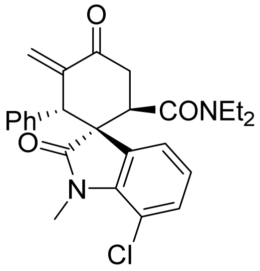

(1*R*,2*R*,6*R*)-7'-Chloro-N,N-diethyl-1'-methyl-3-methylene-2',4-dioxo-2-phenylspiro[cyclohexane-1,3'-indoline]-6-carboxamide (9e): Following **Conditions C**, *tert*-butyl (2-methylene-3-oxo-1-phenylbutyl) carbonate **1a** (41.4 mg, 0.150 mmol), (*E*)-2-(7-chloro-1-methyl-2-oxoindolin-3-ylidene)-N,N-diethylacetamide **8e** (28.3 mg, 0.100 mmol), $Pd(OAc)_2$ (1.1 mg, 0.0050 mmol), **C4** (4.4 mg, 0.0075 mmol) and (*S*)-**L3** (2.7 mg, 0.0075 mmol) was stirred in dry toluene (1.0 mL) at room temperature under Ar. After completion, the major product was obtained as a white solid by flash chromatography on silica gel (EtOAc/petroleum ether = 1/5–1/3); 25.3 mg, 56% yield; mp: 206–208 °C; 99% ee, determined by HPLC analysis [Daicel Chiral AD-H Column, *i*PrOH/*n*-hexane = 10/90, 1.0 mL/min, λ = 254 nm, t (major) = 11.93 min, t (minor) = 19.06 min]; $[\alpha]_D^{25}$ = +23.1 (*c* = 0.26, $CHCl_3$); **^{1}H NMR** (400 MHz, $CDCl_3$): *δ* (ppm) 7.17 (d, *J* = 7.6 Hz, 1H), 7.11–6.98 (m, 4H), 6.97–6.90 (m, 2H), 6.86 (t, *J* = 7.6 Hz, 1H), 6.29 (dd, *J* = 3.2, 1.4 Hz, 1H), 5.08 (t, *J* = 3.0 Hz, 1H), 4.94 (dd, *J* = 3.0, 1.4 Hz, 1H), 3.74 (dd, *J* = 16.7, 6.6 Hz, 1H), 3.64 (dq, *J* = 14.1, 7.1 Hz, 1H), 3.36 (s, 3H), 3.21 (dd, *J* = 6.6, 2.2 Hz, 1H), 3.19–3.11 (m, 1H), 2.80–2.65 (m, 2H), 2.59 (dd, *J* = 16.7, 2.2 Hz, 1H), 1.18 (t, *J* = 7.1 Hz, 3H), 0.84 (t, *J* = 7.1 Hz, 3H); **^{13}C NMR** (100 MHz, $CDCl_3$): *δ* (ppm) 197.6, 177.4, 171.3, 144.8, 138.9, 136.7, 132.0, 131.0, 130.3, 127.7, 127.3, 123.7, 123.2, 122.8, 115.2, 52.7, 51.3, 42.3, 41.1, 41.0, 38.3, 29.3, 14.3, 12.7; **HRMS** (ESI-TOF) m/z: $[M + H]^+$Calcd for $C_{26}H_{28}ClO_3N_2^+$ 451.1783 (^{35}Cl) and 453.1753 (^{37}Cl); Found 451.1776 (^{35}Cl) and 453.1767 (^{37}Cl).

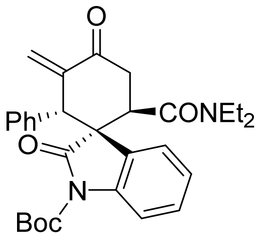

***tert*-Butyl (1*R*,2*R*,6*R*)-6-(diethylcarbamoyl)-3-methylene-2',4-dioxo-2-phenylspiro[cyclohexane-1,3'-indoline]-1'-carboxylate (9f):** Following **Conditions C**, *tert*-butyl (2-methylene-3-oxo-1-phenylbutyl)carbonate **1a** (41.4 mg, 0.150 mmol), *tert*-butyl (*E*)-3-(2-(diethylamino)-2-oxoethylidene)-2-oxoindoline-1-carboxylate **8f** (34.4 mg, 0.100 mmol), $Pd(OAc)_2$ (1.1 mg, 0.0050 mmol), **C4** (4.4 mg, 0.0075 mmol) and (*S*)-**L3** (2.7 mg, 0.0075 mmol) was stirred in dry toluene (1.0 mL) at room temperature under Ar. After completion, the major product was obtained as a semi-solid by flash

chromatography on silica gel (EtOAc/petroleum ether = 1/5–1/3); 23.1 mg, 46% yield; 99% ee, determined by HPLC analysis [Daicel Chiral IB Column, *i*PrOH/*n*-hexane = 20/80, 1.0 mL/min, λ = 254 nm, t (major) = 17.06 min, t (minor) = 19.43 min]; $[\alpha]_D^{25}$ = −15.7 (*c* = 0.46, $CHCl_3$); **^{1}H NMR** (400 MHz, $CDCl_3$): *δ* (ppm) 7.50 (d, *J* = 8.0 Hz, 1H), 7.29 (dd, *J* = 7.6, 1.4 Hz, 1H), 7.15 (td, *J* = 8.0, 1.4 Hz, 1H), 7.08–6.90 (m, 6H), 6.29 (dd, *J* = 3.0, 1.5 Hz, 0H), 5.09 (t, *J* = 2.9 Hz, 1H), 4.97 (dd, *J* = 2.9, 1.5 Hz, 1H), 3.70 (dd, *J* = 16.9, 6.5 Hz, 1H), 3.66–3.58 (m, 1H), 3.31 (dd, *J* = 6.5, 2.0 Hz, 1H), 3.17 (dq, *J* = 14.1, 7.1 Hz, 1H), 2.82–2.61 (m, 2H), 2.58 (dd, *J* = 16.9, 2.0 Hz, 1H), 1.59 (s, 9H), 1.19 (t, *J* = 7.1 Hz, 3H), 0.80 (t, *J* = 7.1 Hz, 3H); **^{13}C NMR** (100 MHz, $CDCl_3$): *δ* (ppm) 197.5, 175.7, 171.2, 148.8, 144.8, 138.9, 136.6, 130.4, 129.0, 128.1, 127.8, 127.3, 124.5, 124.0, 123.9, 114.5, 84.7, 53.0, 52.0, 42.3, 41.7, 41.1, 38.4, 28.1, 14.2, 12.7; **HRMS** (ESI-TOF) m/z: $[M + Na]^+$ Calcd for $C_{30}H_{34}O_5N_2Na^+$ 525.2360; Found 525.2360.

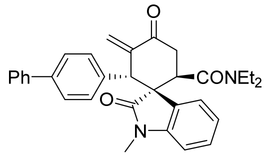

***(1R,2R,6R)*-2-([1,1'-Biphenyl]-4-yl)-N,N-diethyl-1'-methyl-3-methylene-2',4-dioxospiro[cyclohexane-1,3'-indoline]-6-carboxamide (9g):** Following **Conditions C**, 1-([1,1'-biphenyl]-4-yl)-2-methylene-3-oxobutyl *tert*-butyl carbonate **1d** (52.8 mg, 0.150 mmol), (*E*)-N,N-diethyl-2-(1-methyl-2-oxoindolin-3-ylidene) acetamide **8a** (25.8 mg, 0.100 mmol), $Pd(OAc)_2$ (1.1 mg, 0.0050 mmol), **C4** (4.4 mg, 0.0075 mmol) and (*S*)-**L3** (2.7 mg, 0.0075 mmol) was stirred in dry toluene (1.0 mL) at room temperature under Ar. After completion, the major product was obtained as a pale yellow solid by flash chromatography on silica gel (EtOAc/petroleum ether = 1/5–1/3); 26.9 mg, 55% yield; mp: 183–185 °C; 98% ee, determined by HPLC analysis [Daicel Chiral AD-H Column, *i*PrOH/*n*-hexane = 20/80, 1.0 mL/min, λ = 254 nm, t (major) = 12.08 min, t (minor) = 14.88 min]; $[\alpha]_D^{25}$ = −103.2 (*c* = 0.54, $CHCl_3$); **^{1}H NMR** (600 MHz, $CDCl_3$): *δ* (ppm) 7.46 (d, *J* = 7.8 Hz, 2H), 7.36 (t, *J* = 7.4 Hz, 2H), 7.31–7.21 (m, 4H), 7.13 (t, *J* = 7.6 Hz, 1H), 7.05–6.99 (m, 2H), 6.97 (t, *J* = 7.6 Hz, 1H), 6.52 (d, *J* = 7.8 Hz, 1H), 6.31 (s, 1H), 5.15 (s, 1H), 5.02 (s, 1H), 3.82 (dd, *J* = 16.7, 6.1 Hz, 1H), 3.66 (dq, *J* = 14.0, 7.0 Hz, 1H), 3.26 (d, *J* = 6.1 Hz, 1H), 3.20 (dq, *J* = 14.0, 7.0 Hz, 1H), 3.00 (s, 3H), 2.77–2.64 (m, 2H), 2.60 (d, *J* = 16.7 Hz, 1H), 1.21 (t, *J* = 7.1 Hz, 3H), 0.81 (t, *J* = 7.1 Hz, 3H); **^{13}C NMR** (100 MHz, $CDCl_3$): *δ* (ppm) 198.0, 177.2, 171.5, 145.1, 142.8, 140.4, 139.5, 136.2, 130.8, 129.2, 128.8, 128.7, 127.2, 126.8, 126.0, 124.6, 123.5, 122.2, 108.0, 52.9, 50.9, 42.3, 41.2, 41.0, 38.3, 26.0, 14.3, 12.8; **HRMS** (ESI-TOF) m/z: $[M + Na]^+$ Calcd for $C_{32}H_{32}O_3N_2Na^+$ 515.2305; Found 515.2300.

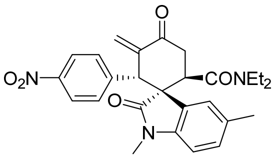

***(1R,2R,6R)*-N,N-Diethyl-1',5'-dimethyl-3-methylene-2-(4-nitrophenyl)-2',4-dioxospiro[cyclohexane-1,3'-indoline]-6-carboxamide (9h):** Following **Conditions C**, *tert*-butyl (2-methylene-1-(4-nitrophenyl)-3-oxobutyl)carbonate **1k** (48.4 mg, 0.150 mmol), (*E*)-2-(1,5-dimethyl-2-oxoindolin-3-ylidene)-N,N-diethylacetamide **8b** (27.2 mg, 0.100 mmol), $Pd(OAc)_2$ (1.1 mg, 0.0050 mmol), **C4** (4.4 mg, 0.0075 mmol) and (*S*)-**L3** (2.7 mg, 0.0075 mmol) was stirred in dry toluene (1.0 mL) at room temperature under Ar. After completion, the major product was obtained as a semi-solid by flash chromatography on silica gel (EtOAc/petroleum ether = 1/5–1/3); 25.0 mg, 53% yield; 97% ee, determined by HPLC analysis [Daicel Chiral IE Column, *i*PrOH/*n*-hexane = 40/60, 1.0 mL/min, λ = 254 nm, t (major) = 23.03 min, t (minor) = 19.66 min]; $[\alpha]_D^{25} = -135.4$ (*c* = 0.35, $CHCl_3$); **^{1}H NMR** (400 MHz, $CDCl_3$): *δ* (ppm) 8.00–7.77 (m, 2H), 7.20–7.12 (m, 2H), 7.09 (s, 1H), 6.96 (d, *J* = 7.9 Hz, 1H), 6.44 (d, *J* = 7.9 Hz, 1H), 6.32 (dd, *J* = 3.1, 1.1 Hz, 1H), 5.28 (t, *J* = 3.1 Hz, 1H), 4.83 (d, *J* = 1.6 Hz, 1H), 3.79 (dd, *J* = 16.9, 6.5 Hz, 1H), 3.75–3.67 (m, 1H), 3.23 (dd, *J* = 6.5, 1.9 Hz, 1H), 3.13 (dq, *J* = 14.1, 7.1 Hz, 1H), 2.98 (s, 3H), 2.81–2.63 (m, 2H), 2.57 (dd, *J* = 16.9, 1.9 Hz, 1H), 2.28 (s, 3H), 1.23 (t, *J* = 7.1 Hz, 3H), 0.81 (t, *J* = 7.1 Hz, 3H); **^{13}C NMR** (100 MHz, $CDCl_3$): *δ* (ppm) 196.8, 176.4, 171.5, 146.8, 145.3, 144.1, 140.2, 132.1, 131.5, 129.5, 128.4, 125.0, 123.6, 122.7, 108.1, 52.7, 50.7, 42.3, 41.2, 41.0, 38.0, 26.1, 21.1, 14.2, 13.0; **HRMS** (ESI-TOF) m/z: $[M + Na]^+$ Calcd for $C_{27}H_{29}O_5N_3Na^+$ 498.1999; Found 498.1999.

5.4 Asymmetric [4+2] annulations involving isatins

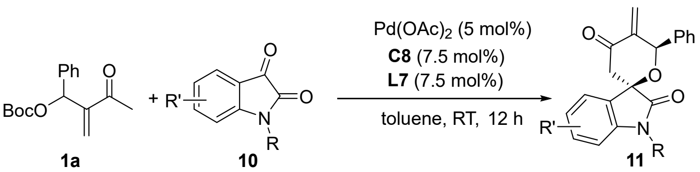

The reaction was conducted with MBH carbonate **1a** (0.12 mmol), isatin **10** (0.10 mmol), $Pd(OAc)_2$ (0.0050 mmol), **C8** (0.0075 mmol) and **L7** (0.0075 mmol) in dry toluene (1.0 mL) under Ar, and the mixture was stirred at room temperature for 12 h. After completion, the product was obtained by flash chromatography on silica gel (EtOAc/petroleum ether = 1/20–1/15). The racemates were obtained similarly by using the combination of achiral tetrabutylammonium bromide (TBAB) and triphenyl phosphite.

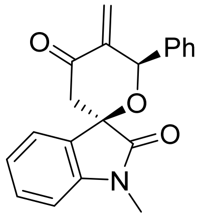

(3*R*,6'*R*)-1-Methyl-5'-methylene-6'-phenyl-5',6'-dihydrospiro[indoline-3,2'-pyran]-2,4'(3'*H*)-dione (11a): A solution of *tert*-butyl (2-methylene-3-oxo-1-phenylbutyl) carbonate **1a** (33.2 mg, 0.120 mmol), 1-methylindoline-2,3-dione **10a** (16.1 mg, 0.100 mmol), $Pd(OAc)_2$ (1.1 mg, 0.0050 mmol), **C8** (3.4 mg, 0.0075 mmol) and **L7** (3.4 mg, 0.0075 mmol) was stirred in dry toluene (1.0 mL) at room temperature under Ar. After completion, the product was obtained as a semi-solid by flash chromatography on silica gel (EtOAc/petroleum ether = 1/20–1/15); 19.8 mg, 62% yield; >19:1 dr; 87% ee, determined by HPLC analysis [Daicel Chiral IB Column, *i*PrOH/*n*-hexane = 10/90, 1.0 mL/min, λ = 254 nm, t (major) = 13.07 min, t (minor) = 10.72 min]; $[\alpha]_D^{25}$ = –82.7 (c = 0.14, $CHCl_3$); **^{1}H NMR** (400 MHz, $CDCl_3$): δ (ppm) 7.46–7.29 (m, 7H), 7.11 (td, J = 7.8, 1.1 Hz, 1H), 6.82 (d, J = 7.8 Hz, 1H), 6.32 (t, J = 2.1 Hz, 1H), 6.28 (dd, J = 2.1, 1.1 Hz, 1H), 4.87 (dd, J = 2.1, 1.1 Hz, 1H), 3.19 (s, 3H), 2.99 (d, J = 2.1 Hz, 2H); **^{13}C NMR** (150 MHz, $CDCl_3$): δ (ppm) 194.0, 174.5, 145.3, 143.1, 139.1, 130.4, 128.9, 128.51, 128.48, 128.2, 124.0, 123.5, 123.0, 108.6, 77.1, 76.7, 44.3, 26.0; **HRMS** (ESI-TOF) m/z: $[M + H]^+$ Calcd for $C_{20}H_{18}O_3N^+$ 320.1281; Found 320.1276.

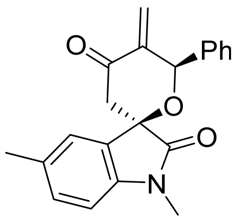

(3*R*,6'*R*)-1,5-Dimethyl-5'-methylene-6'-phenyl-5',6'-dihydrospiro[indoline-3,2'-pyran]-2,4'(3'*H*)-dione (11b): A solution of *tert*-butyl (2-methylene-3-oxo-1-phenylbutyl)carbonate **1a** (33.2 mg, 0.120 mmol), 1,5-dimethylindoline-2,3-dione **10b** (17.5 mg, 0.100 mmol), $Pd(OAc)_2$ (1.1 mg, 0.0050 mmol), **C8** (3.4 mg, 0.0075 mmol) and **L7** (3.4 mg, 0.0075 mmol) was stirred in dry toluene (1.0 mL) at room

temperature under Ar. After completion, the product was obtained as a semi-solid by flash chromatography on silica gel (EtOAc/petroleum ether = 1/20–1/15); 24.0 mg, 72% yield; >19:1 dr; 90% ee, determined by HPLC analysis [Daicel Chiral IE Column, *i*PrOH/*n*-hexane = 40/60, 1.0 mL/min, λ = 254 nm, t (major) = 8.86 min, t (minor) = 10.75 min]; $[\alpha]_D^{25}$ = –40.8 (*c* = 0.24, $CHCl_3$); **^{1}H NMR** (600 MHz, $CDCl_3$): *δ* (ppm) 7.41–7.31 (m, 5H), 7.18 (s, 1H), 7.13 (d, *J* = 7.9 Hz, 1H), 6.70 (d, *J* = 7.9 Hz, 1H), 6.32 (s, 1H), 6.28 (s, 1H), 4.86 (s, 1H), 3.16 (s, 3H), 3.05–2.90 (m, 2H), 2.34 (s, 3H); **^{13}C NMR** (150 MHz, $CDCl_3$): *δ* (ppm) 194.2, 174.5, 145.4, 140.7, 139.2, 133.3, 130.6, 128.9, 128.6, 128.5, 128.3, 124.9, 123.0, 108.4, 77.2, 76.9, 44.5, 26.1, 21.0; **HRMS** (ESI-TOF) m/z: $[M + H]^+$ Calcd for $C_{21}H_{20}O_3N^+$ 334.1438; Found 334.1442.

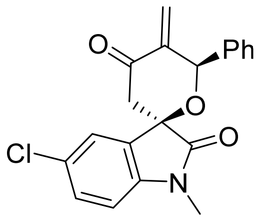

(3*R*,6'*R*)-5-Chloro-1-methyl-5'-methylene-6'-phenyl-5',6'-dihydrospiro [indoline-3,2'-pyran]-2,4'(3'*H*)-dione (11c): A solutioin of *tert*-butyl (2-methylene-3-oxo-1-phenylbutyl)carbonate **1a** (33.2 mg, 0.120 mmol), 1-methyl-5-chloroindoline-2,3-dione **10c** (19.6 mg, 0.100 mmol), $Pd(OAc)_2$ (1.1 mg, 0.0050 mmol), **C8** (3.4 mg, 0.0075 mmol) and **L7** (3.4 mg, 0.0075 mmol) was stirred in dry toluene (1.0 mL) at room temperature under Ar. After completion, the product was obtained as a semi-solid by flash chromatography on silica gel (EtOAc/petroleum ether = 1/20–1/15); 30.1 mg, 85% yield; >19:1 dr; 84% ee, determined by HPLC analysis [Daicel Chiral ID Column, *i*PrOH/*n*-hexane = 20/80, 1.0 mL/min, λ = 254 nm, t (major) = 14.91 min, t (minor) = 13.04 min]; $[\alpha]_D^{25}$ = +18.6 (*c* = 0.14, $CHCl_3$); **^{1}H NMR** (400 MHz, $CDCl_3$): *δ* (ppm) 7.45–7.28 (m, 7H), 6.75 (d, *J* = 8.2 Hz, 1H), 6.31–6.29 (m, 2H), 4.88 (s, 1H), 3.17 (s, 3H), 2.97 (s, 2H); **^{13}C NMR** (100 MHz, $CDCl_3$): *δ* (ppm) 193.5, 174.2, 145.1, 141.7, 138.8, 130.5, 130.4, 129.0, 128.8, 128.7, 128.3, 124.8, 123.5, 109.8, 77.4, 77.2, 44.2, 26.3; **HRMS** (ESI-TOF) m/z: $[M + Na]^+$ Calcd for $C_{20}H_{16}O_3NClNa^+$ 376.0711 (^{35}Cl) and 378.0681 (^{37}Cl); Found 376.0708 (^{35}Cl) and 378.0655 (^{37}Cl).

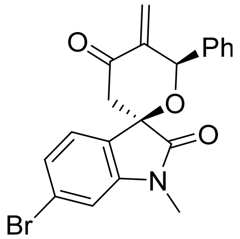

(3*R*,6'*R*)-6-Bromo-1-methyl-5'-methylene-6'-phenyl-5',6'-dihydrospiro [indoline-3,2'-pyran]-2,4'(3'*H*)-dione (11d): A solution of *tert*-butyl (2-methylene-3-oxo-1-phenylbutyl)carbonate **1a** (33.2 mg, 0.120 mmol), 1-methyl-6-bromoindoline-2,3-dione **10d** (24.0 mg, 0.100 mmol), $Pd(OAc)_2$ (1.1 mg, 0.0050 mmol), **C8** (3.4 mg, 0.0075 mmol) and **L7** (3.4 mg, 0.0075 mmol) was stirred in dry toluene (1.0 mL) at room temperature under Ar. After completion, the product was

obtained as a semi-solid by flash chromatography on silica gel (EtOAc/petroleum ether = 1/20–1/15); 24.2 mg, 62% yield; >19:1 dr; 84% ee, determined by HPLC analysis [Daicel Chiral IF Column, *i*PrOH/*n*-hexane = 40/60, 1.0 mL/min, λ = 254 nm, t (major) = 12.23 min, t (minor) = 9.45 min]; $[\alpha]_D^{25}$ = –23.5 (*c* = 0.17, $CHCl_3$); **^{1}H NMR** (400 MHz, $CDCl_3$): *δ* (ppm) 7.42–7.30 (m, 5H), 7.26–7.20 (m, 2H), 6.98 (d, *J* = 1.6 Hz, 1H), 6.29 (s, 2H), 4.88 (s, 1H), 3.17 (s, 3H), 3.02–2.90 (m, 2H); **^{13}C NMR** (150 MHz, $CDCl_3$): *δ* (ppm) 193.6, 174.4, 145.2, 144.5, 138.8, 128.7, 128.6, 128.2, 127.9, 126.4, 125.4, 124.3, 123.4, 112.3, 77.3, 76.5, 44.1, 26.2.; **HRMS** (ESI-TOF) m/z: $[M + Na]^+$ Calcd for $C_{20}H_{16}O_3NBrNa^+$ 398.0386 (^{79}Br) and 400.0366 (^{81}Br); Found 398.0385 (^{79}Br) and 400.0350 (^{81}Br).

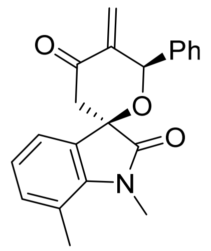

(3*R*,6'*R*)-1,7-Dimethyl-5'-methylene-6'-phenyl-5',6'-dihydrospiro[indoline-3,2'-pyran]-2,4'(3'*H*)-dione (11e): A solution of *tert*-butyl (2-methylene-3-oxo-1-phenylbutyl) carbonate **1a** (33.2 mg, 0.120 mmol), 1,7-dimethylindoline-2,3-dione **10e** (17.5 mg, 0.100 mmol), $Pd(OAc)_2$ (1.1 mg, 0.0050 mmol), **C8** (3.4 mg, 0.0075 mmol) and **L7** (3.4 mg, 0.0075 mmol) was stirred in dry toluene (1.0 mL) at room temperature under Ar. After completion, the product was obtained as a semi-solid by flash chromatography on silica gel (EtOAc/petroleum ether = 1/20–1/15); 27.0 mg, 81% yield; >19:1 dr; 92% ee, determined by HPLC analysis [Daicel Chiral IF Column, *i*PrOH/*n*-hexane = 40/60, 1.0 mL/min, λ = 254 nm, t (major) = 18.36 min, t (minor) = 9.83 min]; $[\alpha]_D^{25}$ = –68.5 (*c* = 0.47, CH_2Cl_2); **^{1}H NMR** (400 MHz, $CDCl_3$): *δ* (ppm) 7.43–7.28 (m, 5H), 7.21 (dd, *J* = 7.4, 1.4 Hz, 1H), 7.07 (d, *J* = 7.7 Hz, 1H), 6.99 (t, *J* = 7.4 Hz, 1H), 6.30 (s, 1H), 6.27 (s, 1H), 4.85 (t, *J* = 1.4 Hz, 1H), 3.46 (s, 3H), 3.19–2.80 (m, 2H), 2.53 (s, 3H); **^{13}C NMR** (100 MHz, $CDCl_3$): *δ* (ppm) 194.3, 175.2, 145.4, 140.8, 139.3, 134.2, 129.6, 128.57, 128.56, 128.3, 123.5, 123.1, 122.1, 120.4, 77.2, 76.2, 44.7, 29.5, 18.8; **HRMS** (ESI-TOF) m/z: $[M + Na]^+$ Calcd for $C_{21}H_{19}O_3NNa^+$ 356.1257; Found 356.1248.

For the reaction on a 1.0 mmol scale: *tert*-butyl (2-methylene-3-oxo-1-phenylbutyl)carbonate **1a** (332 mg, 1.20 mmol), 1,7-dimethylindoline-2,3-dione **10e** (175 mg, 1.00 mmol), $Pd(OAc)_2$ (11.2 mg, 0.0500 mmol), **C8** (34.0 mg, 0.0750 mmol) and **L7** (34.0 mg, 0.0750 mmol) were stirred in dry toluene (10.0 mL) at room temperature under Ar. After completion, the product was obtained as a semi-solid by flash chromatography on silica gel (EtOAc/petroleum ether = 1/20–1/15); 240 mg, 72% yield; >19:1 dr, 91% ee.

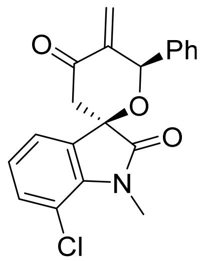

(3*R*,6'*R*)-7-Chlorl-1-methyl-5'-methylene-6'-phenyl-5',6'-dihydrospiro [indoline-3,2'-pyran]-2,4'(3'*H*)-dione (11f): A solution of *tert*-butyl (2-methylene-3-oxo-1-phenylbutyl) carbonate **1a** (33.2 mg, 0.120 mmol), 1-methyl-7-chloroindoline-2,3-dione **10f** (19.6 mg, 0.100 mmol), $Pd(OAc)_2$ (1.1 mg, 0.0050 mmol), **C8** (3.4 mg, 0.0075 mmol) and **L7** (3.4 mg, 0.0075 mmol) was stirred in dry toluene (1.0 mL) at room temperature under Ar. After completion, the product was obtained as a semi-solid by flash chromatography on silica gel (EtOAc/petroleum ether = 1/20–1/15); 23.7 mg, 67% yield; >19:1 dr; 80% ee, determined by HPLC analysis [Daicel Chiral IF Column, *i*PrOH/*n*-hexane = 40/60, 1.0 mL/min, λ = 254 nm, t (major) = 15.89 min, t (minor) = 7.93 min]; $[\alpha]_D^{25}$ = –72.5 (*c* = 0.32, CH_2Cl_2); **^{1}H NMR** (400 MHz, Acetone-*d*6): *δ* (ppm) 7.57–7.30 (m, 7H), 7.13 (dd, *J* = 8.3, 7.3 Hz, 1H), 6.22 (t, *J* = 2.1 Hz, 1H), 6.16–6.11 (m, 1H), 5.27–4.60 (m, 1H), 3.52 (s, 3H), 3.21 (d, *J* = 16.6 Hz, 1H), 2.90 (d, *J* = 16.6 Hz, 1H); **^{13}C NMR** (150 MHz, Acetone-*d*6): *δ* (ppm) 193.9, 175.5, 147.0, 141.0, 140.4, 133.4, 133.1, 129.39, 129.36, 129.2, 125.3, 124.2, 122.3, 116.3, 78.1, 77.3, 45.0, 29.8; **HRMS** (ESI-TOF) m/z: $[M + Na]^+$ Calcd for $C_{20}H_{16}O_3NClNa^+$ 376.0711 (^{35}Cl) and 378.0681 (^{37}Cl); Found 376.0706 (^{35}Cl) and 378.0667 (^{37}Cl).

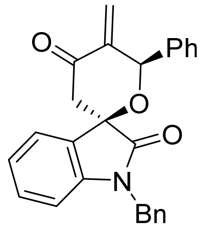

(3*R*,6'*R*)-1-Benzyl-5'-methylene-6'-phenyl-5',6'-dihydrospiro[indoline-3,2'-pyran]-2,4'(3'*H*)-dione (11g): A solution of *tert*-butyl (2-methylene-3-oxo-1-phenylbutyl) carbonate **1a** (33.2 mg, 0.120 mmol), 1-benzylindoline-2,3-dione **10g** (23.7 mg, 0.100 mmol), $Pd(OAc)_2$ (1.1 mg, 0.0050 mmol), **C8** (3.4 mg, 0.0075 mmol) and **L7** (3.4 mg, 0.0075 mmol) was stirred in dry toluene (1.0 mL) at room temperature under Ar. After completion, the product was obtained as a white solid by flash chromatography on silica gel (EtOAc/petroleum ether = 1/20–1/15); 30.9 mg, 78% yield; mp: 116–118 °C; >19:1 dr; 80% ee, determined by HPLC analysis [Daicel Chiral IE Column, *i*PrOH/*n*-hexane = 40/60, 1.0 mL/min, λ = 254 nm, t (major) = 9.81 min, t (minor) = 9.17 min]; $[\alpha]_D^{25}$ = –46.7 (*c* = 0.33, $CHCl_3$); **^{1}H NMR** (600 MHz, Acetone-d_6): *δ* (ppm) ^{1}H NMR (600 MHz,) δ 7.57 (dd, *J* = 7.4, 1.3 Hz, 1H), 7.55–7.51 (m, 2H), 7.48–7.27 (m, 8H), 7.15 (t, *J* = 7.4 Hz, 1H), 6.95 (d, *J* = 7.9 Hz, 1H), 6.35 (t, *J* = 2.2 Hz, 1H), 6.20 (t, *J* = 1.7 Hz, 1H), 5.03–4.97 (m, 2H), 4.83 (t, *J* = 1.7 Hz, 1H), 3.31 (d, *J* = 16.6 Hz, 1H), 2.99 (d, *J* = 16.6 Hz, 1H), 2.84–2.81 (m, 1H); **^{13}C NMR** (100 MHz, $CDCl_3$): *δ* (ppm) 194.1, 174.7, 145.3, 142.3, 139.3, 135.1, 130.4, 129.0, 128.9, 128.7, 128.6, 128.3, 127.9, 127.3, 124.2, 123.6, 123.4, 109.8,

77.2, 77.0, 44.7, 43.7; **HRMS** (ESI-TOF) m/z: $[M + H]^+$ Calcd for $C_{26}H_{22}O_3N^+$ 396.1594; Found 396.1590.

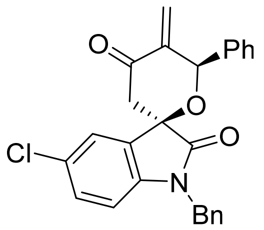

(3*R*,6'*R*)-5-Chloro-1-benzyl-5'-methylene-6'-phenyl-5',6'-dihydrospiro [indoline-3,2'-pyran]-2,4'(3'*H*)-dione (11h): A solution of *tert*-butyl (2-methylene-3-oxo-1-phenylbutyl) carbonate **1a** (33.2 mg, 0.120 mmol), 1-benzyl-5-chloroindoline-2,3-dione **10h** (27.2 mg, 0.100 mmol), $Pd(OAc)_2$ (1.1 mg, 0.0050 mmol), **C8** (3.4 mg, 0.0075 mmol) and **L7** (3.4 mg, 0.0075 mmol) was stirred in dry toluene (1.0 mL) at room temperature under Ar. After completion, the product was obtained as a white solid by flash chromatography on silica gel (EtOAc/petroleum ether = 1/20–1/15); 28.0 mg, 65% yield; mp: 138−139 °C; >19:1 dr; 80% ee, determined by HPLC analysis [Daicel Chiral IE Column, *i*PrOH/*n*-hexane = 40/60, 1.0 mL/min, λ = 254 nm, t (major) = 7.42 min, t (minor) = 8.42 min]; $[\alpha]_D^{25}$ = −7.1 (*c* = 0.34, $CHCl_3$); **^{1}H NMR** (400 MHz, $CDCl_3$): *δ* (ppm) 7.47−7.27 (m, 9H), 7.25−7.23 (m, 2H), 7.18 (dd, *J* = 8.4, 2.1 Hz, 1H), 6.61 (d, *J* = 8.4 Hz, 1H), 6.35 (t, *J* = 2.1 Hz, 1H), 6.32 (dd, *J* = 2.1, 1.0 Hz, 1H), 4.98–4.90 (m, 2H), 4.78 (d, *J* = 15.7 Hz, 1H), 3.01 (s, 2H); **^{13}C NMR** (100 MHz, $CDCl_3$): *δ* (ppm) 193.5, 174.4, 145.0, 140.8, 139.0, 134.7, 130.5, 130.3, 129.1, 129.0, 128.8, 128.7, 128.3, 128.0, 127.2, 124.8, 123.7, 110.9, 77.5, 76.9, 44.5, 43.8; **HRMS** (ESI-TOF) m/z: $[M + Na]^+$ Calcd for $C_{26}H_{20}O_3NClNa^+$ 452.1024 (^{35}Cl) and 454.0994 (^{37}Cl); Found 452.1008 (^{35}Cl) and 454.0995 (^{37}Cl).

5.5 Transformations of the [4+2] annulation product 3a

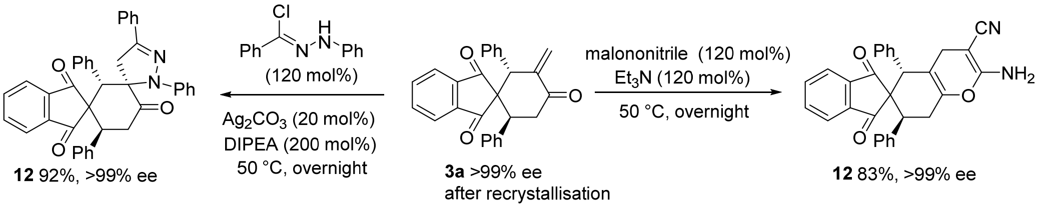

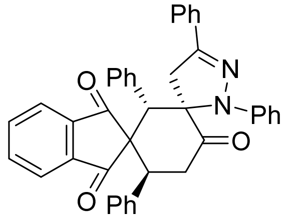

(2'*S*,3'*R*,6'*S*)-2',2'',5'',6'-Tetraphenyl-2'',4''-dihydrodispiro[indene-2,1'-cyclohexane-3',3''-pyrazole]-1,3,4'-trione (12): A mixture of **3a** (9.9 mg, 0.025 mmol, >99% ee (after recrystallization)), (*Z*)-N-phenyl benzohydrazonoyl chloride (7.8 mg, 0.030 mmol), Ag_2CO_3 (1.4 mg, 0.0050 mmol) and DIPEA (0.05 mmol, 6 mg) in toluene (0.25 mL) was stirred at 50 °C overnight.[5] After completion, product **12** was obtained as a pale yellow solid by flash chromatography on silica gel (acetone/petroleum ether = 1/20–1/15), 13.5 mg, 92% yield; mp 267–269 °C; >19:1 dr; >99% ee, determined by HPLC analysis [Daicel Chiral IE Column, *i*PrOH/*n*-hexane = 40/60, 1.0 mL/min, λ = 254 nm, t (major) = 9.04 min, t (minor) = 10.41 min]; $[\alpha]_D^{25}$ = –387.7 (*c* = 0.26, $CHCl_3$); **^{1}H NMR** (400 MHz, $CDCl_3$): *δ* (ppm) 7.89–7.72 (m, 2H), 7.62–7.53 (m, 1H), 7.51–7.33 (m, 6H), 7.15 (t, *J* = 7.7 Hz, 2H), 7.10–6.95 (m, 5H), 6.86–6.75 (m, 4H), 6.66 (t, *J* = 7.7 Hz, 2H), 6.58 (d, *J* = 7.7 Hz, 2H), 5.39 (d, *J* = 17.4 Hz, 1H), 4.89 (s, 1H), 4.28 (dd, *J* = 16.7, 15.0 Hz, 1H), 4.11 (dd, *J* = 15.0, 2.2 Hz, 1H), 3.72 (d, *J* = 17.4 Hz, 1H), 2.96 (dd, *J* = 16.7, 2.2 Hz, 1H); **^{13}C NMR** (100 MHz, $CDCl_3$): *δ* (ppm) 208.1, 203.3, 202.5, 145.0, 142.5, 142.3, 141.6, 136.1, 135.6, 132.8, 132.4, 129.9, 128.7, 128.61, 128.58, 128.4, 128.3, 127.8, 127.74, 127.71, 125.8, 122.6, 122.5, 120.1, 115.4, 76.4, 63.2, 48.0, 44.8, 43.0, 40.8; **HRMS** (ESI-TOF) m/z: $[M + Na]^+$ Calcd for $C_{40}H_{30}N_2NaO_3^+$ 609.2149; Found 609.2136.

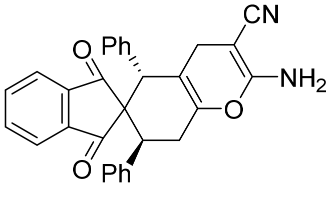

(5*R*,7*S*)-2-Amino-1',3'-dioxo-5,7-diphenyl-1',3',7,8-tetrahydro-4H,5H-spiro[chromene-6,2'-indene]-3-carbonitrile (**13**):A solution of **3a** (14.9 mg, 0.0375 mmol, >99% ee), malononitrine (3.0 mg, 0.045 mmol), and TEA (0.045 mmol, 6.0 *μl*) in toluene (0.4 mL) was stirred at 50 °C overnight.[6] After completion, product **13** was obtained as a white solid by flash chromatography on silica gel (petroleum ether/EtOAc/dichloromethane = 6/1/1), 14.2 mg, 83% yield; mp 164–167 °C; >99% ee, determined by HPLC analysis [Daicel Chiral IE Column, *i*PrOH/*n*-hexane = 40/60, 1.0 mL/min, λ = 254 nm, t (major) = 10.10 min, t (minor) = 7.75 min]; $[\alpha]_D^{25}$ = –42.1 (*c* = 0.28, $CHCl_3$);

^{1}H NMR (400 MHz, $CDCl_3$): δ (ppm) 7.95 (d, J = 7.6 Hz, 1H), 7.75 (td, J = 7.4, 1.2 Hz, 1H), 7.68 (td, J = 7.4, 1.2 Hz, 1H), 7.61 (d, J = 7.6 Hz, 1H), 7.43–7.30 (m, 3H), 7.16–6.99 (m, 7H), 4.44 (s, 2H), 3.71 (dd, J = 11.1, 6.4 Hz, 1H), 3.32 (s, 1H), 3.26–3.17 (m, 1H), 2.66 (dd, J = 17.6, 6.4 Hz, 1H), 2.56–2.45 (m, 2H); **^{13}C NMR** (100 MHz, $CDCl_3$): δ (ppm) 201.3, 198.7, 159.6, 144.5, 141.7, 141.0, 138.9, 137.1, 135.7, 135.5, 129.0, 128.6, 128.3, 128.1, 127.3, 123.5, 123.1, 120.3, 105.0, 60.5, 54.2, 50.5, 39.8, 30.6, 24.5; **HRMS** (ESI-TOF) m/z: $[M + Na]^+$ Calcd for $C_{30}H_{22}NaN_2O_3^+$ 481.1523; Found 481.1520.

5.6 Dimerisation of 11e

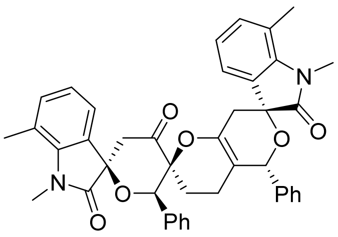

(3*R*,5'*S*,5''*R*,6'*R*,7''*R*)-1,1''',7,7'''-Tetramethyl-5'',6'-diphenyl-3'',4'',5'',8''-tetrahydro-6'H-trispiro[indoline-3,2'-pyran-5',2''-pyrano[4,3-*b*]pyran-7'',3'''-indoline]-2,2''',4'(3'*H*)-trione (14): A solution of compound **11e** (239 mg, 0.720 mmol) in $CHCl_3$ was concentrated and let stand for 5 days. The dimer product was obtained as a white solid by flash chromatography on silica gel (EtOAc/petroleum ether = 1/10–1/7), 172 mg, 72% yield; mp: 264–266 °C; >19:1 dr; >99% ee, determined by HPLC analysis [Daicel Chiral IE Column, *i*PrOH/*n*-hexane = 40/60, 1.0 mL/min, λ = 254 nm, t (major) = 11.23 min, t (minor) = 15.92 min]; $[\alpha]_D^{25}$ = −138.6 (c = 0.14, $CHCl_3$); **^{1}H NMR** (400 MHz, $CDCl_3$): δ (ppm) 7.47–7.45 (m, 2H), 7.37 (d, J = 6.9 Hz, 1H), 7.32–7.17 (m, 9H), 7.10–7.00 (m, 3H), 6.96 (t, J = 7.5 Hz, 1H), 5.75 (s, 1H), 5.52 (s, 1H), 3.70 (d, J = 13.1 Hz, 1H), 3.43 (s, 3H), 3.39 (s, 3H), 2.93 (d, J = 16.8 Hz, 1H), 2.55 (s, 3H), 2.52 (s, 3H), 2.50–2.42 (m, 2H), 2.15 (dd, J = 13.4, 7.1 Hz, 1H), 1.94–1.85 (m, 1H), 1.52 (dd, J = 16.8, 6.0 Hz, 1H), 1.41–1.33 (m, 1H); **^{13}C NMR** (150 MHz, $CDCl_3$): δ (ppm) 202.8, 175.1, 175.0, 141.0, 140.8, 139.5, 139.4, 135.7, 134.1, 133.7, 130.6, 129.22, 129.17, 128.6, 128.5, 128.3, 128.2, 127.9, 123.3, 123.0, 122.10, 122.06, 120.3, 119.7, 107.6, 81.5, 81.2, 77.5, 76.9, 74.9, 43.5, 33.1, 29.2, 22.6, 18.9, 18.8, 18.4; **HRMS** (ESI-TOF) m/z: $[M + H]^+$ Calcd for $C_{42}H_{39}O_6N_2^+$ 667.2803; Found 667.2802.

6. Crystal data for enantiopure products 3s, 6g, 9c and 14

Preparation of the single crystals of enantiopure 3s: Compound **3s** (20.0 mg, 93% ee) was dissolved in *i*PrOH (1.0 mL) in a 10 mL tube and *n*-hexane (3.0 mL) was added. The tube was sealed by parafilm with several tiny holes, thus allowing slow evaporation of the solvents at room temperature. After 72 h, several small particles could be observed at the bottom of the tube. The crystals were chosen and subjected to the single crystal X-ray diffraction analysis for the determination of the absolute configuration of **3s**. The data were collected by an Agilent Gemini equipped with a Cu radiation source (Kα = 1.54184 Å) at 289.62(18) K. CCDC 2073270 (**3s**) contains the supplementary crystallographic data for this paper. These data can be obtained free of charge via www.ccdc.cam.ac.uk/data_request/cif.

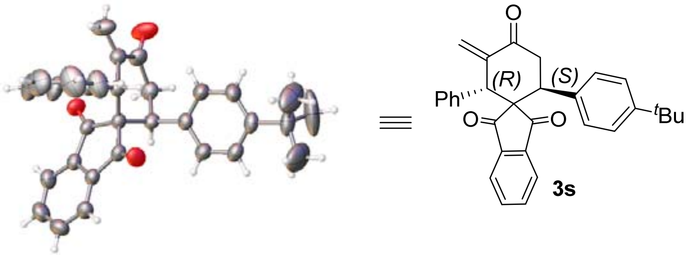

Table S2 Crystal data and structure refinement for **3s**

Identification code	**3s**
Empirical formula	$C_{31}H_{28}O_3$
Formula weight	448.53
Temperature/K	289.62(18)
Crystal system	orthorhombic
Space group	$P2_12_12_1$
a/Å	9.4508(3)
b/Å	10.5895(3)
c/Å	24.6688(6)
α/°	90
β/°	90
γ/°	90
Volume/Å^3	2468.84(12)
Z	4
ρ_{calc}g/cm^3	1.207
μ/mm^{-1}	0.602
F(000)	952.0
Crystal size/mm^3	0.7 × 0.5 × 0.1
Radiation	CuKα (λ = 1.54184)
2Θ range for data collection/°	9.088 to 134.102
Index ranges	$-7 \le h \le 11, -10 \le k \le 12, -26 \le l \le 29$

Reflections collected	10698
Independent reflections	4401 [R_{int} = 0.0418, R_{sigma} = 0.0453]
Data/restraints/parameters	4401/21/310
Goodness-of-fit on F^2	1.033
Final R indexes [I>=2σ (I)]	R_1 = 0.0632, wR_2 = 0.1676
Final R indexes [all data]	R_1 = 0.0668, wR_2 = 0.1746
Largest diff. peak/hole / e $Å^{-3}$	0.26/-0.32
Flack parameter	0.1(2)

The absolute structures of two diastereomers from 3-olefinic oxindoles were confirmed by X-ray crystal diffraction analysis.

Preparation of the single crystals of enantiopure 6g: Compound **6g** (20.0 mg, >99% ee) was dissolved in EtOAc (1.0 mL) in a 10 mL tube and *n*-hexane (3.0 mL) was added. The tube was sealed by parafilm with several tiny holes, thus allowing slow evaporation of the solvents at room temperature. After 48 h, several small particles could be observed at the bottom of the tube. The crystals were chosen and subjected to the single crystal X-ray diffraction analysis for the determination of the absolute configuration of **6g**. The data were collected by an Agilent Gemini equipped with a Cu radiation source (Kα = 1.54184 Å) at 296.2(4) K. CCDC 2073271 (**6g**) contains the supplementary crystallographic data for this paper. These data can be obtained free of charge via www.ccdc.cam.ac.uk/data_request/cif.

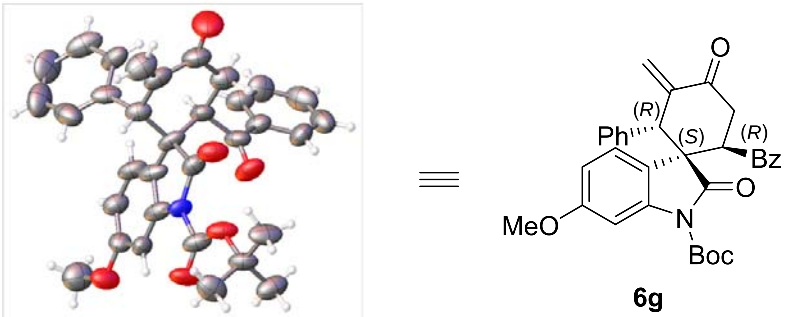

6g

Table S3 Crystal data and structure refinement for **6g**

Identification code	**6g**
Empirical formula	$C_{33}H_{31}NO_6$
Formula weight	537.59
Temperature/K	296.2(4)
Crystal system	orthorhombic
Space group	$P2_12_12_1$
a/Å	17.1259(4)
b/Å	16.6316(3)
c/Å	9.8931(2)
α/°	90
β/°	90

γ/°	90
Volume/Å^3	2817.86(10)
Z	4
ρ_{calc}g/cm^3	1.267
μ/mm^{-1}	0.707
F(000)	1136.0
Crystal size/mm^3	0.45 × 0.15 × 0.15
Radiation	CuKα (λ = 1.54184)
2Θ range for data collection/°	7.41 to 142.812
Index ranges	$-20 \le h \le 20$, $-15 \le k \le 20$, $-11 \le l \le 12$
Reflections collected	15409
Independent reflections	5384 [R_{int} = 0.0539, R_{sigma} = 0.0397]
Data/restraints/parameters	5384/0/365
Goodness-of-fit on F^2	1.061
Final R indexes [I>=2σ (I)]	R_1 = 0.1316, wR_2 = 0.3236
Final R indexes [all data]	R_1 = 0.1356, wR_2 = 0.3259
Largest diff. peak/hole / e Å^{-3}	0.39/-0.38
Flack parameter	0.05(18)

Preparation of the single crystals of enantiopure 9c: Compound **9c** (20.0 mg, 99% ee) was dissolved in EA (1.0 mL) in a 10 mL tube and *n*-hexane (3.0 mL) was added. The tube was sealed by parafilm with several tiny holes, thus allowing slow evaporation of the solvents at room temperature. After 48 h, several small particles could be observed at the bottom of the tube. The crystals were chosen and subjected to the single crystal X-ray diffraction analysis for the determination of the absolute configuration of **9c**. The data were collected by an Agilent Gemini equipped with a Cu radiation source (Kα = 1.54184 Å) at 296.8(5) K. CCDC 2073272 (**9c**) contains the supplementary crystallographic data for this paper. These data can be obtained free of charge via www.ccdc.cam.ac.uk/data_request/cif.

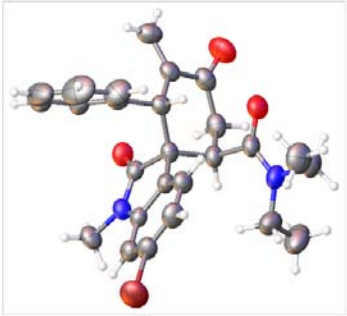

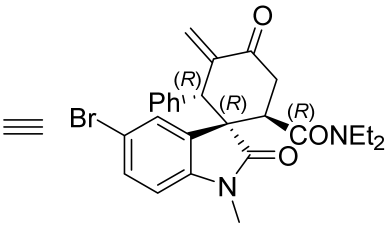

9c

Table S4 Crystal data and structure refinement for **9c**

Identification code	**9c**
Empirical formula	$C_{26}H_{27}BrN_2O_3$

Formula weight	495.40
Temperature/K	296.8(5)
Crystal system	hexagonal
Space group	$P6_2$
a/Å	19.5054(4)
b/Å	19.5054(4)
c/Å	10.8702(2)
α/°	90
β/°	90
γ/°	120
Volume/Å^3	3581.62(16)
Z	6
ρ_{calc}g/cm^3	1.378
μ/mm^{-1}	2.582
F(000)	1536.0
Crystal size/mm^3	0.6 × 0.2 × 0.2
Radiation	CuKα (λ = 1.54184)
2Θ range for data collection/°	9.068 to 142.586
Index ranges	$-23 \leq h \leq 12, -20 \leq k \leq 23, -13 \leq l \leq 11$
Reflections collected	20115
Independent reflections	4217 [R_{int} = 0.0564, R_{sigma} = 0.0328]
Data/restraints/parameters	4217/1/292
Goodness-of-fit on F^2	1.056
Final R indexes [I>=2σ (I)]	R_1 = 0.0458, wR_2 = 0.1230
Final R indexes [all data]	R_1 = 0.0482, wR_2 = 0.1269
Largest diff. peak/hole / e Å^{-3}	0.52/-0.28
Flack parameter	-0.020(12)

The absolute configuration of the dimerisation product **14** from **11e** was determined by X-ray crystal diffraction analysis.

Preparation of the single crystals of enantiopure 14: Compound **14** (20.0 mg, >99% ee) was dissolved in DCM (1.0 mL) in a 10 mL tube and *n*-hexane (3.0 mL) was added. The tube was sealed by parafilm with several tiny holes, thus allowing slow evaporation of the solvents at room temperature. After 48 h, several small particles could be observed at the bottom of the tube. The crystals were chosen and subjected to the single crystal X-ray diffraction analysis for the determination of the absolute configuration of **14**. The data were collected by an Agilent Gemini equipped with a Cu radiation source (Kα = 1.54184 Å) at 150.00(10) K. CCDC 2073273 (**14**) contains the supplementary crystallographic data for this paper. These data can be obtained free of charge via www.ccdc.cam.ac.uk/data_request/cif.

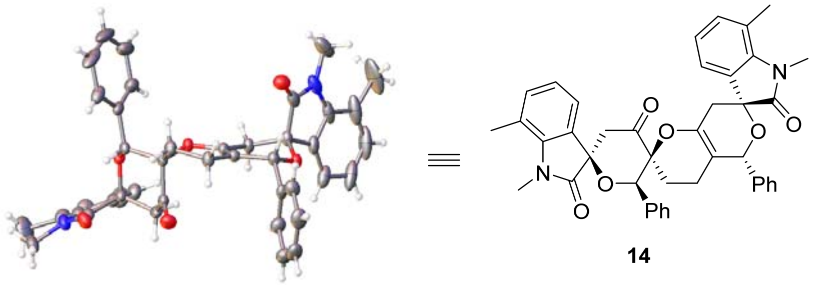

14

Table S5 Crystal data and structure refinement for **14**

Identification code	**14**
Empirical formula	$C_{42}H_{38}N_2O_6$
Formula weight	666.74
Temperature/K	150.00(10)
Crystal system	orthorhombic
Space group	$P2_12_12_1$
a/Å	9.66433(13)
b/Å	12.43414(15)
c/Å	28.7610(4)
α/°	90
β/°	90
γ/°	90
Volume/Å^3	3456.14(8)
Z	4
ρ_{calc}g/cm^3	1.281
μ/mm^{-1}	0.691
F(000)	1408.0
Crystal size/mm^3	0.6 × 0.5 × 0.3
Radiation	CuKα (λ = 1.54184)
2Θ range for data collection/°	7.746 to 143.004

Index ranges	$-11 \leq h \leq 11$, $-15 \leq k \leq 11$, $-35 \leq l \leq 35$
Reflections collected	18584
Independent reflections	6621 [R_{int} = 0.0431, R_{sigma} = 0.0379]
Data/restraints/parameters	6621/0/455
Goodness-of-fit on F^2	1.037
Final R indexes [I>=2σ (I)]	R_1 = 0.0480, wR_2 = 0.1242
Final R indexes [all data]	R_1 = 0.0501, wR_2 = 0.1274
Largest diff. peak/hole / e $Å^{-3}$	0.19/-0.35
Flack parameter	-0.06(10)

7. More unsuccessful attempts

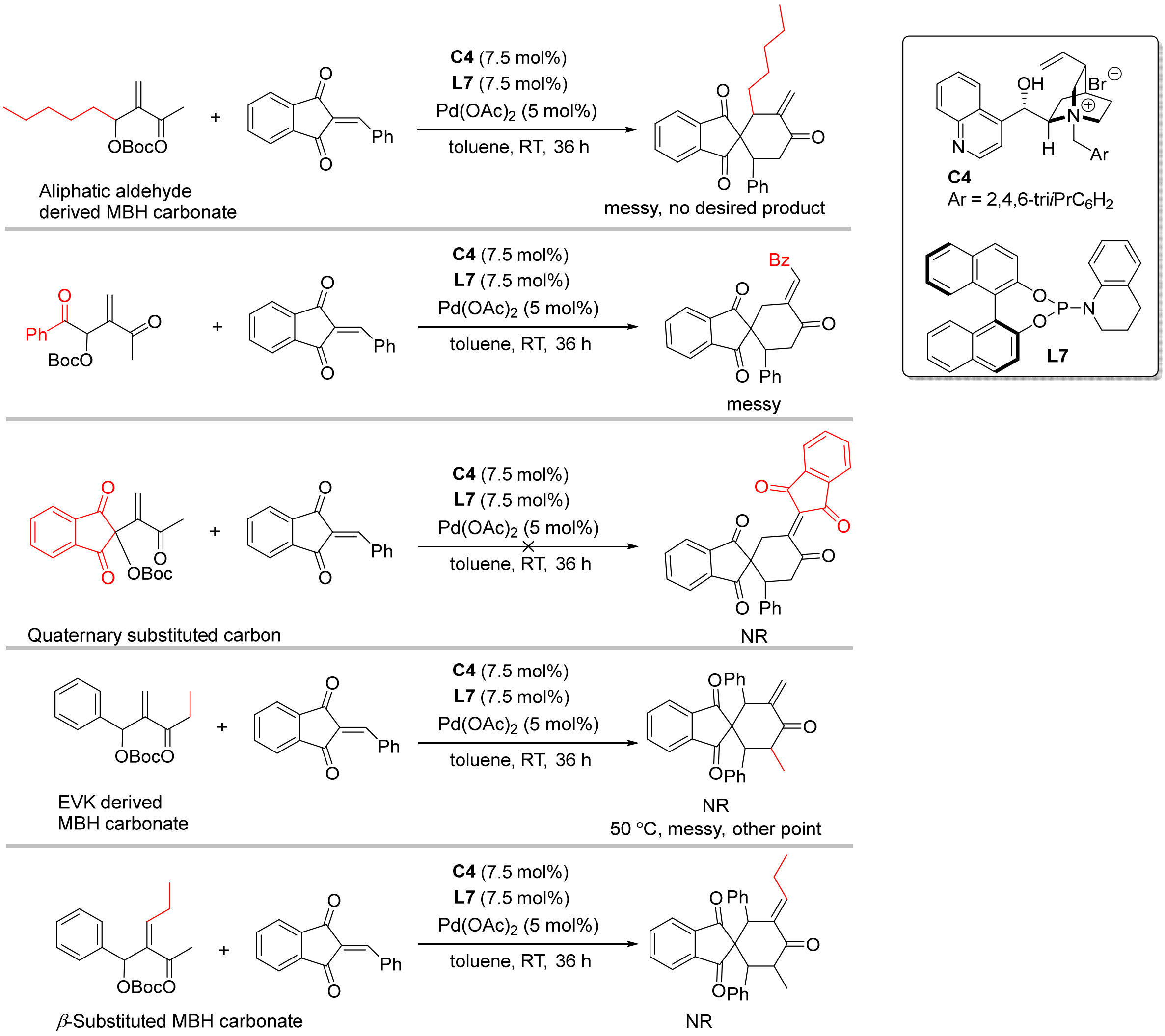

Scheme S10 Unsuccessful attempts with differently substituted MBH carbonates

Unless other noted, the reaction was conducted with MBH carbonate **1** (0.6mmol), enone **2a** (0.05 mmol), $Pd(OAc)_2$ (0.0025 mmol), **C4** (0.005 mmol)and **L7** (0.005 mmol) in dry toluene (0.5 mL) under Ar, and the mixture was stirred at room temperature for 36 h.

8. Bioactivity test

8.1 Biological results

The antiproliferative effects of some products were tested over cisplatin-resistant gastric cancer cell line MGC803 cell line to evaluate their biological activities. Except **11g** and **11h**, other compounds showed high cytotoxicity under 50 μM (Figure **S1**). Next, these products were further evaluated in a dose-dependent manner with IC_{50} values calculated (Table **S6**). Among them, **3q** and **3s** showed the best activities, with IC_{50} values of 4.51 ± 0.09 μM and 5.14 ± 0.19 μM, therefore they were selected as representatives for further investigation. We also evaluated their cytotoxicity towards GES-01, an immortalized gastric epithelial cell line, and found **3s** showed moderate selectivity between cancer cell line and normal cell line (IC_{50} for GES-01 was 12.94 ± 0.58 μM), suggesting **3s** might be a potential lead compound.

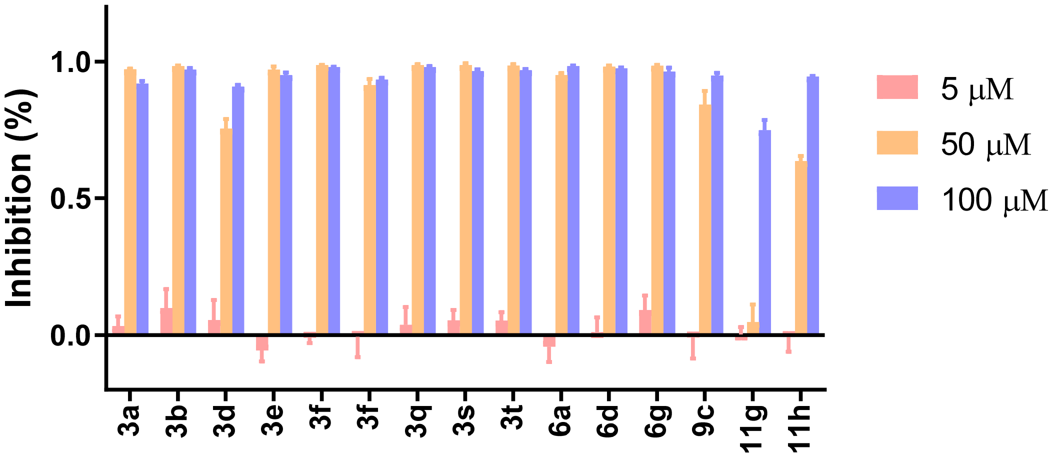

Figure S1 Inhibition rate of some products to the proliferation of MGC803 cells (n = 3)

Table S6 Evaluation of the antiproliferative effects of the indicated products and respective IC_{50} (n = 3)

Entry	Compound	Structure	R[1]	R[2]	IC_{50} (μM)[a]
1	**3a**		Ph	Ph	8.73 ± 0.62
2	**3b**		3-$CH_3C_6H_4$	Ph	12.15 ± 1.22
3	**3d**		4-PhC_6H_4	Ph	42.21 ± 1.80
4	**3e**	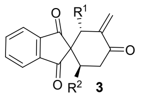	4-FC_6H_4	Ph	21.35 ± 0.55
5	**3f**		3,4-$Cl_2C_6H_3$	Ph	11.61 ± 0.46

6	**3q**		Ph	2-$CH_3C_6H_4$	4.51 ± 0.09[b]
7	**3s**		Ph	4-*t*BuC_6H_4	5.14 ± 0.19[c]
8	**3t**		Ph	3-MeOC_6H_4	10.13 ± 0.23
9	**6a**	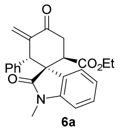 6a			14.58 ± 0.41
10	**6d**	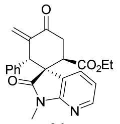 6d			7.81 ± 0.09
11	**6g**	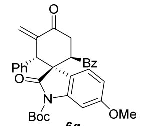 6g			6.25 ± 0.21
12	**9c**	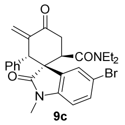 9c			30.91 ± 0.53

[a]IC_{50} was tested based on the proliferation of MGC803 cells.

[b]Cytotoxicity for normal cell was tested based on anti-proliferative experiments over an immortalized gastric epithelial cell line, GES-01. IC_{50} value for 3q was 4.33 ± 0.57 μM.

[c]Cytotoxicity for normal cell was tested based on GES-01 cell line with IC_{50} value of 12.94 ± 0.58 μM.

8.2 Biological evaluation methods

Cell culture

Cis-platin resistant cell line MGC803 was constructed and provided by Department of Gastroenterology, Xinqiao Hospital, Third Military Medical University (Chongqing, China), and was cultured in DMEM (Gbico, USA, C11995599bt) medium containing 10% fetal bovin serum (Lonsera, URY), 100 U/mL penicillin, 100 μg/mL streptomycin. Cells were incubated at 37 °C under 5% CO_2 atmosphere, and was passaged when the cell confluence reached 80%.

Anti-proliferative assay

The anti-proliferative activity of the selected annulation products against MGC803 cell line was evaluated using cell counting kit-8 (CCK-8, Beyotime, China). Cells were plated on 96-well plates at density of 5000 cells/well and cultured overnight. After the cells have reattached the plate, administrate the compounds and incubate for 24 h. Then 10% CCK-8 reagent was added and incubated for 2 h. The absorbance at 450 nm was then read on a Multi-Mode Detection Platform (Spectra Max Paradigm, Molecular Devices, USA) to calculate the cell viability. IC_{50} values were calculated by Graph Pad Prism software.

9. Mechanism study

9.1 Control experiments

Table S7 Evaluation of ammonium halides

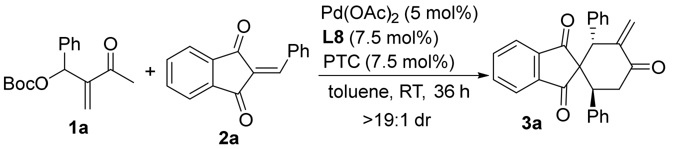

Entry[a]	IPC	yield (%)[b]
1	None	NR
2	TBAC	78
3	TBAB	71
4	TBAI	<10

[a]Unless otherwise noted, the reactions were conducted with **1a** (0.075 mmol), **2a** (0.05 mmol), $Pd(OAc)_2$ (0.0025 mmol), **IPC** (0.005 mmol) and **L8** (0.005 mmol) in dry solvent (0.5 mL) under Ar. [b]Isolated yield. NR = no reaction.

To gain more insight into the catalytic mechanism, several control experiments were conducted. $Pd(OAc)_2$ in combination of achiral phosphoramidite **L8** was utilized for the [4+2] annulation between **1a** and **2a**, and different IPCs were tested. As summarized in Table S7, no reaction occurred without IPC. Remarkably, both TBAB and TBAC significantly enhanced the conversion, but TBAI delivered poor results, demonstrating that halide anion also affected the reaction apparently.

9.2 UV-Vis absorption analysis

Figure S2 The UV-Vis spectra of various catalytic species

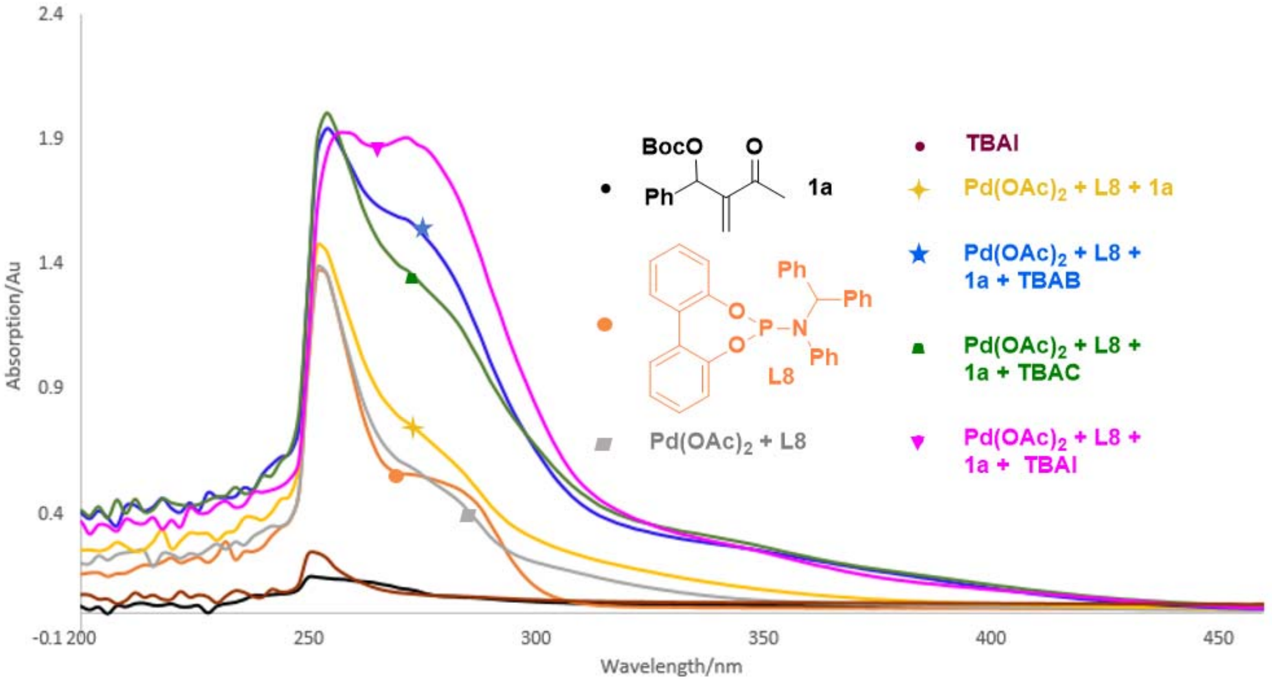

Method of UV-Vis spectrum measurement: To deduct the background UV absorption of the solvent and promote the reaction process smoothly, EtOAc was chosen as the measurement solvent. The 0.0025 mmol reactant (or metal or ligand or IPC, all with 1.0 equiv) was weighted accurately, and dissolved in 0.5 mL dry EtOAc. After stirred at room temperature for 15 min under Ar, a 50 μL solution was diluted to 3.0 mL by adding dry EtOAc. The UV-Vis absorption curve of the reaction solution was obtained accordingly.

To further investigate the effect of IPCs, UV-Vis absorption experiments were carried out. As outlined above, a slightly different absorption spectrum was observed after adding MBH carbonate **1a** into the mixture of $Pd(OAc)_2$ and **L8**, while apparent changes were observed by adding TBAC, TBAB, and TBAI, featuring a stronger absorption at 320–380 nm. These results supported that the previously formed 1,4-carbodipole-type complex would be converted to a new type of species after an ammonium halide was added.

UV-Visible absorption data

Wave-length (nm)	Absorption/Au							
	**1a**	**L8**	$Pd(OAc)_2$ + **L8**	$Pd(OAc)_2$ + **L8** +**1a**	$Pd(OAc)_2$ + **L8** +**1a** +TBAB	$Pd(OAc)_2$ + **L8** +**1a** +TBAC	$Pd(OAc)_2$ + **L8** +**1a** +TBAI	TBAI
600	0.020	0.012	0.004	0.010	0.004	0.015	-0.008	0.035
598	0.020	0.012	0.007	0.010	0.006	0.015	-0.008	0.034
596	0.019	0.012	0.006	0.010	0.005	0.013	-0.008	0.035
594	0.018	0.012	0.007	0.011	0.006	0.015	-0.007	0.035

592	0.015	0.013	0.007	0.012	0.004	0.013	-0.007	0.032
590	0.018	0.011	0.004	0.010	0.006	0.014	-0.007	0.034
588	0.017	0.010	0.007	0.011	0.003	0.015	-0.008	0.036
586	0.020	0.012	0.007	0.009	0.004	0.014	-0.005	0.034
584	0.017	0.012	0.005	0.009	0.005	0.012	-0.007	0.033
582	0.018	0.011	0.006	0.010	0.007	0.014	-0.007	0.035
580	0.018	0.011	0.006	0.010	0.007	0.014	-0.008	0.034
578	0.017	0.012	0.007	0.010	0.005	0.014	-0.007	0.035
576	0.021	0.014	0.007	0.011	0.006	0.015	-0.008	0.035
574	0.019	0.012	0.007	0.011	0.007	0.015	-0.008	0.035
572	0.018	0.012	0.005	0.010	0.006	0.014	-0.008	0.035
570	0.019	0.012	0.007	0.011	0.006	0.017	-0.010	0.036
568	0.018	0.013	0.006	0.010	0.006	0.016	-0.007	0.034
566	0.019	0.011	0.008	0.010	0.006	0.015	-0.010	0.033
564	0.016	0.013	0.008	0.008	0.007	0.016	-0.007	0.035
562	0.020	0.011	0.007	0.012	0.007	0.015	-0.008	0.035
560	0.019	0.013	0.006	0.011	0.008	0.015	-0.008	0.033
558	0.018	0.011	0.006	0.011	0.006	0.015	-0.008	0.035
556	0.020	0.014	0.007	0.011	0.007	0.014	-0.009	0.036
554	0.020	0.013	0.008	0.012	0.006	0.015	-0.008	0.035
552	0.019	0.014	0.007	0.011	0.007	0.016	-0.007	0.037
550	0.018	0.012	0.008	0.012	0.008	0.017	-0.008	0.036
548	0.019	0.013	0.008	0.011	0.006	0.016	-0.008	0.035
546	0.019	0.012	0.007	0.012	0.005	0.016	-0.008	0.034
544	0.019	0.013	0.008	0.011	0.006	0.016	-0.008	0.035
542	0.020	0.013	0.008	0.011	0.006	0.016	-0.008	0.035
540	0.019	0.013	0.008	0.011	0.006	0.017	-0.008	0.034
538	0.020	0.012	0.008	0.011	0.006	0.018	-0.008	0.035
536	0.019	0.013	0.008	0.012	0.007	0.017	-0.007	0.036
534	0.020	0.013	0.008	0.012	0.006	0.017	-0.008	0.035
532	0.020	0.013	0.008	0.011	0.006	0.017	-0.007	0.035
530	0.020	0.013	0.007	0.011	0.007	0.017	-0.007	0.036
528	0.020	0.013	0.008	0.011	0.007	0.018	-0.007	0.036
526	0.020	0.013	0.008	0.012	0.007	0.018	-0.006	0.036
524	0.020	0.014	0.010	0.013	0.007	0.019	-0.007	0.036
522	0.018	0.014	0.008	0.012	0.008	0.018	-0.007	0.037
520	0.020	0.013	0.007	0.012	0.007	0.018	-0.006	0.036
518	0.020	0.013	0.008	0.012	0.008	0.019	-0.005	0.036
516	0.019	0.012	0.009	0.013	0.008	0.018	-0.005	0.035
514	0.020	0.013	0.009	0.011	0.008	0.019	-0.005	0.037
512	0.020	0.013	0.009	0.013	0.008	0.019	-0.005	0.035
510	0.020	0.013	0.008	0.012	0.007	0.019	-0.004	0.037
508	0.020	0.014	0.008	0.011	0.009	0.019	-0.004	0.035
506	0.021	0.013	0.009	0.012	0.008	0.020	-0.003	0.035

504	0.020	0.014	0.008	0.012	0.008	0.019	-0.003	0.035
502	0.020	0.014	0.009	0.012	0.009	0.020	-0.002	0.035
500	0.020	0.014	0.010	0.012	0.008	0.021	-0.002	0.036
498	0.021	0.014	0.009	0.013	0.009	0.021	-0.001	0.037
496	0.021	0.014	0.009	0.013	0.009	0.021	0.000	0.037
494	0.021	0.014	0.010	0.012	0.009	0.022	0.001	0.036
492	0.021	0.014	0.009	0.013	0.009	0.022	0.002	0.036
490	0.021	0.014	0.010	0.013	0.010	0.022	0.002	0.036
488	0.021	0.014	0.009	0.013	0.010	0.023	0.003	0.037
486	0.021	0.014	0.010	0.013	0.010	0.023	0.005	0.037
484	0.021	0.014	0.010	0.013	0.011	0.023	0.005	0.037
482	0.021	0.014	0.010	0.014	0.011	0.024	0.006	0.037
480	0.021	0.014	0.010	0.014	0.012	0.025	0.007	0.037
478	0.021	0.015	0.010	0.014	0.012	0.025	0.008	0.037
476	0.021	0.015	0.011	0.014	0.012	0.025	0.009	0.037
474	0.021	0.015	0.011	0.014	0.013	0.026	0.011	0.037
472	0.022	0.015	0.011	0.015	0.013	0.027	0.011	0.037
470	0.022	0.015	0.011	0.015	0.014	0.027	0.013	0.037
468	0.021	0.015	0.011	0.015	0.014	0.028	0.014	0.037
466	0.022	0.014	0.012	0.015	0.015	0.029	0.015	0.037
464	0.022	0.015	0.012	0.016	0.016	0.030	0.017	0.037
462	0.022	0.015	0.012	0.016	0.017	0.030	0.018	0.038
460	0.022	0.015	0.012	0.016	0.018	0.031	0.020	0.038
458	0.022	0.015	0.012	0.017	0.018	0.032	0.021	0.038
456	0.022	0.015	0.013	0.017	0.019	0.033	0.023	0.037
454	0.022	0.015	0.013	0.017	0.020	0.035	0.025	0.038
452	0.022	0.015	0.013	0.018	0.021	0.036	0.026	0.038
450	0.022	0.016	0.014	0.018	0.022	0.037	0.028	0.038
448	0.022	0.016	0.014	0.018	0.024	0.039	0.030	0.038
446	0.022	0.016	0.014	0.018	0.025	0.040	0.032	0.038
444	0.022	0.016	0.014	0.019	0.027	0.042	0.034	0.038
442	0.023	0.016	0.015	0.020	0.029	0.044	0.036	0.039
440	0.023	0.016	0.015	0.020	0.030	0.045	0.038	0.039
438	0.023	0.016	0.016	0.021	0.032	0.048	0.041	0.039
436	0.023	0.016	0.016	0.021	0.034	0.050	0.043	0.038
434	0.023	0.016	0.016	0.022	0.036	0.052	0.046	0.039
432	0.023	0.016	0.017	0.022	0.039	0.055	0.048	0.039
430	0.023	0.017	0.017	0.023	0.041	0.057	0.050	0.039
428	0.024	0.017	0.018	0.024	0.044	0.060	0.053	0.039
426	0.024	0.017	0.018	0.024	0.047	0.063	0.056	0.039
424	0.024	0.017	0.019	0.025	0.050	0.066	0.059	0.039
422	0.024	0.017	0.019	0.026	0.053	0.069	0.062	0.040
420	0.024	0.017	0.020	0.026	0.056	0.073	0.064	0.040
418	0.024	0.017	0.020	0.027	0.060	0.077	0.068	0.040

416	0.024	0.018	0.021	0.028	0.064	0.081	0.071	0.040
414	0.025	0.018	0.022	0.029	0.068	0.085	0.073	0.040
412	0.025	0.018	0.023	0.030	0.072	0.089	0.077	0.040
410	0.025	0.018	0.023	0.031	0.076	0.093	0.080	0.041
408	0.025	0.018	0.024	0.031	0.081	0.097	0.083	0.041
406	0.025	0.018	0.025	0.032	0.085	0.102	0.086	0.041
404	0.025	0.018	0.025	0.033	0.090	0.107	0.089	0.041
402	0.025	0.019	0.025	0.034	0.095	0.111	0.092	0.041
400	0.026	0.019	0.026	0.035	0.100	0.116	0.095	0.041
398	0.026	0.019	0.026	0.036	0.106	0.121	0.098	0.041
396	0.026	0.019	0.028	0.037	0.111	0.126	0.102	0.041
394	0.026	0.019	0.028	0.038	0.116	0.131	0.105	0.041
392	0.026	0.019	0.029	0.040	0.122	0.136	0.108	0.042
390	0.026	0.019	0.030	0.041	0.127	0.141	0.112	0.042
388	0.026	0.019	0.030	0.042	0.133	0.146	0.116	0.042
386	0.026	0.019	0.031	0.044	0.139	0.151	0.119	0.042
384	0.026	0.019	0.032	0.045	0.144	0.156	0.123	0.042
382	0.027	0.020	0.032	0.046	0.150	0.161	0.128	0.042
380	0.027	0.019	0.033	0.048	0.155	0.166	0.132	0.042
378	0.027	0.020	0.034	0.050	0.161	0.171	0.137	0.042
376	0.027	0.020	0.035	0.052	0.166	0.176	0.142	0.043
374	0.027	0.019	0.036	0.054	0.171	0.181	0.148	0.043
372	0.027	0.020	0.037	0.056	0.177	0.186	0.154	0.043
370	0.028	0.020	0.037	0.059	0.184	0.192	0.161	0.043
368	0.028	0.020	0.039	0.062	0.190	0.199	0.168	0.043
366	0.028	0.020	0.040	0.064	0.196	0.205	0.176	0.043
364	0.028	0.020	0.041	0.067	0.203	0.212	0.185	0.043
362	0.028	0.020	0.042	0.070	0.209	0.219	0.191	0.043
360	0.028	0.020	0.043	0.073	0.215	0.227	0.200	0.044
358	0.029	0.021	0.045	0.077	0.222	0.235	0.209	0.044
356	0.029	0.021	0.046	0.080	0.229	0.243	0.218	0.044
354	0.029	0.021	0.048	0.084	0.236	0.251	0.227	0.044
352	0.030	0.021	0.049	0.088	0.242	0.260	0.236	0.044
350	0.030	0.021	0.052	0.092	0.249	0.268	0.245	0.044
348	0.030	0.021	0.054	0.096	0.255	0.277	0.254	0.044
346	0.031	0.021	0.057	0.102	0.260	0.284	0.262	0.044
344	0.031	0.022	0.059	0.106	0.266	0.291	0.269	0.044
342	0.031	0.022	0.063	0.111	0.271	0.298	0.277	0.044
340	0.032	0.022	0.066	0.117	0.276	0.305	0.284	0.045
338	0.032	0.022	0.070	0.122	0.281	0.312	0.292	0.044
336	0.032	0.022	0.074	0.128	0.287	0.319	0.300	0.045
334	0.033	0.023	0.079	0.134	0.292	0.325	0.309	0.045
332	0.033	0.023	0.083	0.140	0.298	0.331	0.317	0.045
330	0.033	0.024	0.089	0.147	0.305	0.339	0.328	0.045

328	0.034	0.024	0.094	0.153	0.312	0.346	0.338	0.046
326	0.034	0.024	0.100	0.161	0.320	0.355	0.351	0.046
324	0.035	0.024	0.106	0.168	0.329	0.364	0.365	0.047
322	0.035	0.025	0.112	0.175	0.337	0.373	0.378	0.047
320	0.036	0.026	0.119	0.183	0.349	0.386	0.395	0.047
318	0.037	0.027	0.126	0.191	0.360	0.399	0.413	0.048
316	0.037	0.029	0.134	0.199	0.375	0.416	0.434	0.048
314	0.038	0.030	0.142	0.208	0.391	0.434	0.457	0.049
312	0.038	0.034	0.151	0.217	0.412	0.459	0.487	0.049
310	0.039	0.039	0.159	0.228	0.437	0.484	0.521	0.050
308	0.040	0.045	0.168	0.239	0.468	0.514	0.565	0.051
306	0.042	0.054	0.177	0.251	0.506	0.549	0.620	0.052
304	0.043	0.065	0.185	0.264	0.549	0.587	0.684	0.053
302	0.044	0.082	0.195	0.279	0.601	0.629	0.757	0.054
300	0.046	0.106	0.204	0.296	0.655	0.673	0.836	0.056
298	0.048	0.139	0.216	0.314	0.714	0.718	0.922	0.056
296	0.050	0.182	0.230	0.335	0.778	0.765	1.010	0.058
294	0.052	0.234	0.249	0.361	0.848	0.815	1.106	0.060
292	0.055	0.299	0.275	0.394	0.929	0.875	1.213	0.062
290	0.058	0.359	0.307	0.432	1.011	0.934	1.313	0.063
288	0.061	0.419	0.351	0.478	1.106	1.006	1.427	0.065
286	0.063	0.458	0.392	0.521	1.190	1.069	1.527	0.067
284	0.064	0.487	0.434	0.564	1.275	1.135	1.624	0.068
282	0.067	0.506	0.466	0.601	1.347	1.186	1.703	0.069
280	0.068	0.520	0.492	0.634	1.407	1.228	1.765	0.070
278	0.071	0.532	0.516	0.667	1.459	1.265	1.821	0.072
276	0.078	0.542	0.541	0.699	1.502	1.300	1.862	0.074
274	0.090	0.552	0.569	0.731	1.544	1.338	1.880	0.077
272	0.097	0.556	0.591	0.760	1.576	1.373	1.907	0.080
270	0.102	0.558	0.610	0.785	1.588	1.395	1.900	0.083
268	0.112	0.575	0.645	0.825	1.605	1.435	1.883	0.089
266	0.118	0.613	0.692	0.871	1.625	1.479	1.873	0.096
264	0.126	0.688	0.761	0.938	1.655	1.545	1.873	0.107
262	0.129	0.794	0.852	1.020	1.700	1.628	1.887	0.121
260	0.132	0.936	0.973	1.126	1.770	1.738	1.921	0.137
258	0.138	1.085	1.102	1.235	1.842	1.844	1.928	0.157
256	0.139	1.238	1.238	1.349	1.909	1.949	1.918	0.183
254	0.143	1.360	1.363	1.452	1.942	2.005	1.832	0.222
252	0.147	1.364	1.382	1.471	1.843	1.896	1.615	0.241
250	0.146	0.974	0.991	1.072	1.302	1.313	1.051	0.236
248	0.097	0.476	0.477	0.588	0.737	0.799	0.605	0.118
246	0.081	0.369	0.374	0.468	0.640	0.652	0.538	0.090
244	0.078	0.342	0.348	0.454	0.626	0.611	0.508	0.086
242	0.075	0.308	0.349	0.402	0.595	0.552	0.494	0.097

240	0.071	0.264	0.329	0.372	0.557	0.552	0.485	0.065
238	0.072	0.246	0.308	0.382	0.496	0.494	0.485	0.068
236	0.055	0.246	0.273	0.380	0.504	0.481	0.435	0.085
234	0.043	0.214	0.286	0.329	0.467	0.507	0.424	0.091
232	0.038	0.286	0.256	0.320	0.464	0.473	0.449	0.070
230	0.028	0.231	0.278	0.290	0.465	0.469	0.435	0.043
228	-0.001	0.202	0.256	0.317	0.483	0.454	0.403	0.067
226	0.030	0.223	0.234	0.316	0.410	0.438	0.401	0.075
224	0.025	0.214	0.239	0.319	0.442	0.431	0.423	0.065
222	0.025	0.201	0.220	0.287	0.404	0.436	0.345	0.089
220	0.037	0.221	0.236	0.255	0.420	0.444	0.373	0.057
218	0.031	0.204	0.240	0.331	0.460	0.460	0.337	0.057
216	0.031	0.191	0.187	0.285	0.426	0.418	0.384	0.074
214	0.040	0.193	0.251	0.252	0.410	0.407	0.359	0.065
212	0.038	0.178	0.248	0.245	0.432	0.459	0.362	0.083
210	0.025	0.160	0.243	0.265	0.431	0.401	0.378	0.053
208	0.030	0.158	0.235	0.282	0.349	0.456	0.329	0.040
206	-0.004	0.147	0.213	0.256	0.382	0.383	0.351	0.058
204	0.024	0.162	0.231	0.247	0.410	0.429	0.355	0.058
202	0.012	0.161	0.198	0.252	0.378	0.391	0.317	0.074
200	0.027	0.156	0.200	0.252	0.406	0.414	0.368	0.075
198	0.032	0.163	0.206	0.271	0.436	0.381	0.317	0.058
196	0.031	0.156	0.241	0.249	0.392	0.378	0.324	0.055
194	0.020	0.168	0.213	0.229	0.339	0.355	0.292	0.062
192	0.015	0.130	0.220	0.211	0.328	0.370	0.321	0.078
190	0.036	0.155	0.199	0.251	0.355	0.349	0.326	0.070

We have monitored the UV absorption of the mixture of $Pd(OAc)_2$ + **L8** with/without TBAB or **1a**. The results indicated that the sole addition of TBAB or **1a** did slightly change the UV absorption, while the addition of both of them significantly changed UV spectra. The control experiments have been carried out with other ammonium salts, and similar results were obtained.

Figure S3 Comparison of UV-Visible Spectra with/without TBAB

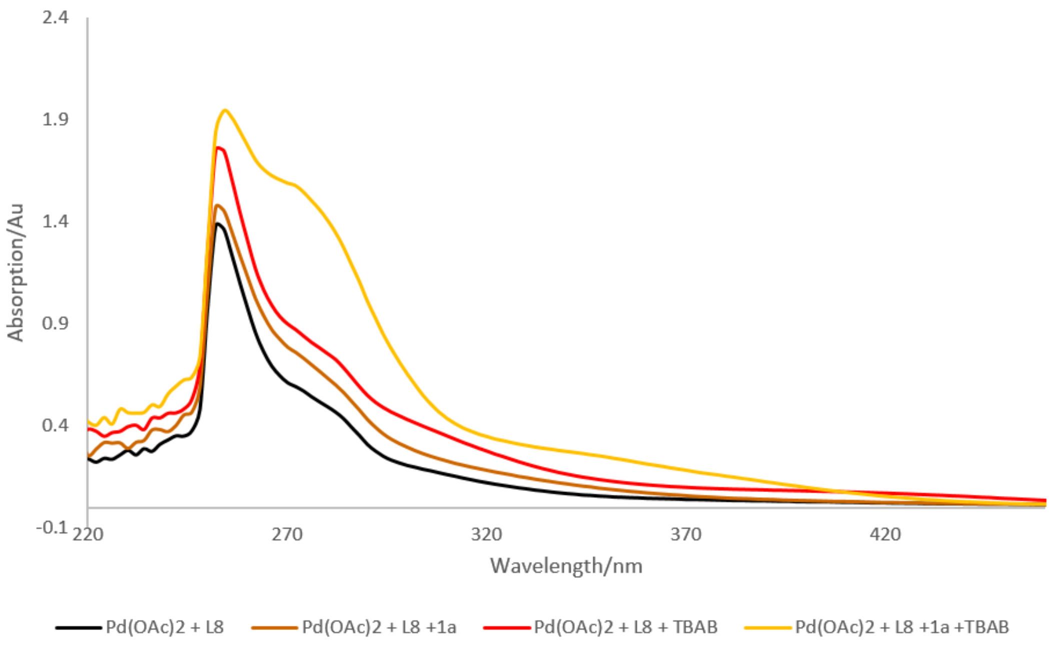

UV-Visible absorption data

Wave length (nm)	Absorption/Au			
	$Pd(OAc)_2$ + **L8**	$Pd(OAc)_2$ + **L8** +**1a**	$Pd(OAc)_2$ + **L8** + TBAB	$Pd(OAc)_2$ + **L8** +**1a** +TBAB
600	0.004	0.010	−0.002	0.004
598	0.007	0.010	−0.004	0.006
596	0.006	0.010	−0.004	0.005
594	0.007	0.011	−0.003	0.006
592	0.007	0.012	−0.004	0.004
590	0.004	0.010	−0.005	0.006
588	0.007	0.011	−0.002	0.003
586	0.007	0.009	−0.002	0.004
584	0.005	0.009	−0.003	0.005
582	0.006	0.010	−0.001	0.007
580	0.006	0.010	−0.003	0.007
578	0.007	0.010	−0.002	0.005
576	0.007	0.011	−0.001	0.006
574	0.007	0.011	−0.003	0.007
572	0.005	0.010	−0.001	0.006
570	0.007	0.011	−0.001	0.006

568	0.006	0.010	0	0.006
566	0.008	0.010	–0.001	0.006
564	0.008	0.008	–0.001	0.007
562	0.007	0.012	0	0.007
560	0.006	0.011	–0.001	0.008
558	0.006	0.011	0	0.006
556	0.007	0.011	0.001	0.007
554	0.008	0.012	0.001	0.006
552	0.007	0.011	0.001	0.007
550	0.008	0.012	0.001	0.008
548	0.008	0.011	0.001	0.006
546	0.007	0.012	0.002	0.005
544	0.008	0.011	0.002	0.006
542	0.008	0.011	0.003	0.006
540	0.008	0.011	0.003	0.006
538	0.008	0.011	0.002	0.006
536	0.008	0.012	0.002	0.007
534	0.008	0.012	0.003	0.006
532	0.008	0.011	0.004	0.006
530	0.007	0.011	0.004	0.007
528	0.008	0.011	0.004	0.007
526	0.008	0.012	0.005	0.007
524	0.010	0.013	0.005	0.007
522	0.008	0.012	0.007	0.008
520	0.007	0.012	0.006	0.007
518	0.008	0.012	0.008	0.008
516	0.009	0.013	0.007	0.008
514	0.009	0.011	0.007	0.008
512	0.009	0.013	0.007	0.008
510	0.008	0.012	0.008	0.007
508	0.008	0.011	0.009	0.009
506	0.009	0.012	0.011	0.008
504	0.008	0.012	0.009	0.008
502	0.009	0.012	0.010	0.009
500	0.010	0.012	0.011	0.008
498	0.009	0.013	0.011	0.009
496	0.009	0.013	0.012	0.009
494	0.010	0.012	0.013	0.009
492	0.009	0.013	0.014	0.009
490	0.010	0.013	0.015	0.010
488	0.009	0.013	0.016	0.010
486	0.010	0.013	0.017	0.010
484	0.010	0.013	0.017	0.011
482	0.010	0.014	0.019	0.011

480	0.010	0.014	0.020	0.012
478	0.010	0.014	0.021	0.012
476	0.011	0.014	0.022	0.012
474	0.011	0.014	0.024	0.013
472	0.011	0.015	0.025	0.013
470	0.011	0.015	0.026	0.014
468	0.011	0.015	0.028	0.014
466	0.012	0.015	0.029	0.015
464	0.012	0.016	0.031	0.016
462	0.012	0.016	0.033	0.017
460	0.012	0.016	0.034	0.018
458	0.012	0.017	0.036	0.018
456	0.013	0.017	0.039	0.019
454	0.013	0.017	0.040	0.020
452	0.013	0.018	0.042	0.021
450	0.014	0.018	0.044	0.022
448	0.014	0.018	0.046	0.024
446	0.014	0.018	0.048	0.025
444	0.014	0.019	0.050	0.027
442	0.015	0.020	0.052	0.029
440	0.015	0.020	0.054	0.030
438	0.016	0.021	0.056	0.032
436	0.016	0.021	0.057	0.034
434	0.016	0.022	0.059	0.036
432	0.017	0.022	0.061	0.039
430	0.017	0.023	0.062	0.041
428	0.018	0.024	0.064	0.044
426	0.018	0.024	0.066	0.047
424	0.019	0.025	0.067	0.050
422	0.019	0.026	0.068	0.053
420	0.020	0.026	0.070	0.056
418	0.020	0.027	0.071	0.060
416	0.021	0.028	0.072	0.064
414	0.022	0.029	0.073	0.068
412	0.023	0.030	0.075	0.072
410	0.023	0.031	0.076	0.076
408	0.024	0.031	0.077	0.081
406	0.025	0.032	0.078	0.085
404	0.025	0.033	0.079	0.090
402	0.025	0.034	0.080	0.095
400	0.026	0.035	0.081	0.100
398	0.026	0.036	0.082	0.106
396	0.028	0.037	0.083	0.111
394	0.028	0.038	0.084	0.116

392	0.029	0.040	0.084	0.122
390	0.030	0.041	0.086	0.127
388	0.030	0.042	0.087	0.133
386	0.031	0.044	0.087	0.139
384	0.032	0.045	0.089	0.144
382	0.032	0.046	0.089	0.150
380	0.033	0.048	0.091	0.155
378	0.034	0.050	0.092	0.161
376	0.035	0.052	0.093	0.166
374	0.036	0.054	0.095	0.171
372	0.037	0.056	0.096	0.177
370	0.037	0.059	0.098	0.184
368	0.039	0.062	0.100	0.190
366	0.040	0.064	0.102	0.196
364	0.041	0.067	0.105	0.203
362	0.042	0.070	0.108	0.209
360	0.043	0.073	0.111	0.215
358	0.045	0.077	0.114	0.222
356	0.046	0.080	0.118	0.229
354	0.048	0.084	0.122	0.236
352	0.049	0.088	0.126	0.242
350	0.052	0.092	0.132	0.249
348	0.054	0.096	0.137	0.255
346	0.057	0.102	0.143	0.260
344	0.059	0.106	0.148	0.266
342	0.063	0.111	0.155	0.271
340	0.066	0.117	0.163	0.276
338	0.070	0.122	0.171	0.281
336	0.074	0.128	0.181	0.287
334	0.079	0.134	0.190	0.292
332	0.083	0.140	0.200	0.298
330	0.089	0.147	0.212	0.305
328	0.094	0.153	0.223	0.312
326	0.100	0.161	0.236	0.320
324	0.106	0.168	0.249	0.329
322	0.112	0.175	0.261	0.337
320	0.119	0.183	0.276	0.349
318	0.126	0.191	0.289	0.360
316	0.134	0.199	0.304	0.375
314	0.142	0.208	0.319	0.391
312	0.151	0.217	0.334	0.412
310	0.159	0.228	0.350	0.437
308	0.168	0.239	0.366	0.468
306	0.177	0.251	0.382	0.506

304	0.185	0.264	0.398	0.549
302	0.195	0.279	0.414	0.601
300	0.204	0.296	0.431	0.655
298	0.216	0.314	0.449	0.714
296	0.230	0.335	0.467	0.778
294	0.249	0.361	0.489	0.848
292	0.275	0.394	0.516	0.929
290	0.307	0.432	0.549	1.011
288	0.351	0.478	0.592	1.106
286	0.392	0.521	0.639	1.190
284	0.434	0.564	0.685	1.275
282	0.466	0.601	0.725	1.347
280	0.492	0.634	0.754	1.407
278	0.516	0.667	0.782	1.459
276	0.541	0.699	0.809	1.502
274	0.569	0.731	0.840	1.544
272	0.591	0.760	0.872	1.576
270	0.610	0.785	0.899	1.588
268	0.645	0.825	0.936	1.605
266	0.692	0.871	0.991	1.625
264	0.761	0.938	1.065	1.655
262	0.852	1.020	1.165	1.700
260	0.973	1.126	1.304	1.770
258	1.102	1.235	1.449	1.842
256	1.238	1.349	1.608	1.909
254	1.363	1.452	1.748	1.942
252	1.382	1.471	1.753	1.843
250	0.991	1.072	1.273	1.302
248	0.477	0.588	0.692	0.737
246	0.374	0.468	0.530	0.640
244	0.348	0.454	0.482	0.626
242	0.349	0.402	0.463	0.595
240	0.329	0.372	0.461	0.557
238	0.308	0.382	0.439	0.496
236	0.273	0.380	0.438	0.504
234	0.286	0.329	0.382	0.467
232	0.256	0.320	0.403	0.464
230	0.278	0.290	0.396	0.465
228	0.256	0.317	0.373	0.483
226	0.234	0.316	0.366	0.410
224	0.239	0.319	0.348	0.442
222	0.220	0.287	0.373	0.404
220	0.236	0.255	0.383	0.420
218	0.240	0.331	0.353	0.460

216	0.187	0.285	0.368	0.426
214	0.251	0.252	0.317	0.410
212	0.248	0.245	0.313	0.432
210	0.243	0.265	0.342	0.431
208	0.235	0.282	0.361	0.349
206	0.213	0.256	0.316	0.382
204	0.231	0.247	0.334	0.410
202	0.198	0.252	0.341	0.378
200	0.200	0.252	0.294	0.406
198	0.206	0.271	0.312	0.436
196	0.241	0.249	0.318	0.392
194	0.213	0.229	0.324	0.339
192	0.220	0.211	0.291	0.328
190	0.199	0.251	0.320	0.355

Table S8 Evaluation of the Pd-BINAP catalytic system

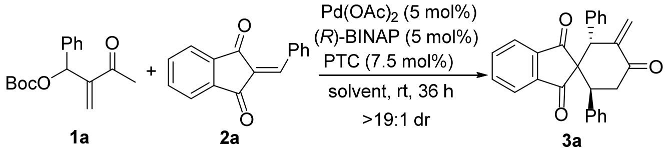

Entry[a]	IPC	Solvent	Yield (%)[b]	ee (%)[c]
1	None	Toluene	86	–3
2	TBAB	Toluene	trace	/
3	TBAB	EtOAc	trace	/

[a]Unless otherwise noted, the reactions were conducted with **1a** (0.075 mmol), **2a** (0.05 mmol), $Pd(OAc)_2$ (0.0025 mmol), TBAB (0.005 mmol) and **L8** (0.005 mmol) in dry solvent (0.5 mL) under Ar. [b]Isolated yield. [c]Determined by chiral HPLC analysis on a chiral stationary phase; >19:1 dr.

Figure S4 The UV-Vis spectra of catalytic species involving (*R*)-BINAP

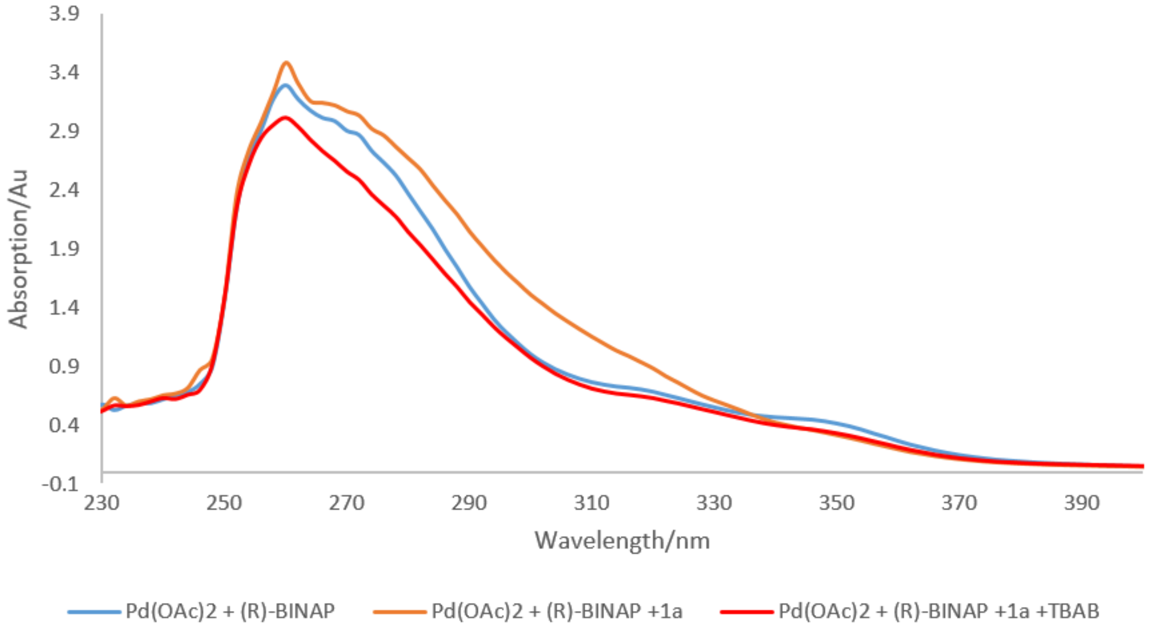

Method of UV-Vis spectrum measurement: To deduct the background UV absorption of the solvent and promote the reaction process smoothly, EtOAc was chosen as the measurement solvent. The 0.0025 mmol reactant (or metal or ligand or IPC, with 1.0 equiv) was weighted accurately, and dissolved in 0.5 mL dry EtOAc. After stirred at room temperature for 15 min under Ar, a 50 μL solution was diluted to 3.0 mL by adding dry EtOAc. The UV-Vis absorption curve of the reaction solution was obtained accordingly.

It was found that the combination of $Pd(OAc)_2$-BINAP could well promote the reaction of **1a** and **2a**, but adding TBAB resulted in no reaction. The UV-Vis absorption experiments with $Pd(OAc)_2$ and BINAP with/without TBAB were also conducted. As outlined above, a different absorption spectrum was also observed after adding TBAB, but with apparently decreased absorption potency. Although

we have no reasonable elucidation for the apparent changes of the catalytic activity at the current stage, the structure of the previously formed Pd-BINAP-allyl complex of **1a** would also be converted to a new intermediate after adding TBAB, probably even involving the diassociation of the *π*-allylpalladium complex (based on the decreased absorption potency). In fact, we could observe the consumption of MBH carbonate **1a** when stoichiometric TBAB was used, probably via the substitution of Pd-BINAP-allyl complex of **1a** by nucleophilic species (such as Br^- or others) in the reaction.

UV-Visible absorption data

Wavelength(nm)	Absorption/Au		
	$Pd(OAc)_2$ + (*R*)-BINAP	$Pd(OAc)_2$ + (*R*)-BINAP +**1a**	$Pd(OAc)_2$ + (*R*)-BINAP +**1a** +TBAB
600	-0.009	0.011	-0.002
598	-0.010	0.013	0
596	-0.011	0.010	-0.001
594	-0.011	0.010	0
592	-0.010	0.010	0
590	-0.012	0.011	0
588	-0.010	0.011	-0.001
586	-0.012	0.011	-0.002
584	-0.008	0.010	-0.004
582	-0.012	0.011	-0.001
580	-0.012	0.011	0
578	-0.011	0.011	-0.002
576	-0.012	0.011	-0.003
574	-0.012	0.010	-0.001
572	-0.011	0.010	-0.002
570	-0.014	0.011	-0.002
568	-0.012	0.008	-0.002
566	-0.013	0.009	-0.001
564	-0.012	0.009	-0.004
562	-0.013	0.010	-0.003
560	-0.013	0.012	-0.002
558	-0.014	0.009	-0.004
556	-0.014	0.009	-0.003
554	-0.014	0.010	-0.005
552	-0.015	0.010	-0.006
550	-0.015	0.009	-0.003

548	-0.015	0.009	-0.003
546	-0.015	0.009	-0.004
544	-0.015	0.009	-0.004
542	-0.015	0.008	-0.004
540	-0.017	0.008	-0.005
538	-0.016	0.008	-0.004
536	-0.016	0.007	-0.005
534	-0.016	0.007	-0.005
532	-0.016	0.007	-0.005
530	-0.016	0.007	-0.005
528	-0.017	0.007	-0.005
526	-0.017	0.006	-0.006
524	-0.017	0.006	-0.006
522	-0.017	0.005	-0.005
520	-0.019	0.006	-0.006
518	-0.018	0.006	-0.006
516	-0.018	0.005	-0.006
514	-0.019	0.006	-0.007
512	-0.019	0.006	-0.007
510	-0.019	0.004	-0.005
508	-0.019	0.004	-0.006
506	-0.019	0.005	-0.006
504	-0.019	0.004	-0.007
502	-0.020	0.005	-0.006
500	-0.020	0.004	-0.006
498	-0.020	0.004	-0.006
496	-0.020	0.004	-0.006
494	-0.020	0.004	-0.006
492	-0.020	0.004	-0.005
490	-0.020	0.003	-0.005
488	-0.020	0.003	-0.005
486	-0.020	0.003	-0.004
484	-0.021	0.003	-0.004
482	-0.021	0.003	-0.004
480	-0.021	0.003	-0.003
478	-0.02	0.003	-0.003
476	-0.020	0.003	-0.002
474	-0.020	0.003	-0.002
472	-0.020	0.003	-0.001
470	-0.020	0.003	0
468	-0.020	0.003	0
466	-0.020	0.003	0.001

464	-0.019	0.003	0.002
462	-0.019	0.004	0.002
460	-0.019	0.004	0.003
458	-0.018	0.004	0.004
456	-0.017	0.004	0.005
454	-0.016	0.005	0.006
452	-0.016	0.005	0.007
450	-0.015	0.006	0.008
448	-0.014	0.006	0.009
446	-0.013	0.007	0.010
444	-0.011	0.008	0.011
442	-0.010	0.009	0.012
440	-0.009	0.010	0.014
438	-0.007	0.011	0.015
436	-0.005	0.012	0.017
434	-0.003	0.013	0.018
432	-0.001	0.014	0.020
430	0.001	0.016	0.021
428	0.004	0.017	0.023
426	0.006	0.019	0.025
424	0.008	0.021	0.027
422	0.011	0.022	0.029
420	0.013	0.024	0.031
418	0.017	0.026	0.033
416	0.020	0.028	0.035
414	0.023	0.030	0.038
412	0.026	0.033	0.041
410	0.029	0.034	0.042
408	0.032	0.037	0.045
406	0.036	0.039	0.047
404	0.039	0.041	0.049
402	0.042	0.043	0.051
400	0.046	0.045	0.053
398	0.049	0.047	0.055
396	0.052	0.050	0.057
394	0.056	0.051	0.059
392	0.059	0.053	0.061
390	0.063	0.056	0.063
388	0.067	0.058	0.066
386	0.071	0.061	0.068
384	0.076	0.064	0.071
382	0.082	0.068	0.074

380	0.089	0.072	0.079
378	0.096	0.077	0.084
376	0.104	0.083	0.090
374	0.115	0.090	0.098
372	0.128	0.099	0.108
370	0.143	0.109	0.119
368	0.161	0.121	0.133
366	0.182	0.135	0.149
364	0.207	0.153	0.169
362	0.232	0.170	0.189
360	0.263	0.193	0.212
358	0.296	0.217	0.238
356	0.327	0.241	0.262
354	0.360	0.267	0.287
352	0.389	0.291	0.310
350	0.412	0.314	0.330
348	0.431	0.336	0.348
346	0.445	0.357	0.363
344	0.452	0.375	0.374
342	0.459	0.397	0.386
340	0.465	0.420	0.400
338	0.474	0.451	0.417
336	0.486	0.489	0.438
334	0.504	0.531	0.462
332	0.525	0.570	0.486
330	0.550	0.608	0.511
328	0.575	0.650	0.535
326	0.602	0.704	0.560
324	0.630	0.762	0.585
322	0.656	0.817	0.606
320	0.683	0.882	0.629
318	0.703	0.935	0.645
316	0.717	0.987	0.657
314	0.727	1.033	0.668
312	0.743	1.091	0.686
310	0.764	1.151	0.710
308	0.793	1.215	0.744
306	0.830	1.282	0.786
304	0.875	1.351	0.837
302	0.932	1.430	0.900
300	1.002	1.509	0.971
298	1.091	1.605	1.055

296	1.189	1.700	1.140
294	1.300	1.807	1.234
292	1.438	1.926	1.342
290	1.577	2.048	1.447
288	1.742	2.192	1.573
286	1.899	2.313	1.686
284	2.069	2.441	1.809
282	2.219	2.575	1.929
280	2.371	2.672	2.041
278	2.525	2.768	2.170
276	2.636	2.866	2.266
274	2.738	2.924	2.362
272	2.871	3.035	2.483
270	2.907	3.071	2.554
268	2.990	3.122	2.646
266	3.018	3.143	2.729
264	3.081	3.159	2.826
262	3.178	3.311	2.937
260	3.295	3.485	3.013
258	3.180	3.235	2.952
256	2.914	2.979	2.840
254	2.687	2.741	2.617
252	2.244	2.362	2.247
250	1.458	1.483	1.475
248	0.894	0.962	0.912
246	0.749	0.869	0.699
244	0.679	0.715	0.658
242	0.652	0.668	0.622
240	0.619	0.653	0.630
238	0.586	0.619	0.601
236	0.585	0.601	0.571
234	0.564	0.566	0.561
232	0.527	0.629	0.567
230	0.574	0.532	0.520
228	0.493	0.523	0.489
226	0.484	0.527	0.510
224	0.496	0.523	0.494
222	0.450	0.538	0.507
220	0.485	0.469	0.466
218	0.517	0.494	0.456
216	0.474	0.493	0.465
214	0.432	0.483	0.450

212	0.475	0.495	0.462
210	0.426	0.451	0.445
208	0.469	0.501	0.402
206	0.436	0.525	0.481
204	0.418	0.482	0.460
202	0.426	0.460	0.444
200	0.473	0.402	0.441
198	0.372	0.443	0.378
196	0.363	0.470	0.374
194	0.331	0.460	0.414
192	0.407	0.424	0.439
190	0.416	0.430	0.386

10. DFT calculations

10.1 Coordination model

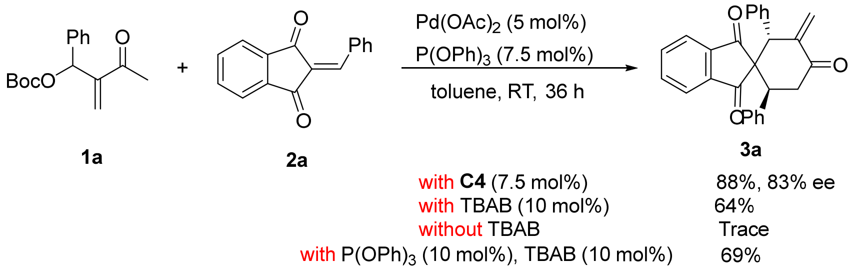

Scheme S11 Control experiments using $Pd(OAc)_2$ and $P(OPh)_3$

To gain more insight into the mechanism, control experiments using $Pd(OAc)_2$ and $P(OPh)_3$ was first carried out. As outlined in Scheme **S11**, similar results were obtained in the presence or absence of TBAB. Therefore, $P(OPh)_3$ could be used as a simpler ligand for calculations. To explain the role of TBAB, DFT calculations were carried out at the B3LYP-D3/6-31(d) // B3LYP-D3/6-311++G(d,p) SDD for Pd (toluene) level.

Firstly, the conformation of the complex model of the intermediates **(INT1**) formed by **1a** and Pd(0) was calculated, as shown in Figure **S5**. We tried to use molecular mechanics based software such as SYBYL 2.0X and Amber 2.0 to initially screen these conformations, while the results were not consistent with the energy calculated by DFT, because energy of coordinated bond formed by metal atom Pd might be not able to be correctly evaluated by molecular mechanics. Thus, the structures and energies of conformations were calculated by DFT. For both models with Pd coordinating with one $P(OPh)_3$ ligand and two ligands, the conformations employing two Pd-C σ bonds (**INT1-B** and **DL-**

INT1-B) had the lower energies. On the other side, the formation of charge separation model proposed as the precursor for addition, in which Pd was coordinated with π-allyl by η^3-model, was quit hard, since the lowest energies among charge separation models for **INT1-D′** was 23.1 kcal/mol higher than that of **INT1-B**. Similarly, **DL-INT1-C′** was 18.4 kcal/mol higher than that of **DL-INT1-B.**

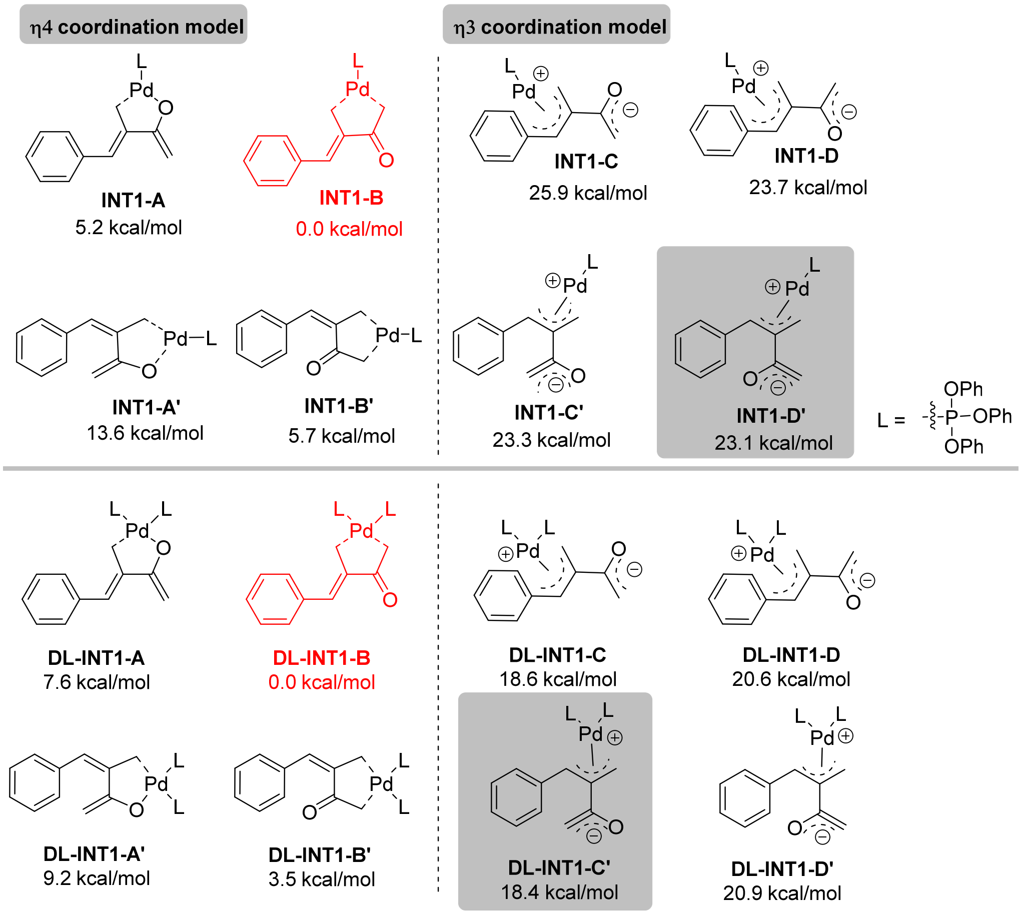

Figure S5 The structures and energies of the conformations for the complexes in which Pd is coordinating with one/two ligands at the B3LYP-D3/6-31(d)// B3LYP-D3/6-311++G(d,p) SDD for Pd (toluene) level. The energies are given in kcal/mol relative to energy of **INT1-B/DL-INT1-B**

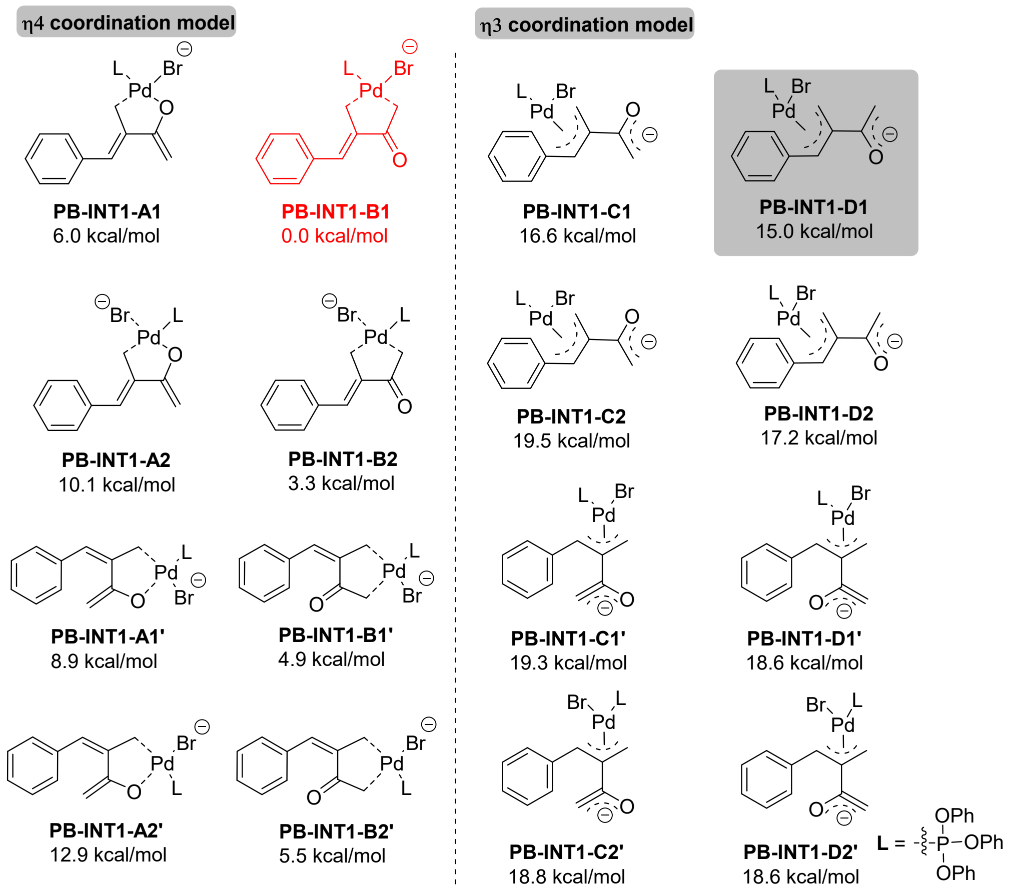

Figure S6 The structures and energies of the conformations for the complexes in which Pd is coordinating with $P(OPh)_3$ and bromide anion at the B3LYP-D3/6-31(d)// B3LYP-D3/6-311++G(d,p) SDD for Pd (toluene) level. The energies are given in kcal/mol relative to energy of **PB-INT1-B1**

When TMAB was added (in order to simplify the calculation work, tetramethylammonium bromide, TMAB, was used instead of very flexible TBAB), the coordination model might be different and more complex. To generate the energy difference between stable conformation and charge separation model, we firstly calculated the conformations of **PB-INT1** in which Pd was coordinated with $P(OPh)_3$ and bromide anion. The energies of **PB-INT1-B1** and **PB-INT1-D1** were the lowest among the η^4-model and η^3-model, respectively (Figure **S6**). The energy difference of them was 15.0 kcal/mol. Because the ammonium motif may locate at different positions, the computation work is too large to calculate each pose for different conformations with ammonium salt. Thus, to evaluate the coordination model with TMAB, **PB-INT1-B1** and **PB-INT1-D1** were selected as the representative conformations for η^4-model and η^3-model, respectively. More than 6 poses started form **PB-INT1-B1** and **PB-INT1-D1** were calculated and representative conformations were shown in Figure **S7**. The energies of **INT1-B1-TMA1** and **INT1-D1-TMA1** were the lowest among the η^4-

model and η^3-model, respectively, and the energy of **INT1-D1-TMA1** was 10.6 kcal/mol higher than that of **INT1-B1-TMA1**. To conform the results of this simplified model, the lowest energy among the poses started form **INT1-C1 (INT1-C1-TMA1)** was also calculated and it is higher than that of **INT1-D1-TMA1**, suggesting generating the pose of η^3-model **PB-INT1** and tetramethylammonium with the lowest energy from **PB-INT1-D1** might be reasonable. Similarly, the energy of **INT-B2-TMA1** was 1.7 kcal/mol higher than that of **INT-B1-TMA1**.

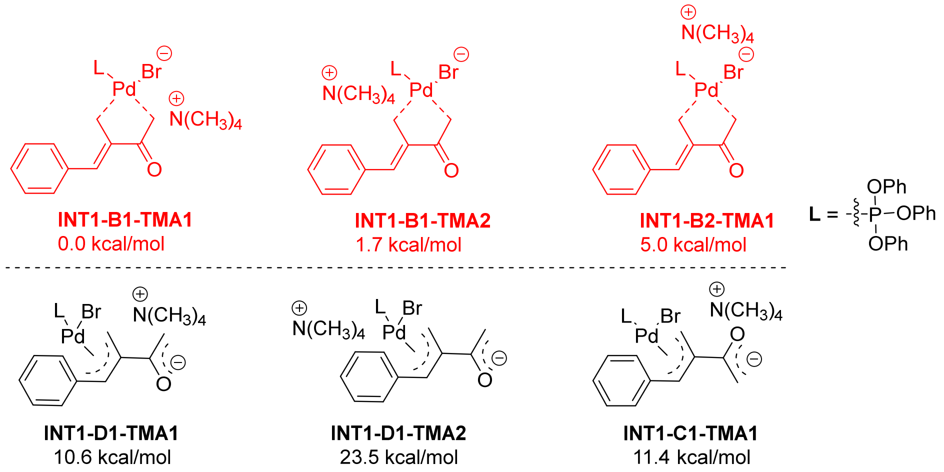

Figure S7 The structures and energies of the conformations for the complexes **PB-INT1** at the B3LYP-D3/6-31(d)// B3LYP-D3/6-311++G(d,p) SDD for Pd (toluene) level. The energies are given in kcal/mol relative to energy of **PB-INT1-B1-TMA1**

10.2 Reaction process

To rationalize the mechanism of this reaction, the reaction process was calculated as shown in Figure **S8**. The formation of charge separation model was proposed as the precursor for addition. After the formation of **INT1-D1-TMA1** from the stable conformation **INT1-B1-TMA1**, the addition of carbanion to **2a** would lead to **INT2** via **TS1** with a value of 20.4 kcal/mol. The resulting **INT2** would be easily converted to the ion-pairing intermediate **INT3**, which would undergo allylic substitution via **TS2** with an energy barrier of 8.8 kcal/mol, finally affording the intermediate **INT4**. We also compared the possible formation of regioisomer via **TS2′** from ion-pair **INT3′**, which had a higher energy barrier of 10.6 kcal/mol, also in good accordance to a predicted ratio (21:1) (**pathway B**).

In contrary, the energy barrier of the rate-limiting addition step for the reaction without ammonium salt was quit high. The energy of **DL-TS1** was much higher than that of **TS1** (**pathway A**), suggesting

the addition might not be promoted in the absence of TMAB, which was consistent with our experiments.

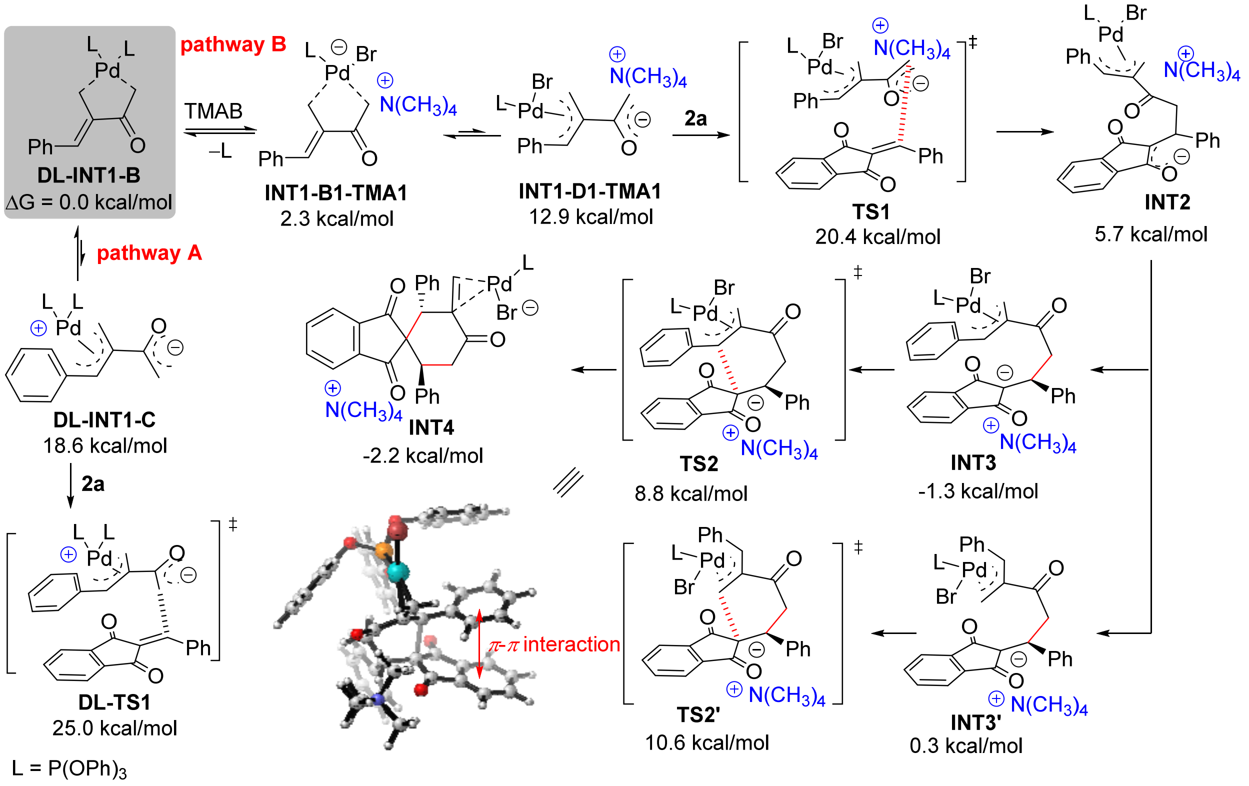

Figure S8 Computed potential energy surface of the reaction of **1a** and **2a** with TMAB at the B3LYP-D3/6-31(d)// B3LYP-D3/6-311++G(d,p) SDD for Pd (toluene) level and is given in kcal/mol relative to energy sum of **PB-INT1-B1-TMA1** and **2a**

The optimized structures of **INT3** and **TS2** were also outlined in Figure **S9**. Structure analysis showed that in intermediate **INT3** the dihedral angles of C1-C2-C3-C4 and O-C5C6-C7 were 159° and -3.9°, respectively, while the corresponding dihedral angles in **TS2** were determined to be 135.0° and 32.0°, respectively. These results clearly exhibited the sp^3 hybridization trend of the C3 and C6 centers in C-C bond formation. Moreover, the intrinsic reaction coordinate analysis (IRC) of **TS2** also proved this C-C bond formation (see below for details).

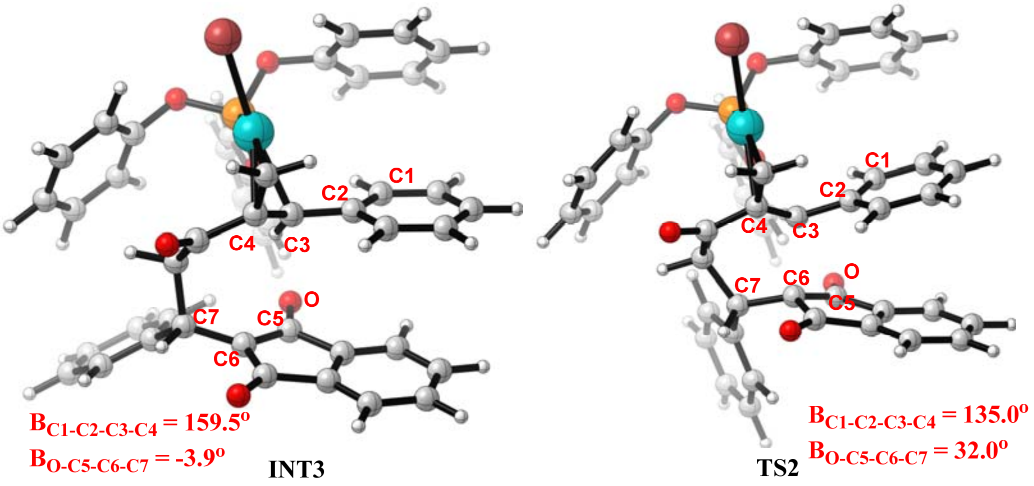

Figure S9 The optimized structures of **INT3** and **TS2**

Theoretically, to explain the role of TMAB, more rigorous calculations should be carried out. We have tried but the calculation work was too large to finish. Thus, a simplified model was used. We confirmed the role of TMAB in reducing the energy difference of η^4-model and η^3-model, which further led to the reduced energy barrier of the rate-limiting addition step.

10.3 The regioselectivity

When using different MBH carbonates **1**, the regiodivergent [4+2] annulations were observed. As the regioselectivity was determined by the second step, to clarify the mechanism for the regioselectivity, the **TS**s for these steps were calculated. Since the energy barrier of **TS2′** was 1.8 kcal/mol higher than **TS2**, suggesting **3a** was the main product and predicted regio-isomer radio value was 21:1.

Comparing the structure of these **TS**s, we found there was an obvious π-π interaction between the benzene ring and indane ring, which might be the main reason to reduce the energy of **TS2** and to produce regioisomer **3a**.

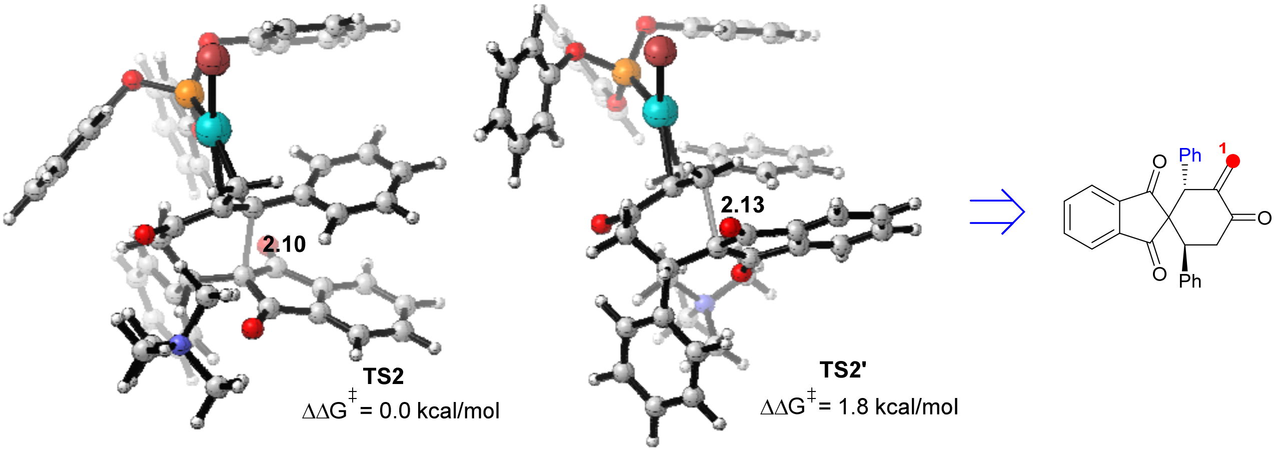

Figure S10 The energy of **TS2** and **TS2′** at the B3LYP-D3/6-311++G(d,p) // B3LYP-D3/6-31(d) SDD for Pd (toluene) level and is given in kcal/mol relative to energy of **TS2.**

On the other side, the energy barrier of **1q-TS2** was 7.3 kcal/mol higher than that of **1q-TS2′**, suggesting **4a** was the main product. These results were consistent with the experimental results.

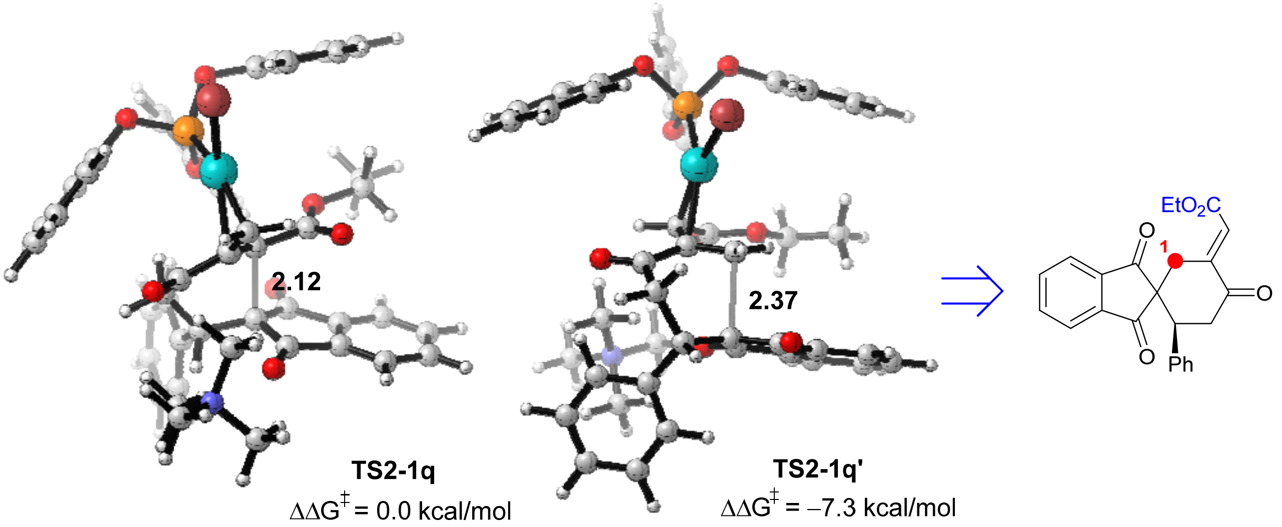

Figure S11 The energy of **TS2** and **TS2′** at the B3LYP-D3/6-311++G(d,p) // B3LYP-D3/6-31(d) SDD for Pd (toluene) level and is given in kcal/mol relative to energy of **1q-TS2′**

a)

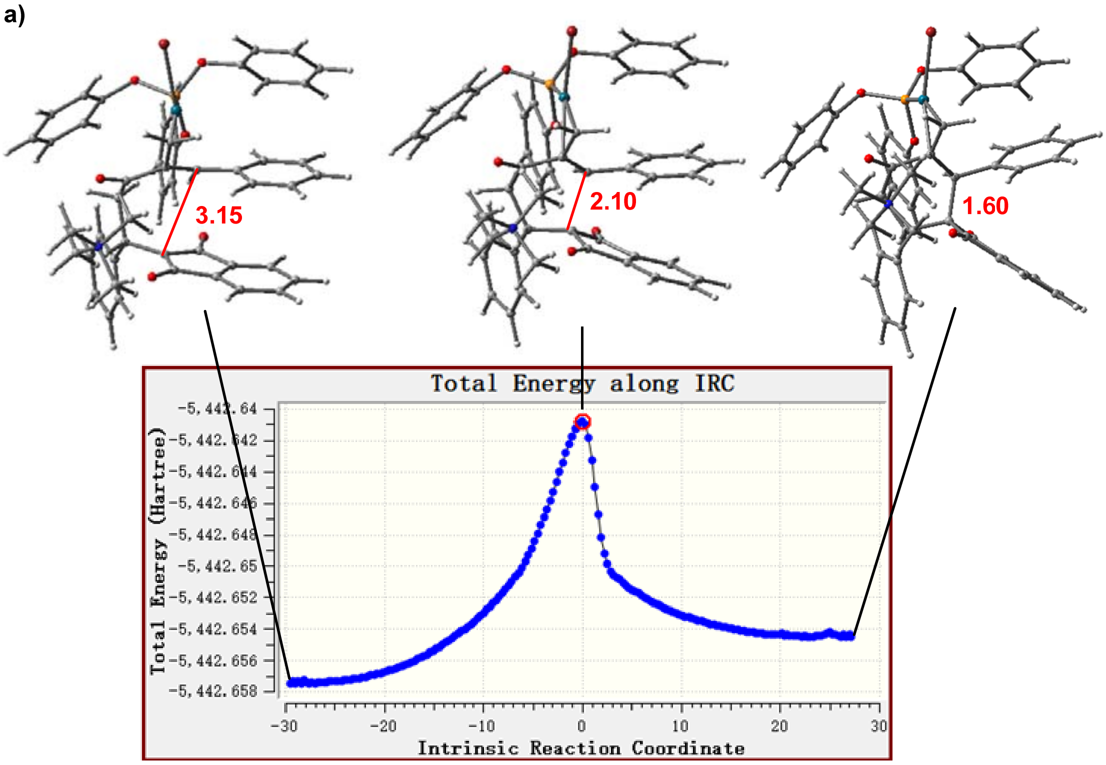

b)

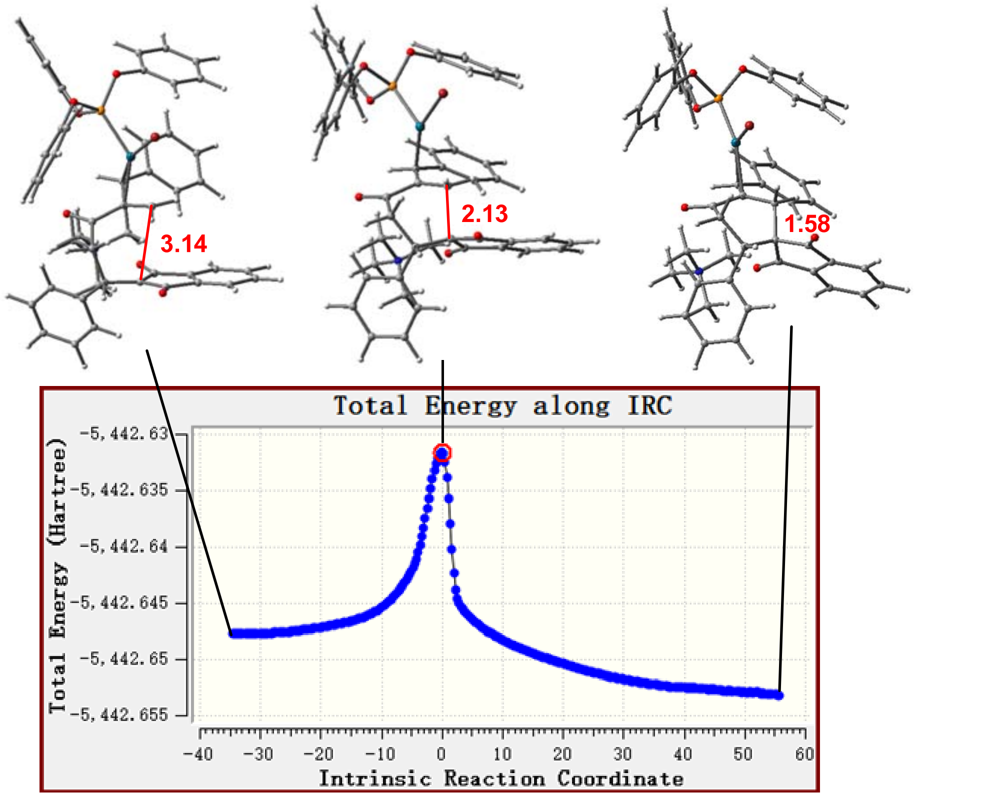

Figure S12 Intrinsic reaction coordinate analysis (IRC) for transition state **TS2 (a)** and **TS2′ (b).** The values are given in angstrom and represent bond length.

12.NMR, HRMS spectra and HPLC chromatograms

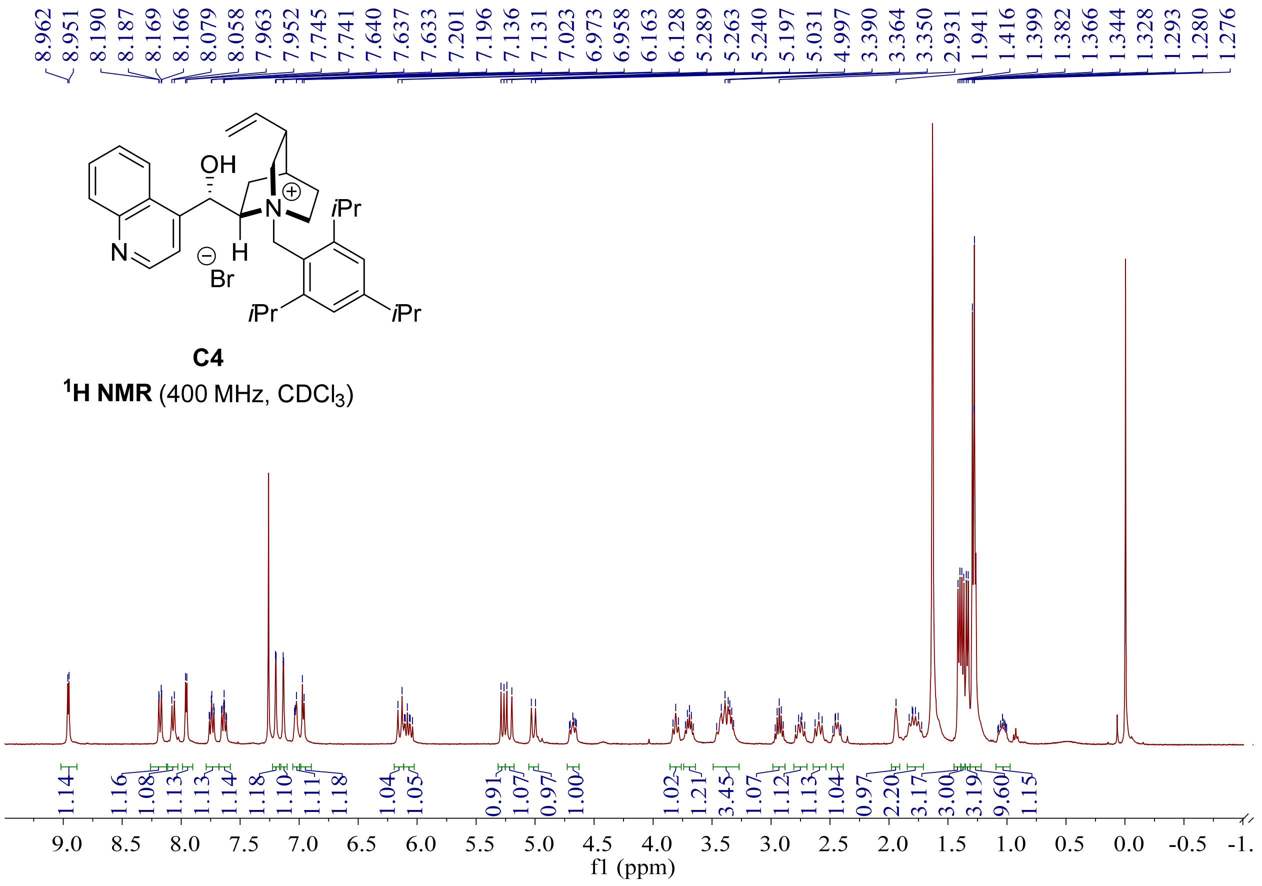

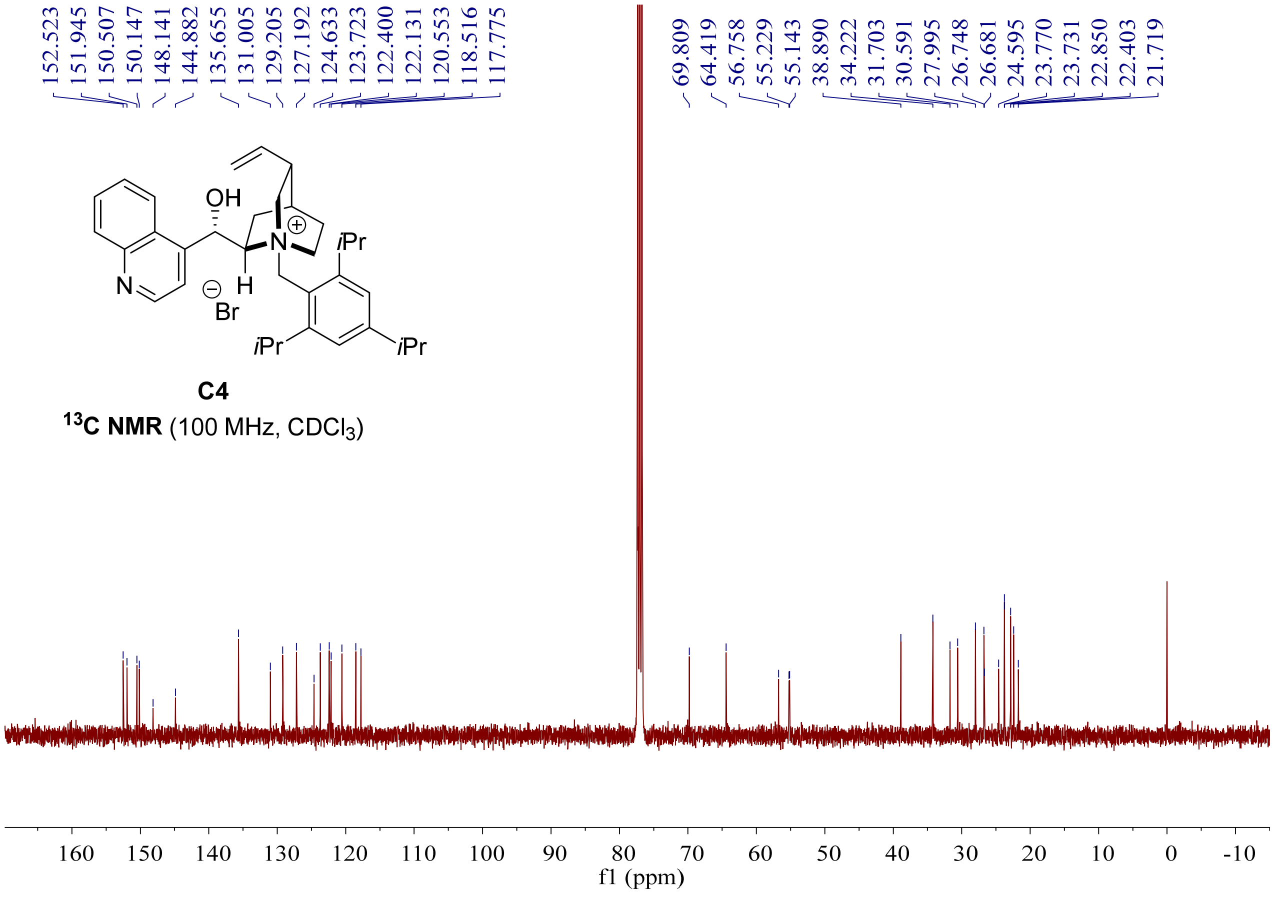

HRMS (ESI-TOF) m/z: [M − Br]$^+$ Calcd for $C_{35}H_{47}N_2O^+$ 511.3683; Found 511.3686.

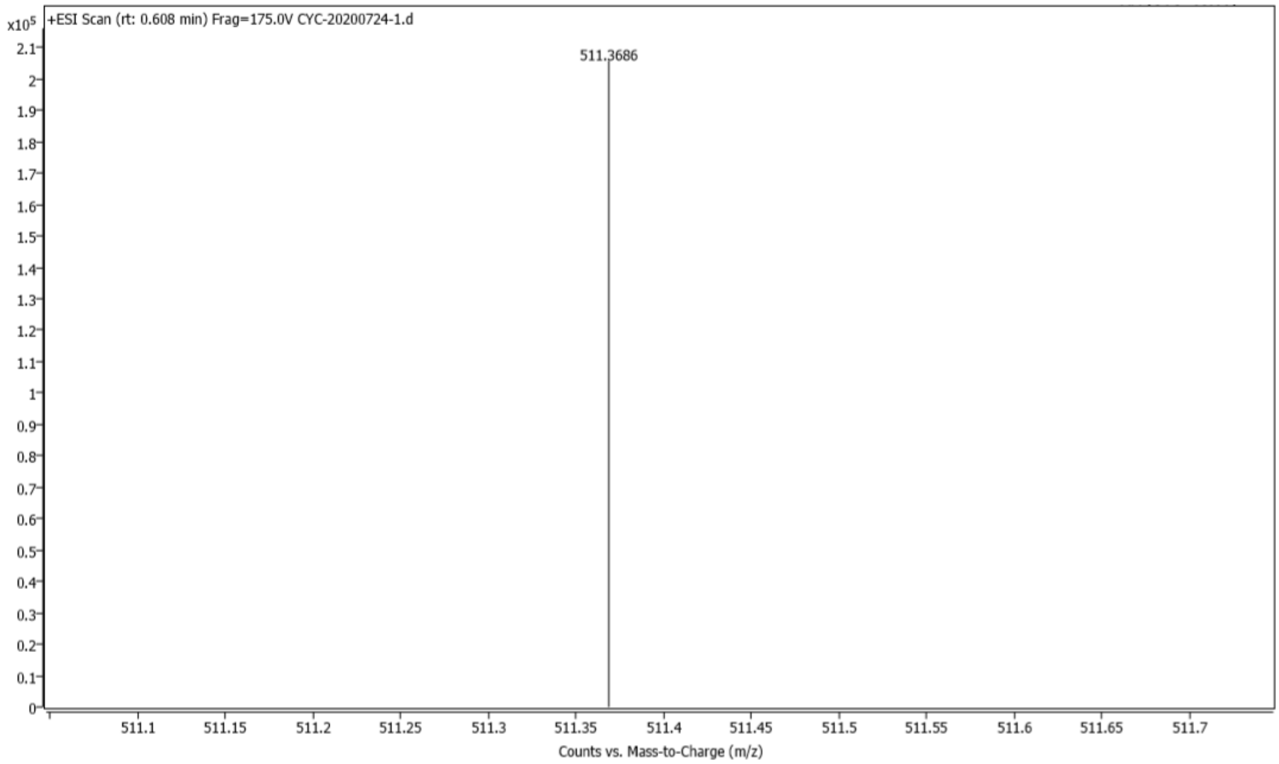

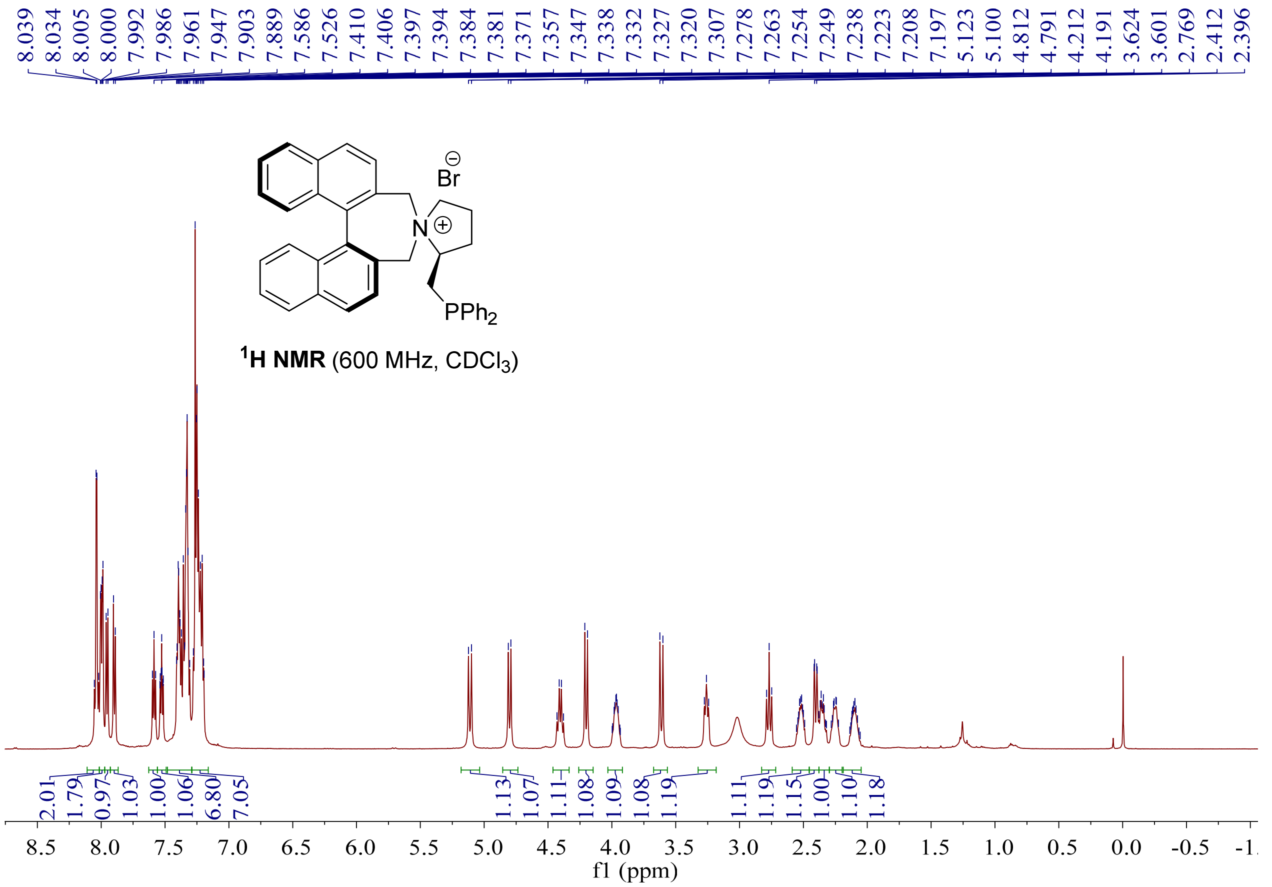
8.039 8.034 8.005 8.000 7.992 7.986 7.961 7.947 7.903 7.889 7.586 7.526 7.410 7.406 7.397 7.394 7.384 7.381 7.371 7.357 7.347 7.338 7.332 7.327 7.320 7.307 7.278 7.263 7.254 7.249 7.238 7.223 7.208 7.197 5.123 5.100 4.812 4.791 4.212 4.191 3.624 3.601 2.769 2.412 2.396
Br
N
PPh2
1H NMR (600 MHz, CDCl3)
2.01 1.79 0.97 1.03 1.00 1.06 6.80 7.05 1.13 1.07 1.11 1.08 1.09 1.08 1.19 1.11 1.19 1.15 1.00 1.10 1.18
f1 (ppm)

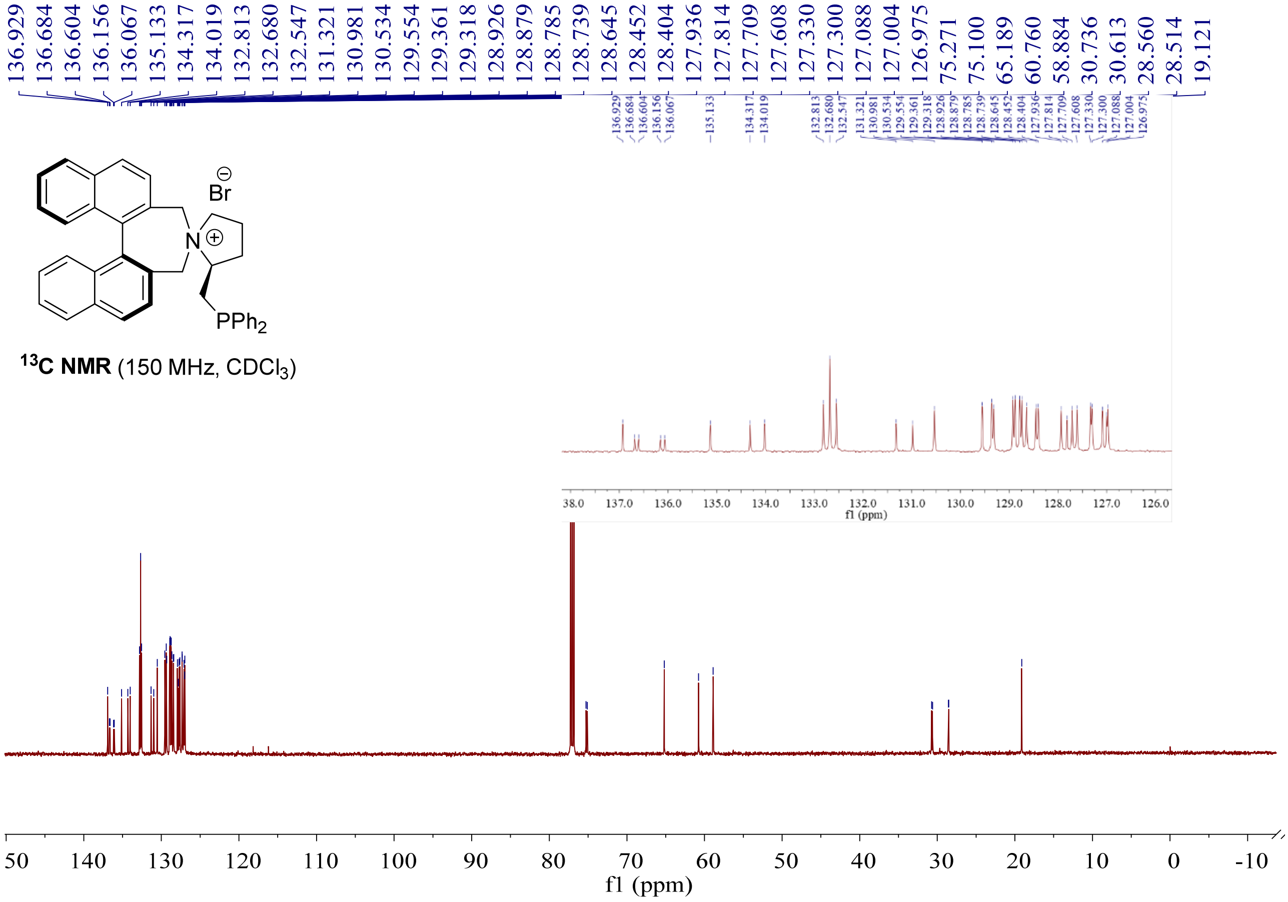
136.929 136.684 136.604 136.156 136.067 135.133 134.317 134.019 132.813 132.680 132.547 131.321 130.981 130.534 129.554 129.361 129.318 128.926 128.879 128.785 128.739 128.645 128.452 128.404 127.936 127.814 127.709 127.608 127.330 127.300 127.088 127.004 126.975 75.271 75.100 65.189 60.760 58.884 30.736 30.613 28.560 28.514 19.121
Br
N
PPh2
13C NMR (150 MHz, CDCl3)
f1 (ppm)

HRMS (ESI-TOF) m/z: $[M - Br]^+$ Calcd for $C_{39}H_{35}NP^+$ 548.2502 (^{31}P) and 549.2535 (^{32}P); Found 548.2503 (^{31}P) and 549.2543 (^{32}P).

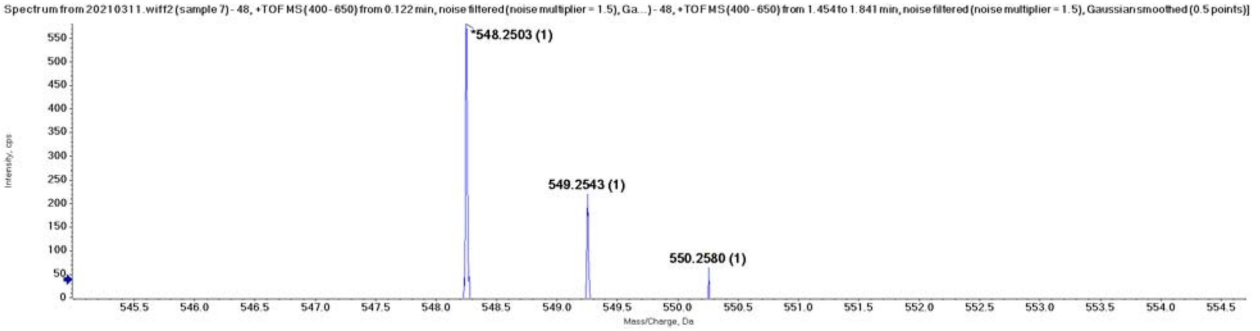

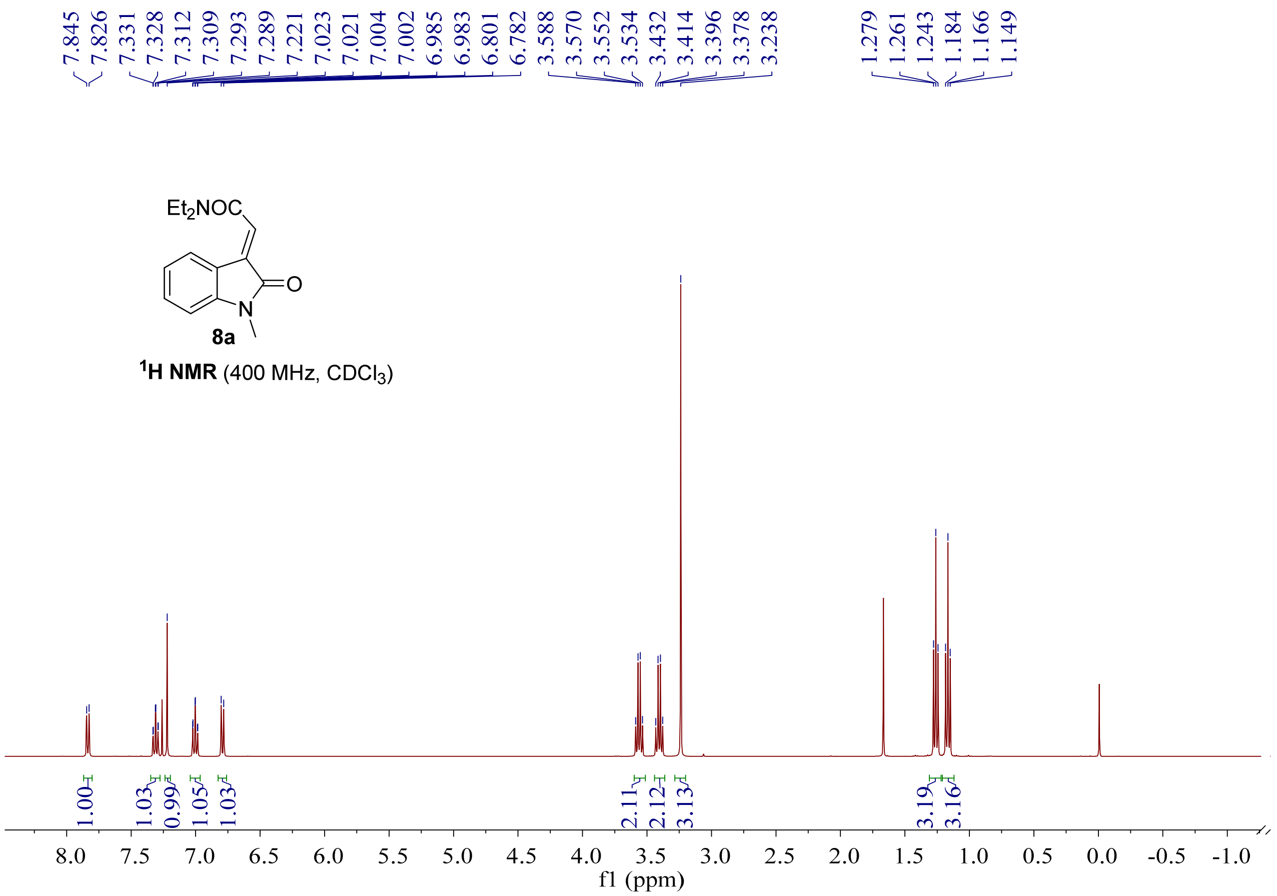
7.845
7.826
7.331
7.328
7.312
7.309
7.293
7.289
7.221
7.023
7.021
7.004
7.002
6.985
6.983
6.801
6.782
3.588
3.570
3.552
3.534
3.432
3.414
3.396
3.378
3.238
1.279
1.261
1.243
1.184
1.166
1.149
Et2NOC
O
N
8a
1H NMR (400 MHz, CDCl3)
1.00
1.03
0.99
1.05
1.03
2.11
2.12
3.13
3.19
3.16
8.0 7.5 7.0 6.5 6.0 5.5 5.0 4.5 4.0 3.5 3.0 2.5 2.0 1.5 1.0 0.5 0.0 -0.5 -1.0
f1 (ppm)

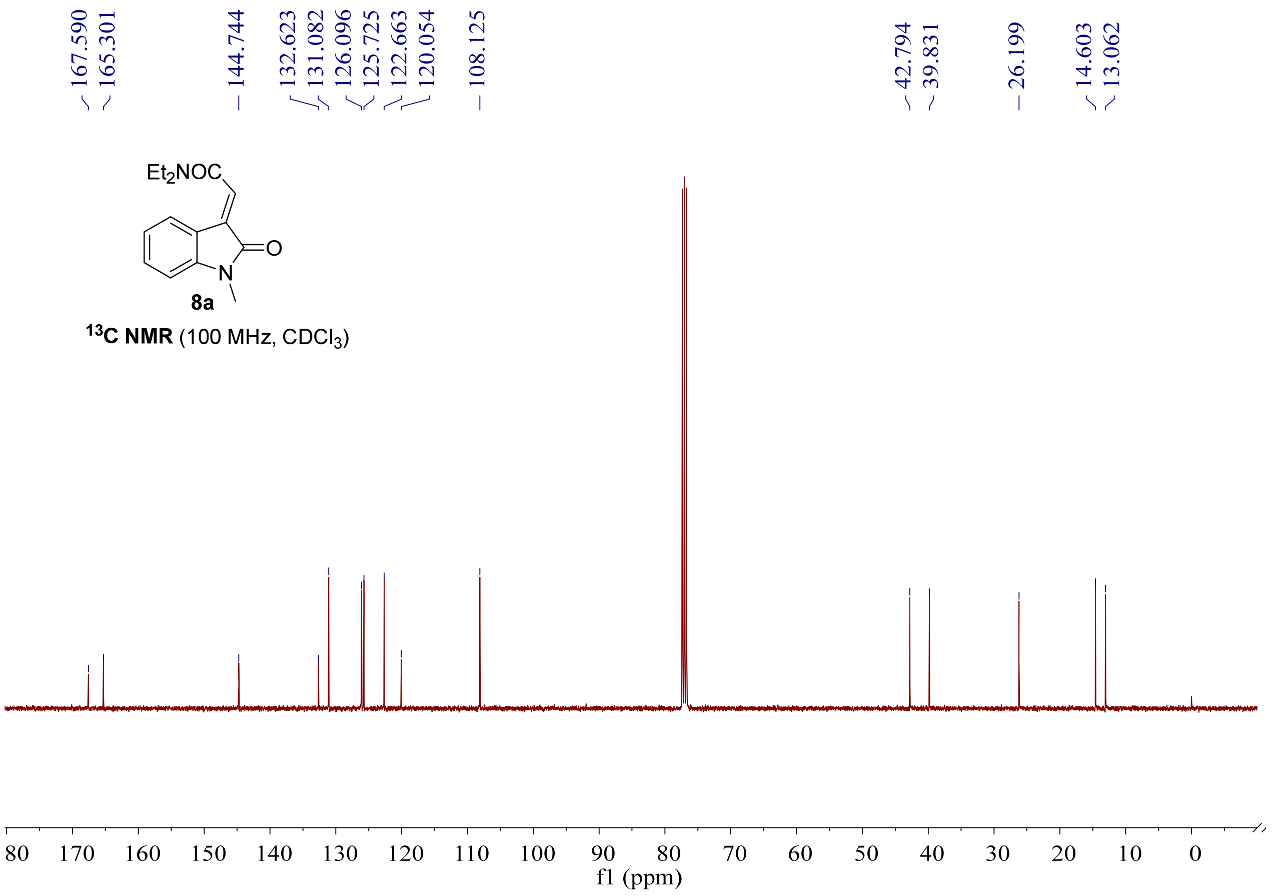
167.590
165.301
144.744
132.623
131.082
126.096
125.725
122.663
120.054
108.125
42.794
39.831
26.199
14.603
13.062
Et2NOC
O
N
8a
13C NMR (100 MHz, CDCl3)
80 170 160 150 140 130 120 110 100 90 80 70 60 50 40 30 20 10 0
f1 (ppm)

HRMS (ESI-TOF) m/z: $[M + Na]^+$ Calcd for $C_{15}H_{18}N_2O_2Na^+$ 281.1260; Found 281.1260.

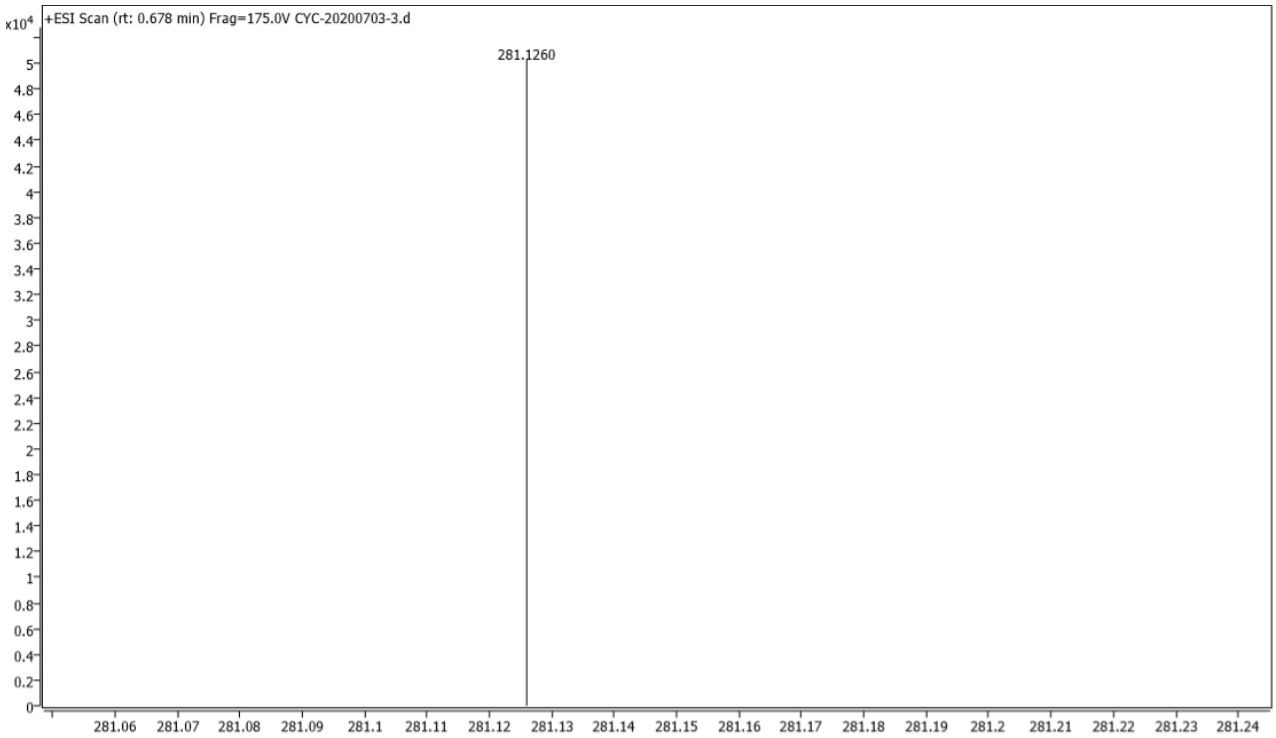

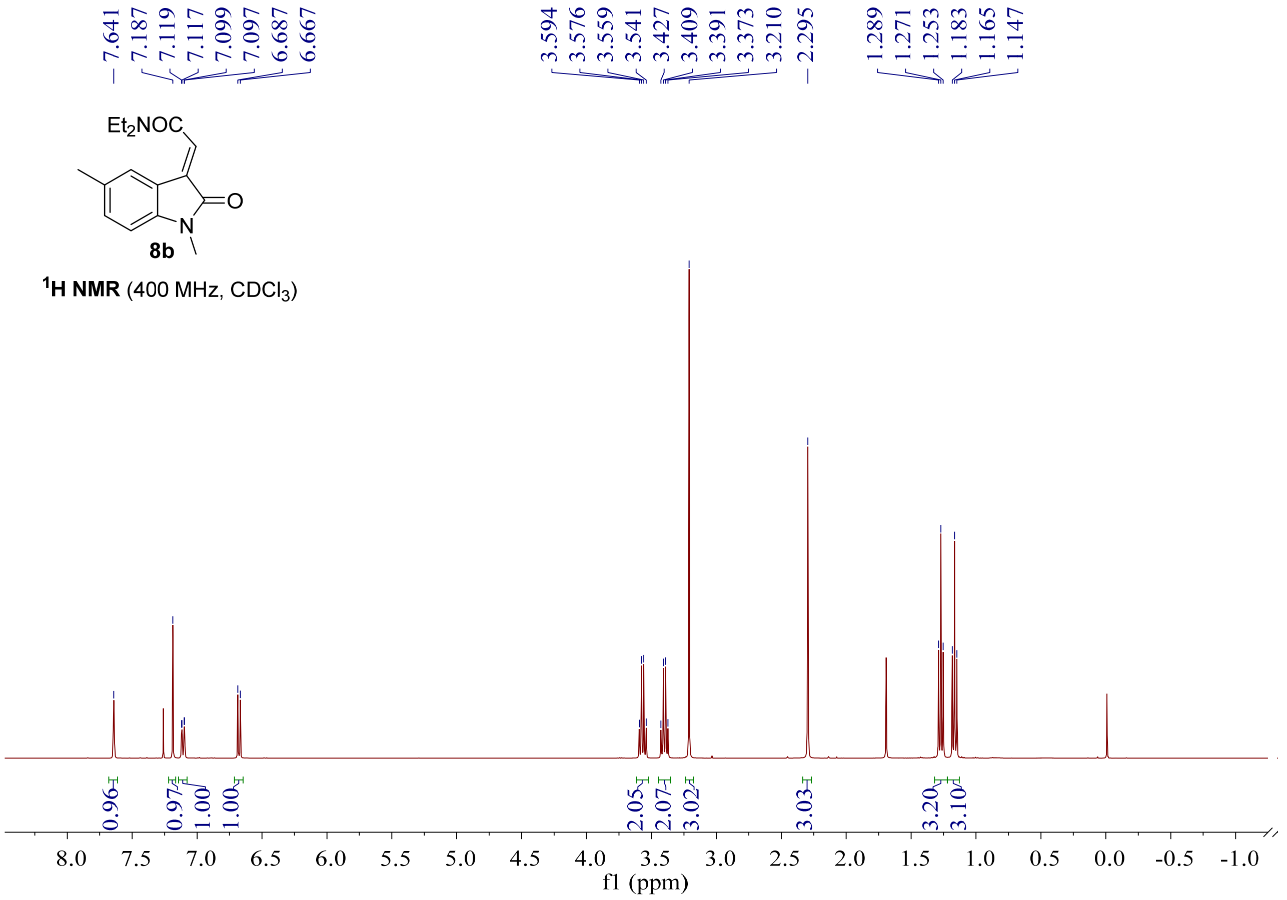

7.641
7.187
7.119
7.117
7.099
7.097
6.687
6.667
3.594
3.576
3.559
3.541
3.427
3.409
3.391
3.373
3.210
2.295
1.289
1.271
1.253
1.183
1.165
1.147
Et2NOC
8b
1H NMR (400 MHz, CDCl3)
0.96
0.97
1.00
1.00
2.05
2.07
3.02
3.03
3.20
3.10
f1 (ppm)

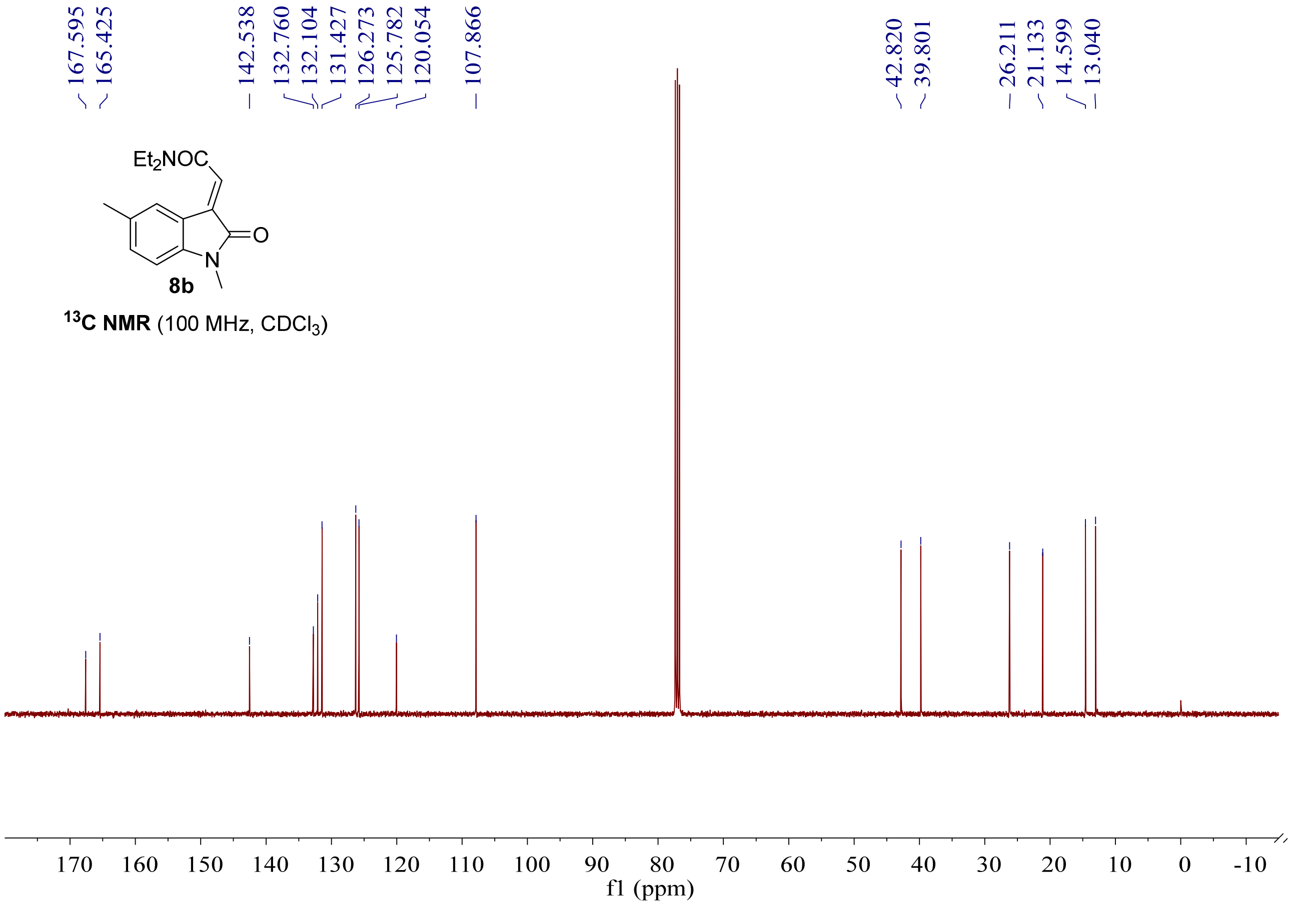

167.595
165.425
142.538
132.760
132.104
131.427
126.273
125.782
120.054
107.866
42.820
39.801
26.211
21.133
14.599
13.040
Et2NOC
8b
13C NMR (100 MHz, CDCl3)
f1 (ppm)

HRMS (ESI-TOF) m/z: [M + Na]$^+$ Calcd for $C_{16}H_{21}N_2O_2Na^+$ 295.1417; Found 295.1420.

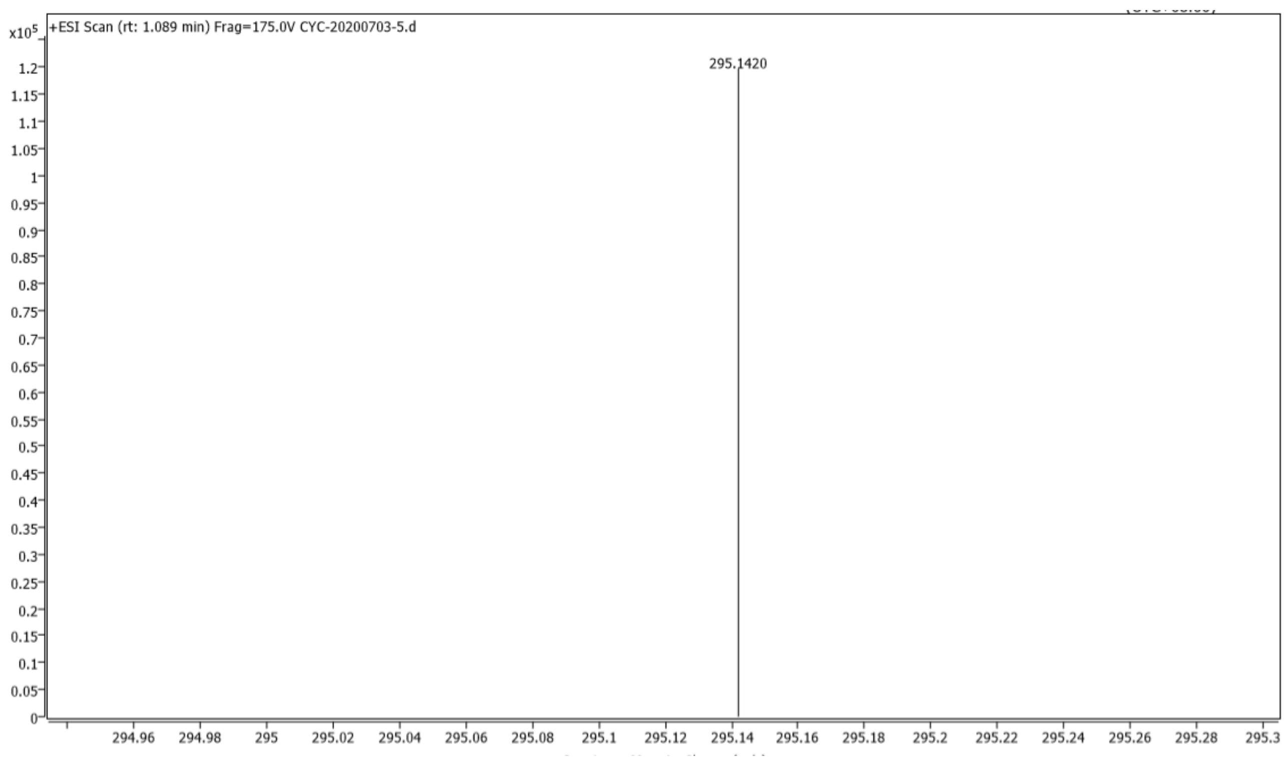

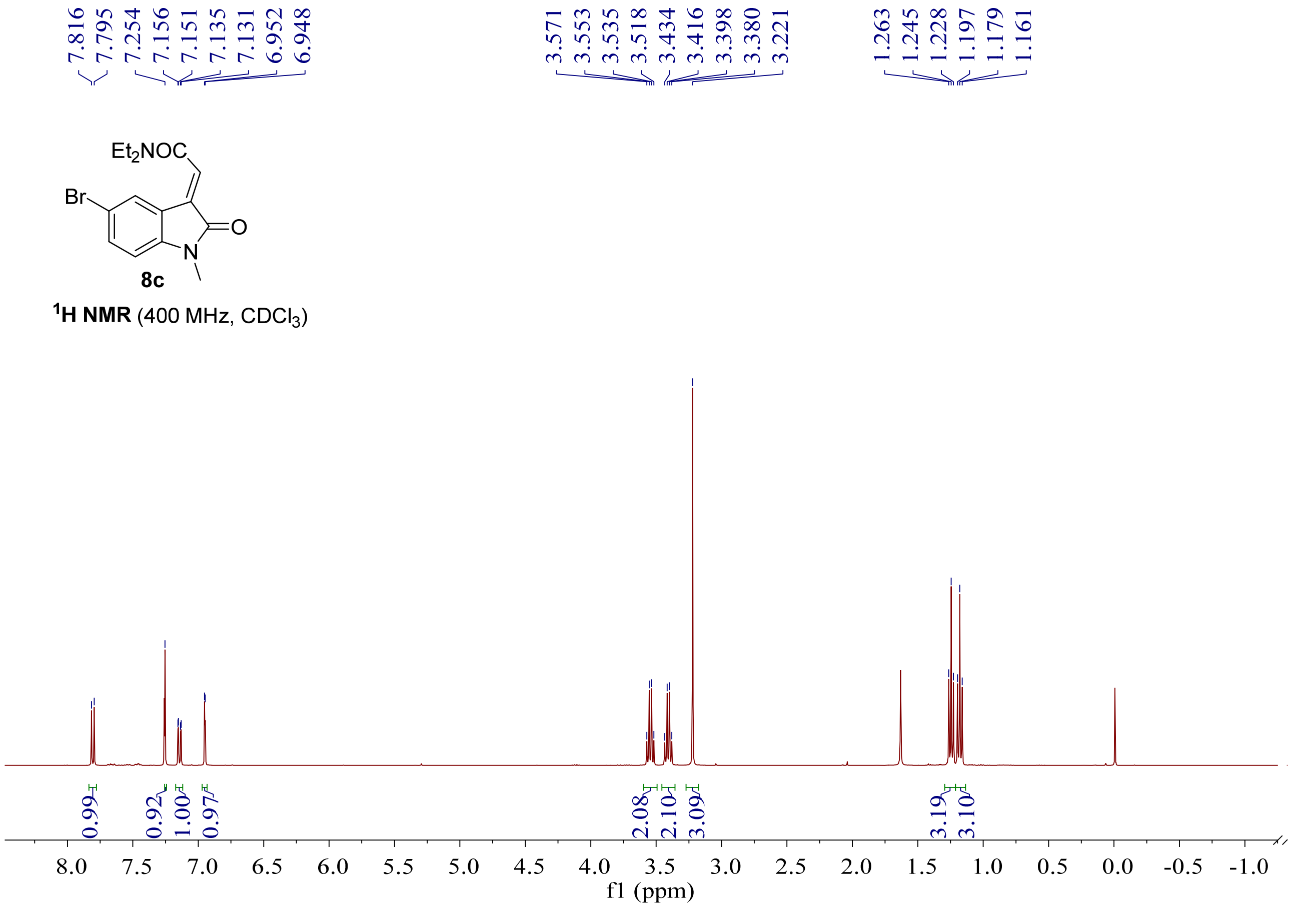
7.816
7.795
7.254
7.156
7.151
7.135
7.131
6.952
6.948
3.571
3.553
3.535
3.518
3.434
3.416
3.398
3.380
3.221
1.263
1.245
1.228
1.197
1.179
1.161
Et2NOC
Br
O
N
8c
1H NMR (400 MHz, CDCl3)
0.99
0.92
1.00
0.97
2.08
2.10
3.09
3.19
3.10
8.0
7.5
7.0
6.5
6.0
5.5
5.0
4.5
4.0
3.5
3.0
2.5
2.0
1.5
1.0
0.5
0.0
-0.5
-1.0
f1 (ppm)

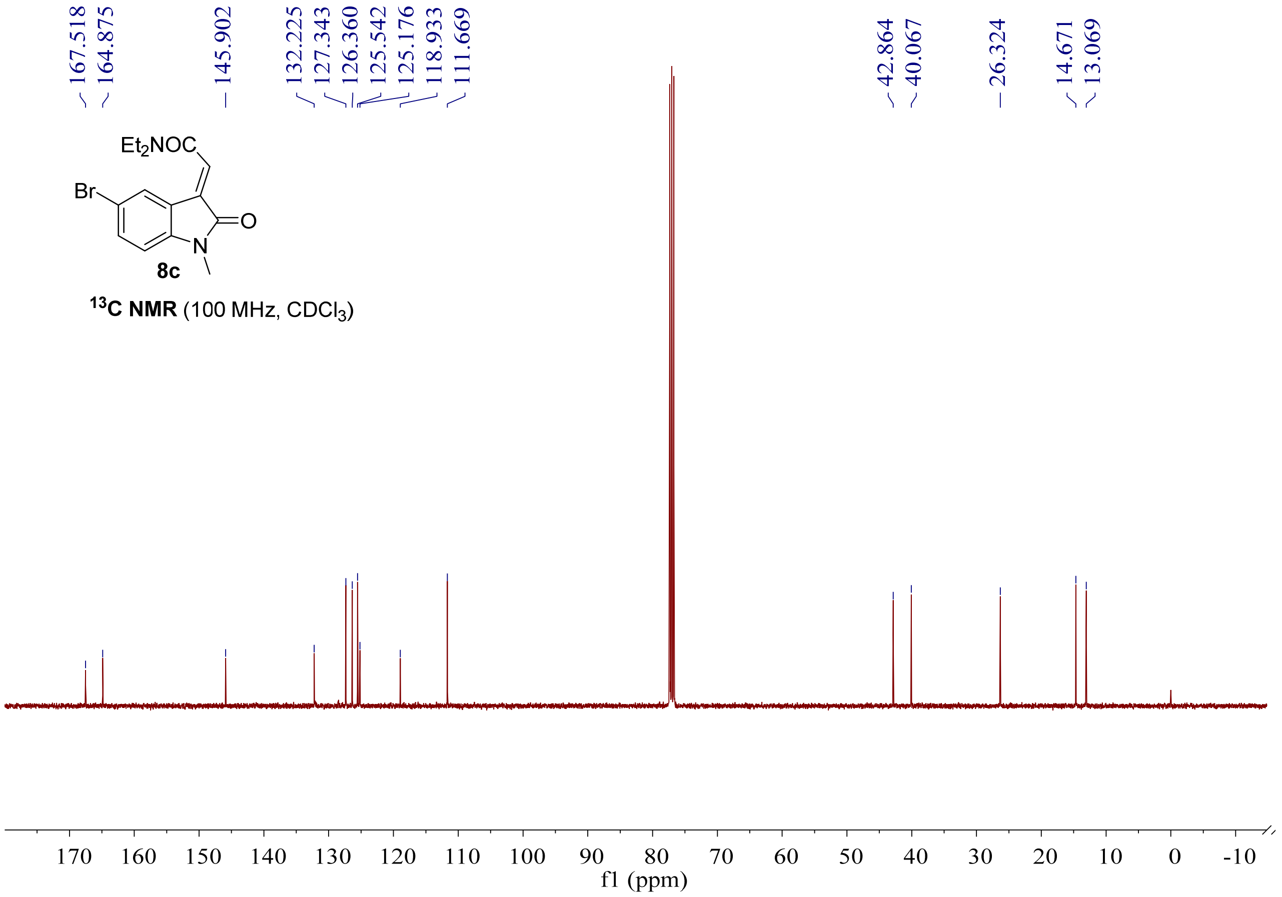
167.518
164.875
145.902
132.225
127.343
126.360
125.542
125.176
118.933
111.669
42.864
40.067
26.324
14.671
13.069
Et2NOC
Br
O
N
8c
13C NMR (100 MHz, CDCl3)
170
160
150
140
130
120
110
100
90
80
70
60
50
40
30
20
10
0
-10
f1 (ppm)

HRMS (ESI-TOF) m/z: $[M + H]^+$ Calcd for $C_{15}H_{18}N_2O_2Br^+$ 337.0546 (^{79}Br) and 339.0526 (^{81}Br); Found 337.0545 (^{79}Br) and 339.0525 (^{81}Br).

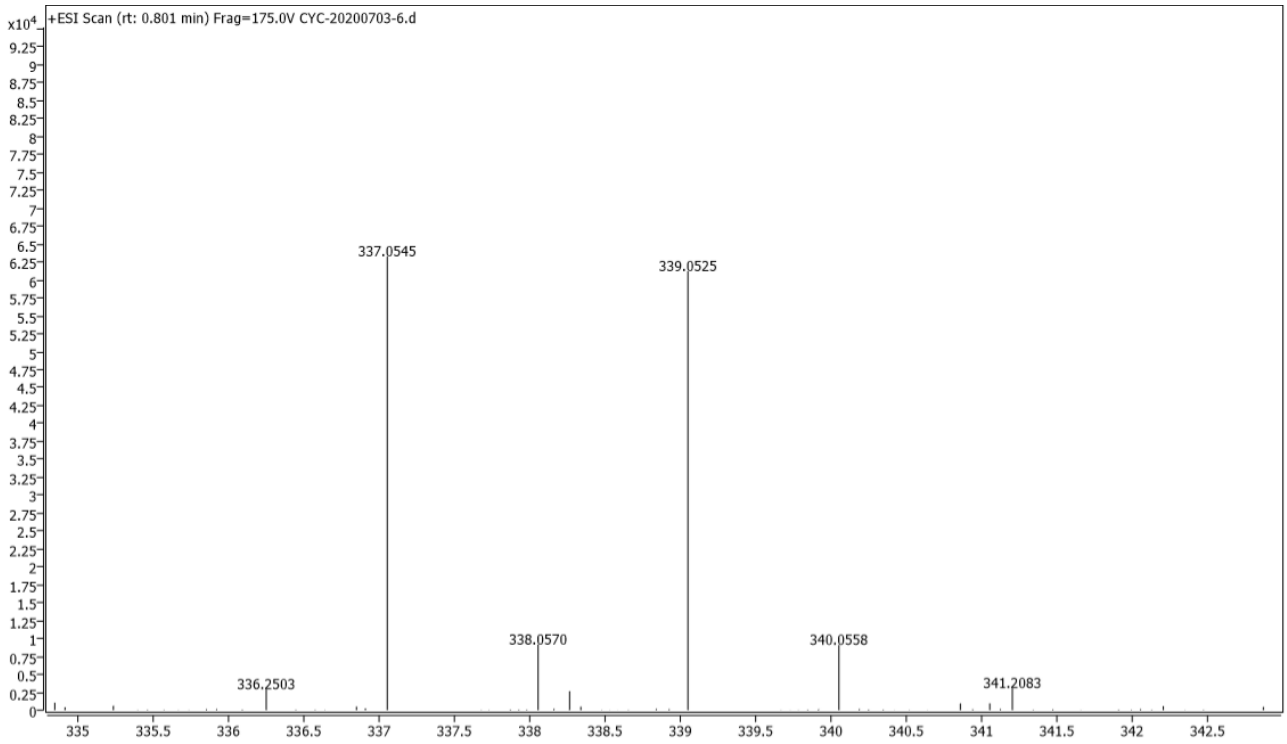

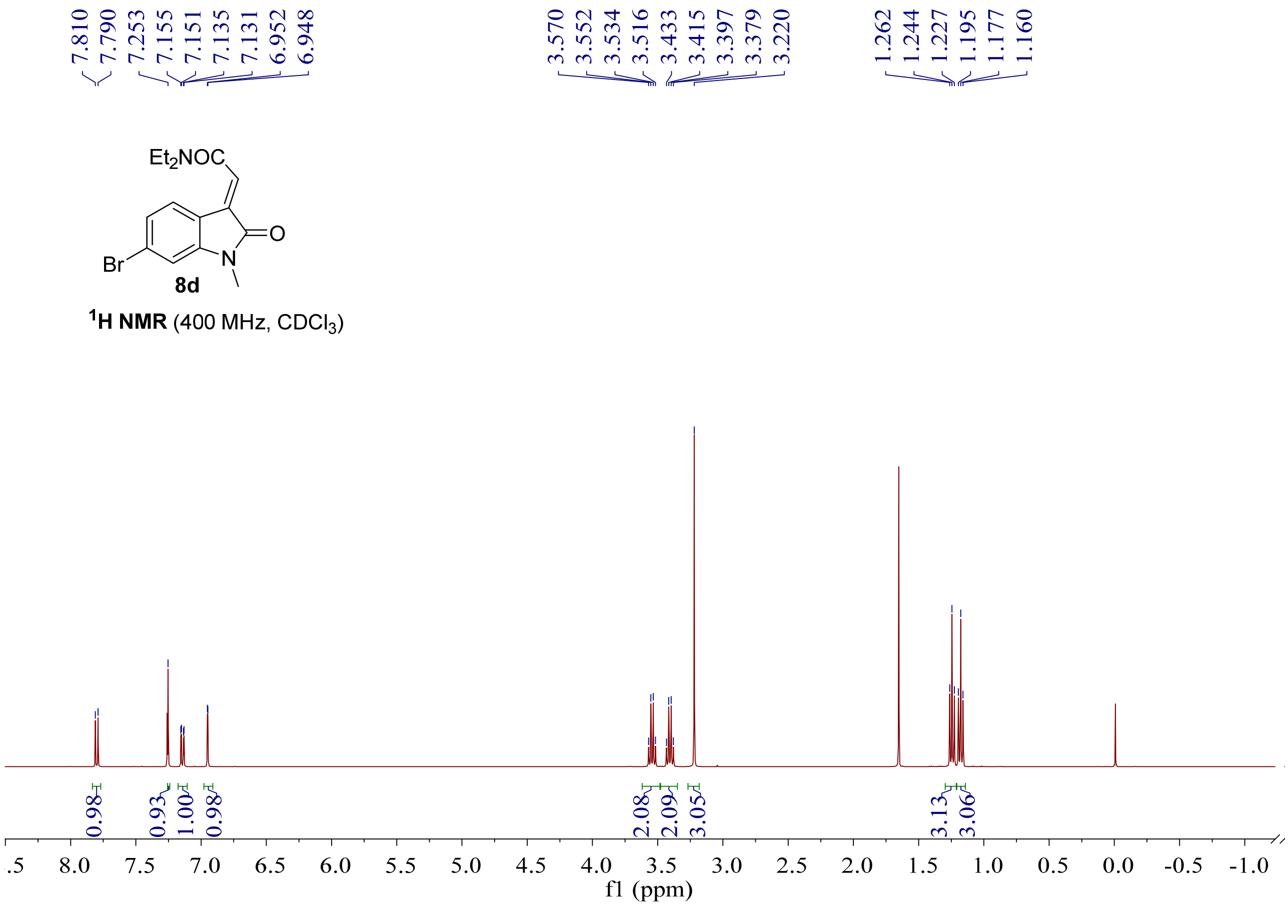
7.810
7.790
7.253
7.155
7.151
7.135
7.131
6.952
6.948
3.570
3.552
3.534
3.516
3.433
3.415
3.397
3.379
3.220
1.262
1.244
1.227
1.195
1.177
1.160
Et2NOC
O
Br
N
8d
1H NMR (400 MHz, CDCl3)
0.98
0.93
1.00
0.98
2.08
2.09
3.05
3.13
3.06
f1 (ppm)

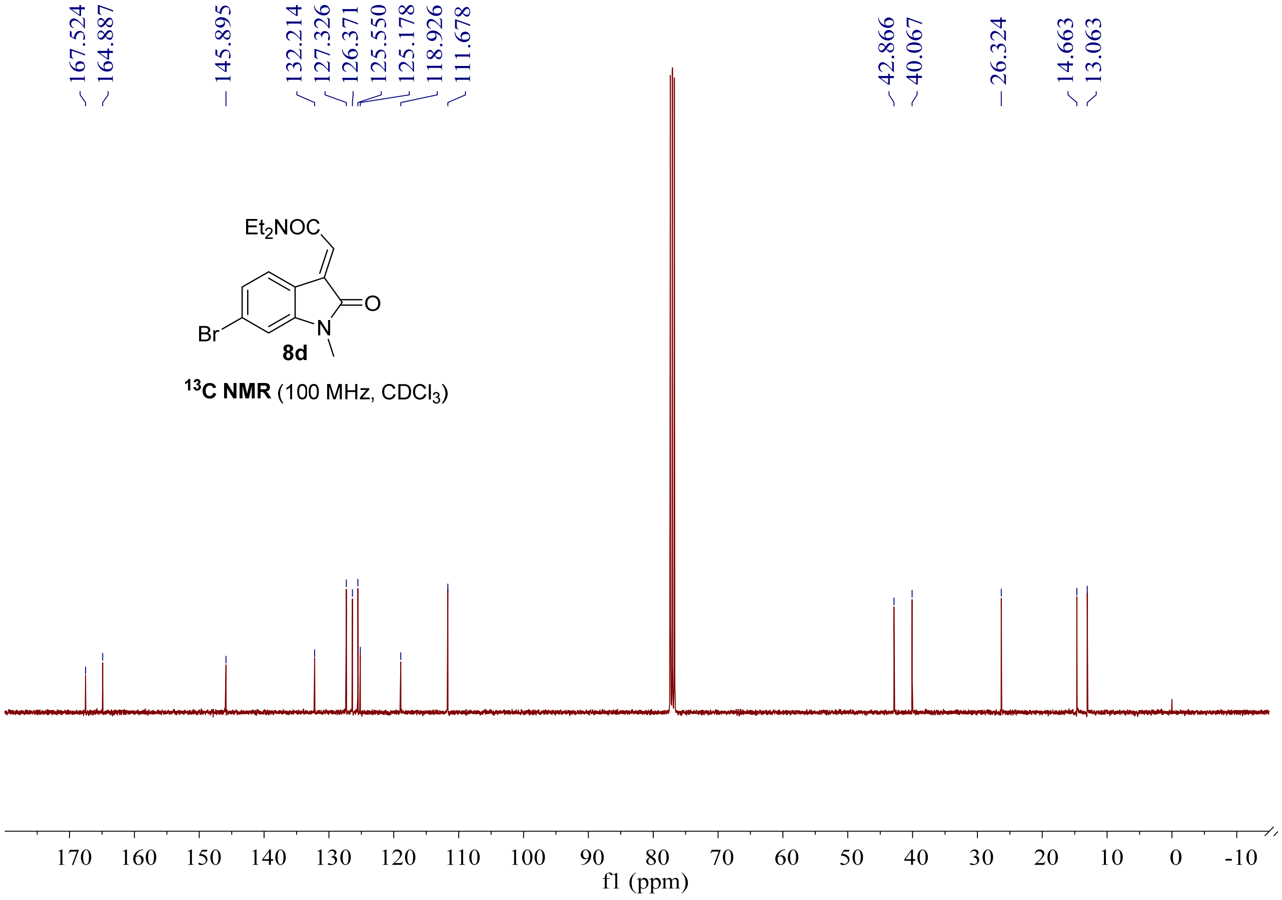
167.524
164.887
145.895
132.214
127.326
126.371
125.550
125.178
118.926
111.678
42.866
40.067
26.324
14.663
13.063
Et2NOC
O
Br
N
8d
13C NMR (100 MHz, CDCl3)
f1 (ppm)

HRMS (ESI-TOF) m/z: [M + Na]$^+$Calcd for $C_{15}H_{17}N_2O_2NaBr^+$ 359.0366 (^{79}Br) and 361.0345 (^{81}Br); Found 359.0371 (^{79}Br) and 361.0352 (^{81}Br).

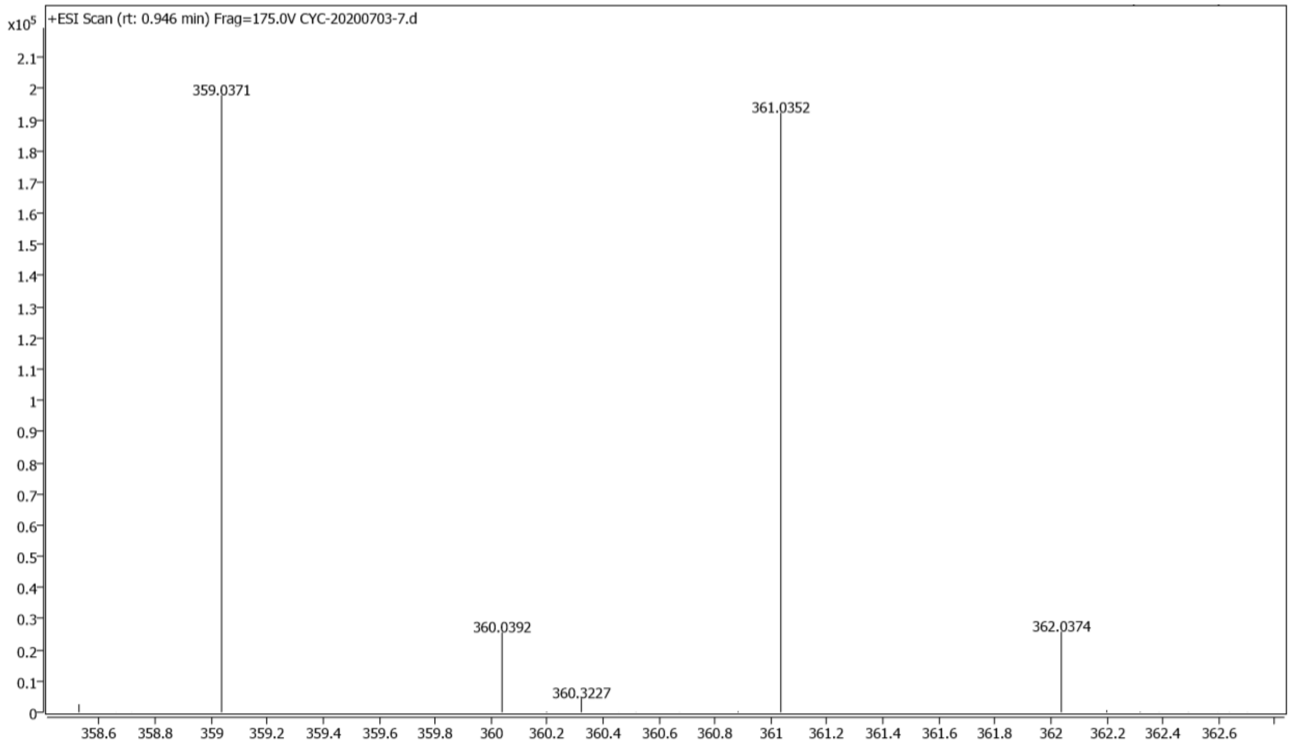

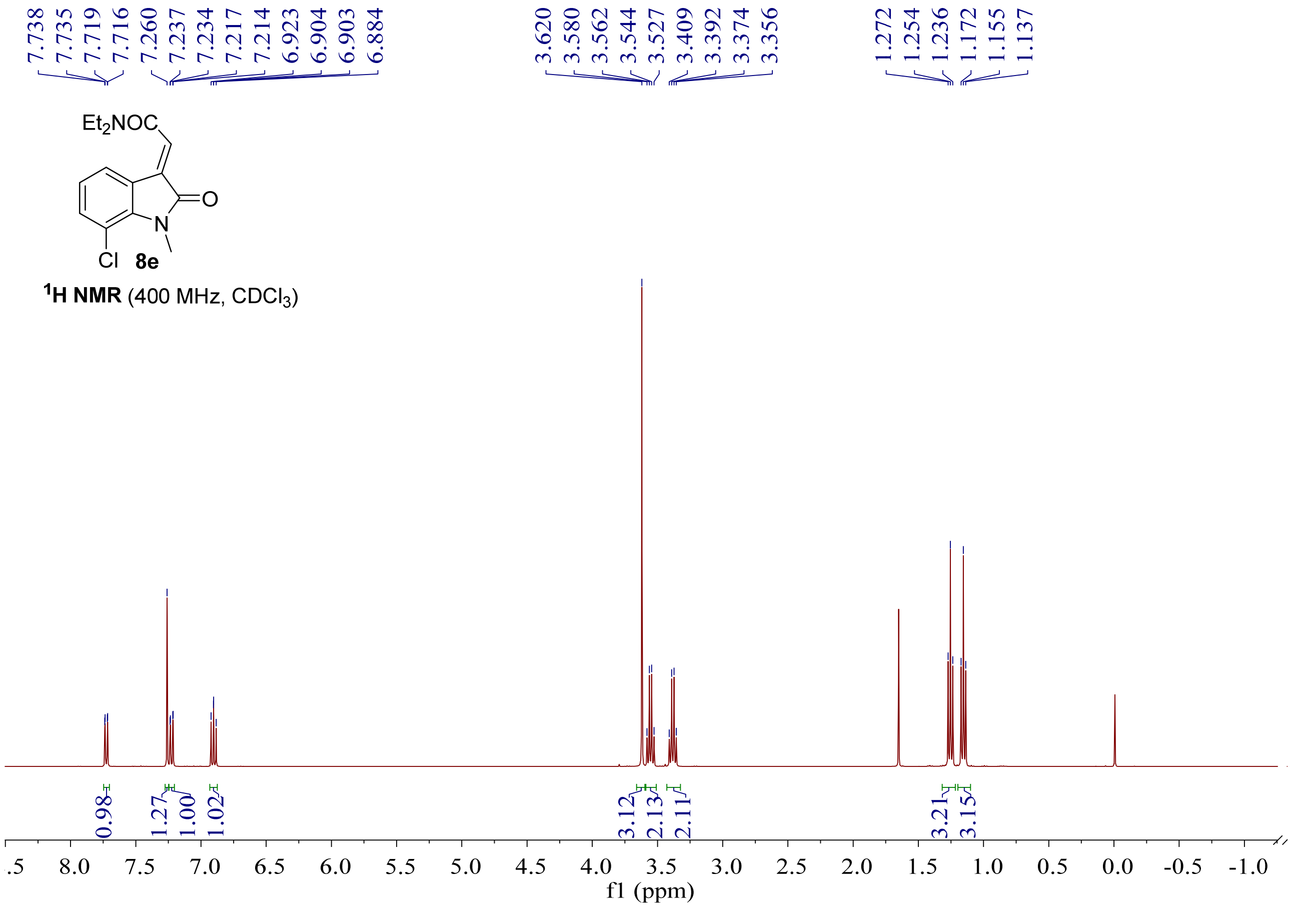
7.738
7.735
7.719
7.716
7.260
7.237
7.234
7.217
7.214
6.923
6.904
6.903
6.884
3.620
3.580
3.562
3.544
3.527
3.409
3.392
3.374
3.356
1.272
1.254
1.236
1.172
1.155
1.137
Et2NOC
O
N
Cl
8e
1H NMR (400 MHz, CDCl3)
0.98
1.27
1.00
1.02
3.12
2.13
2.11
3.21
3.15
f1 (ppm)

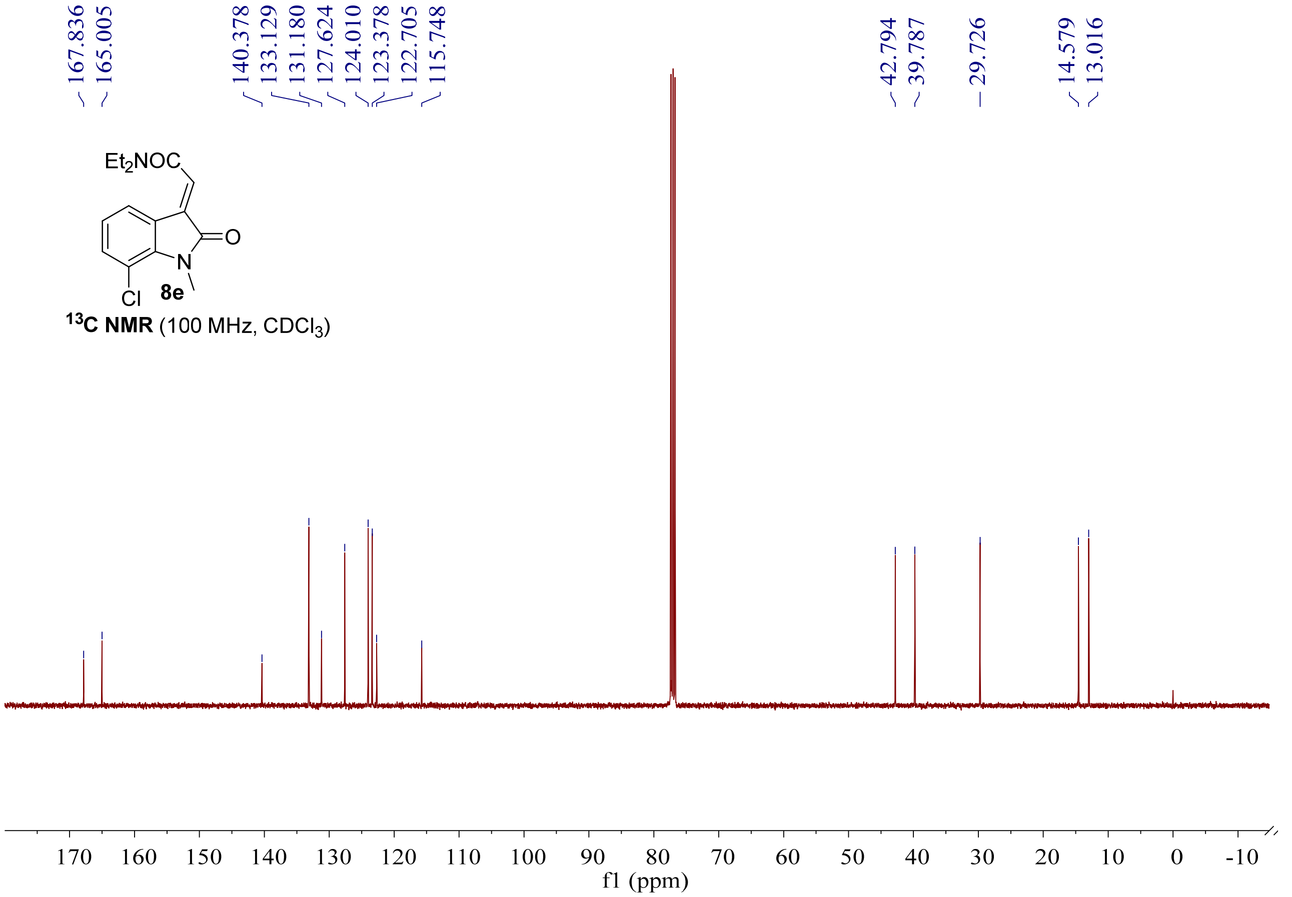
167.836
165.005
140.378
133.129
131.180
127.624
124.010
123.378
122.705
115.748
42.794
39.787
29.726
14.579
13.016
Et2NOC
O
N
Cl
8e
13C NMR (100 MHz, CDCl3)
f1 (ppm)

HRMS (ESI-TOF) m/z: $[M + Na]^+$ Calcd for $C_{15}H_{17}N_2O_2NaCl^+$ 315.0871 (^{35}Cl) and 317.0841 (^{37}Cl); Found 315.0883 (^{35}Cl) and 317.0861 (^{37}Cl).

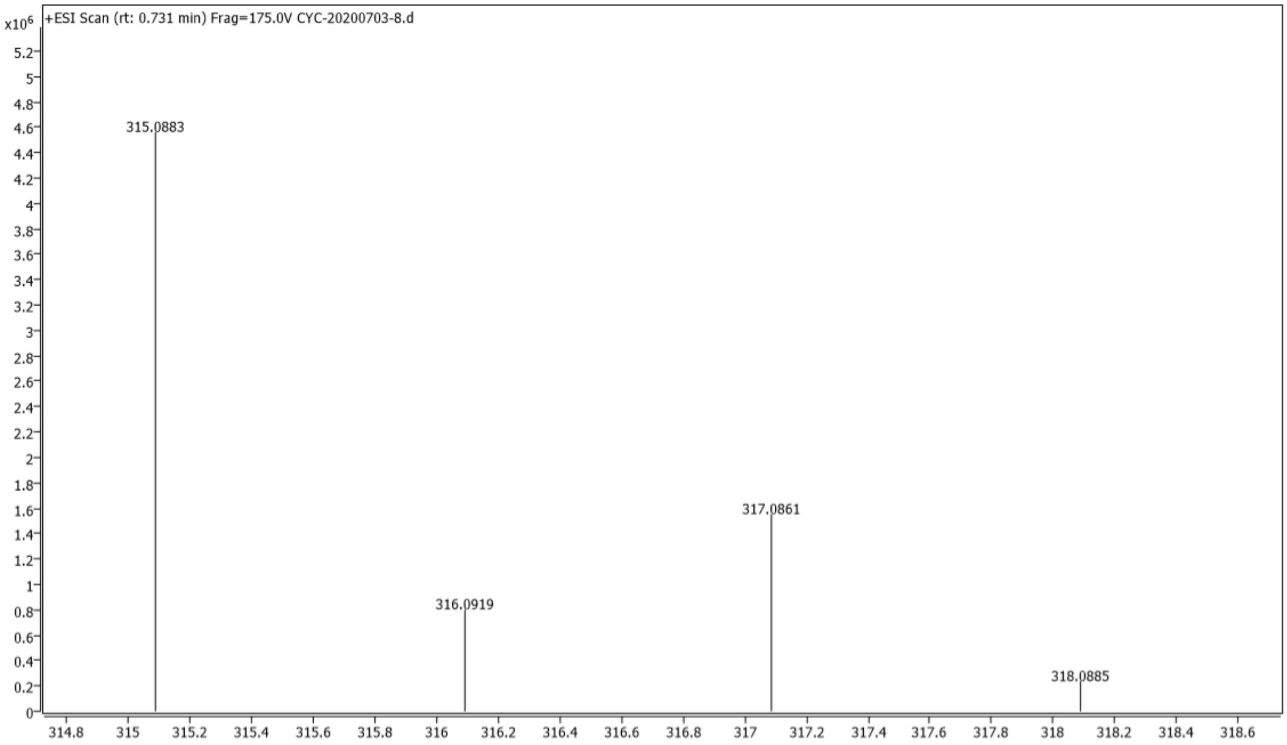

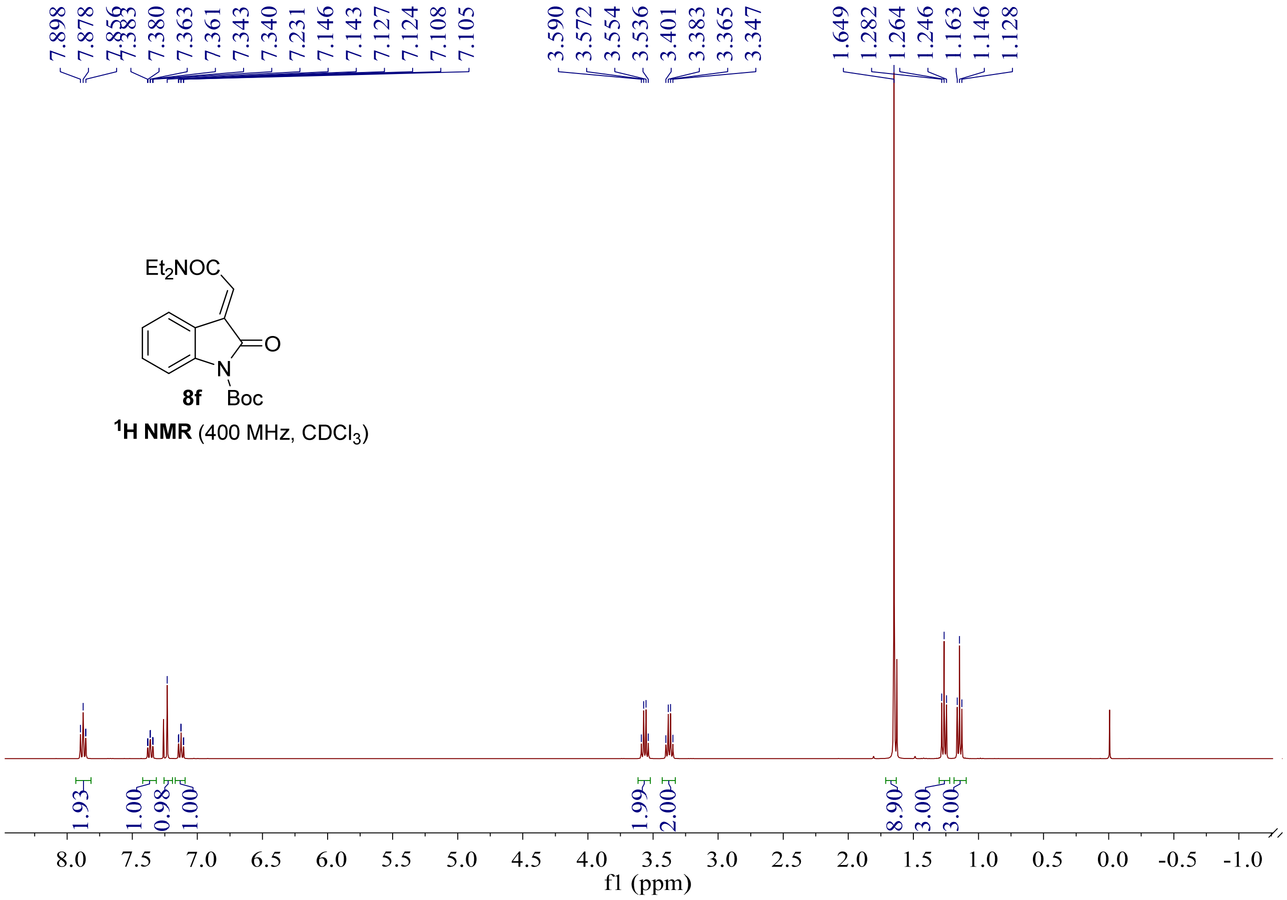
7.898
7.878
7.856
7.383
7.380
7.363
7.361
7.343
7.340
7.231
7.146
7.143
7.127
7.124
7.108
7.105
3.590
3.572
3.554
3.536
3.401
3.383
3.365
3.347
1.649
1.282
1.264
1.246
1.163
1.146
1.128
Et2NOC
O
N
8f
Boc
1H NMR (400 MHz, CDCl3)
1.93
1.00
0.98
1.00
1.99
2.00
8.90
3.00
3.00
f1 (ppm)

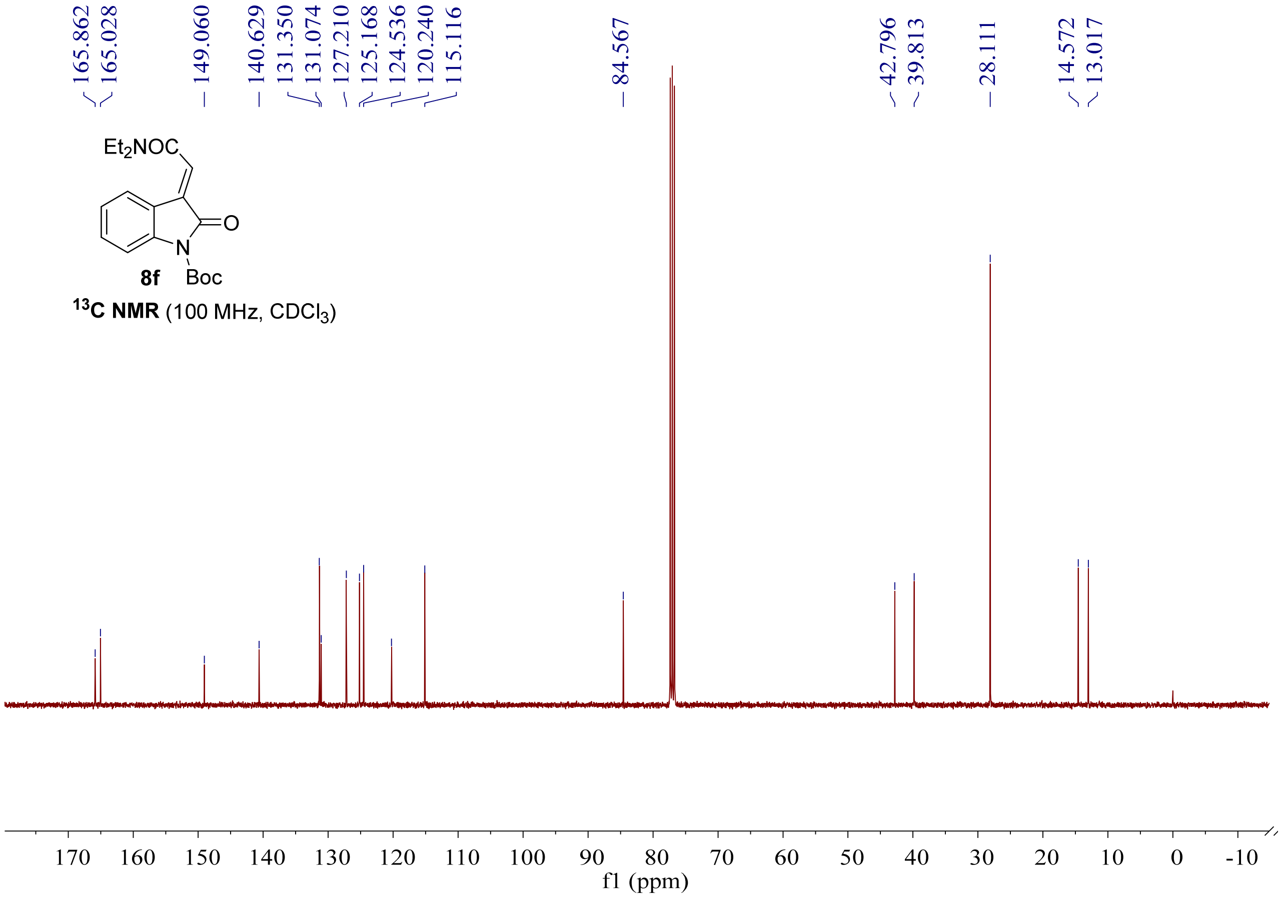
165.862
165.028
149.060
140.629
131.350
131.074
127.210
125.168
124.536
120.240
115.116
84.567
42.796
39.813
28.111
14.572
13.017
Et2NOC
O
N
8f
Boc
13C NMR (100 MHz, CDCl3)
f1 (ppm)

HRMS (ESI-TOF) m/z: $[M + H]^+$ Calcd for $C_{19}H_{25}N_2O_4^+$ 345.1809; Found 345.1811.

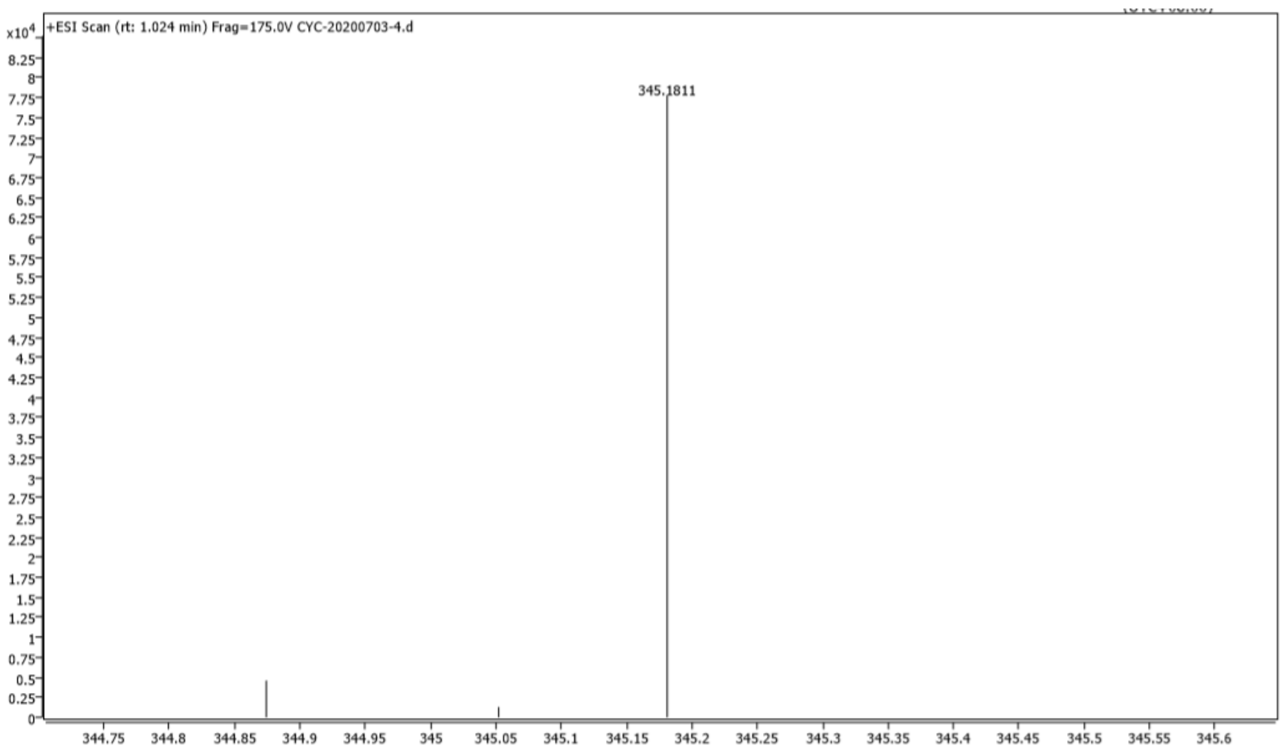

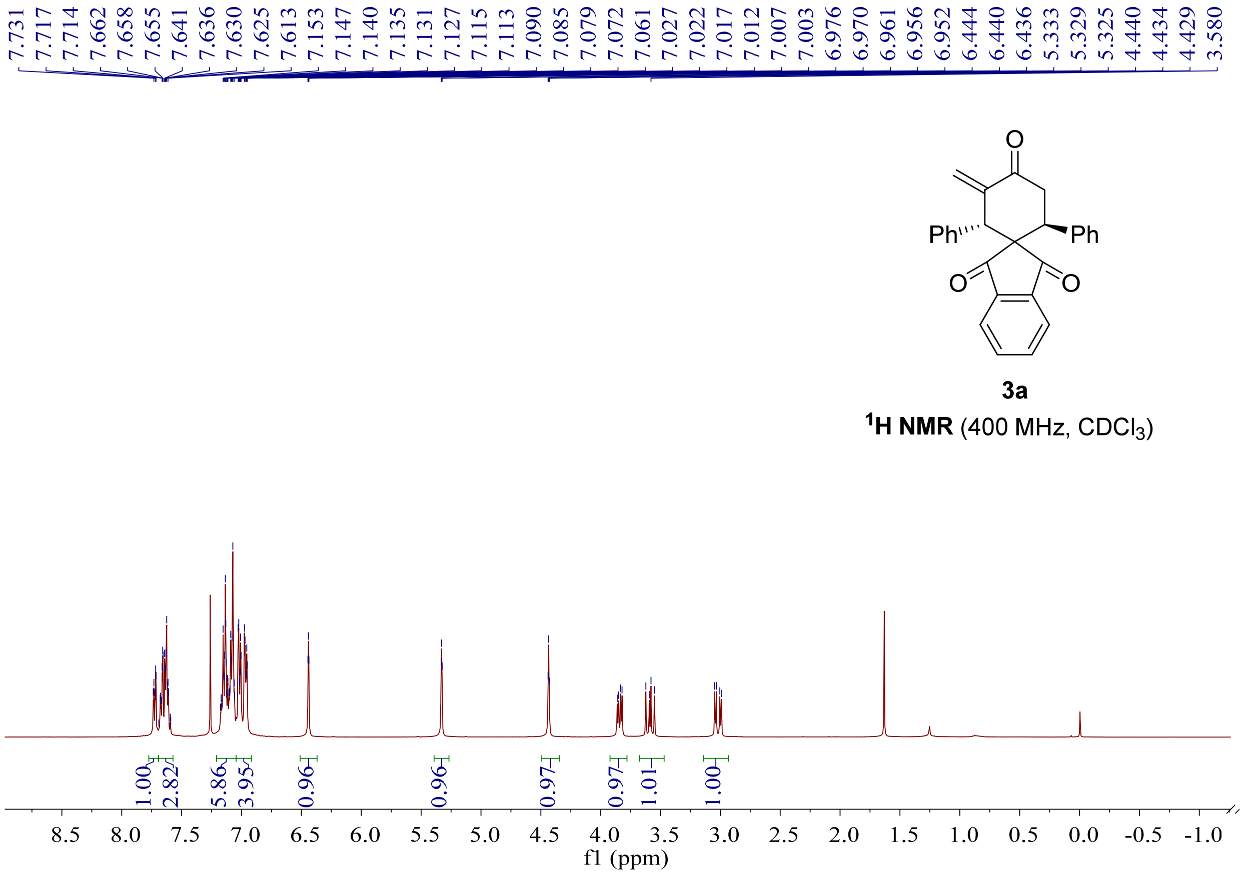
7.731
7.717
7.714
7.662
7.658
7.655
7.641
7.636
7.630
7.625
7.613
7.153
7.147
7.140
7.135
7.131
7.127
7.115
7.113
7.090
7.085
7.079
7.072
7.061
7.027
7.022
7.017
7.012
7.007
7.003
6.976
6.970
6.961
6.956
6.952
6.444
6.440
6.436
5.333
5.329
5.325
4.440
4.434
4.429
3.580
O
Ph
Ph
O
O
3a
1H NMR (400 MHz, CDCl3)
1.00
2.82
5.86
3.95
0.96
0.96
0.97
0.97
1.01
1.00
8.5 8.0 7.5 7.0 6.5 6.0 5.5 5.0 4.5 4.0 3.5 3.0 2.5 2.0 1.5 1.0 0.5 0.0 -0.5 -1.0
f1 (ppm)

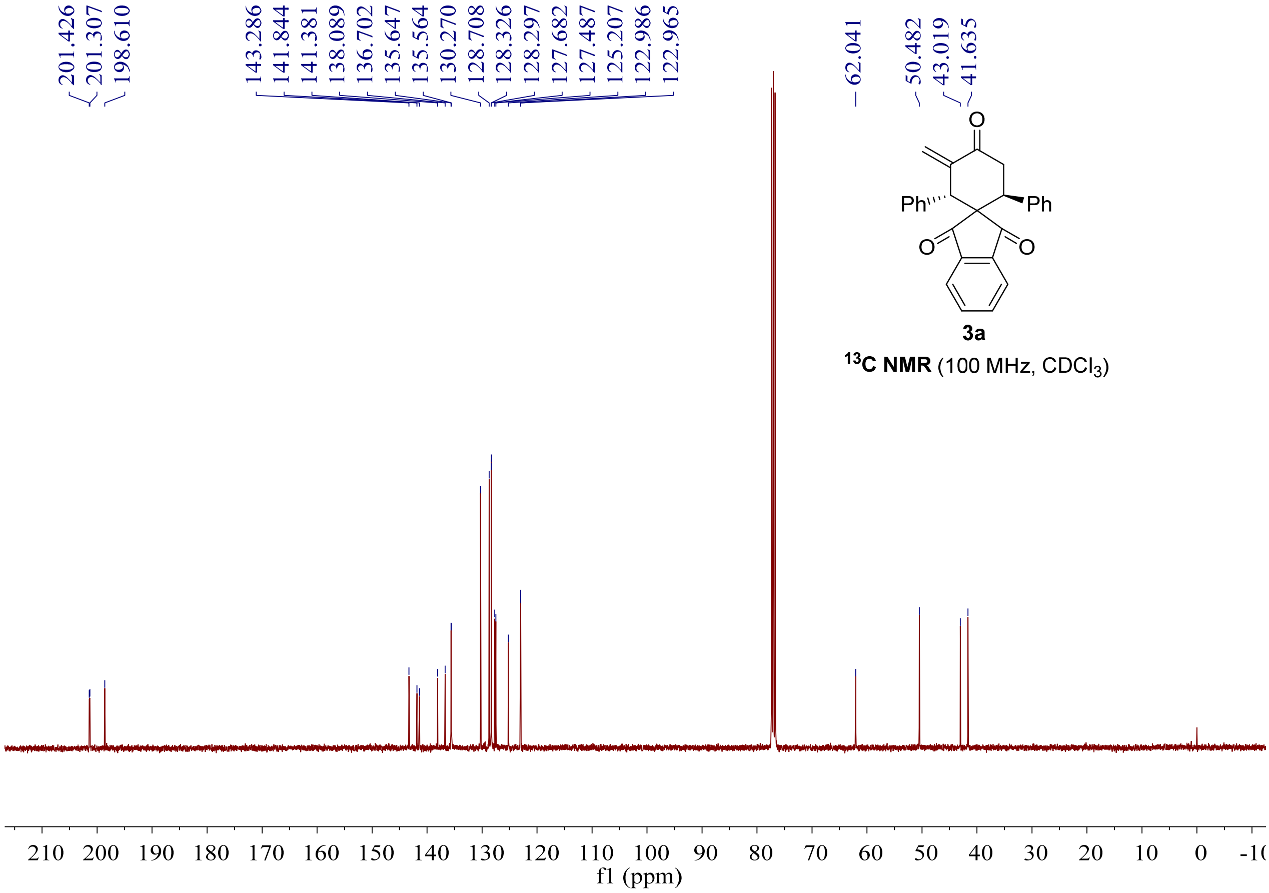
201.426
201.307
198.610
143.286
141.844
141.381
138.089
136.702
135.647
135.564
130.270
128.708
128.326
128.297
127.682
127.487
125.207
122.986
122.965
62.041
50.482
43.019
41.635
O
Ph
Ph
O
O
3a
13C NMR (100 MHz, CDCl3)
210 200 190 180 170 160 150 140 130 120 110 100 90 80 70 60 50 40 30 20 10 0 -1(
f1 (ppm)

Daicel Chiral AD-H Column, (*i*PrOH/*n*-hexane = 40/60, 1.0 mL/min)

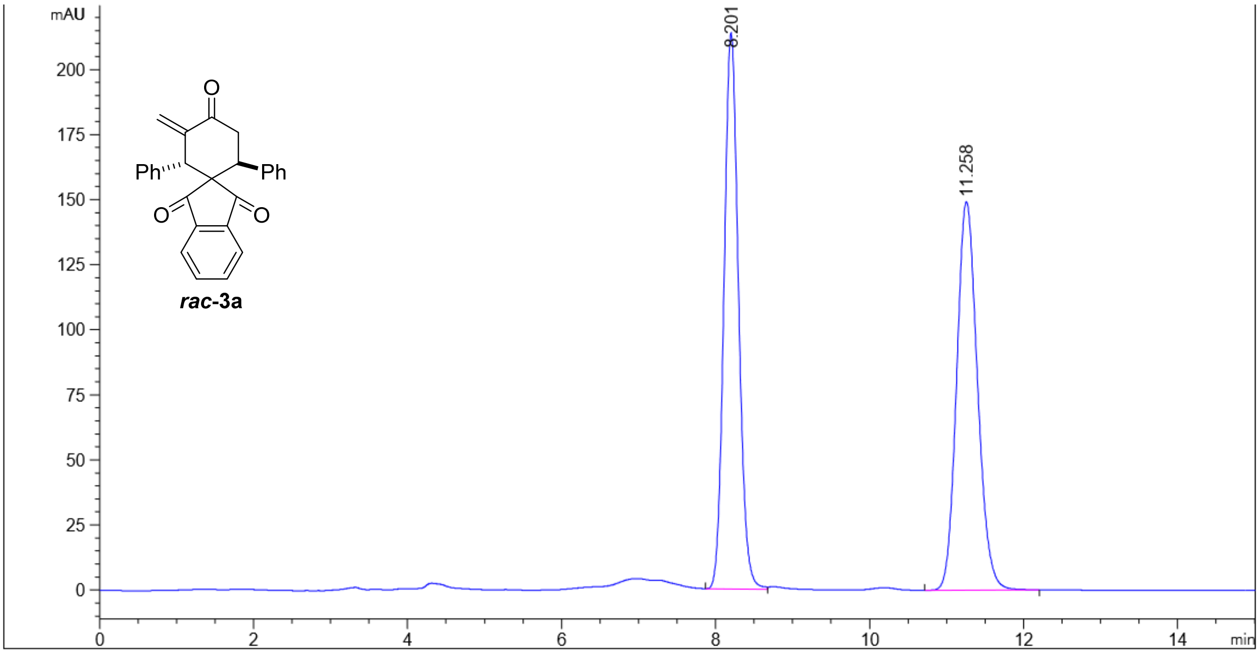

Time	Area (%)
8.201	49.9
11.258	50.1

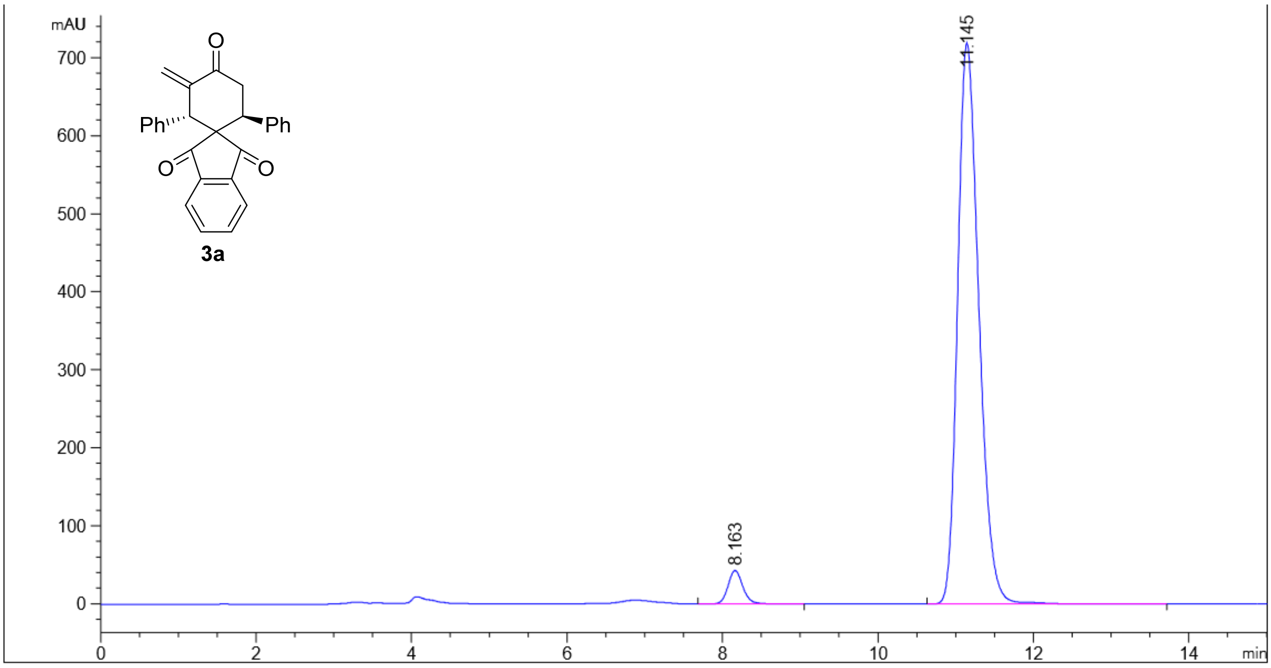

Time	Area (%)
8.163	4.0
11.145	96.0

HRMS (ESI-TOF) m/z: $[M + Na]^+$ Calcd for $C_{27}H_{20}O_3Na^+$ 415.1305; Found 415.1308.

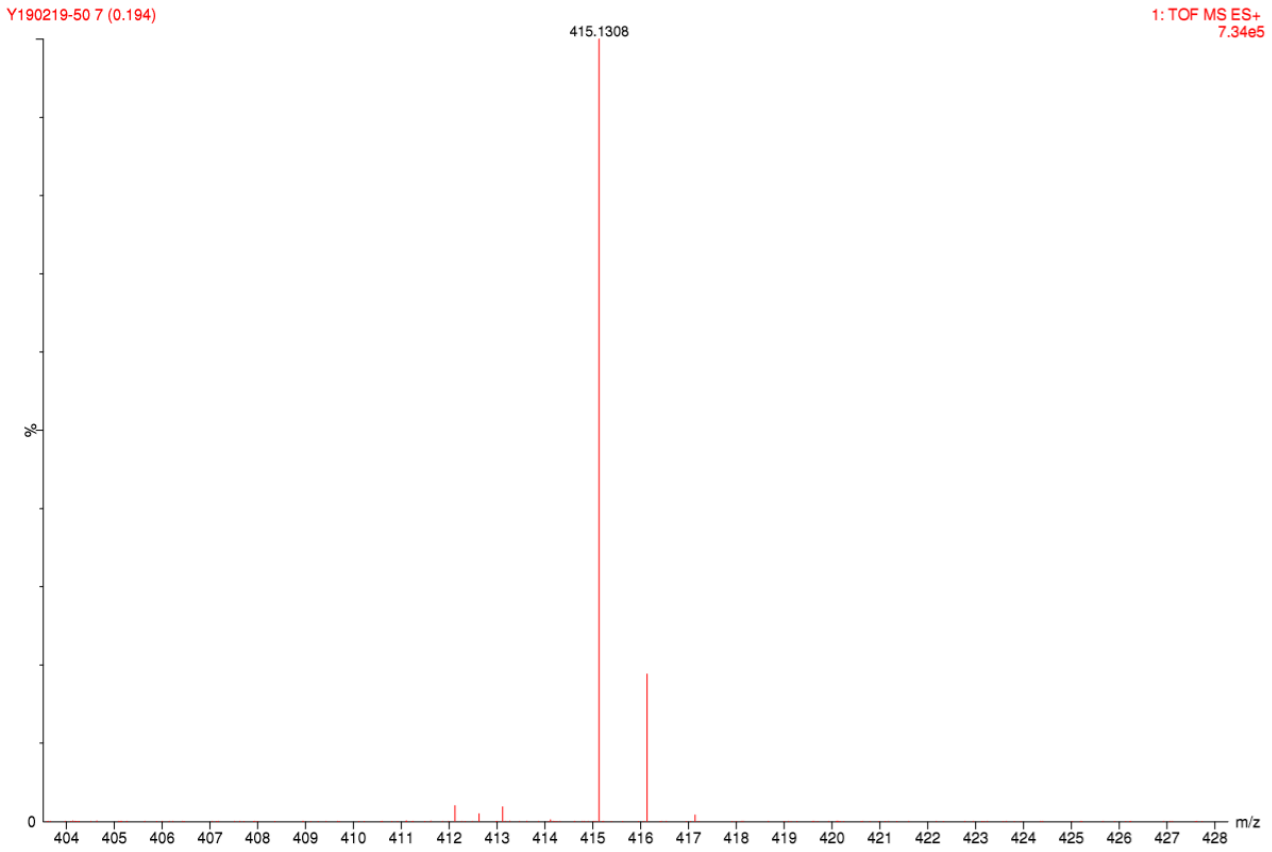

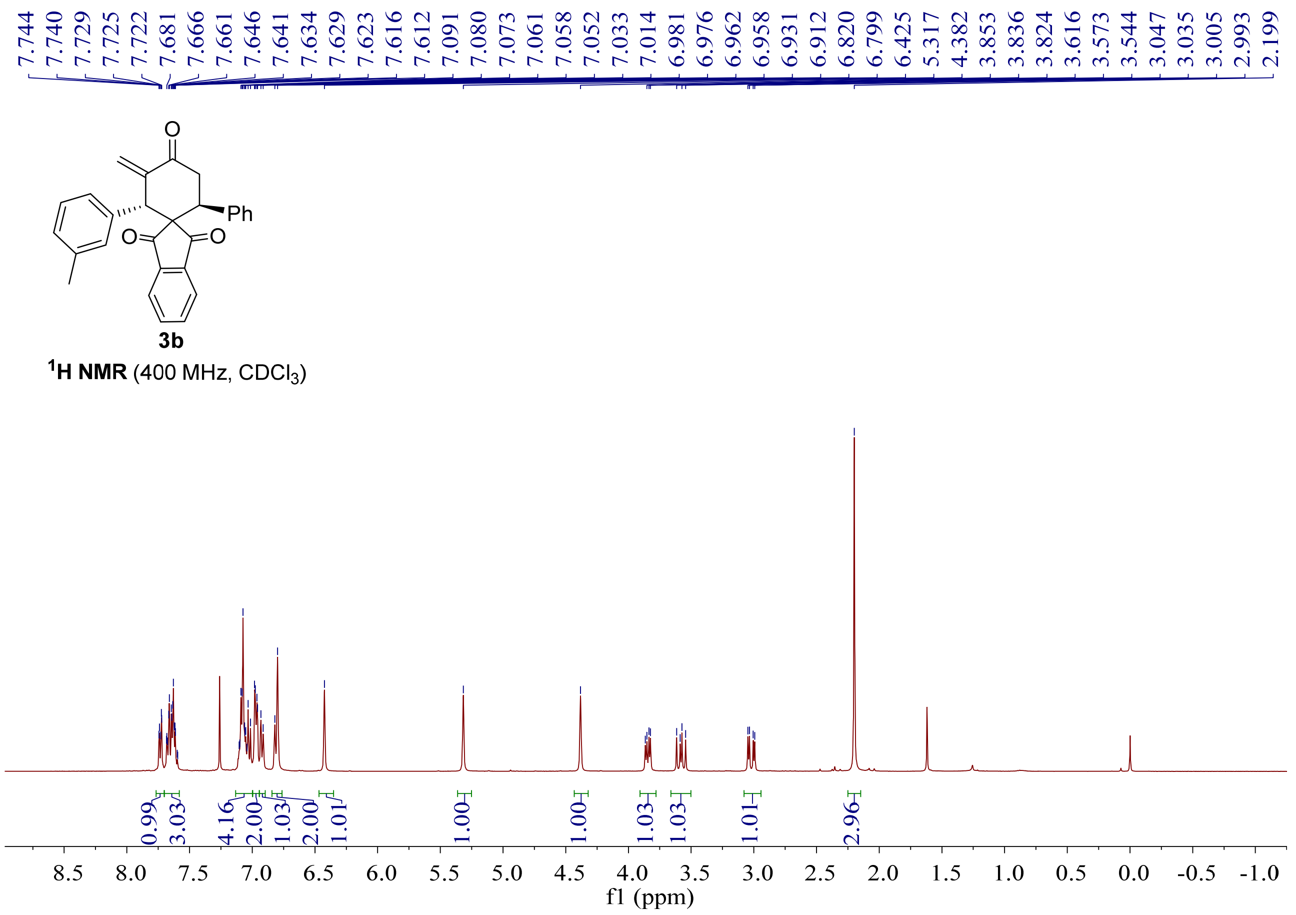

3b

^{1}H NMR (400 MHz, $CDCl_3$)

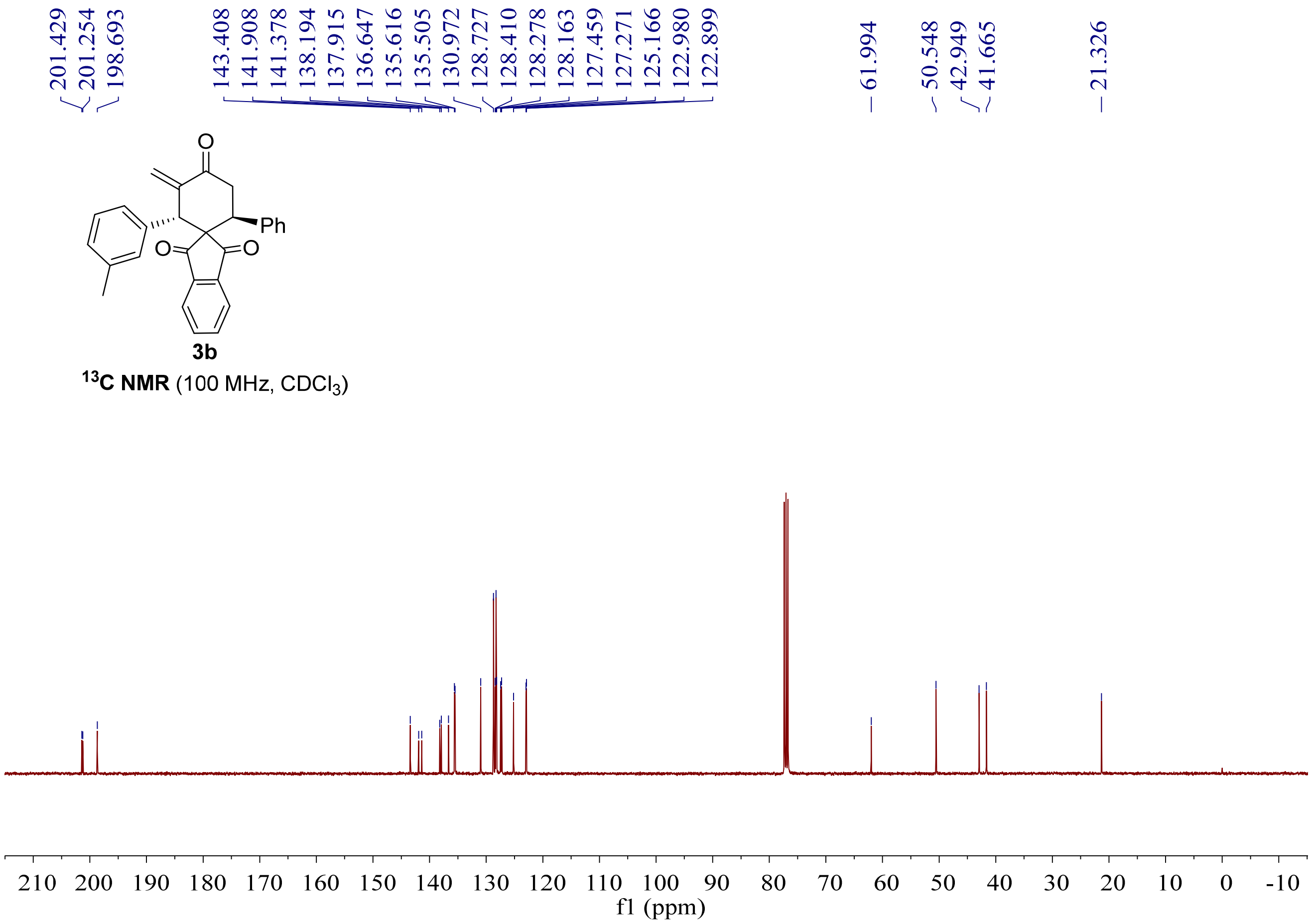

3b

^{13}C NMR (100 MHz, $CDCl_3$)

Daicel Chiral AD-H Column (*i*PrOH/*n*-hexane = 20/80, 1.0 mL/min)

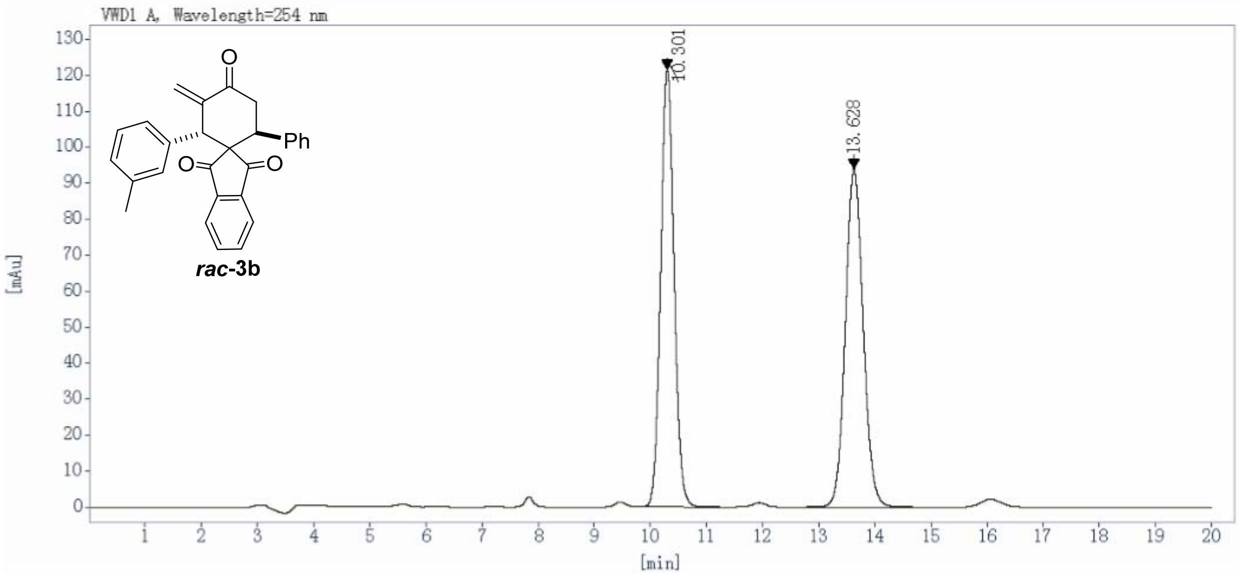

Ret Time [min]	Peak Type	Width [min]	Height [mAU]	Area [mAU*s]	Area [%]
10.301	BB	0.26	121.6093	2009.4531	49.1494
13.628	BB	0.34	94.0892	2079.0024	50.8506
			Totals:	4088.4556	100.0000

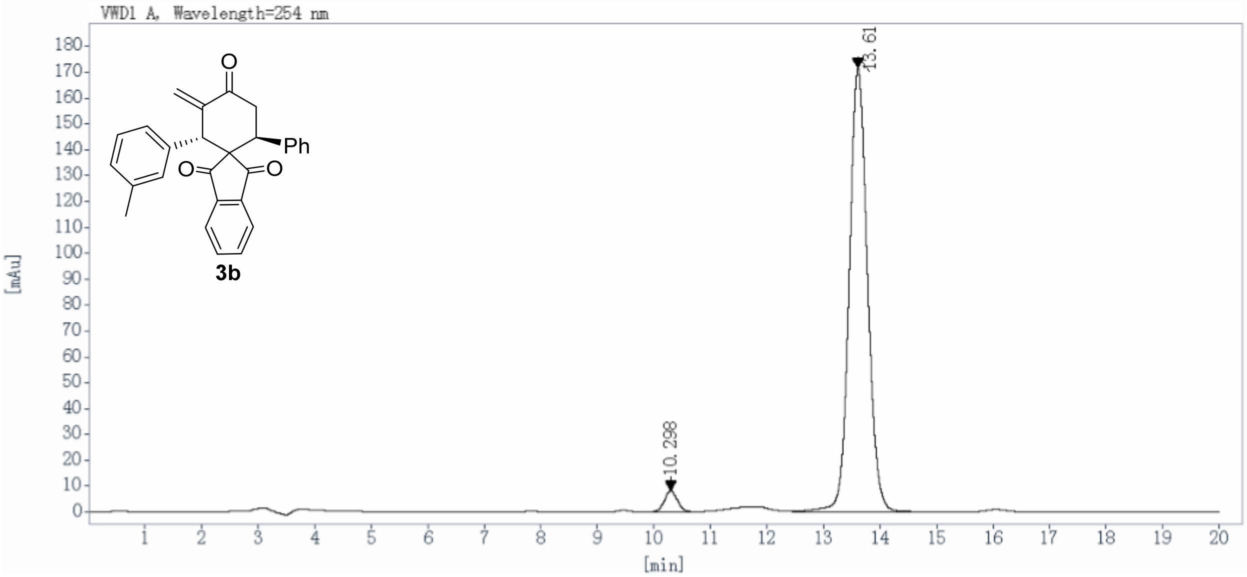

Ret Time [min]	Peak Type	Width [min]	Height [mAU]	Area [mAU*s]	Area [%]
10.298	BB	0.25	8.0437	130.7146	3.2699
13.610	MM	0.38	171.6996	3866.8374	96.7301
			Totals:	3997.5520	100.0000

HRMS (ESI-TOF) m/z: $[M + Na]^+$ Calcd for $C_{28}H_{22}O_3Na^+$ 429.1461; Found 429.1465.

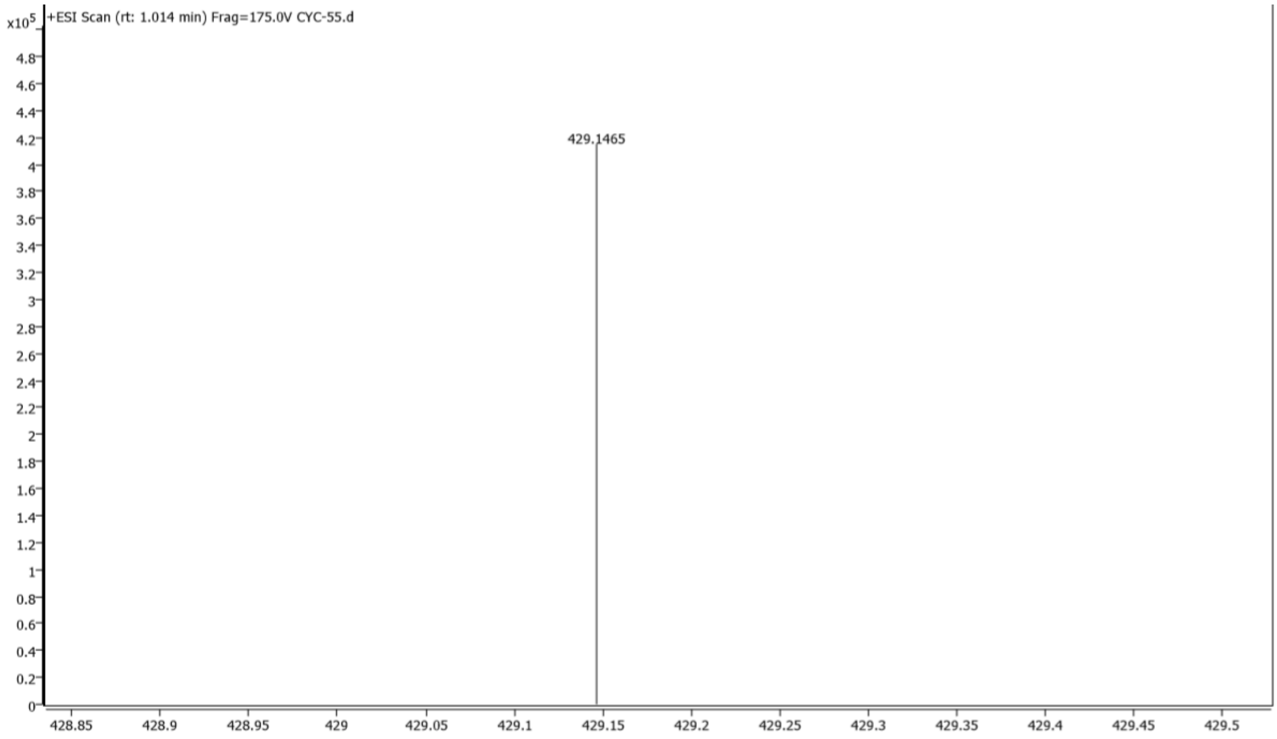

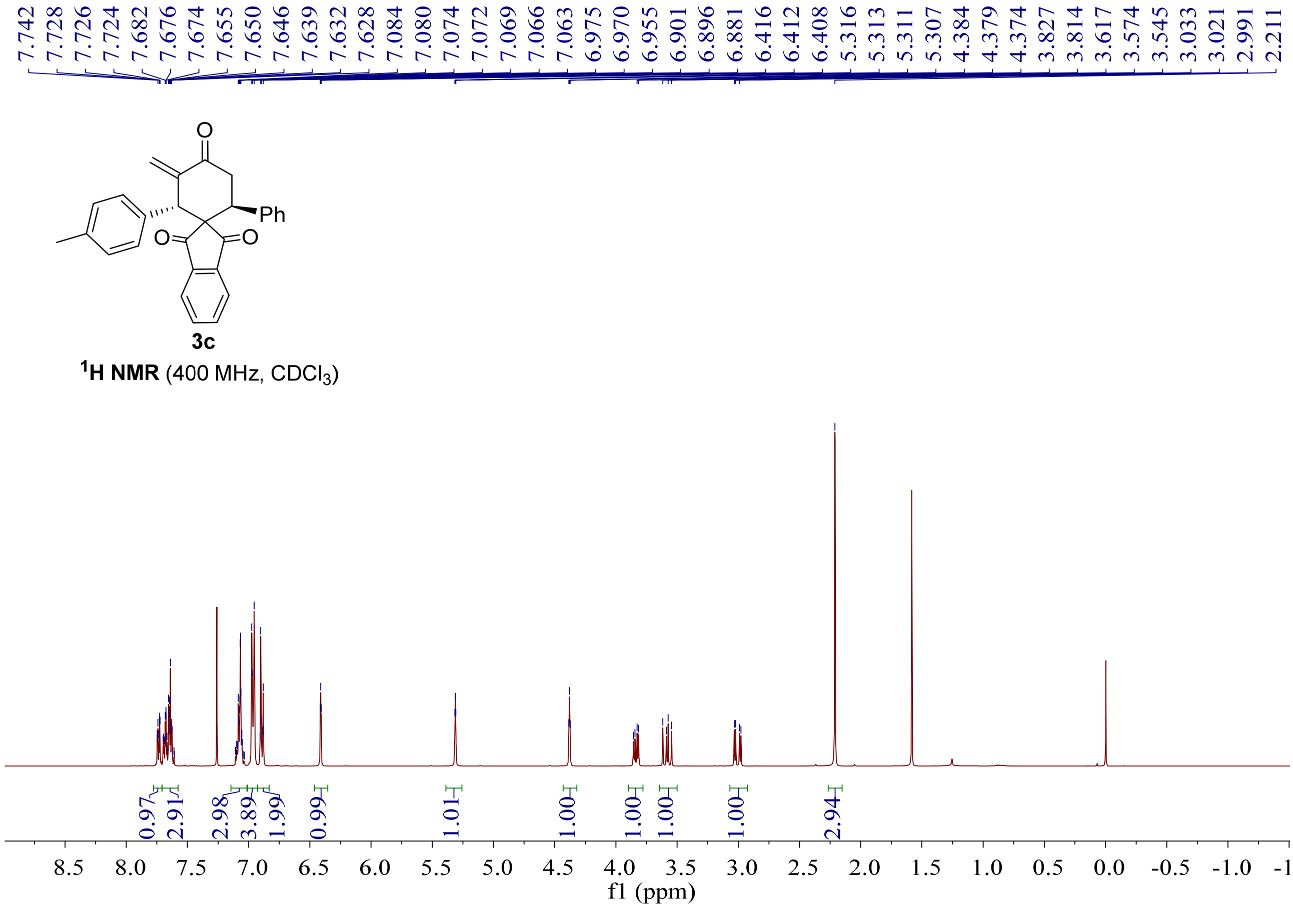
7.742 7.728 7.726 7.724 7.682 7.676 7.674 7.655 7.650 7.646 7.639 7.632 7.628 7.084 7.080 7.074 7.072 7.069 7.066 7.063 6.975 6.970 6.955 6.901 6.896 6.881 6.416 6.412 6.408 5.316 5.313 5.311 5.307 4.384 4.379 4.374 3.827 3.814 3.617 3.574 3.545 3.033 3.021 2.991 2.211
O
Ph
O
O
3c
^{1}H NMR (400 MHz, CDCl$_3$)
0.97 2.91 2.98 3.89 1.99 0.99 1.01 1.00 1.00 1.00 1.00 2.94
8.5 8.0 7.5 7.0 6.5 6.0 5.5 5.0 4.5 4.0 3.5 3.0 2.5 2.0 1.5 1.0 0.5 0.0 -0.5 -1.0 -1
f1 (ppm)

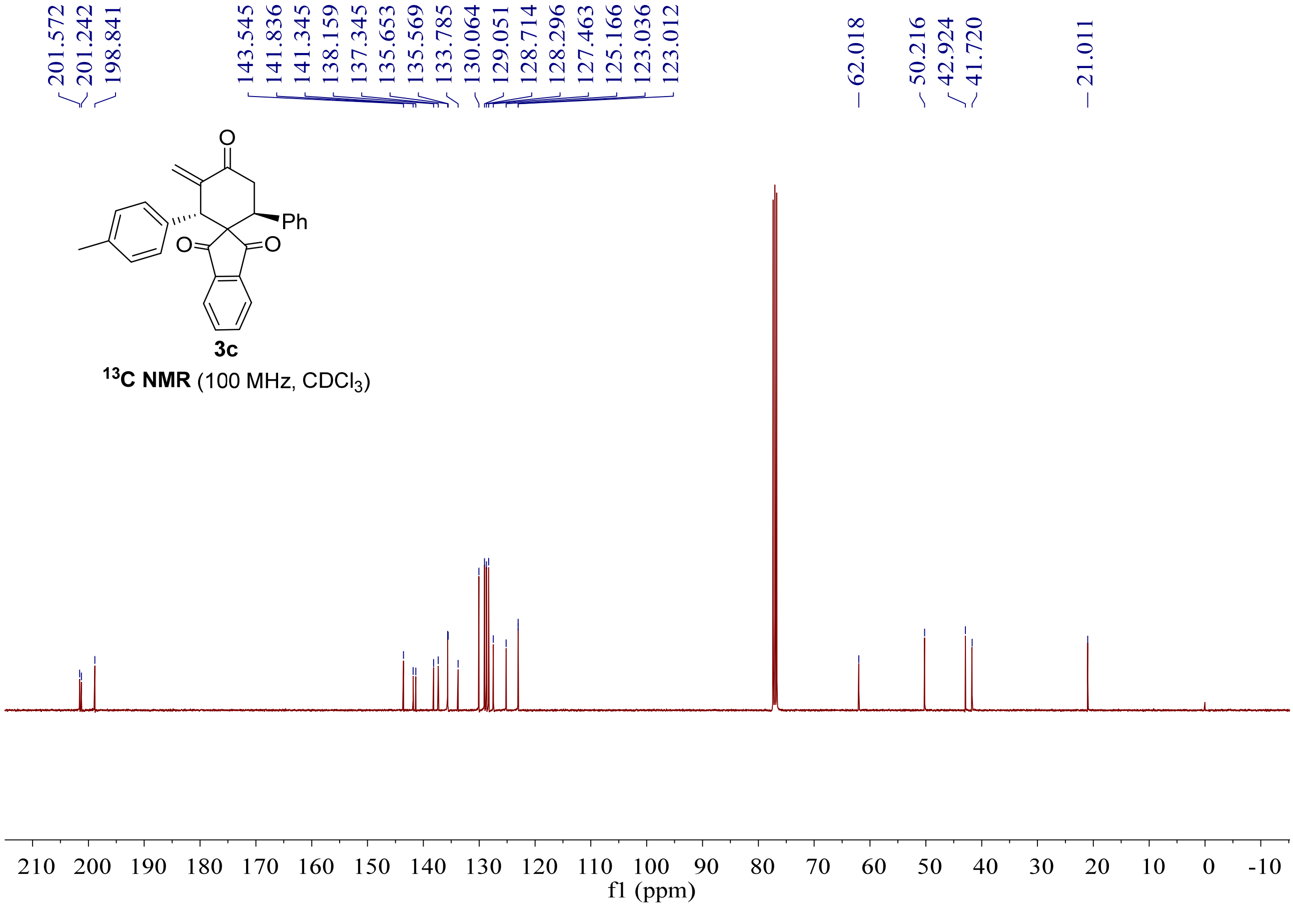
201.572 201.242 198.841 143.545 141.836 141.345 138.159 137.345 135.653 135.569 133.785 130.064 129.051 128.714 128.296 127.463 125.166 123.036 123.012 62.018 50.216 42.924 41.720 21.011
O
Ph
O
O
3c
^{13}C NMR (100 MHz, CDCl$_3$)
210 200 190 180 170 160 150 140 130 120 110 100 90 80 70 60 50 40 30 20 10 0 -10
f1 (ppm)

Daicel Chiral AD-H Column (*i*PrOH/*n*-hexane = 20/80, 1.0 mL/min)

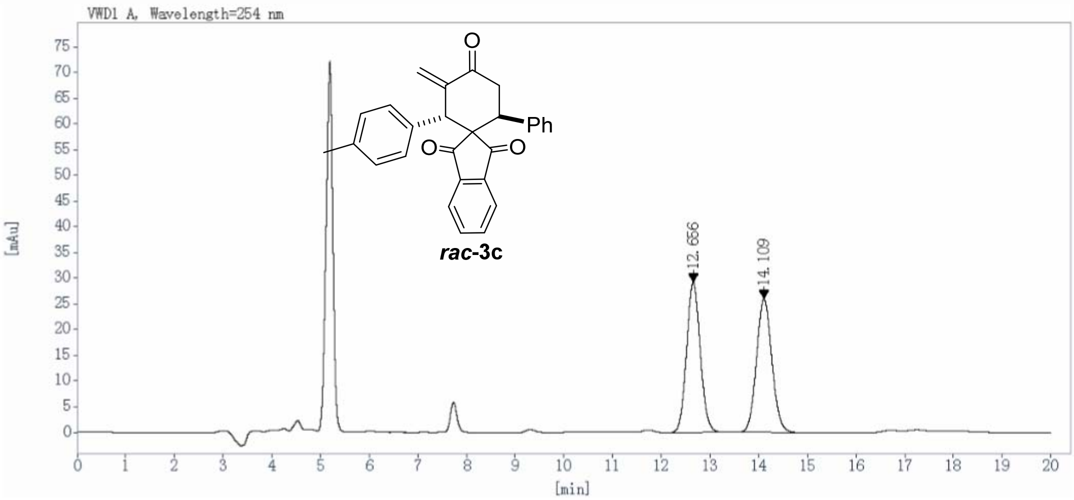

Ret Time [min]	Peak Type	Width [min]	Height [mAU]	Area [mAU*s]	Area [%]
12.656	BB	0.30	29.2135	573.2819	49.9790
14.109	BB	0.34	25.9890	573.7645	50.0210
			Totals:	1147.0463	100.0000

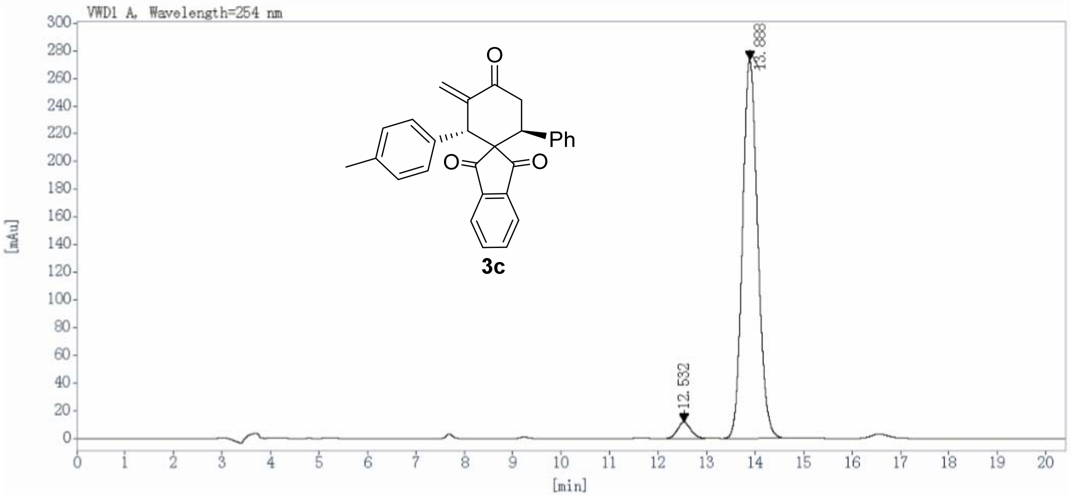

Ret Time [min]	Peak Type	Width [min]	Height [mAU]	Area [mAU*s]	Area [%]
12.532	MF	0.32	11.5854	223.3806	3.6164
13.888	BB	0.34	273.5276	5953.5215	96.3836
			Totals:	6176.9021	100.0000

HRMS (ESI-TOF) m/z: $[M + Na]^+$ Calcd for $C_{28}H_{22}O_3Na^+$ 429.1461; Found 429.1465.

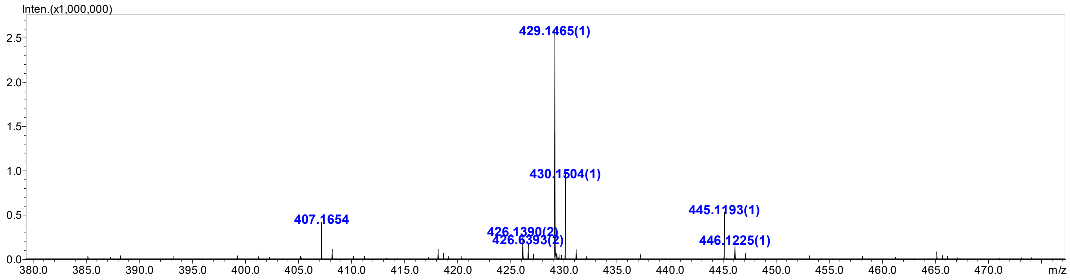

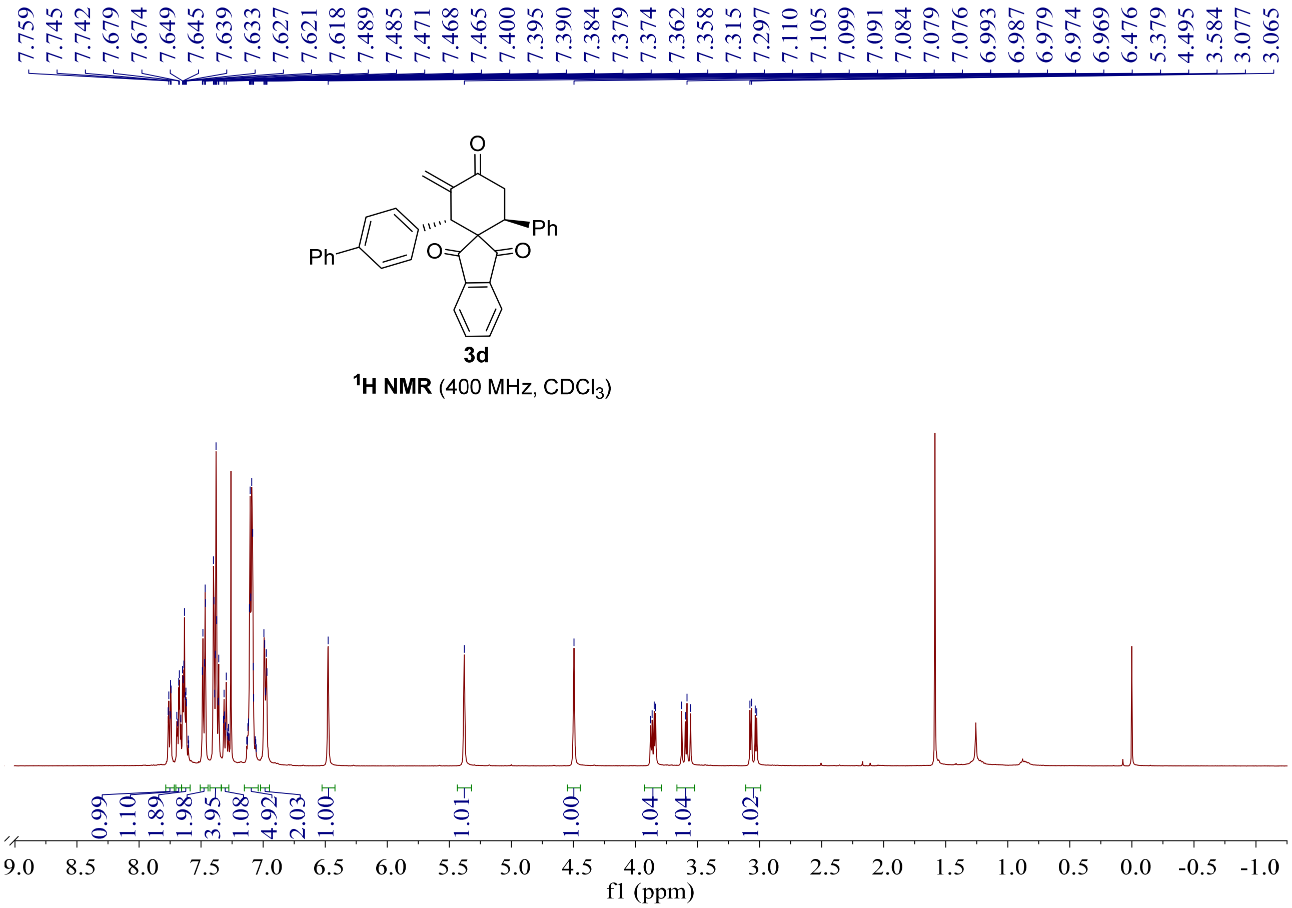
7.759 7.745 7.742 7.679 7.674 7.649 7.645 7.639 7.633 7.627 7.621 7.618 7.489 7.485 7.471 7.468 7.465 7.400 7.395 7.390 7.384 7.379 7.374 7.362 7.358 7.315 7.297 7.110 7.105 7.099 7.091 7.084 7.079 7.076 6.993 6.987 6.979 6.974 6.969 6.476 5.379 4.495 3.584 3.077 3.065
O
Ph
Ph
O
O
3d
^{1}H NMR (400 MHz, $CDCl_3$)
0.99 1.10 1.89 1.98 3.95 1.08 4.92 2.03 1.00 1.01 1.00 1.04 1.04 1.02
9.0 8.5 8.0 7.5 7.0 6.5 6.0 5.5 5.0 4.5 4.0 3.5 3.0 2.5 2.0 1.5 1.0 0.5 0.0 -0.5 -1.0
f1 (ppm)

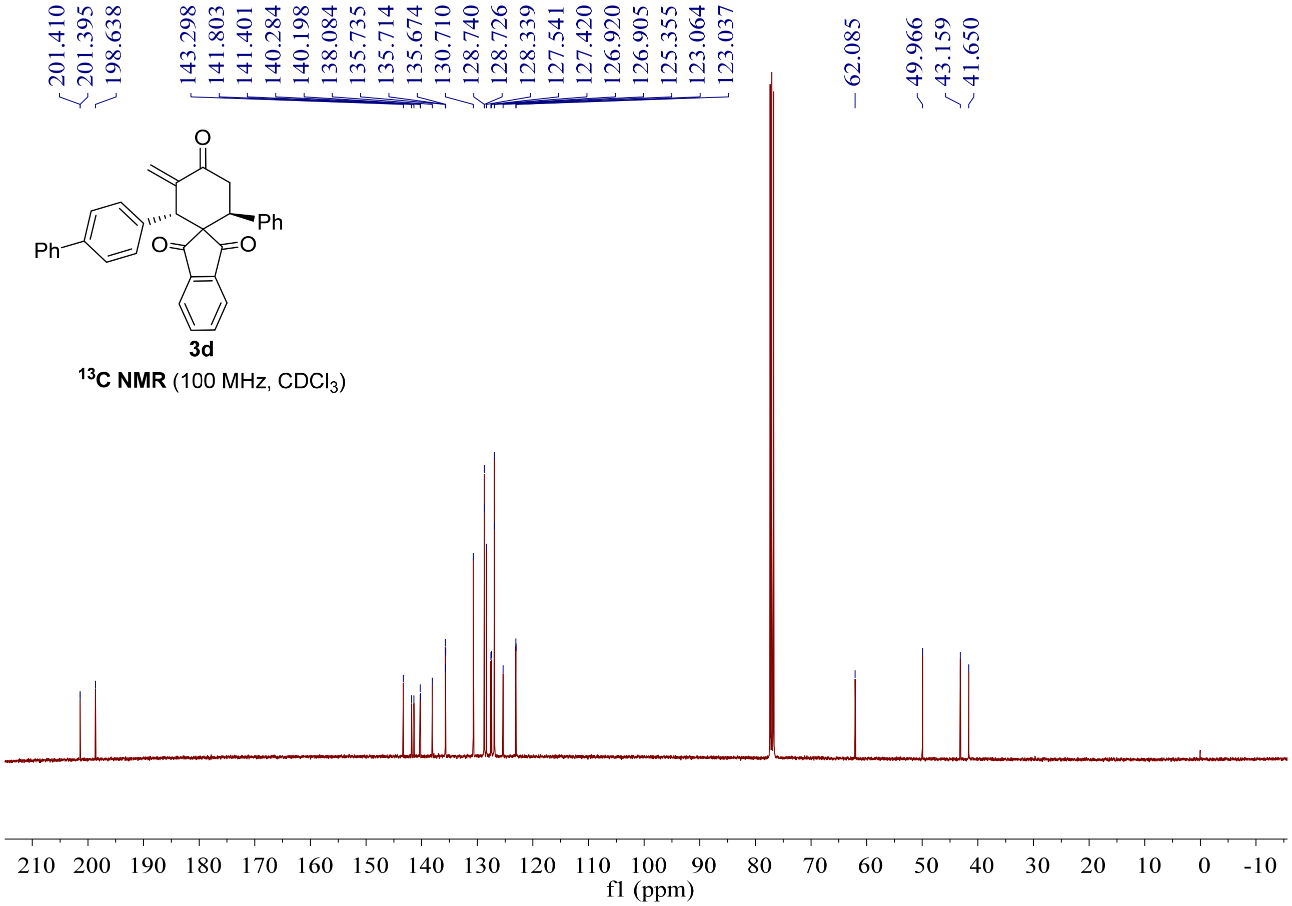
201.410 201.395 198.638
143.298 141.803 141.401 140.284 140.198 138.084 135.735 135.714 135.674 130.710 128.740 128.726 128.339 127.541 127.420 126.920 126.905 125.355 123.064 123.037
62.085
49.966 43.159 41.650
O
Ph
Ph
O
O
3d
^{13}C NMR (100 MHz, $CDCl_3$)
210 200 190 180 170 160 150 140 130 120 110 100 90 80 70 60 50 40 30 20 10 0 -10
f1 (ppm)

Daicel Chiral IA Column (*i*PrOH/*n*-hexane = 20/80, 1.0 mL/min)

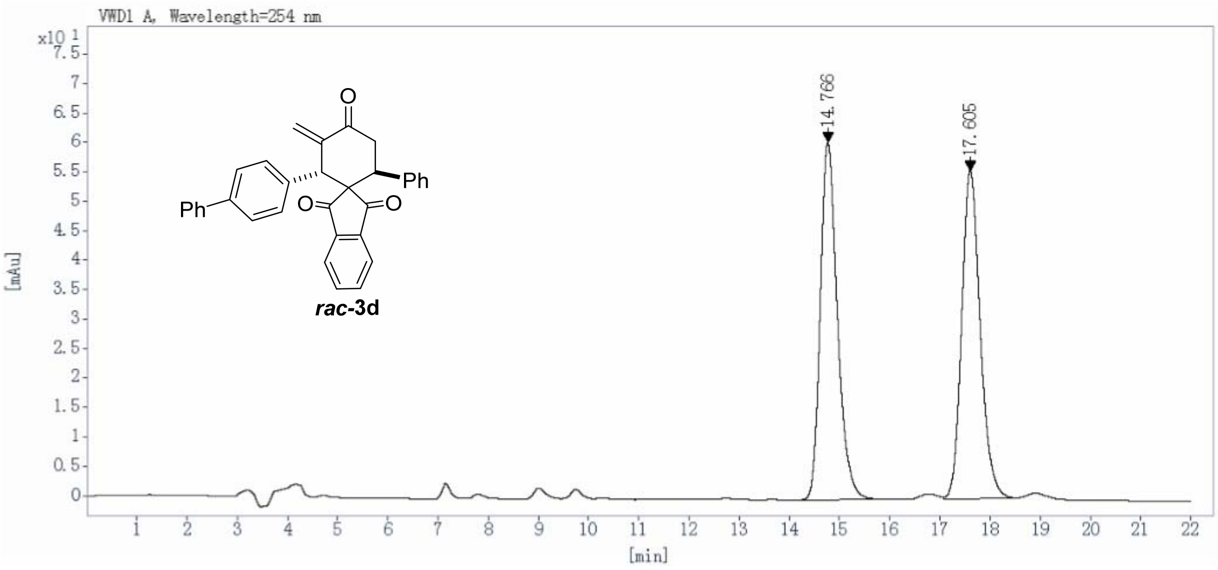

Ret Time [min]	Peak Type	Width [min]	Height [mAU]	Area [mAU*s]	Area [%]
14.766	BB	0.36	60.6653	1431.9075	50.1354
17.605	FM	0.43	55.7125	1424.1742	49.8646
			Totals:	2856.0817	100.0000

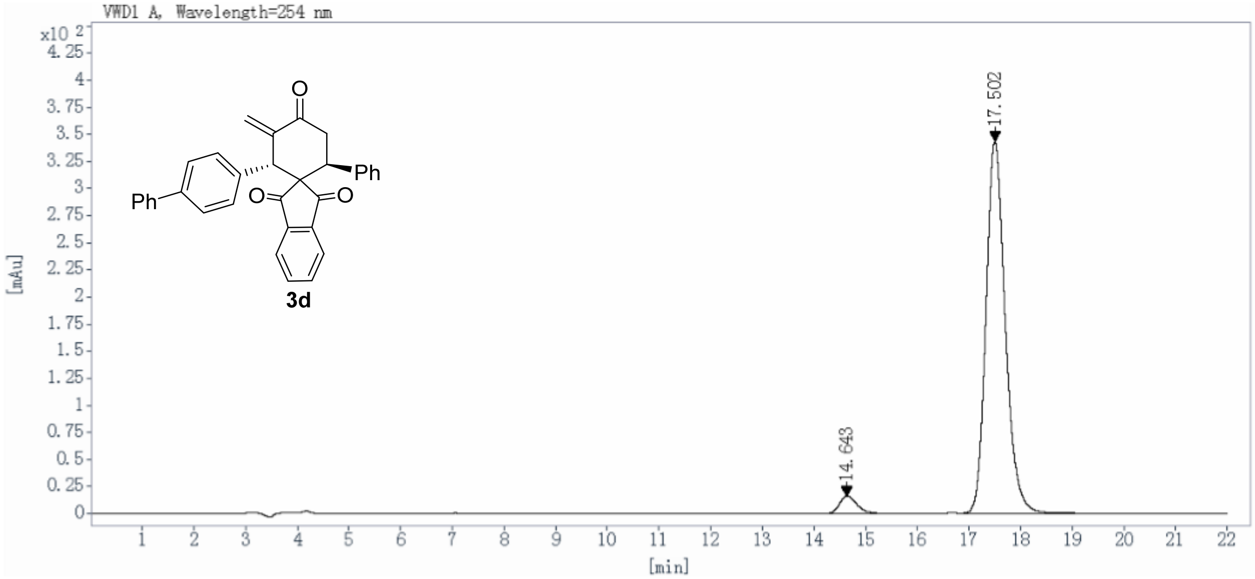

Ret Time [min]	Peak Type	Width [min]	Height [mAU]	Area [mAU*s]	Area [%]
14.643	BB	0.36	16.4071	387.8263	4.1790
17.502	BB	0.40	342.8974	8892.6143	95.8210
			Totals:	9280.4406	100.0000

HRMS (ESI-TOF) m/z: [M + Na]$^+$Calcd for $C_{33}H_{24}O_3Na^+$ 491.1618; Found 491.1620.

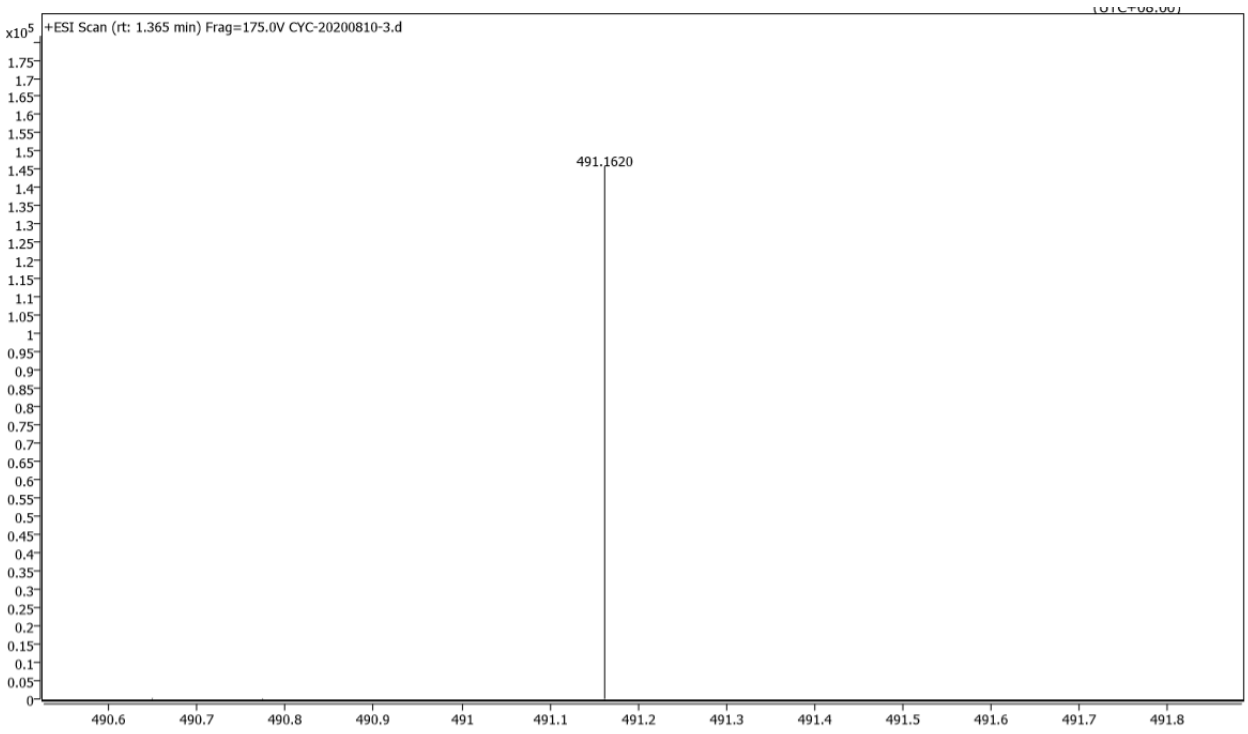

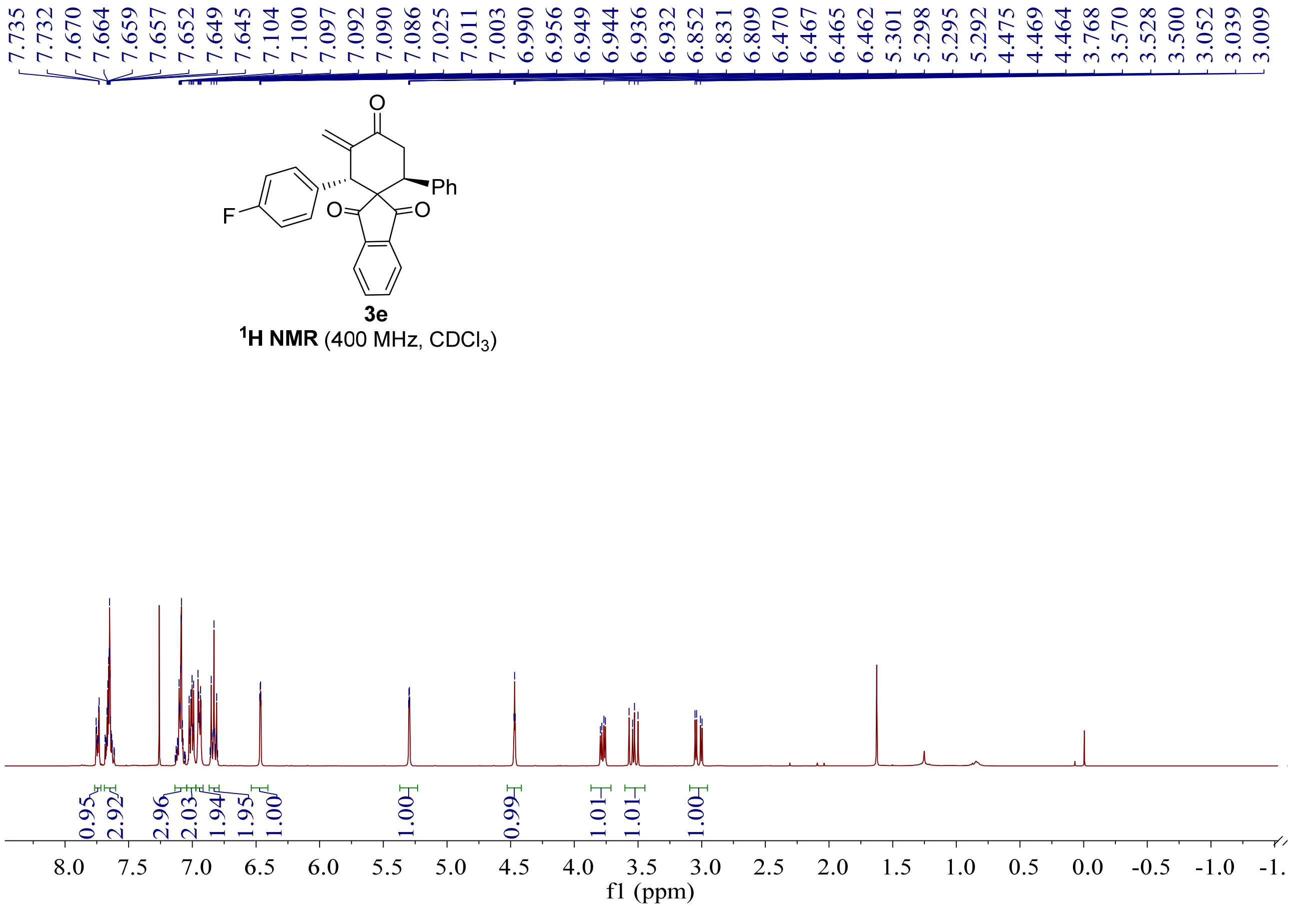
7.735 7.732 7.670 7.664 7.659 7.657 7.652 7.649 7.645 7.104 7.100 7.097 7.092 7.090 7.086 7.025 7.011 7.003 6.990 6.956 6.949 6.944 6.936 6.932 6.852 6.831 6.809 6.470 6.467 6.465 6.462 5.301 5.298 5.295 5.292 4.475 4.469 4.464 3.768 3.570 3.528 3.500 3.052 3.039 3.009
O
Ph
F
O
O
3e
1H NMR (400 MHz, CDCl3)
0.95 2.92 2.96 2.03 1.94 1.95 1.00 1.00 0.99 1.01 1.01 1.00
f1 (ppm)

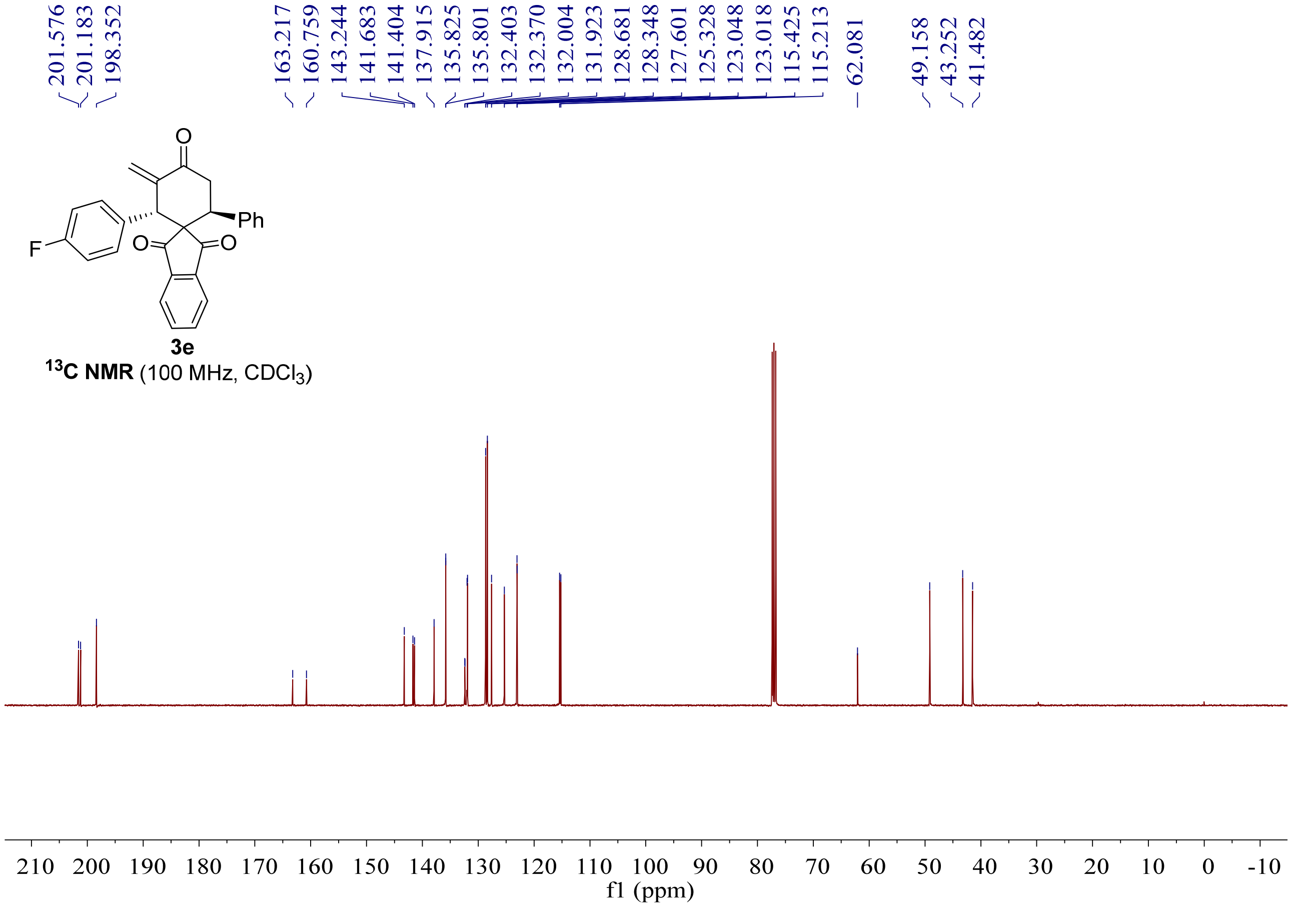
201.576 201.183 198.352 163.217 160.759 143.244 141.683 141.404 137.915 135.825 135.801 132.403 132.370 132.004 131.923 128.681 128.348 127.601 125.328 123.048 123.018 115.425 115.213 62.081 49.158 43.252 41.482
O
Ph
F
O
O
3e
13C NMR (100 MHz, CDCl3)
f1 (ppm)

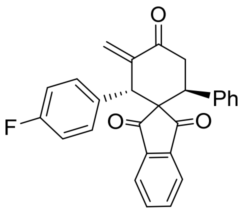

3e

^{19}F NMR (376 MHz, $CDCl_3$)

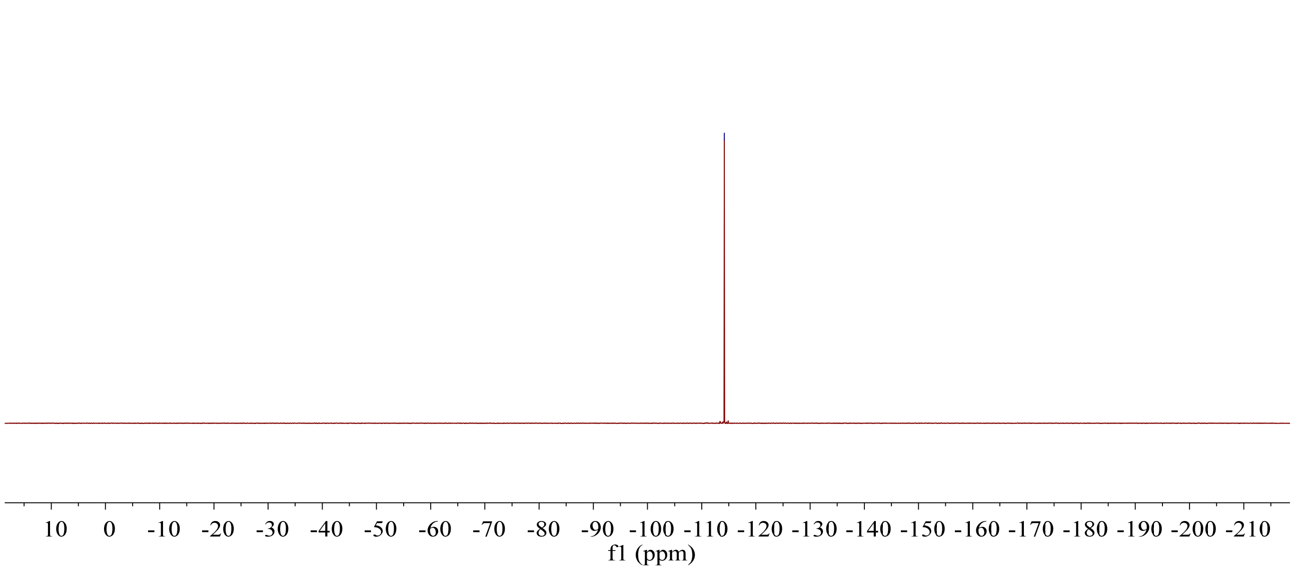

Daicel Chiral AD-H Column (*i*PrOH/*n*-hexane = 20/80, 1.0 mL/min)

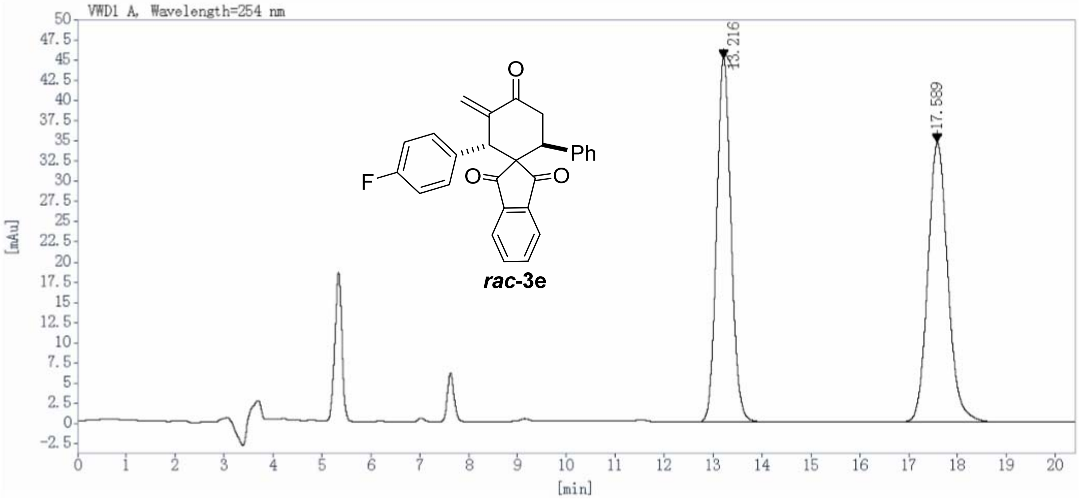

Ret Time [min]	Peak Type	Width [min]	Height [mAU]	Area [mAU*s]	Area [%]
13.216	BB	0.32	45.1503	922.6016	48.3643
17.589	BB	0.44	34.6755	985.0057	51.6357
			Totals:	1907.6072	100.0000

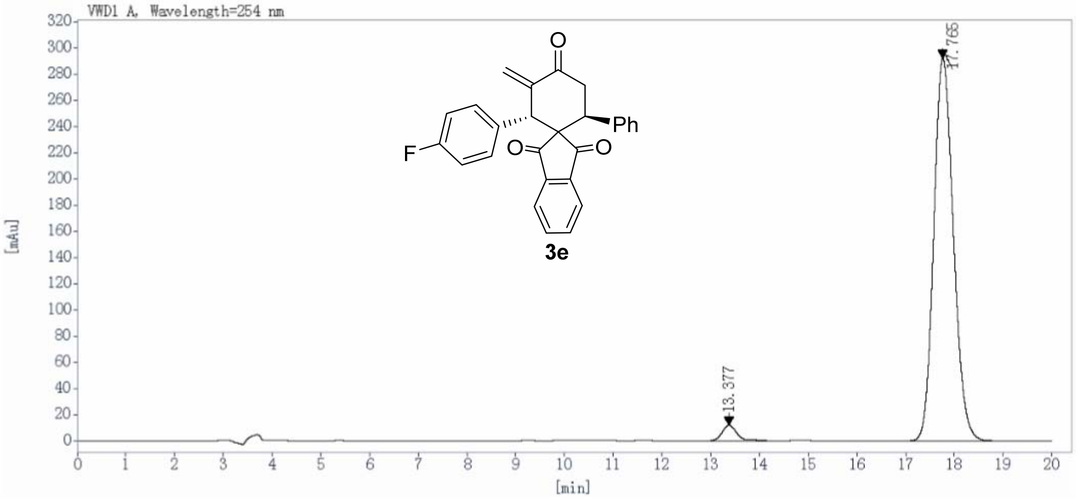

Ret Time [min]	Peak Type	Width [min]	Height [mAU]	Area [mAU*s]	Area [%]
13.377	BB	0.33	11.9404	261.4232	3.1008
17.765	BB	0.43	292.0090	8169.5376	96.8992
			Totals:	8430.9608	100.0000

HRMS (ESI-TOF) m/z: $[M + Na]^+$ Calcd for $C_{27}H_{19}FNaO_3^+$ 433.1210; Found 433.1207.

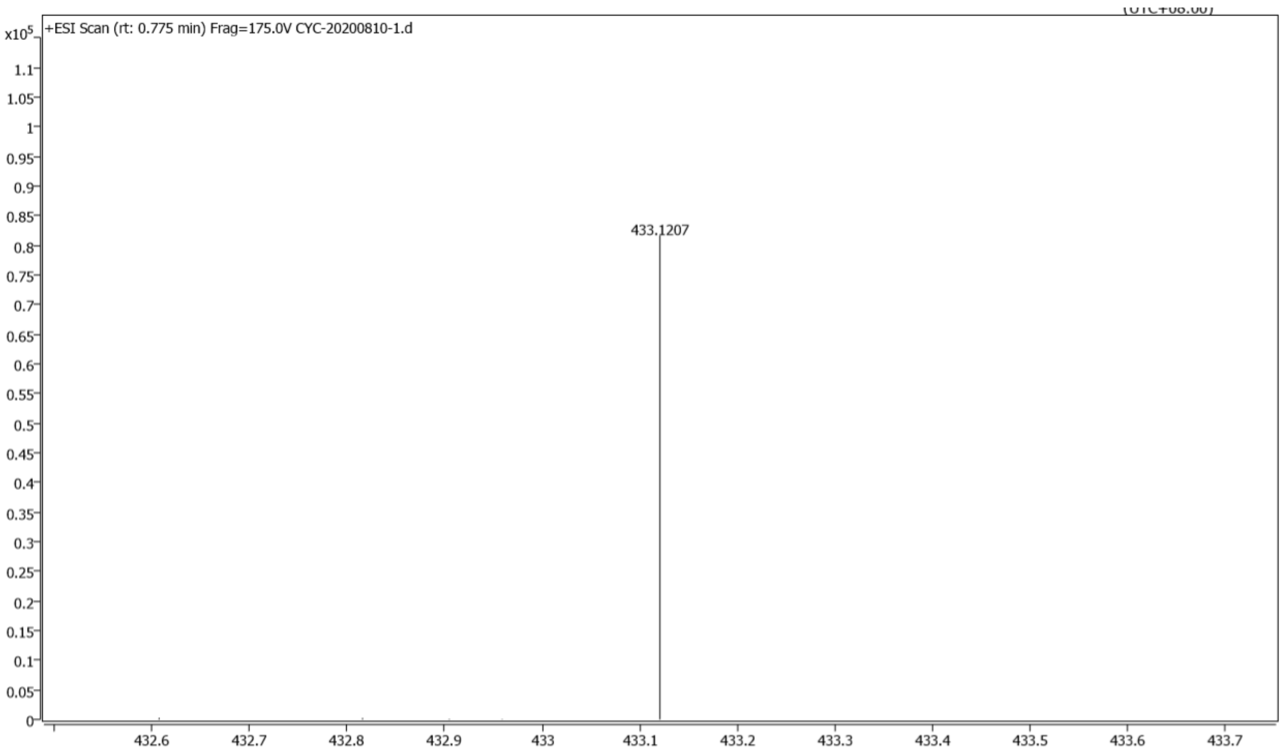

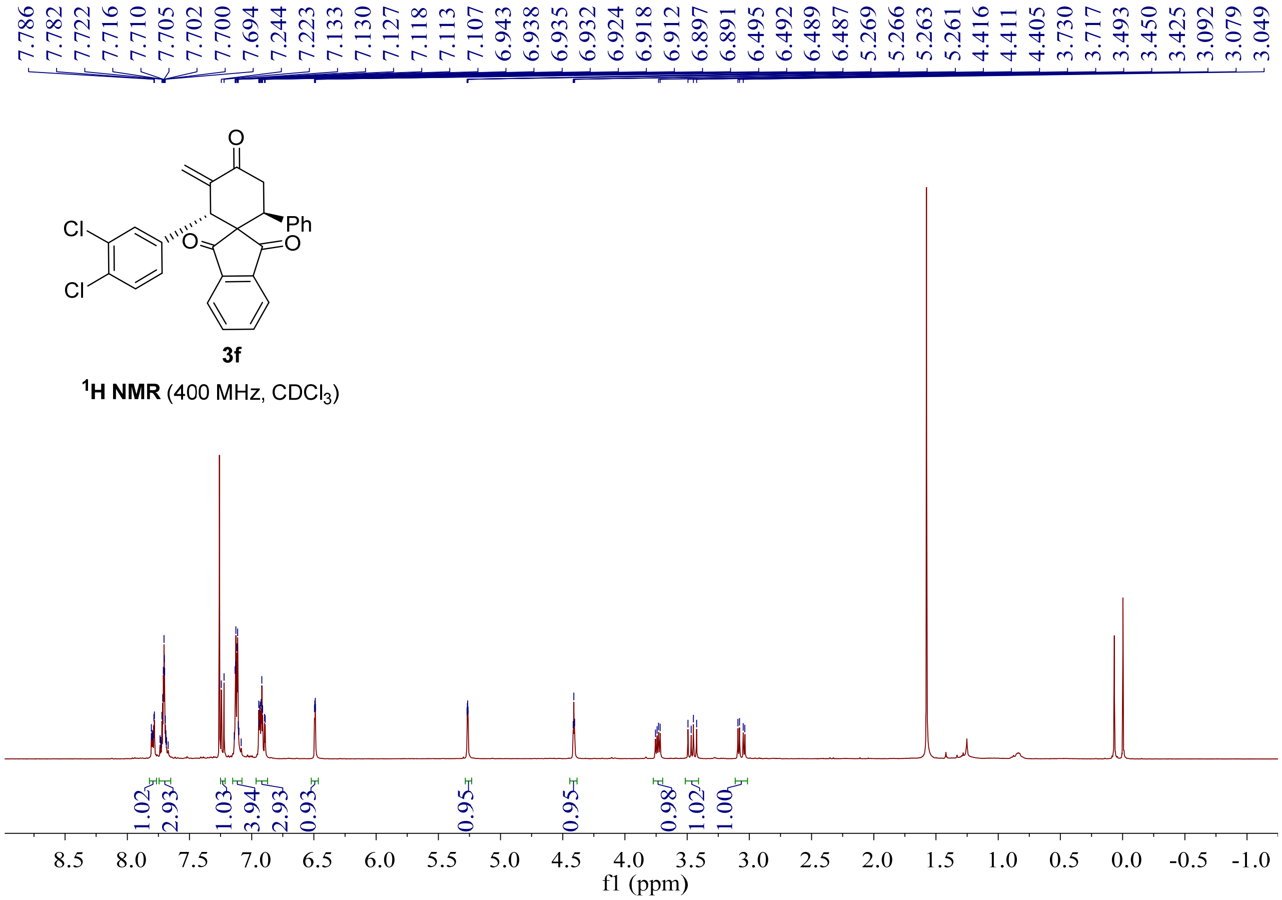
7.786 7.782 7.722 7.716 7.710 7.705 7.702 7.700 7.694 7.244 7.223 7.133 7.130 7.127 7.118 7.113 7.107 6.943 6.938 6.935 6.932 6.924 6.918 6.912 6.897 6.891 6.495 6.492 6.489 6.487 5.269 5.266 5.263 5.261 4.416 4.411 4.405 3.730 3.717 3.493 3.450 3.425 3.092 3.079 3.049
O
Cl
Cl
Ph
O
O
3f
1H NMR (400 MHz, CDCl3)
1.02 2.93 1.03 3.94 2.93 0.93 0.95 0.95 0.98 1.02 1.00
8.5 8.0 7.5 7.0 6.5 6.0 5.5 5.0 4.5 4.0 3.5 3.0 2.5 2.0 1.5 1.0 0.5 0.0 -0.5 -1.0
f1 (ppm)

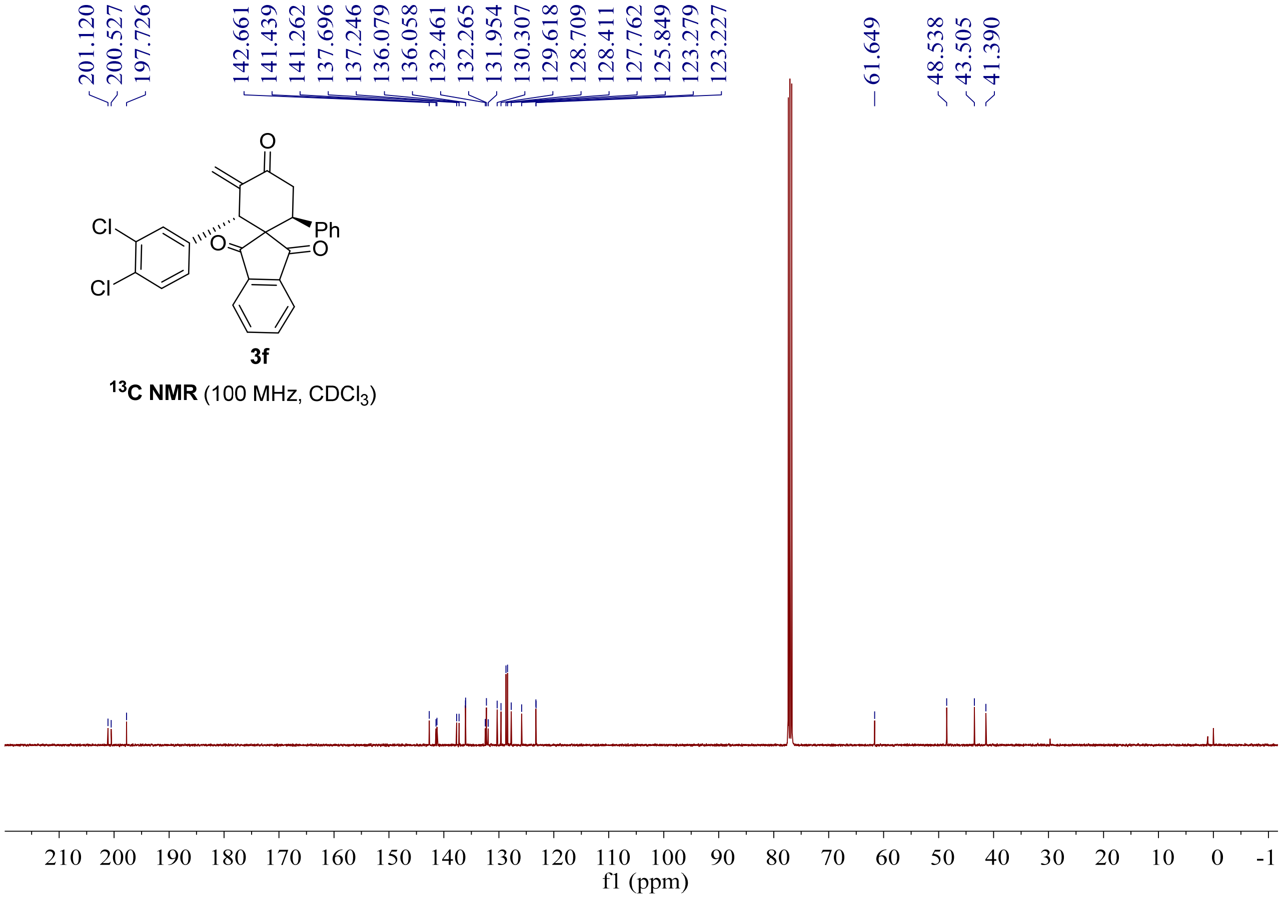
201.120 200.527 197.726
142.661 141.439 141.262 137.696 137.246 136.079 136.058 132.461 132.265 131.954 130.307 129.618 128.709 128.411 127.762 125.849 123.279 123.227
61.649
48.538 43.505 41.390
O
Cl
Cl
Ph
O
O
3f
13C NMR (100 MHz, CDCl3)
210 200 190 180 170 160 150 140 130 120 110 100 90 80 70 60 50 40 30 20 10 0 -1
f1 (ppm)

Daicel Chiral IA Column (*i*PrOH/ *n*-hexane = 20/80, 1.0 mL/min)

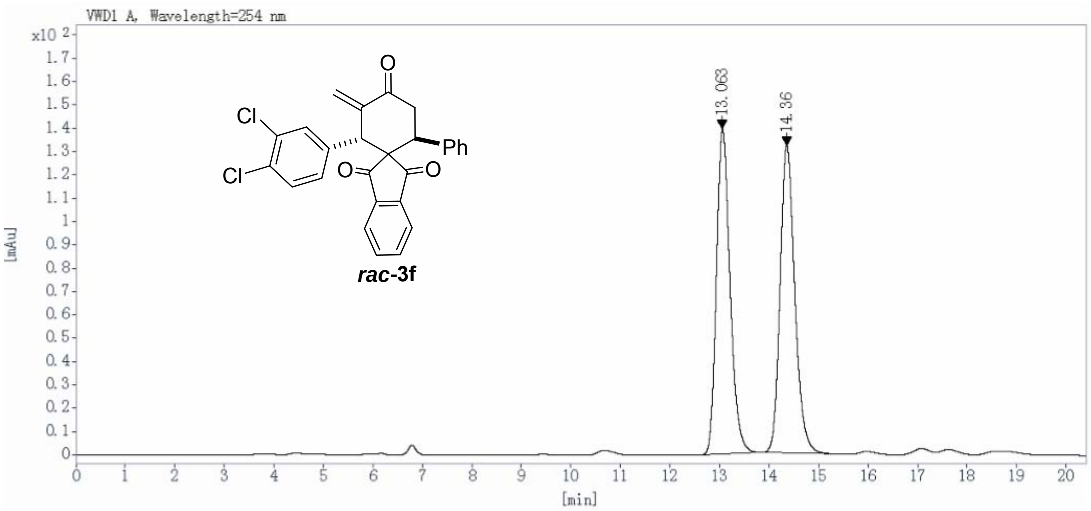

Ret Time [min]	Peak Type	Width [min]	Height [mAU]	Area [mAU*s]	Area [%]
13.063	BB	0.29	139.4626	2662.9590	49.4179
14.360	BB	0.32	131.9399	2725.6902	50.5821
			Totals:	5388.6492	100.0000

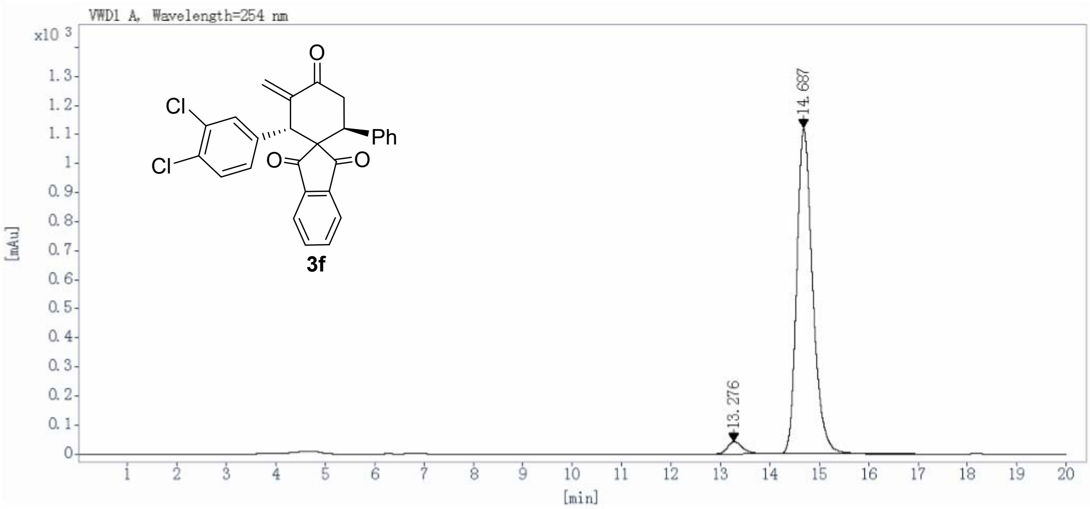

Ret Time [min]	Peak Type	Width [min]	Height [mAU]	Area [mAU*s]	Area [%]
13.276	BB	0.32	42.5270	897.3829	3.4413
14.687	BB	0.34	1121.1852	25179.2949	96.5587
			Totals:	26076.6779	100.0000

HRMS (ESI-TOF) m/z: $[M + H]^+$ Calcd for $C_{27}H_{19}Cl_2O_3^+$ 461.0706 (^{35}Cl*2) and 463.0676 ($^{35}Cl + {}^{37}Cl$); Found 461.0700 (^{35}Cl*2) and 463.0675 ($^{35}Cl + {}^{37}Cl$).

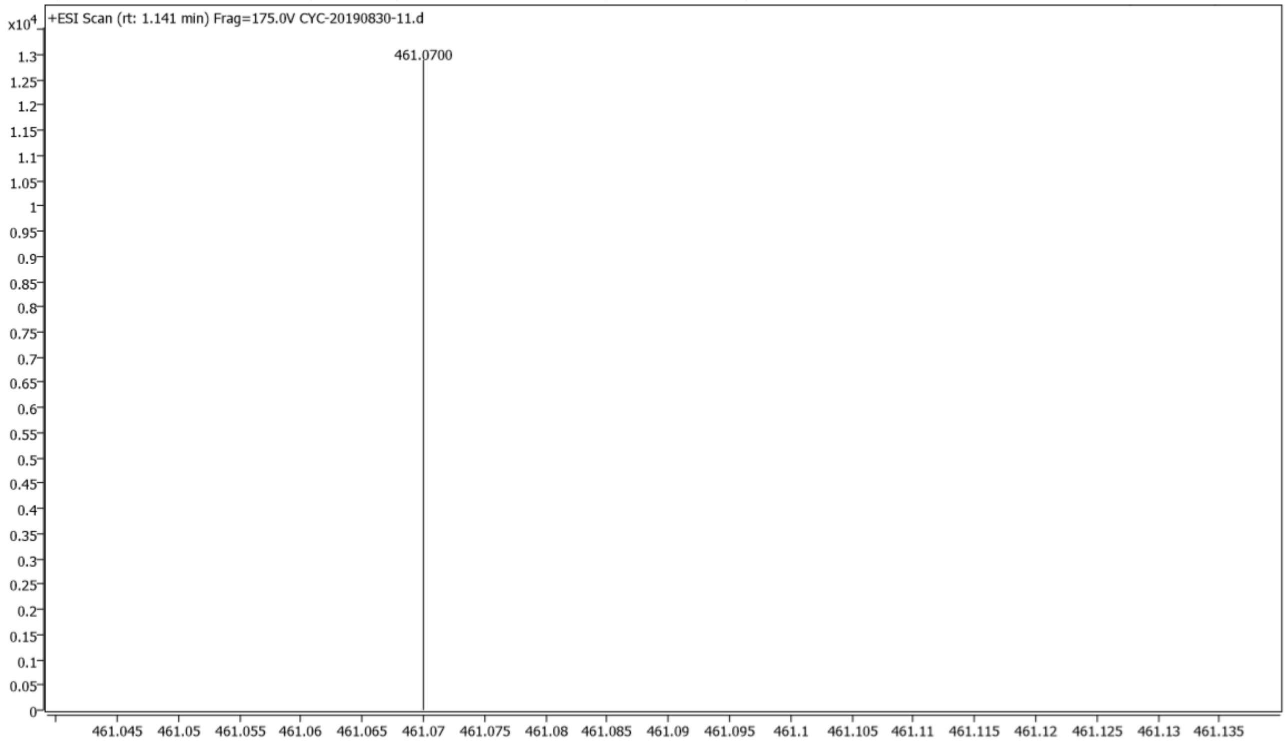

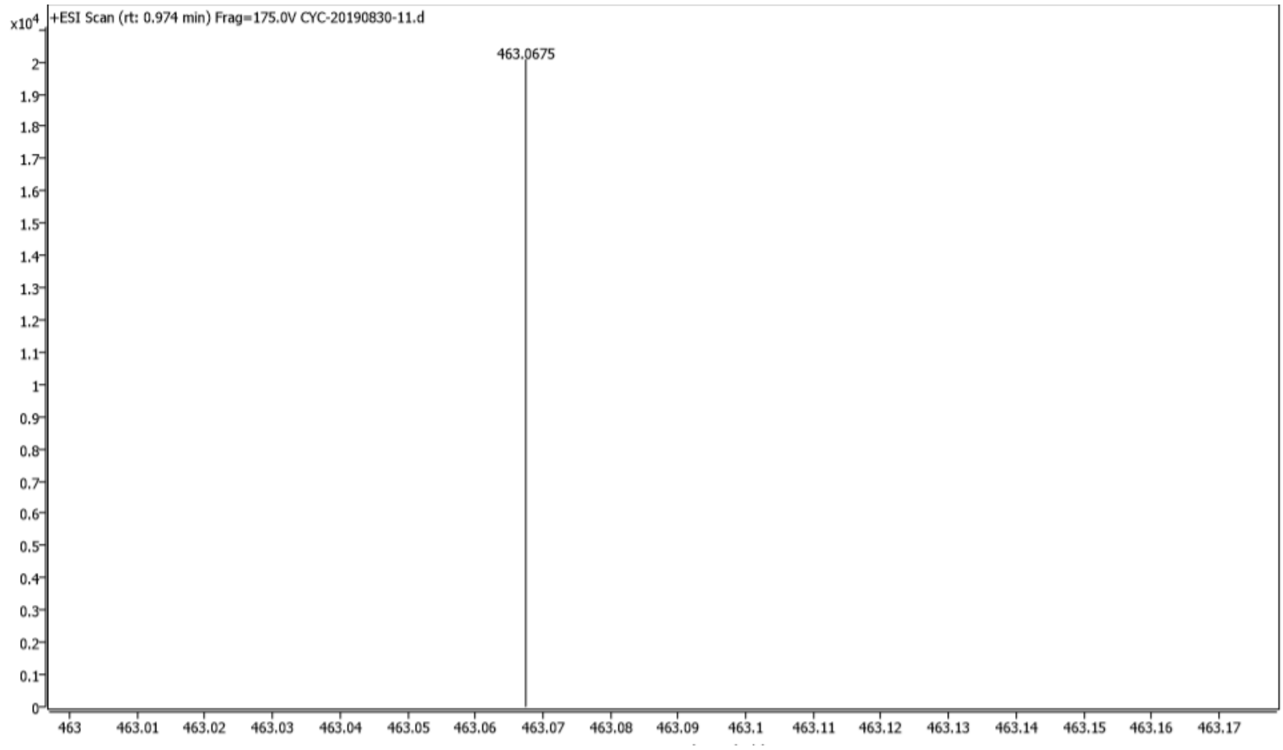

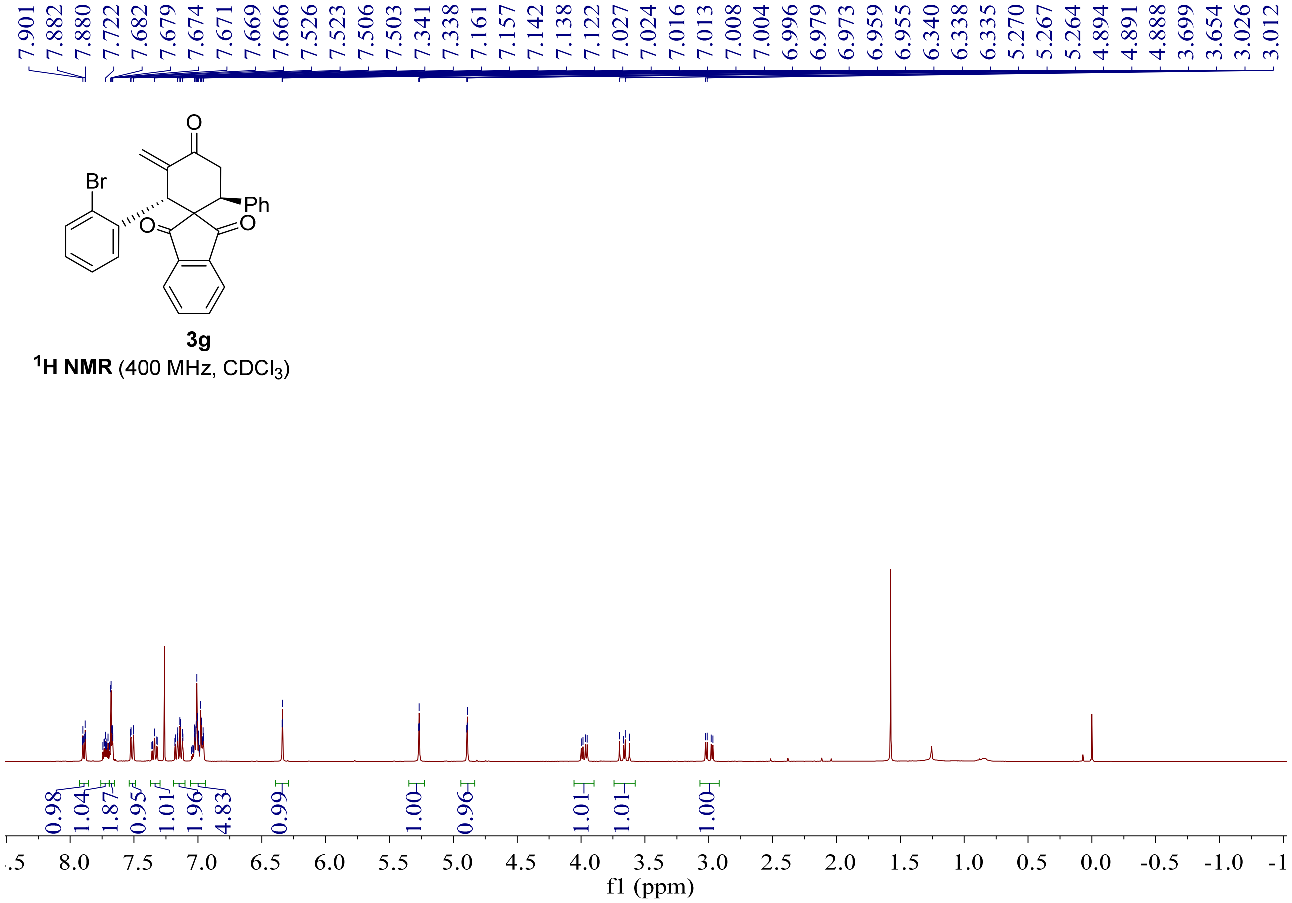
7.901
7.882
7.880
7.722
7.682
7.679
7.674
7.671
7.669
7.666
7.526
7.523
7.506
7.503
7.341
7.338
7.161
7.157
7.142
7.138
7.122
7.027
7.024
7.016
7.013
7.008
7.004
6.996
6.979
6.973
6.959
6.955
6.340
6.338
6.335
5.270
5.267
5.264
4.894
4.891
4.888
3.699
3.654
3.026
3.012
Br
O
Ph
O
O
3g
1H NMR (400 MHz, CDCl3)
0.98
1.04
1.87
0.95
1.01
1.96
4.83
0.99
1.00
0.96
1.01
1.01
1.00
f1 (ppm)

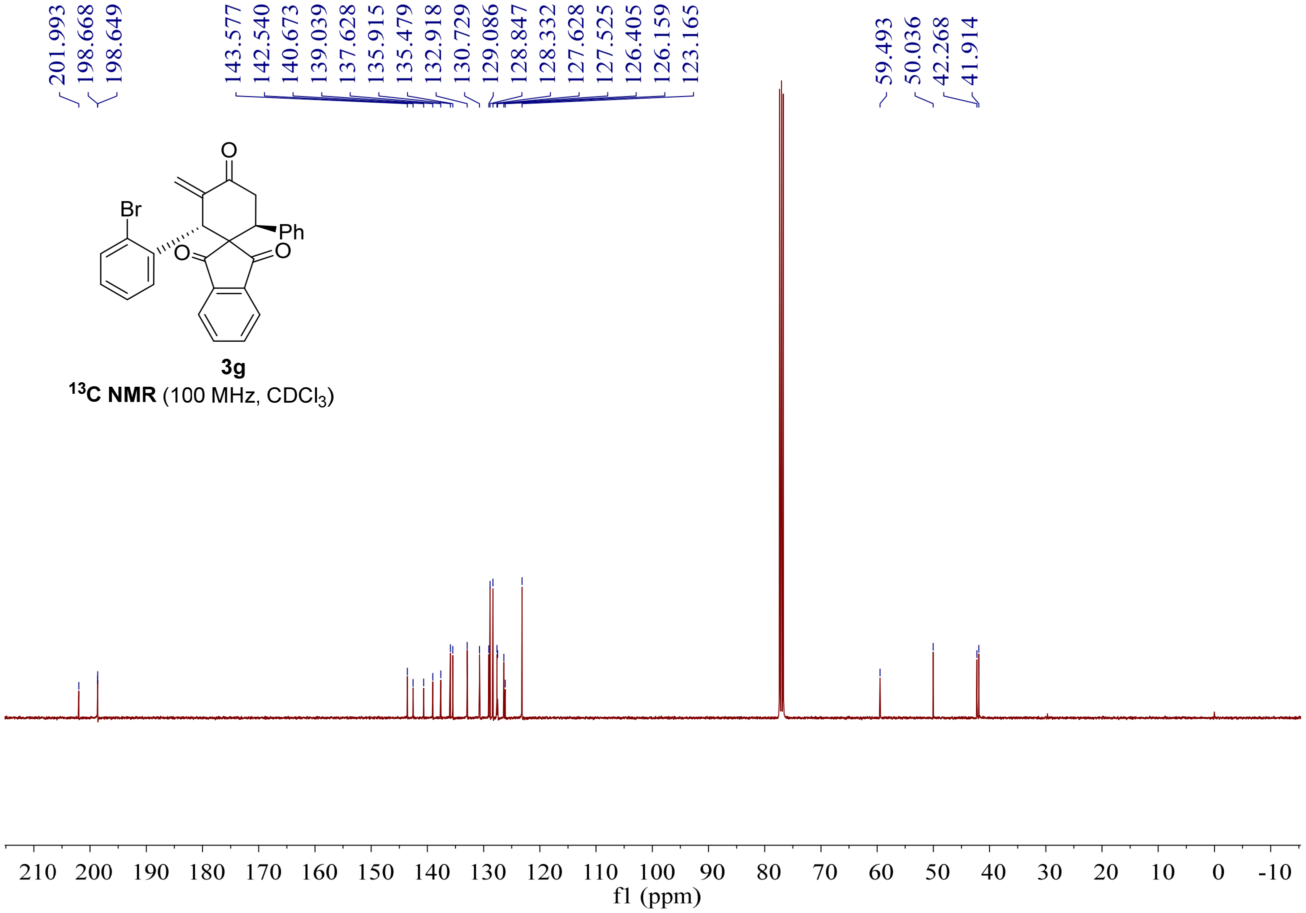
201.993
198.668
198.649
143.577
142.540
140.673
139.039
137.628
135.915
135.479
132.918
130.729
129.086
128.847
128.332
127.628
127.525
126.405
126.159
123.165
59.493
50.036
42.268
41.914
Br
O
Ph
O
O
3g
13C NMR (100 MHz, CDCl3)
f1 (ppm)

Daicel Chiral IC Column, (*i*PrOH/*n*-hexane = 40/60, 1.0 mL/min)

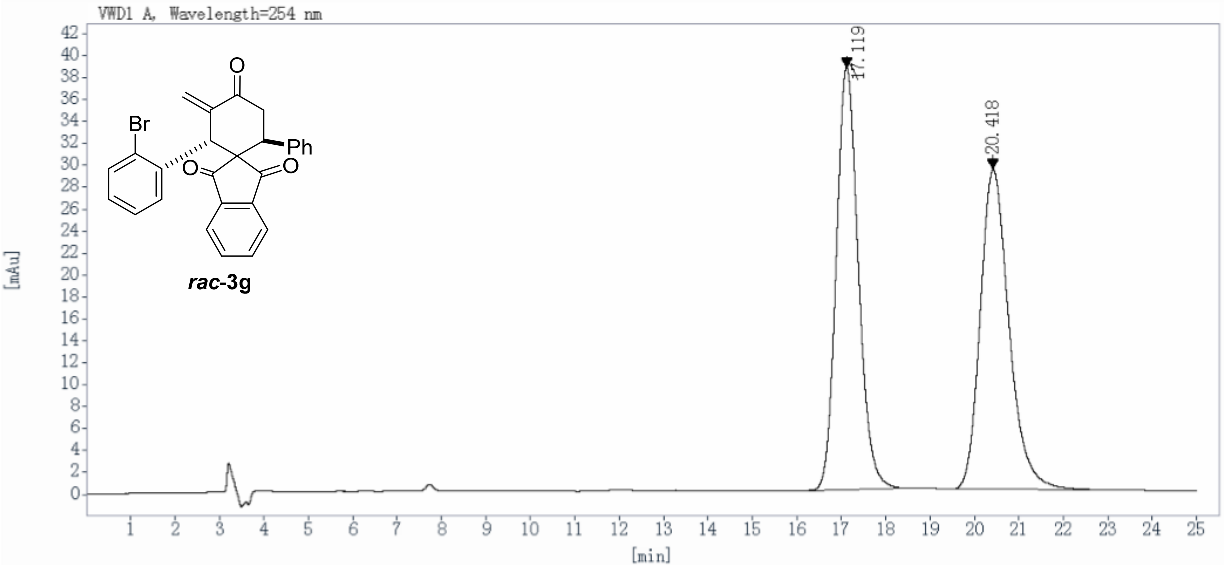

Ret Time [min]	Peak Type	Width [min]	Height [mAU]	Area [mAU*s]	Area [%]
17.119	BB	0.53	38.5068	1329.6179	50.1740
20.418	BB	0.69	29.2005	1320.3956	49.8260
			Totals:	2650.0135	100.0000

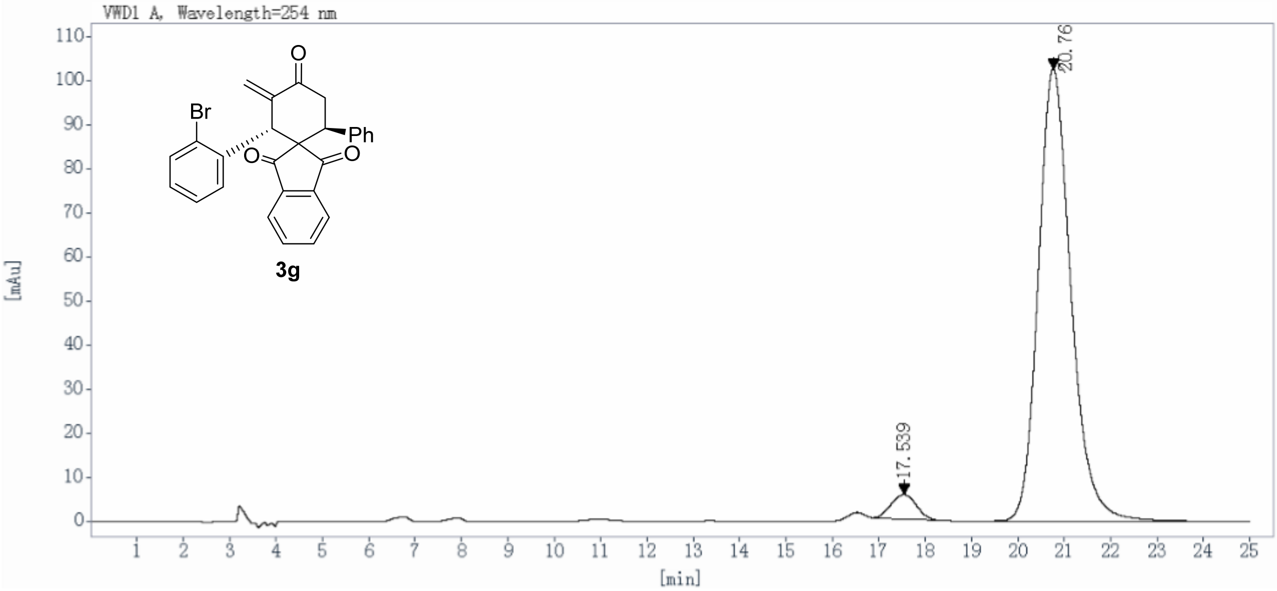

Ret Time [min]	Peak Type	Width [min]	Height [mAU]	Area [mAU*s]	Area [%]
17.539	BB	0.60	5.4962	209.9604	4.0145
20.760	BB	0.75	102.5790	5020.1069	95.9855
			Totals:	5230.0674	100.0000

HRMS (ESI-TOF) m/z: $[M + Na]^+$ Calcd for $C_{27}H_{19}BrO_3Na^+$ 493.0410 (^{79}Br) and 495.0389 (^{81}Br); Found 493.0414 (^{79}Br) and 495.0399 (^{81}Br).

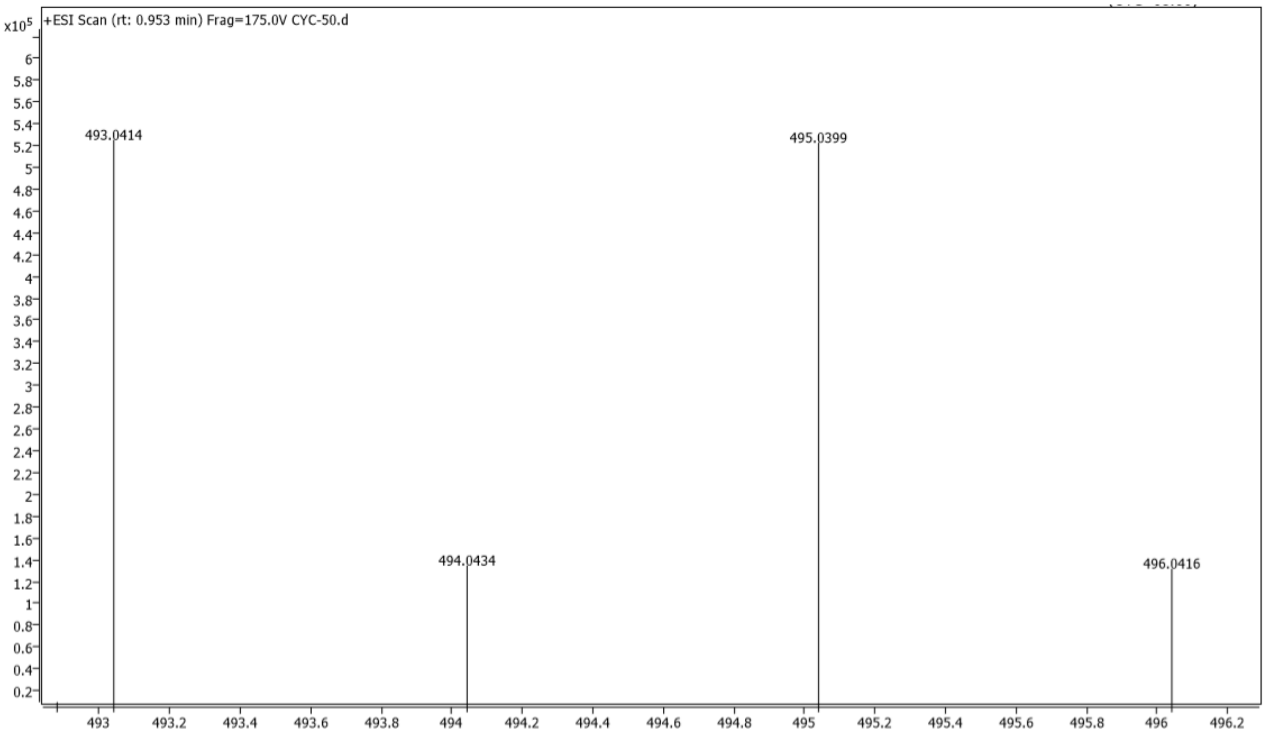

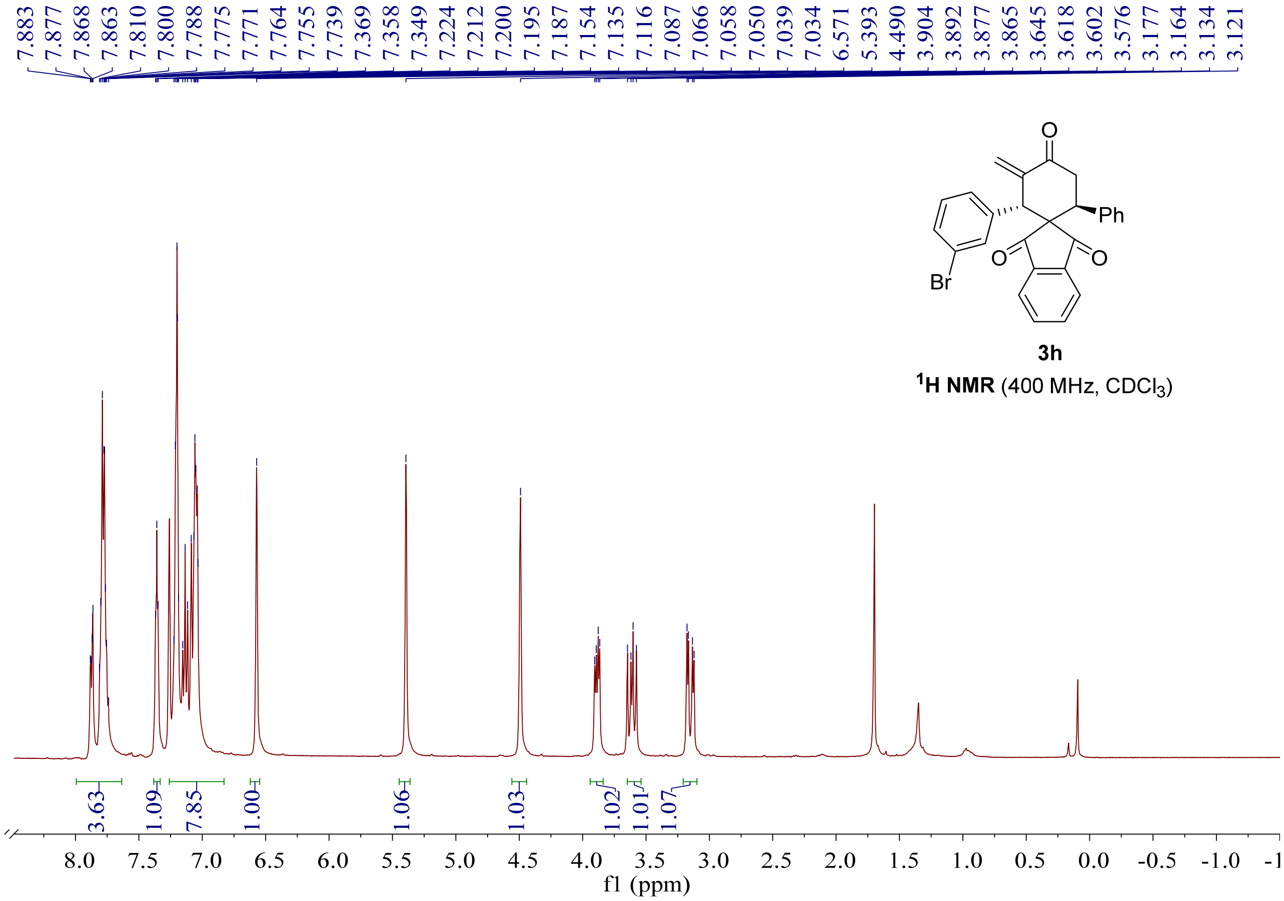
7.883
7.877
7.868
7.863
7.810
7.800
7.788
7.775
7.771
7.764
7.755
7.739
7.369
7.358
7.349
7.224
7.212
7.200
7.195
7.187
7.154
7.135
7.116
7.087
7.066
7.058
7.050
7.039
7.034
6.571
5.393
4.490
3.904
3.892
3.877
3.865
3.645
3.618
3.602
3.576
3.177
3.164
3.134
3.121
O
O
O
Ph
Br
3h
1H NMR (400 MHz, CDCl3)
3.63
1.09
7.85
1.00
1.06
1.03
1.02
1.01
1.07
f1 (ppm)

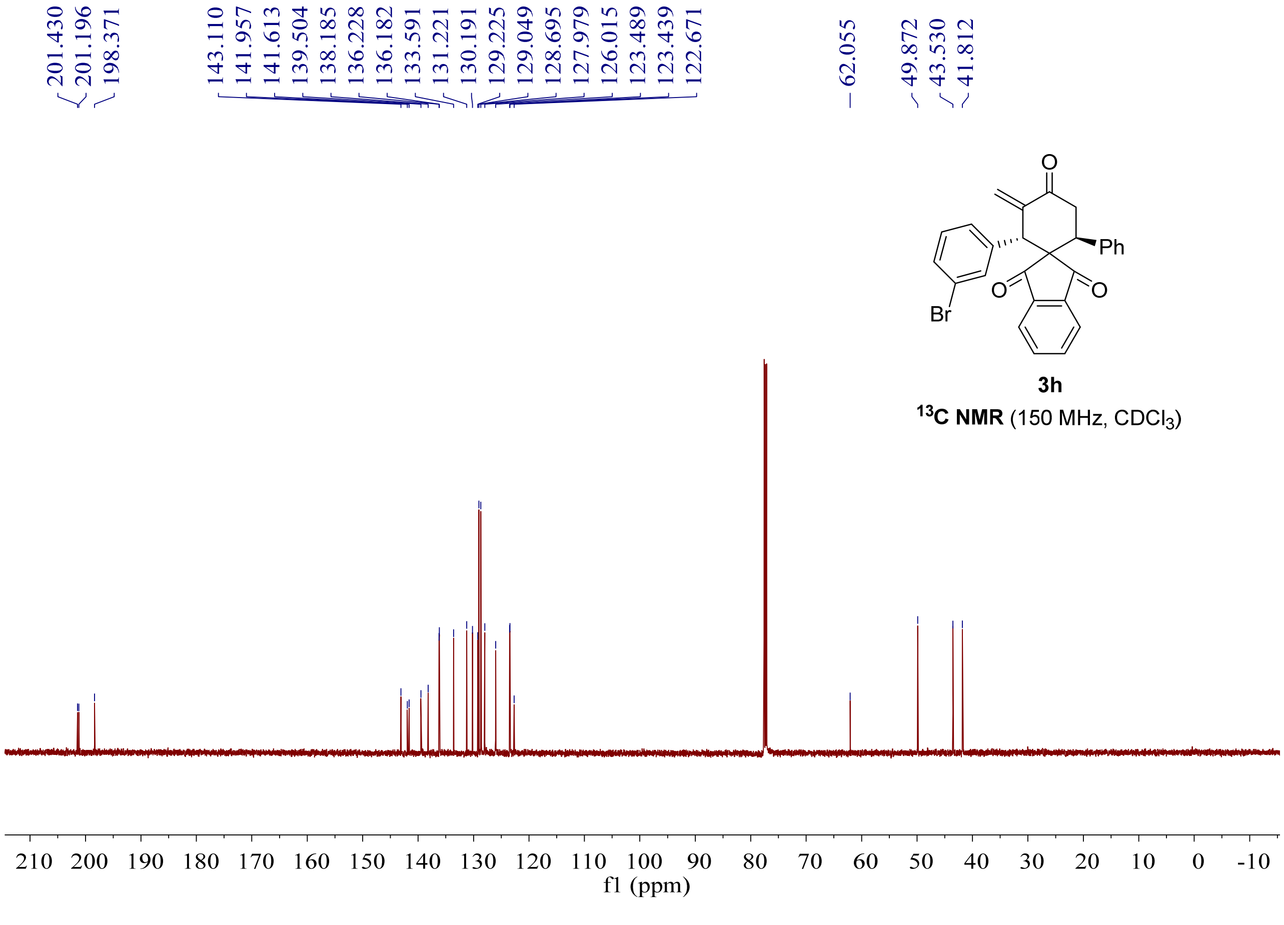
201.430
201.196
198.371
143.110
141.957
141.613
139.504
138.185
136.228
136.182
133.591
131.221
130.191
129.225
129.049
128.695
127.979
126.015
123.489
123.439
122.671
62.055
49.872
43.530
41.812
O
O
O
Ph
Br
3h
13C NMR (150 MHz, CDCl3)
f1 (ppm)

Daicel Chiral IC Column, (*i*PrOH/*n*-hexane = 10/90, 1.0 mL/min)

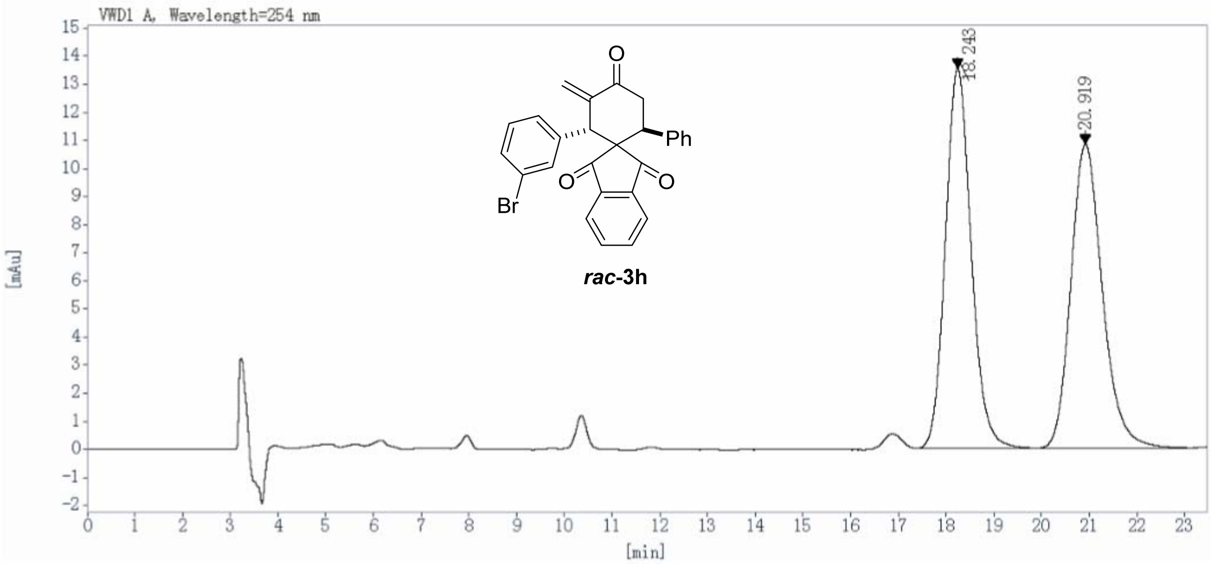

Ret Time [min]	Peak Type	Width [min]	Height [mAU]	Area [mAU*s]	Area [%]
18.243	BB	0.57	13.5275	506.3877	50.4796
20.919	BB	0.70	10.8245	496.7648	49.5204
			Totals:	1003.1524	100.0000

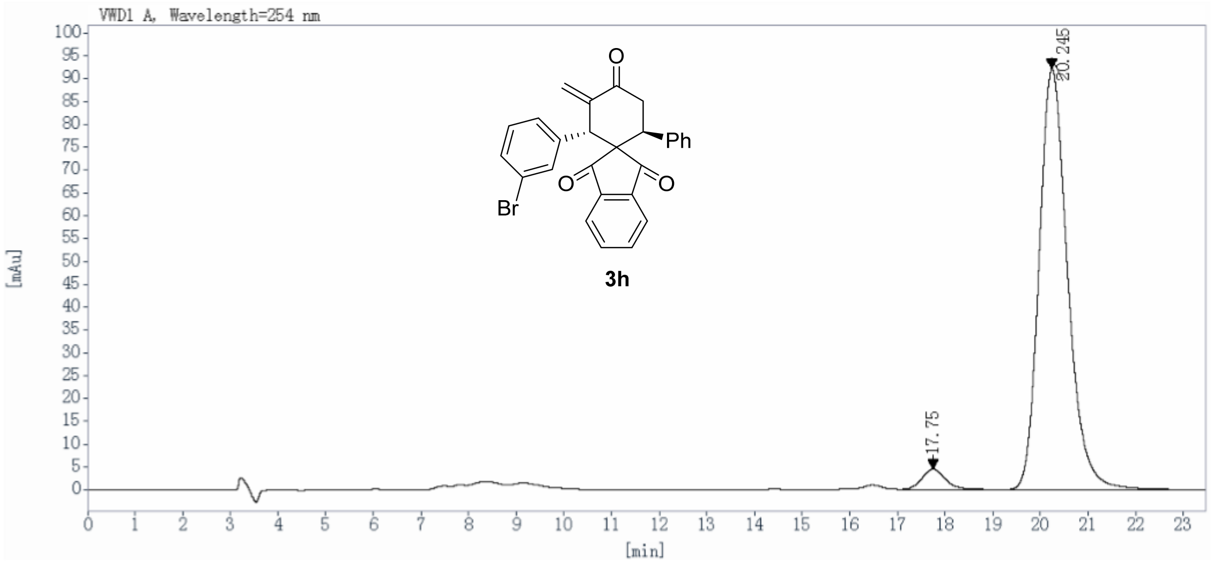

Ret Time [min]	Peak Type	Width [min]	Height [mAU]	Area [mAU*s]	Area [%]
17.750	BB	0.52	4.4152	150.3494	3.7861
20.245	BB	0.63	92.2033	3820.7161	96.2139
			Totals:	3971.0655	100.0000

HRMS (ESI-TOF) m/z: [M + Na]$^+$ Calcd for $C_{27}H_{19}O_3BrNa^+$ 493.0410 (^{79}Br) and 495.0389 (^{81}Br); Found 493.0408 (^{79}Br) and 495.0395 (^{81}Br).

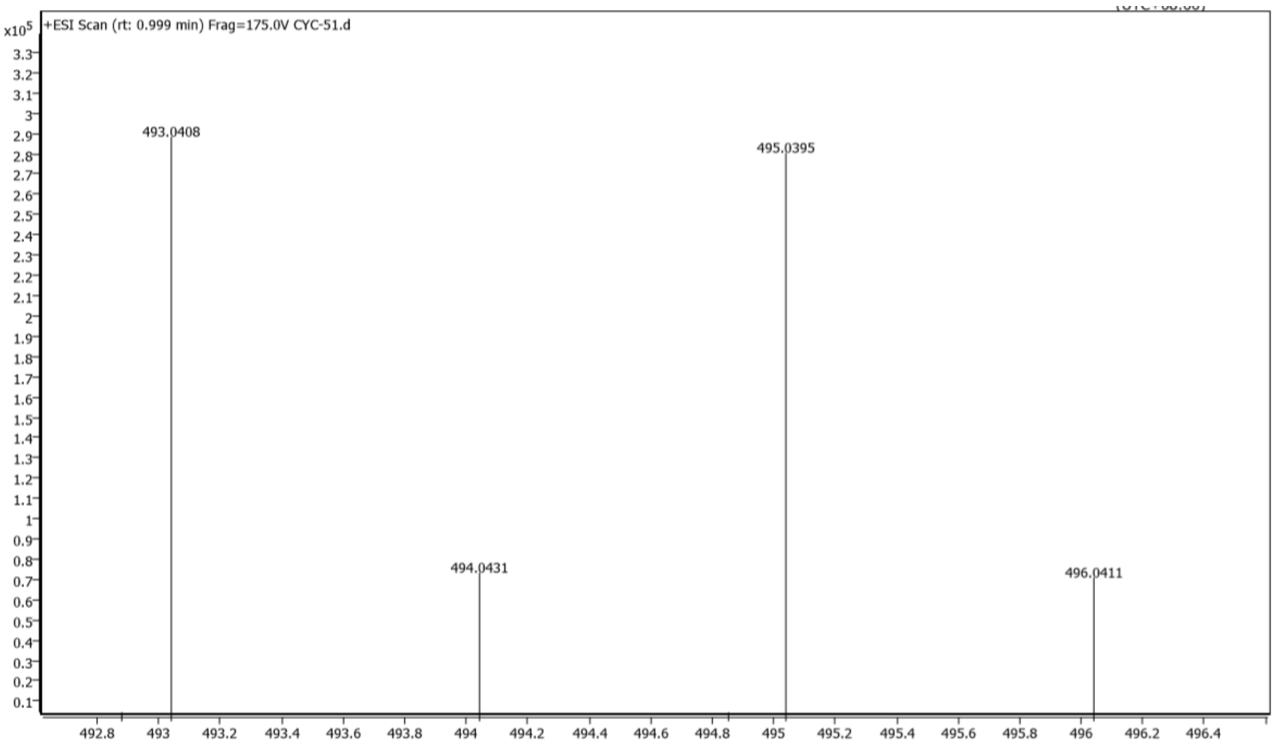

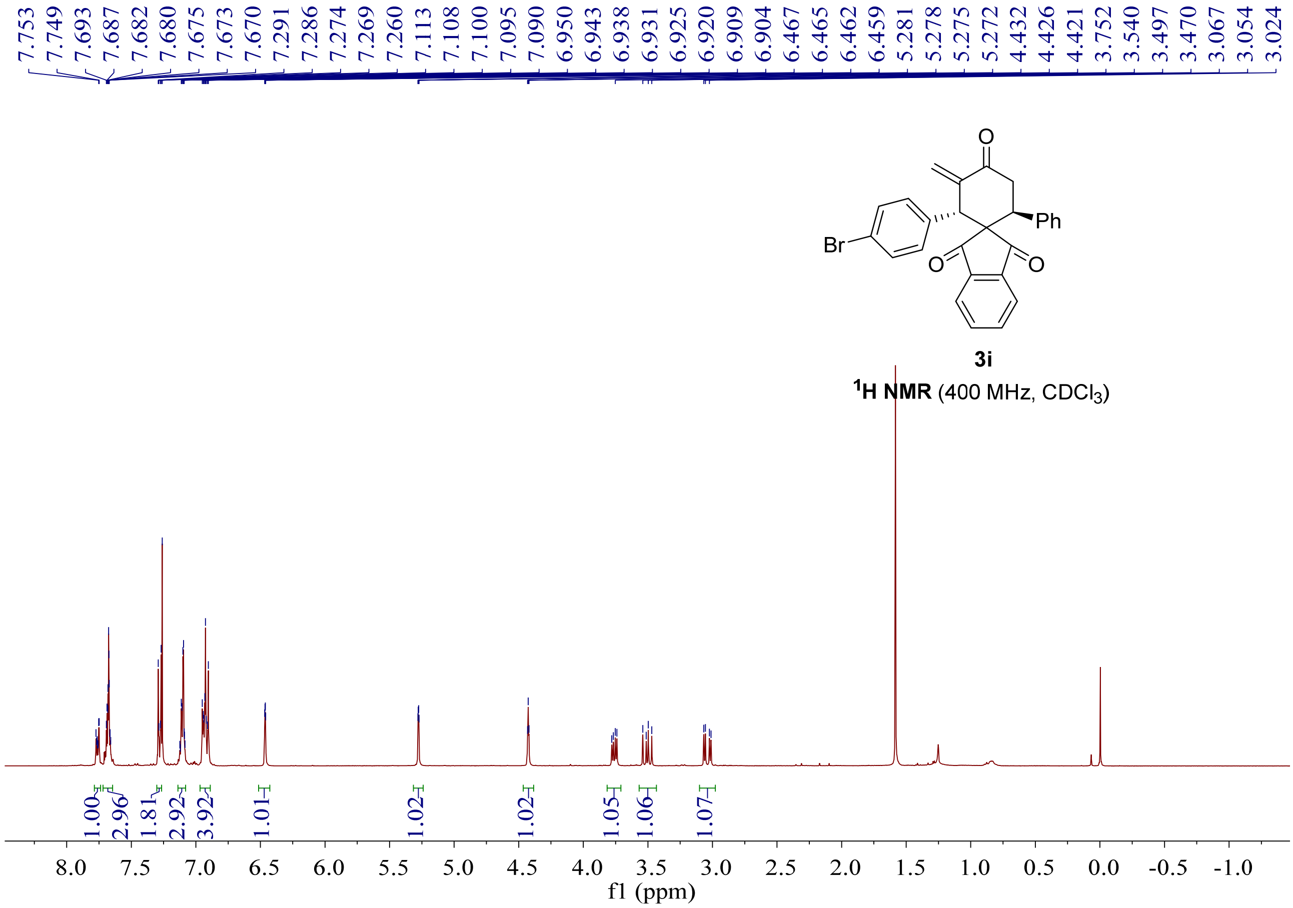
7.753
7.749
7.693
7.687
7.682
7.680
7.675
7.673
7.670
7.291
7.286
7.274
7.269
7.260
7.113
7.108
7.100
7.095
7.090
6.950
6.943
6.938
6.931
6.925
6.920
6.909
6.904
6.467
6.465
6.462
6.459
5.281
5.278
5.275
5.272
4.432
4.426
4.421
3.752
3.540
3.497
3.470
3.067
3.054
3.024
O
Br
Ph
O
O
3i
1H NMR (400 MHz, CDCl3)
1.00
2.96
1.81
2.92
3.92
1.01
1.02
1.02
1.05
1.06
1.07
8.0 7.5 7.0 6.5 6.0 5.5 5.0 4.5 4.0 3.5 3.0 2.5 2.0 1.5 1.0 0.5 0.0 -0.5 -1.0
f1 (ppm)

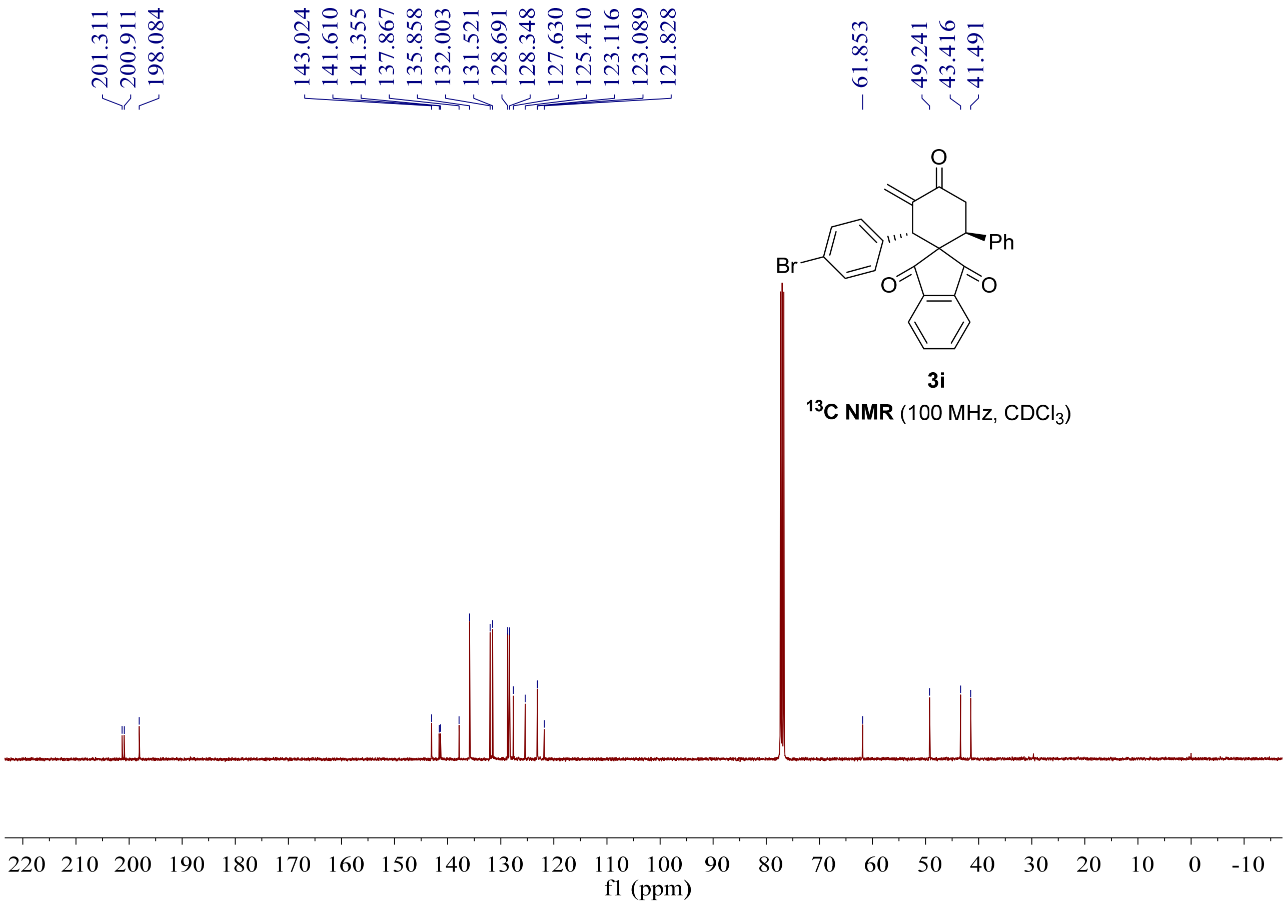
201.311
200.911
198.084
143.024
141.610
141.355
137.867
135.858
132.003
131.521
128.691
128.348
127.630
125.410
123.116
123.089
121.828
61.853
49.241
43.416
41.491
O
Br
Ph
O
O
3i
13C NMR (100 MHz, CDCl3)
220 210 200 190 180 170 160 150 140 130 120 110 100 90 80 70 60 50 40 30 20 10 0 -10
f1 (ppm)

Daicel Chiral IA Column, (*i*PrOH/*n*-hexane = 20/80, 1.0 mL/min)

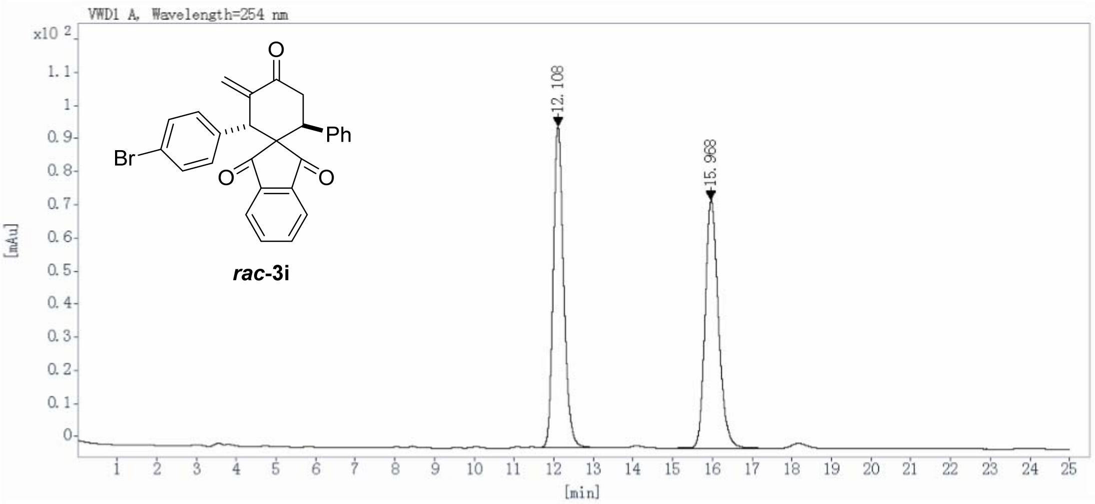

Ret Time [min]	Peak Type	Width [min]	Height [mAU]	Area [mAU*s]	Area [%]
12.108	BB	0.28	97.0560	1775.9832	50.2675
15.968	BB	0.36	75.1599	1757.0834	49.7325
			Totals:	3533.0665	100.0000

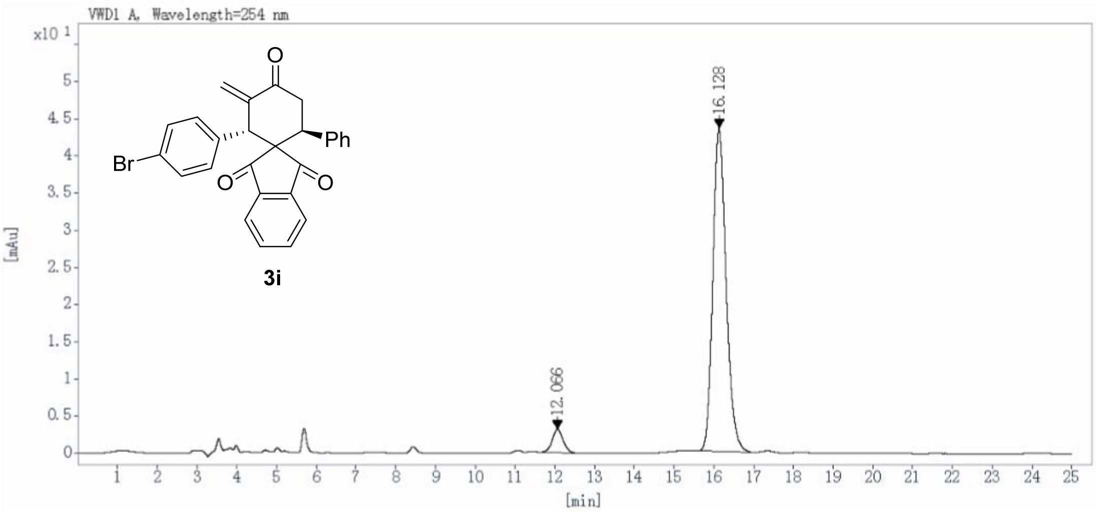

Ret Time [min]	Peak Type	Width [min]	Height [mAU]	Area [mAU*s]	Area [%]
12.066	BB	0.29	3.1588	59.9039	5.6101
16.128	BB	0.36	43.4869	1007.8905	94.3899
			Totals:	1067.7944	100.0000

HRMS (ESI-TOF) m/z: $[M + H]^+$ Calcd for $C_{27}H_{20}BrO_3^+$ 471.0590 (^{79}Br) and 473.0570 (^{81}Br); Found 471.0584 (^{79}Br) and 473.0568 (^{81}Br).

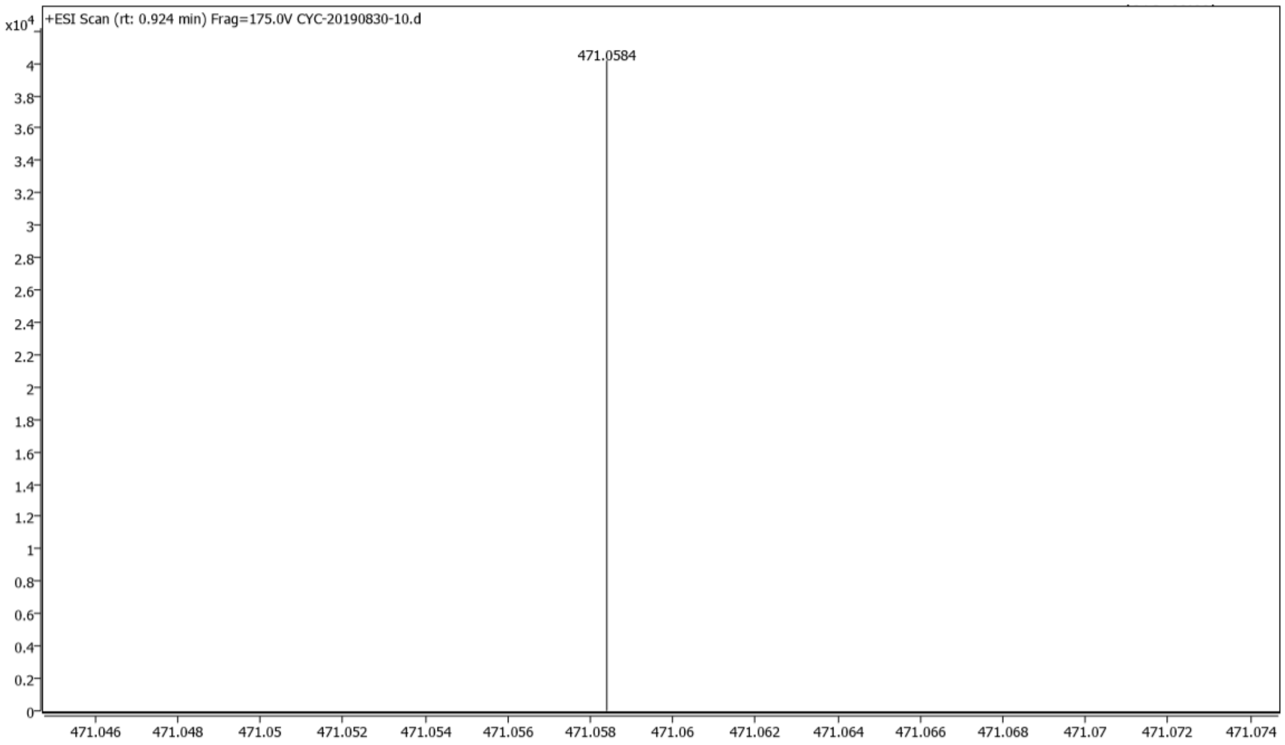

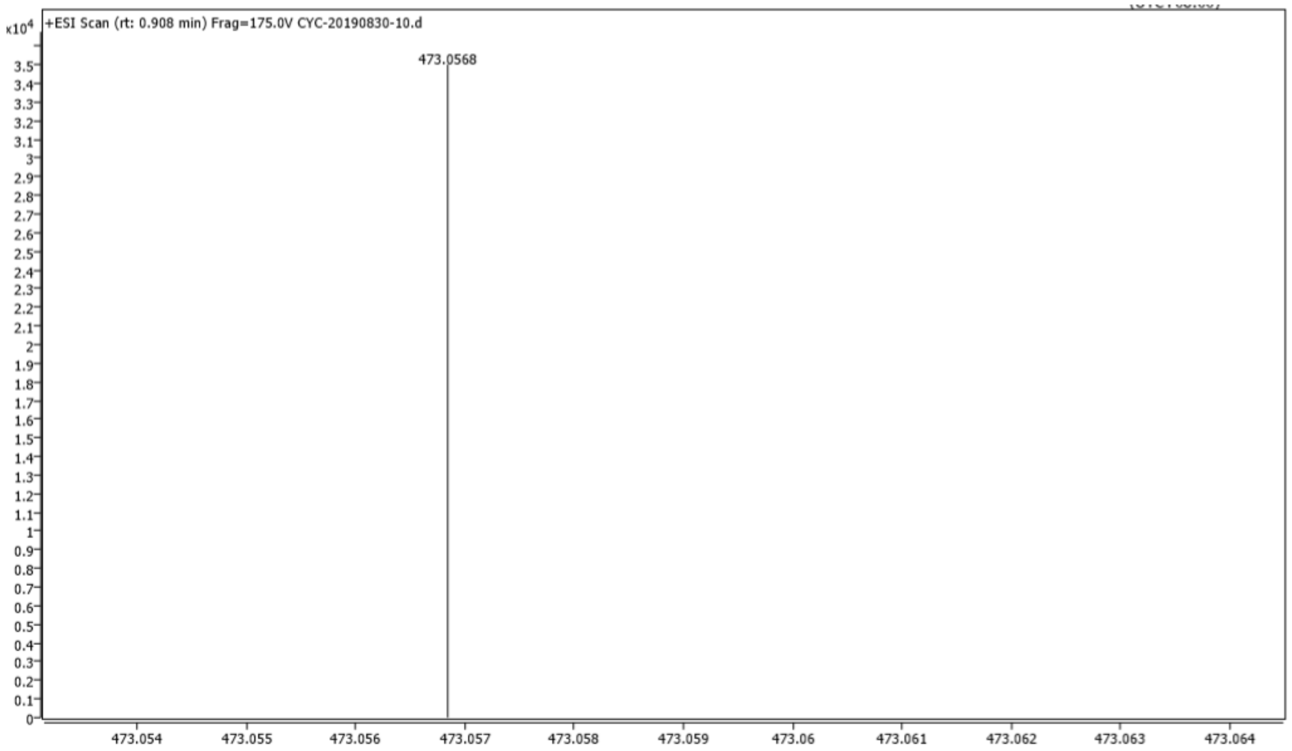

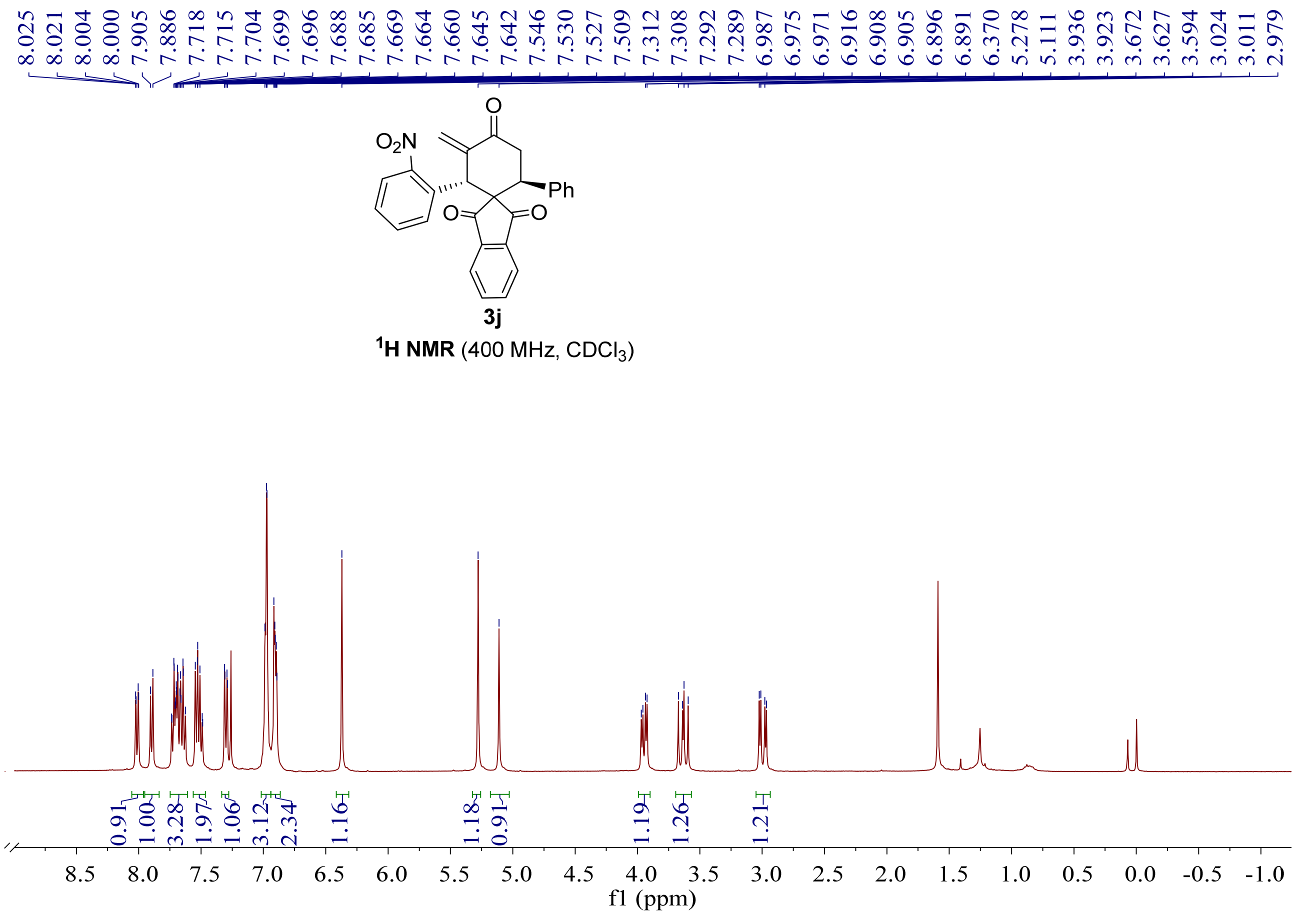
8.025 8.021 8.004 8.000 7.905 7.886 7.718 7.715 7.704 7.699 7.696 7.688 7.685 7.669 7.664 7.660 7.645 7.642 7.546 7.530 7.527 7.509 7.312 7.308 7.292 7.289 6.987 6.975 6.971 6.916 6.908 6.905 6.896 6.891 6.370 5.278 5.111 3.936 3.923 3.672 3.627 3.594 3.024 3.011 2.979
O2N
O
Ph
O
O
3j
1H NMR (400 MHz, CDCl3)
0.91 1.00 3.28 1.97 1.06 3.12 2.34 1.16 1.18 0.91 1.19 1.26 1.21
8.5 8.0 7.5 7.0 6.5 6.0 5.5 5.0 4.5 4.0 3.5 3.0 2.5 2.0 1.5 1.0 0.5 0.0 -0.5 -1.0
f1 (ppm)

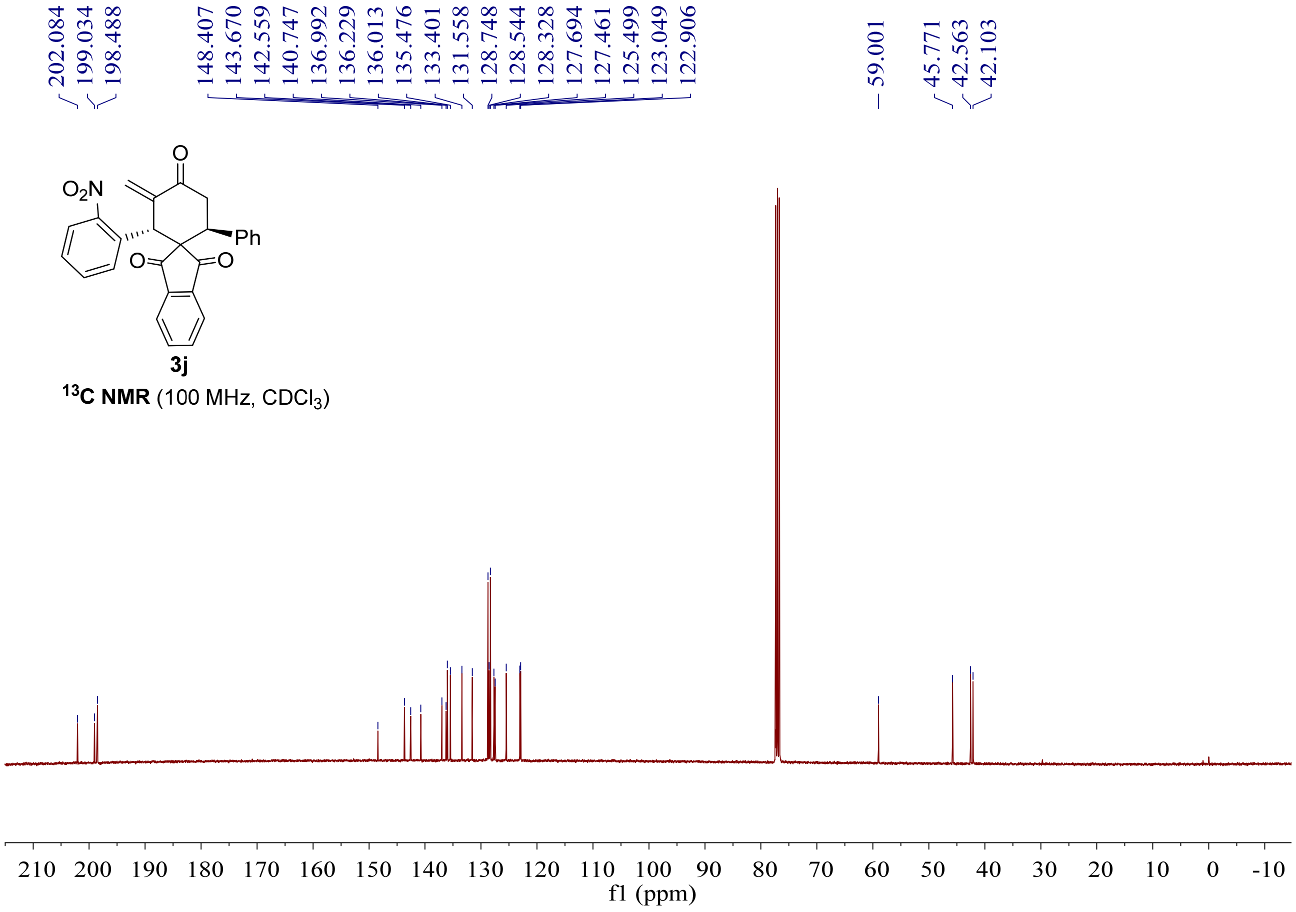
202.084 199.034 198.488
148.407 143.670 142.559 140.747 136.992 136.229 136.013 135.476 133.401 131.558 128.748 128.544 128.328 127.694 127.461 125.499 123.049 122.906
59.001
45.771 42.563 42.103
O2N
O
Ph
O
O
3j
13C NMR (100 MHz, CDCl3)
210 200 190 180 170 160 150 140 130 120 110 100 90 80 70 60 50 40 30 20 10 0 -10
f1 (ppm)

Daicel Chiral IA Column (*i*PrOH/*n*-hexane = 20/80, 1.0 mL/min)

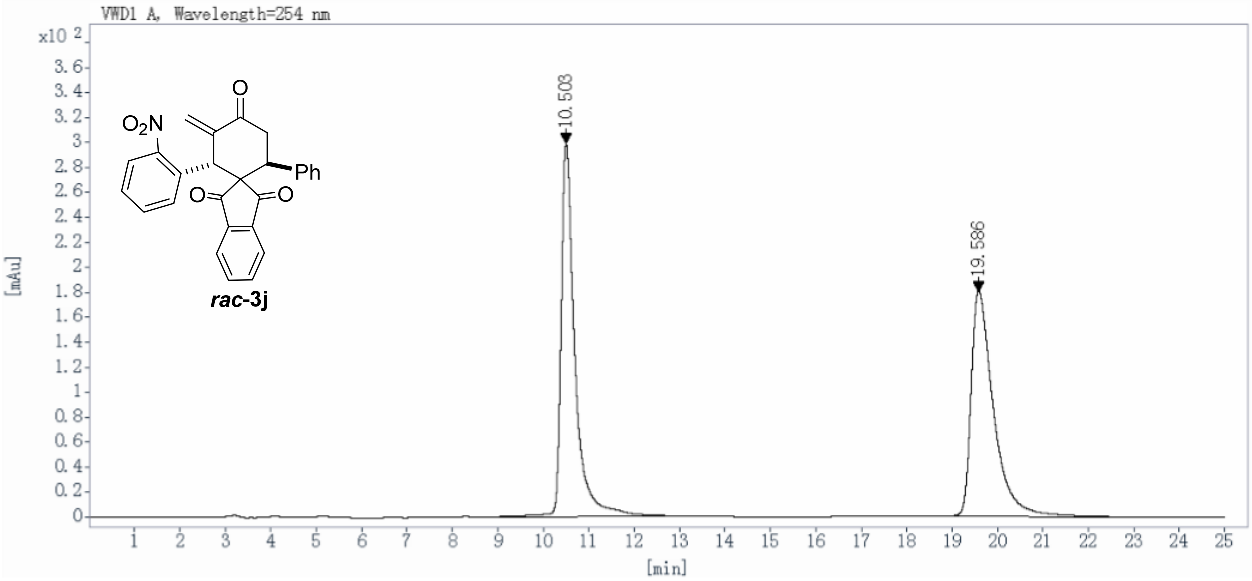

Ret Time [min]	Peak Type	Width [min]	Height [mAU]	Area [mAU*s]	Area [%]
10.503	BB	0.31	298.7657	6314.8315	50.4759
19.586	BB	0.51	180.3753	6195.7510	49.5241
			Totals:	12510.5825	100.0000

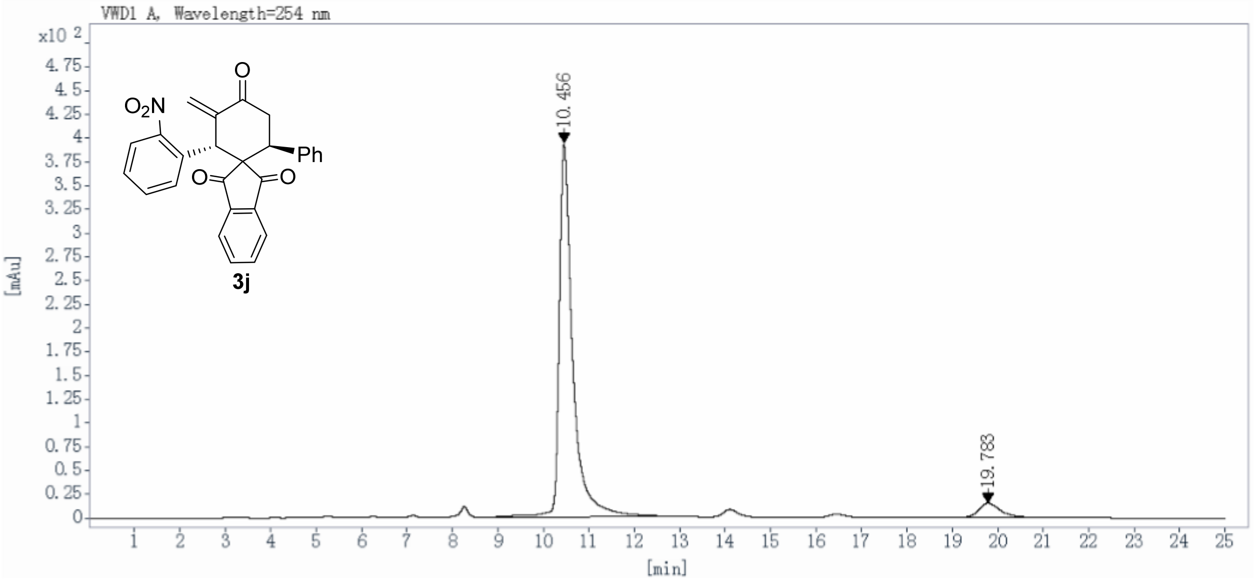

Ret Time [min]	Peak Type	Width [min]	Height [mAU]	Area [mAU*s]	Area [%]
10.456	BBA	0.30	393.3174	8128.3730	94.0611
19.783	BBA	0.50	15.2203	513.2161	5.9389
			Totals:	8641.5892	100.0000

HRMS (ESI-TOF) m/z: $[M + Na]^+$ Calcd for $C_{27}H_{19}NO_5Na^+$ 460.1155; Found 460.1154.

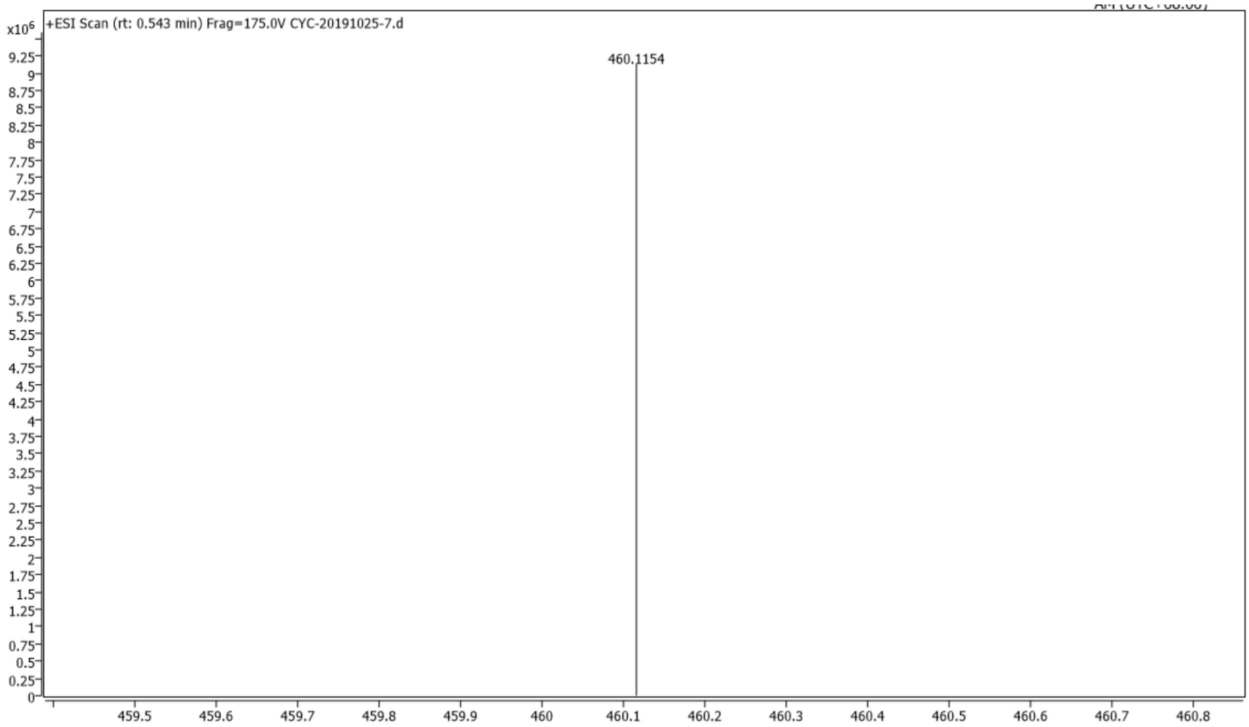

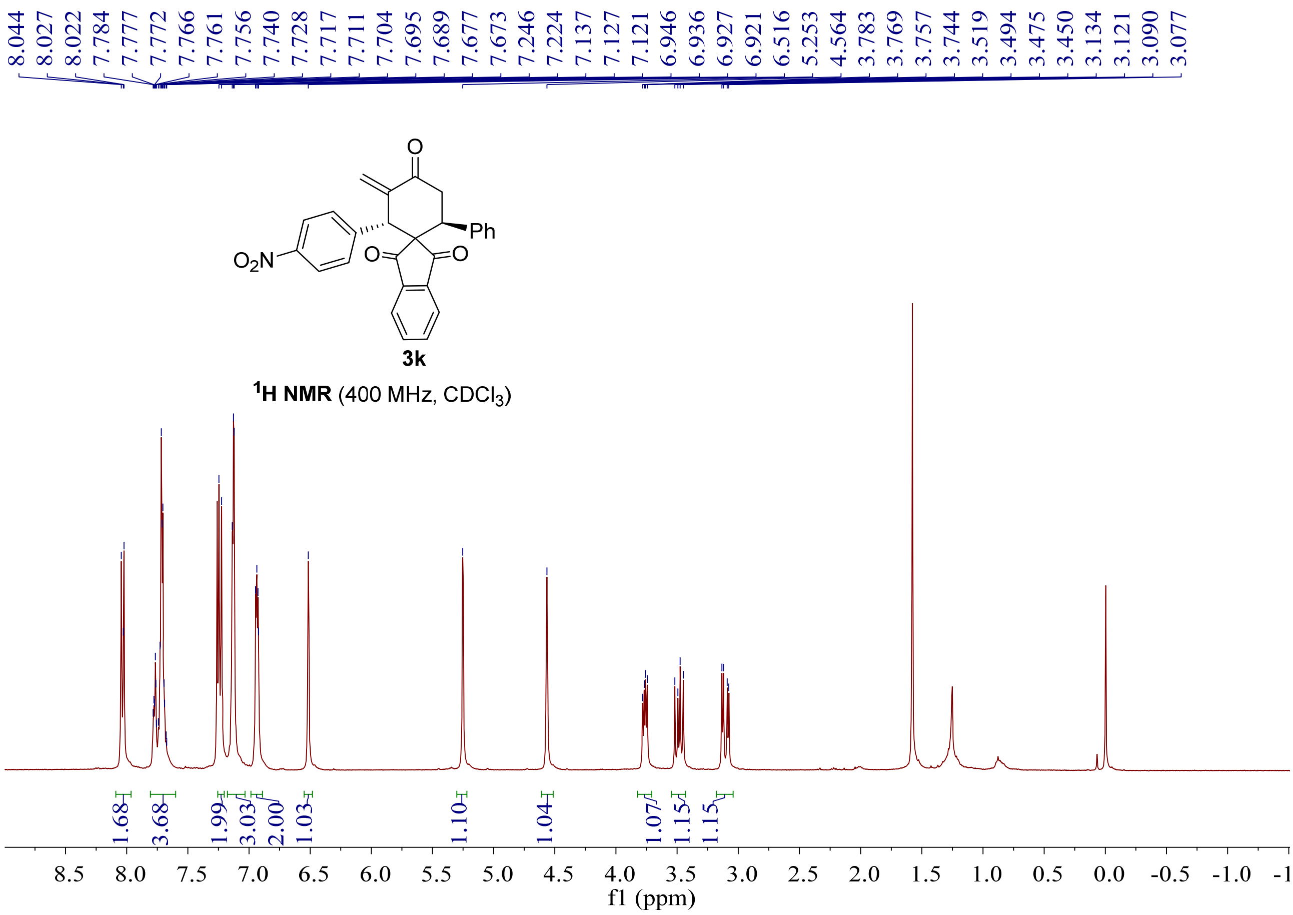

^{1}H NMR (400 MHz, $CDCl_3$)

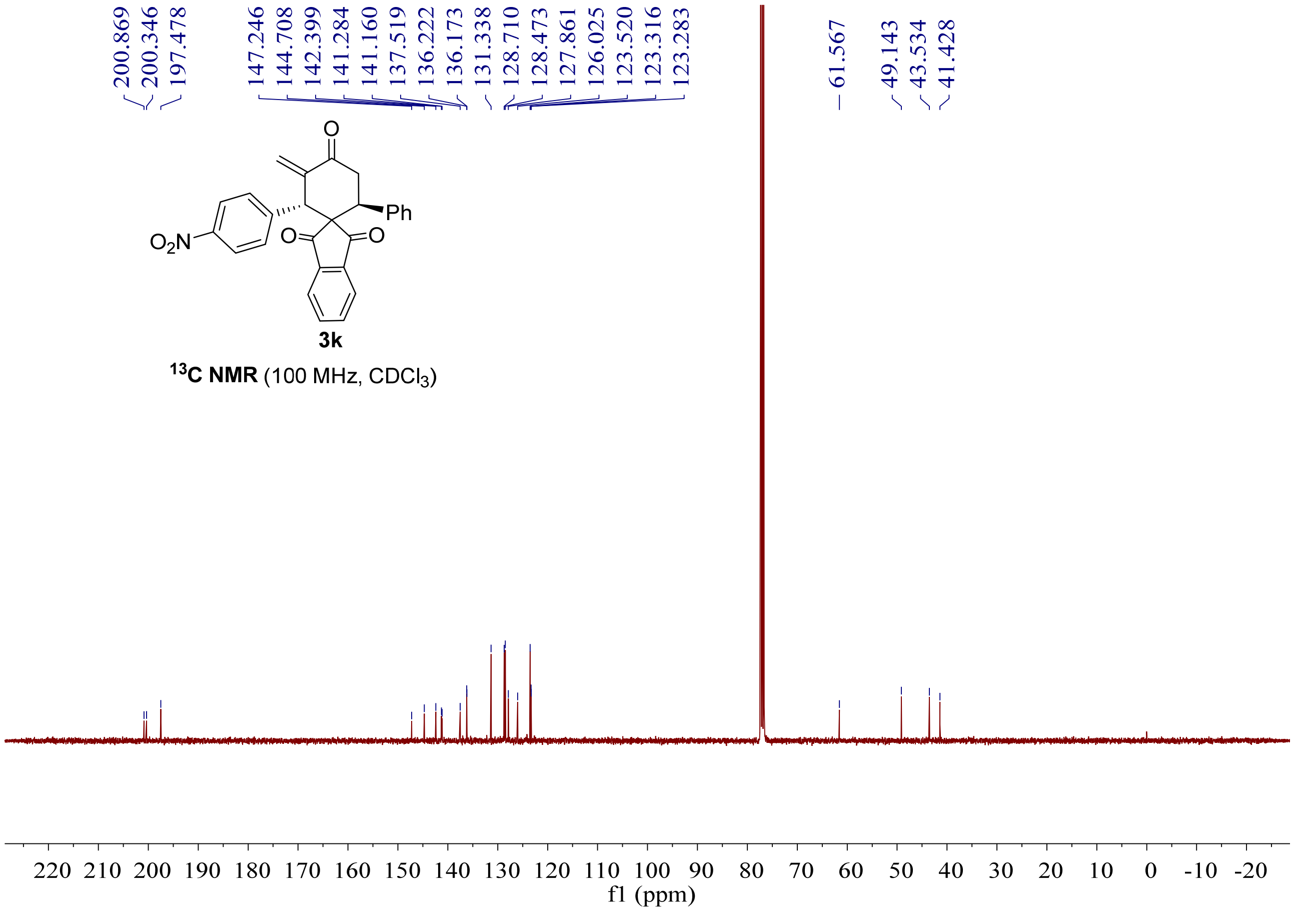

^{13}C NMR (100 MHz, $CDCl_3$)

Daicel Chiral IC Column (iPrOH/n-hexane = 40/60, 1.0 mL/min)

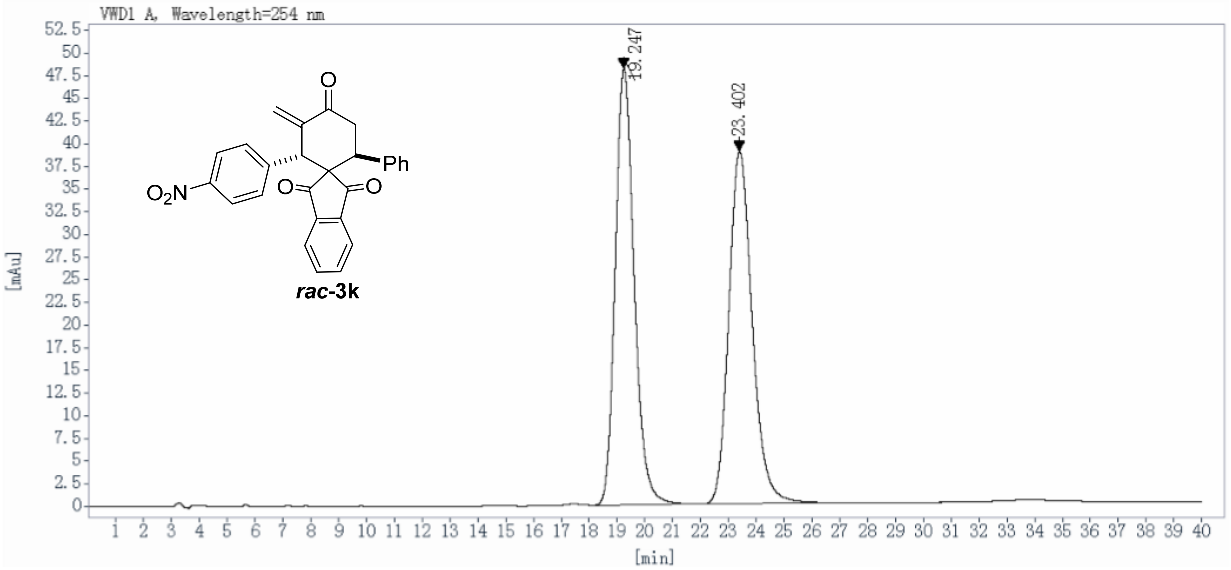

Ret Time [min]	Peak Type	Width [min]	Height [mAU]	Area [mAU*s]	Area [%]
19.247	BB	0.72	48.2004	2272.5864	50.0733
23.402	BB	0.89	38.8323	2265.9341	49.9267
			Totals:	4538.5205	100.0000

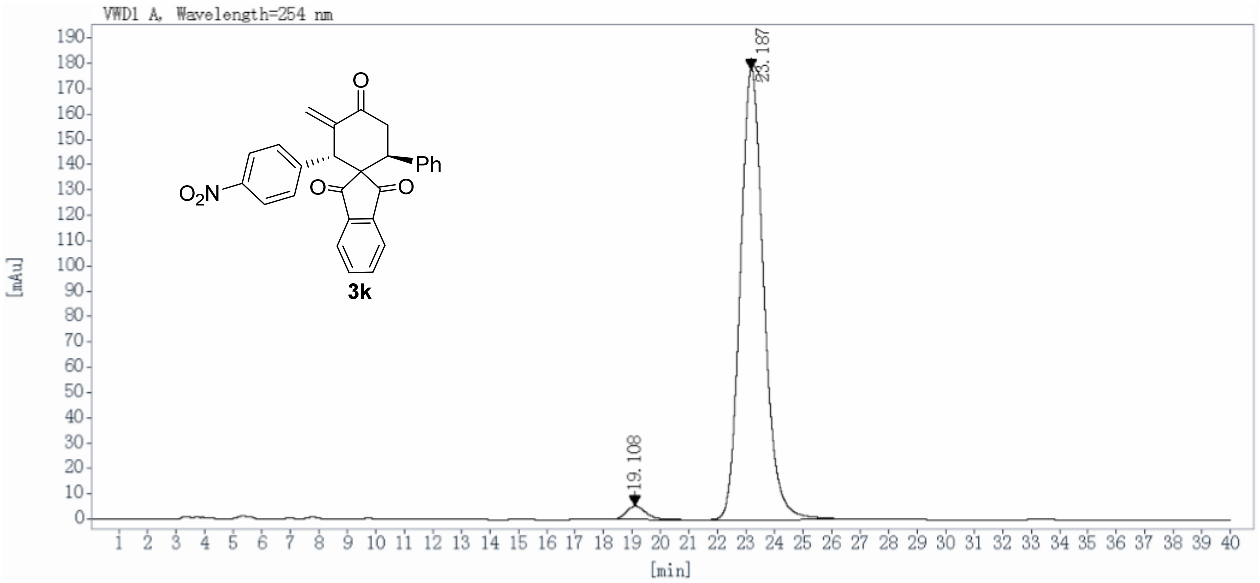

Ret Time [min]	Peak Type	Width [min]	Height [mAU]	Area [mAU*s]	Area [%]
19.108	BB	0.70	5.3157	249.3024	2.3881
23.187	BB	0.88	177.9464	10190.2285	97.6119
			Totals:	10439.5309	100.0000

HRMS (ESI-TOF) m/z: [M + Na]$^+$ Calcd for $C_{27}H_{19}NO_5Na^+$ 460.1155 ; Found 460.1152.

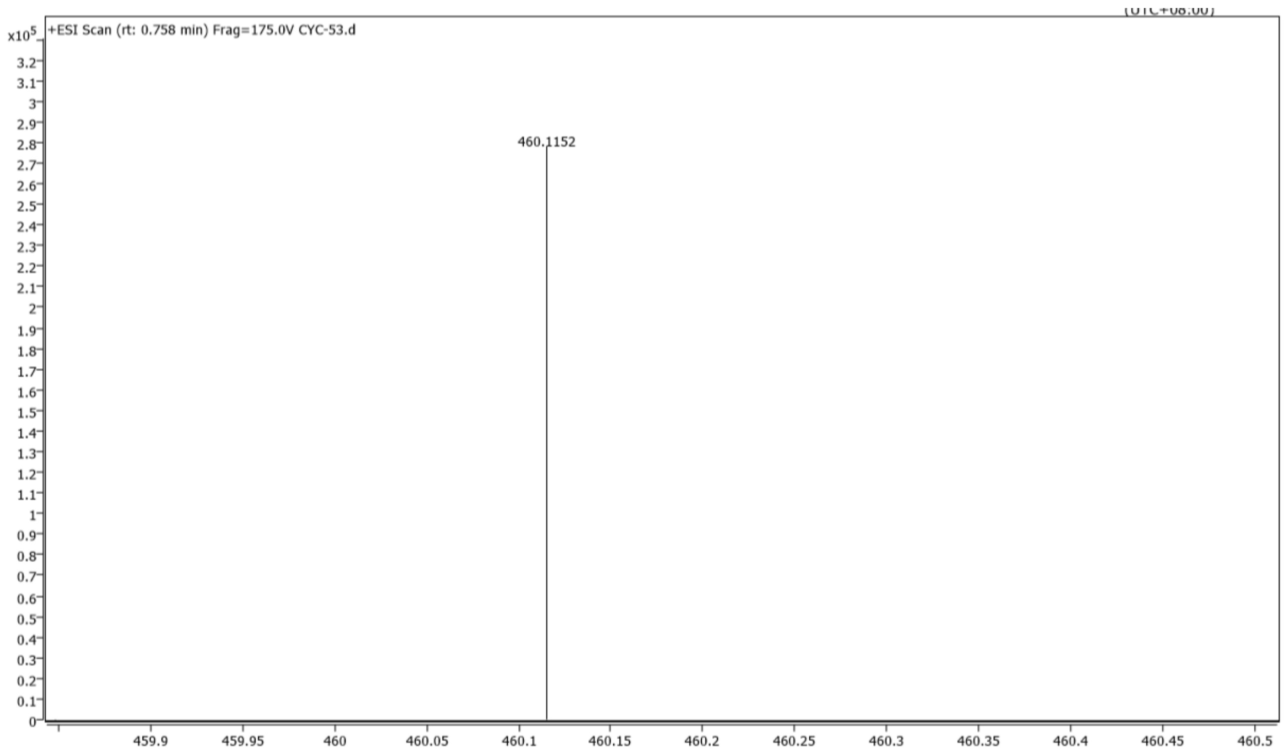

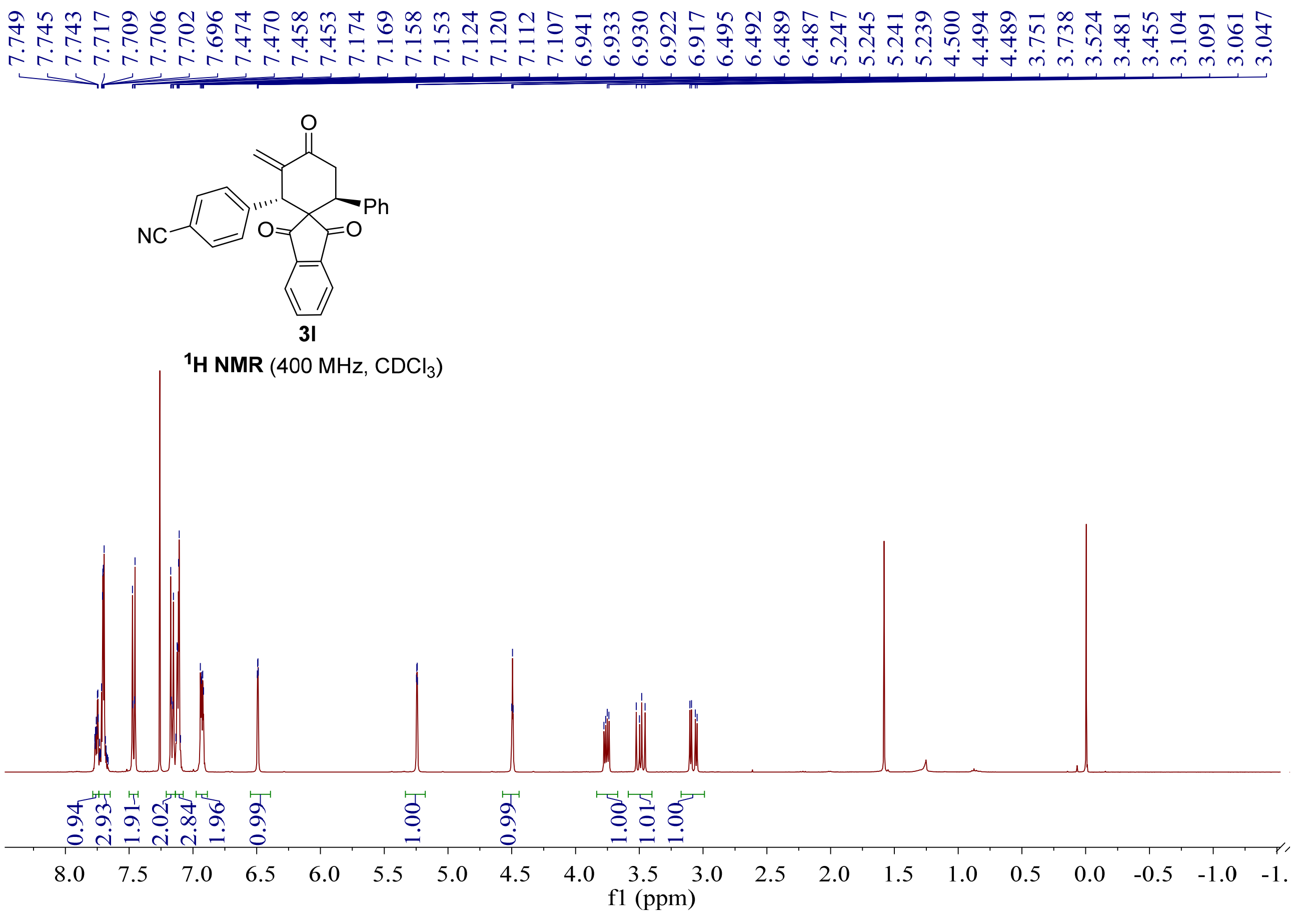
7.749 7.745 7.743 7.717 7.709 7.706 7.702 7.696 7.474 7.470 7.458 7.453 7.174 7.169 7.158 7.153 7.124 7.120 7.112 7.107 6.941 6.933 6.930 6.922 6.917 6.495 6.492 6.489 6.487 5.247 5.245 5.241 5.239 4.500 4.494 4.489 3.751 3.738 3.524 3.481 3.455 3.104 3.091 3.061 3.047
O
Ph
NC
O
O
3l
1H NMR (400 MHz, CDCl3)
0.94 2.93 1.91 2.02 2.84 1.96 0.99 1.00 0.99 1.00 1.01 1.00
f1 (ppm)

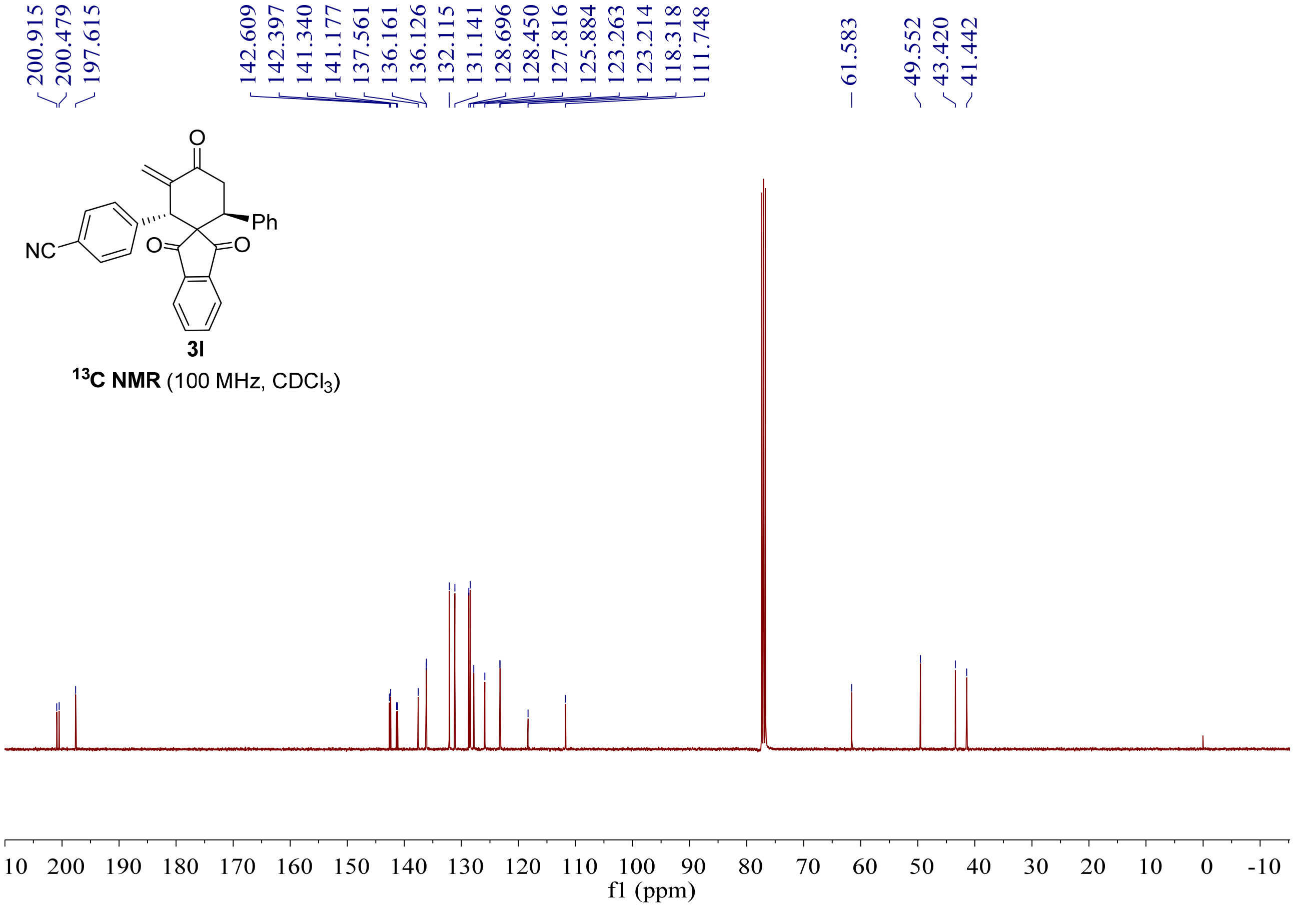
200.915 200.479 197.615 142.609 142.397 141.340 141.177 137.561 136.161 136.126 132.115 131.141 128.696 128.450 127.816 125.884 123.263 123.214 118.318 111.748 61.583 49.552 43.420 41.442
O
Ph
NC
O
O
3l
13C NMR (100 MHz, CDCl3)
f1 (ppm)

Daicel Chiral AD-H Column (*n*-hexane/*i*PrOH = 80/20, 1.0 mL/min)

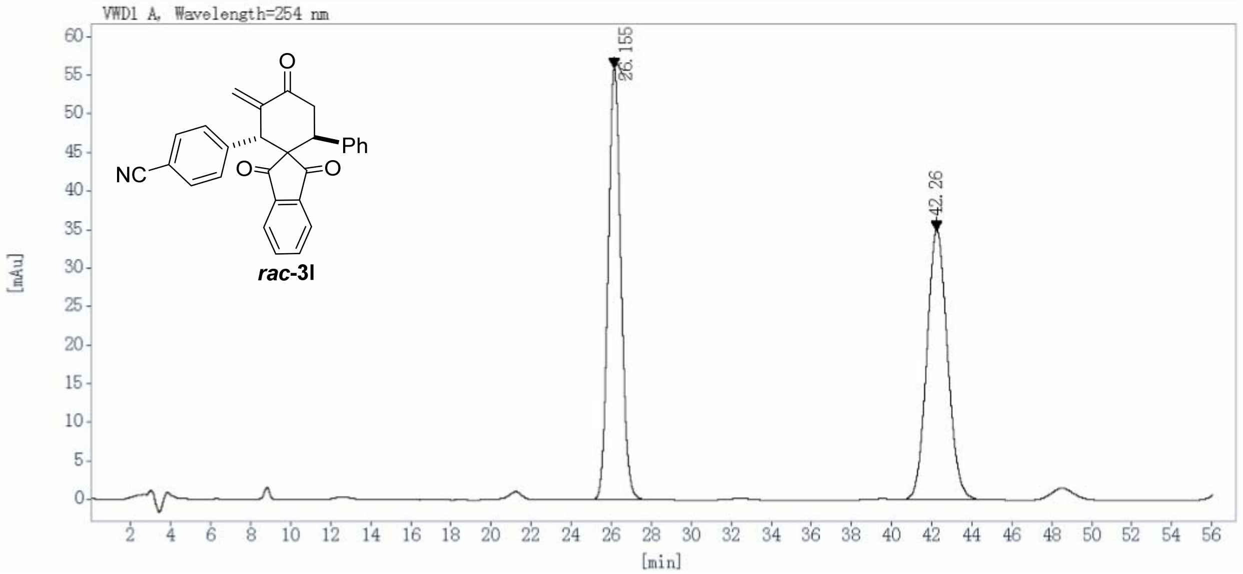

Ret Time [min]	Peak Type	Width [min]	Height [mAU]	Area [mAU*s]	Area [%]
26.155	BB	0.69	55.9674	2475.9138	49.7806
42.260	BB	1.10	34.8499	2497.7432	50.2194
			Totals:	4973.6570	100.0000

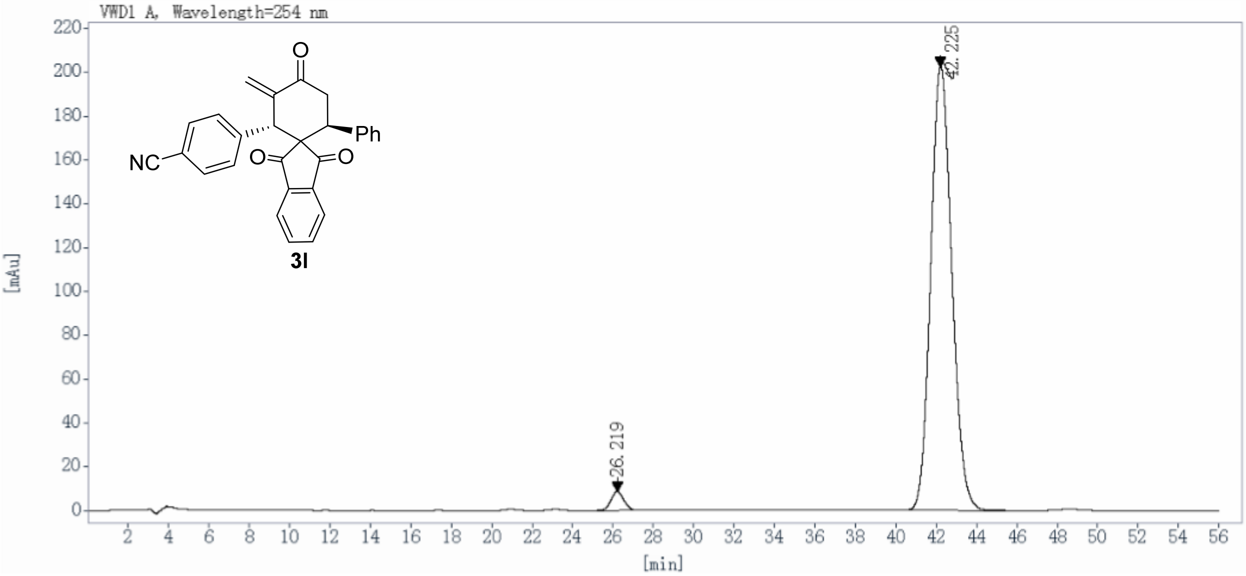

Ret Time [min]	Peak Type	Width [min]	Height [mAU]	Area [mAU*s]	Area [%]
26.219	BB	0.68	8.5985	380.1435	2.5230
42.225	BB	1.13	202.4628	14686.9131	97.4770
			Totals:	15067.0566	100.0000

HRMS (ESI-TOF) m/z: $[M + Na]^+$ Calcd for $C_{28}H_{19}NO_3Na^+$ 440.1257 ; Found 440.1257.

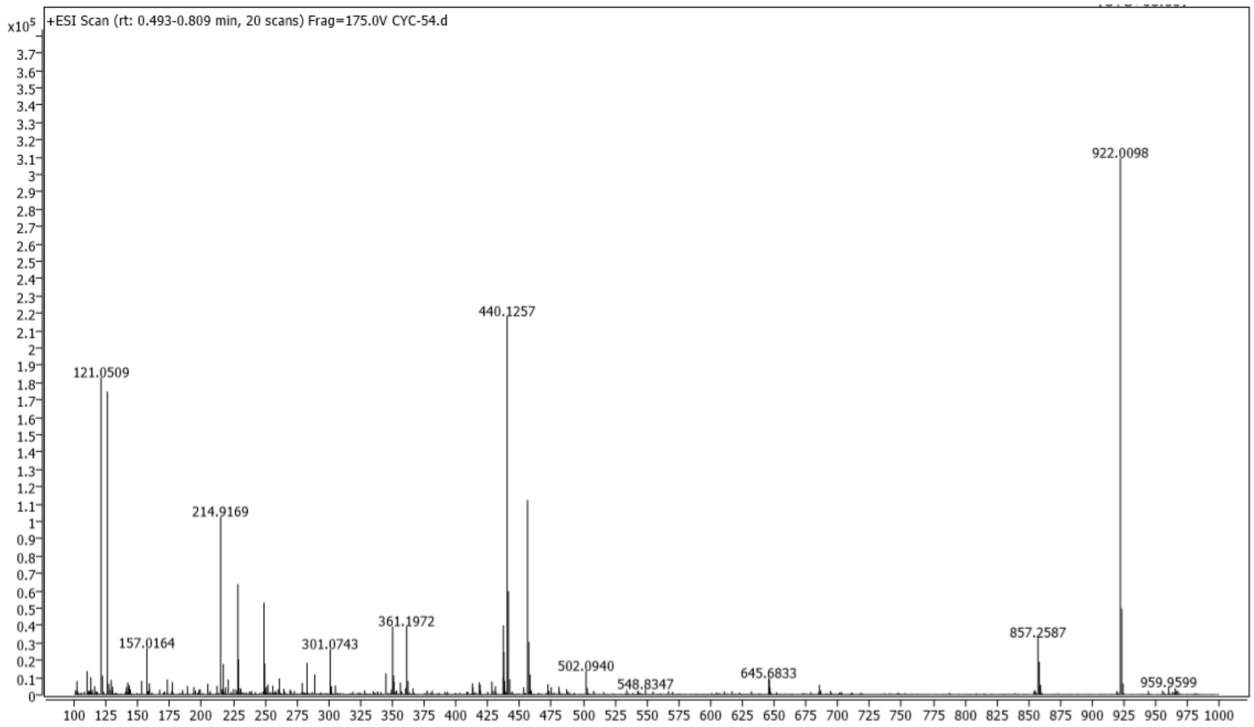

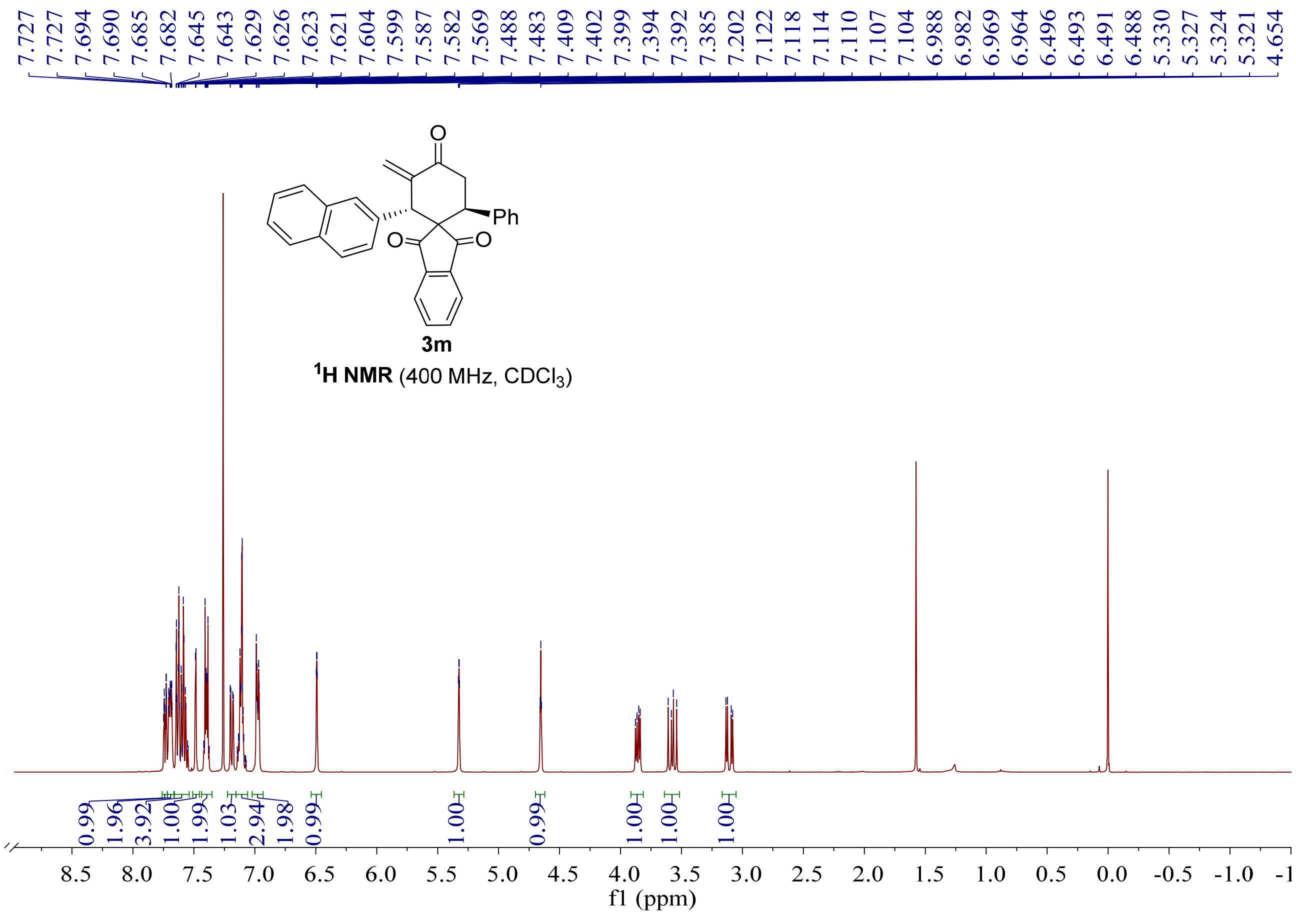
7.727 7.727 7.694 7.690 7.685 7.682 7.645 7.643 7.629 7.626 7.623 7.621 7.604 7.599 7.587 7.582 7.569 7.488 7.483 7.409 7.402 7.399 7.394 7.392 7.385 7.202 7.122 7.118 7.114 7.110 7.107 7.104 6.988 6.982 6.969 6.964 6.496 6.493 6.491 6.488 5.330 5.327 5.324 5.321 4.654
O
Ph
3m
¹H NMR (400 MHz, CDCl3)
0.99 1.96 3.92 1.00 1.99 1.03 2.94 1.98 0.99 1.00 0.99 1.00 1.00 1.00
8.5 8.0 7.5 7.0 6.5 6.0 5.5 5.0 4.5 4.0 3.5 3.0 2.5 2.0 1.5 1.0 0.5 0.0 -0.5 -1.0 -1
f1 (ppm)

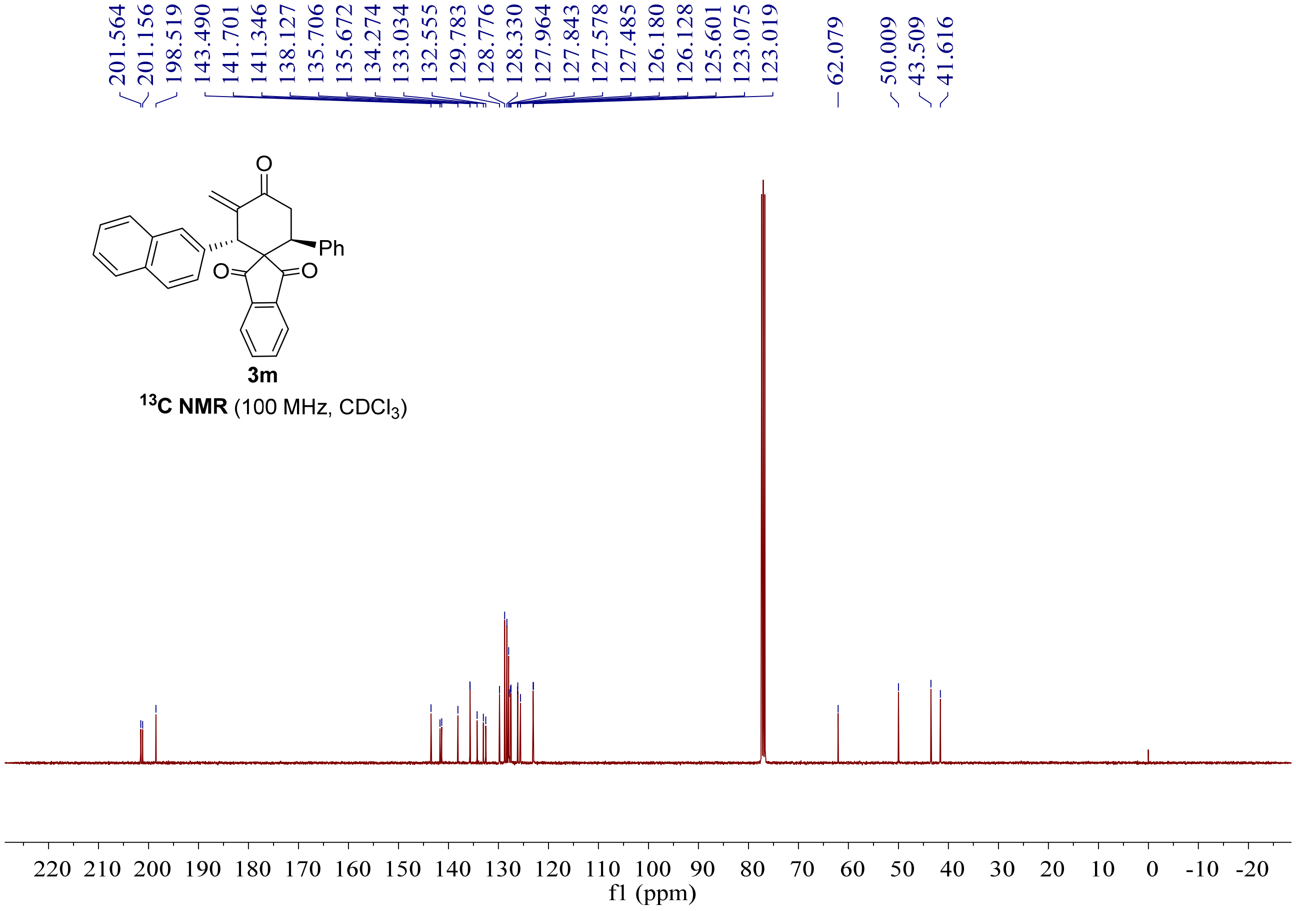
201.564 201.156 198.519 143.490 141.701 141.346 138.127 135.706 135.672 134.274 133.034 132.555 129.783 128.776 128.330 127.964 127.843 127.578 127.485 126.180 126.128 125.601 123.075 123.019 62.079 50.009 43.509 41.616
O
Ph
3m
¹³C NMR (100 MHz, CDCl3)
220 210 200 190 180 170 160 150 140 130 120 110 100 90 80 70 60 50 40 30 20 10 0 -10 -20
f1 (ppm)

Daicel Chiral IE Column (*i*PrOH/*n*-hexane = 40/60, 1.0 mL/min)

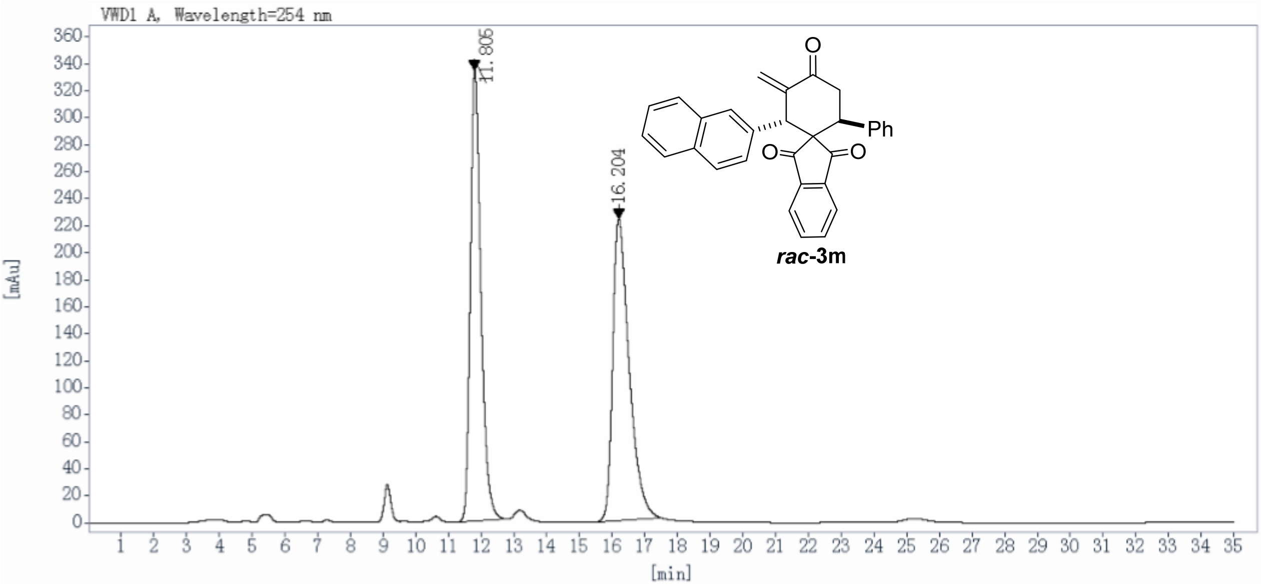

Ret Time [min]	Peak Type	Width [min]	Height [mAU]	Area [mAU*s]	Area [%]
11.805	BB	0.34	334.3717	7525.6519	49.0955
16.204	BBA	0.52	223.7099	7802.9463	50.9045
			Totals:	15328.5981	100.0000

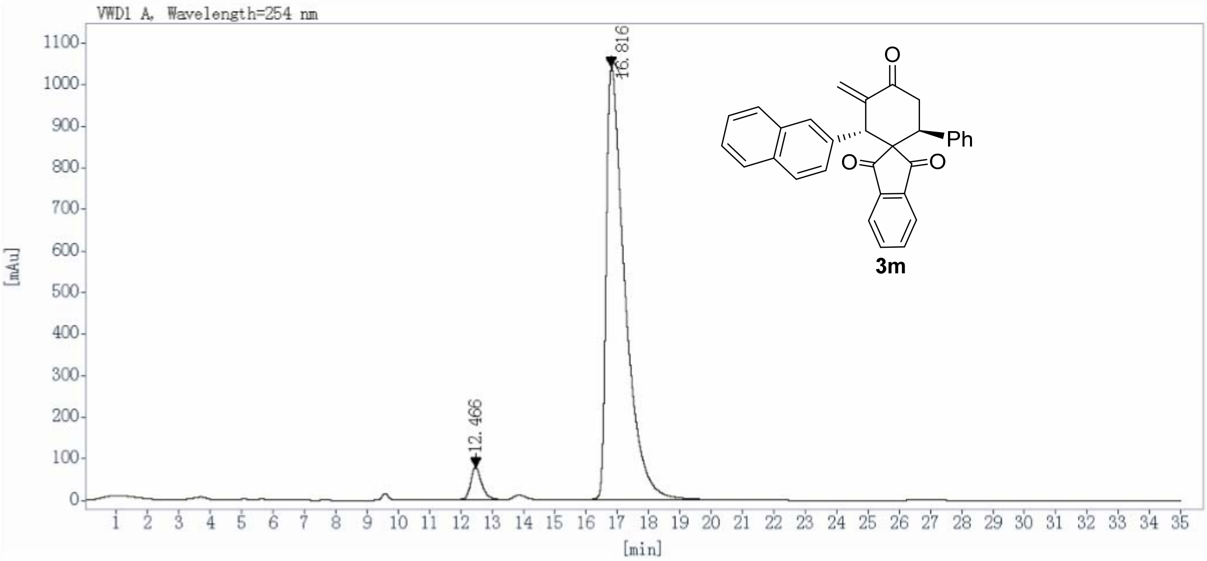

Ret Time [min]	Peak Type	Width [min]	Height [mAU]	Area [mAU*s]	Area [%]
12.466	BB	0.37	77.5145	1897.5538	4.1965
16.816	BBA	0.61	1042.6466	43320.0898	95.8035
			Totals:	45217.6437	100.0000

HRMS (ESI-TOF) m/z: $[M + Na]^+$ Calcd for $C_{31}H_{22}O_3Na^+$ 465.1461 ; Found 465.1465.

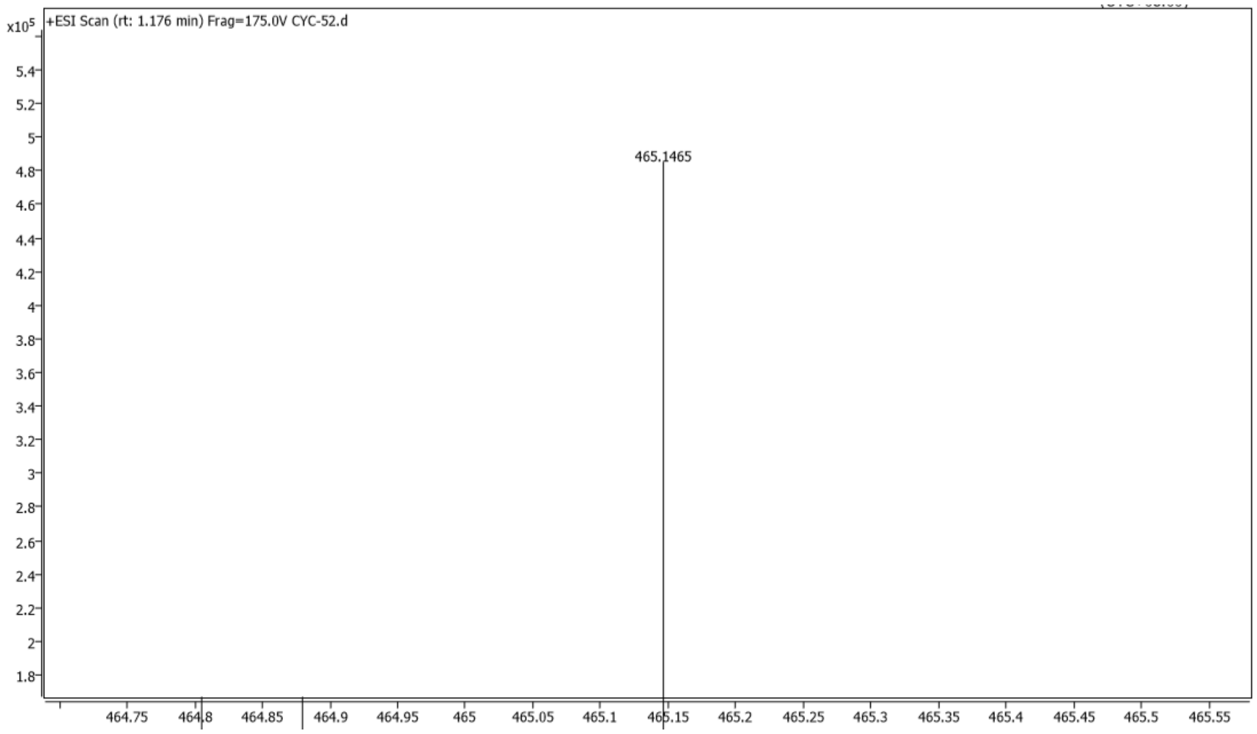

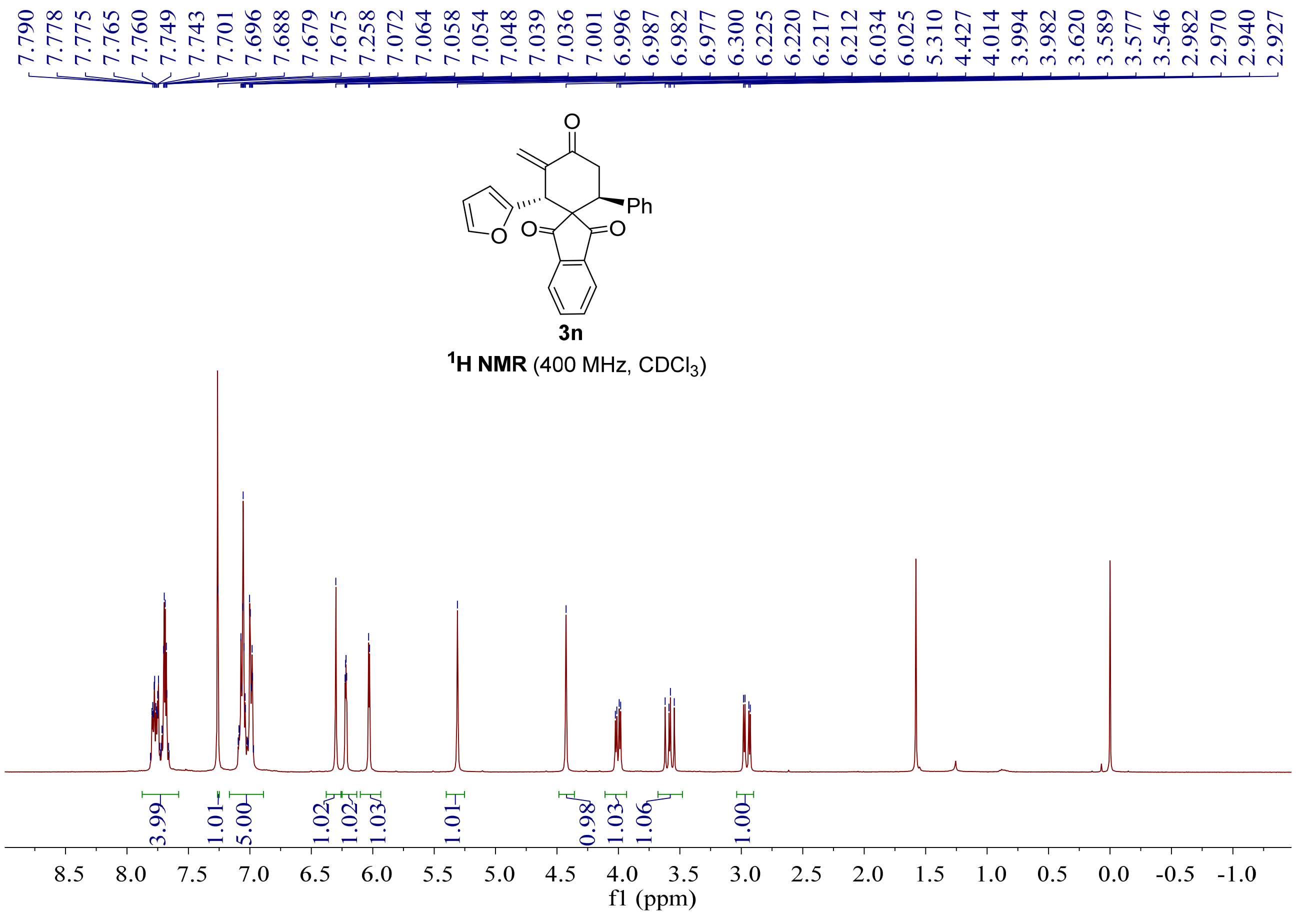
7.790 7.778 7.775 7.765 7.760 7.749 7.743 7.701 7.696 7.688 7.679 7.675 7.258 7.072 7.064 7.058 7.054 7.048 7.039 7.036 7.001 6.996 6.987 6.982 6.977 6.300 6.225 6.220 6.217 6.212 6.034 6.025 5.310 4.427 4.014 3.994 3.982 3.620 3.589 3.577 3.546 2.982 2.970 2.940 2.927
O
Ph
O
O
O
3n
1H NMR (400 MHz, CDCl3)
3.99 1.01 5.00 1.02 1.02 1.03 1.01 0.98 1.03 1.06 1.00
8.5 8.0 7.5 7.0 6.5 6.0 5.5 5.0 4.5 4.0 3.5 3.0 2.5 2.0 1.5 1.0 0.5 0.0 -0.5 -1.0
f1 (ppm)

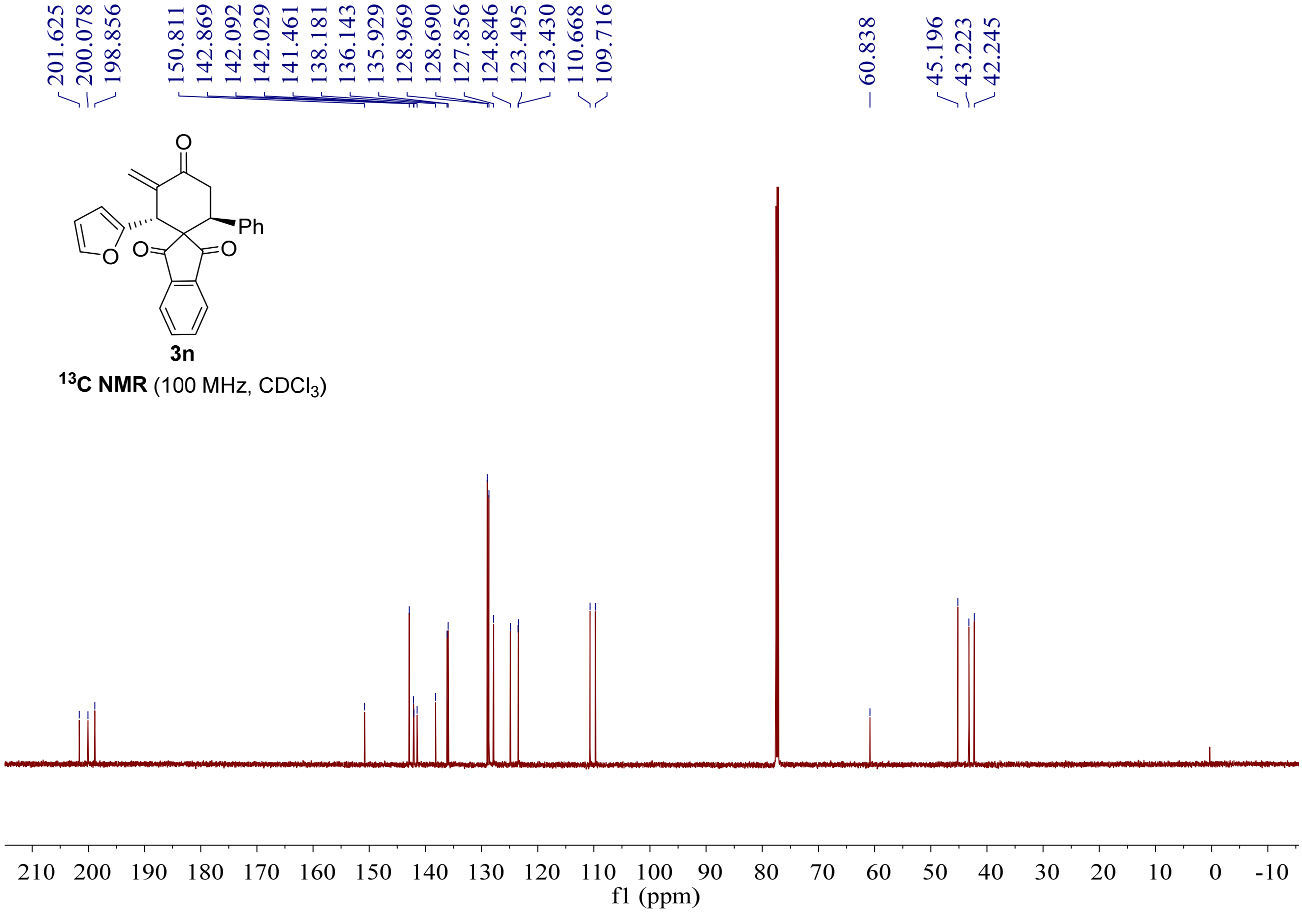
201.625 200.078 198.856 150.811 142.869 142.092 142.029 141.461 138.181 136.143 135.929 128.969 128.690 127.856 124.846 123.495 123.430 110.668 109.716 60.838 45.196 43.223 42.245
O
Ph
O
O
O
3n
13C NMR (100 MHz, CDCl3)
210 200 190 180 170 160 150 140 130 120 110 100 90 80 70 60 50 40 30 20 10 0 -10
f1 (ppm)

Daicel Chiral IC Column (*i*PrOH/*n*-hexane = 20/80, 1.0 mL/min)

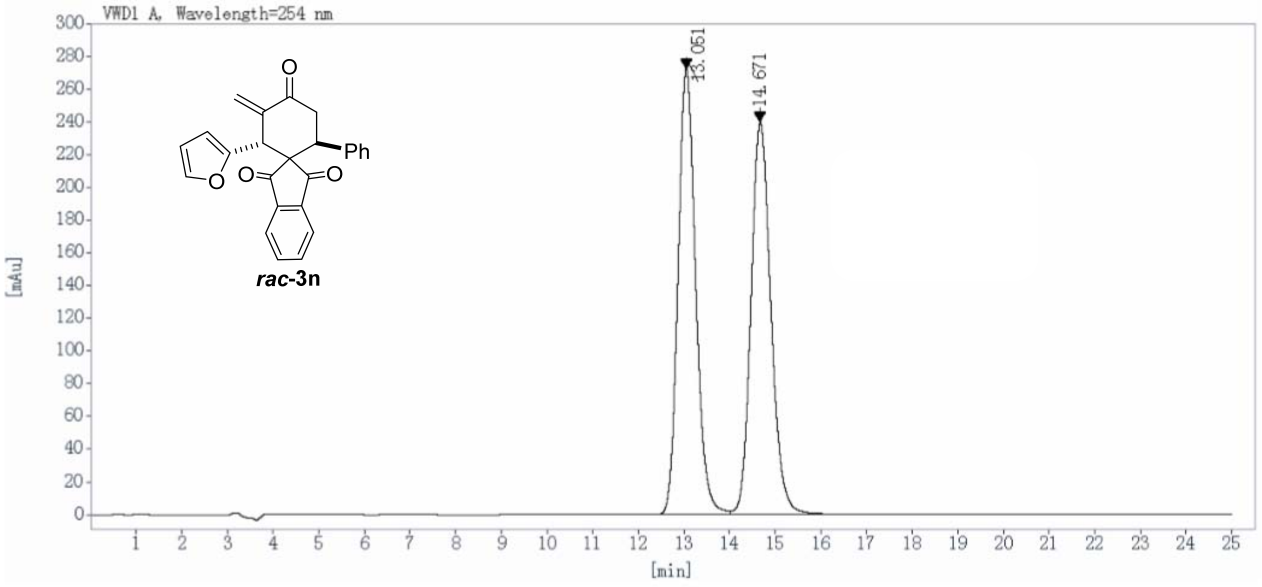

Ret Time [min]	Peak Type	Width [min]	Height [mAU]	Area [mAU*s]	Area [%]
13.051	BV	0.41	272.4947	7306.7109	50.0801
14.671	VB	0.47	239.8722	7283.3398	49.9199
			Totals:	14590.0508	100.0000

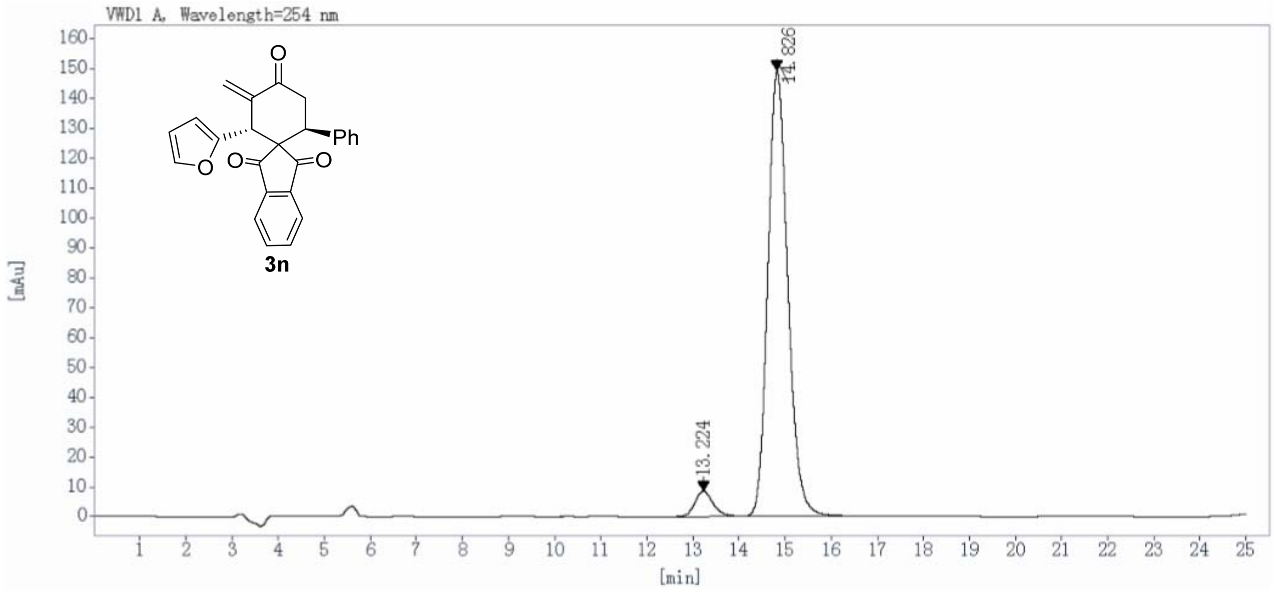

Ret Time [min]	Peak Type	Width [min]	Height [mAU]	Area [mAU*s]	Area [%]
13.224	BB	0.42	8.4319	230.3022	4.8723
14.826	BB	0.46	149.3270	4496.4780	95.1277
			Totals:	4726.7802	100.0000

HRMS (ESI-TOF) m/z: $[M + H]^+$ Calcd for $C_{25}H_{19}O_4^+$ 383.1278 ; Found 383.1277.

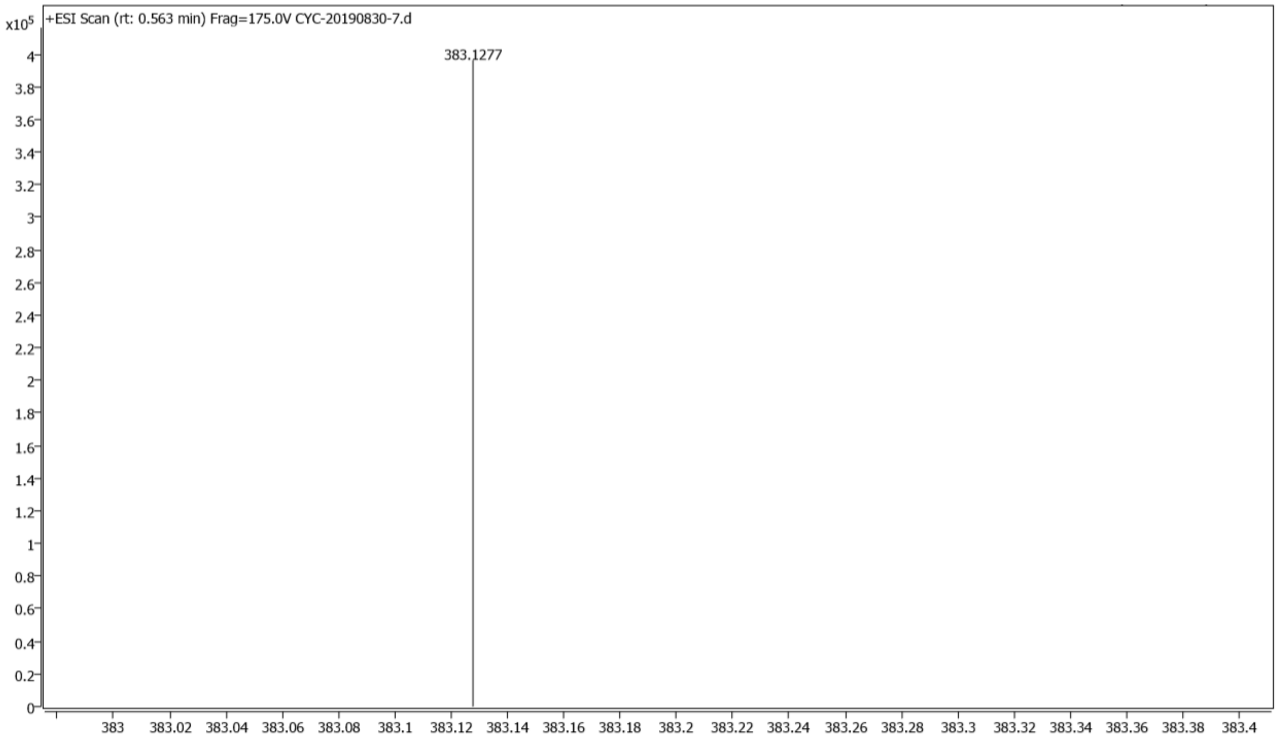

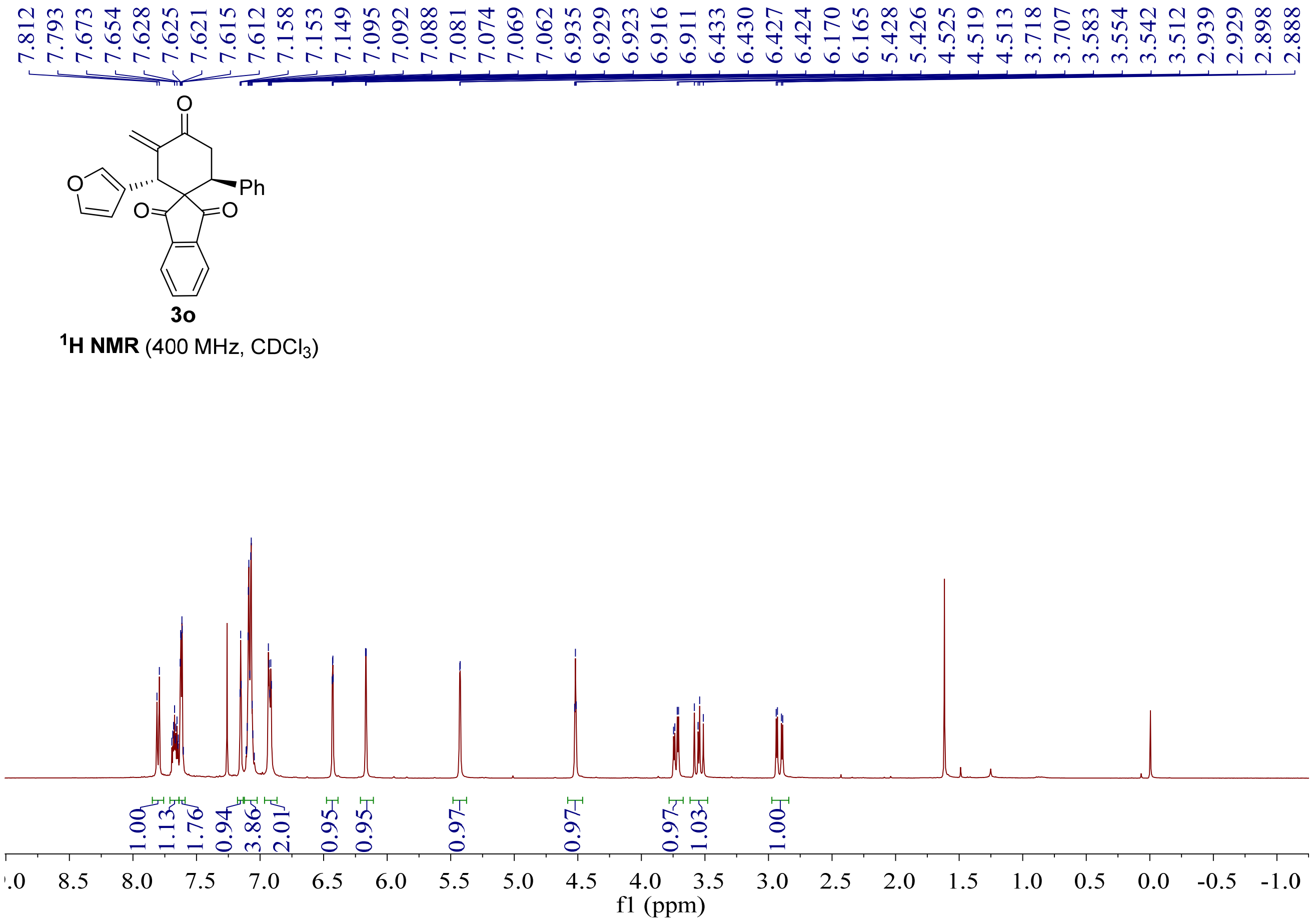

3o

¹H NMR (400 MHz, $CDCl_3$)

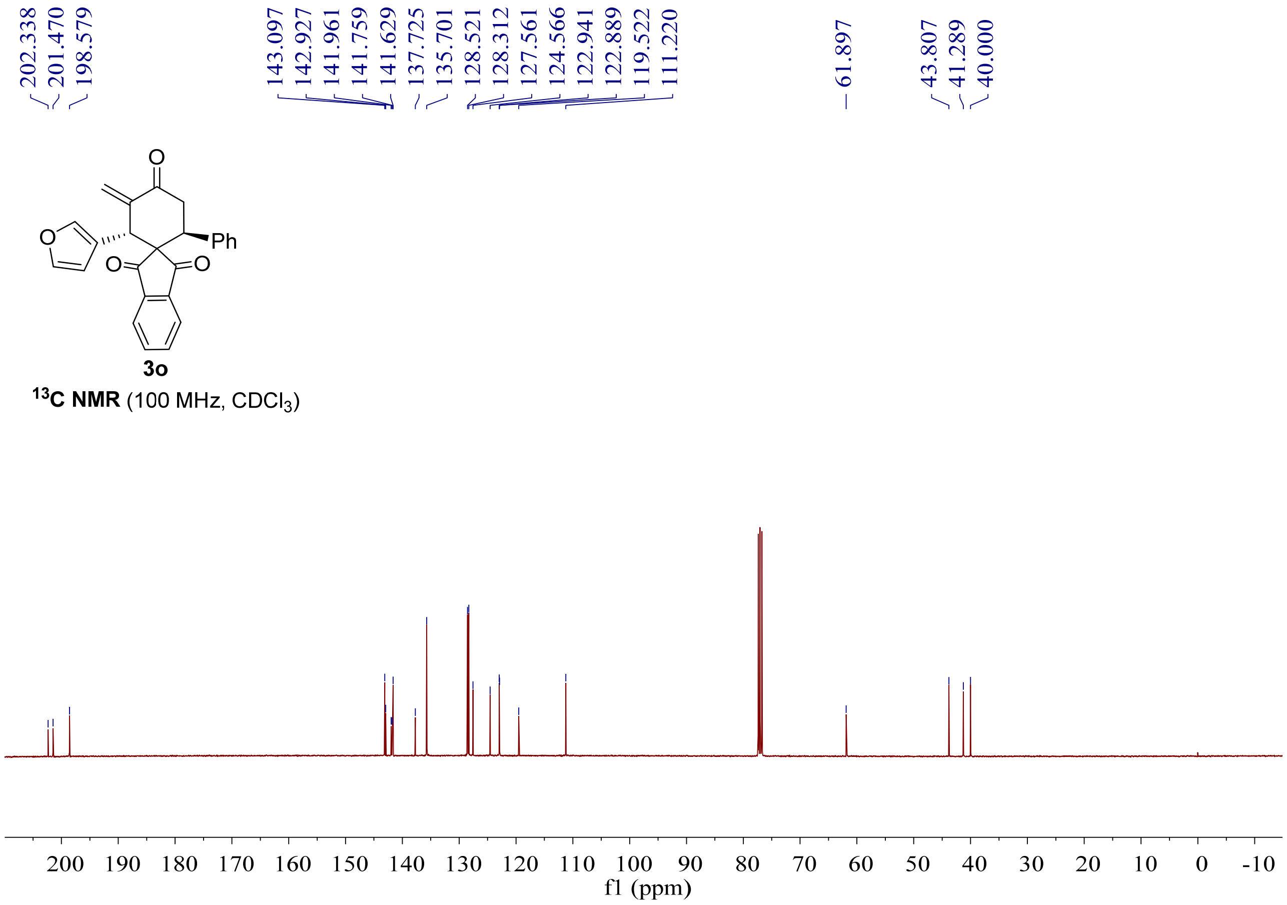

3o

¹³C NMR (100 MHz, $CDCl_3$)

Daicel Chiral IB Column (*i*PrOH/*n*-hexane = 10/90, 1.0 mL/min)

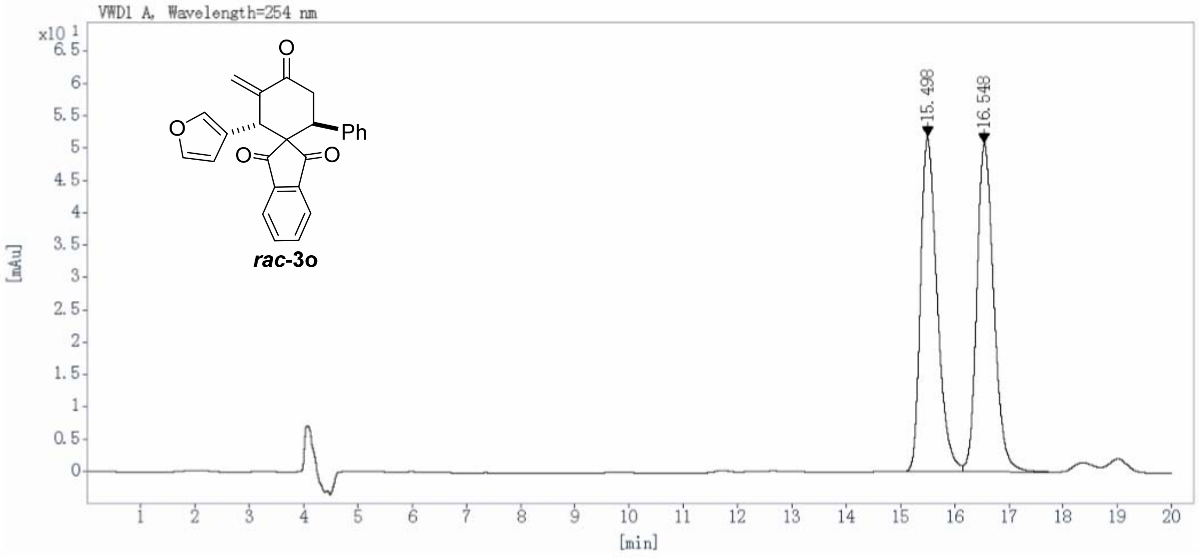

Ret Time [min]	Peak Type	Width [min]	Height [mAU]	Area [mAU*s]	Area [%]
15.498	BV	0.33	51.8397	1100.7234	49.7966
16.548	VB	0.34	50.8790	1109.7161	50.2034
			Totals:	2210.4395	100.0000

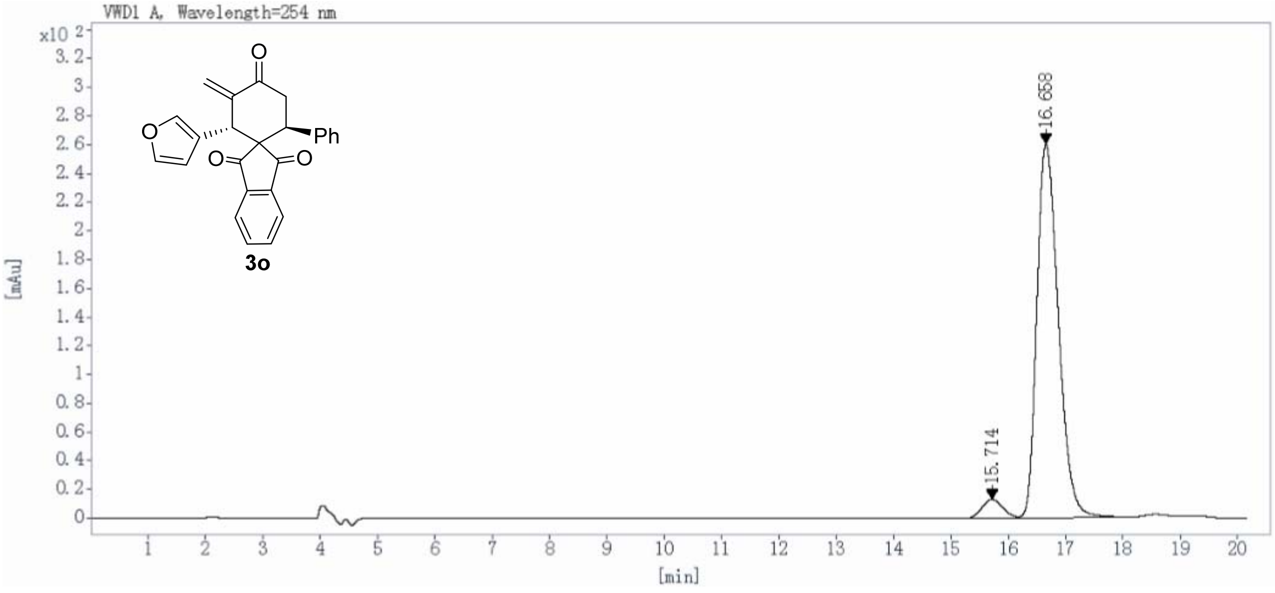

Ret Time [min]	Peak Type	Width [min]	Height [mAU]	Area [mAU*s]	Area [%]
15.714	MF	0.42	13.2275	329.6733	4.6444
16.658	FM	0.43	260.1070	6768.6870	95.3556
			Totals:	7098.3603	100.0000

HRMS (ESI-TOF) m/z: [M+ Na]$^+$ Calcd for $C_{25}H_{18}O_4Na^+$ 405.1097; Found 405.1096.

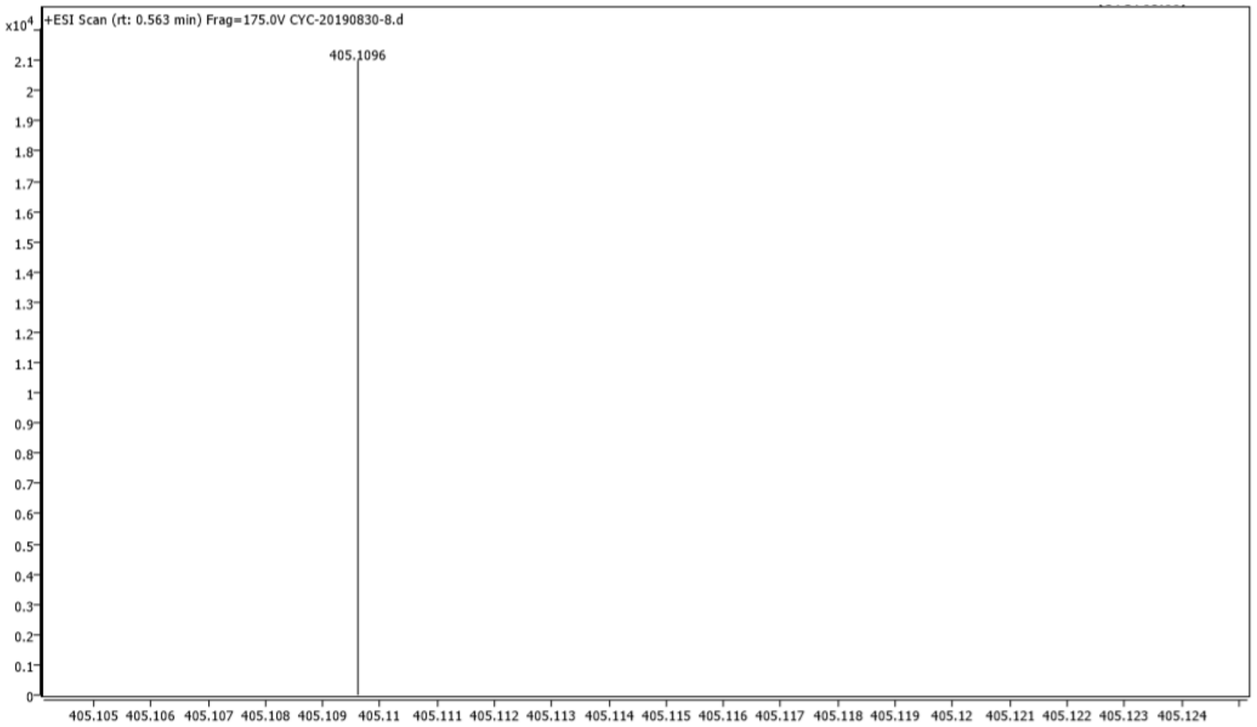

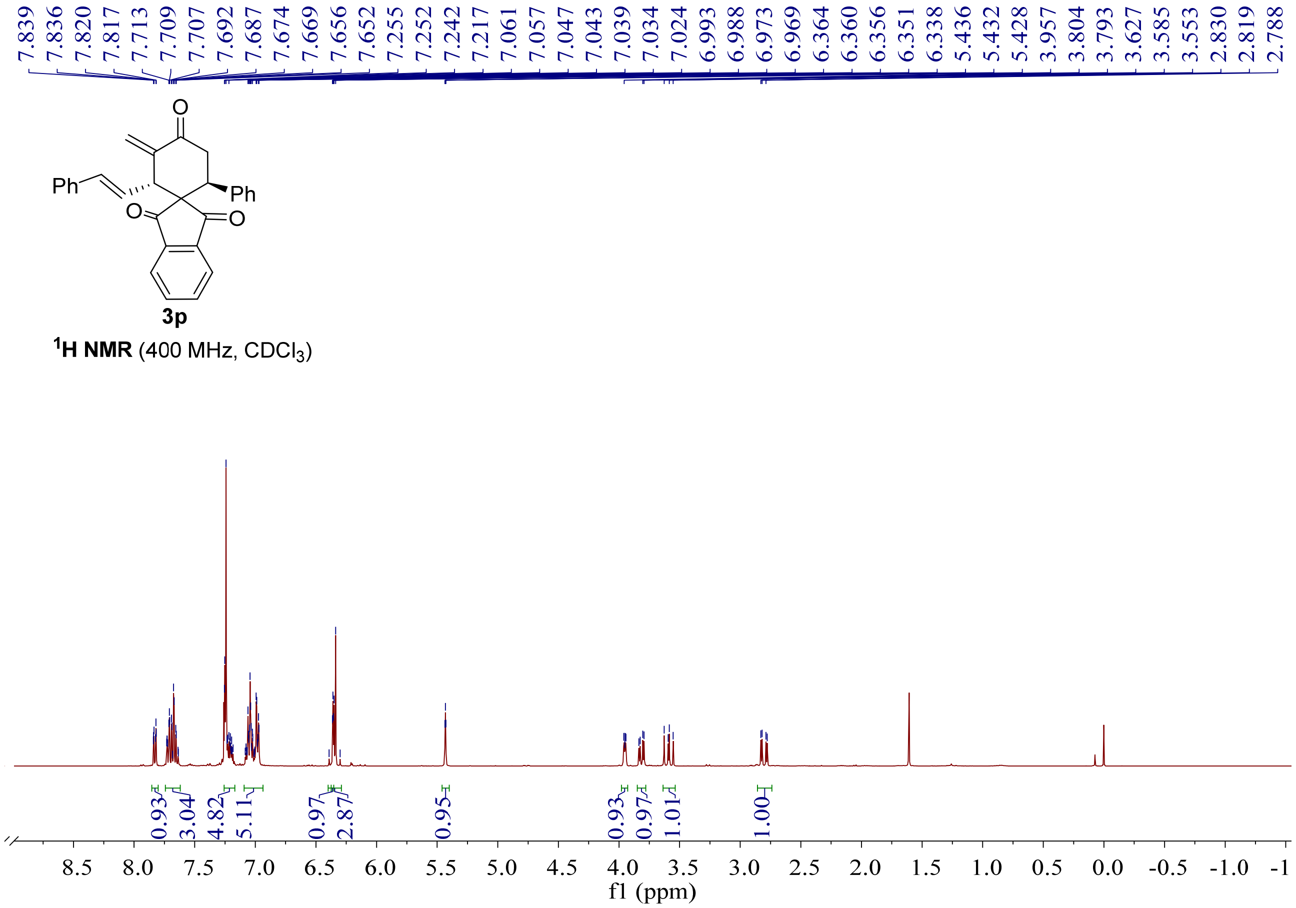
7.839
7.836
7.820
7.817
7.713
7.709
7.707
7.692
7.687
7.674
7.669
7.656
7.652
7.255
7.252
7.242
7.217
7.061
7.057
7.047
7.043
7.039
7.034
7.024
6.993
6.988
6.973
6.969
6.364
6.360
6.356
6.351
6.338
5.436
5.432
5.428
3.957
3.804
3.793
3.627
3.585
3.553
2.830
2.819
2.788
Ph
Ph
O
O
O
3p
^{1}H NMR (400 MHz, CDCl$_3$)
0.93
3.04
4.82
5.11
0.97
2.87
0.95
0.93
0.97
1.01
1.00
8.5 8.0 7.5 7.0 6.5 6.0 5.5 5.0 4.5 4.0 3.5 3.0 2.5 2.0 1.5 1.0 0.5 0.0 -0.5 -1.0 -1
f1 (ppm)

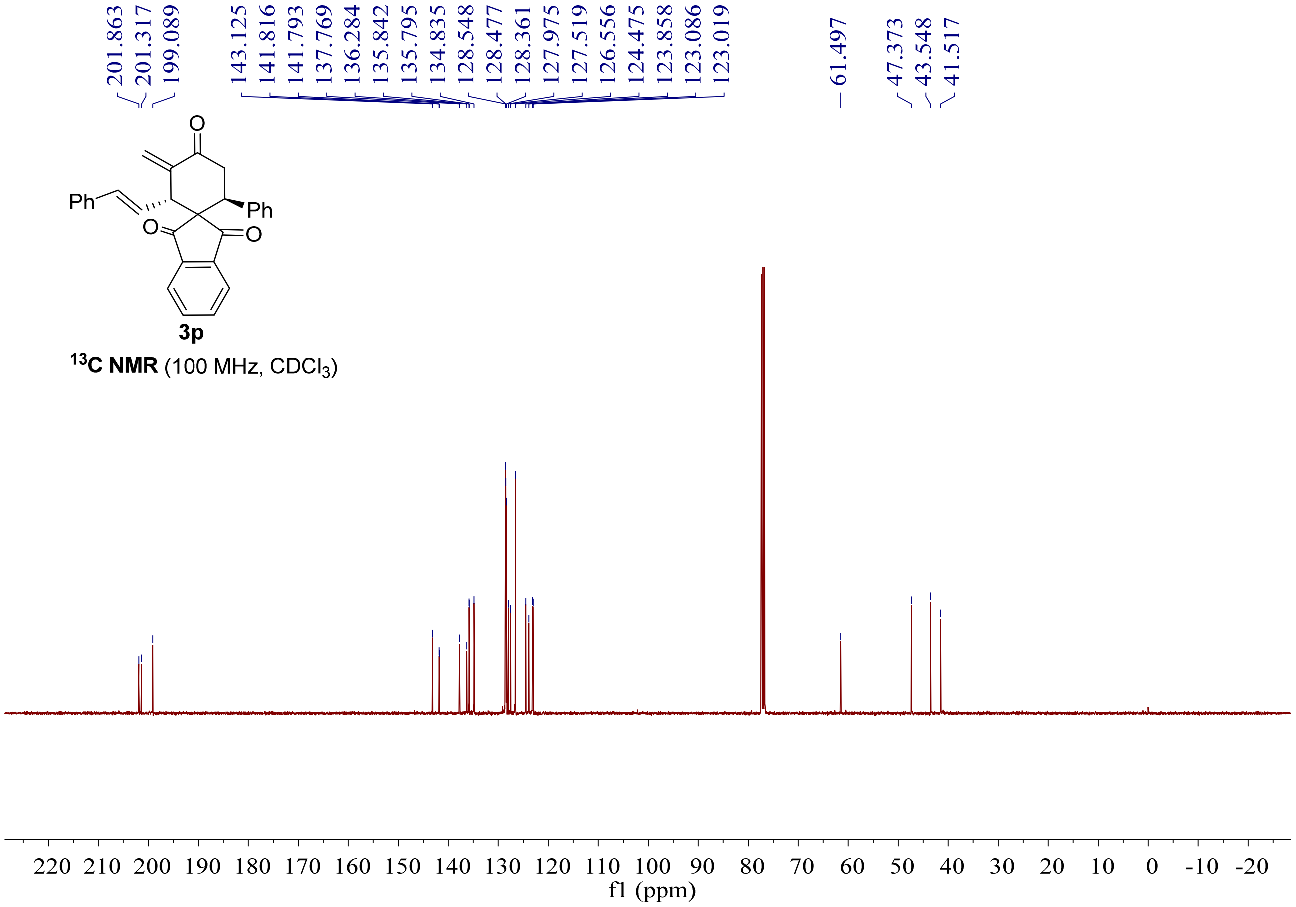
201.863
201.317
199.089
143.125
141.816
141.793
137.769
136.284
135.842
135.795
134.835
128.548
128.477
128.361
127.975
127.519
126.556
124.475
123.858
123.086
123.019
61.497
47.373
43.548
41.517
Ph
Ph
O
O
O
3p
^{13}C NMR (100 MHz, CDCl$_3$)
220 210 200 190 180 170 160 150 140 130 120 110 100 90 80 70 60 50 40 30 20 10 0 -10 -20
f1 (ppm)

Daicel Chiral IF Column (*i*PrOH/*n*-hexane = 40/60, 1.0 mL/min)

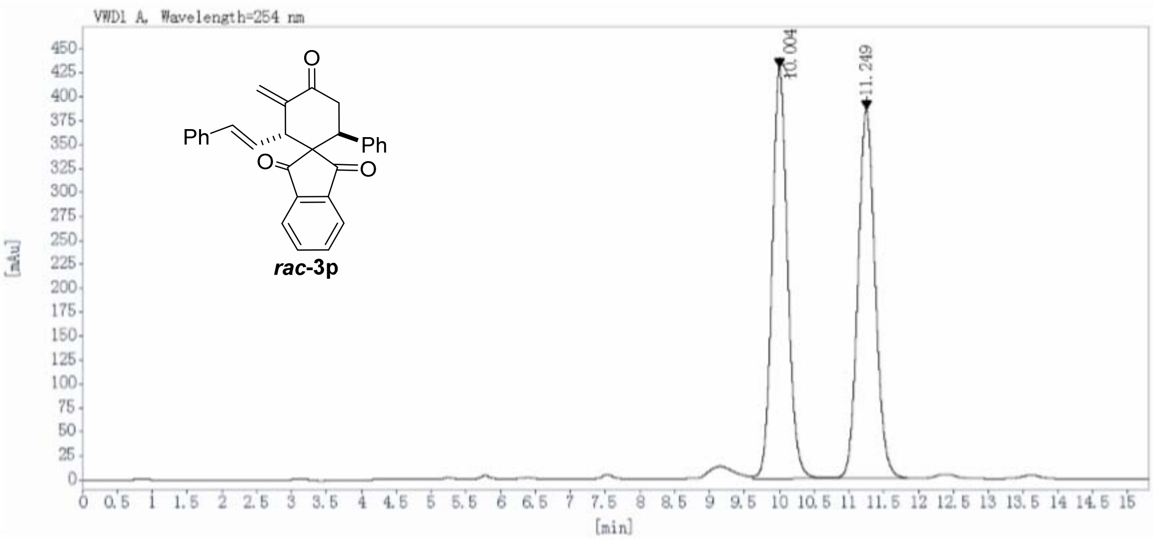

Ret Time [min]	Peak Type	Width [min]	Height [mAU]	Area [mAU*s]	Area [%]
10.004	FM	0.26	429.4279	6650.0313	50.1699
11.249	VB	0.27	384.5505	6605.0024	49.8301
			Totals:	13255.0337	100.0000

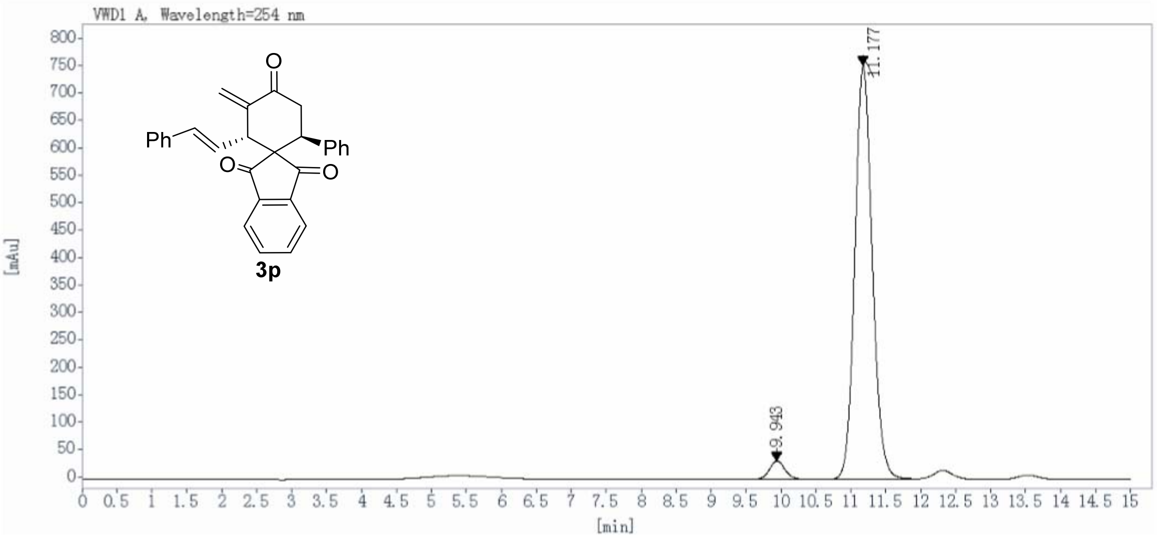

Ret Time [min]	Peak Type	Width [min]	Height [mAU]	Area [mAU*s]	Area [%]
9.943	BB	0.24	33.8376	529.5729	3.8780
11.177	MF	0.29	754.8874	13126.1221	96.1220
			Totals:	13655.6950	100.0000

HRMS (ESI-TOF) m/z: $[M+ Na]^+$ Calcd for $C_{29}H_{22}O_3Na^+$ 441.1461; Found 441.1463.

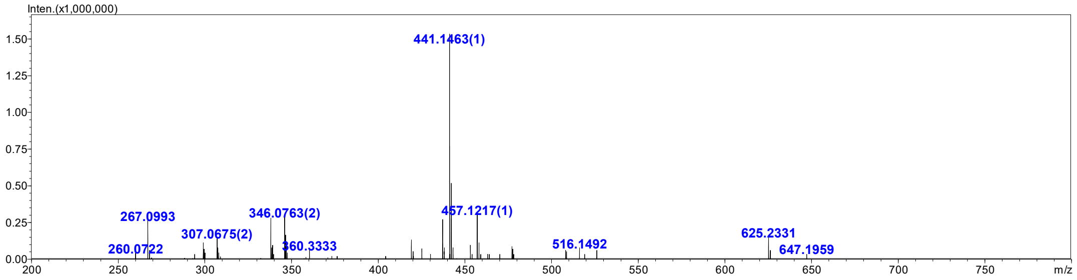

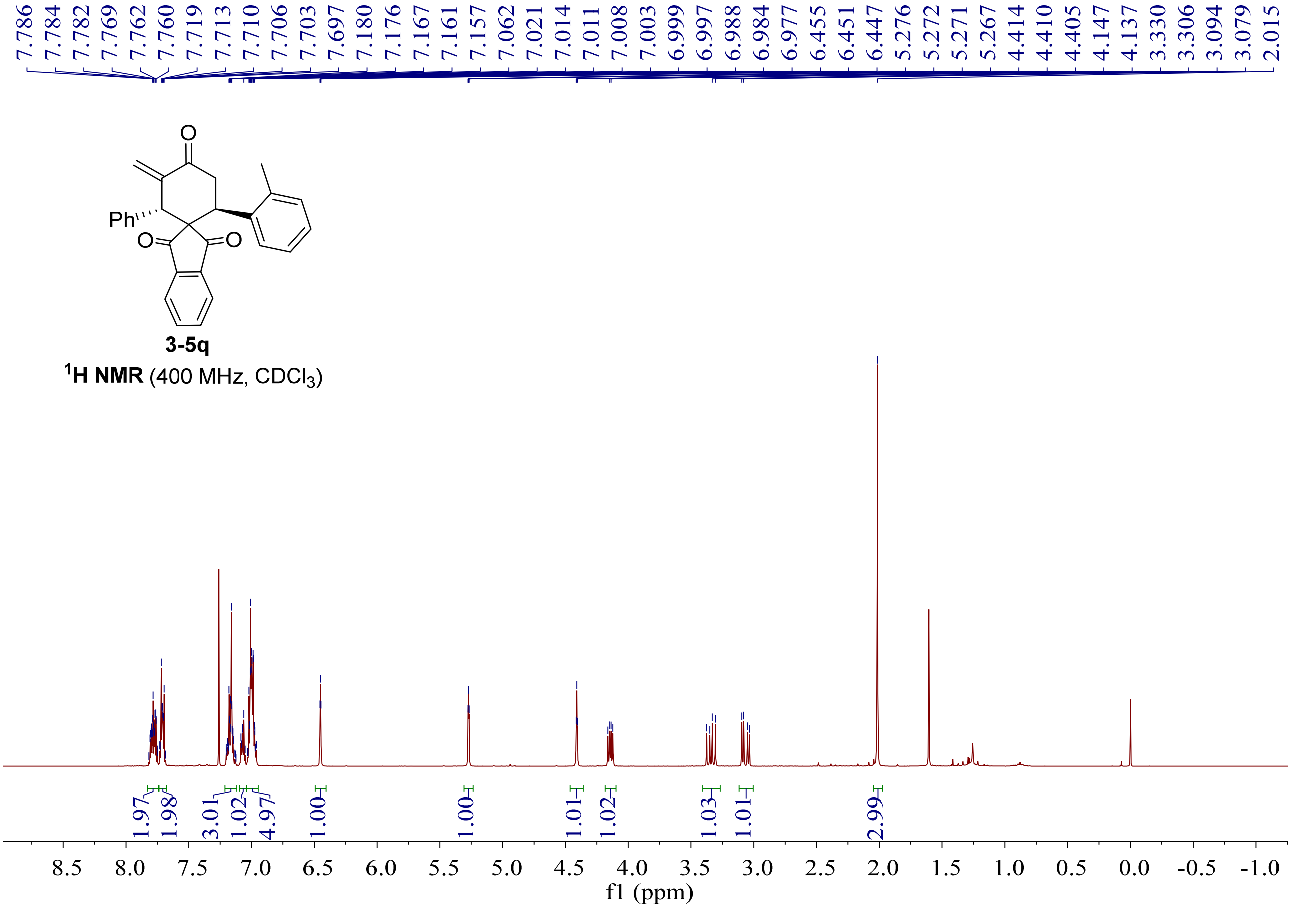
7.786 7.784 7.782 7.769 7.762 7.760 7.719 7.713 7.710 7.706 7.703 7.697 7.180 7.176 7.167 7.161 7.157 7.062 7.021 7.014 7.011 7.008 7.003 6.999 6.997 6.988 6.984 6.977 6.455 6.451 6.447 5.276 5.272 5.271 5.267 4.414 4.410 4.405 4.147 4.137 3.330 3.306 3.094 3.079 2.015
O
Ph
O
O
3-5q
1H NMR (400 MHz, CDCl3)
1.97 1.98 3.01 1.02 4.97 1.00 1.00 1.01 1.02 1.03 1.01 2.99
8.5 8.0 7.5 7.0 6.5 6.0 5.5 5.0 4.5 4.0 3.5 3.0 2.5 2.0 1.5 1.0 0.5 0.0 -0.5 -1.0
f1 (ppm)

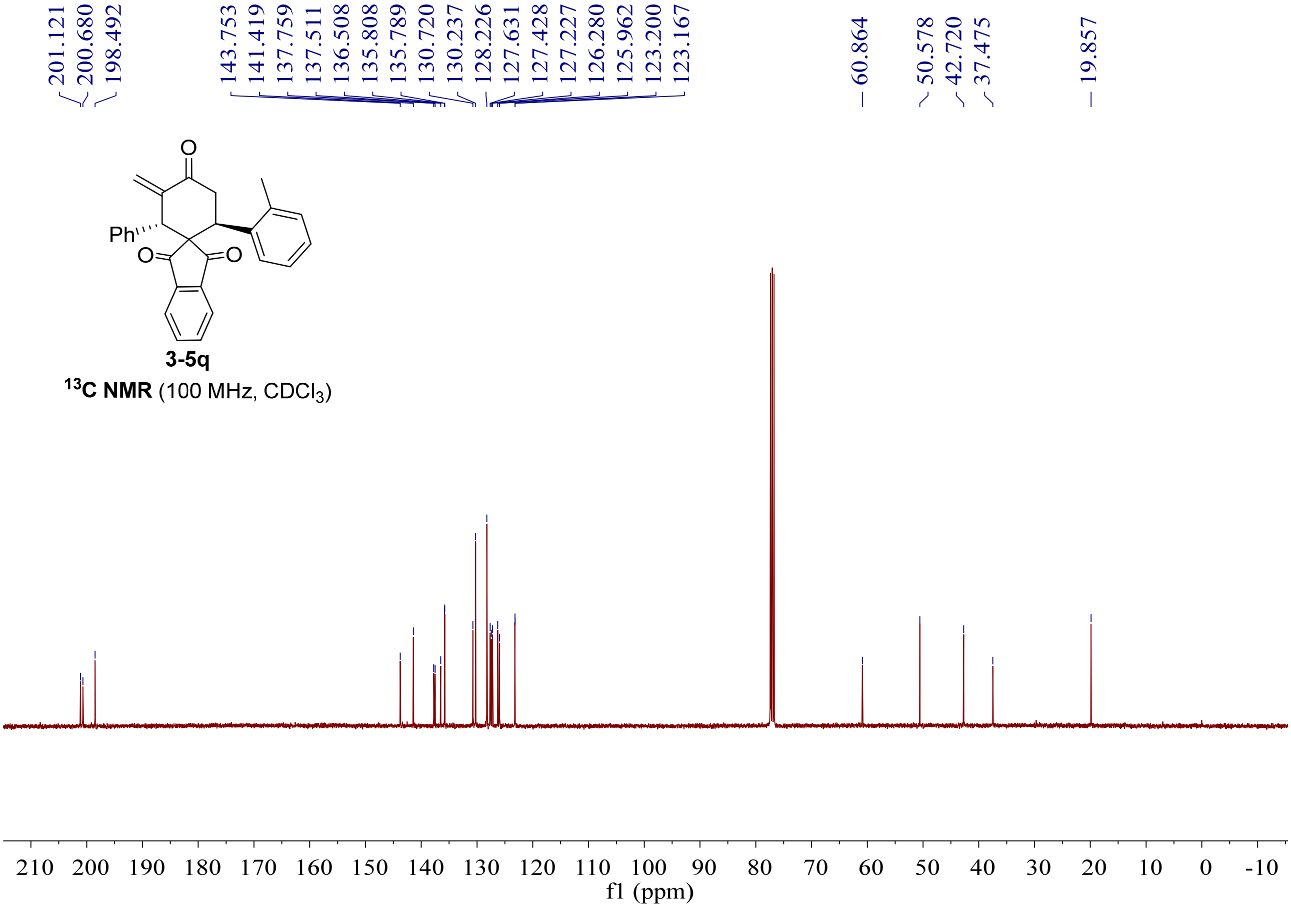
201.121 200.680 198.492 143.753 141.419 137.759 137.511 136.508 135.808 135.789 130.720 130.237 128.226 127.631 127.428 127.227 126.280 125.962 123.200 123.167 60.864 50.578 42.720 37.475 19.857
O
Ph
O
O
3-5q
13C NMR (100 MHz, CDCl3)
210 200 190 180 170 160 150 140 130 120 110 100 90 80 70 60 50 40 30 20 10 0 -10
f1 (ppm)

Daicel Chiral ID Column (*i*PrOH/*n*-hexane = 20/80, 1.0 mL/min)

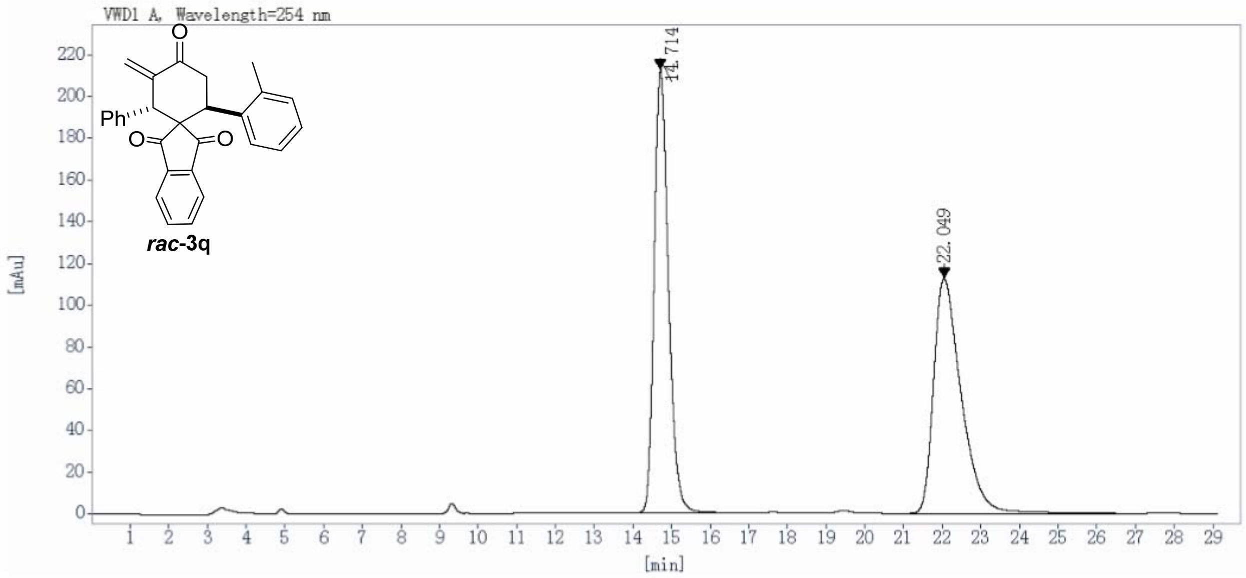

Ret Time [min]	Peak Type	Width [min]	Height [mAU]	Area [mAU*s]	Area [%]
14.714	BB	0.39	212.4359	5418.5640	49.2575
22.049	BB	0.74	112.6765	5581.9253	50.7425
			Totals:	11000.4893	100.0000

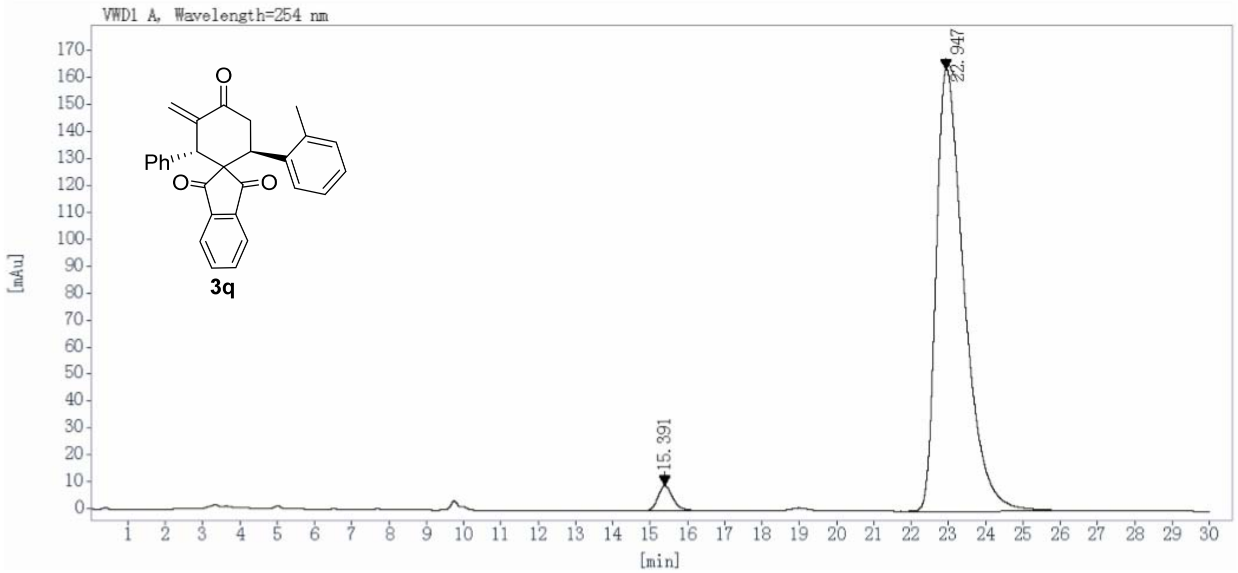

Ret Time [min]	Peak Type	Width [min]	Height [mAU]	Area [mAU*s]	Area [%]
15.391	BB	0.42	9.2687	253.1785	2.8725
22.947	BB	0.79	164.1081	8560.6797	97.1275
			Totals:	8813.8581	100.0000

HRMS (ESI-TOF) m/z: [M + Na]$^+$ Calcd for $C_{28}H_{22}O_3Na^+$ 429.1461; Found 429.1490.

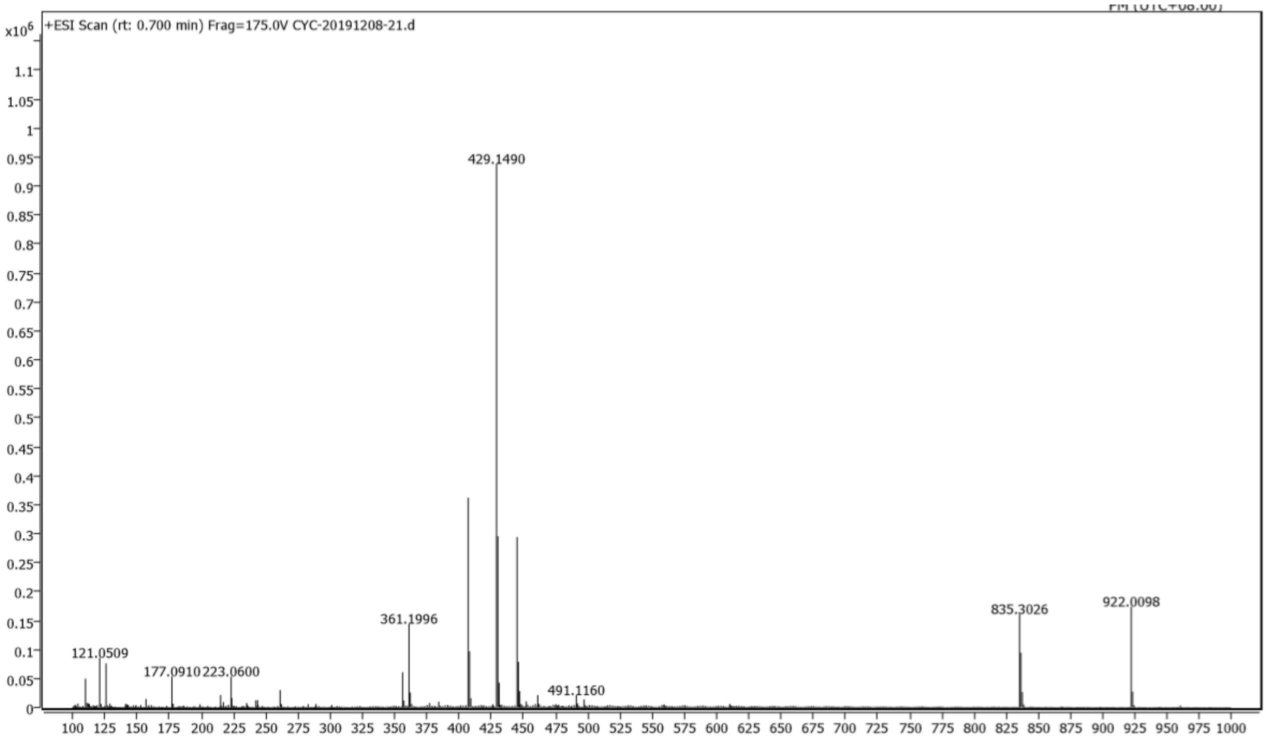

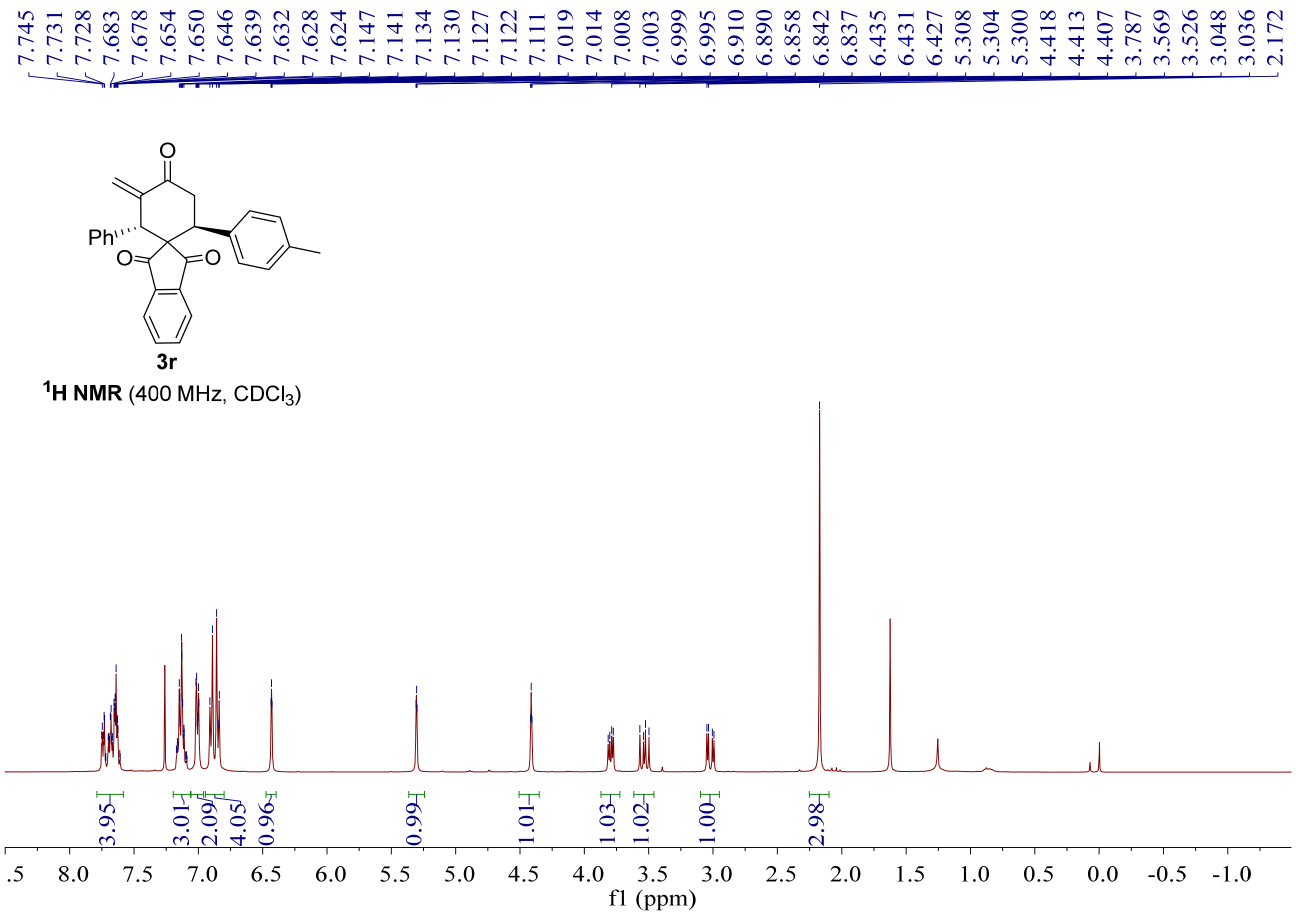
7.745
7.731
7.728
7.683
7.678
7.654
7.650
7.646
7.639
7.632
7.628
7.624
7.147
7.141
7.134
7.130
7.127
7.122
7.111
7.019
7.014
7.008
7.003
6.999
6.995
6.910
6.890
6.858
6.842
6.837
6.435
6.431
6.427
5.308
5.304
5.300
4.418
4.413
4.407
3.787
3.569
3.526
3.048
3.036
2.172
Ph
O
3r
^{1}H NMR (400 MHz, $CDCl_3$)
3.95
3.01
2.09
4.05
0.96
0.99
1.01
1.03
1.02
1.00
2.98
.5
8.0
7.5
7.0
6.5
6.0
5.5
5.0
4.5
4.0
3.5
3.0
2.5
2.0
1.5
1.0
0.5
0.0
-0.5
-1.0
f1 (ppm)

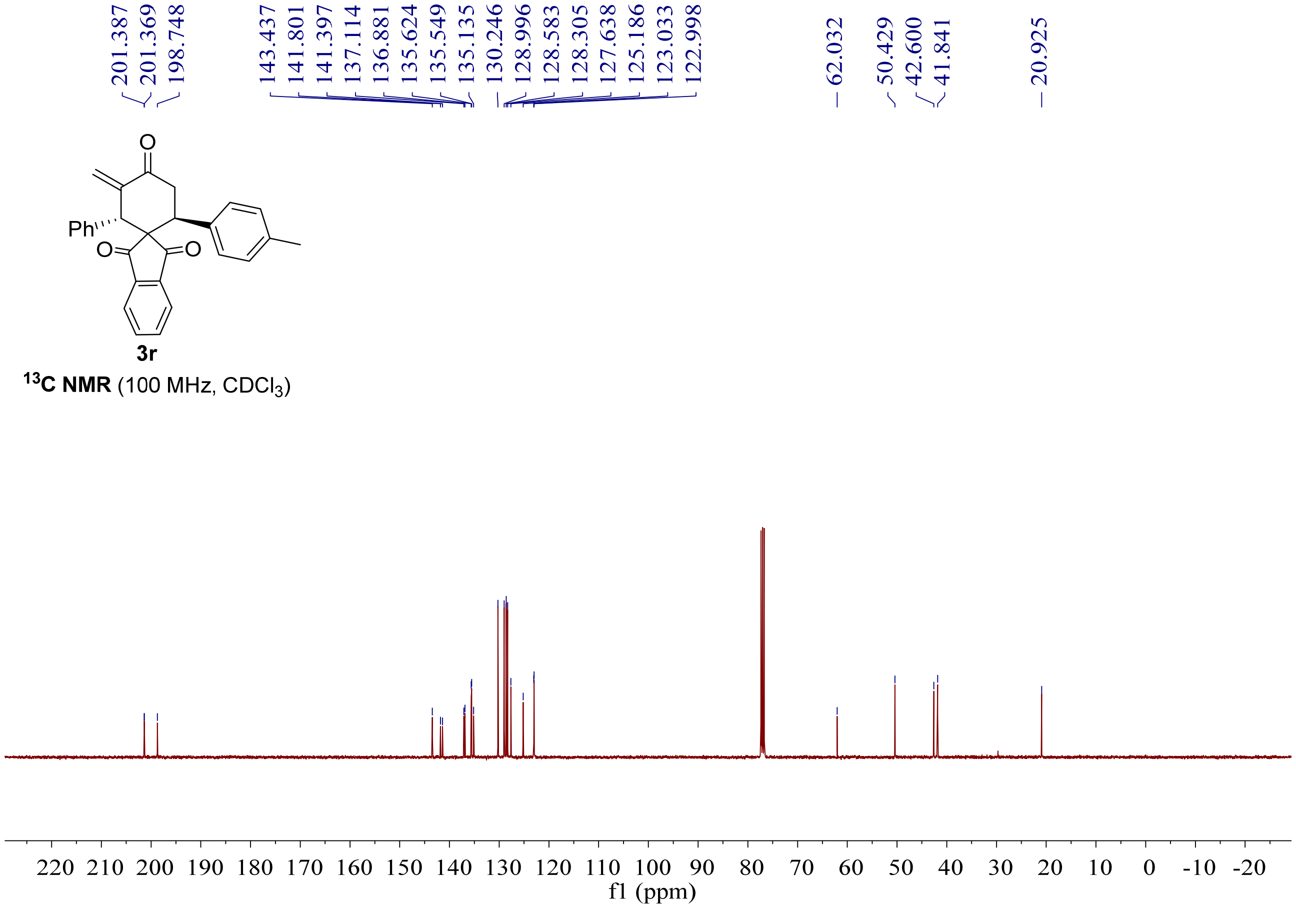
201.387
201.369
198.748
143.437
141.801
141.397
137.114
136.881
135.624
135.549
135.135
130.246
128.996
128.583
128.305
127.638
125.186
123.033
122.998
62.032
50.429
42.600
41.841
20.925
Ph
O
3r
^{13}C NMR (100 MHz, $CDCl_3$)
220
210
200
190
180
170
160
150
140
130
120
110
100
90
80
70
60
50
40
30
20
10
0
-10
-20
f1 (ppm)

Daicel Chiral IB Column (*i*PrOH/*n*-hexane = 20/80, 1.0 mL/min)

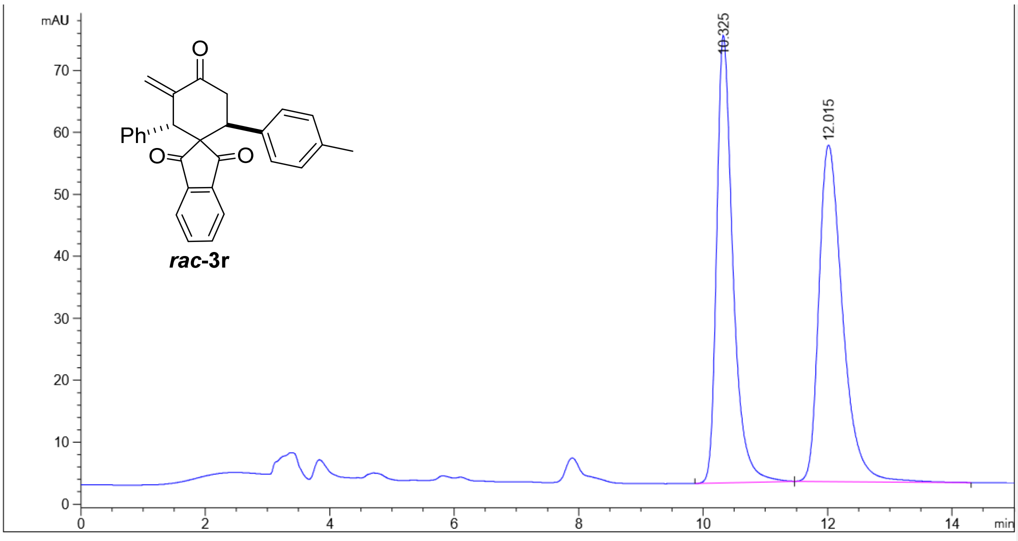

Time	Area (%)
10.325	47.7
12.015	52.3

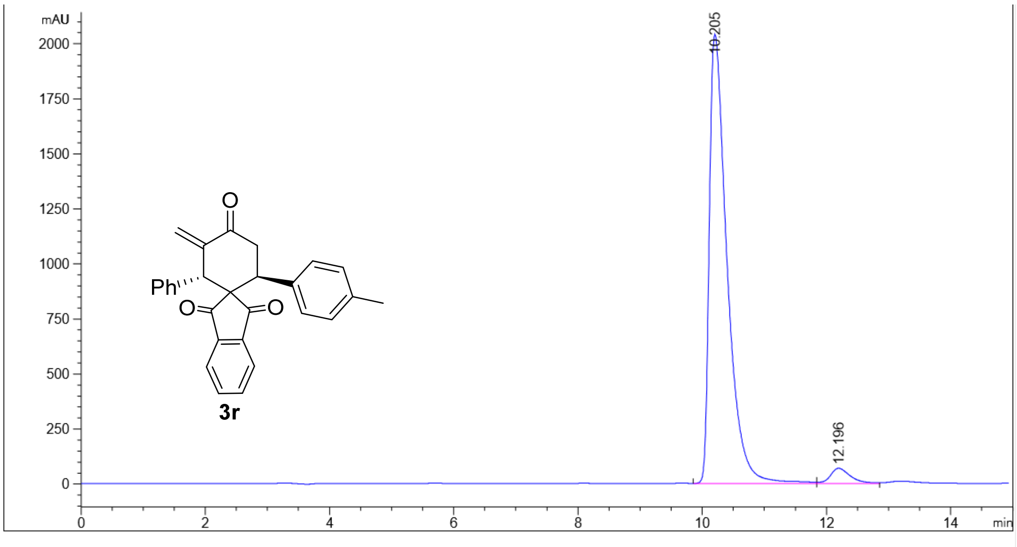

Time	Area (%)
10.205	96.3
12.196	3.7

HRMS (ESI-TOF) m/z: $[M + Na]^+$ Calcd for $C_{28}H_{22}O_3Na^+$ 429.1461; Found 429.1460.

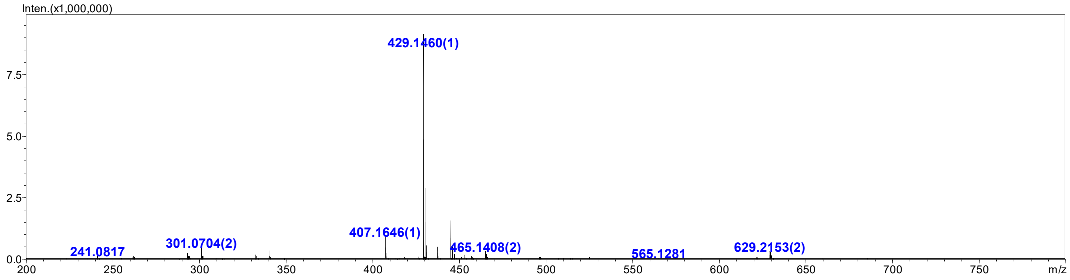

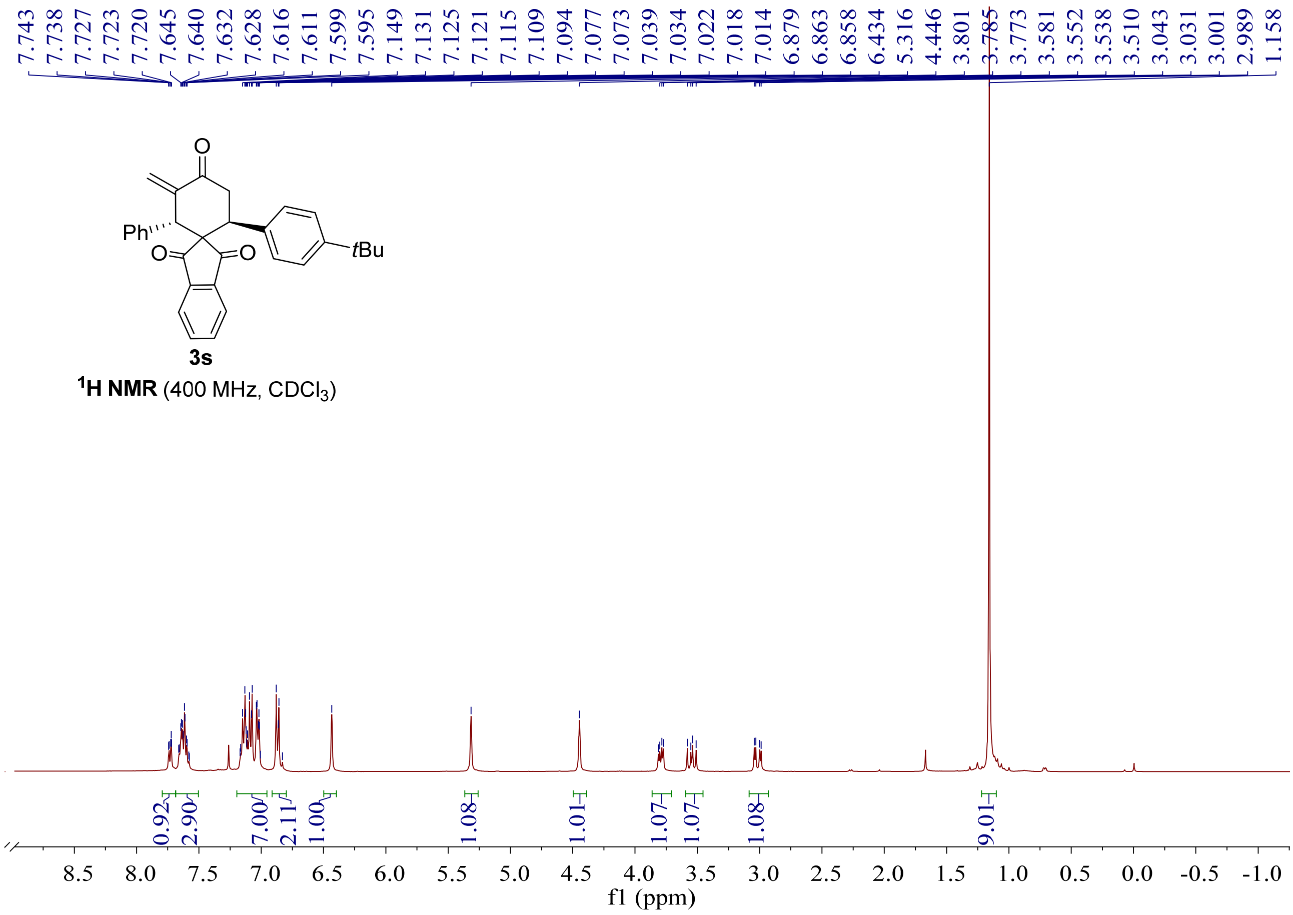
7.743 7.738 7.727 7.723 7.720 7.645 7.640 7.632 7.628 7.616 7.611 7.599 7.595 7.149 7.131 7.125 7.121 7.115 7.109 7.094 7.077 7.073 7.039 7.034 7.022 7.018 7.014 6.879 6.863 6.858 6.434 5.316 4.446 3.801 3.785 3.773 3.581 3.552 3.538 3.510 3.043 3.031 3.001 2.989 1.158
O
Ph
O
O
tBu
3s
¹H NMR (400 MHz, CDCl3)
0.92 2.90 7.00 2.11 1.00 1.08 1.01 1.07 1.07 1.08 9.01
8.5 8.0 7.5 7.0 6.5 6.0 5.5 5.0 4.5 4.0 3.5 3.0 2.5 2.0 1.5 1.0 0.5 0.0 -0.5 -1.0
f1 (ppm)

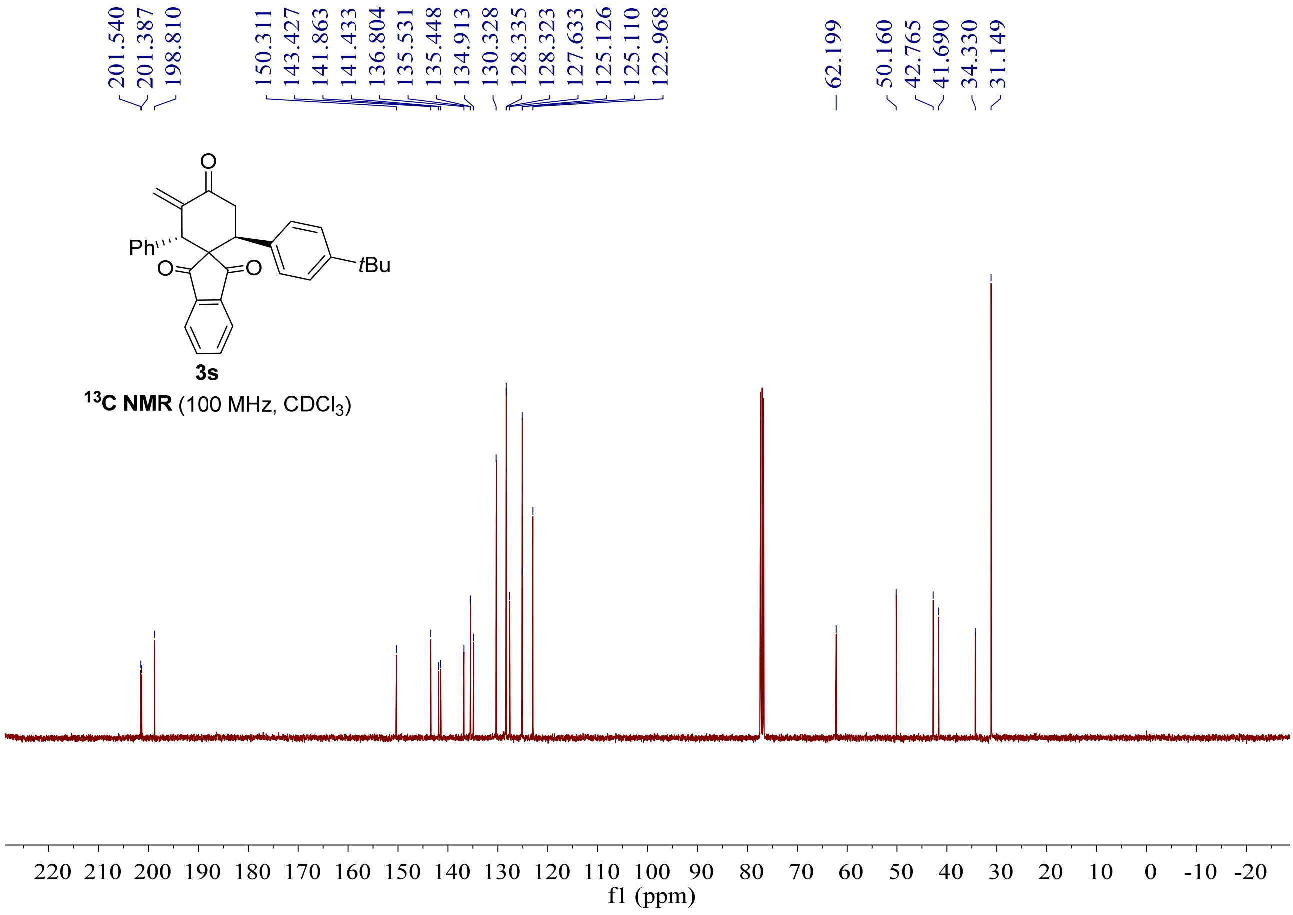
201.540 201.387 198.810 150.311 143.427 141.863 141.433 136.804 135.531 135.448 134.913 130.328 128.335 128.323 127.633 125.126 125.110 122.968 62.199 50.160 42.765 41.690 34.330 31.149
O
Ph
O
O
tBu
3s
¹³C NMR (100 MHz, CDCl3)
220 210 200 190 180 170 160 150 140 130 120 110 100 90 80 70 60 50 40 30 20 10 0 -10 -20
f1 (ppm)

Daicel Chiral AD-H Column (*i*PrOH/*n*-hexane = 30/70, 1.0 mL/min)

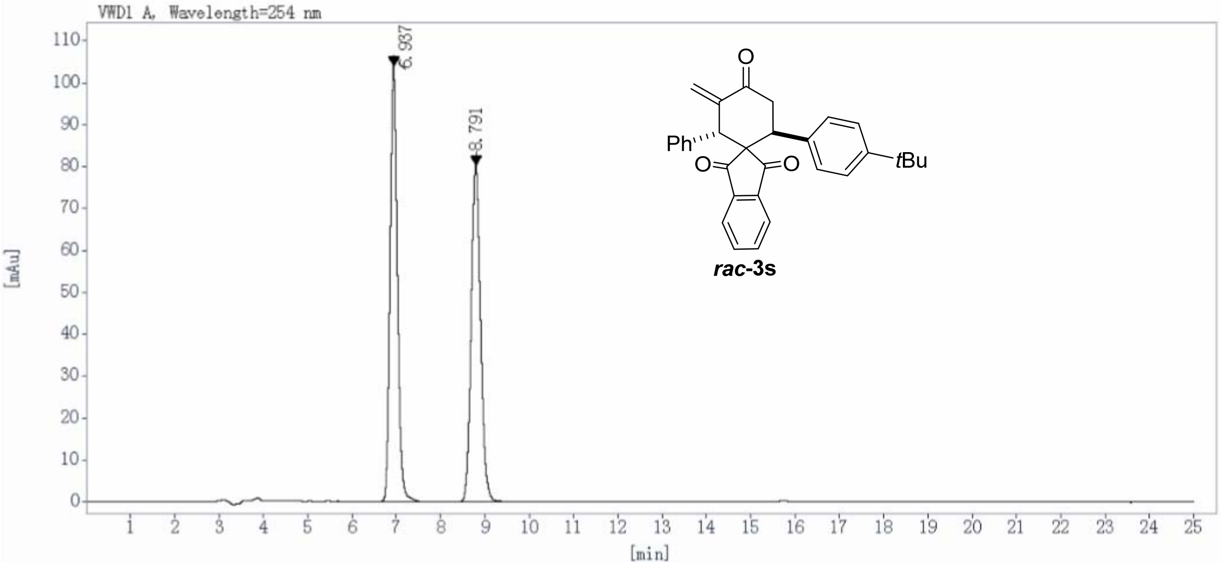

Ret Time [min]	Peak Type	Width [min]	Height [mAU]	Area [mAU*s]	Area [%]
6.937	BB	0.18	103.9956	1197.3231	50.2877
8.791	BBA	0.23	80.1533	1183.6246	49.7123
			Totals:	2380.9478	100.0000

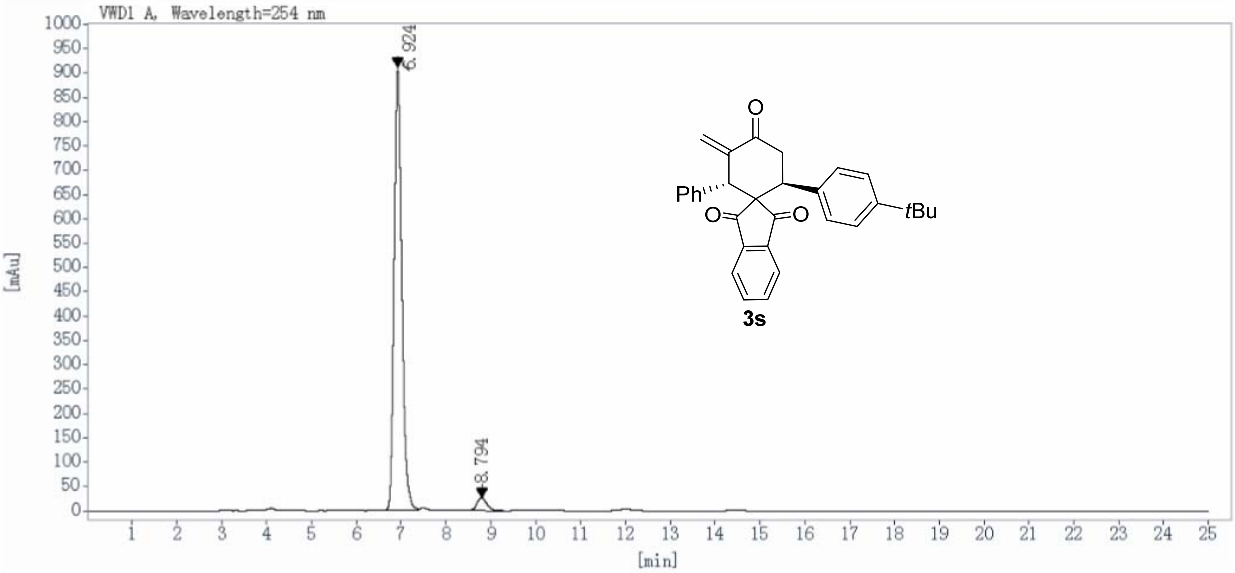

Ret Time [min]	Peak Type	Width [min]	Height [mAU]	Area [mAU*s]	Area [%]
6.924	MF	0.20	911.7954	10873.9873	96.5194
8.794	BB	0.23	26.1442	392.1301	3.4806
			Totals:	11266.1174	100.0000

HRMS (ESI-TOF) m/z: [M + Na]$^+$ Calcd for $C_{31}H_{28}O_3Na^+$ 471.1931; Found 471.1944.

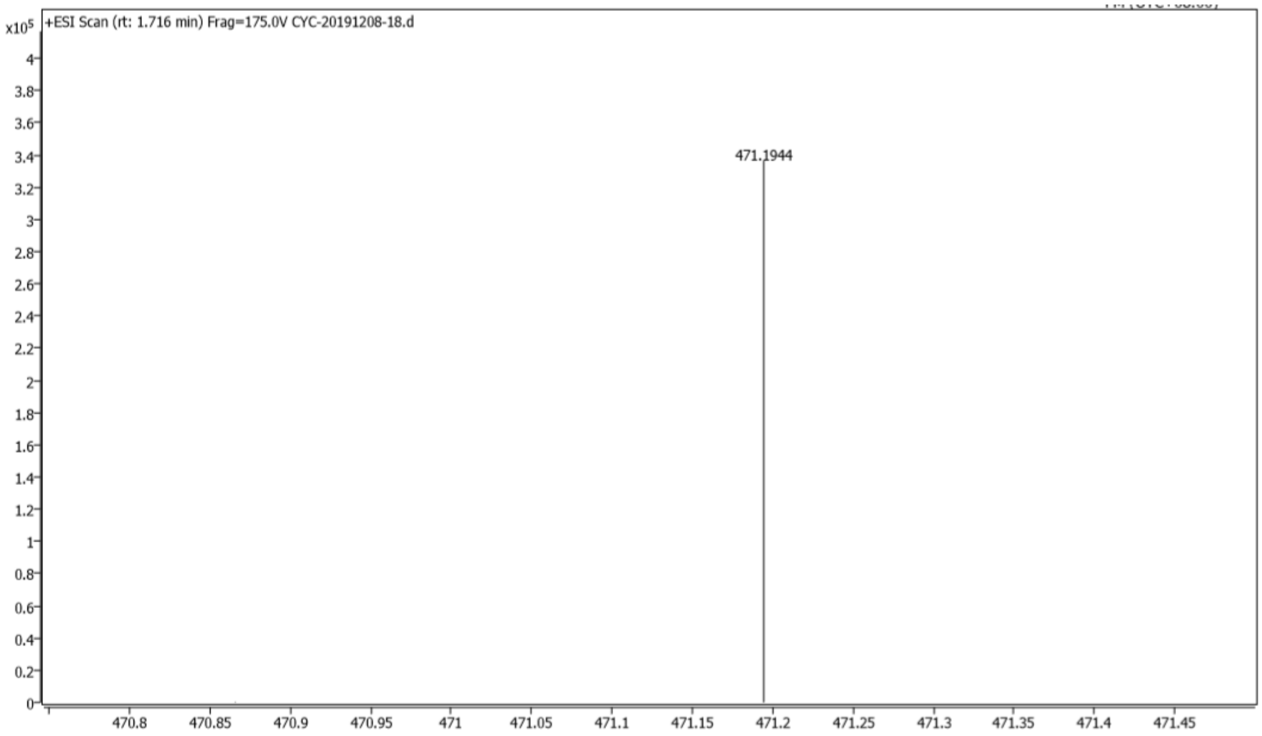

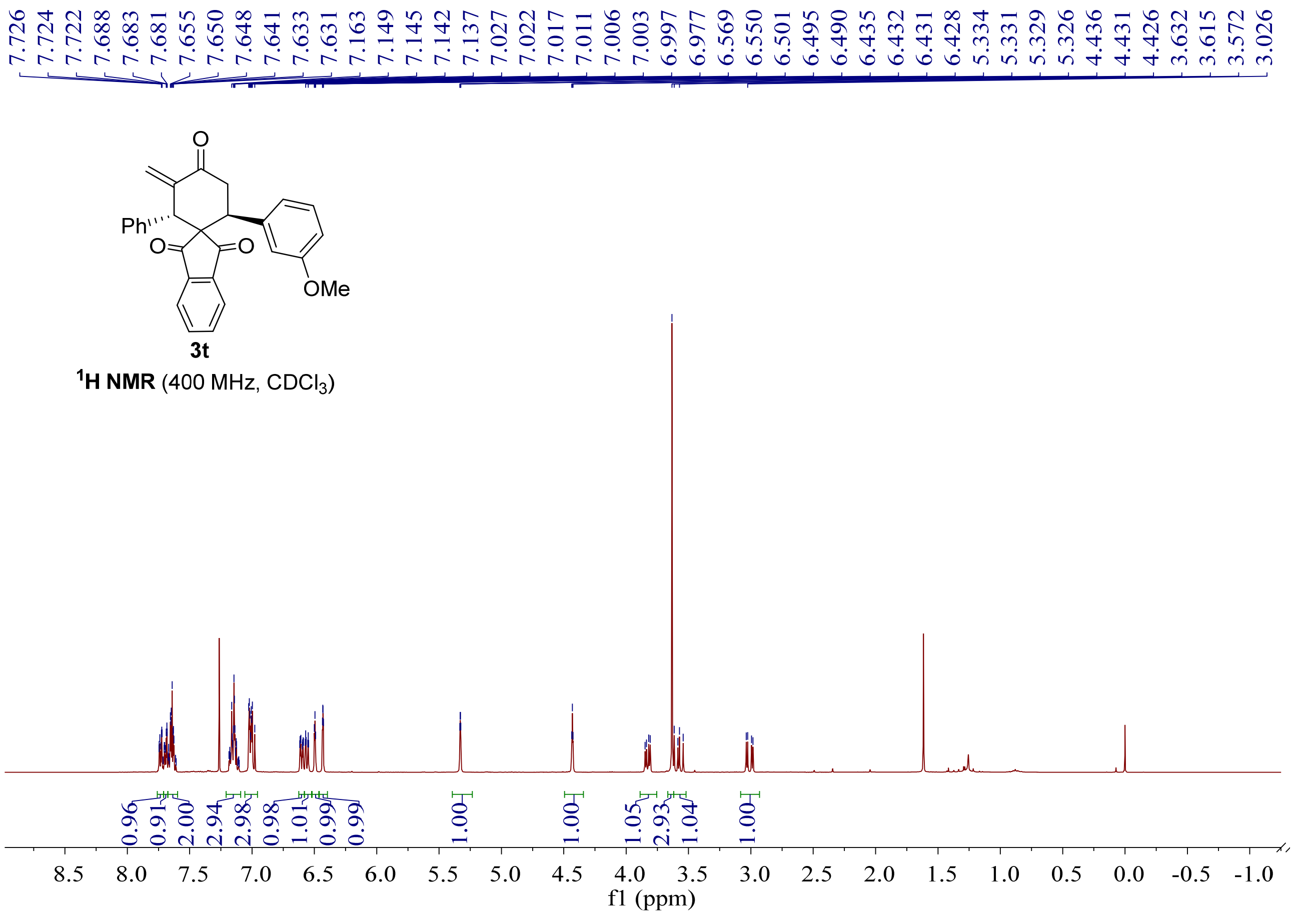
7.726 7.724 7.722 7.688 7.683 7.681 7.655 7.650 7.648 7.641 7.633 7.631 7.163 7.149 7.145 7.142 7.137 7.027 7.022 7.017 7.011 7.006 7.003 6.997 6.977 6.569 6.550 6.501 6.495 6.490 6.435 6.432 6.431 6.428 5.334 5.331 5.329 5.326 4.436 4.431 4.426 3.632 3.615 3.572 3.026
O
Ph
O
O
OMe
3t
1H NMR (400 MHz, CDCl3)
0.96 0.91 2.00 2.94 2.98 0.98 1.01 0.99 0.99 1.00 1.00 1.05 2.93 1.04 1.00
8.5 8.0 7.5 7.0 6.5 6.0 5.5 5.0 4.5 4.0 3.5 3.0 2.5 2.0 1.5 1.0 0.5 0.0 -0.5 -1.0
f1 (ppm)

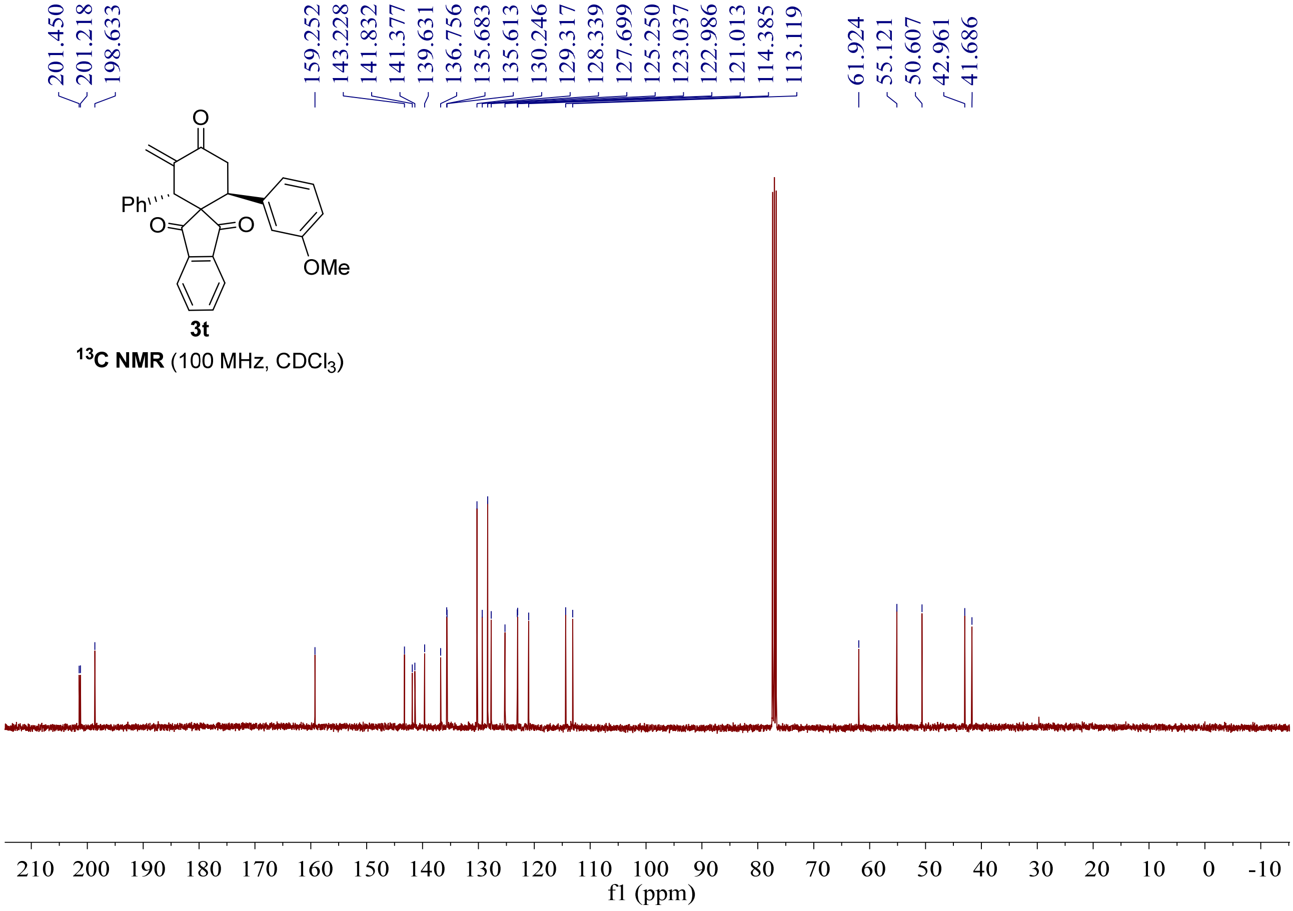
201.450 201.218 198.633 159.252 143.228 141.832 141.377 139.631 136.756 135.683 135.613 130.246 129.317 128.339 127.699 125.250 123.037 122.986 121.013 114.385 113.119 61.924 55.121 50.607 42.961 41.686
O
Ph
O
O
OMe
3t
13C NMR (100 MHz, CDCl3)
210 200 190 180 170 160 150 140 130 120 110 100 90 80 70 60 50 40 30 20 10 0 -10
f1 (ppm)

Daicel Chiral IA Column (*i*PrOH/*n*-hexane = 10/90, 1.0 mL/min)

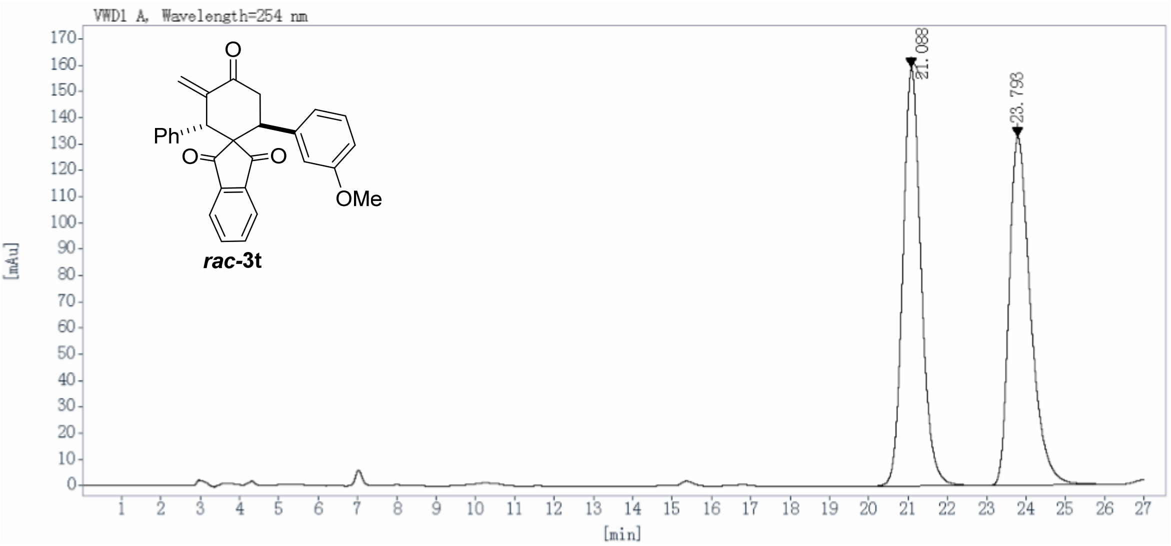

Ret Time [min]	Peak Type	Width [min]	Height [mAU]	Area [mAU*s]	Area [%]
21.088	BB	0.49	159.3876	5134.3203	50.4081
23.793	BB	0.58	132.7708	5051.1821	49.5919
			Totals:	10185.5024	100.0000

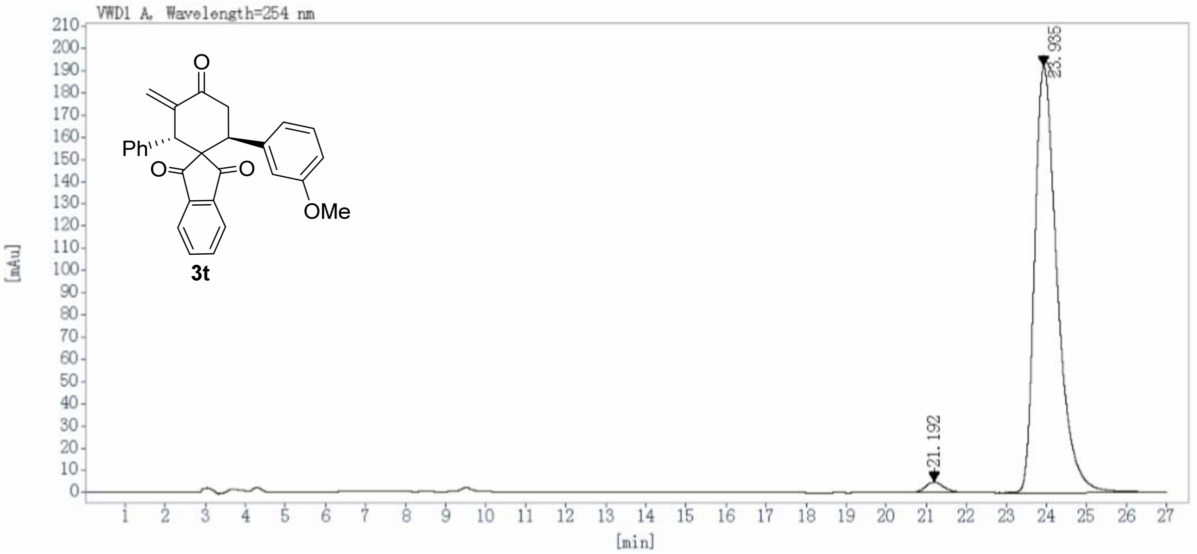

Ret Time [min]	Peak Type	Width [min]	Height [mAU]	Area [mAU*s]	Area [%]
21.192	BB	0.50	4.8386	158.2449	2.0632
23.935	BBA	0.60	192.3088	7511.7407	97.9368
			Totals:	7669.9856	100.0000

HRMS (ESI-TOF) m/z: [M + Na]$^+$ Calcd for $C_{28}H_{22}O_4Na^+$ 445.1410; Found 445.1419.

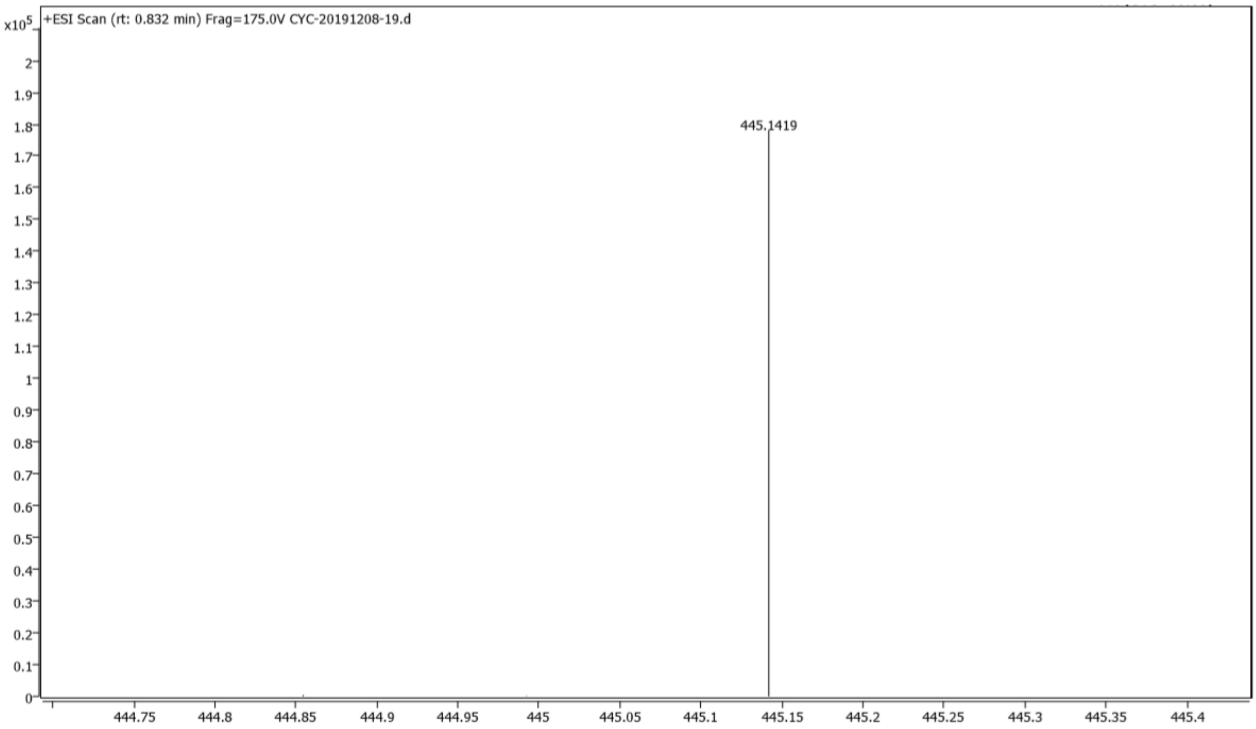

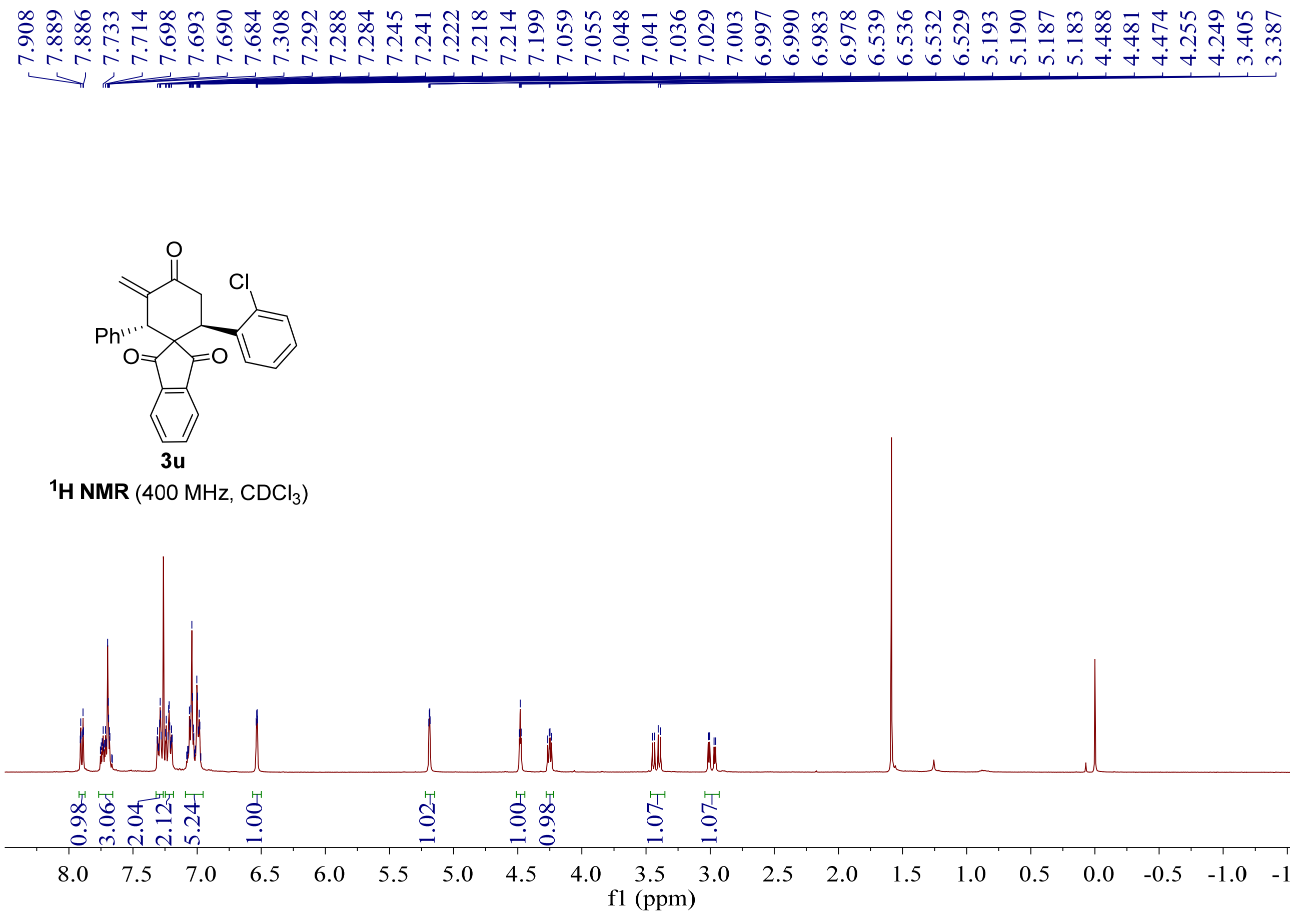
7.908
7.889
7.886
7.733
7.714
7.698
7.693
7.690
7.684
7.308
7.292
7.288
7.284
7.245
7.241
7.222
7.218
7.214
7.199
7.059
7.055
7.048
7.041
7.036
7.029
7.003
6.997
6.990
6.983
6.978
6.539
6.536
6.532
6.529
5.193
5.190
5.187
5.183
4.488
4.481
4.474
4.255
4.249
3.405
3.387
O
Cl
Ph
O
O
3u
^{1}H NMR (400 MHz, $CDCl_3$)
0.98
3.06
2.04
2.12
5.24
1.00
1.02
1.00
0.98
1.07
1.07
8.0
7.5
7.0
6.5
6.0
5.5
5.0
4.5
4.0
3.5
3.0
2.5
2.0
1.5
1.0
0.5
0.0
-0.5
-1.0
-1
f1 (ppm)

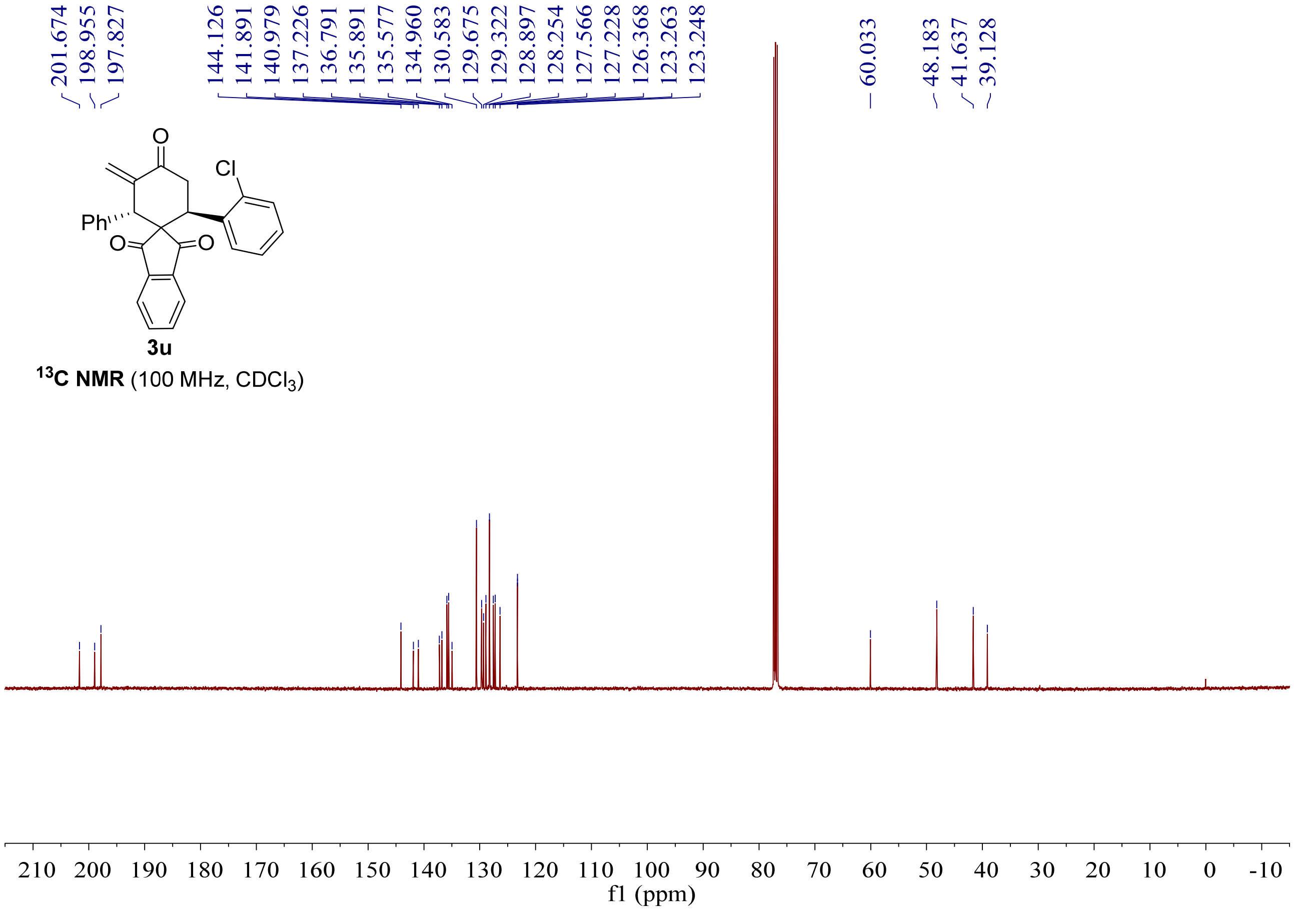
201.674
198.955
197.827
144.126
141.891
140.979
137.226
136.791
135.891
135.577
134.960
130.583
129.675
129.322
128.897
128.254
127.566
127.228
126.368
123.263
123.248
60.033
48.183
41.637
39.128
O
Cl
Ph
O
O
3u
^{13}C NMR (100 MHz, $CDCl_3$)
210
200
190
180
170
160
150
140
130
120
110
100
90
80
70
60
50
40
30
20
10
0
-10
f1 (ppm)

Daicel Chiral AD-H Column (*i*PrOH/*n*-hexane = 10/90, 1.0 mL/min)

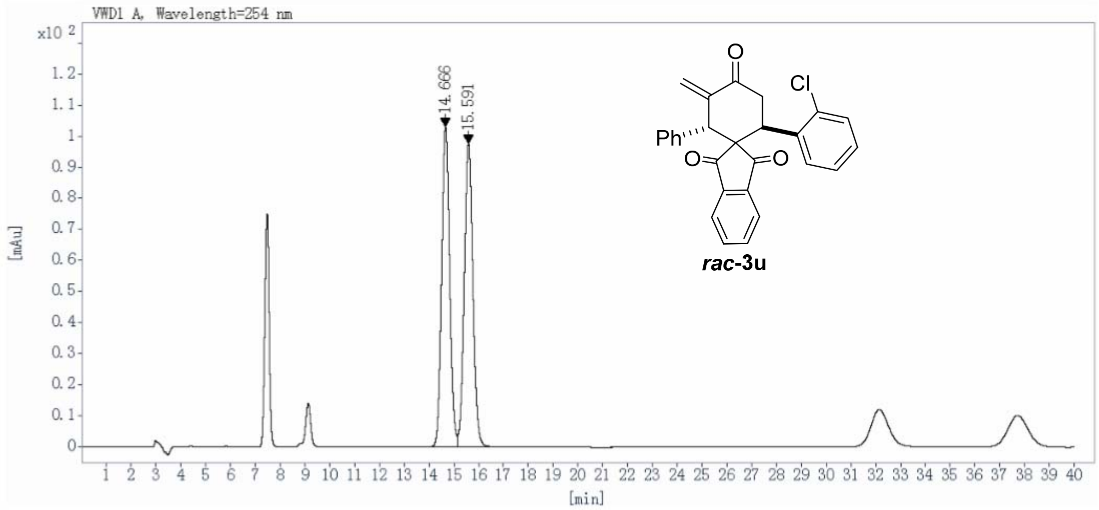

Ret Time [min]	Peak Type	Width [min]	Height [mAU]	Area [mAU*s]	Area [%]
14.666	BV	0.35	102.9334	2316.7617	49.5042
15.591	VB	0.38	97.7268	2363.1726	50.4958
			Totals:	4679.9343	100.0000

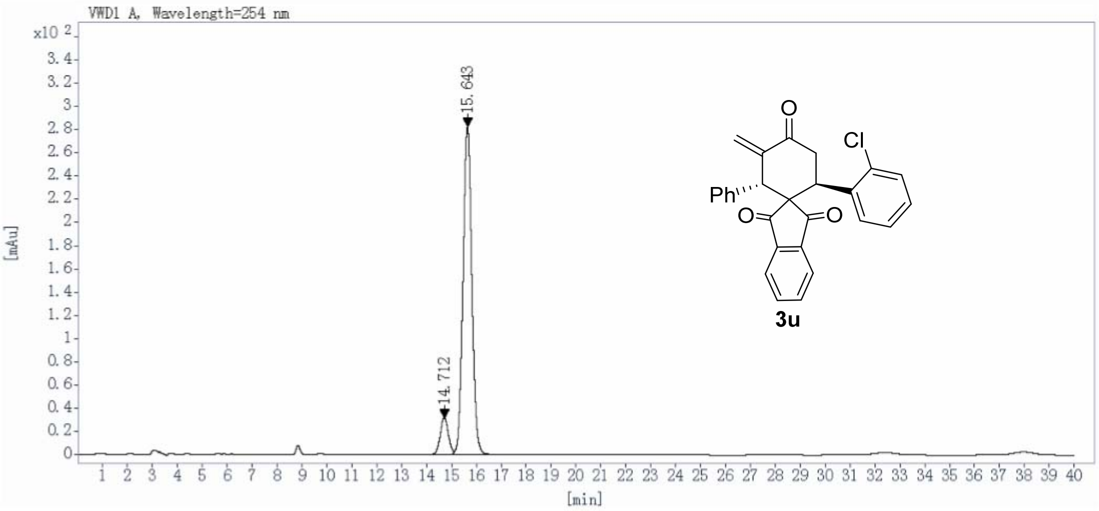

Ret Time [min]	Peak Type	Width [min]	Height [mAU]	Area [mAU*s]	Area [%]
14.712	BV E	0.36	31.7191	735.0936	9.5061
15.643	VB R	0.38	282.1132	6997.7334	90.4939
			Totals:	7732.8270	100.0000

HRMS (ESI-TOF) m/z: [M + Na]$^+$ Calcd for $C_{27}H_{19}O_3ClNa^+$ 449.0915 (^{35}Cl) and 451.0885 (^{37}Cl); Found 449.0916 (^{35}Cl) and 451.0895 (^{37}Cl).

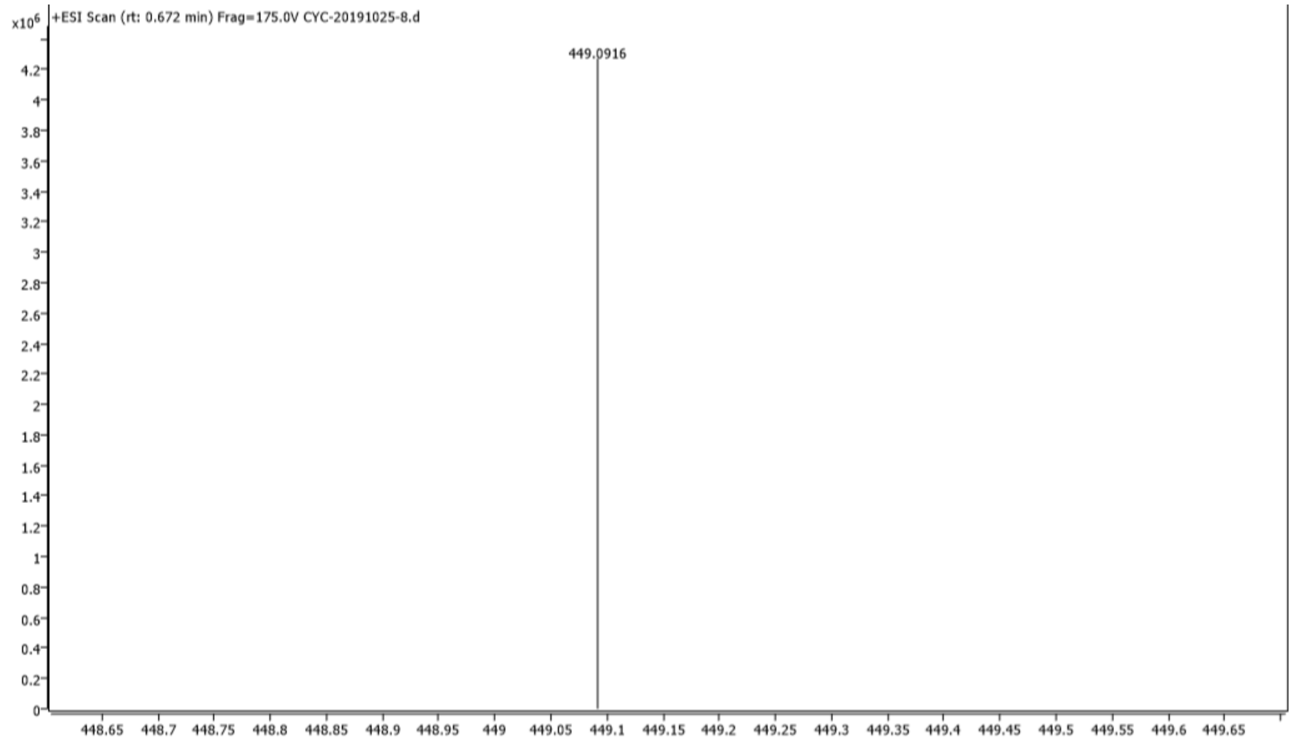

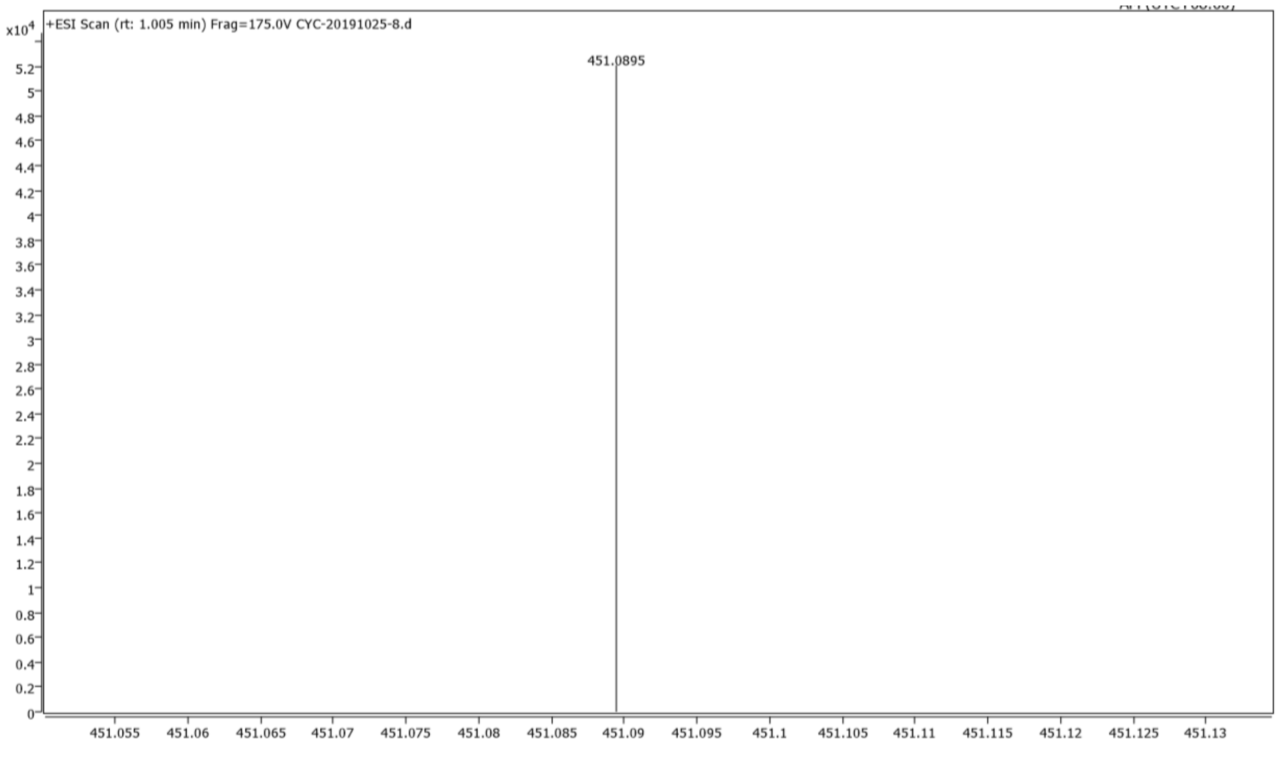

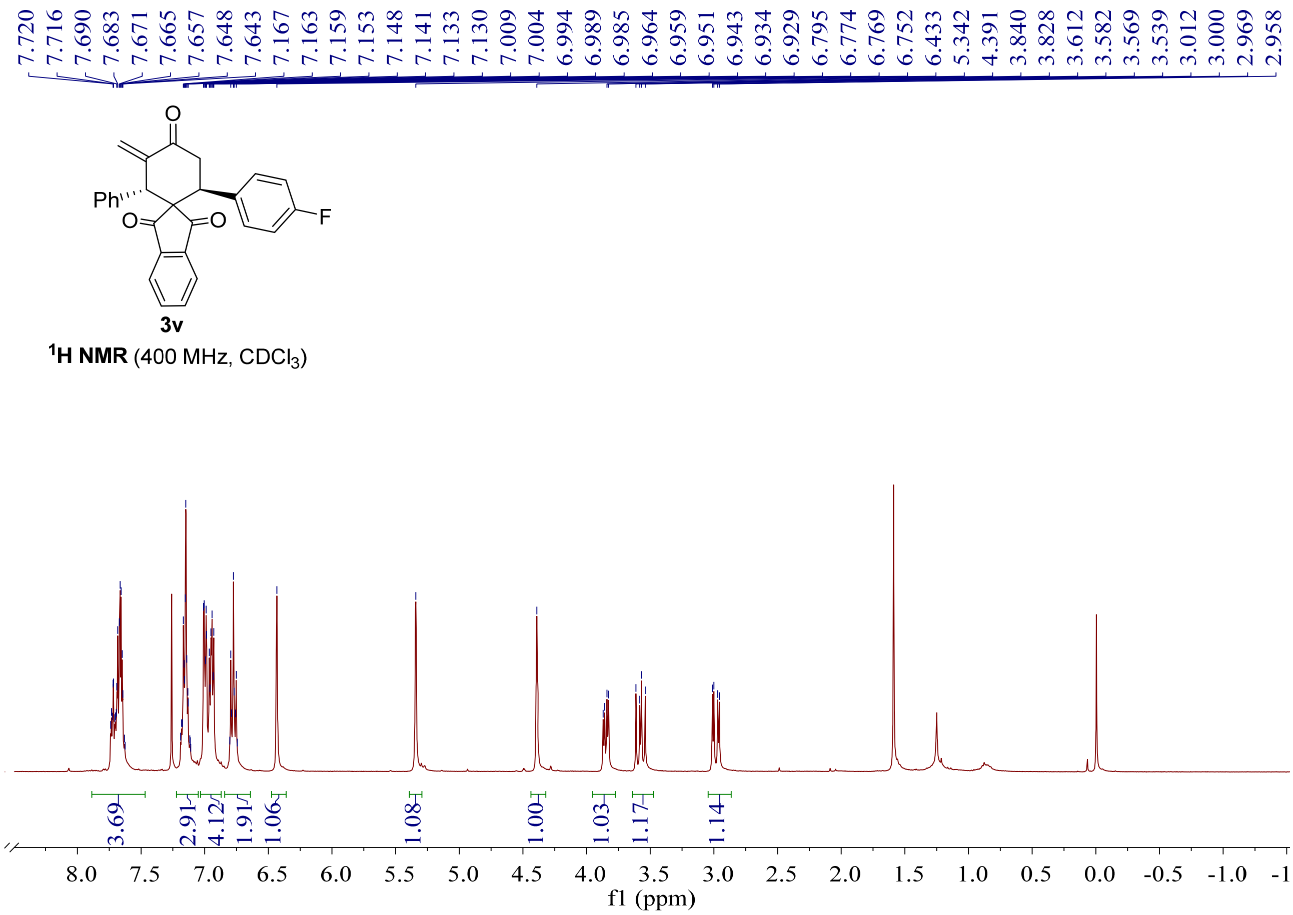

3v

^{1}H NMR (400 MHz, $CDCl_3$)

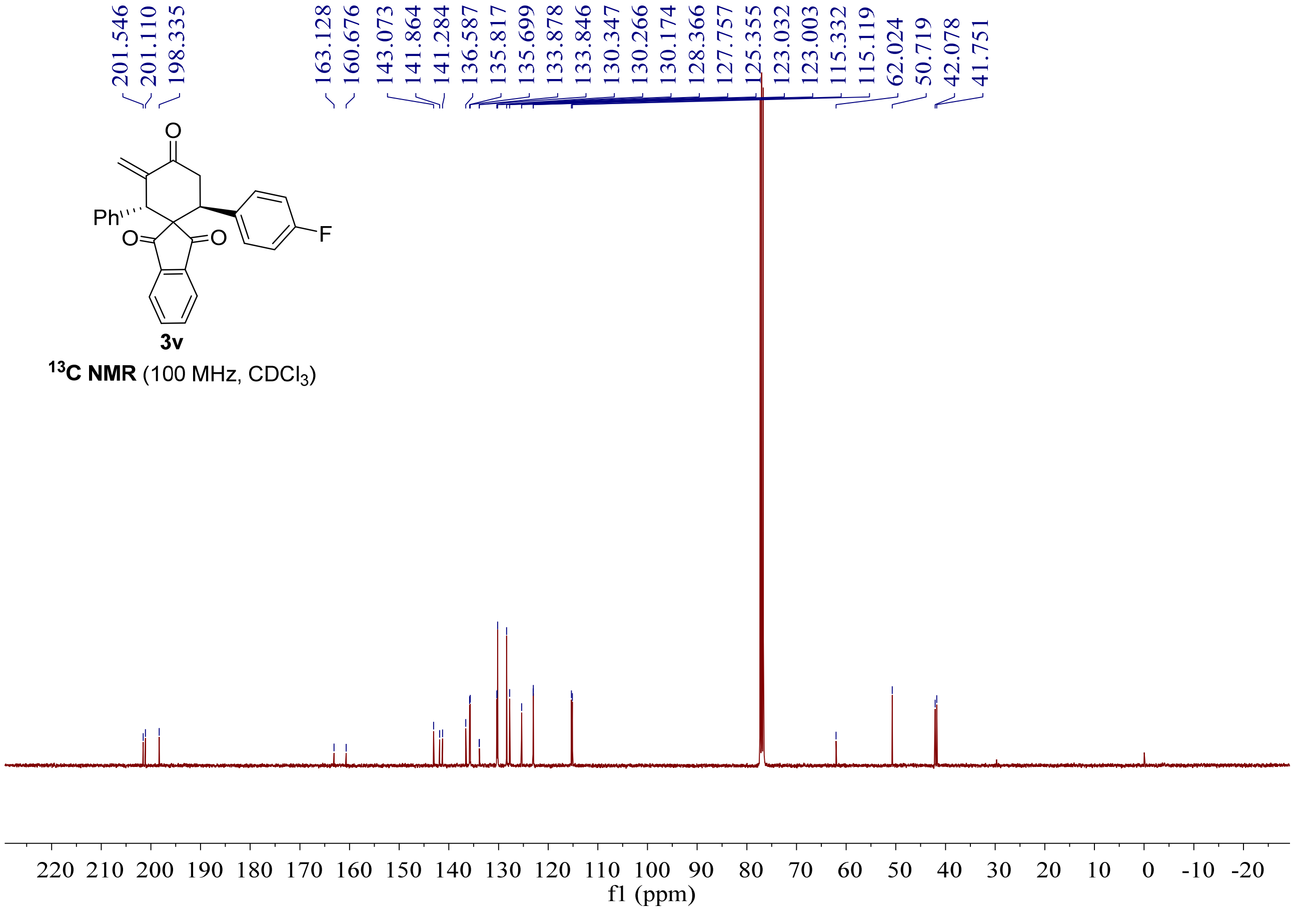

3v

^{13}C NMR (100 MHz, $CDCl_3$)

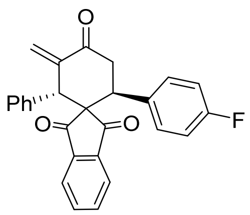

3v

^{19}F NMR (376 MHz, $CDCl_3$)

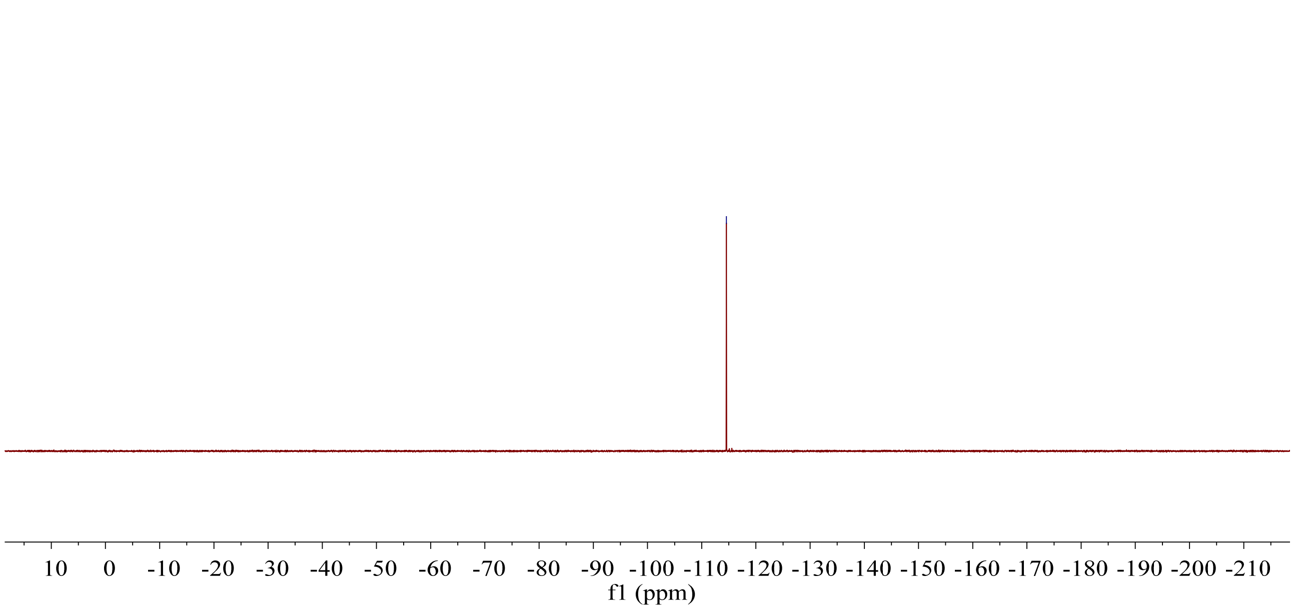

Daicel Chiral IA Column (*i*PrOH/*n*-hexane = 40/60, 0.8 mL/min)

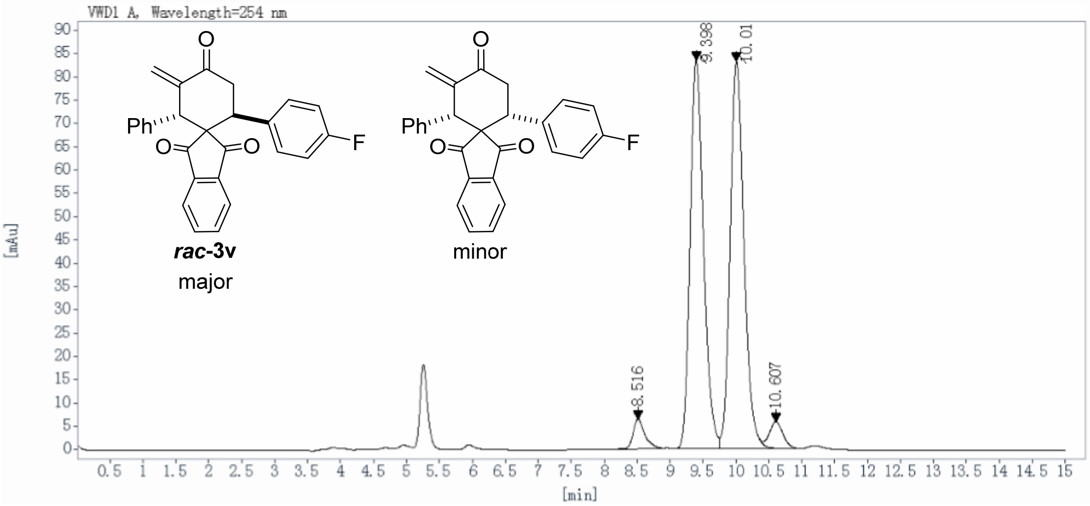

Ret Time [min]	Peak Type	Width [min]	Height [mAU]	Area [mAU*s]	Area [%]
8.516	BB	0.19	6.3749	78.9041	3.1030
9.398	BV	0.22	83.3714	1173.4047	46.1454
10.010	VV R	0.22	83.0753	1208.2971	47.5176
10.607	VB E	0.22	5.7075	82.2358	3.2340
			Totals:	2542.8417	100.0000

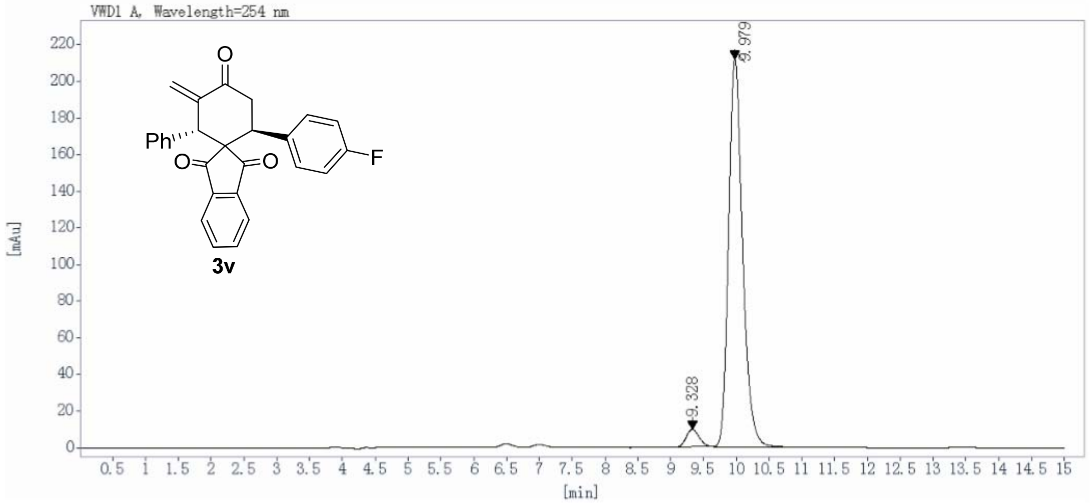

Ret Time [min]	Peak Type	Width [min]	Height [mAU]	Area [mAU*s]	Area [%]
9.328	MM	0.23	9.7537	135.9941	4.1133
9.979	VB	0.23	211.9662	3170.2351	95.8867
			Totals:	3306.2292	100.0000

HRMS (ESI-TOF) m/z: $[M + Na]^+$ Calcd for $C_{27}H_{19}O_3FNa^+$ 433.1210; Found 433.1237.

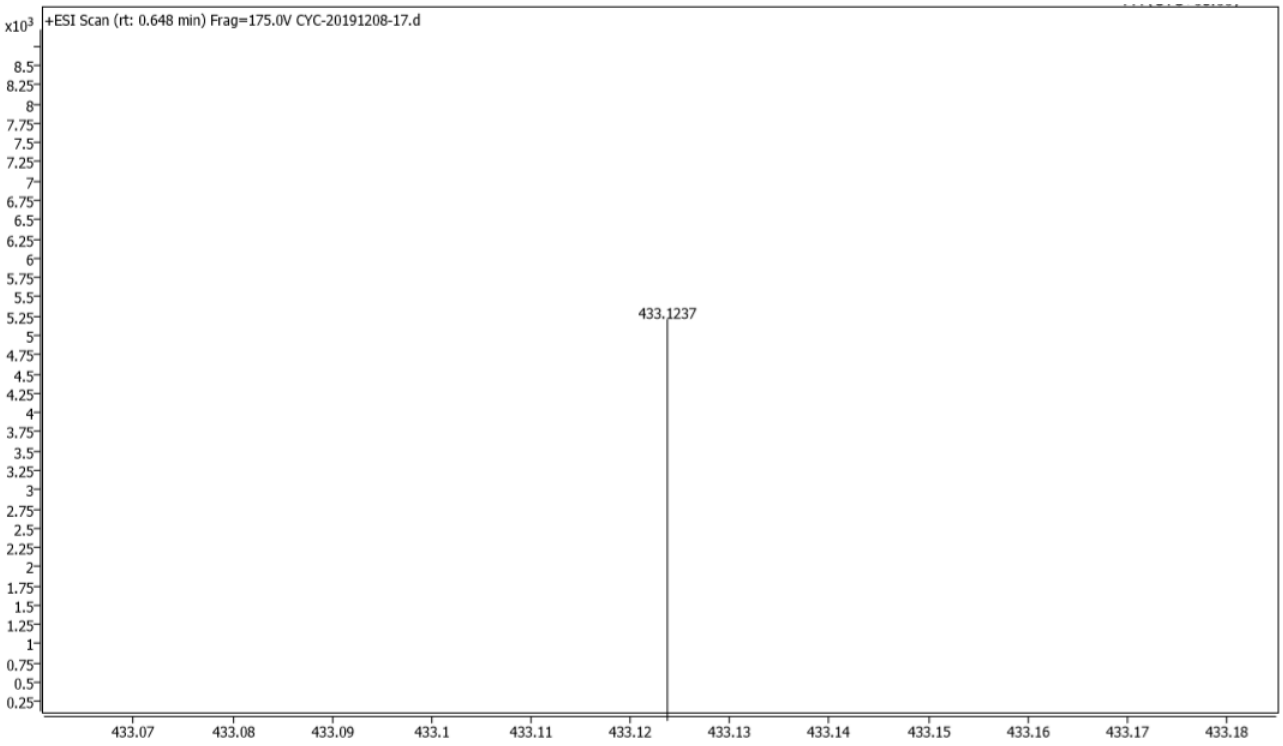

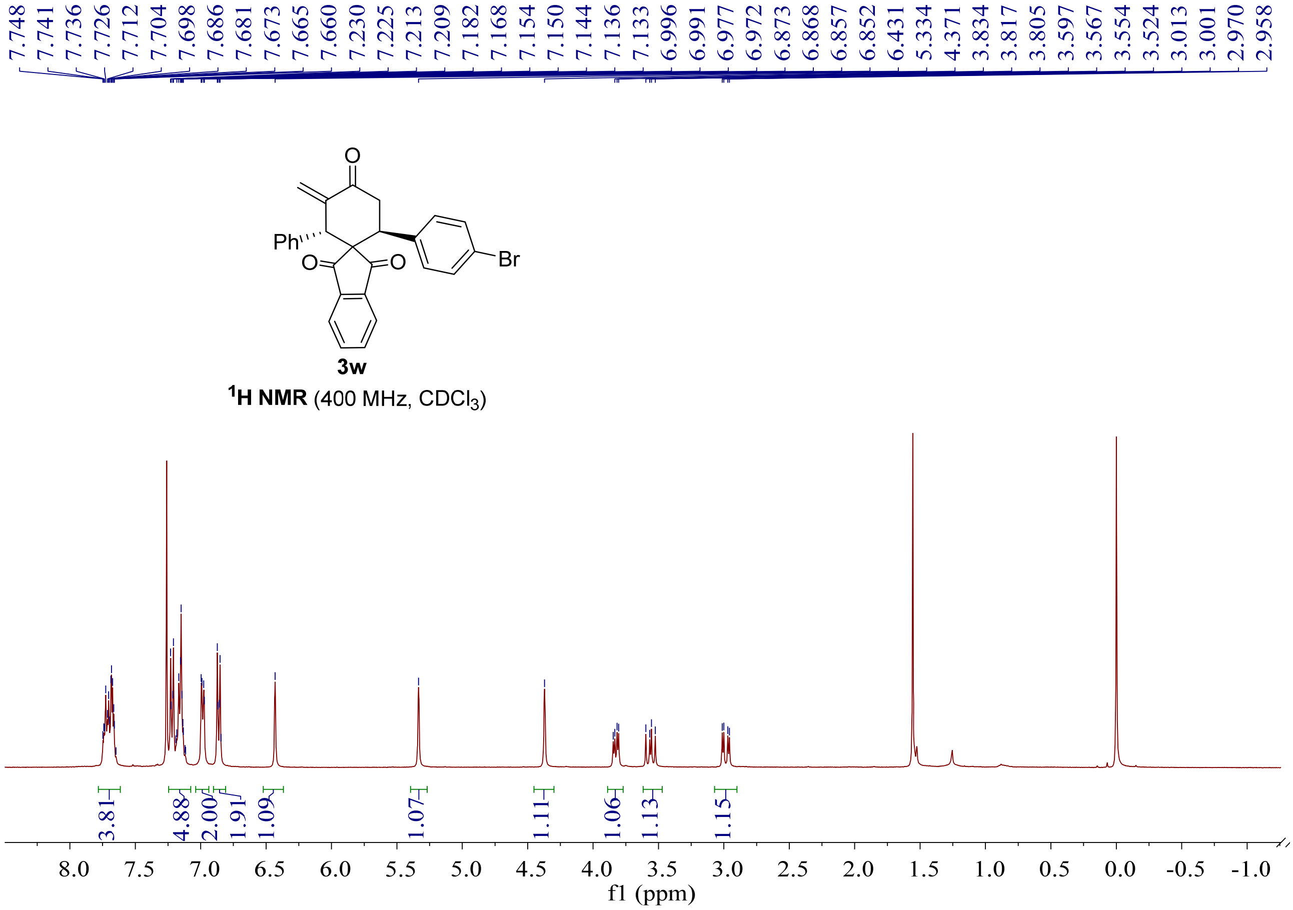
7.748 7.741 7.736 7.726 7.712 7.704 7.698 7.686 7.681 7.673 7.665 7.660 7.230 7.225 7.213 7.209 7.182 7.168 7.154 7.150 7.144 7.136 7.133 6.996 6.991 6.977 6.972 6.873 6.868 6.857 6.852 6.431 5.334 4.371 3.834 3.817 3.805 3.597 3.567 3.554 3.524 3.013 3.001 2.970 2.958
Ph
O
Br
3w
¹H NMR (400 MHz, CDCl3)
3.81 4.88 2.00 1.91 1.09 1.07 1.11 1.06 1.13 1.15
8.0 7.5 7.0 6.5 6.0 5.5 5.0 4.5 4.0 3.5 3.0 2.5 2.0 1.5 1.0 0.5 0.0 -0.5 -1.0
f1 (ppm)

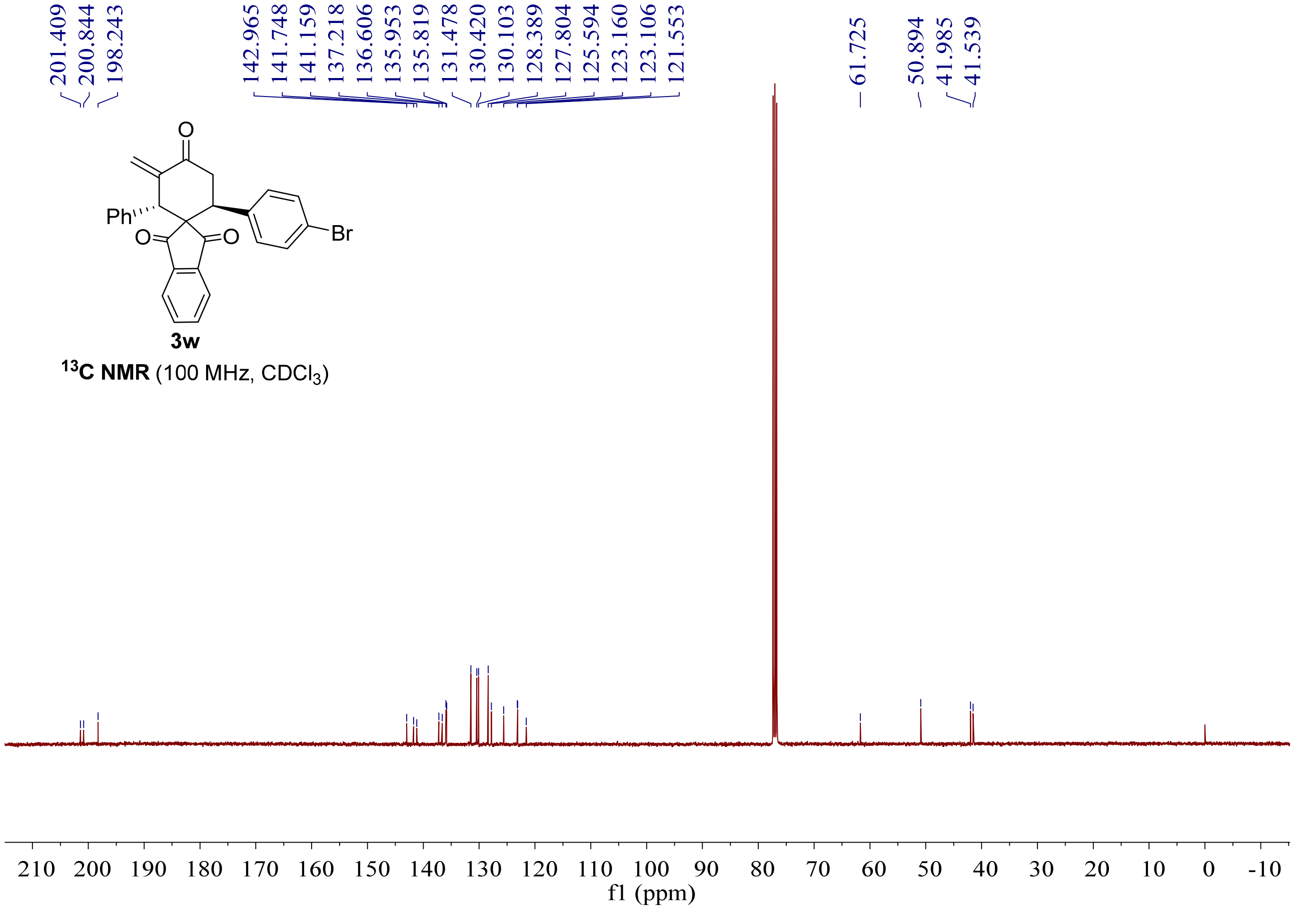
201.409 200.844 198.243 142.965 141.748 141.159 137.218 136.606 135.953 135.819 131.478 130.420 130.103 128.389 127.804 125.594 123.160 123.106 121.553 61.725 50.894 41.985 41.539
Ph
O
Br
3w
¹³C NMR (100 MHz, CDCl3)
210 200 190 180 170 160 150 140 130 120 110 100 90 80 70 60 50 40 30 20 10 0 -10
f1 (ppm)

Daicel Chiral IA Column (*i*PrOH/*n*-hexane = 20/80, 1.0 mL/min)

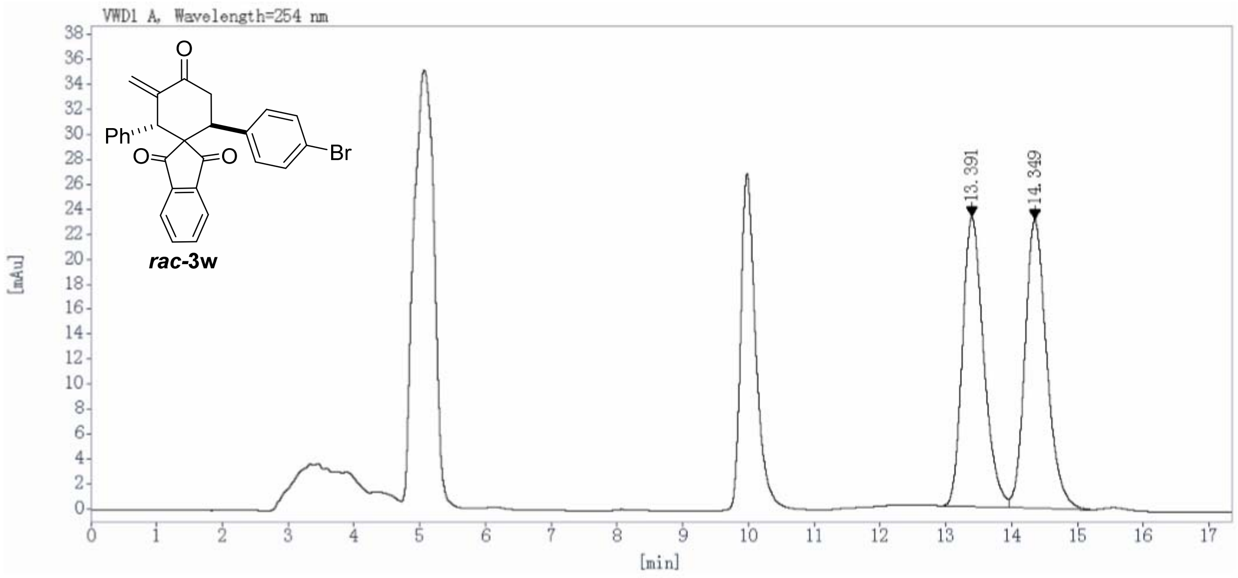

Ret Time [min]	Peak Type	Width [min]	Height [mAU]	Area [mAU*s]	Area [%]
13.391	BV	0.34	23.1678	519.0718	49.4120
14.349	VB	0.35	23.0858	531.4262	50.5880
			Totals:	1050.4980	100.0000

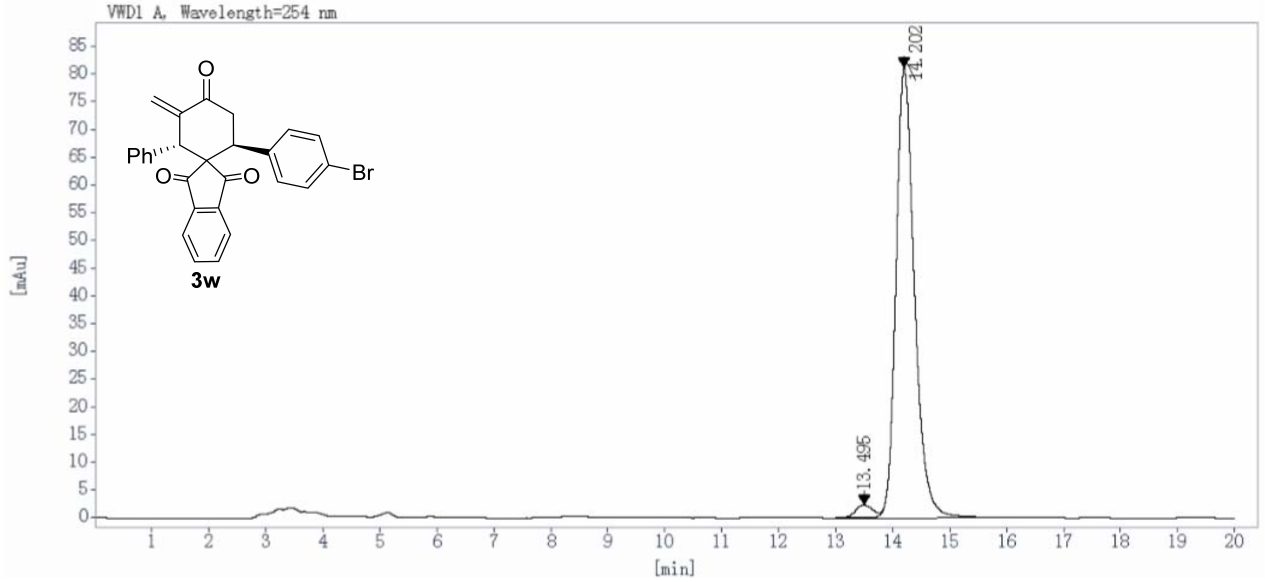

Ret Time [min]	Peak Type	Width [min]	Height [mAU]	Area [mAU*s]	Area [%]
13.495	BV E	0.32	2.3451	48.8492	2.5951
14.202	VB R	0.34	81.2030	1833.4836	97.4049
			Totals:	1882.3328	100.0000

HRMS (ESI-TOF) m/z: [M + K]$^+$ Calcd for $C_{27}H_{19}O_3BrK^+$ 509.1049 (^{79}Br) and 511.0129 (^{81}Br); Found 509.1048 (^{79}Br) and 511.0138 (^{81}Br).

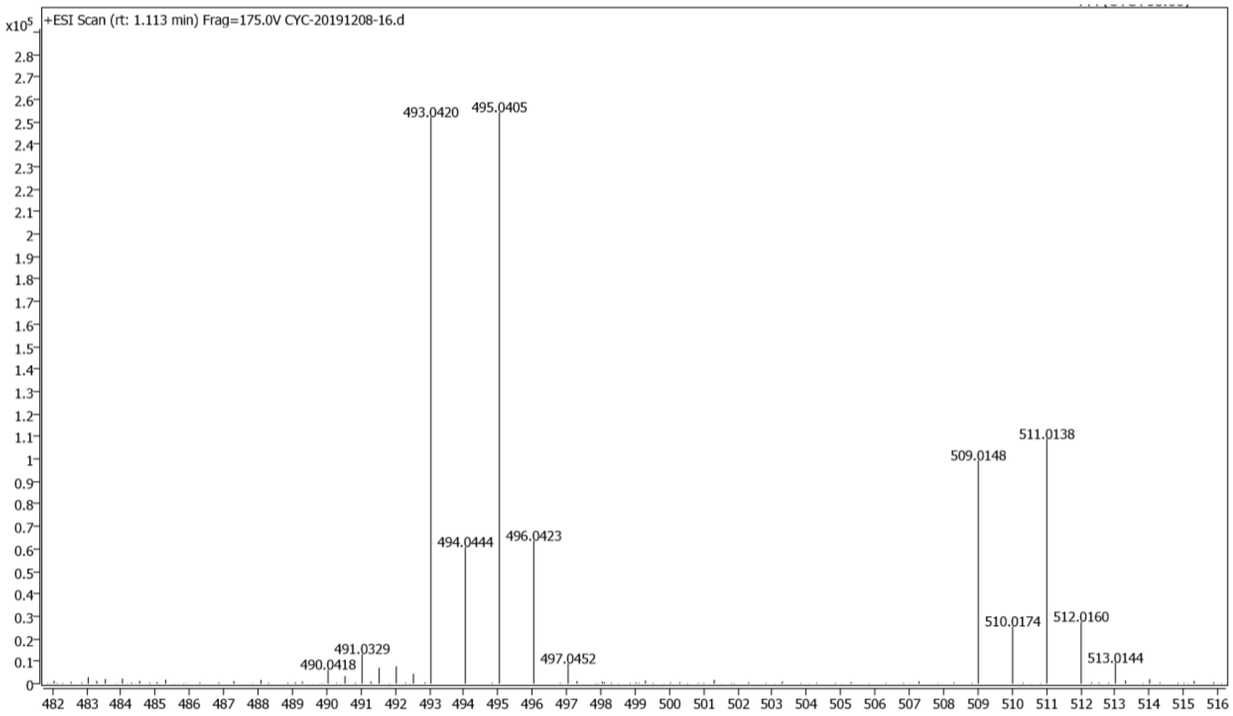

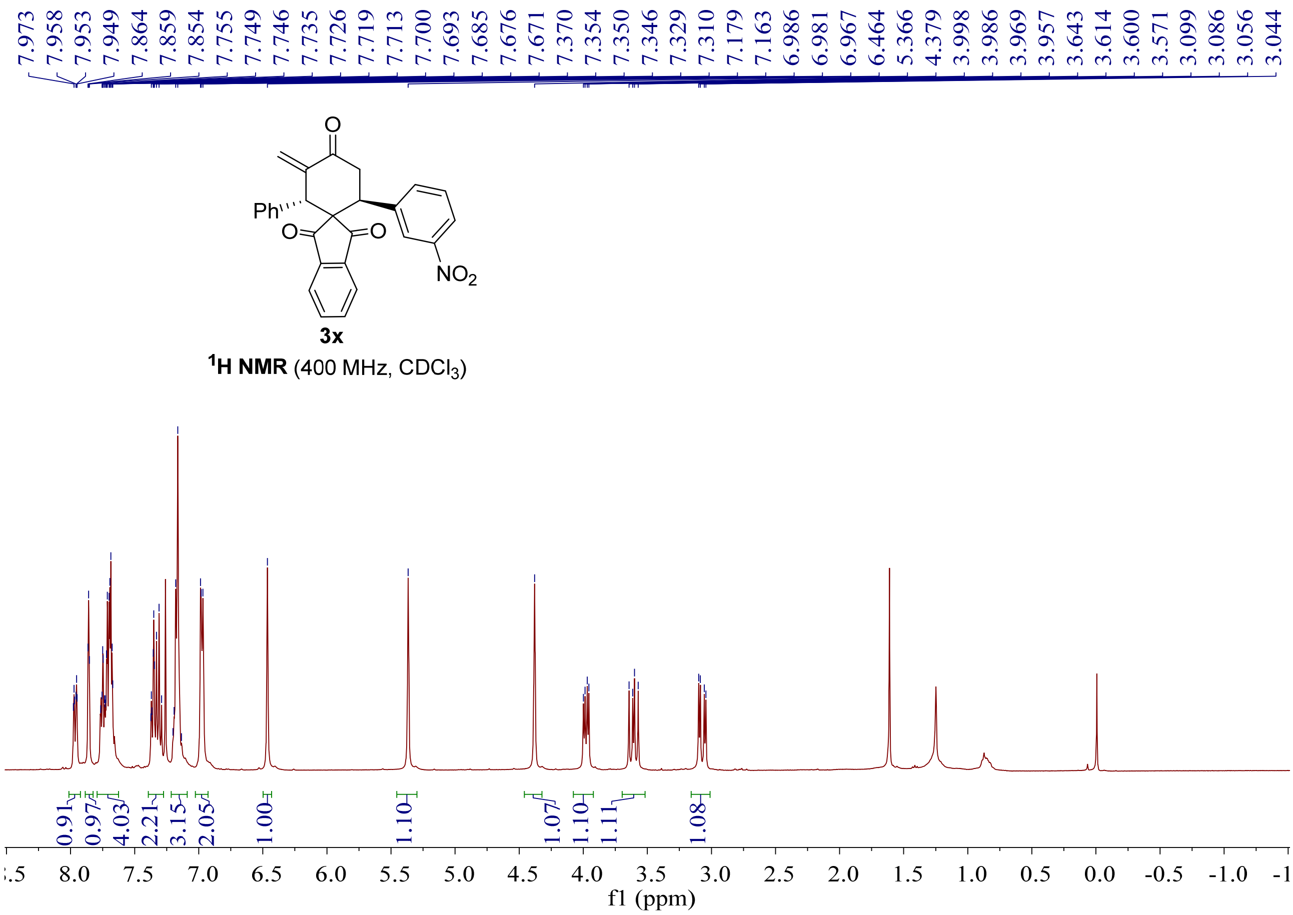
7.973 7.958 7.953 7.949 7.864 7.859 7.854 7.755 7.749 7.746 7.735 7.726 7.719 7.713 7.700 7.693 7.685 7.676 7.671 7.370 7.354 7.350 7.346 7.329 7.310 7.179 7.163 6.986 6.981 6.967 6.464 5.366 4.379 3.998 3.986 3.969 3.957 3.643 3.614 3.600 3.571 3.099 3.086 3.056 3.044
O
Ph
O
O
NO2
3x
1H NMR (400 MHz, CDCl3)
0.91 0.97 4.03 2.21 3.15 2.05 1.00 1.10 1.07 1.10 1.11 1.08
f1 (ppm)

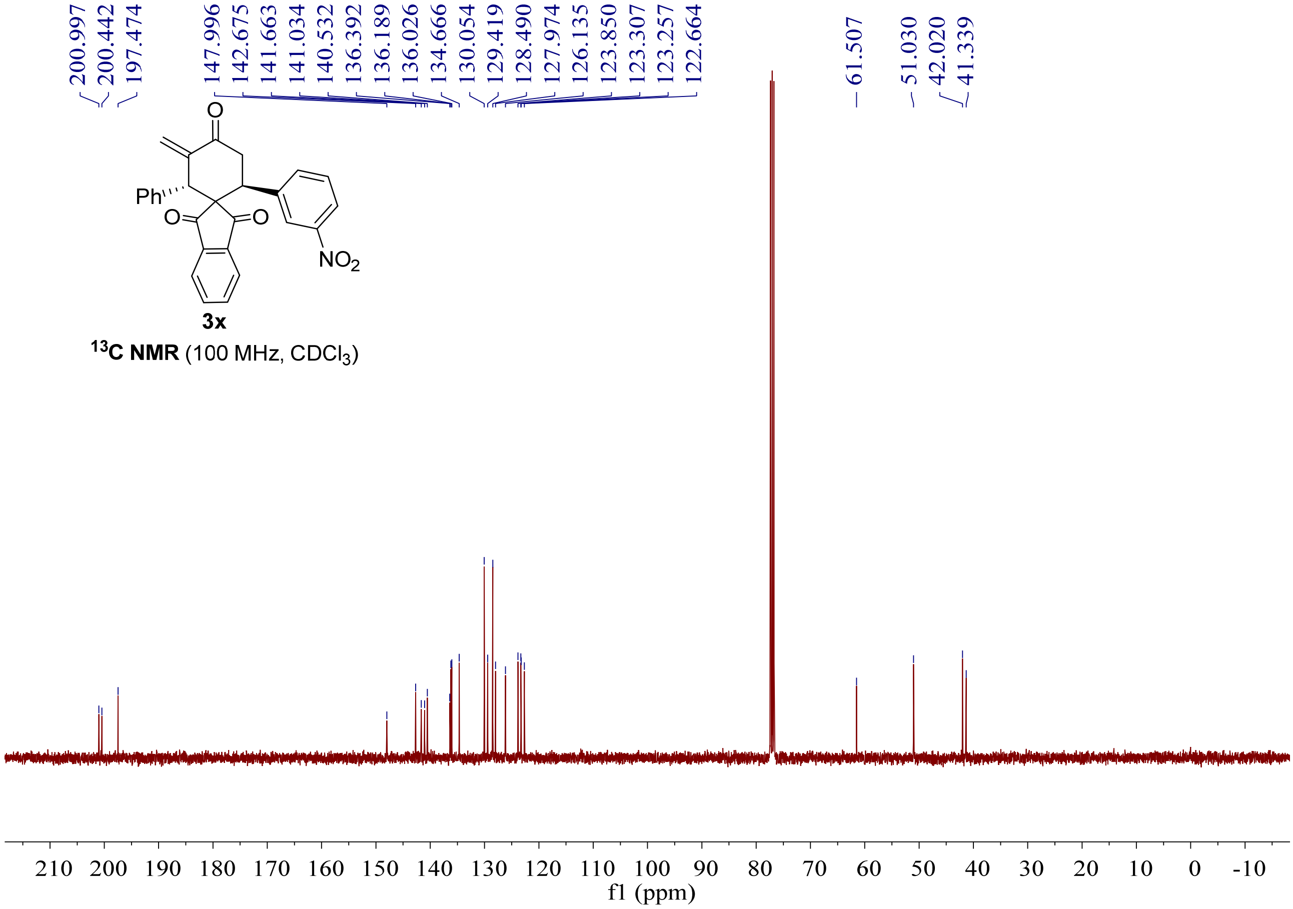
200.997 200.442 197.474 147.996 142.675 141.663 141.034 140.532 136.392 136.189 136.026 134.666 130.054 129.419 128.490 127.974 126.135 123.850 123.307 123.257 122.664 61.507 51.030 42.020 41.339
O
Ph
O
O
NO2
3x
13C NMR (100 MHz, CDCl3)
f1 (ppm)

Daicel Chiral IC Column (*i*PrOH/*n*-hexane = 40/60, 1.0 mL/min)

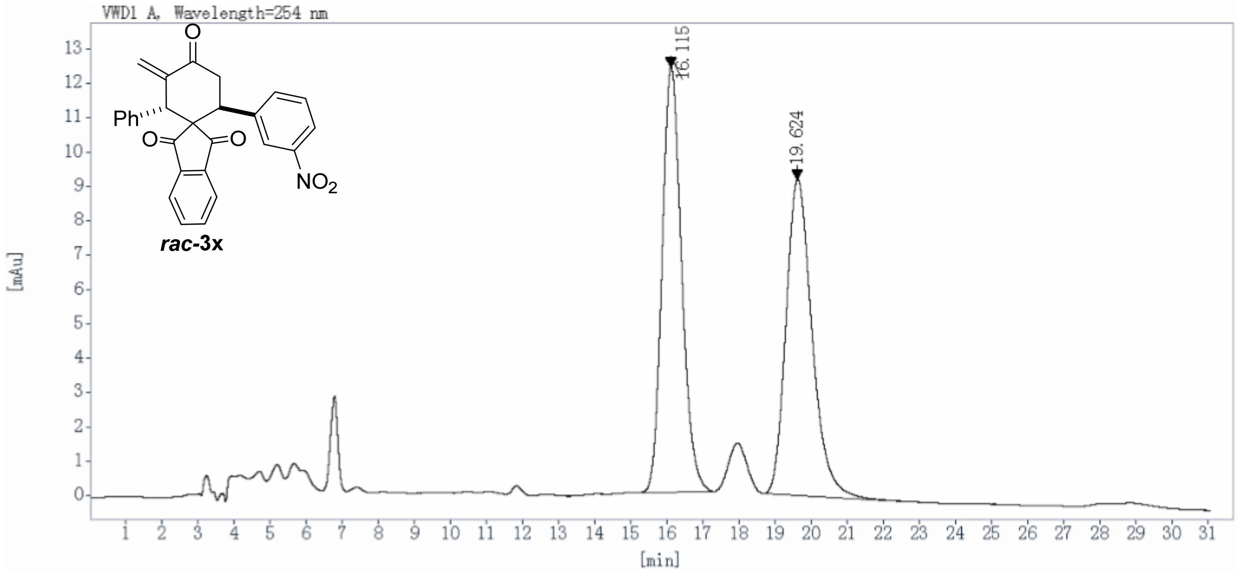

Ret Time [min]	Peak Type	Width [min]	Height [mAU]	Area [mAU*s]	Area [%]
16.115	BB	0.58	12.3632	464.6705	50.0420
19.624	BB	0.76	9.2111	463.8903	49.9580
			Totals:	928.5607	100.0000

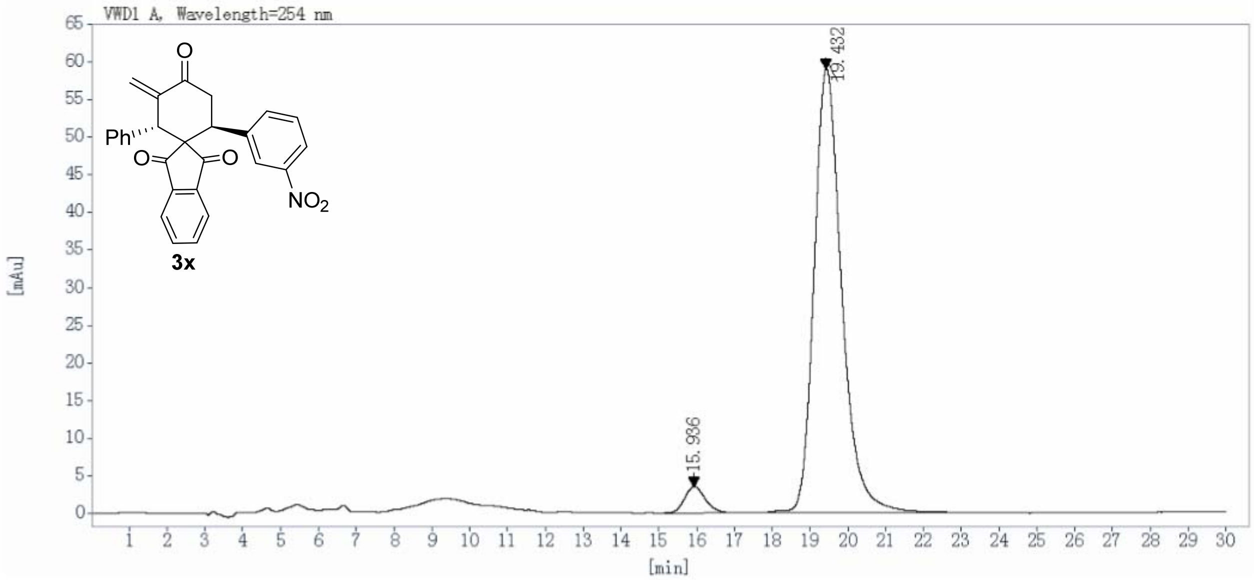

Ret Time [min]	Peak Type	Width [min]	Height [mAU]	Area [mAU*s]	Area [%]
15.936	BB	0.58	3.4850	132.3162	4.2331
19.432	BB	0.78	58.9245	2993.4478	95.7669
			Totals:	3125.7640	100.0000

HRMS (ESI-TOF) m/z: $[M + Na]^+$ Calcd for $C_{27}H_{19}NO_5Na^+$ 460.1155 ; Found 460.1153.

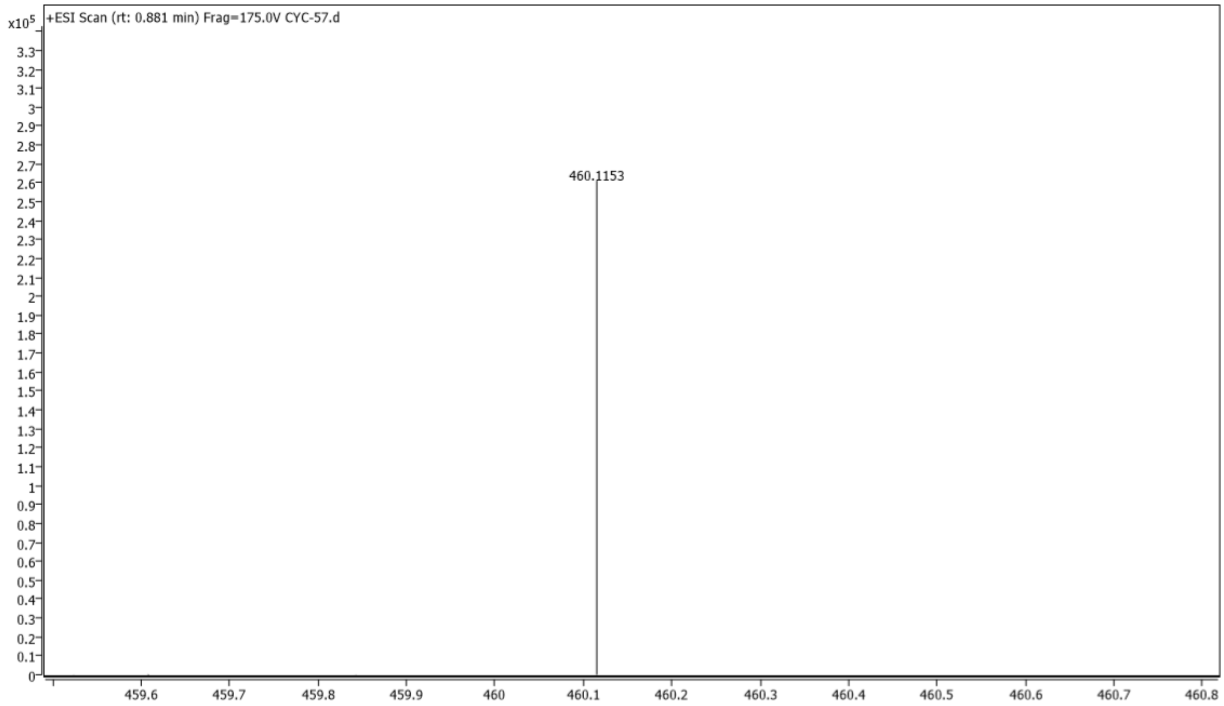

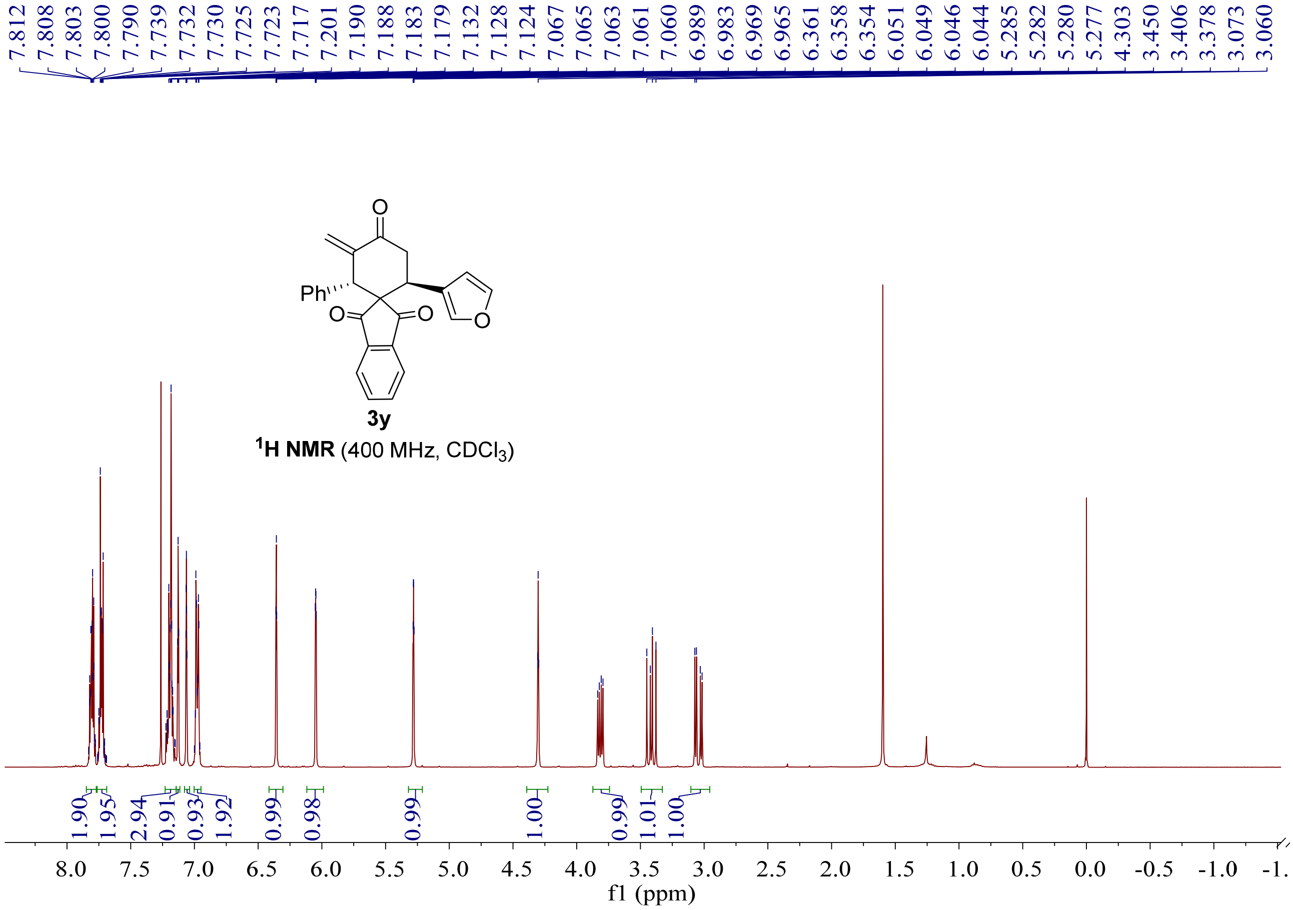
7.812 7.808 7.803 7.800 7.790 7.739 7.732 7.730 7.725 7.723 7.717 7.201 7.190 7.188 7.183 7.179 7.132 7.128 7.124 7.067 7.065 7.063 7.061 7.060 6.989 6.983 6.969 6.965 6.361 6.358 6.354 6.051 6.049 6.046 6.044 5.285 5.282 5.280 5.277 4.303 3.450 3.406 3.378 3.073 3.060
3y
^{1}H NMR (400 MHz, CDCl$_3$)
1.90 1.95 2.94 0.91 0.93 1.92 0.99 0.98 0.99 1.00 0.99 1.01 1.00
f1 (ppm)

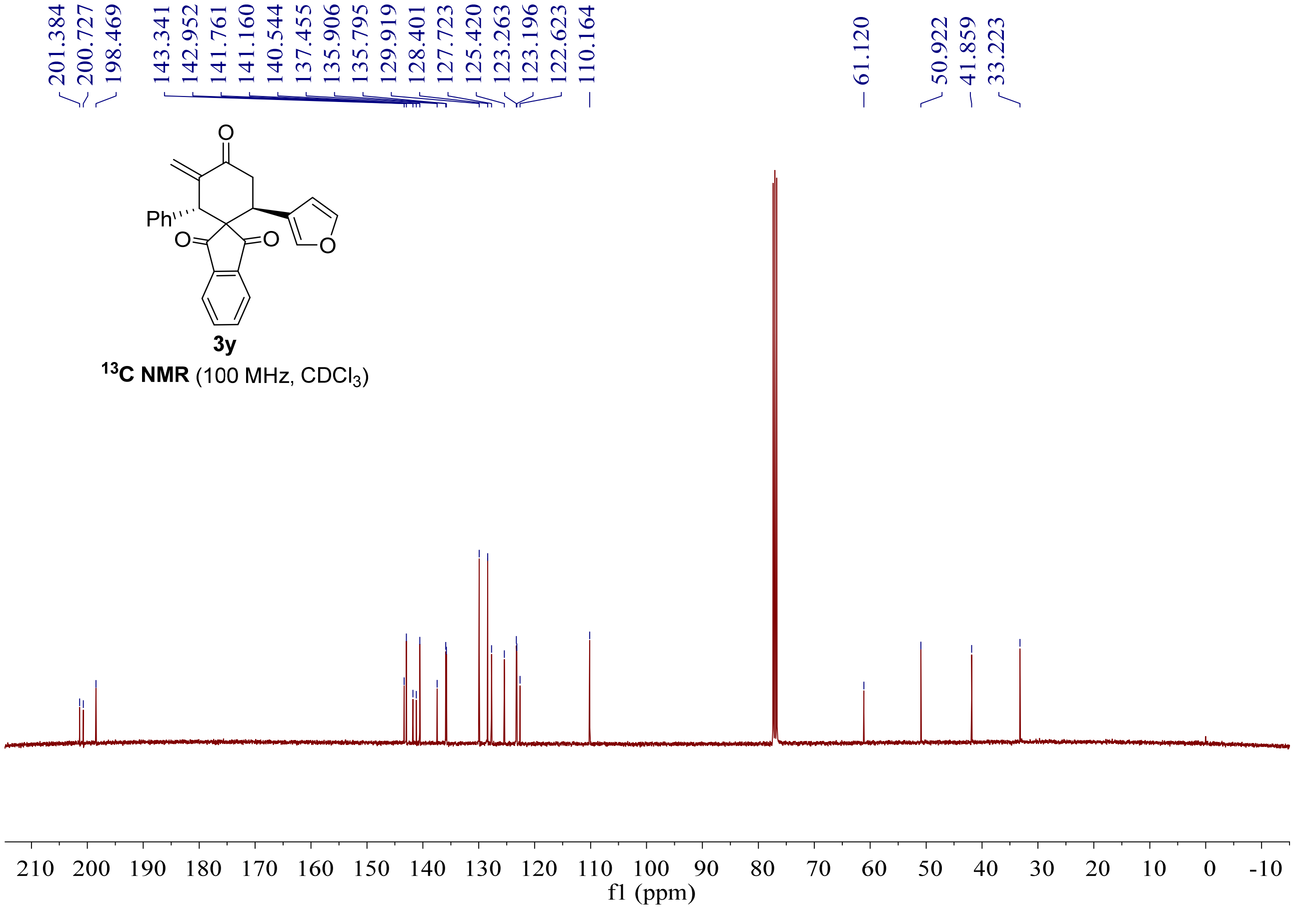
201.384 200.727 198.469 143.341 142.952 141.761 141.160 140.544 137.455 135.906 135.795 129.919 128.401 127.723 125.420 123.263 123.196 122.623 110.164 61.120 50.922 41.859 33.223
3y
^{13}C NMR (100 MHz, CDCl$_3$)
f1 (ppm)

Daicel Chiral AD-H Column (*i*PrOH/*n*-hexane = 20/80, 1.0 mL/min)

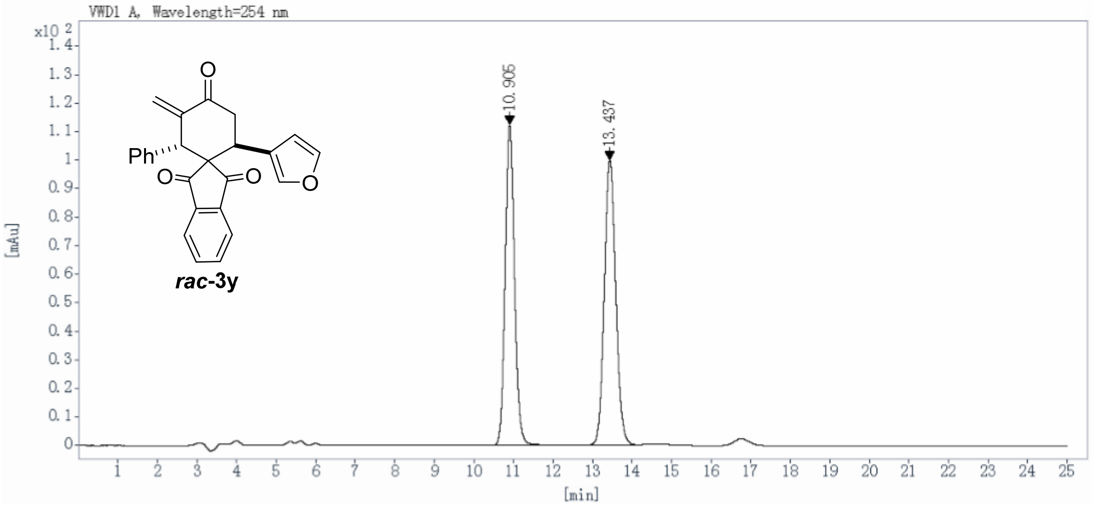

Ret Time [min]	Peak Type	Width [min]	Height [mAU]	Area [mAU*s]	Area [%]
10.905	BB	0.25	112.3217	1832.5845	47.8299
13.437	BB	0.31	99.9150	1998.8755	52.1701
			Totals:	3831.4600	100.0000

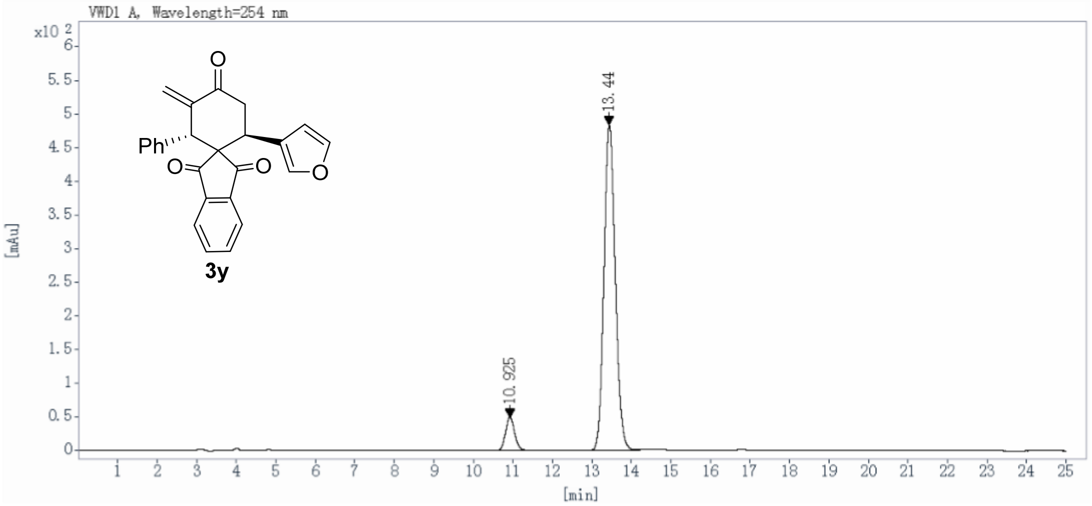

Ret Time [min]	Peak Type	Width [min]	Height [mAU]	Area [mAU*s]	Area [%]
10.925	BB	0.25	49.6372	797.1687	7.6509
13.440	MF	0.33	483.2584	9622.1514	92.3491
			Totals:	10419.3201	100.0000

HRMS (ESI-TOF) m/z: $[M + Na]^+$ Calcd for $C_{25}H_{14}O_4Na^+$ 405.1097; Found 405.1099.

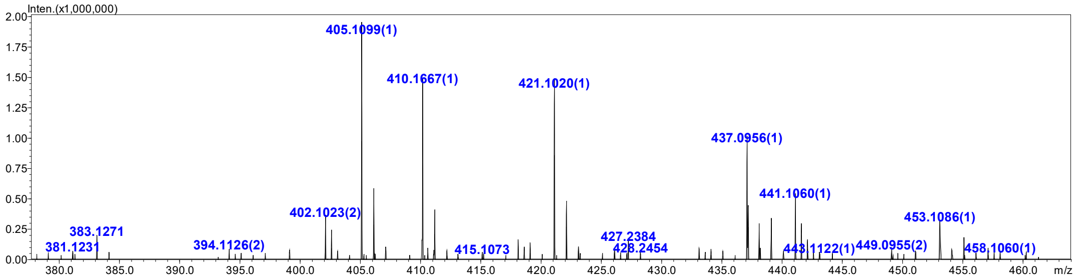

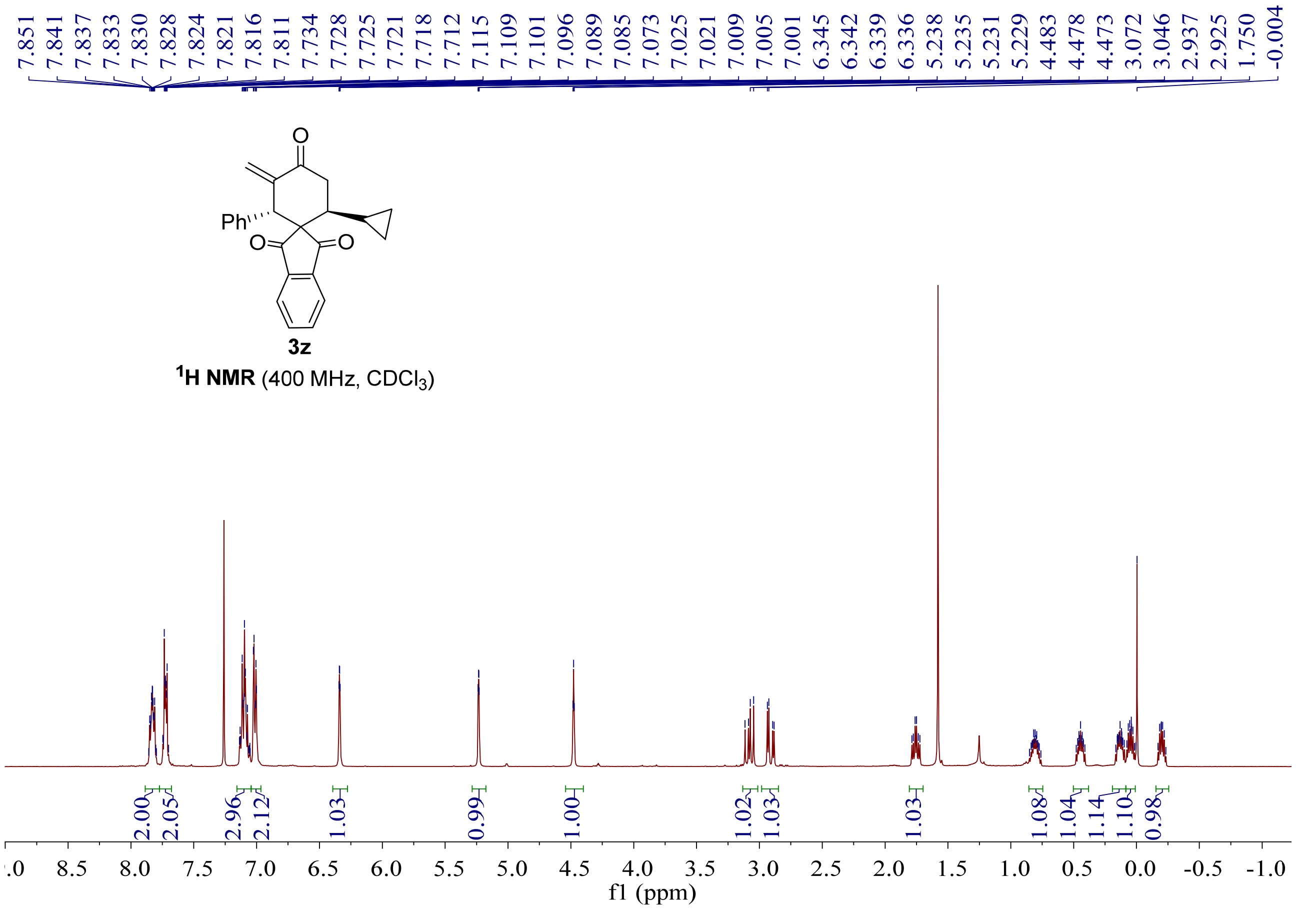
7.851 7.841 7.837 7.833 7.830 7.828 7.824 7.821 7.816 7.811 7.734 7.728 7.725 7.721 7.718 7.712 7.115 7.109 7.101 7.096 7.089 7.085 7.073 7.025 7.021 7.009 7.005 7.001 6.345 6.342 6.339 6.336 5.238 5.235 5.231 5.229 4.483 4.478 4.473 3.072 3.046 2.937 2.925 1.750 -0.004
Ph
O
3z
1H NMR (400 MHz, CDCl3)
2.00 2.05 2.96 2.12 1.03 0.99 1.00 1.02 1.03 1.03 1.08 1.04 1.14 1.10 0.98
.0 8.5 8.0 7.5 7.0 6.5 6.0 5.5 5.0 4.5 4.0 3.5 3.0 2.5 2.0 1.5 1.0 0.5 0.0 -0.5 -1.0
f1 (ppm)

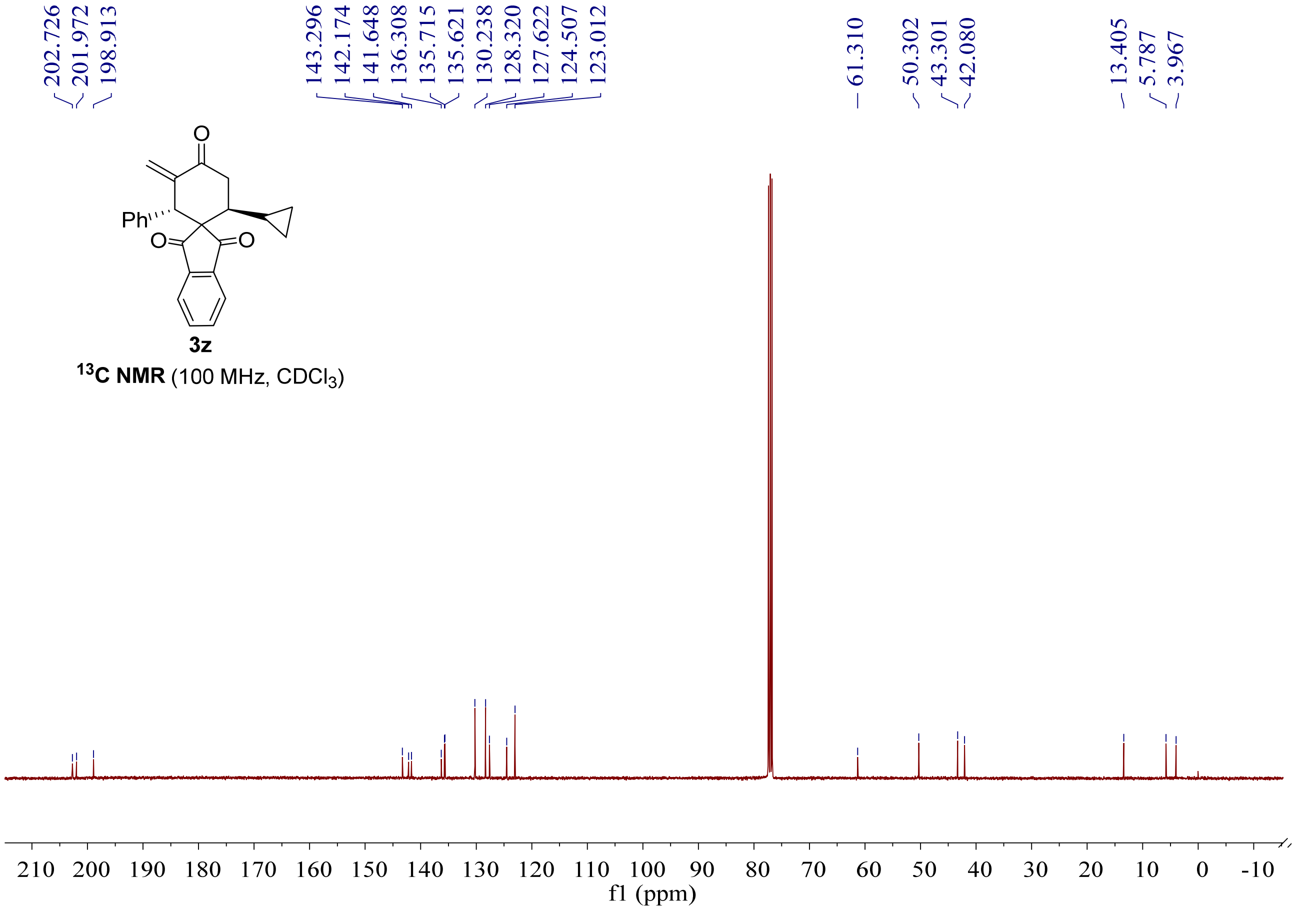
202.726 201.972 198.913
143.296 142.174 141.648 136.308 135.715 135.621 130.238 128.320 127.622 124.507 123.012
61.310
50.302 43.301 42.080
13.405 5.787 3.967
Ph
O
3z
13C NMR (100 MHz, CDCl3)
210 200 190 180 170 160 150 140 130 120 110 100 90 80 70 60 50 40 30 20 10 0 -10
f1 (ppm)

Daicel Chiral AD-H Column (*i*PrOH/*n*-hexane = 10/90, 1.0 mL/min)

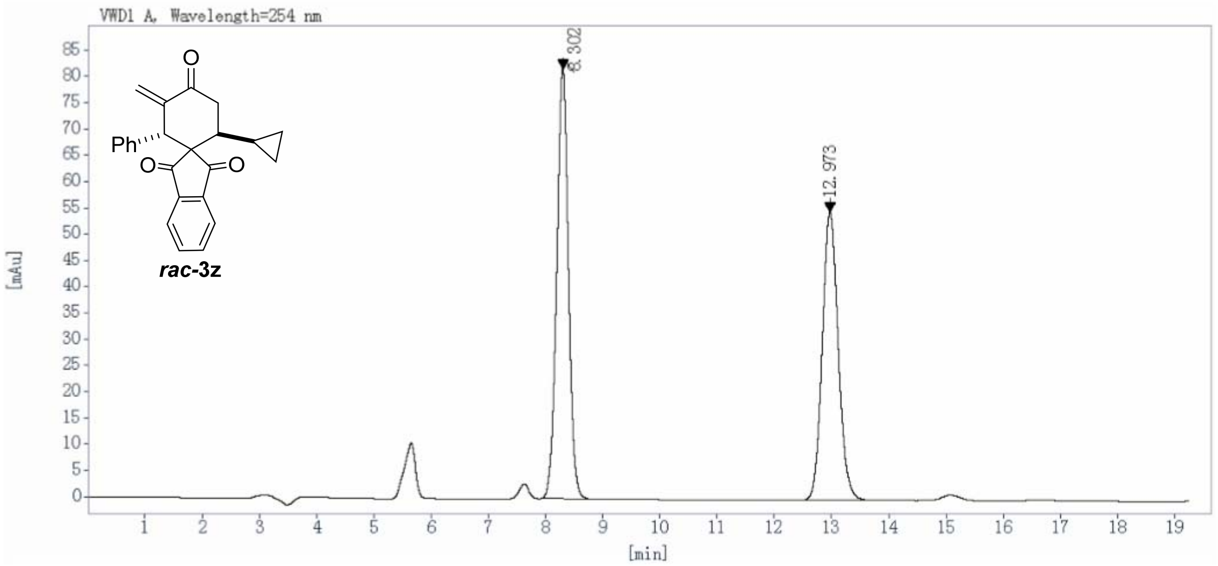

Ret Time [min]	Peak Type	Width [min]	Height [mAU]	Area [mAU*s]	Area [%]
8.302	MM	0.24	81.8294	1159.5342	52.1519
12.973	BB	0.30	54.9037	1063.8427	47.8481
			Totals:	2223.3768	100.0000

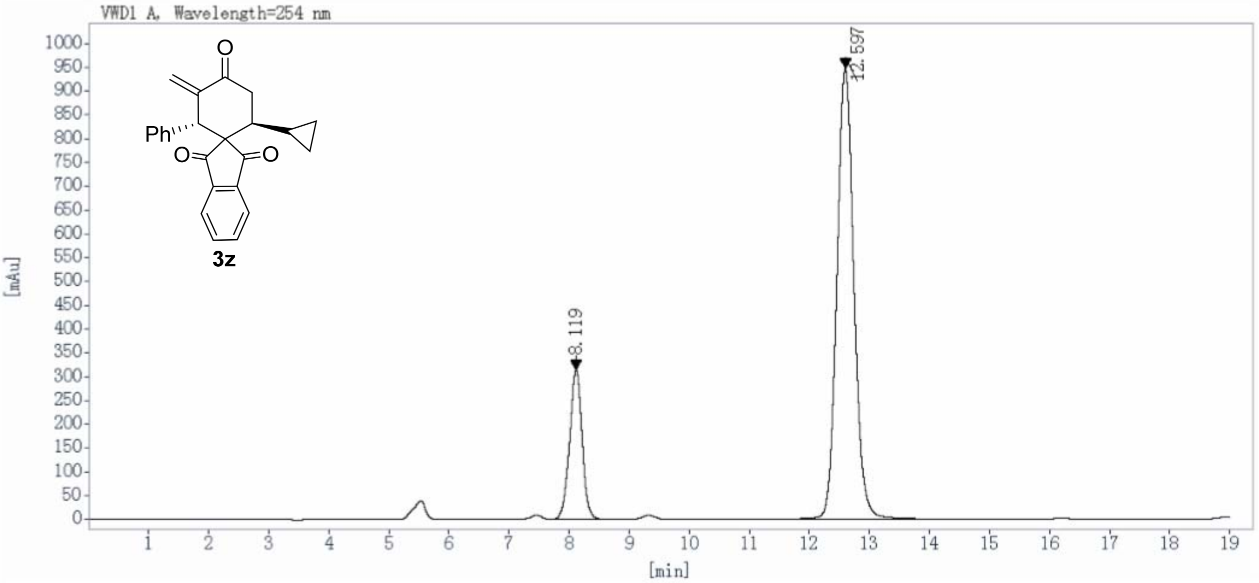

Ret Time [min]	Peak Type	Width [min]	Height [mAU]	Area [mAU*s]	Area [%]
8.119	VB	0.22	313.5812	4550.4336	19.5090
12.597	BB	0.31	948.2735	18774.3750	80.4910
			Totals:	23324.8086	100.0000

HRMS (ESI-TOF) m/z: [M + Na]$^+$ Calcd for $C_{24}H_{20}O_3Na^+$ 379.1305; Found 379.1324.

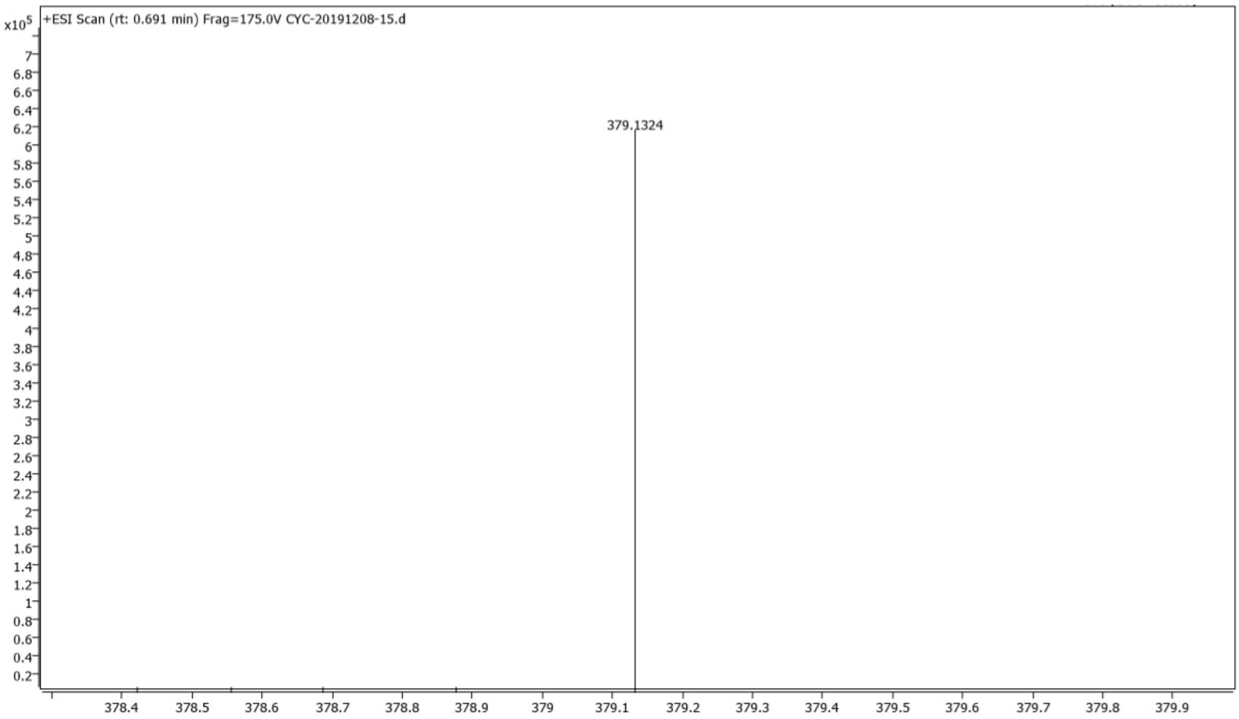

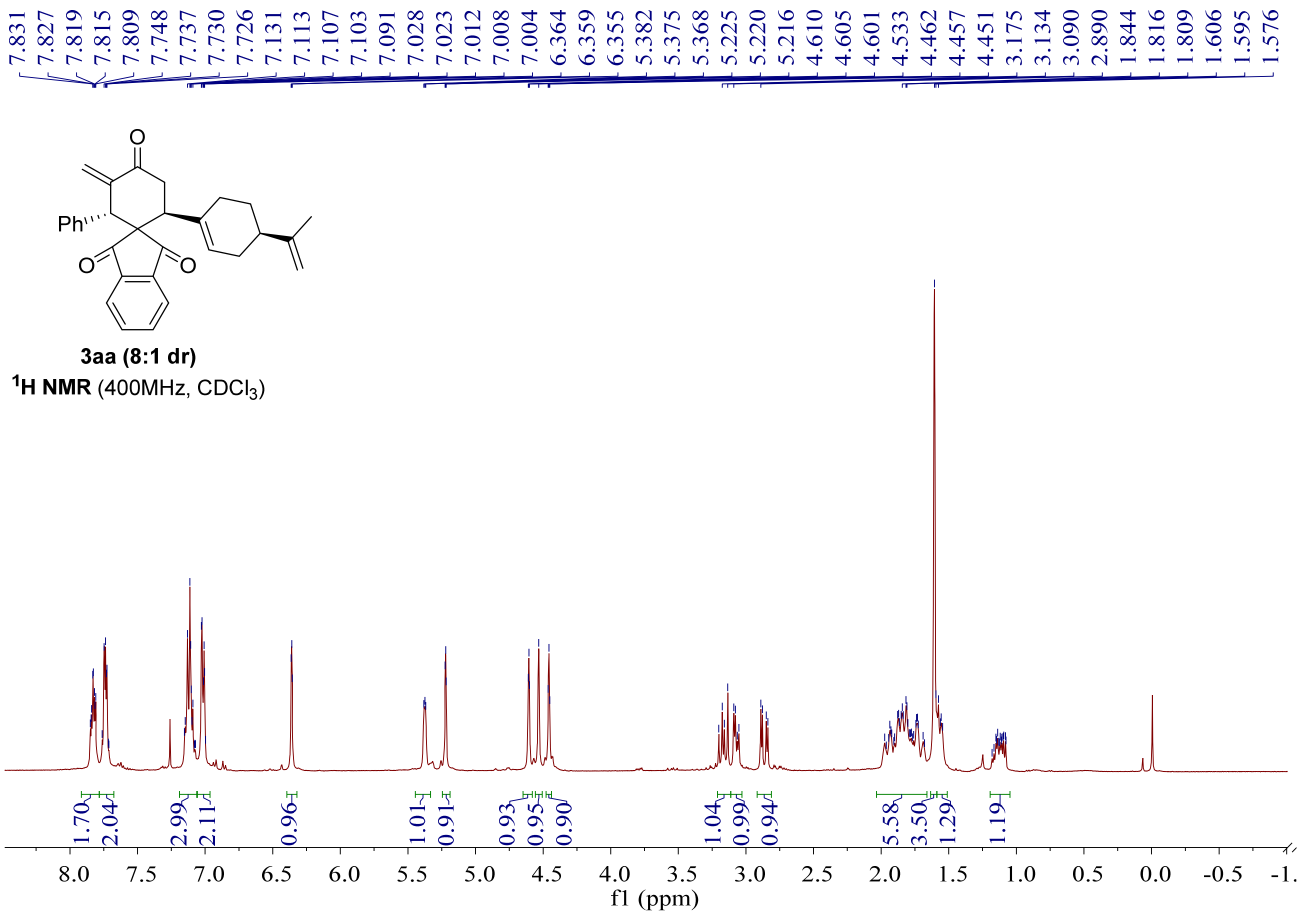
7.831 7.827 7.819 7.815 7.809 7.748 7.737 7.730 7.726 7.131 7.113 7.107 7.103 7.091 7.028 7.023 7.012 7.008 7.004 6.364 6.359 6.355 5.382 5.375 5.368 5.225 5.220 5.216 4.610 4.605 4.601 4.533 4.462 4.457 4.451 3.175 3.134 3.090 2.890 1.844 1.816 1.809 1.606 1.595 1.576
O
O
O
Ph
3aa (8:1 dr)
1H NMR (400MHz, CDCl3)
1.70 2.04 2.99 2.11 0.96 1.01 0.91 0.93 0.95 0.90 1.04 0.99 0.94 5.58 3.50 1.29 1.19
8.0 7.5 7.0 6.5 6.0 5.5 5.0 4.5 4.0 3.5 3.0 2.5 2.0 1.5 1.0 0.5 0.0 -0.5 -1.
f1 (ppm)

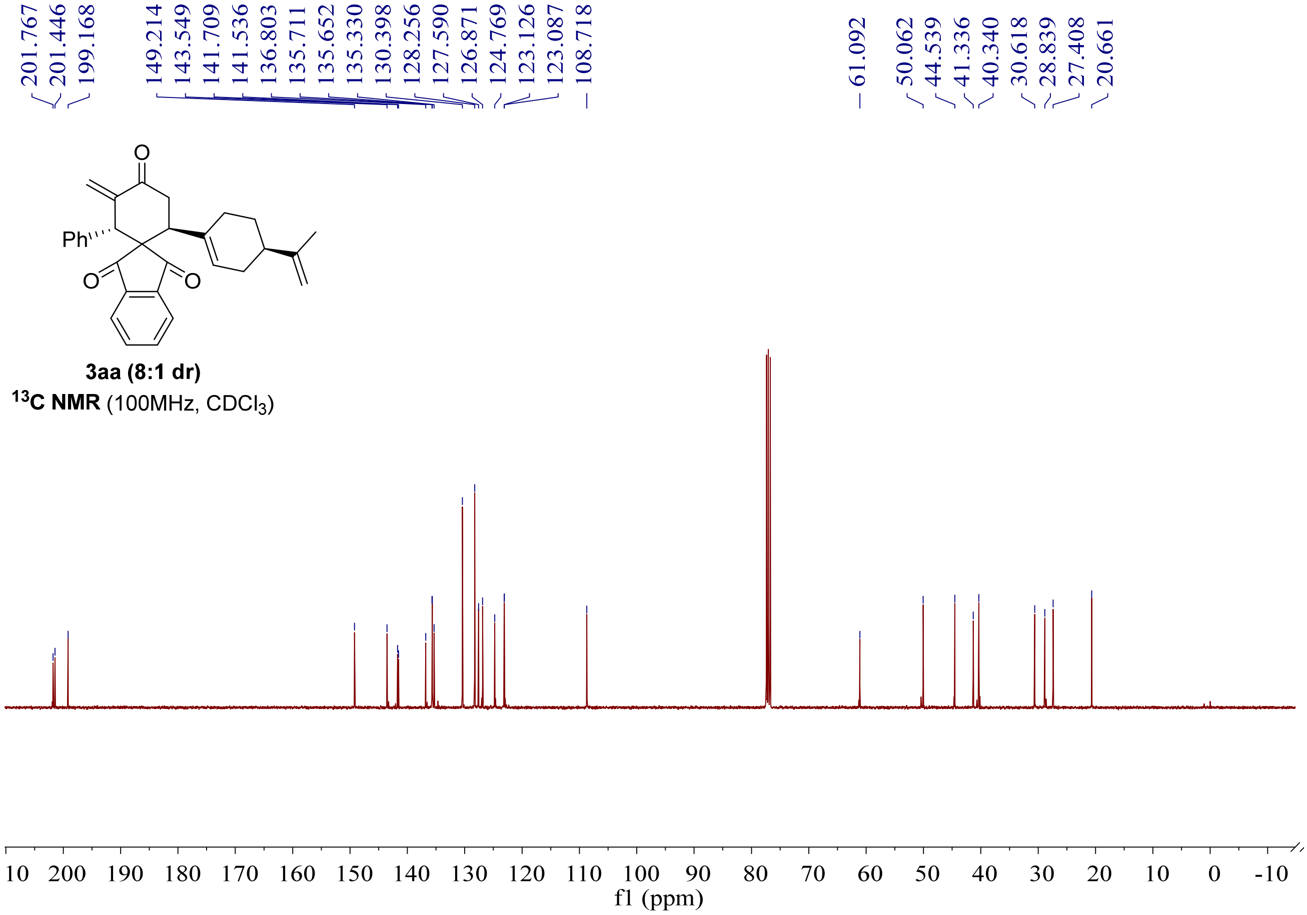
201.767 201.446 199.168 149.214 143.549 141.709 141.536 136.803 135.711 135.652 135.330 130.398 128.256 127.590 126.871 124.769 123.126 123.087 108.718 61.092 50.062 44.539 41.336 40.340 30.618 28.839 27.408 20.661
O
O
O
Ph
3aa (8:1 dr)
13C NMR (100MHz, CDCl3)
10 200 190 180 170 160 150 140 130 120 110 100 90 80 70 60 50 40 30 20 10 0 -10
f1 (ppm)

HRMS (ESI-TOF) m/z: $[M + Na]^+$ Calcd for $C_{30}H_{28}O_3Na^+$ 459.1931; Found 459.1932.

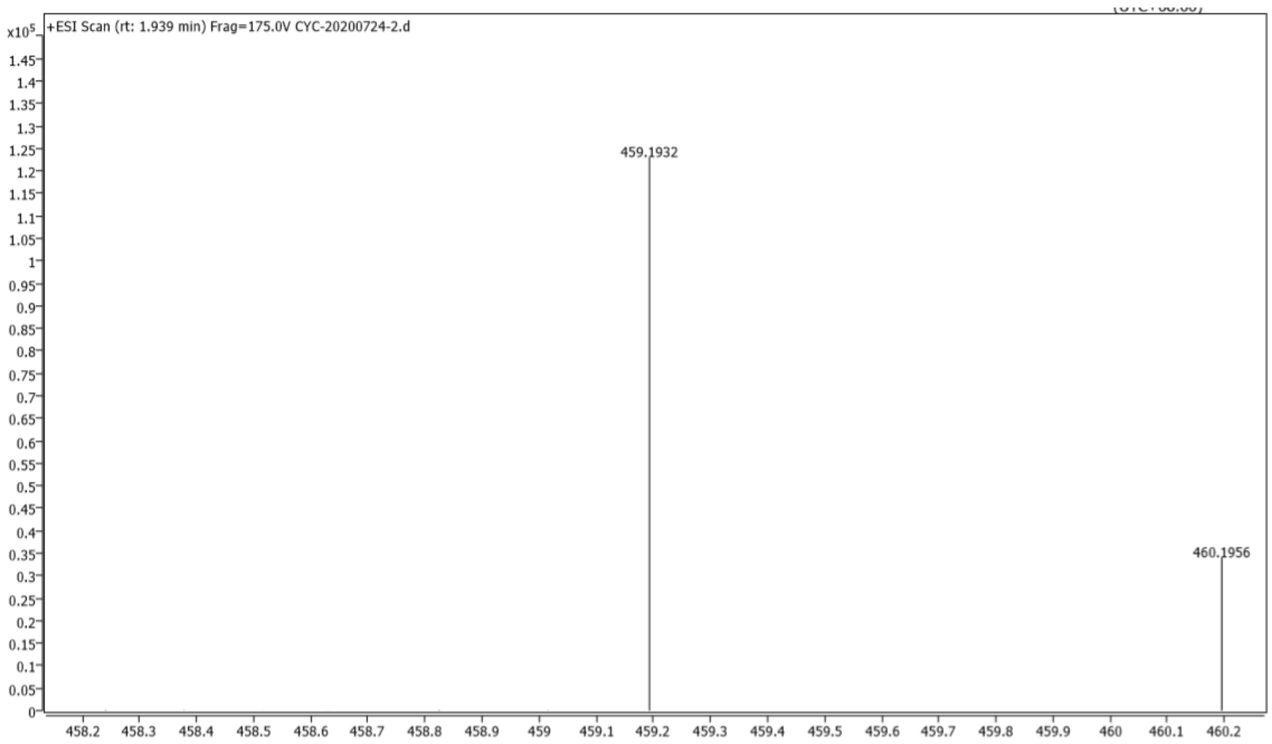

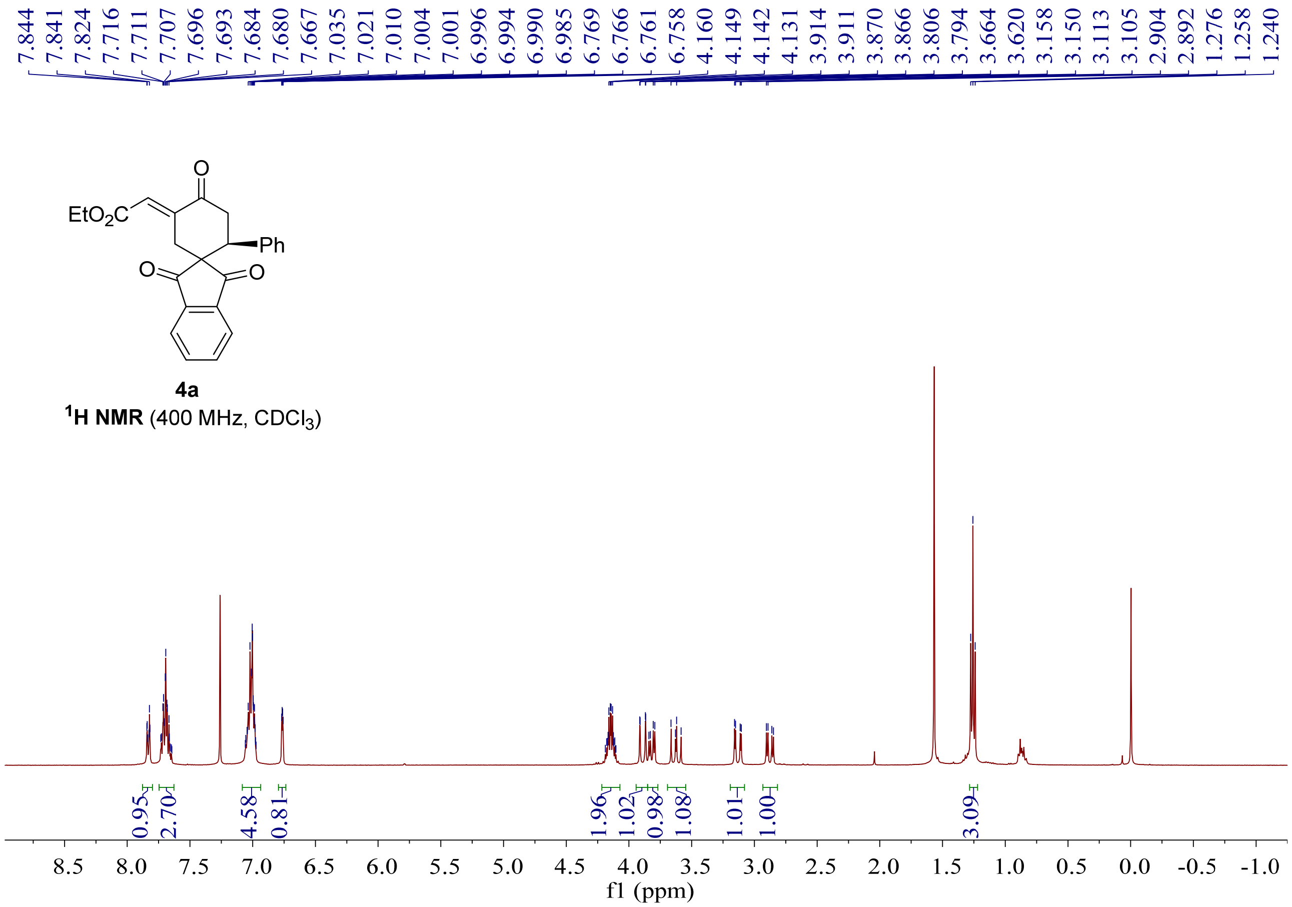

4a
^{1}H NMR (400 MHz, $CDCl_3$)

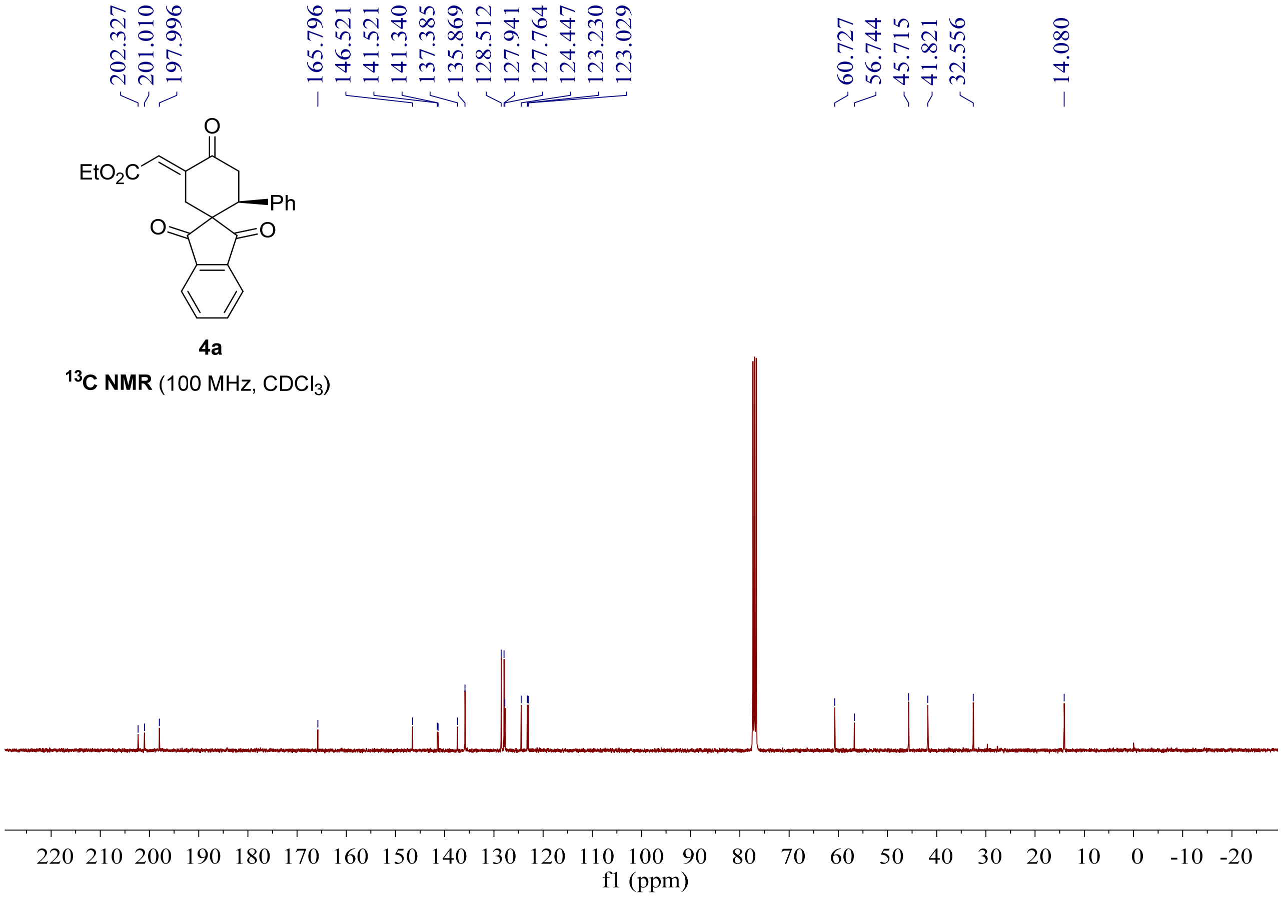

4a
^{13}C NMR (100 MHz, $CDCl_3$)

Daicel Chiral AD-H Column, (iPrOH/n-hexane = 40/60, 1.0 mL/min)

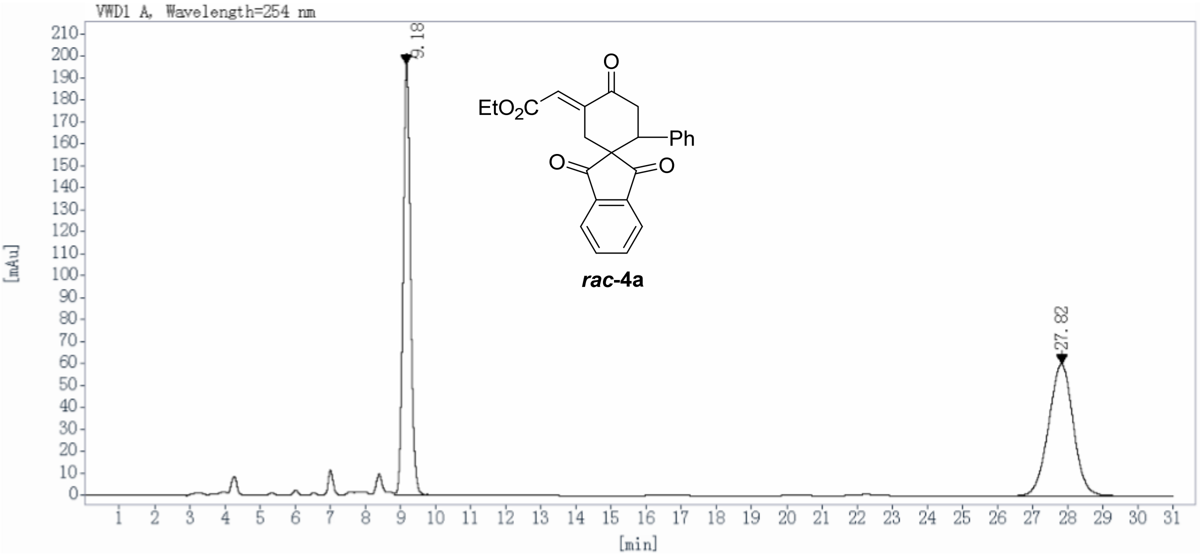

Ret Time [min]	Peak Type	Width [min]	Height [mAU]	Area [mAU*s]	Area [%]
9.180	VB	0.22	195.8432	2835.0830	49.3663
27.820	BB	0.75	59.8321	2907.8706	50.6337
			Totals:	5742.9536	100.0000

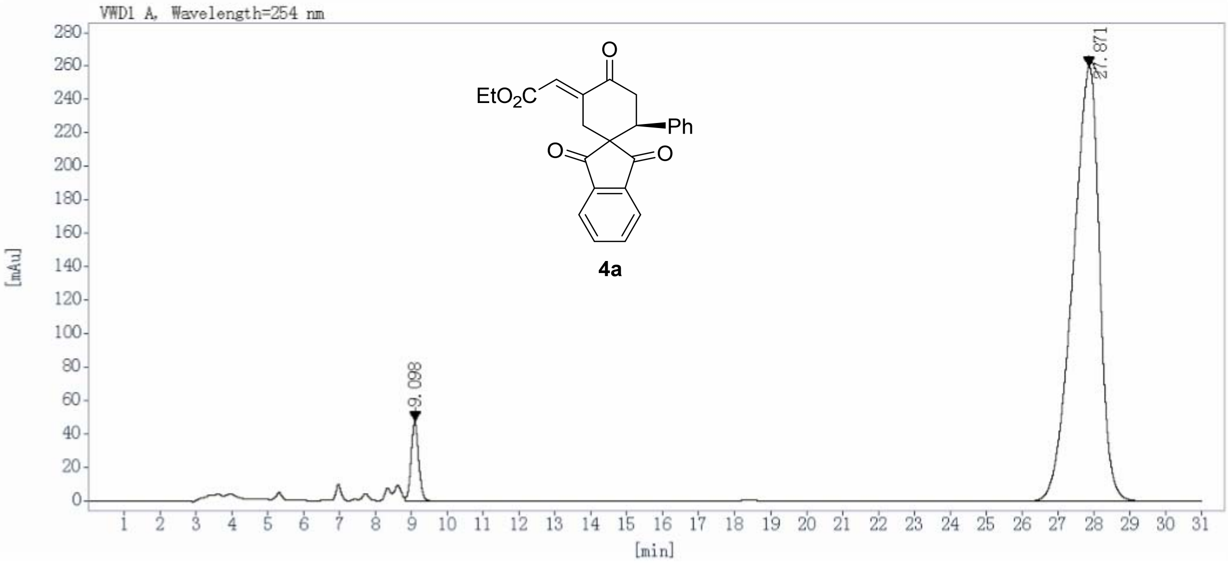

Ret Time [min]	Peak Type	Width [min]	Height [mAU]	Area [mAU*s]	Area [%]
9.098	FM	0.24	47.4678	688.1624	4.8934
27.871	BB	0.79	259.8821	13374.9316	95.1066
			Totals:	14063.0940	100.0000

HRMS (ESI-TOF) m/z: [M + Na]$^+$ Calcd for $C_{24}H_{20}O_5Na^+$ 411.1203; Found 411.1206.

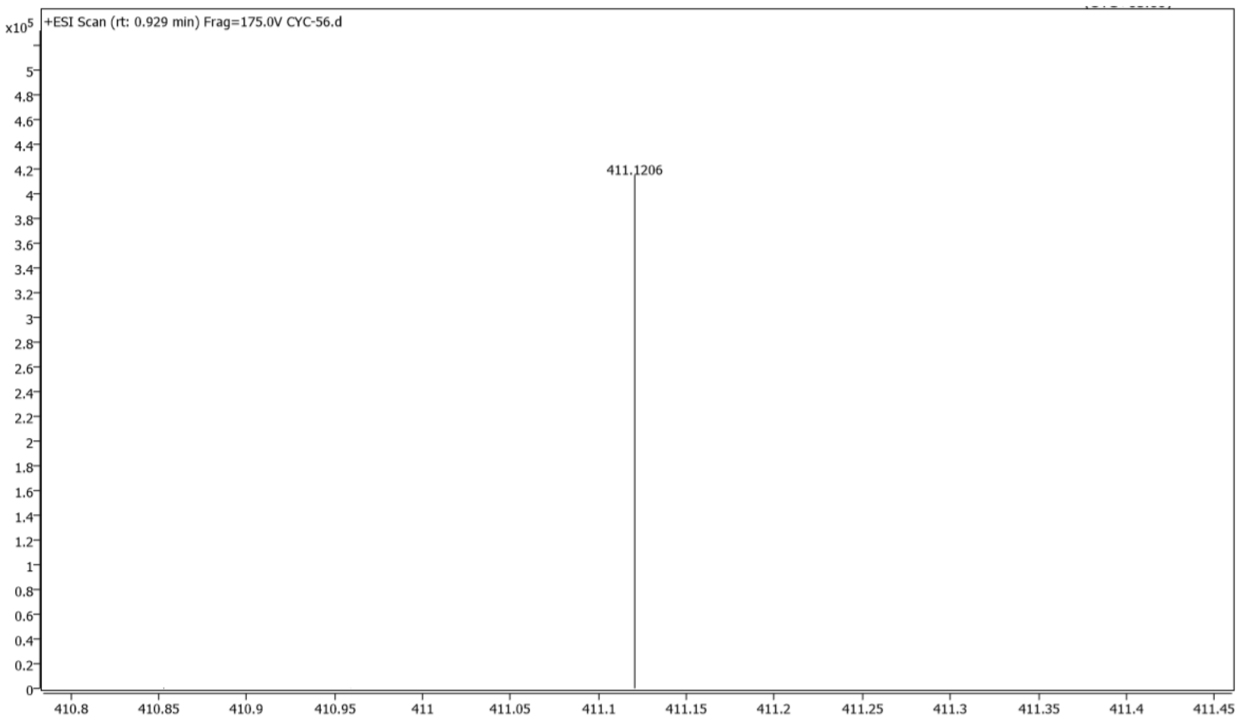

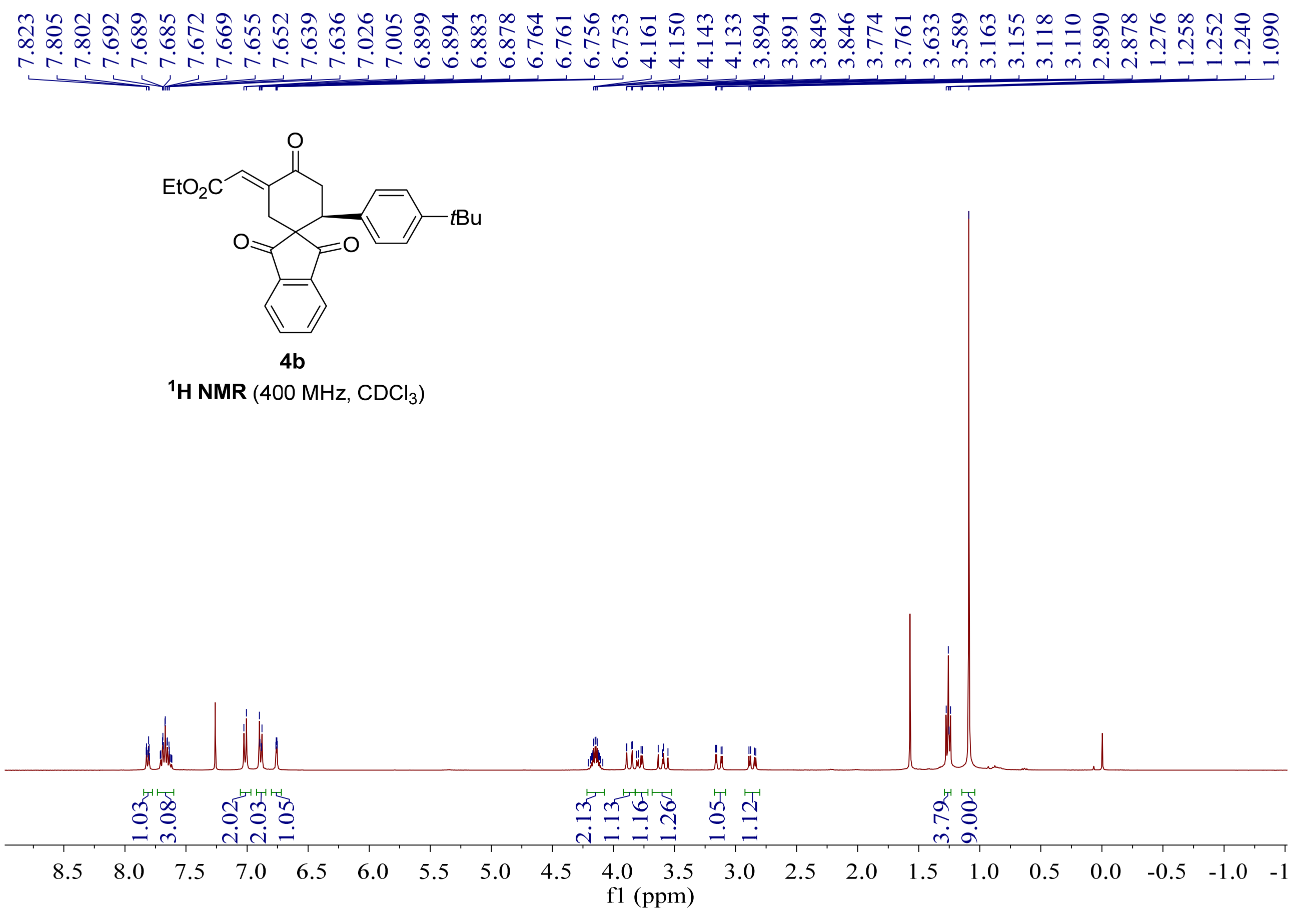
7.823 7.805 7.802 7.692 7.689 7.685 7.672 7.669 7.655 7.652 7.639 7.636 7.026 7.005 6.899 6.894 6.883 6.878 6.764 6.761 6.756 6.753 4.161 4.150 4.143 4.133 3.894 3.891 3.849 3.846 3.774 3.761 3.633 3.589 3.163 3.155 3.118 3.110 2.890 2.878 1.276 1.258 1.252 1.240 1.090
EtO2C
O
tBu
4b
1H NMR (400 MHz, CDCl3)
1.03 3.08 2.02 2.03 1.05 2.13 1.13 1.16 1.26 1.05 1.12 3.79 9.00
f1 (ppm)

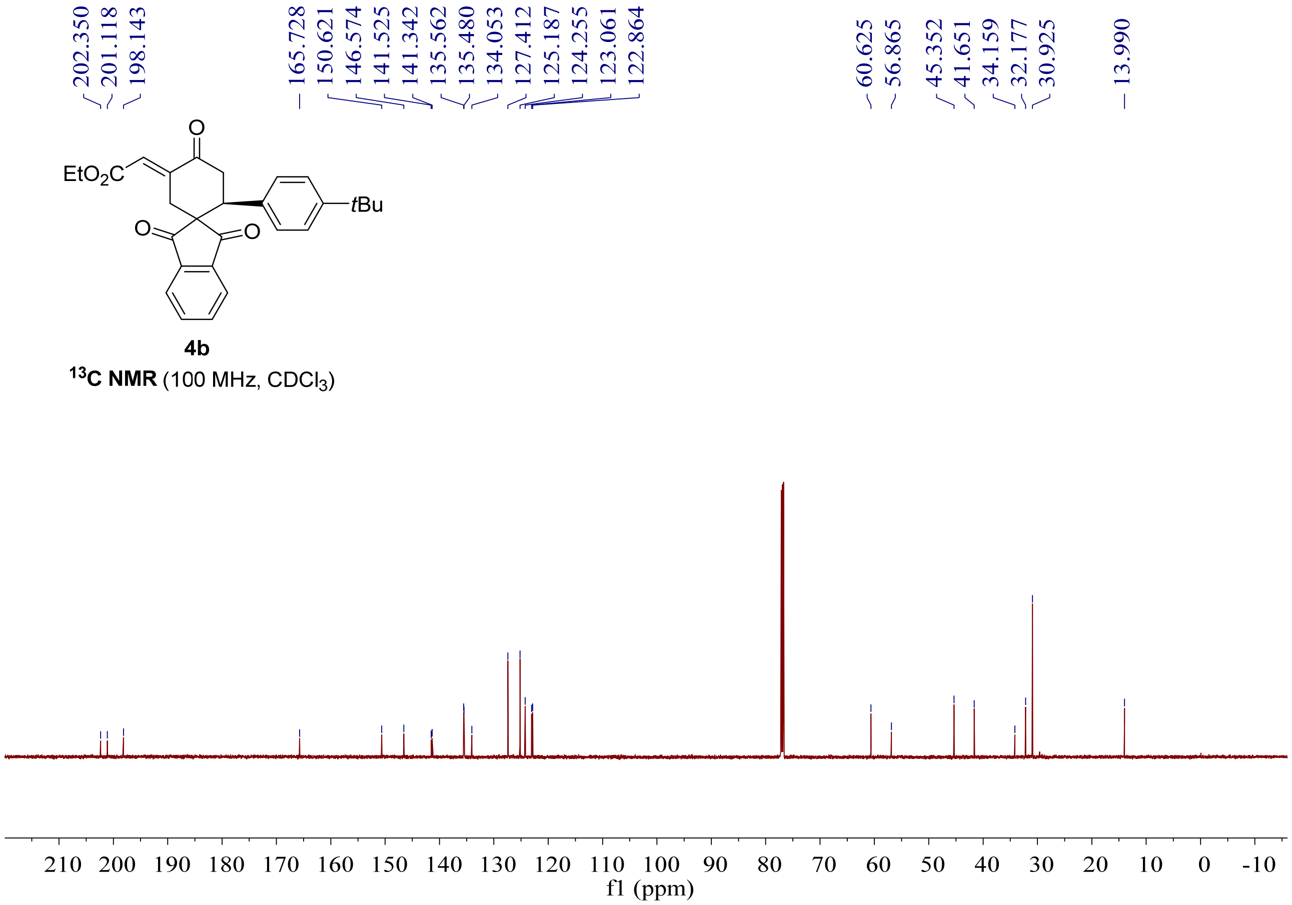
202.350 201.118 198.143 165.728 150.621 146.574 141.525 141.342 135.562 135.480 134.053 127.412 125.187 124.255 123.061 122.864 60.625 56.865 45.352 41.651 34.159 32.177 30.925 13.990
EtO2C
O
tBu
4b
13C NMR (100 MHz, CDCl3)
f1 (ppm)

Daicel Chiral IF Column, (*i*PrOH/*n*-hexane = 40/60, 1.0 mL/min)

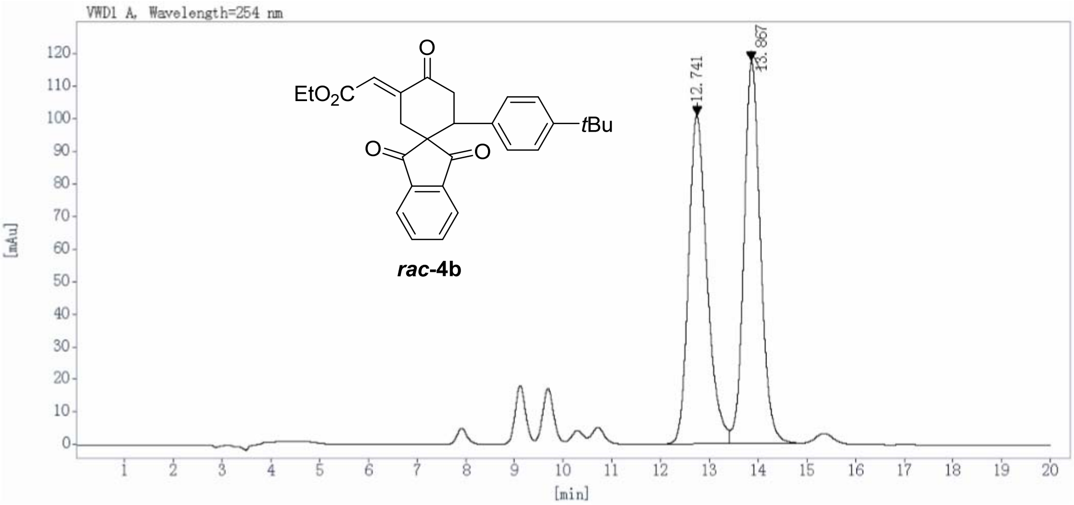

Ret Time [min]	Peak Type	Width [min]	Height [mAU]	Area [mAU*s]	Area [%]
12.741	BV	0.40	100.6865	2666.3538	49.1048
13.867	VB	0.36	117.5403	2763.5706	50.8952
			Totals:	5429.9243	100.0000

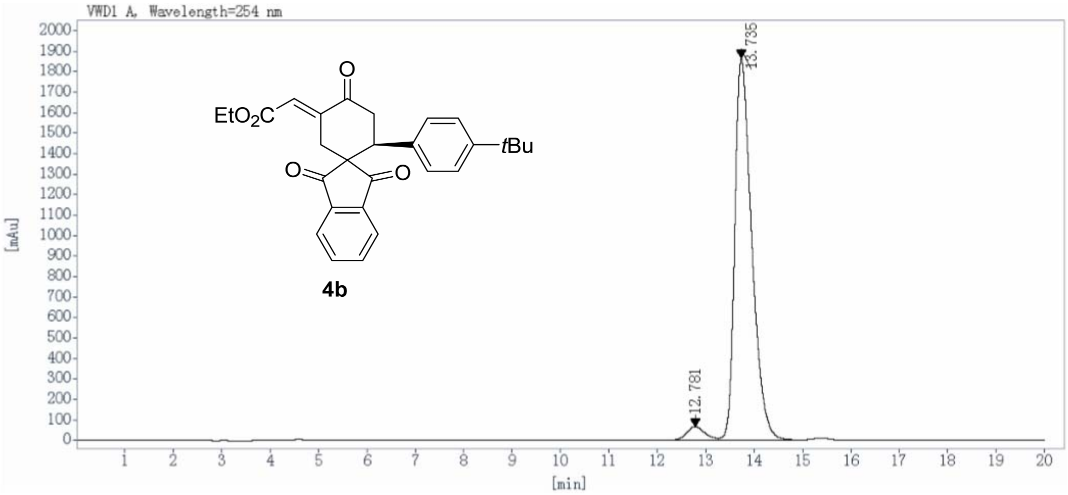

Ret Time [min]	Peak Type	Width [min]	Height [mAU]	Area [mAU*s]	Area [%]
12.781	FM	0.44	66.1446	1759.6094	3.7262
13.735	FM	0.41	1865.1442	45463.6016	96.2738
			Totals:	47223.2109	100.0000

HRMS (ESI-TOF) m/z: $[M + Na]^+$ Calcd for $C_{28}H_{28}O_5Na^+$ 467.1829; Found 467.1827.

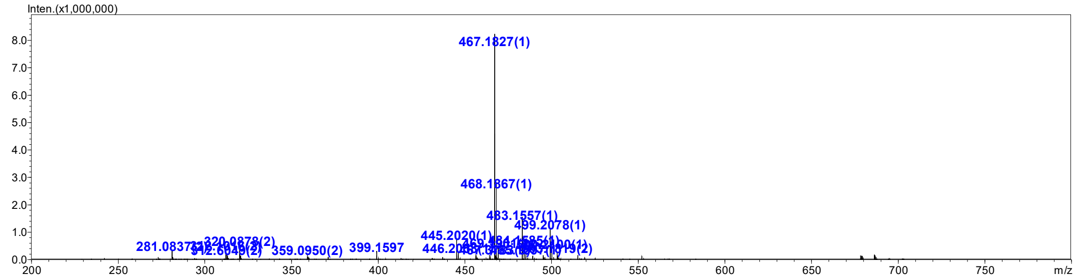

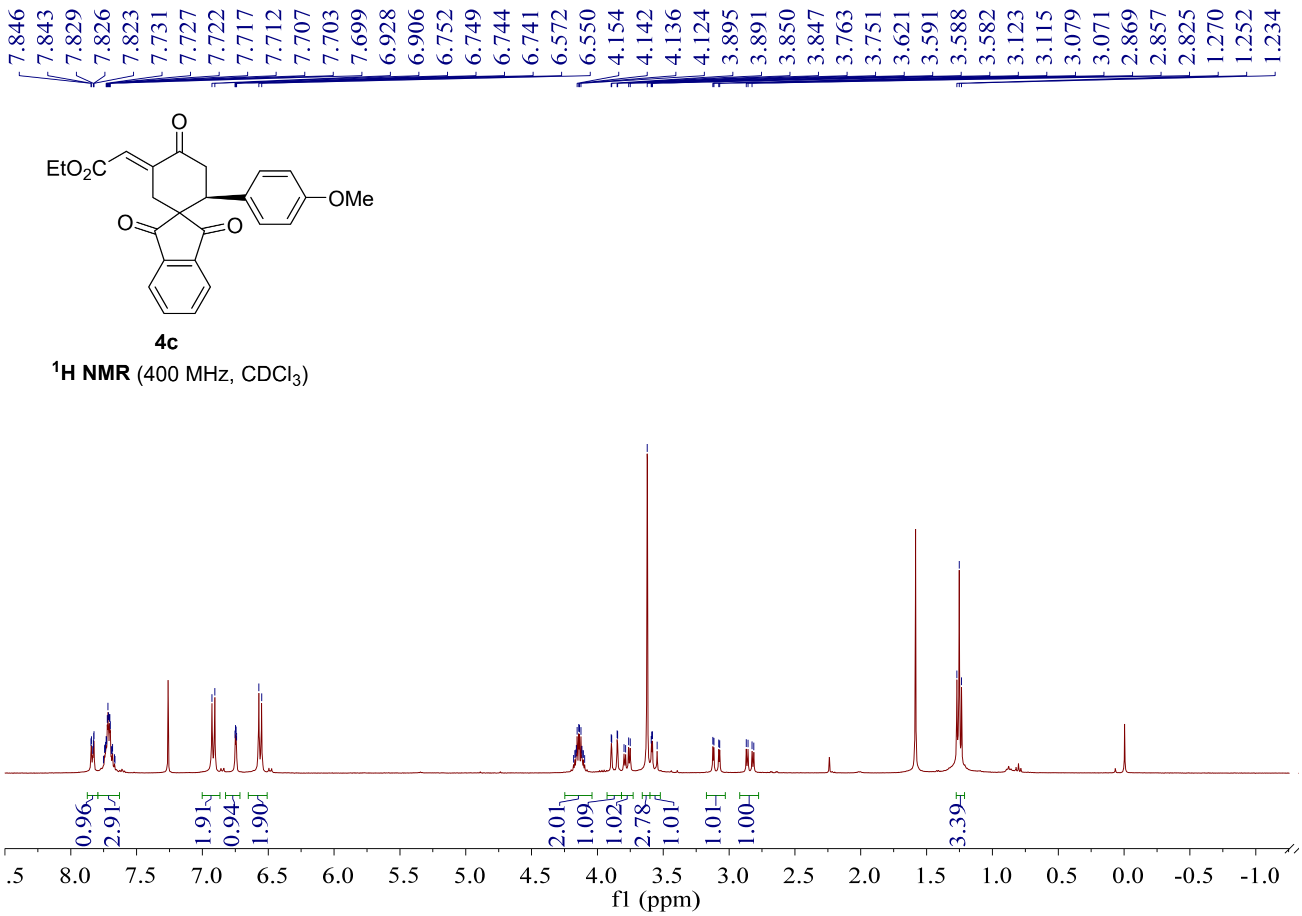
7.846 7.843 7.829 7.826 7.823 7.731 7.727 7.722 7.717 7.712 7.707 7.703 7.699 6.928 6.906 6.752 6.749 6.744 6.741 6.572 6.550 4.154 4.142 4.136 4.124 3.895 3.891 3.850 3.847 3.763 3.751 3.621 3.591 3.588 3.582 3.123 3.115 3.079 3.071 2.869 2.857 2.825 1.270 1.252 1.234
EtO2C
OMe
4c
1H NMR (400 MHz, CDCl3)
0.96 2.91 1.91 0.94 1.90 2.01 1.09 1.02 2.78 1.01 1.01 1.00 3.39
f1 (ppm)

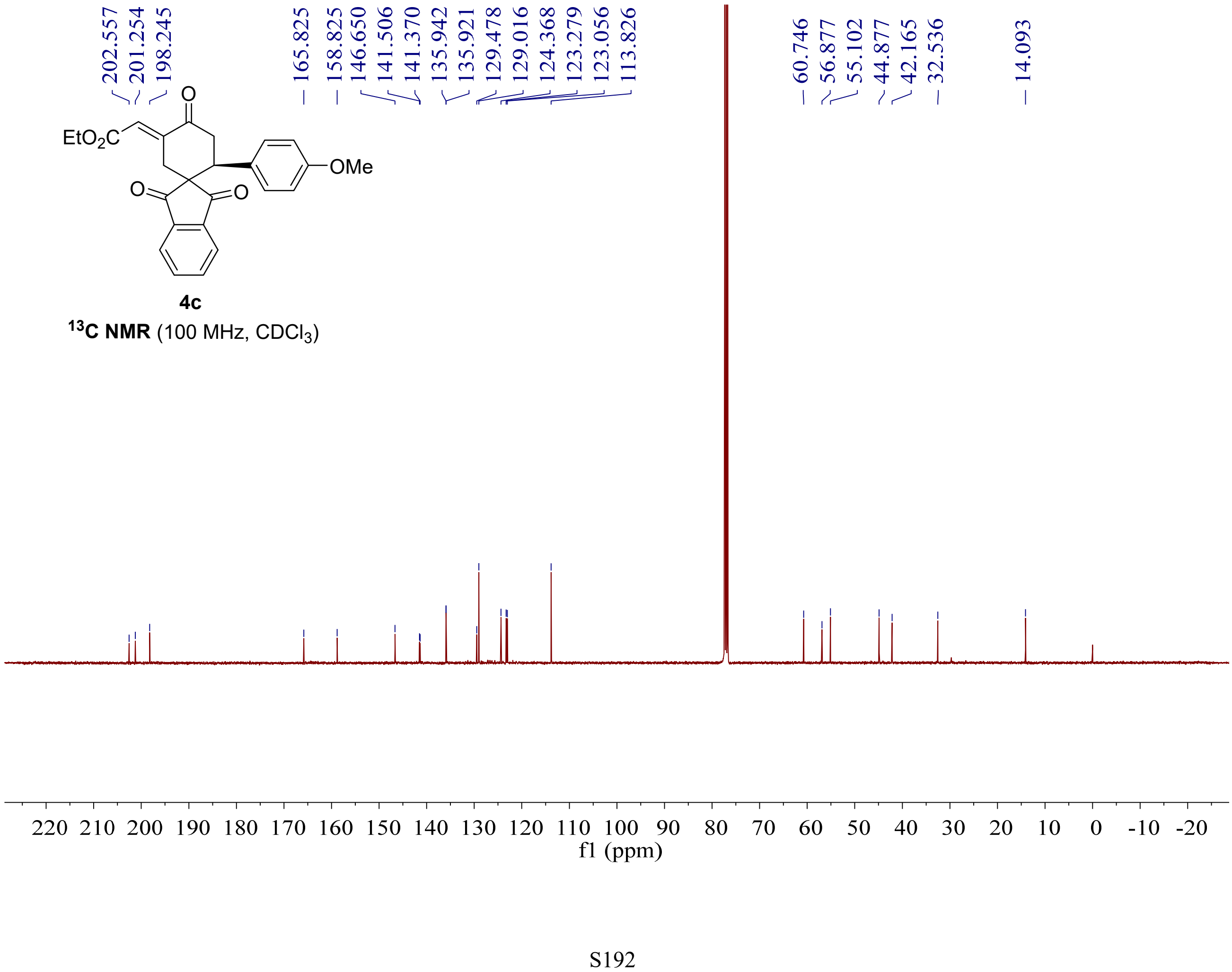
202.557 201.254 198.245 165.825 158.825 146.650 141.506 141.370 135.942 135.921 129.478 129.016 124.368 123.279 123.056 113.826 60.746 56.877 55.102 44.877 42.165 32.536 14.093
EtO2C
OMe
4c
13C NMR (100 MHz, CDCl3)
f1 (ppm)

Given the catalytic systems of generated compound **4c** and **3s** are exactly same and the stereoselectivity is directly controlled by ion-pair catalyst in the first step of this palladium promoted annulation, combining the 1H-1H NOESY spectrum of **4c**, the configuration was supposed to “*trans*”.

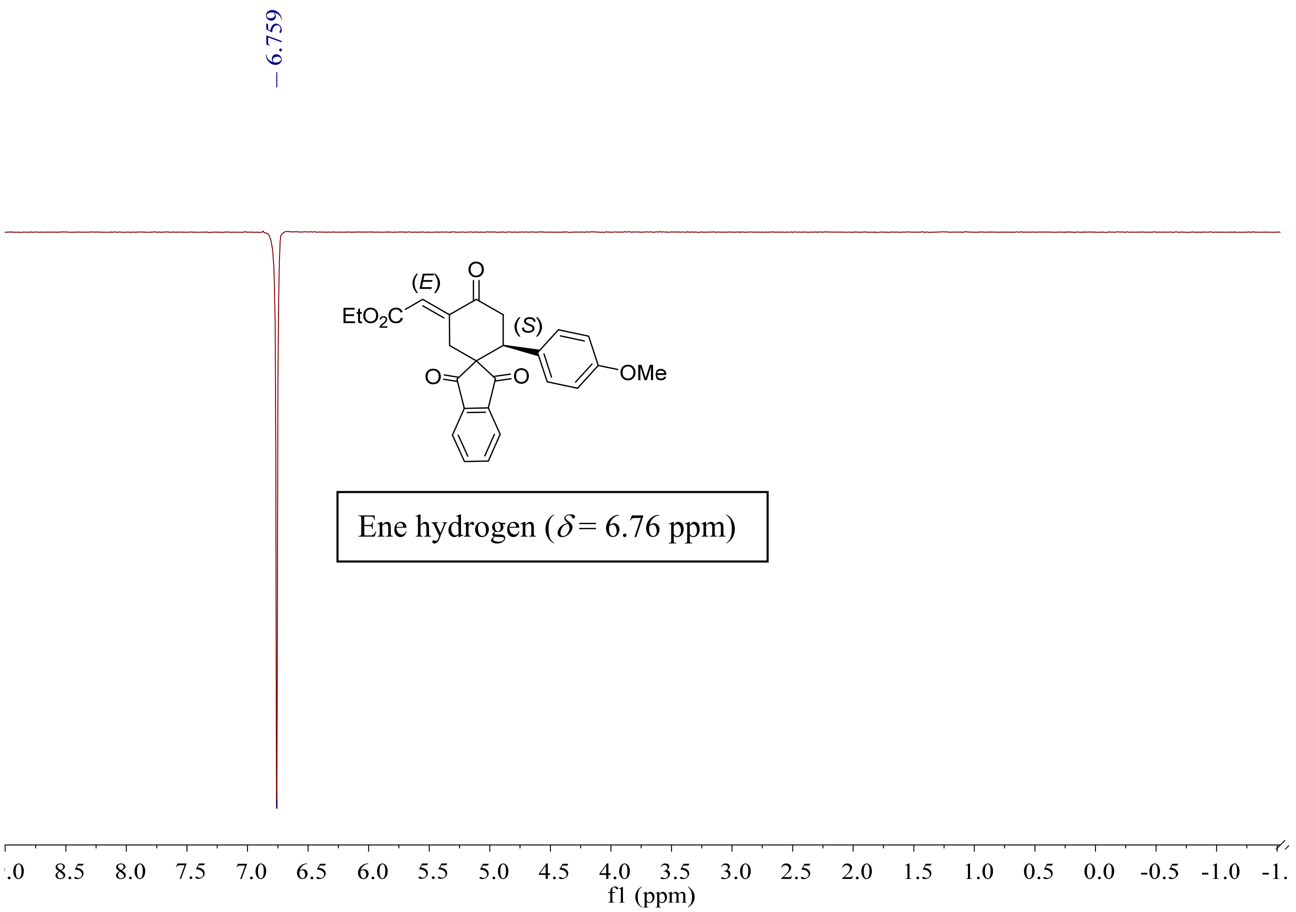

The ^{1}**H-**^{1}**H NOESY** (δ = 6.76 ppm) of **4c**.

Daicel Chiral AD-H Column, (*i*PrOH/*n*-hexane = 40/60, 1.0 mL/min)

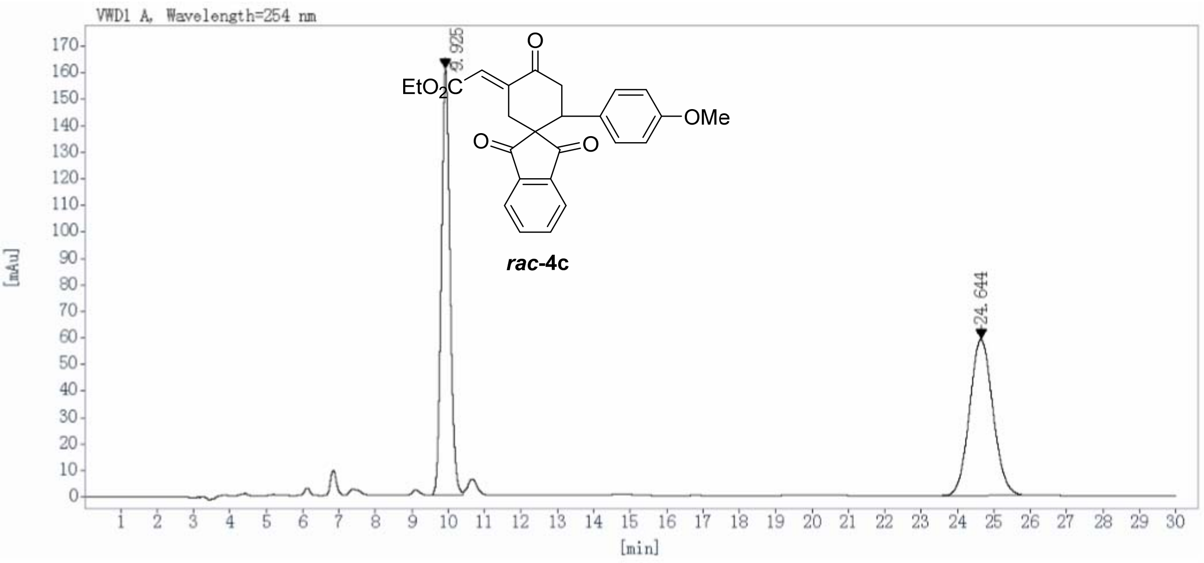

Ret Time [min]	Peak Type	Width [min]	Height [mAU]	Area [mAU*s]	Area [%]
9.925	MF	0.28	161.0131	2665.7849	50.2545
24.644	BB	0.70	58.9427	2638.7834	49.7455
			Totals:	5304.5684	100.0000

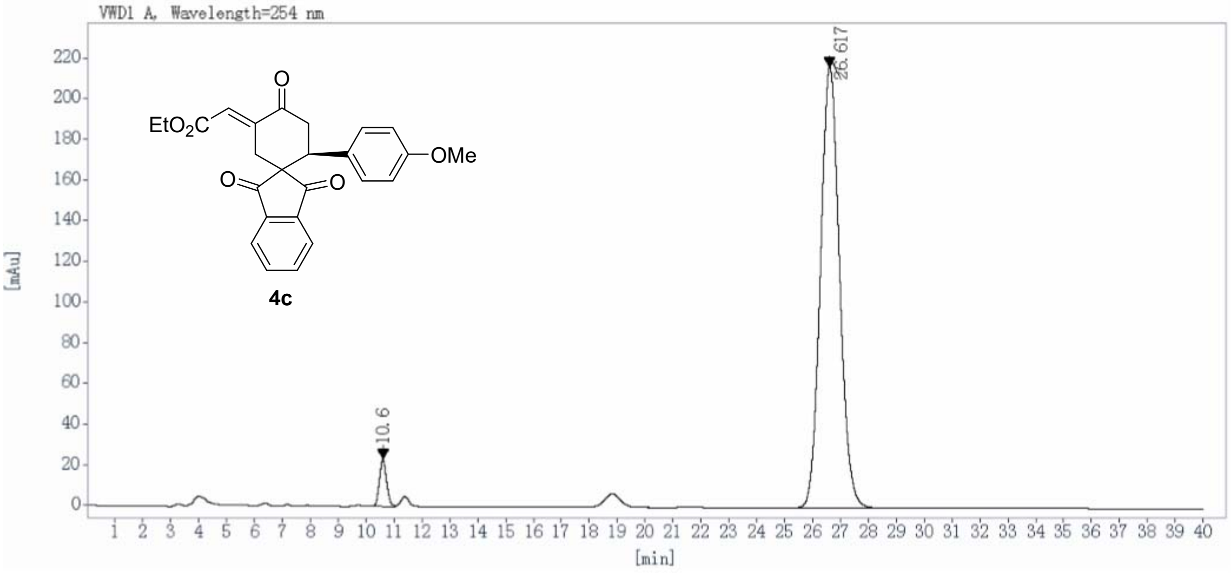

Ret Time [min]	Peak Type	Width [min]	Height [mAU]	Area [mAU*s]	Area [%]
10.600	BV	0.27	23.2621	413.2669	3.8613
26.617	BB	0.74	216.8391	10289.4336	96.1387
			Totals:	10702.7005	100.0000

HRMS (ESI-TOF) m/z: [M + Na]$^+$ Calcd for $C_{25}H_{22}O_6Na^+$ 441.1309; Found 441.1306.

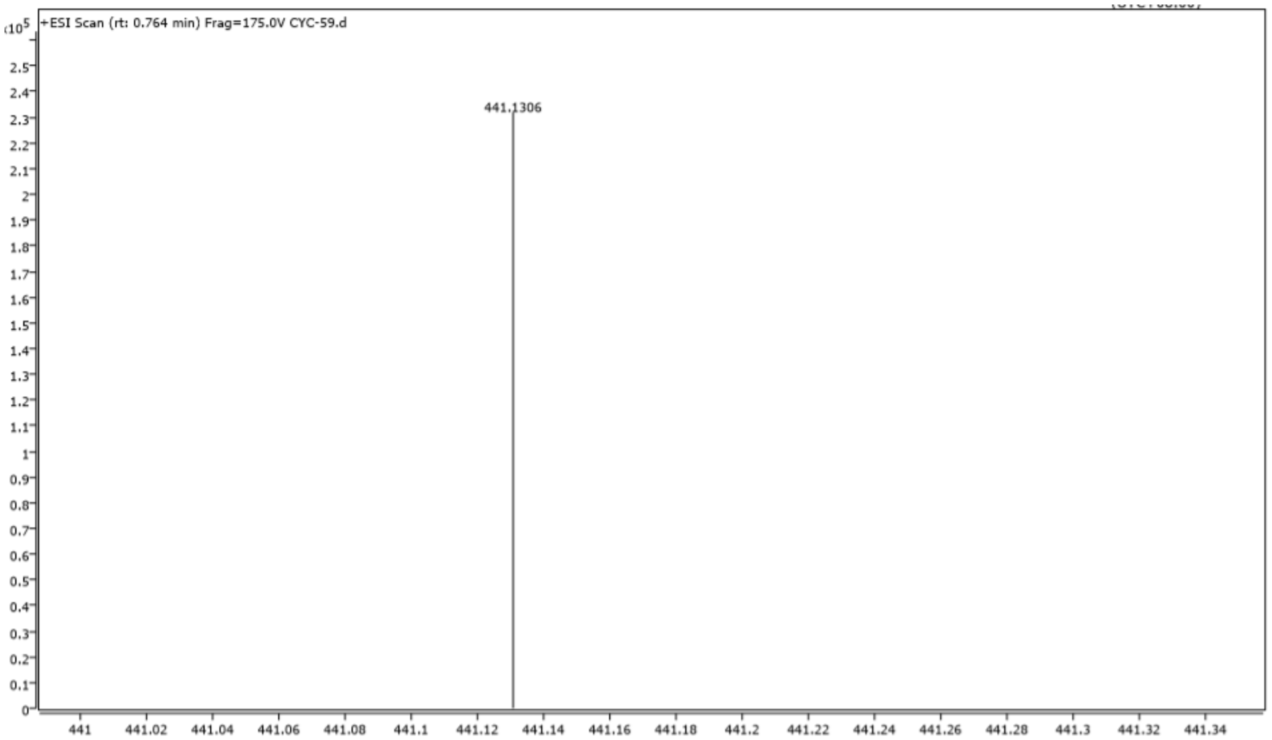

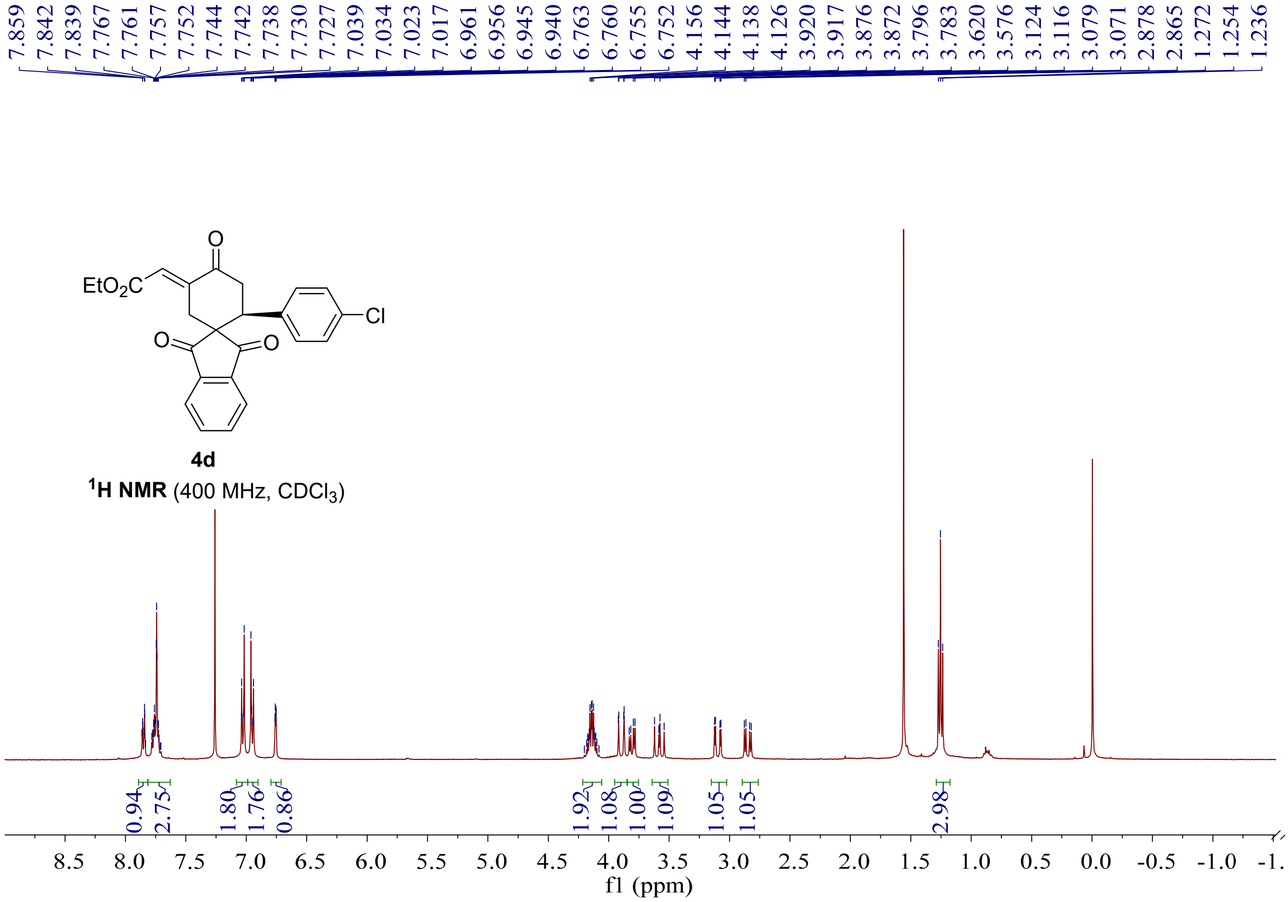
7.859 7.842 7.839 7.767 7.761 7.757 7.752 7.744 7.742 7.738 7.730 7.727 7.039 7.034 7.023 7.017 6.961 6.956 6.945 6.940 6.763 6.760 6.755 6.752 4.156 4.144 4.138 4.126 3.920 3.917 3.876 3.872 3.796 3.783 3.620 3.576 3.124 3.116 3.079 3.071 2.878 2.865 1.272 1.254 1.236
EtO2C
O
Cl
O
O
4d
1H NMR (400 MHz, CDCl3)
0.94 2.75 1.80 1.76 0.86 1.92 1.08 1.00 1.09 1.05 1.05 2.98
8.5 8.0 7.5 7.0 6.5 6.0 5.5 5.0 4.5 4.0 3.5 3.0 2.5 2.0 1.5 1.0 0.5 0.0 -0.5 -1.0
f1 (ppm)

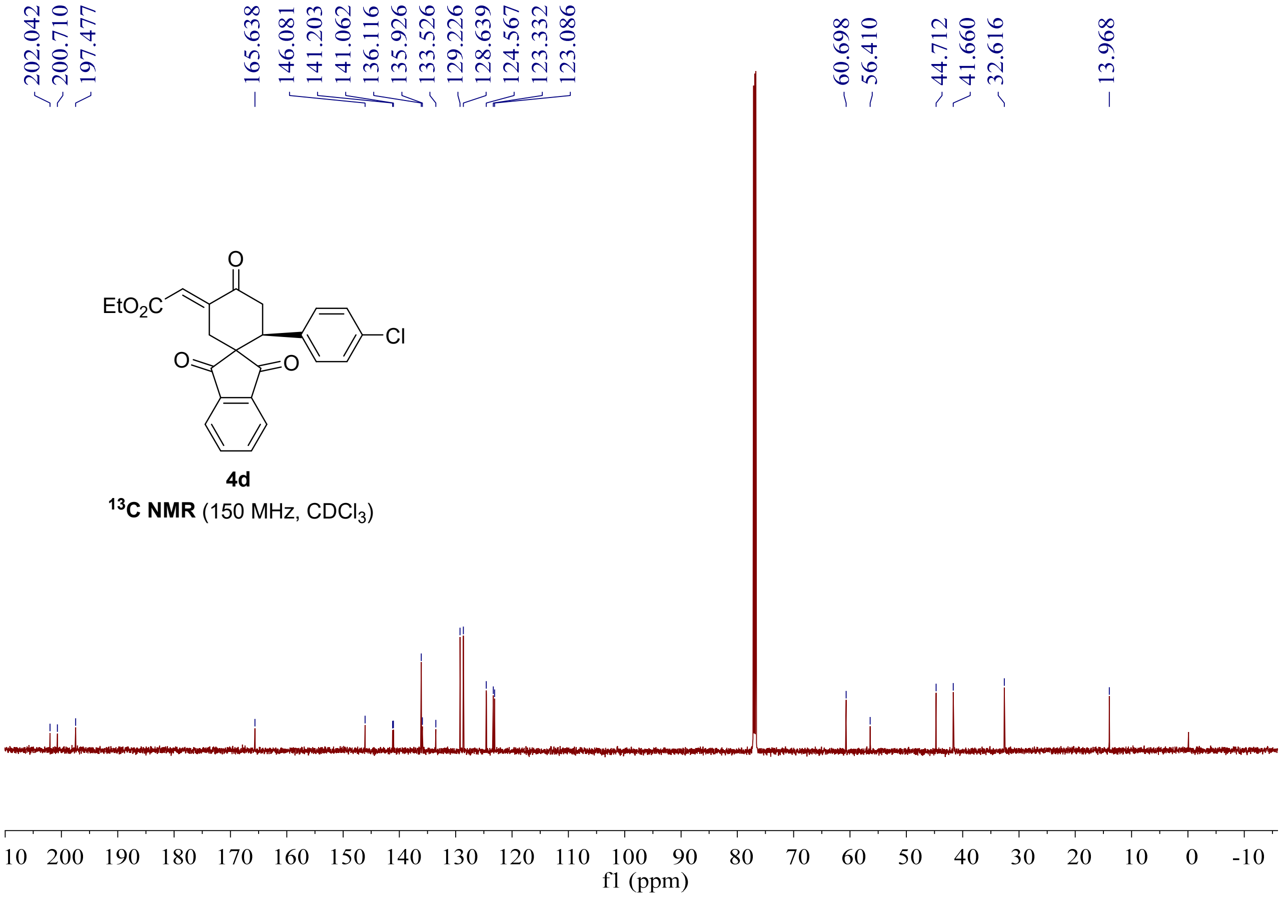
202.042 200.710 197.477 165.638 146.081 141.203 141.062 136.116 135.926 133.526 129.226 128.639 124.567 123.332 123.086 60.698 56.410 44.712 41.660 32.616 13.968
EtO2C
O
Cl
O
O
4d
13C NMR (150 MHz, CDCl3)
200 190 180 170 160 150 140 130 120 110 100 90 80 70 60 50 40 30 20 10 0 -10
f1 (ppm)

Daicel Chiral IA Column, (iPrOH/n-hexane = 40/60, 1.0 mL/min)

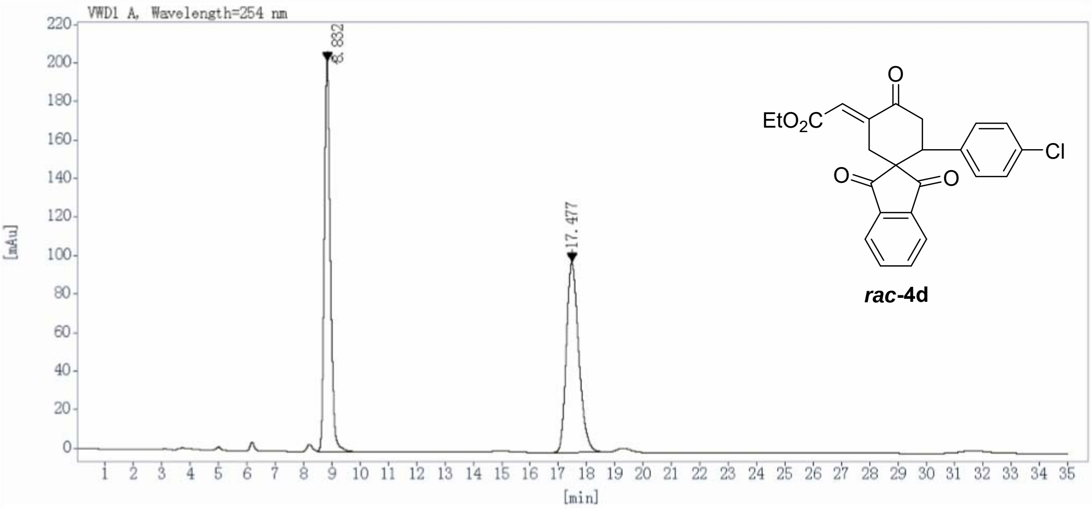

Ret Time [min]	Peak Type	Width [min]	Height [mAU]	Area [mAU*s]	Area [%]
8.832	FM	0.25	203.2294	3034.4146	50.1910
17.477	BB	0.47	98.9306	3011.3254	49.8090
			Totals:	6045.7400	100.0000

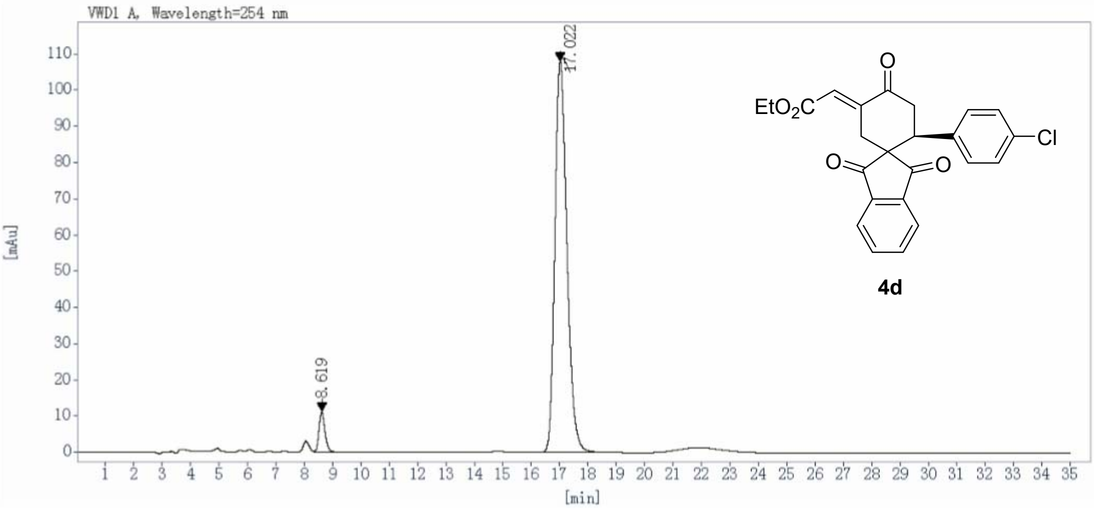

Ret Time [min]	Peak Type	Width [min]	Height [mAU]	Area [mAU*s]	Area [%]
8.619	VB	0.23	11.1887	166.2435	4.9656
17.022	BB	0.45	108.0145	3181.6421	95.0344
			Totals:	3347.8856	100.0000

HRMS (ESI-TOF) m/z: $[M + Na]^+$ Calcd for $C_{24}H_{19}O_5NaCl^+$ 445.0813 (^{35}Cl) and 447.0784 (^{37}Cl); Found 445.0812 (^{35}Cl) and 447.0792 (^{37}Cl).

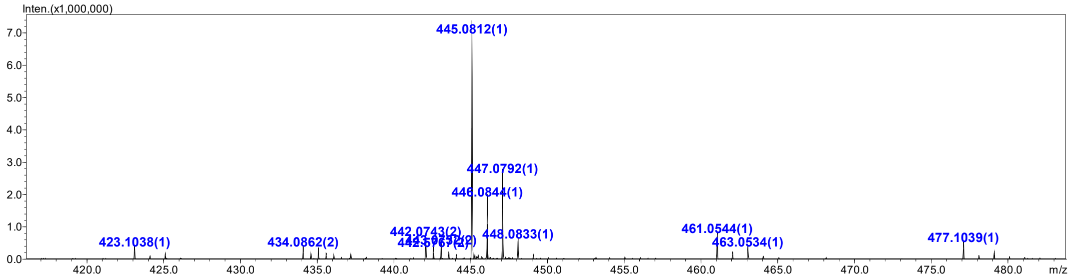

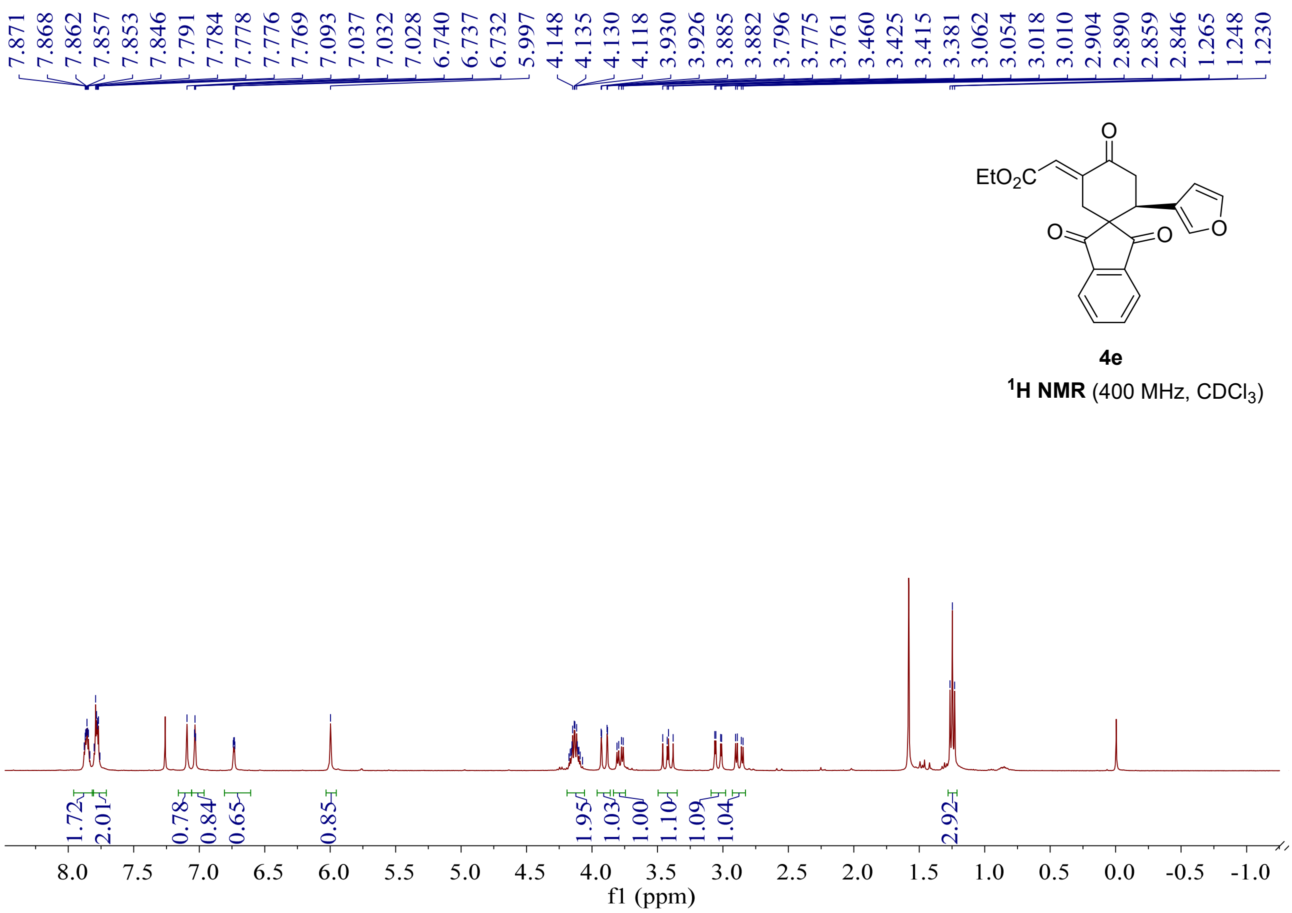
7.871 7.868 7.862 7.857 7.853 7.846 7.791 7.784 7.778 7.776 7.769 7.093 7.037 7.032 7.028 6.740 6.737 6.732 5.997 4.148 4.135 4.130 4.118 3.930 3.926 3.885 3.882 3.796 3.775 3.761 3.460 3.425 3.415 3.381 3.062 3.054 3.018 3.010 2.904 2.890 2.859 2.846 1.265 1.248 1.230
EtO2C
O
4e
1H NMR (400 MHz, CDCl3)
1.72 2.01 0.78 0.84 0.65 0.85 1.95 1.03 1.00 1.10 1.09 1.04 2.92
8.0 7.5 7.0 6.5 6.0 5.5 5.0 4.5 4.0 3.5 3.0 2.5 2.0 1.5 1.0 0.5 0.0 -0.5 -1.0
f1 (ppm)

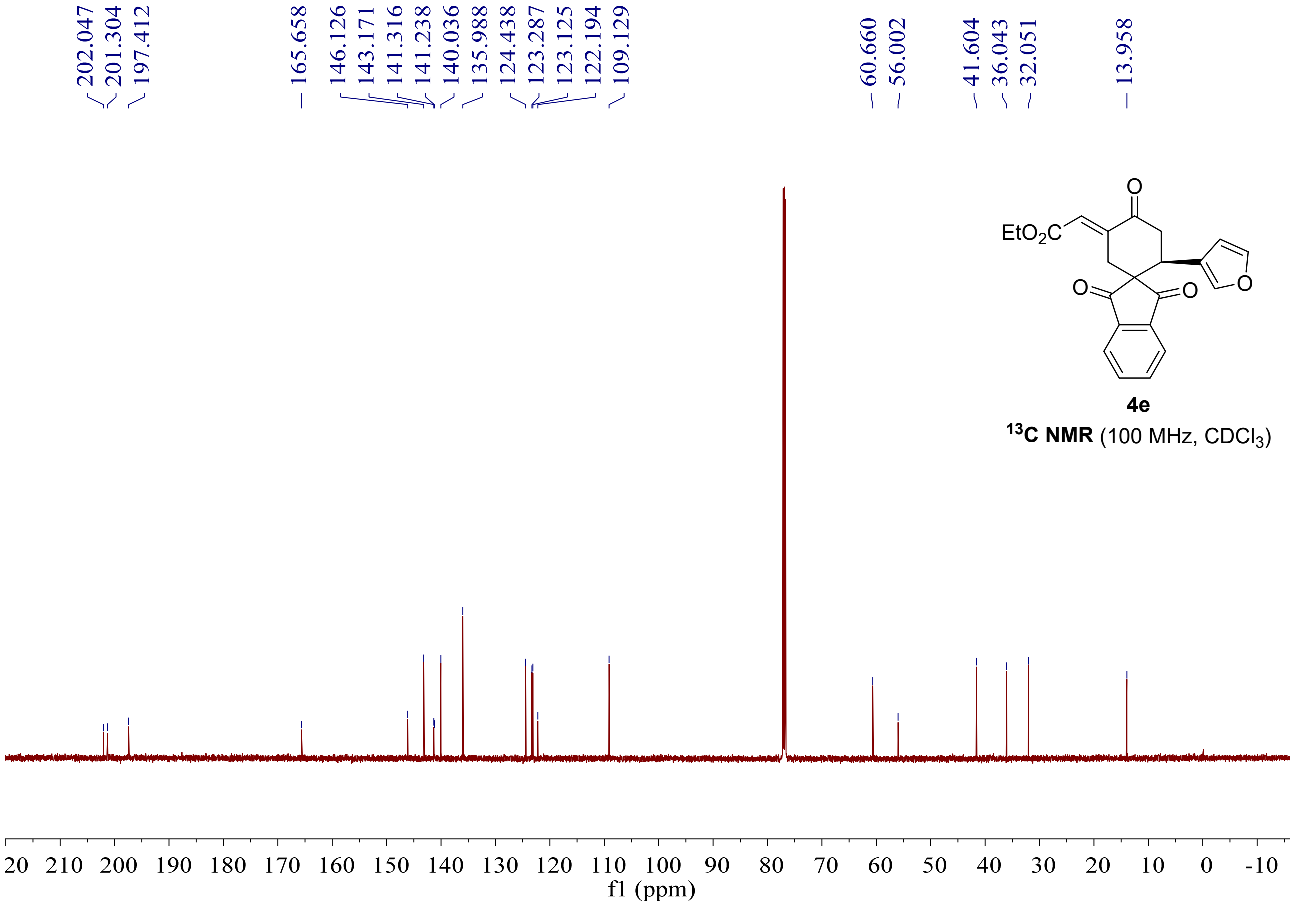
202.047 201.304 197.412 165.658 146.126 143.171 141.316 141.238 140.036 135.988 124.438 123.287 123.125 122.194 109.129 60.660 56.002 41.604 36.043 32.051 13.958
EtO2C
O
4e
13C NMR (100 MHz, CDCl3)
20 210 200 190 180 170 160 150 140 130 120 110 100 90 80 70 60 50 40 30 20 10 0 -10
f1 (ppm)

Daicel Chiral IE Column, (*i*PrOH/*n*-hexane = 40/60, 1.0 mL/min)

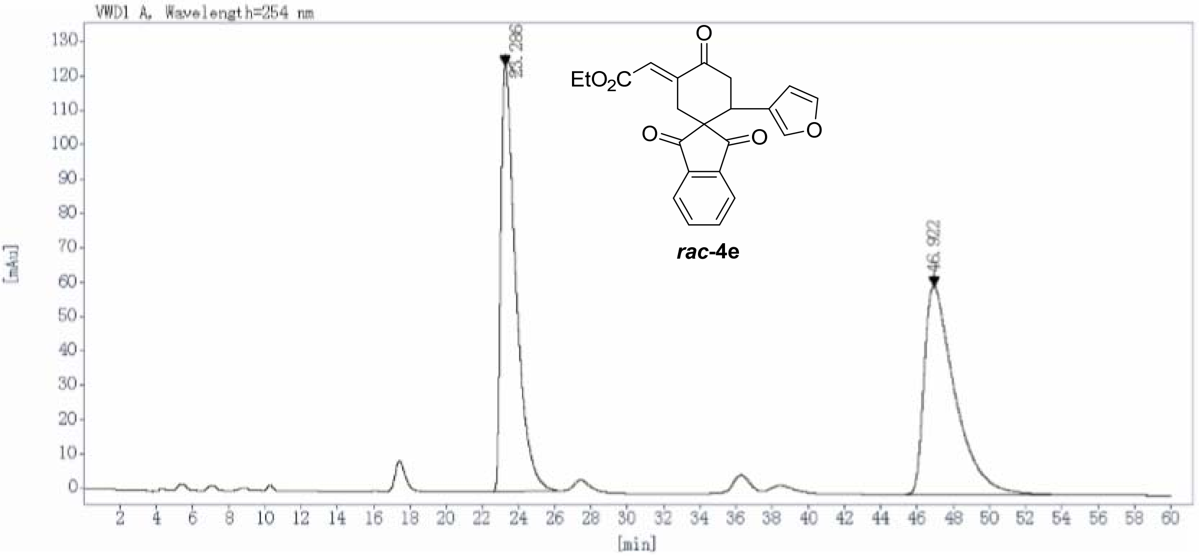

Ret Time [min]	Peak Type	Width [min]	Height [mAU]	Area [mAU*s]	Area [%]
23.286	BB	0.86	124.1702	7191.7593	50.0355
46.922	BB	1.72	60.6972	7181.5435	49.9645
			Totals:	14373.3027	100.0000

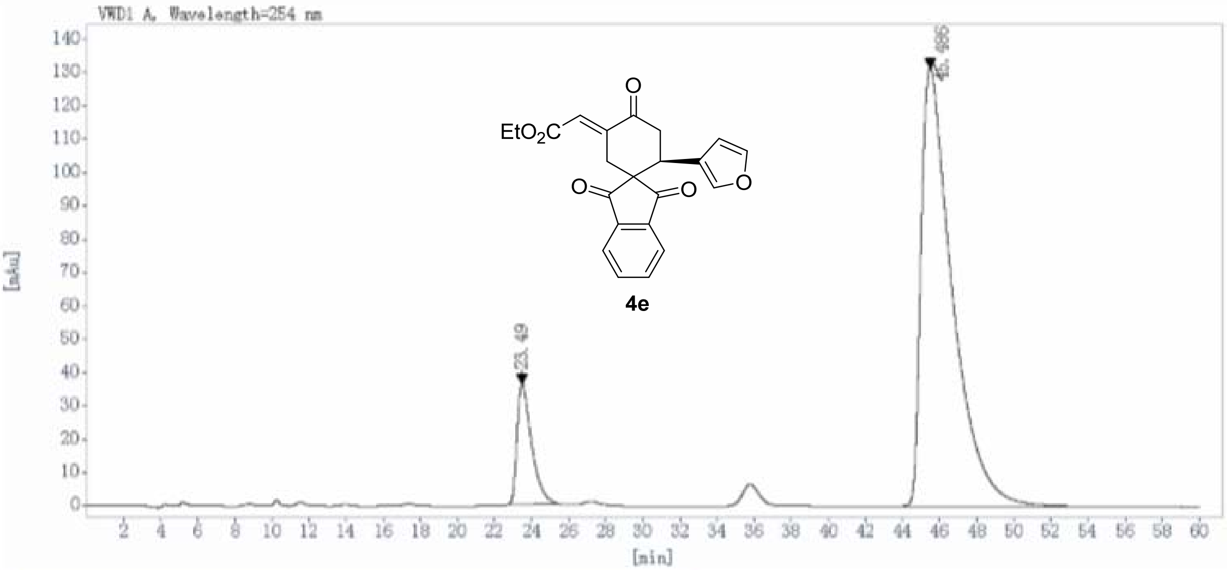

Ret Time [min]	Peak Type	Width [min]	Height [mAU]	Area [mAU*s]	Area [%]
23.490	MM	0.92	36.1954	1993.6246	11.4641
45.486	BB	1.71	131.5221	15396.5537	88.5359
			Totals:	17390.1783	100.0000

HRMS (ESI-TOF) m/z: [M + Na]$^+$ Calcd for $C_{22}H_{18}NaO_6^+$ 401.0996; Found 401.0996.

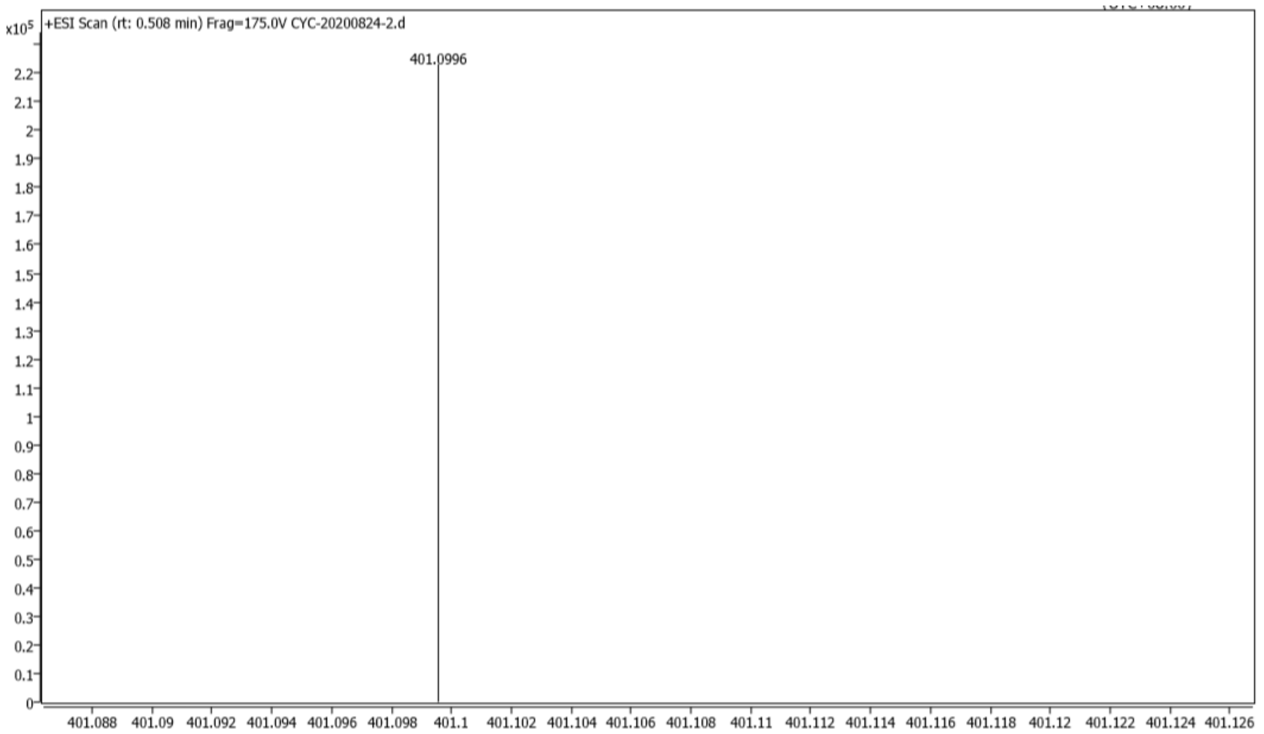

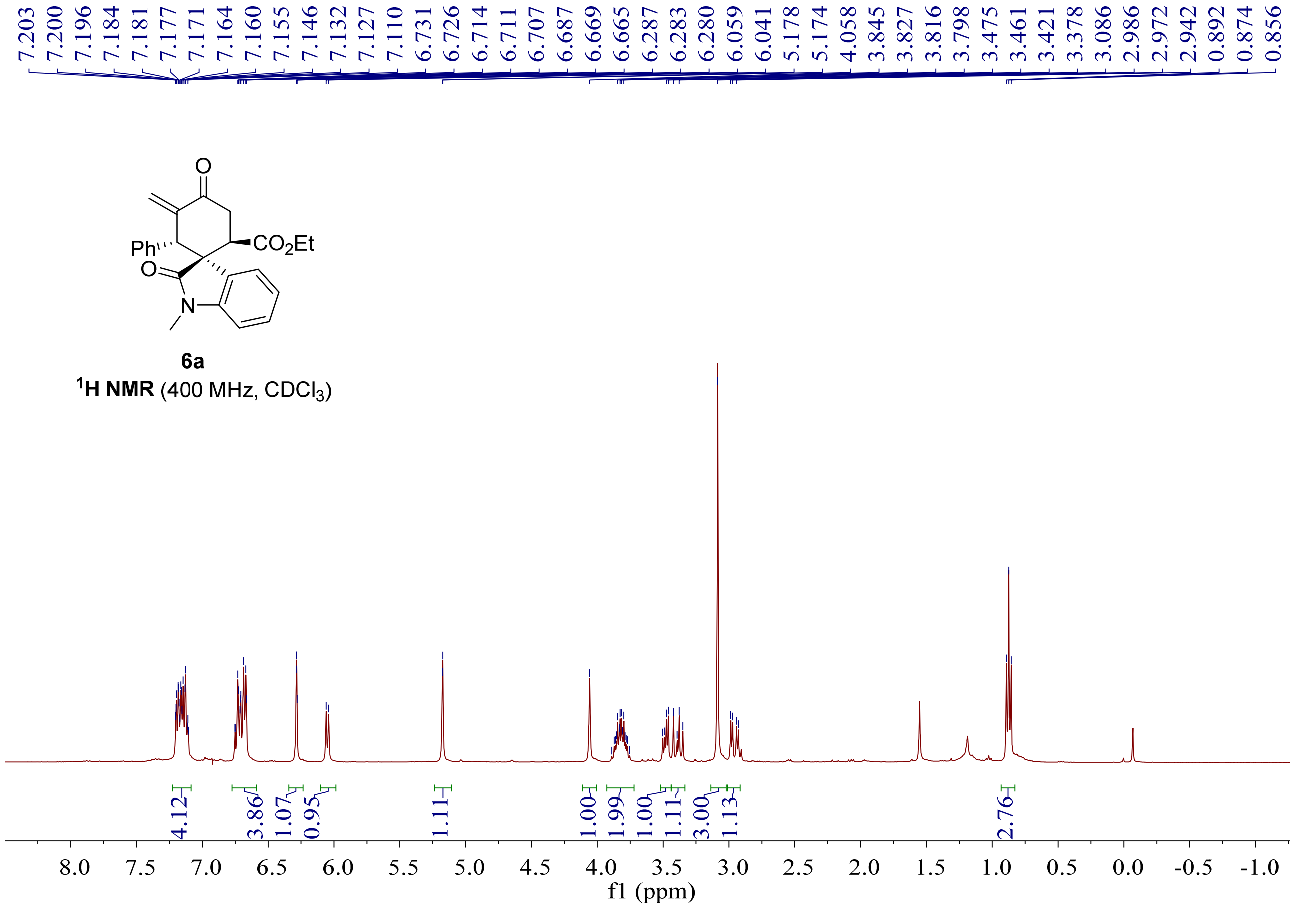
7.203
7.200
7.196
7.184
7.181
7.177
7.171
7.164
7.160
7.155
7.146
7.132
7.127
7.110
6.731
6.726
6.714
6.711
6.707
6.687
6.669
6.665
6.287
6.283
6.280
6.059
6.041
5.178
5.174
4.058
3.845
3.827
3.816
3.798
3.475
3.461
3.421
3.378
3.086
2.986
2.972
2.942
0.892
0.874
0.856
O
Ph
CO2Et
O
N
6a
1H NMR (400 MHz, CDCl3)
4.12
3.86
1.07
0.95
1.11
1.00
1.99
1.00
1.11
3.00
1.13
2.76
8.0 7.5 7.0 6.5 6.0 5.5 5.0 4.5 4.0 3.5 3.0 2.5 2.0 1.5 1.0 0.5 0.0 -0.5 -1.0
f1 (ppm)

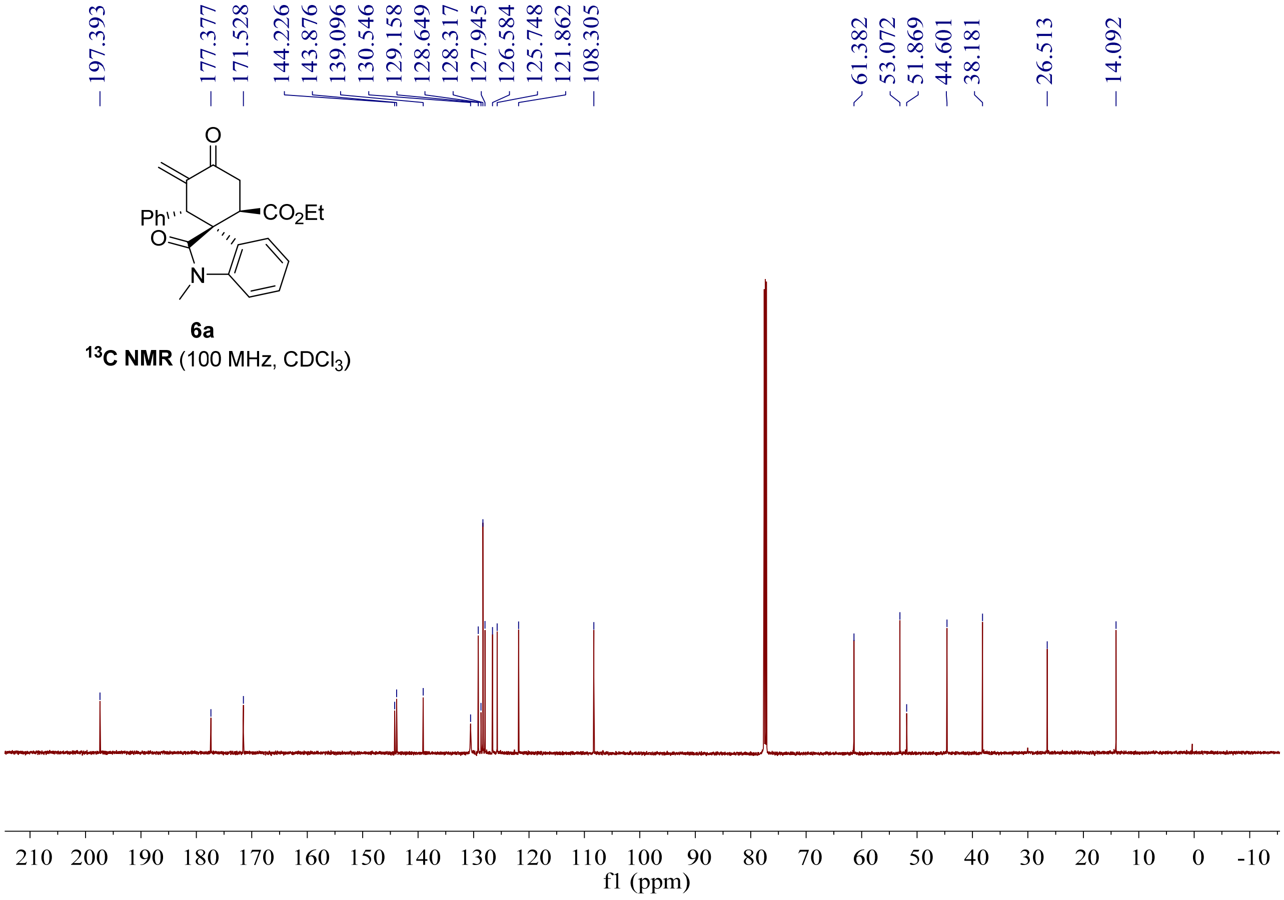
197.393
177.377
171.528
144.226
143.876
139.096
130.546
129.158
128.649
128.317
127.945
126.584
125.748
121.862
108.305
61.382
53.072
51.869
44.601
38.181
26.513
14.092
O
Ph
CO2Et
O
N
6a
13C NMR (100 MHz, CDCl3)
210 200 190 180 170 160 150 140 130 120 110 100 90 80 70 60 50 40 30 20 10 0 -10
f1 (ppm)

Daicel Chiral IE Column, (*i*PrOH/*n*-hexane = 40/60, 1.0 mL/min)

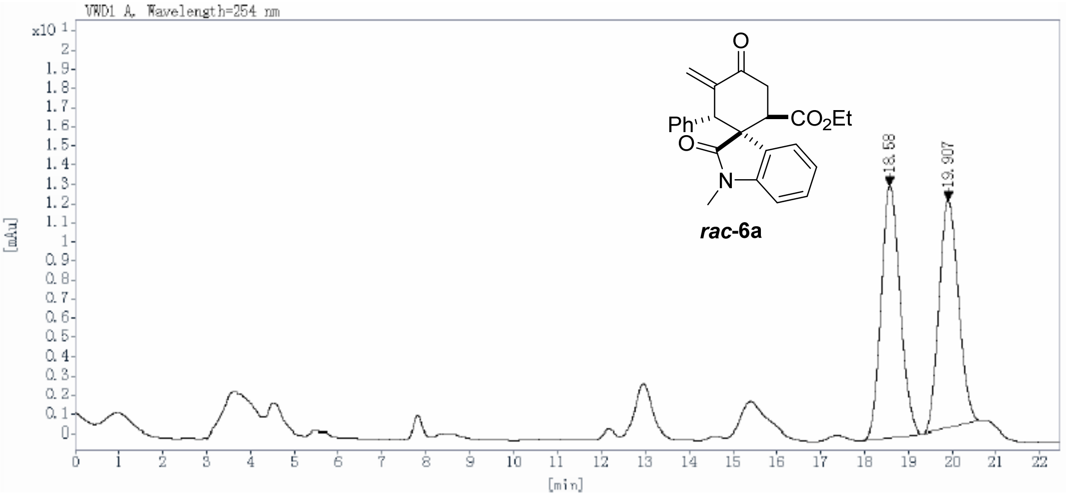

Ret Time [min]	Peak Type	Width [min]	Height [mAU]	Area [mAU*s]	Area [%]
18.580	BB	0.46	13.1198	392.3362	51.8751
19.907	BB S	0.48	11.8742	363.9729	48.1249
			Totals:	756.3091	100.0000

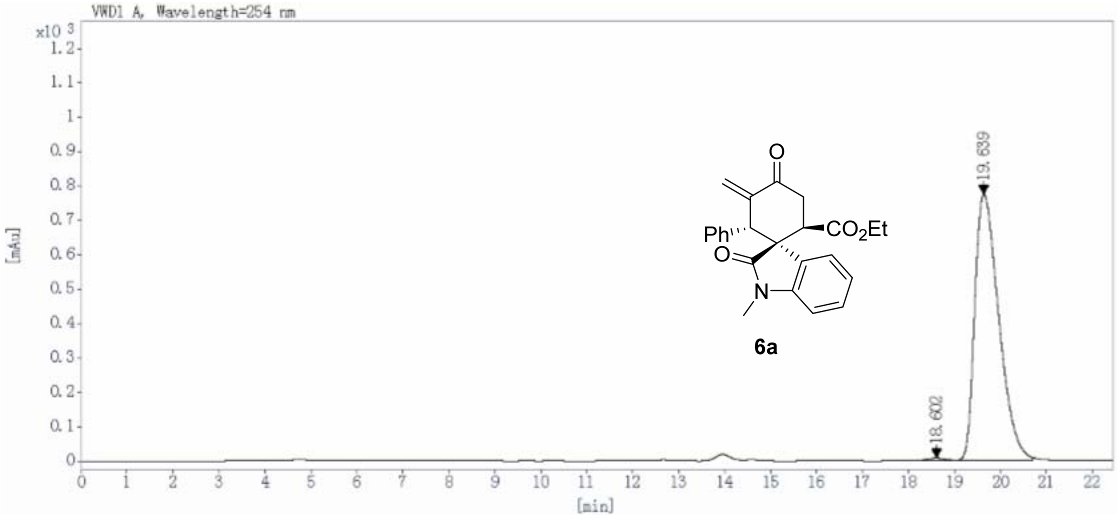

Ret Time [min]	Peak Type	Width [min]	Height [mAU]	Area [mAU*s]	Area [%]
18.602	BV	0.43	7.7711	212.3712	0.7481
19.639	VBAS	0.56	775.9315	28174.2949	99.2519
			Totals:	28386.6661	100.0000

HRMS (ESI-TOF) m/z: $[M + Na]^+$ Calcd for $C_{24}H_{23}O_4NNa^+$ 412.1519; Found 412.1526.

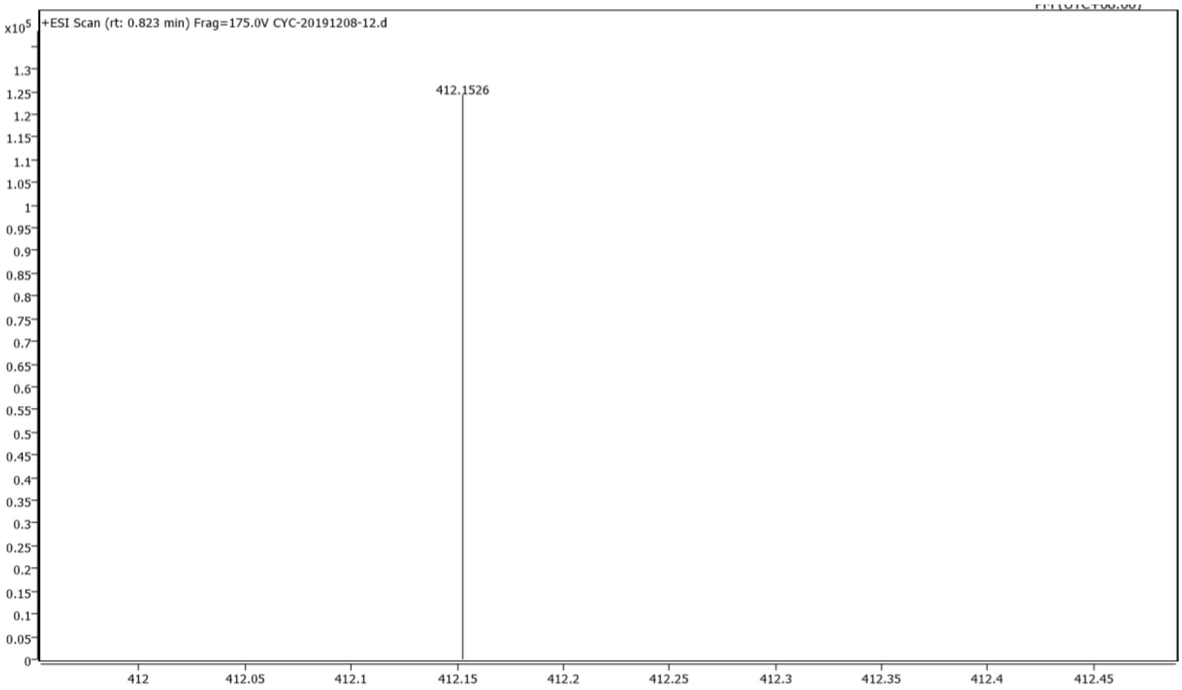

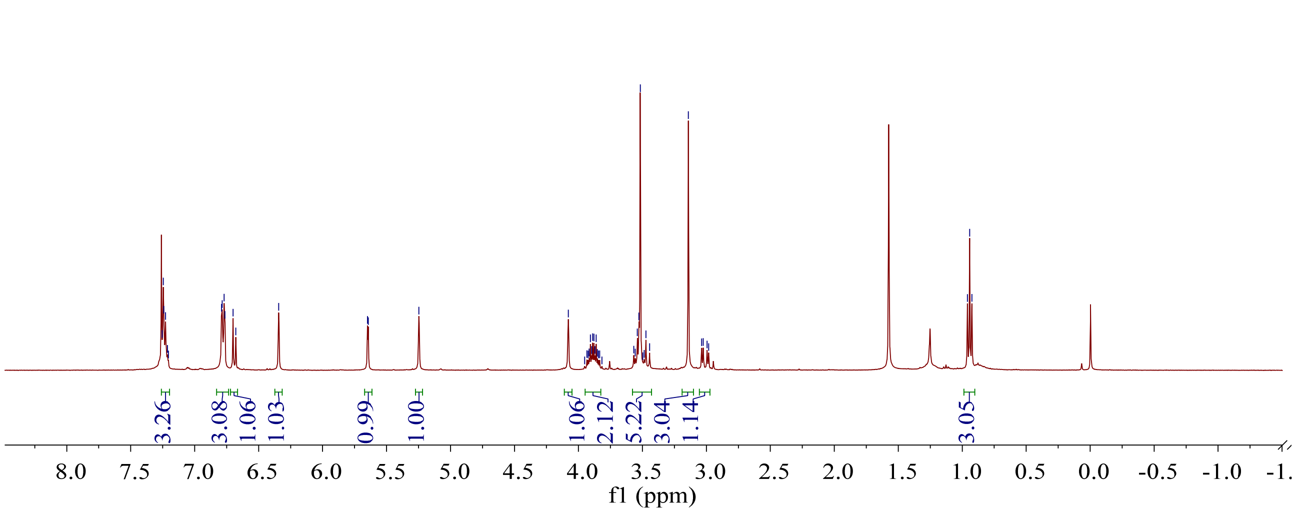

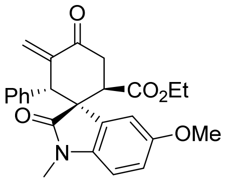

6b

¹H NMR (400 MHz, $CDCl_3$)

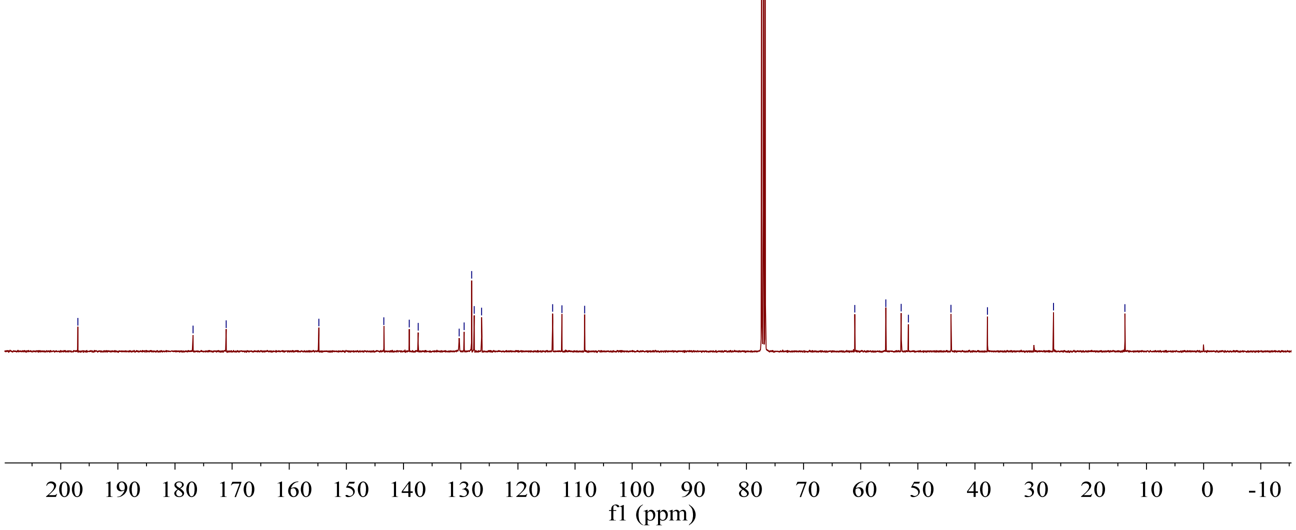

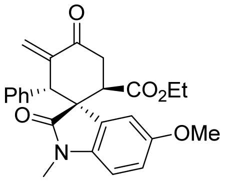

6b

¹³C NMR (100 MHz, $CDCl_3$)

Daicel Chiral IE Column, *i*PrOH/*n*-hexane = 40/60, 1.0 mL/min

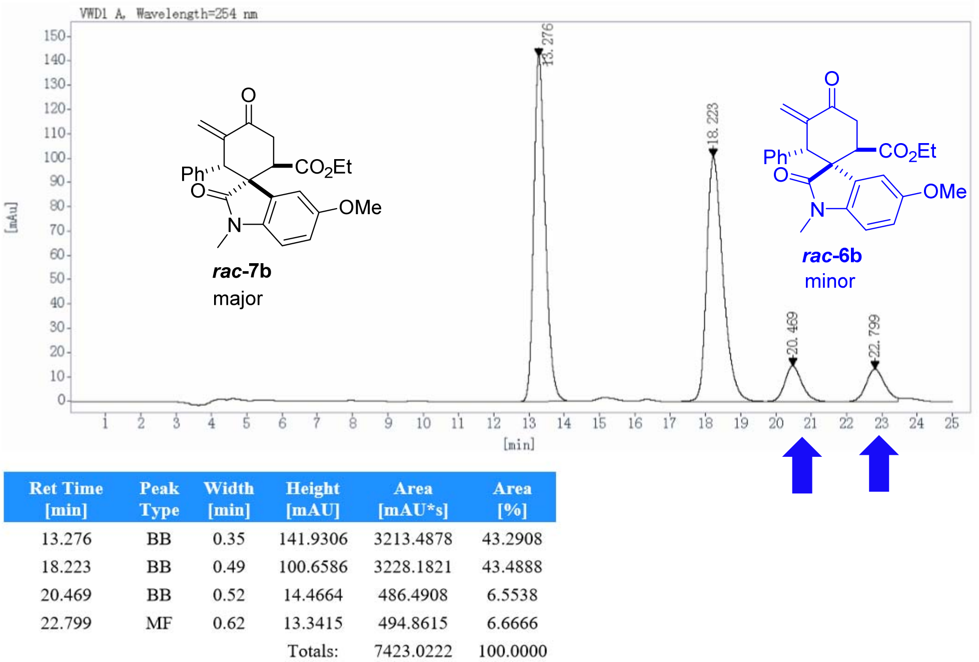

Ret Time [min]	Peak Type	Width [min]	Height [mAU]	Area [mAU*s]	Area [%]
13.276	BB	0.35	141.9306	3213.4878	43.2908
18.223	BB	0.49	100.6586	3228.1821	43.4888
20.469	BB	0.52	14.4664	486.4908	6.5538
22.799	MF	0.62	13.3415	494.8615	6.6666
			Totals:	7423.0222	100.0000

By using Pd(OAc)$_2$, triphenyl phosphite and TBAB system, *rac*-6b was formed as the minor diastereomer, whereas using Pd(OAc)$_2$, L8 and chiral C4 gave 6b as the major diastereomer. The mixture of diastereomers were tested.

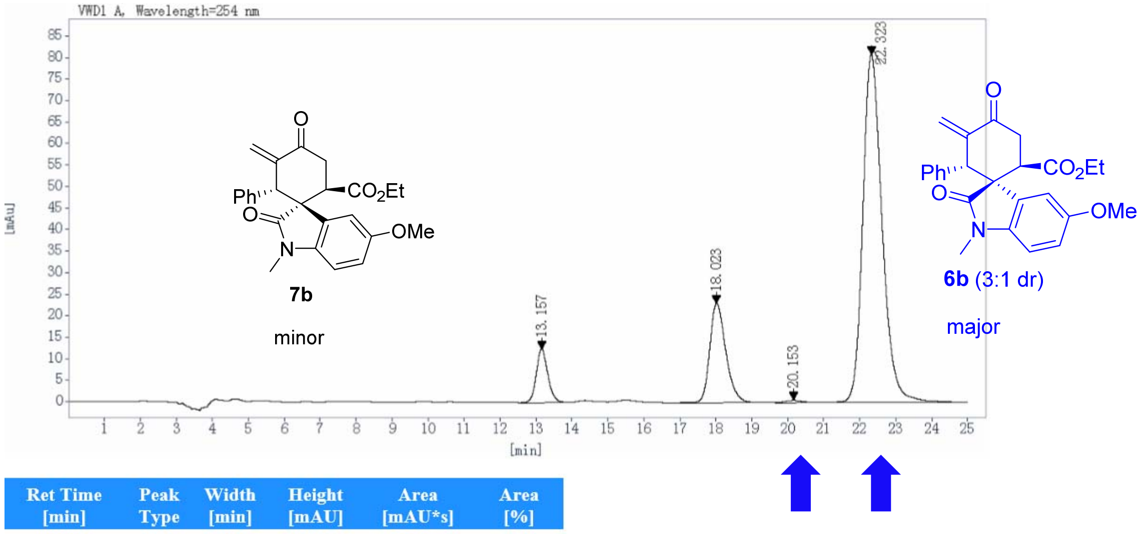

Ret Time [min]	Peak Type	Width [min]	Height [mAU]	Area [mAU*s]	Area [%]
13.157	BB	0.34	12.4331	276.1911	6.8660
18.023	BB	0.47	23.1510	718.1597	17.8531
20.153	MM	0.55	0.5738	18.9559	0.4712
22.323	BBA	0.57	80.8123	3009.2878	74.8096
			Totals:	4022.5946	100.0000

HRMS (ESI-TOF) m/z: $[M + Na]^+$ Calcd for $C_{25}H_{25}O_5NNa^+$ 442.1625; Found 442.1623.

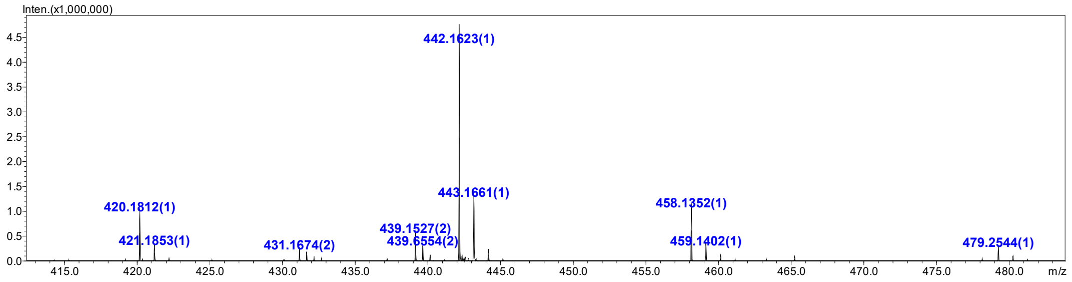

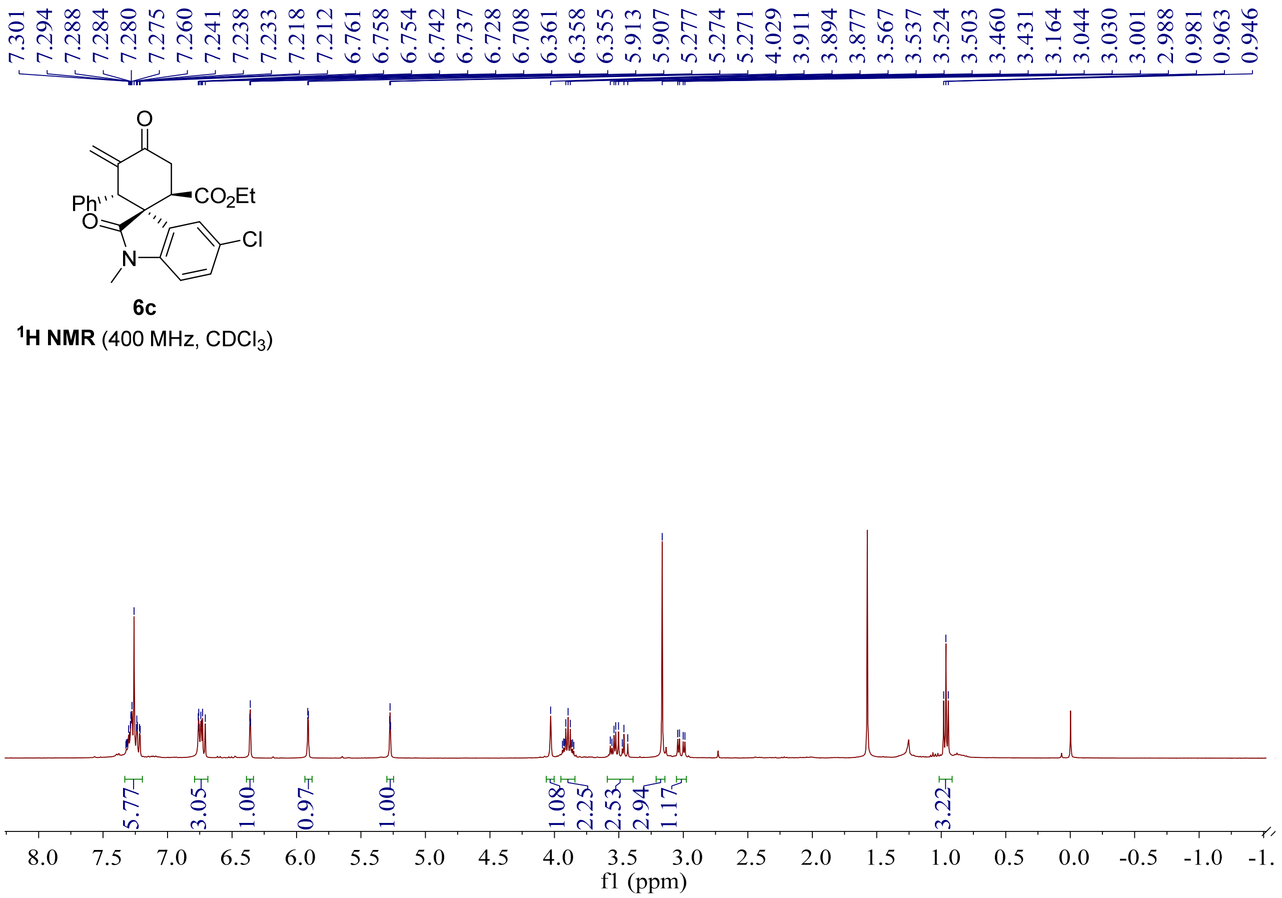
7.301 7.294 7.288 7.284 7.280 7.275 7.260 7.241 7.238 7.233 7.218 7.212 6.761 6.758 6.754 6.742 6.737 6.728 6.708 6.361 6.358 6.355 5.913 5.907 5.277 5.274 5.271 4.029 3.911 3.894 3.877 3.567 3.537 3.524 3.503 3.460 3.431 3.164 3.044 3.030 3.001 2.988 0.981 0.963 0.946
O
Ph
CO2Et
O
N
Cl
6c
1H NMR (400 MHz, CDCl3)
5.77 3.05 1.00 0.97 1.00 1.08 2.25 2.53 2.94 1.17 3.22
8.0 7.5 7.0 6.5 6.0 5.5 5.0 4.5 4.0 3.5 3.0 2.5 2.0 1.5 1.0 0.5 0.0 -0.5 -1.0
f1 (ppm)

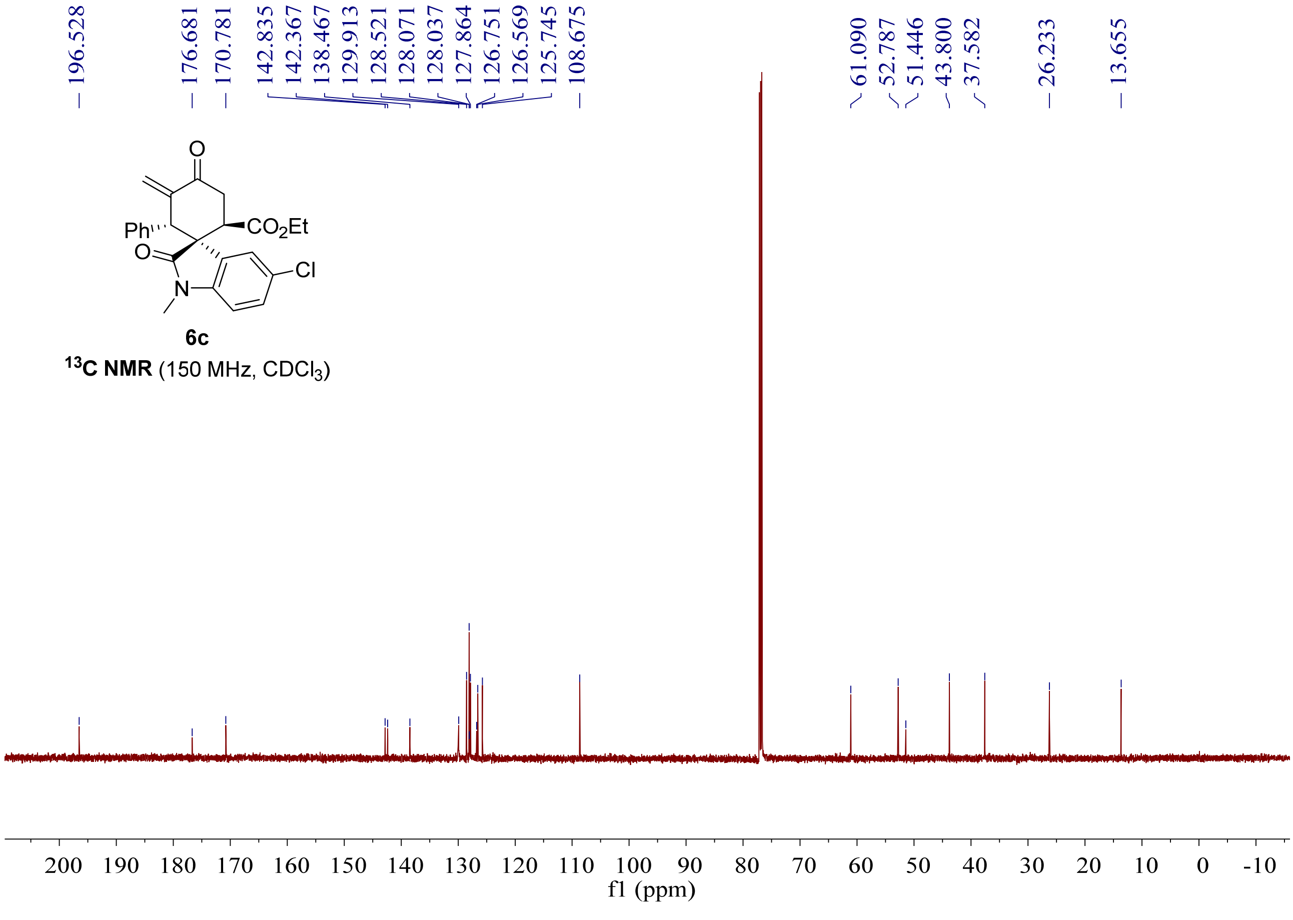
196.528 176.681 170.781 142.835 142.367 138.467 129.913 128.521 128.071 128.037 127.864 126.751 126.569 125.745 108.675 61.090 52.787 51.446 43.800 37.582 26.233 13.655
O
Ph
CO2Et
O
N
Cl
6c
13C NMR (150 MHz, CDCl3)
200 190 180 170 160 150 140 130 120 110 100 90 80 70 60 50 40 30 20 10 0 -10
f1 (ppm)

Daicel Chiral IE Column, *i*PrOH/*n*-hexane = 40/60, 1.0 mL/min.

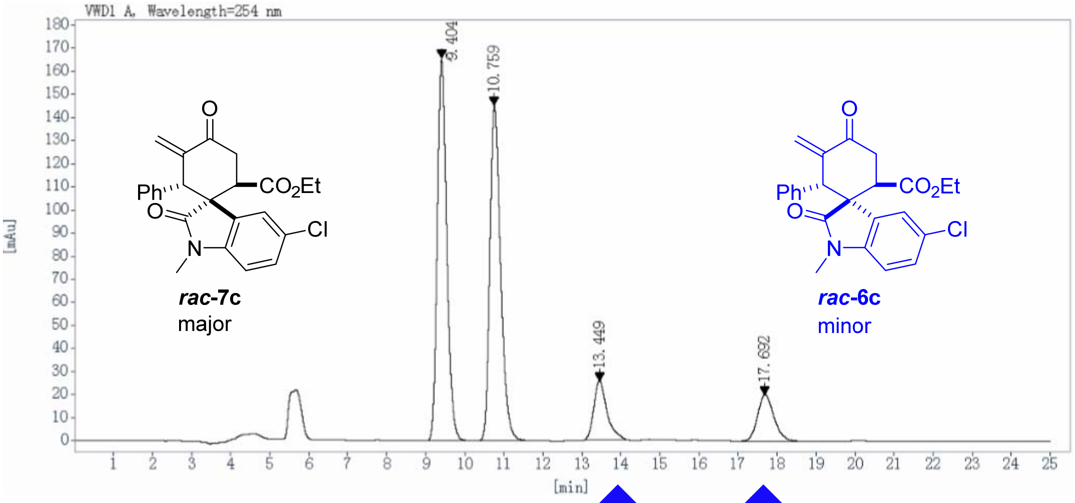

Ret Time [min]	Peak Type	Width [min]	Height [mAU]	Area [mAU*s]	Area [%]
9.404	BB	0.26	165.7225	2779.1226	41.2923
10.759	BB	0.29	145.1131	2773.0681	41.2024
13.449	MM	0.39	25.3899	591.8856	8.7943
17.692	BB	0.45	20.1106	586.2861	8.7111
			Totals:	6730.3624	100.0000

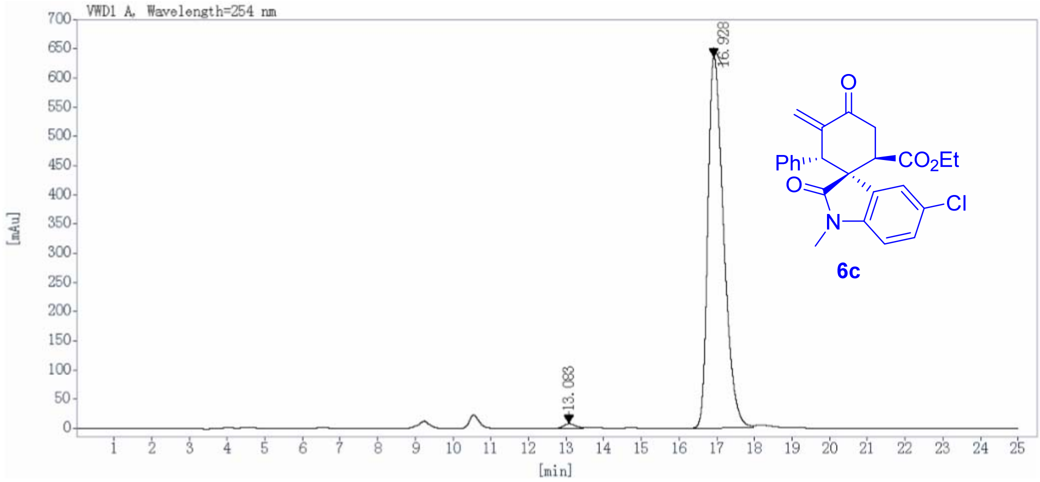

Ret Time [min]	Peak Type	Width [min]	Height [mAU]	Area [mAU*s]	Area [%]
13.083	BV	0.32	8.2050	175.2725	0.9381
16.928	MF	0.49	635.6058	18507.6309	99.0619
			Totals:	18682.9034	100.0000

HRMS (ESI-TOF) m/z: $[M + Na]^+$ Calcd for $C_{24}H_{22}O_4NClNa^+$ 446.1130 (^{35}Cl) and 448.1100 (^{37}Cl); Found 446.1126 (^{35}Cl) and 448.1102 (^{37}Cl).

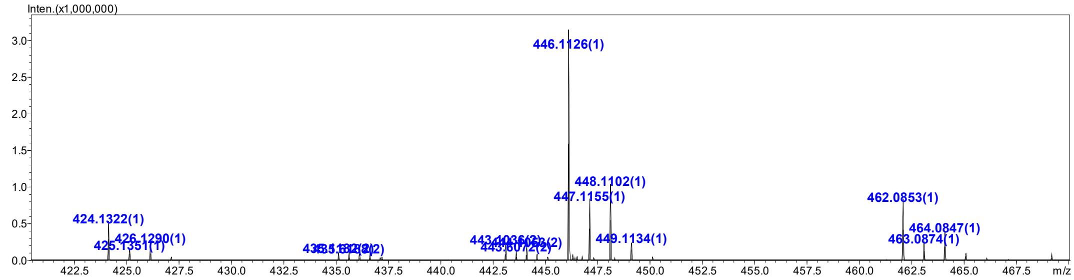

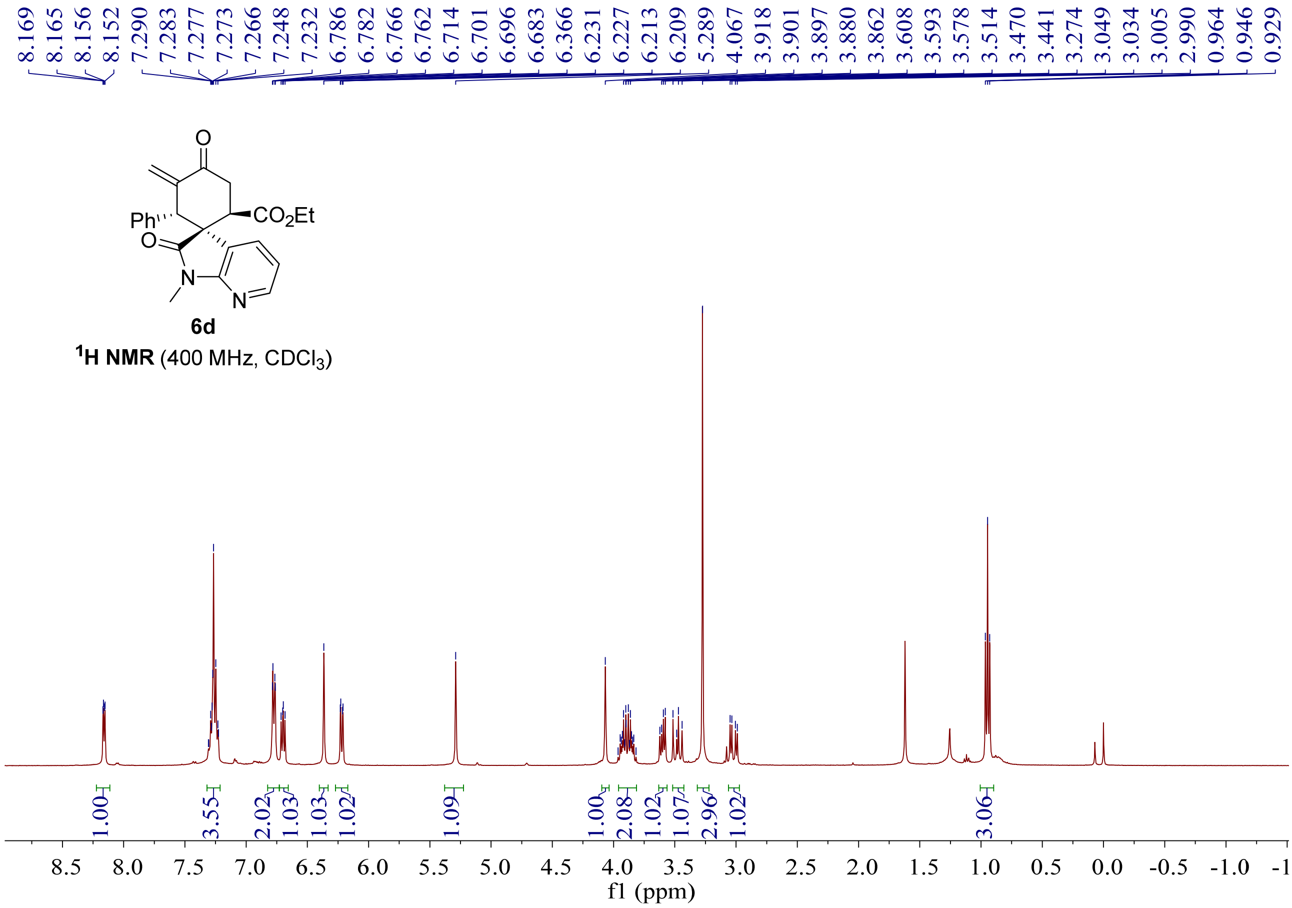
8.169
8.165
8.156
8.152
7.290
7.283
7.277
7.273
7.266
7.248
7.232
6.786
6.782
6.766
6.762
6.714
6.701
6.696
6.683
6.366
6.231
6.227
6.213
6.209
5.289
4.067
3.918
3.901
3.897
3.880
3.862
3.608
3.593
3.578
3.514
3.470
3.441
3.274
3.049
3.034
3.005
2.990
0.964
0.946
0.929
O
Ph
CO_2Et
O
N
N
6d
^{1}H NMR (400 MHz, $CDCl_3$)
1.00
3.55
2.02
1.03
1.03
1.02
1.09
1.00
2.08
1.02
1.07
2.96
1.02
3.06
8.5 8.0 7.5 7.0 6.5 6.0 5.5 5.0 4.5 4.0 3.5 3.0 2.5 2.0 1.5 1.0 0.5 0.0 -0.5 -1.0 -1
f1 (ppm)

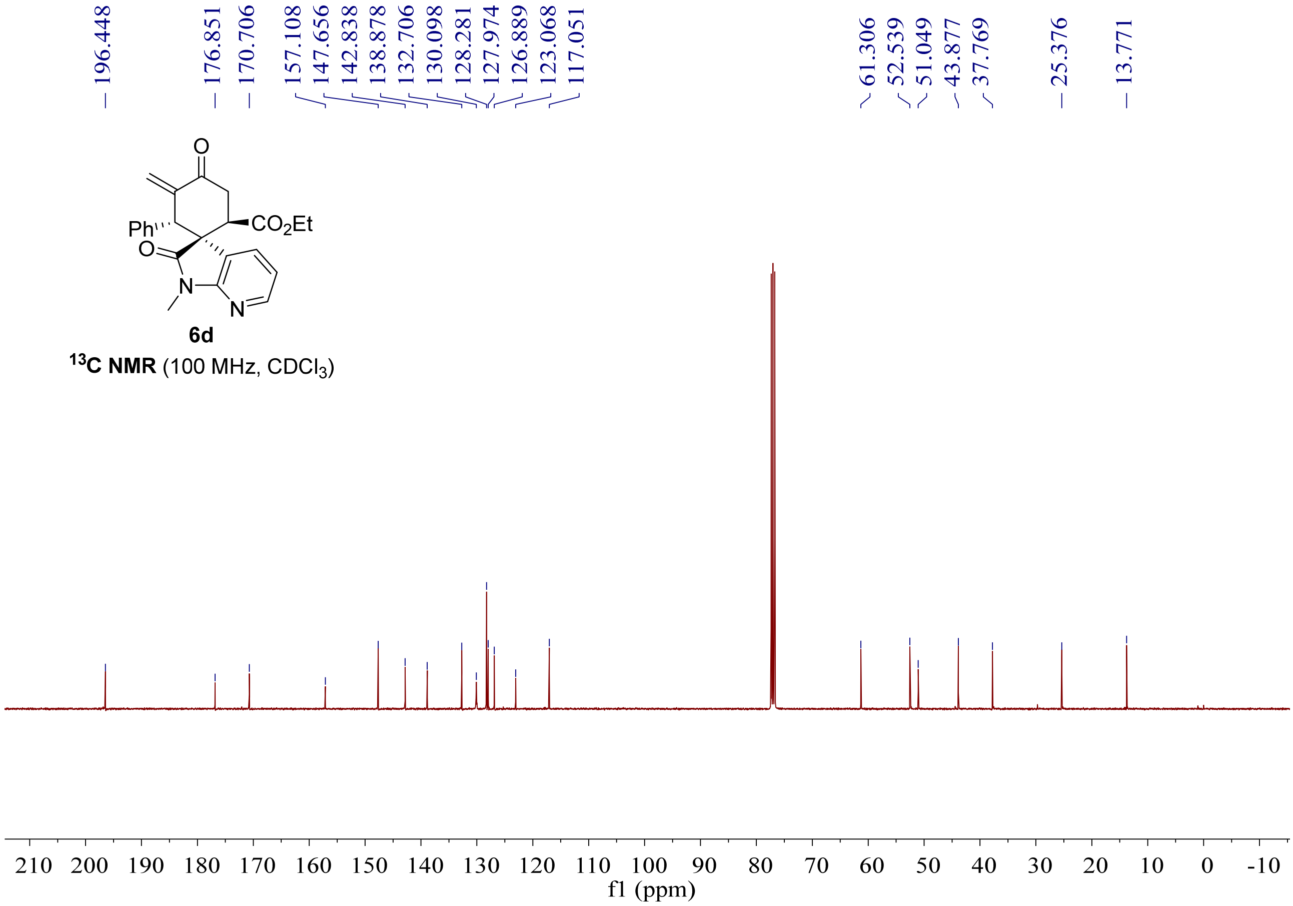
196.448
176.851
170.706
157.108
147.656
142.838
138.878
132.706
130.098
128.281
127.974
126.889
123.068
117.051
61.306
52.539
51.049
43.877
37.769
25.376
13.771
O
Ph
CO_2Et
O
N
N
6d
^{13}C NMR (100 MHz, $CDCl_3$)
210 200 190 180 170 160 150 140 130 120 110 100 90 80 70 60 50 40 30 20 10 0 -10
f1 (ppm)

Daicel Chiral IE Column, *i*PrOH/*n*-hexane = 40/60, 1.0 mL/min.

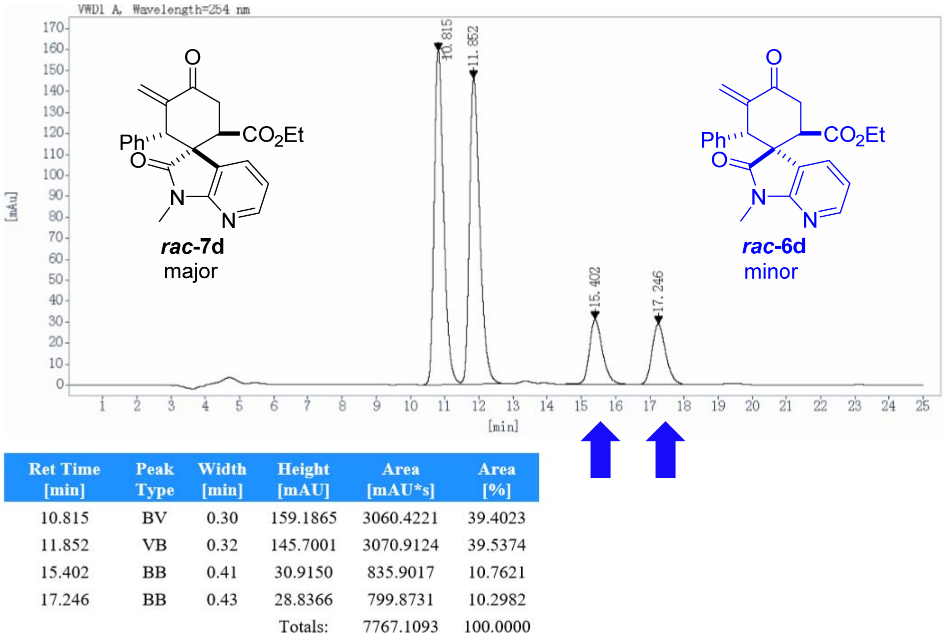

Ret Time [min]	Peak Type	Width [min]	Height [mAU]	Area [mAU*s]	Area [%]
10.815	BV	0.30	159.1865	3060.4221	39.4023
11.852	VB	0.32	145.7001	3070.9124	39.5374
15.402	BB	0.41	30.9150	835.9017	10.7621
17.246	BB	0.43	28.8366	799.8731	10.2982
			Totals:	7767.1093	100.0000

By using Pd(OAc)$_2$, triphenyl phosphite and TBAB system, *rac*-6d was formed as the minor diastereomer, whereas using Pd(OAc)$_2$, L8 and chiral C4 gave 6d as the major diastereomer. The mixture of diastereomers were tested.

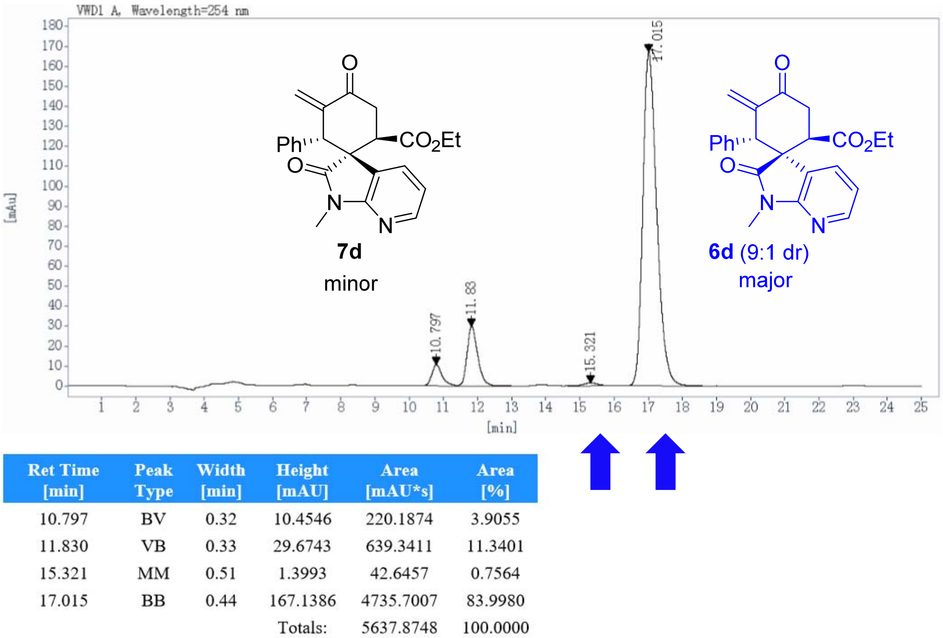

Ret Time [min]	Peak Type	Width [min]	Height [mAU]	Area [mAU*s]	Area [%]
10.797	BV	0.32	10.4546	220.1874	3.9055
11.830	VB	0.33	29.6743	639.3411	11.3401
15.321	MM	0.51	1.3993	42.6457	0.7564
17.015	BB	0.44	167.1386	4735.7007	83.9980
			Totals:	5637.8748	100.0000

HRMS (ESI-TOF) m/z: $[M + Na]^+$ Calcd for $C_{23}H_{22}O_4N_2Na^+$ 413.1472; Found 413.1466.

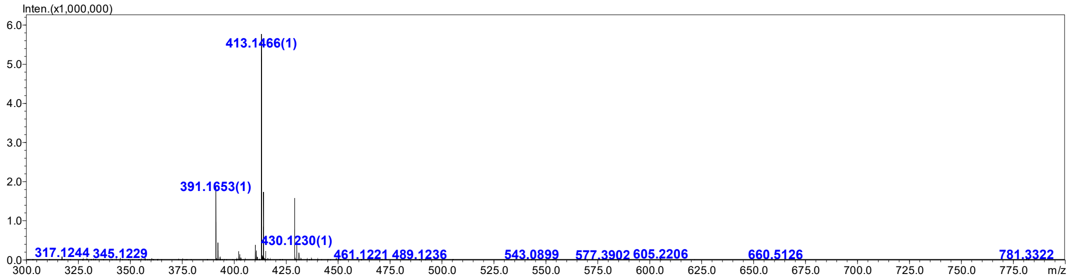

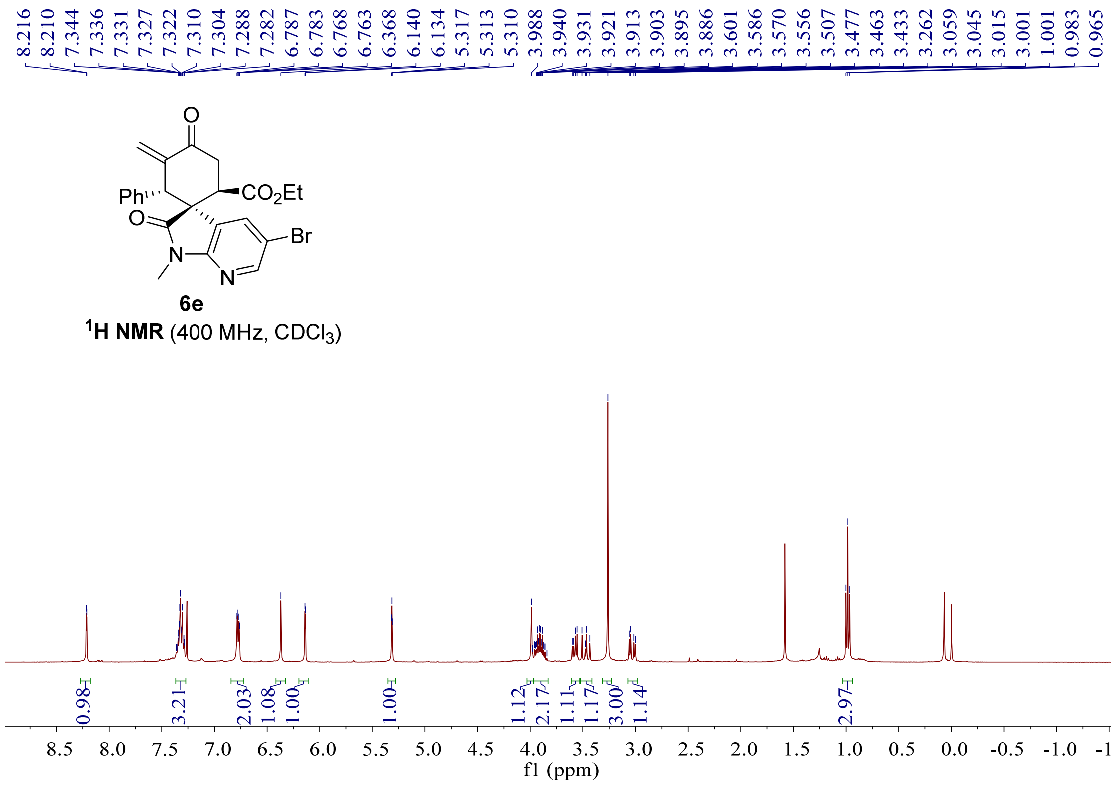
8.216
8.210
7.344
7.336
7.331
7.327
7.322
7.310
7.304
7.288
7.282
6.787
6.783
6.768
6.763
6.368
6.140
6.134
5.317
5.313
5.310
3.988
3.940
3.931
3.921
3.913
3.903
3.895
3.886
3.601
3.586
3.570
3.556
3.507
3.477
3.463
3.433
3.262
3.059
3.045
3.015
3.001
1.001
0.983
0.965
Ph
CO2Et
O
N
Br
6e
1H NMR (400 MHz, CDCl3)
0.98
3.21
2.03
1.08
1.00
1.00
1.12
2.17
1.11
1.17
3.00
1.14
2.97
f1 (ppm)

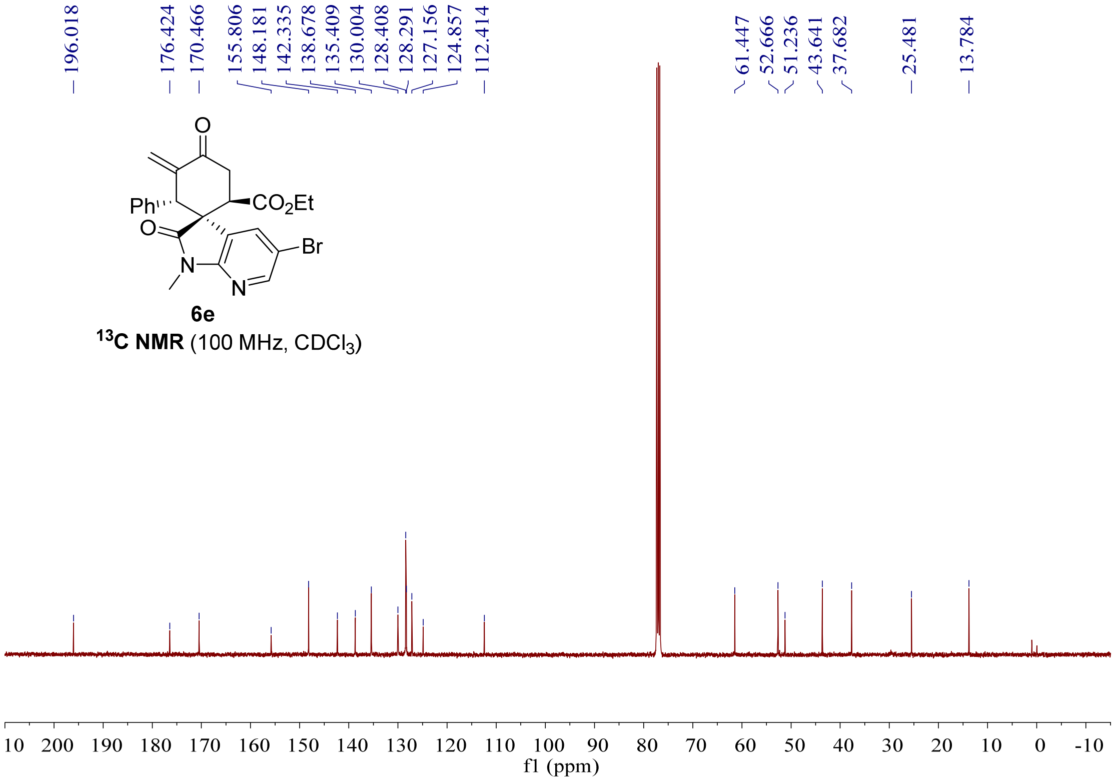
196.018
176.424
170.466
155.806
148.181
142.335
138.678
135.409
130.004
128.408
128.291
127.156
124.857
112.414
61.447
52.666
51.236
43.641
37.682
25.481
13.784
Ph
CO2Et
O
N
Br
6e
13C NMR (100 MHz, CDCl3)
f1 (ppm)

Daicel Chiral IE Column, *i*PrOH/*n*-hexane = 40/60, 1.0 mL/min, λ = 254 nm,

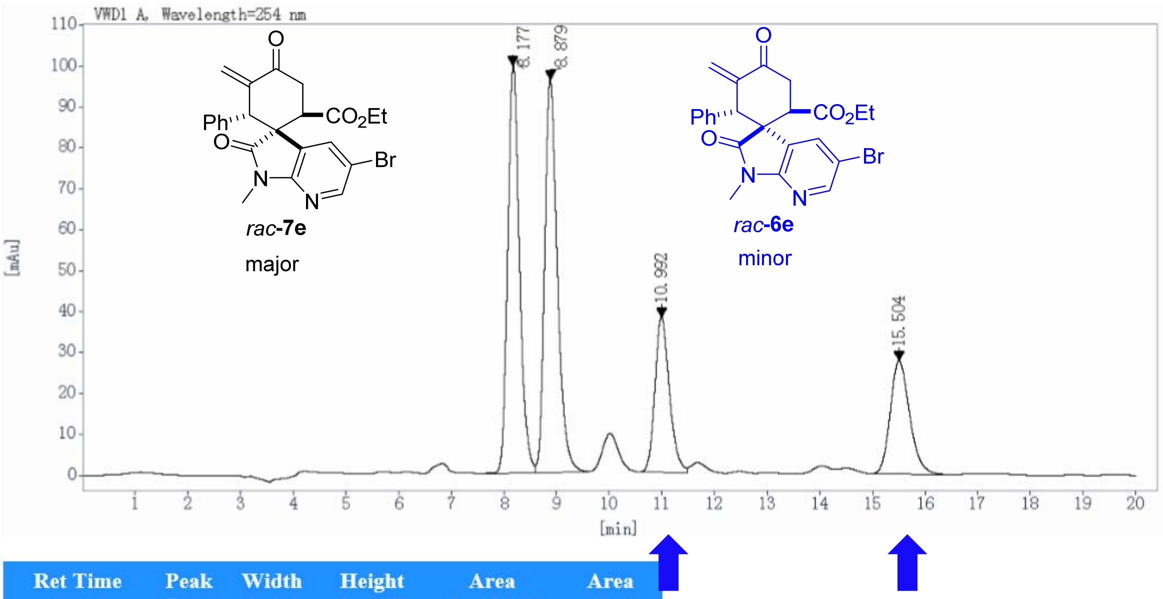

Ret Time [min]	Peak Type	Width [min]	Height [mAU]	Area [mAU*s]	Area [%]
8.177	BV	0.24	99.5759	1547.4913	33.7796
8.879	VB	0.26	95.9335	1601.6337	34.9614
10.992	MF	0.32	37.8753	721.2298	15.7434
15.504	BB	0.39	27.7685	710.7916	15.5156
			Totals:	4581.1464	100.0000

By using Pd(OAc)$_2$, triphenyl phosphite and TBAB system, *rac*-6e was formed as the minor diastereomer, whereas using Pd(OAc)$_2$, L8 and chiral C4 gave 6e as the major diastereomer. The mixture of diastereomers were tested.

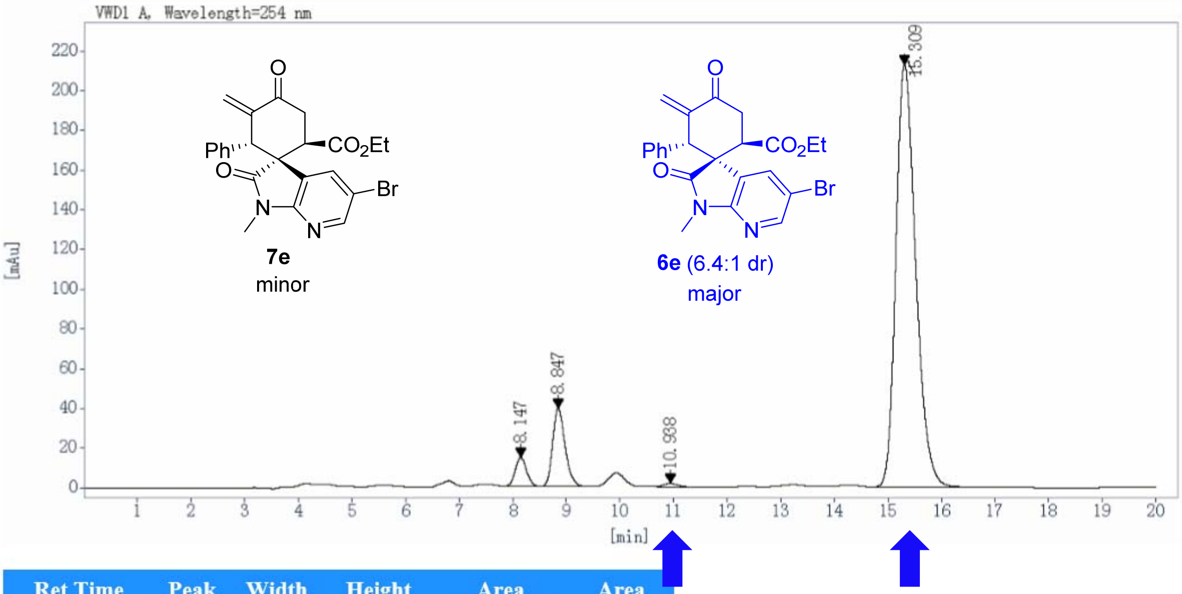

Ret Time [min]	Peak Type	Width [min]	Height [mAU]	Area [mAU*s]	Area [%]
8.147	FM	0.25	14.6131	215.5051	3.3979
8.847	BB	0.25	39.4695	633.5749	9.9896
10.938	VB	0.27	1.8403	32.5716	0.5136
15.309	BB	0.40	212.6768	5460.6792	86.0989
			Totals:	6342.3307	100.0000

HRMS (ESI-TOF) m/z: $[M + Na]^+$ Calcd for $C_{23}H_{21}O_4N_2BrNa^+$ 491.0577 (^{79}Br) and 493.0556 (^{81}Br); Found 491.0577 (^{79}Br) and 493.0561 (^{81}Br).

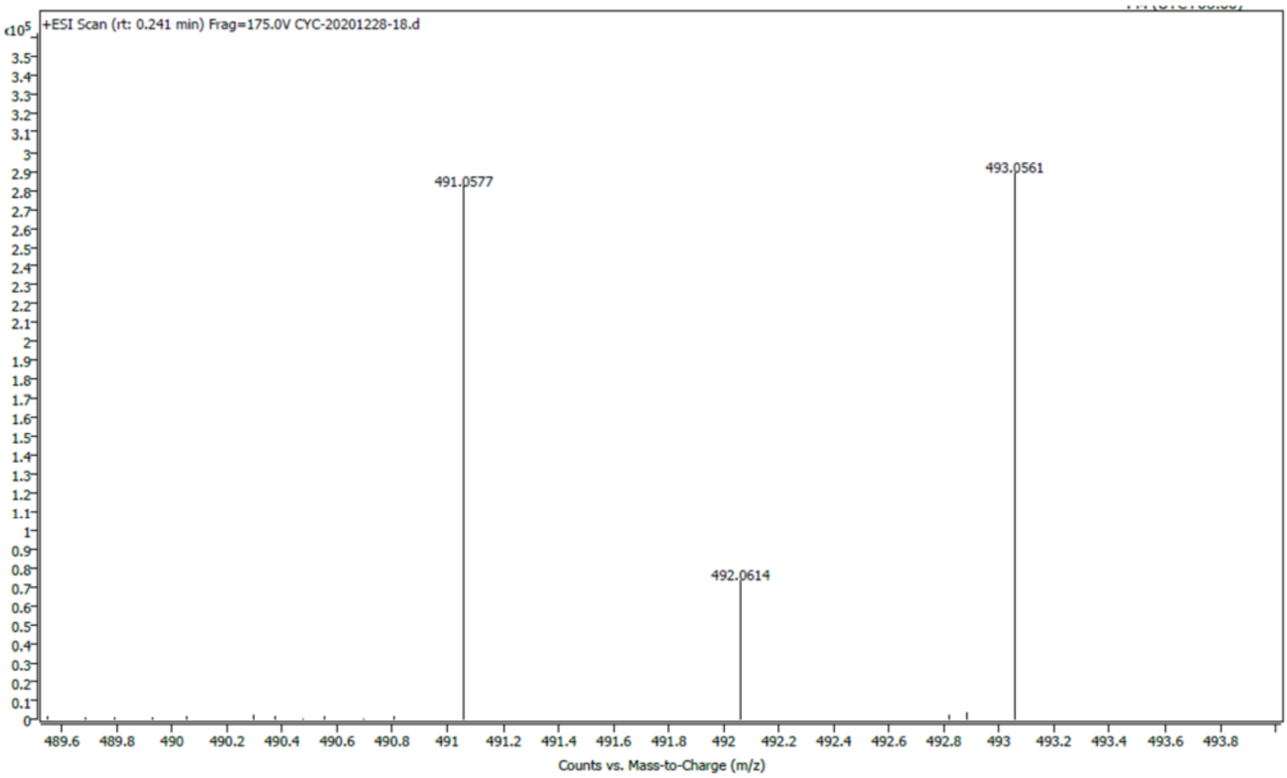

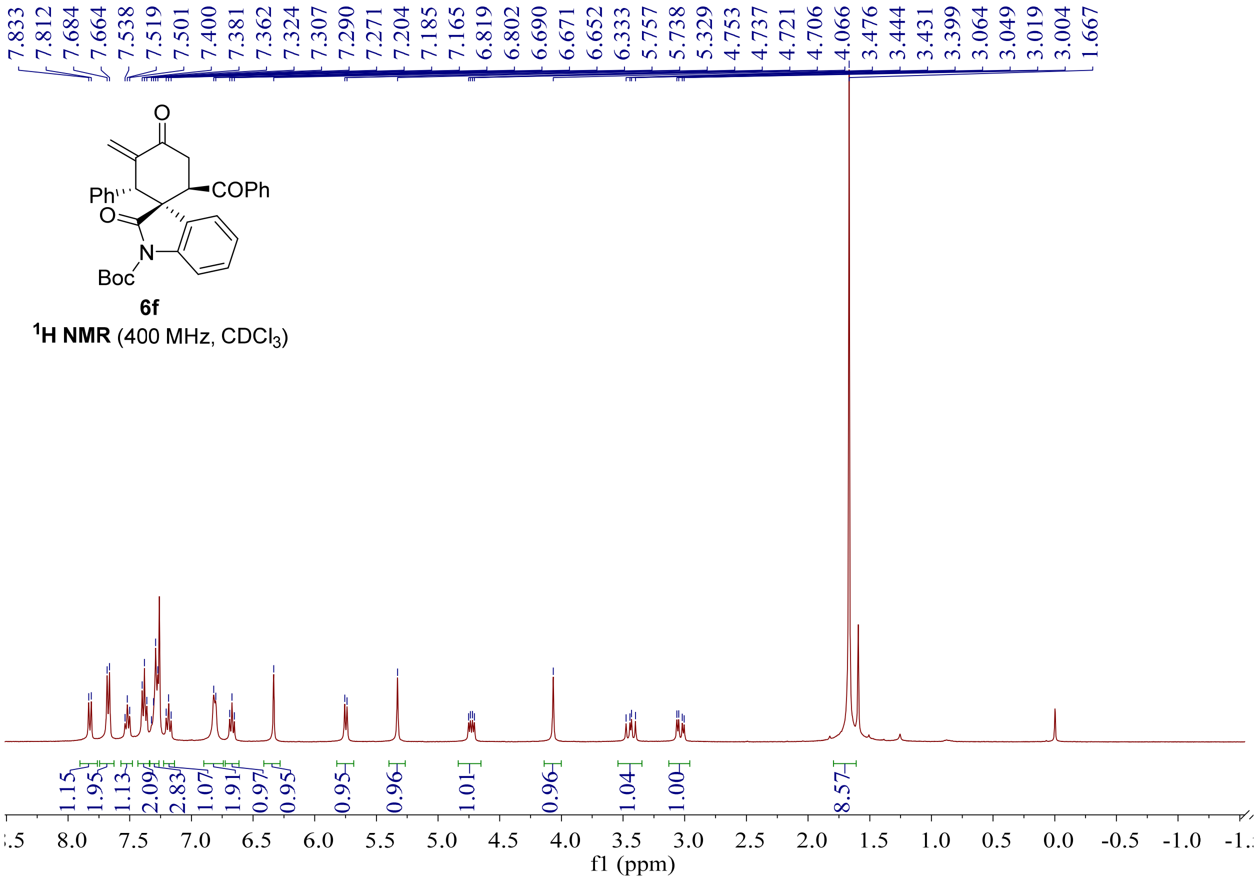

6f

^{1}H NMR (400 MHz, $CDCl_3$)

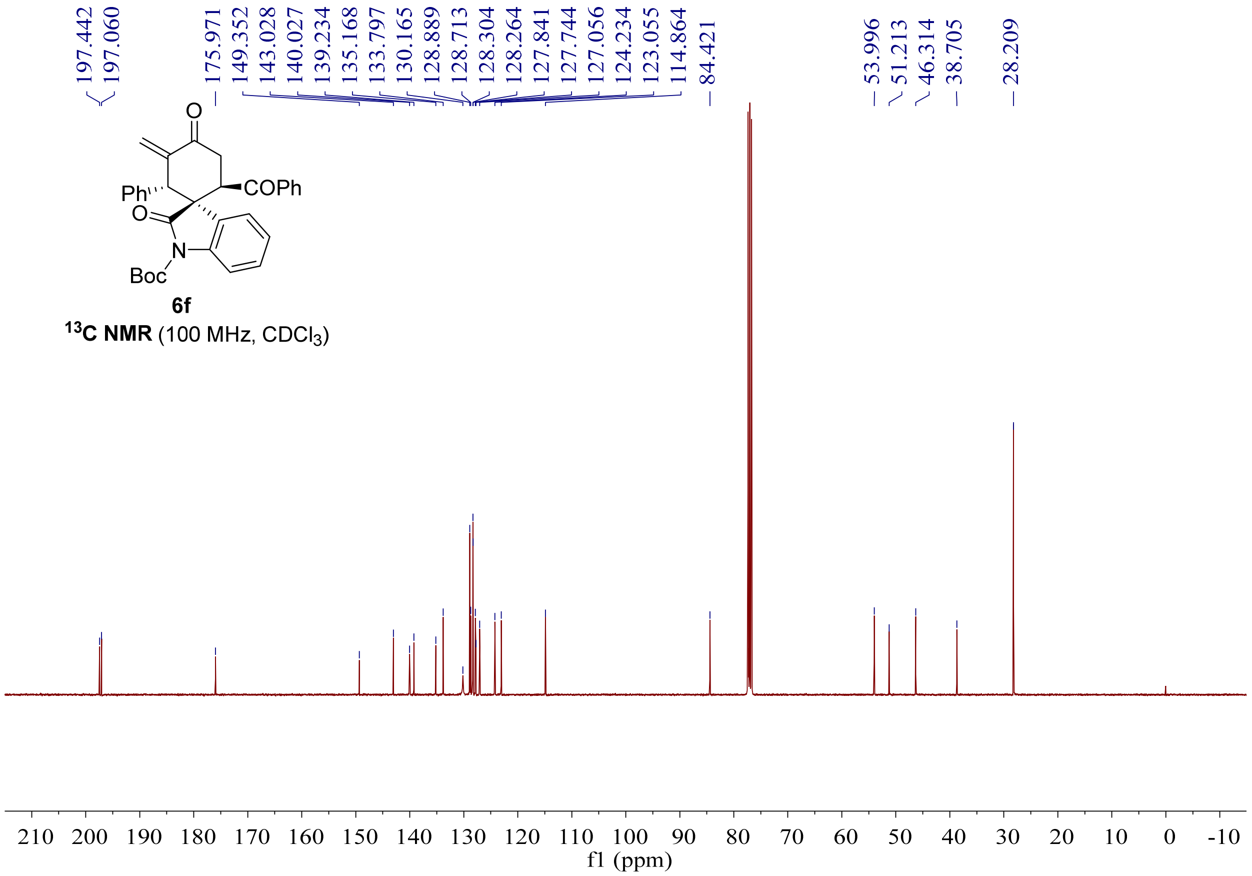

6f

^{13}C NMR (100 MHz, $CDCl_3$)

Daicel Chiral ID Column, *i*PrOH/*n*-hexane = 40/60, 1.0 mL/min.

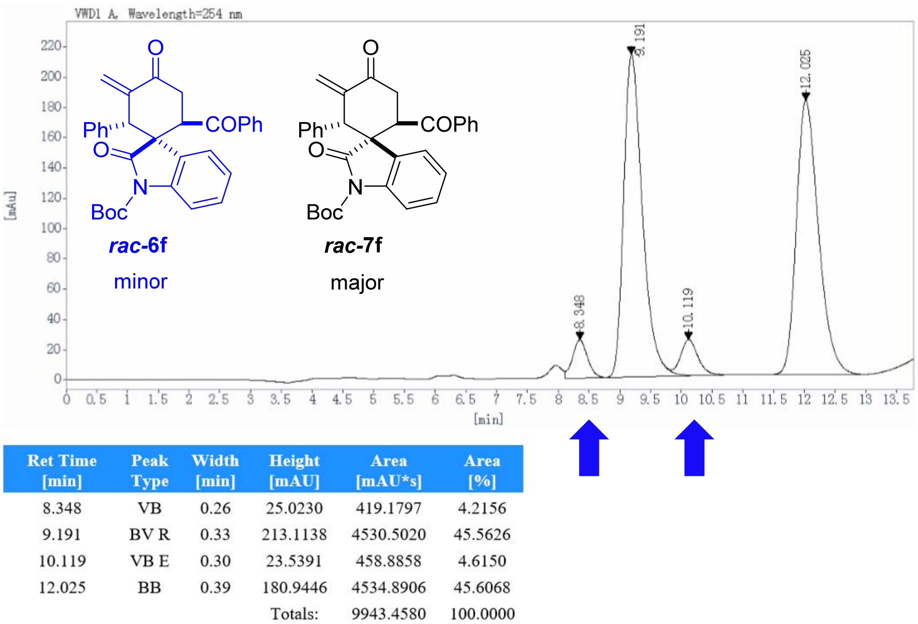

Ret Time [min]	Peak Type	Width [min]	Height [mAU]	Area [mAU*s]	Area [%]
8.348	VB	0.26	25.0230	419.1797	4.2156
9.191	BV R	0.33	213.1138	4530.5020	45.5626
10.119	VB E	0.30	23.5391	458.8858	4.6150
12.025	BB	0.39	180.9446	4534.8906	45.6068
			Totals:	9943.4580	100.0000

By using Pd(OAc)$_2$, triphenyl phosphite and TBAB system, *rac*-6f was formed as the minor diastereomer, whereas using Pd(OAc)$_2$, L8 and chiral C4 gave 6f as the major diastereomer. The mixture of diastereomers were tested.

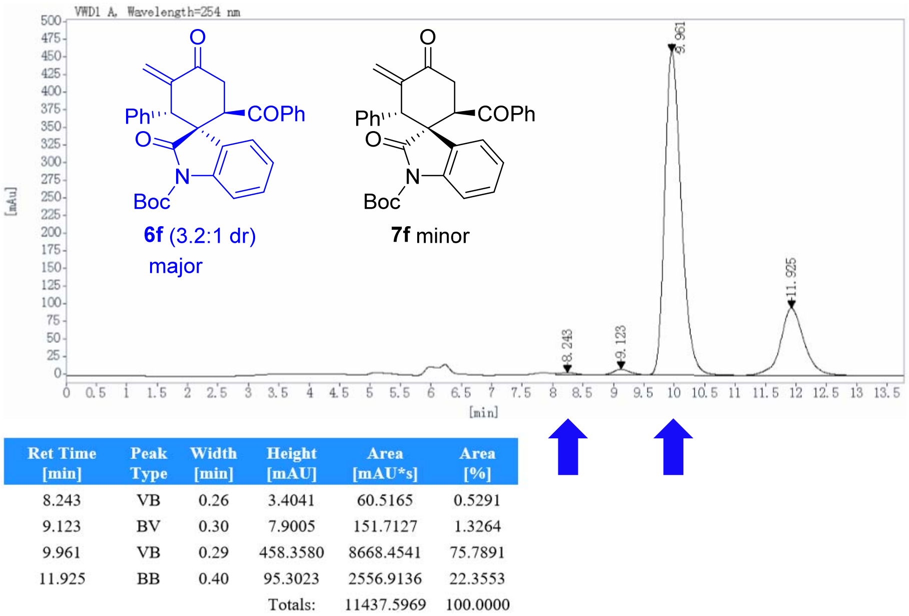

Ret Time [min]	Peak Type	Width [min]	Height [mAU]	Area [mAU*s]	Area [%]
8.243	VB	0.26	3.4041	60.5165	0.5291
9.123	BV	0.30	7.9005	151.7127	1.3264
9.961	VB	0.29	458.3580	8668.4541	75.7891
11.925	BB	0.40	95.3023	2556.9136	22.3553
			Totals:	11437.5969	100.0000

HRMS (ESI-TOF) m/z: [M + Na]$^+$ Calcd for $C_{32}H_{29}O_5NNa^+$ 530.1938; Found 530.1940.

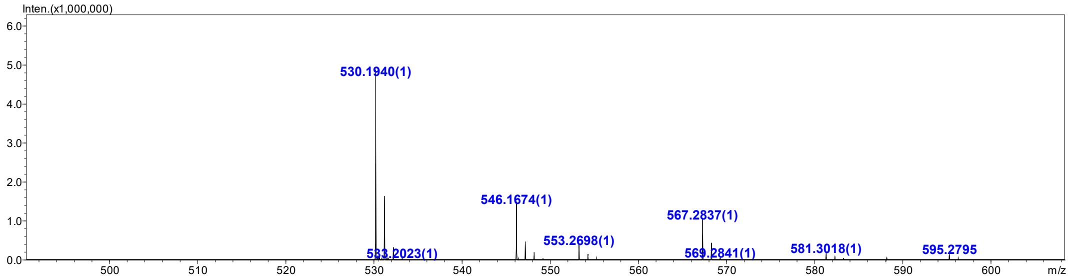

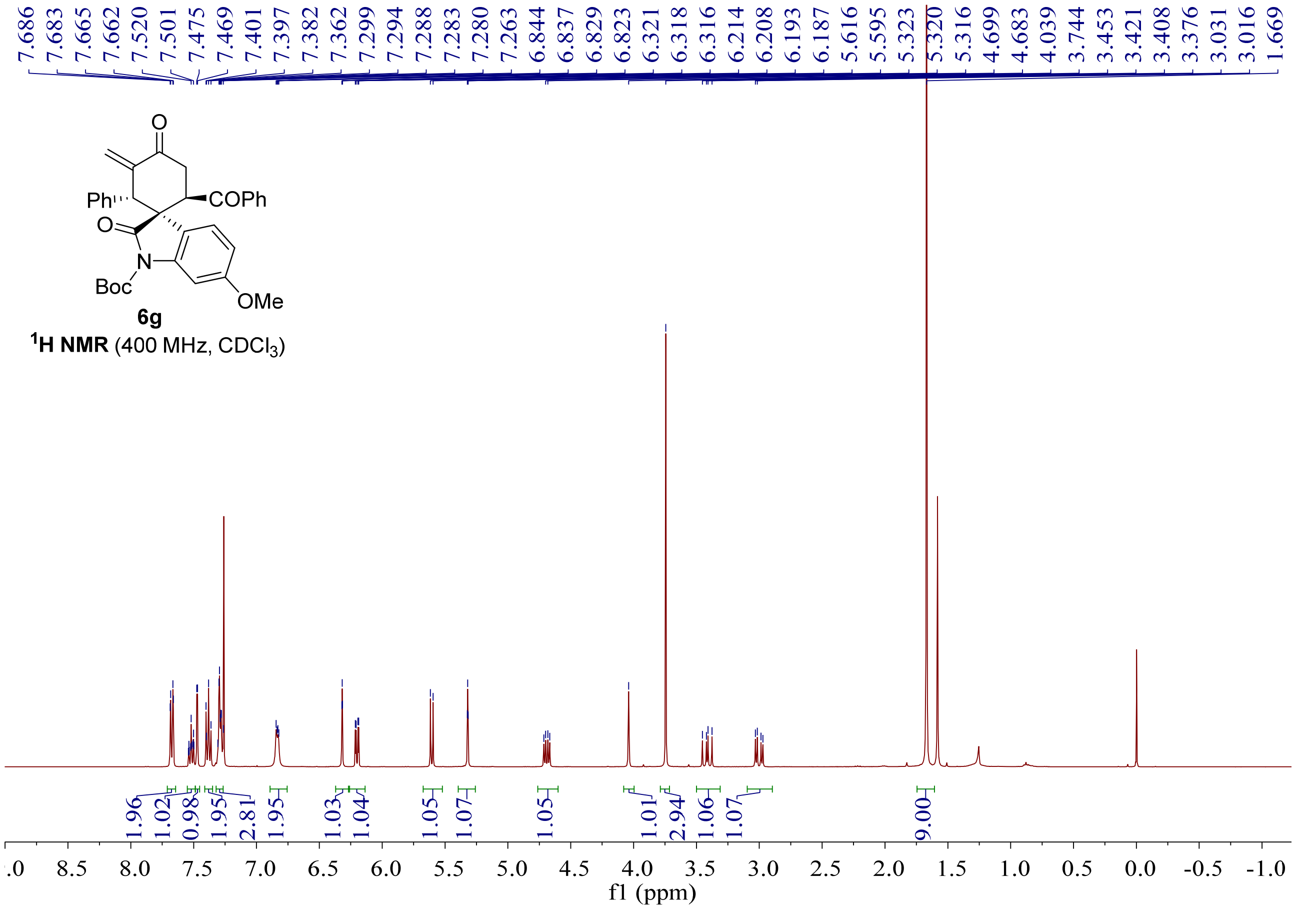
7.686 7.683 7.665 7.662 7.520 7.501 7.475 7.469 7.401 7.397 7.382 7.362 7.299 7.294 7.288 7.283 7.280 7.263 6.844 6.837 6.829 6.823 6.321 6.318 6.316 6.214 6.208 6.193 6.187 5.616 5.595 5.323 5.320 5.316 4.699 4.683 4.039 3.744 3.453 3.421 3.408 3.376 3.031 3.016 1.669
O
Ph
COPh
O
N
Boc
OMe
6g
1H NMR (400 MHz, CDCl3)
1.96 1.02 0.98 1.95 2.81 1.95 1.03 1.04 1.05 1.07 1.05 1.01 2.94 1.06 1.07 9.00
f1 (ppm)

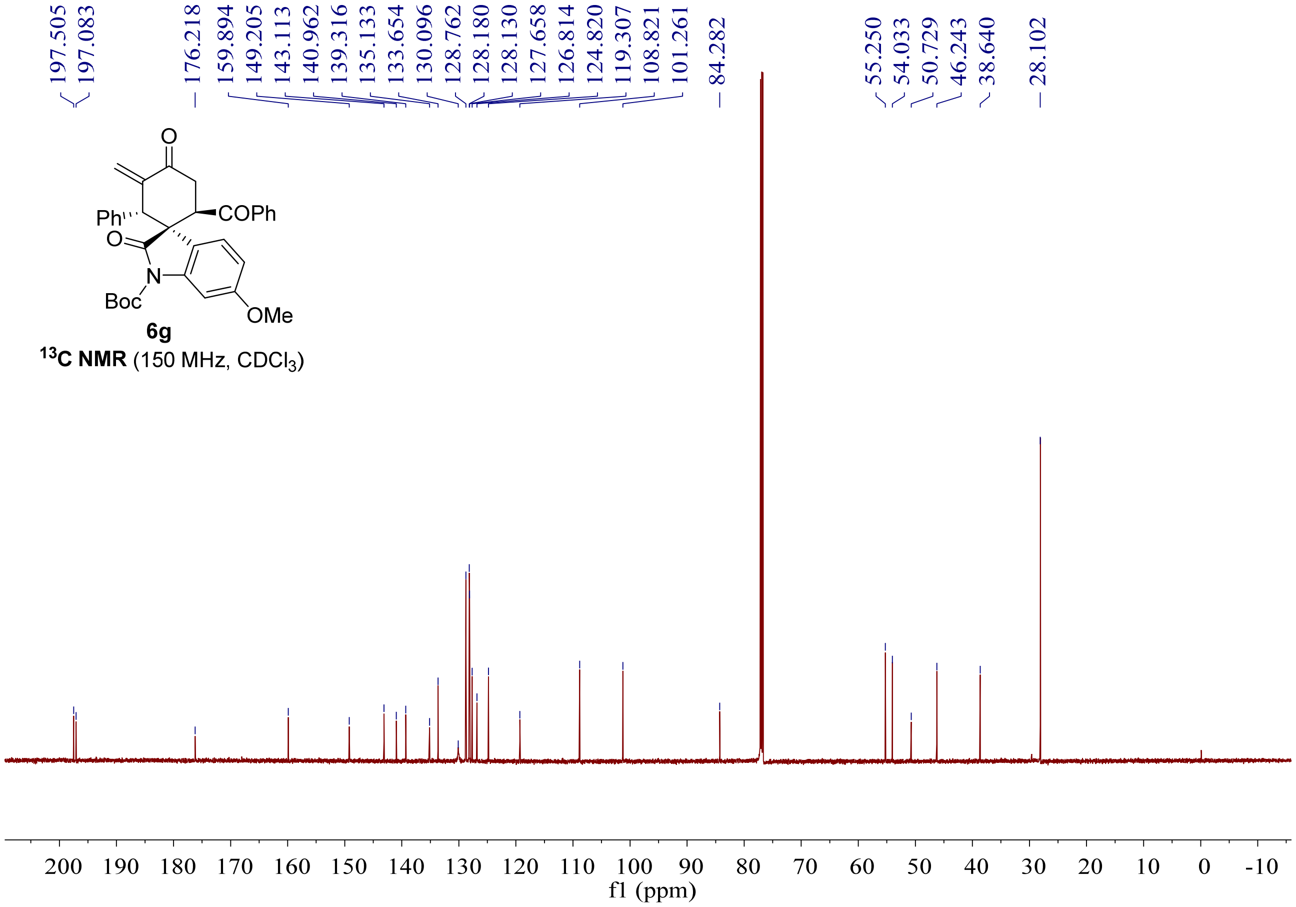
197.505 197.083 176.218 159.894 149.205 143.113 140.962 139.316 135.133 133.654 130.096 128.762 128.180 128.130 127.658 126.814 124.820 119.307 108.821 101.261 84.282 55.250 54.033 50.729 46.243 38.640 28.102
O
Ph
COPh
O
N
Boc
OMe
6g
13C NMR (150 MHz, CDCl3)
f1 (ppm)

Daicel Chiral IF Column, eluent (20% V/V isopropanol dissolved in *n*-hexane)

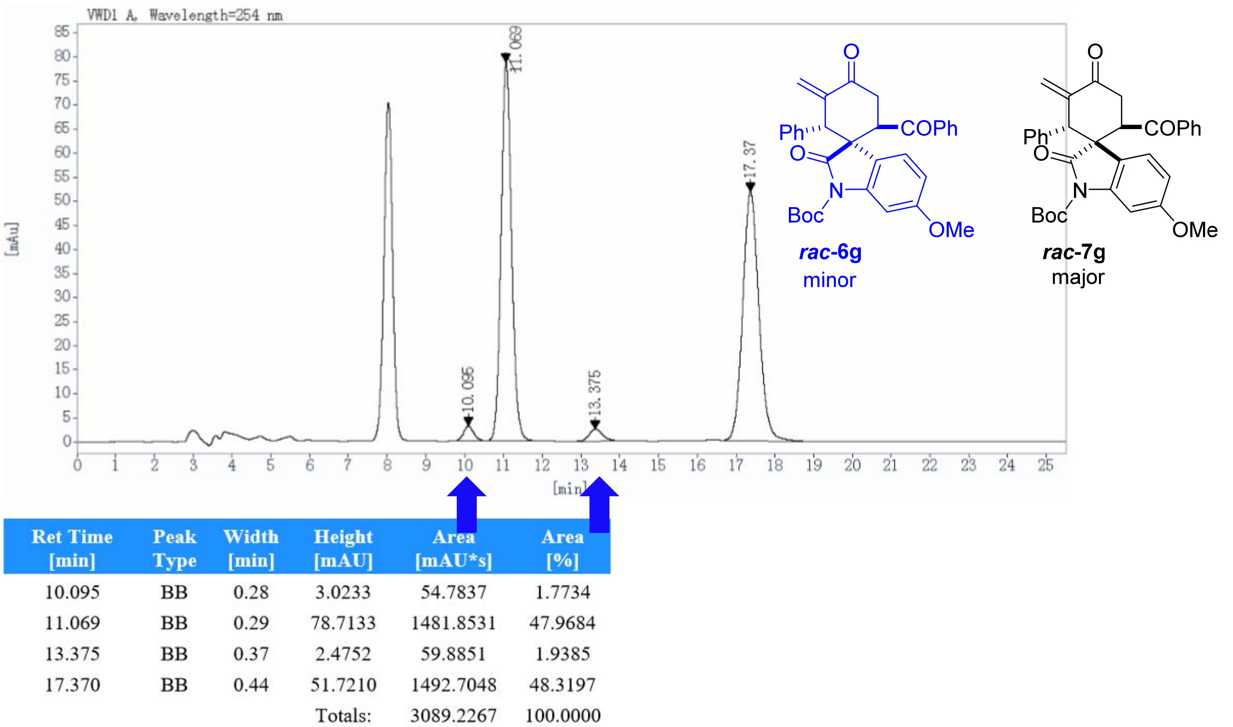

Ret Time [min]	Peak Type	Width [min]	Height [mAU]	Area [mAU*s]	Area [%]
10.095	BB	0.28	3.0233	54.7837	1.7734
11.069	BB	0.29	78.7133	1481.8531	47.9684
13.375	BB	0.37	2.4752	59.8851	1.9385
17.370	BB	0.44	51.7210	1492.7048	48.3197
			Totals:	3089.2267	100.0000

By using Pd(OAc)₂, triphenyl phosphite and TBAB system, *rac*-6g was formed as the minor diastereomer, whereas using Pd(OAc)₂, L8 and chiral C4 gave 6g as the major diastereomer. The mixture of diastereomers were tested.

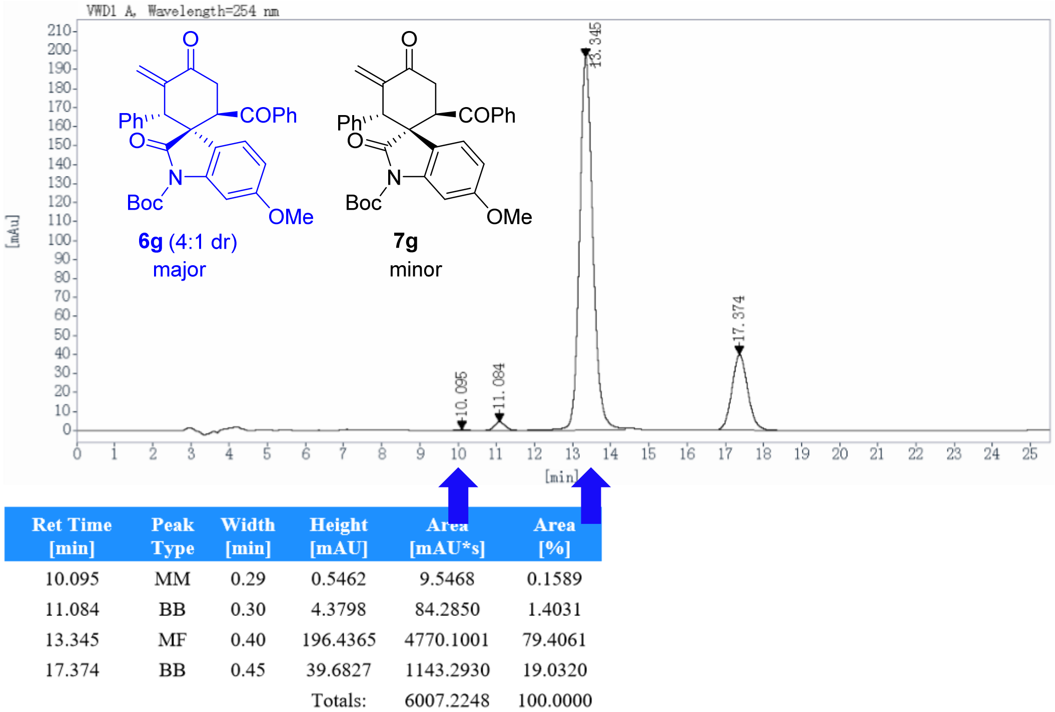

Ret Time [min]	Peak Type	Width [min]	Height [mAU]	Area [mAU*s]	Area [%]
10.095	MM	0.29	0.5462	9.5468	0.1589
11.084	BB	0.30	4.3798	84.2850	1.4031
13.345	MF	0.40	196.4365	4770.1001	79.4061
17.374	BB	0.45	39.6827	1143.2930	19.0320
			Totals:	6007.2248	100.0000

HRMS (ESI-TOF) m/z: $[M + Na]^+$ Calcd for $C_{32}H_{31}O_6NNa^+$ 560.2044; Found 560.2042.

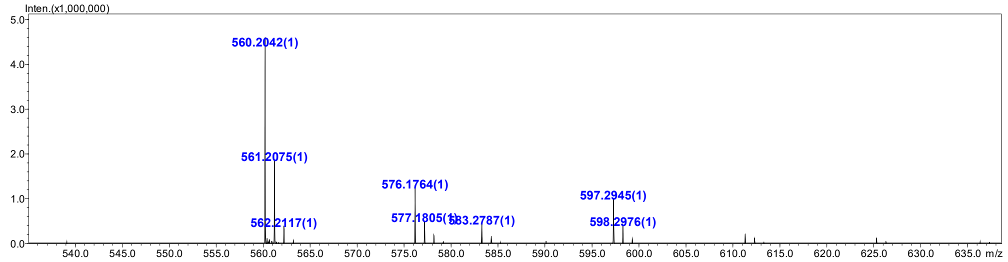

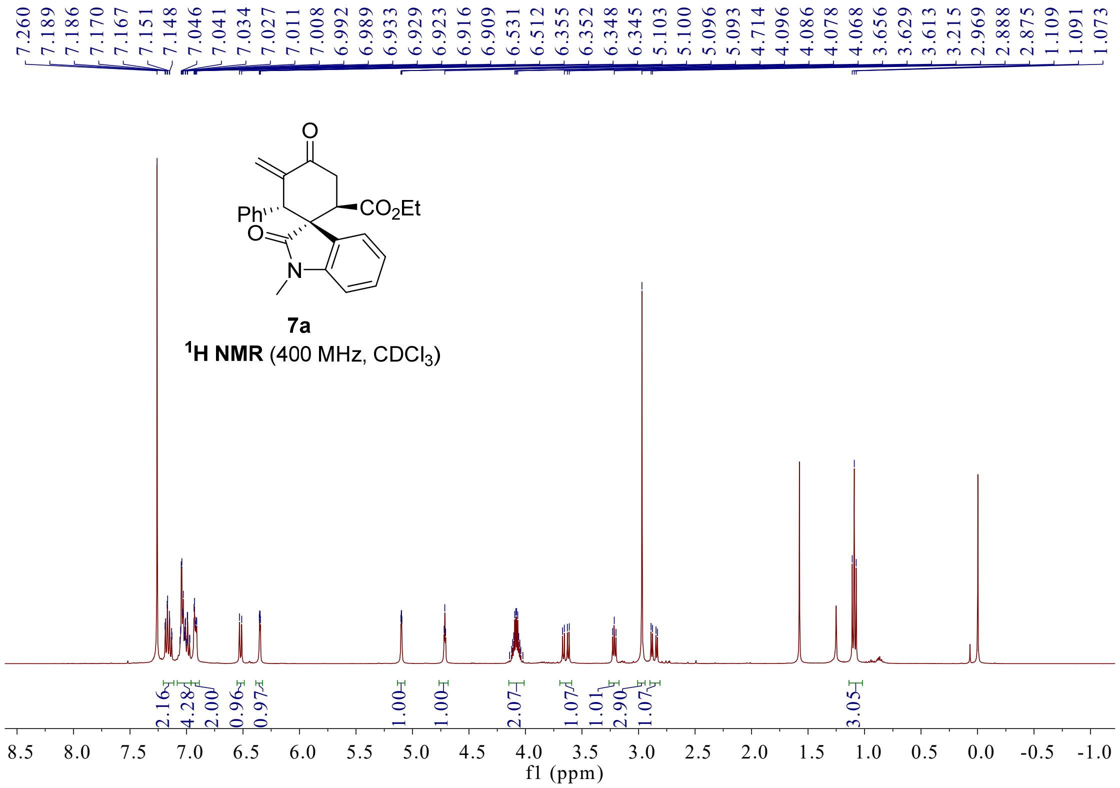
7.260 7.189 7.186 7.170 7.167 7.151 7.148 7.046 7.041 7.034 7.027 7.011 7.008 6.992 6.989 6.933 6.929 6.923 6.916 6.909 6.531 6.512 6.355 6.352 6.348 6.345 5.103 5.100 5.096 5.093 4.714 4.096 4.086 4.078 4.068 3.656 3.629 3.613 3.215 2.969 2.888 2.875 1.109 1.091 1.073
O
Ph
CO2Et
O
N
7a
1H NMR (400 MHz, CDCl3)
2.16 4.28 2.00 0.96 0.97 1.00 1.00 2.07 1.07 1.01 2.90 1.07 3.05
8.5 8.0 7.5 7.0 6.5 6.0 5.5 5.0 4.5 4.0 3.5 3.0 2.5 2.0 1.5 1.0 0.5 0.0 -0.5 -1.0
f1 (ppm)

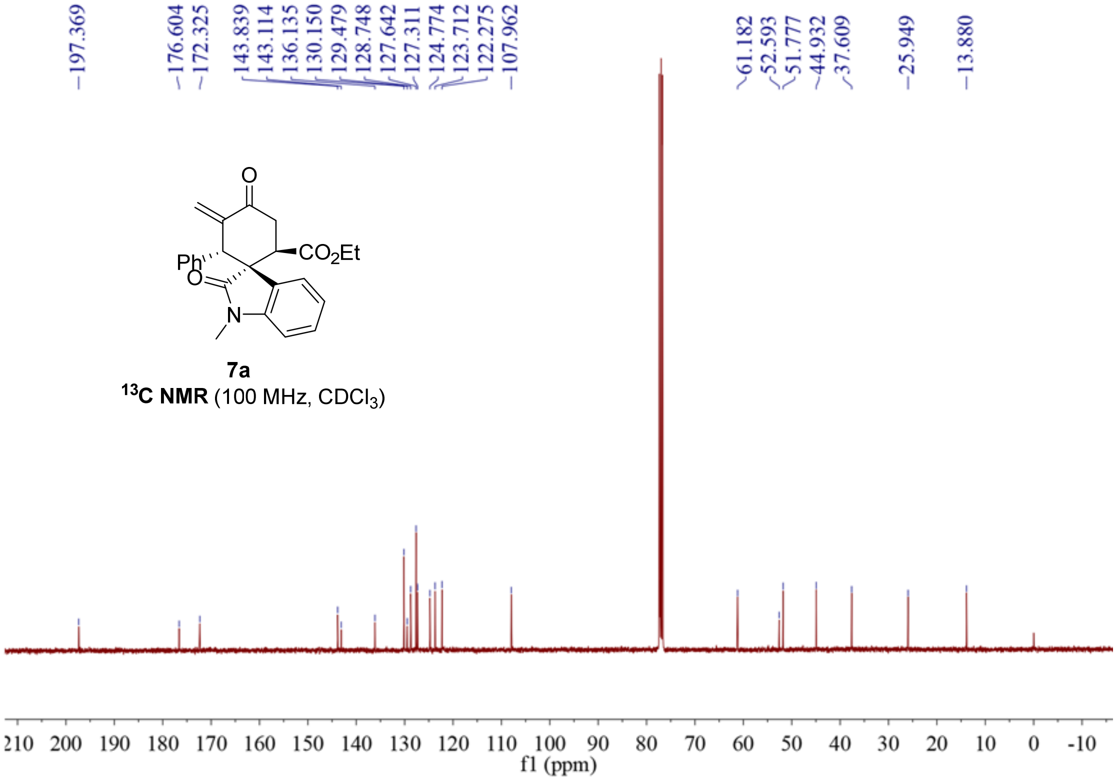
197.369
176.604 172.325
143.839 143.114 136.135 130.150 129.479 128.748 127.642 127.311 124.774 123.712 122.275 107.962
61.182 52.593 51.777 44.932 37.609
25.949
13.880
O
Ph
CO2Et
O
N
7a
13C NMR (100 MHz, CDCl3)
210 200 190 180 170 160 150 140 130 120 110 100 90 80 70 60 50 40 30 20 10 0 -10
f1 (ppm)

Daicel Chiral IE Column, (*i*PrOH/*n*-hexane = 40/60, 1.0 mL/min)

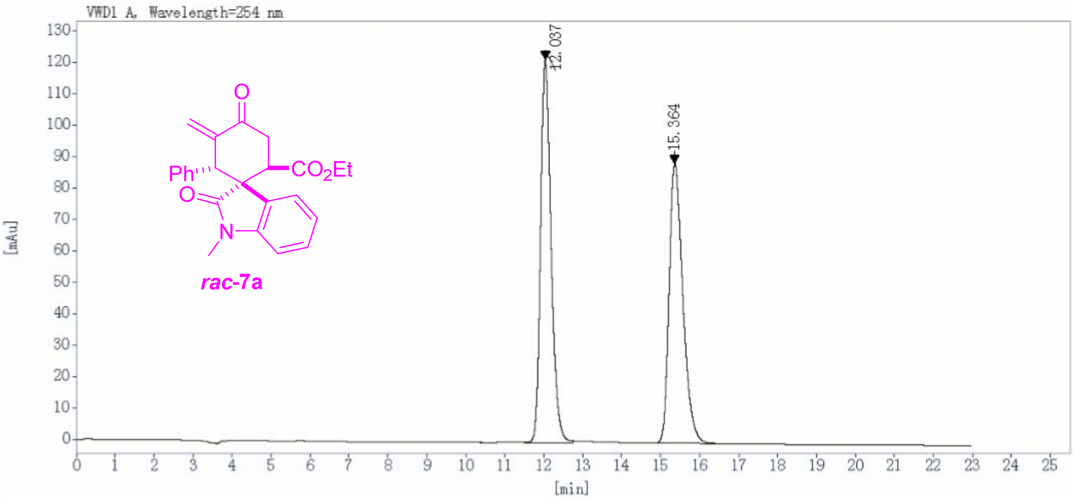

Ret Time [min]	Peak Type	Width [min]	Height [mAU]	Area [mAU*s]	Area [%]
12.037	FM	0.31	122.0207	2281.0793	51.0986
15.364	BB	0.38	89.0237	2182.9937	48.9014
			Totals:	4464.0730	100.0000

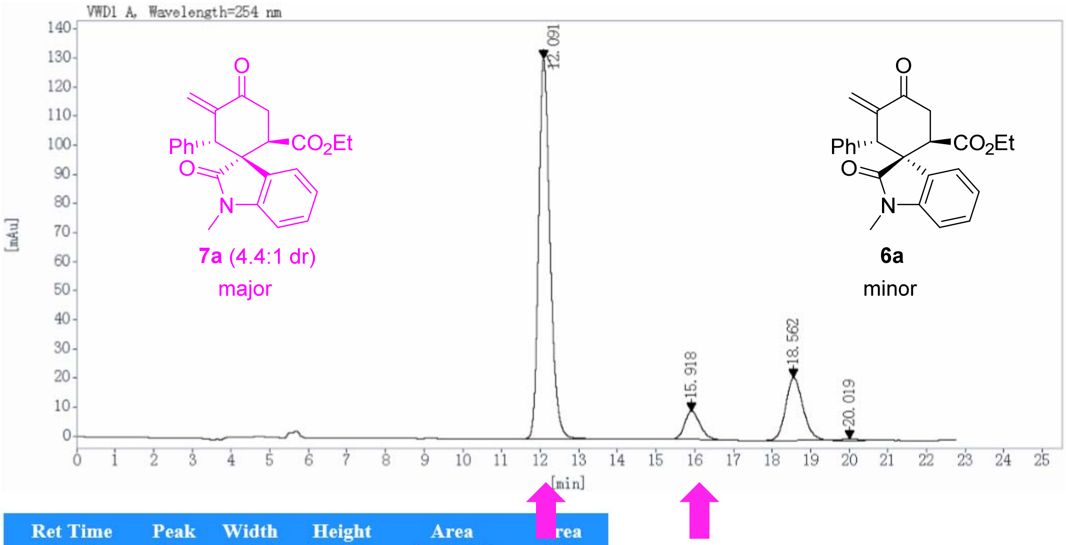

Ret Time [min]	Peak Type	Width [min]	Height [mAU]	Area [mAU*s]	[illegible]rea [%]
12.091	MF	0.34	130.7411	2689.7427	73.7939
15.918	BB	0.42	9.7852	270.3073	7.4160
18.562	BB	0.48	21.5139	669.1799	18.3591
20.019	MF	0.54	0.4820	15.7096	0.4310
			Totals:	3644.9394	100.0000

HRMS (ESI-TOF) m/z: $[M + Na]^+$ Calcd for $C_{24}H_{23}O_4NNa^+$ 412.1519; Found 412.1518.

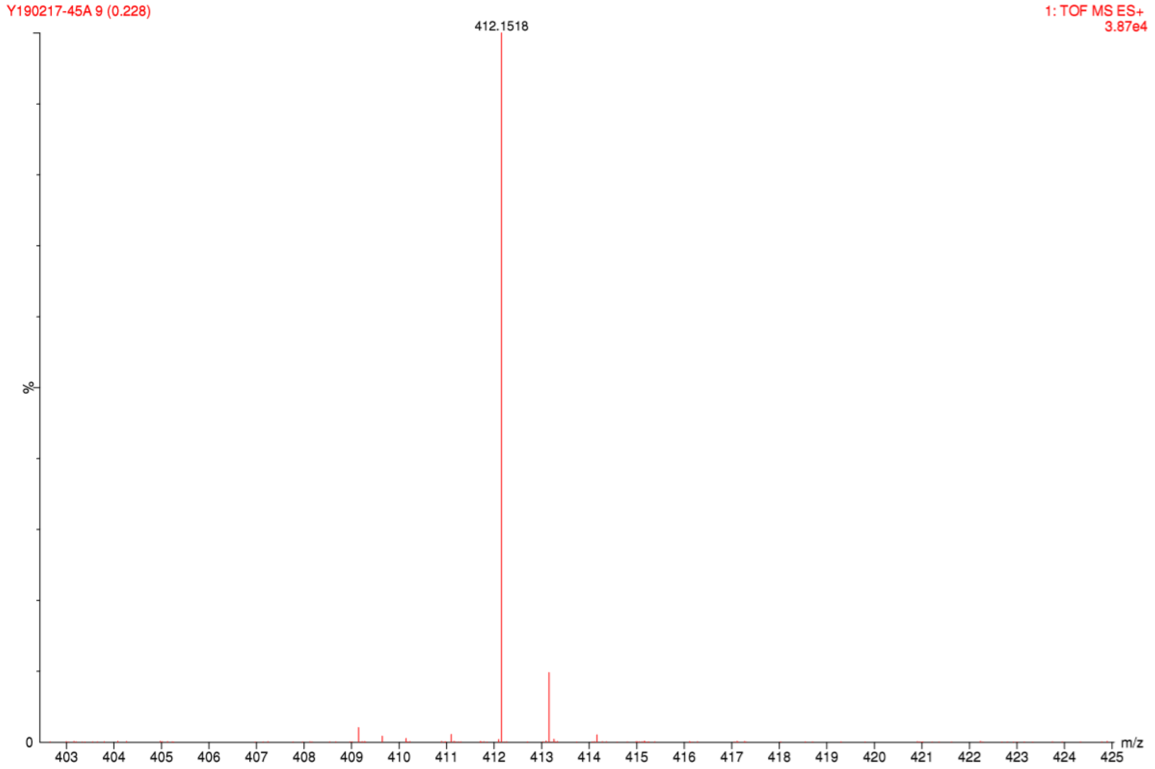

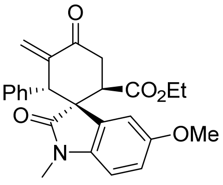

7b

^{1}H NMR (400 MHz, $CDCl_3$)

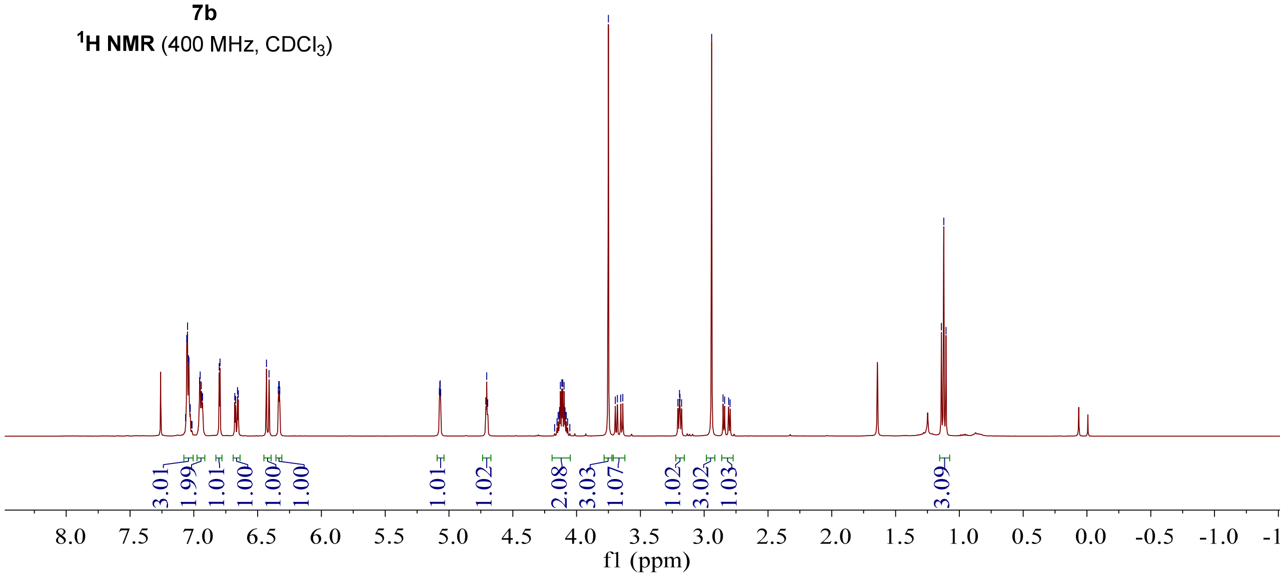

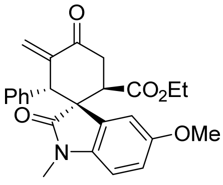

7b

^{13}C NMR (100 MHz, $CDCl_3$)

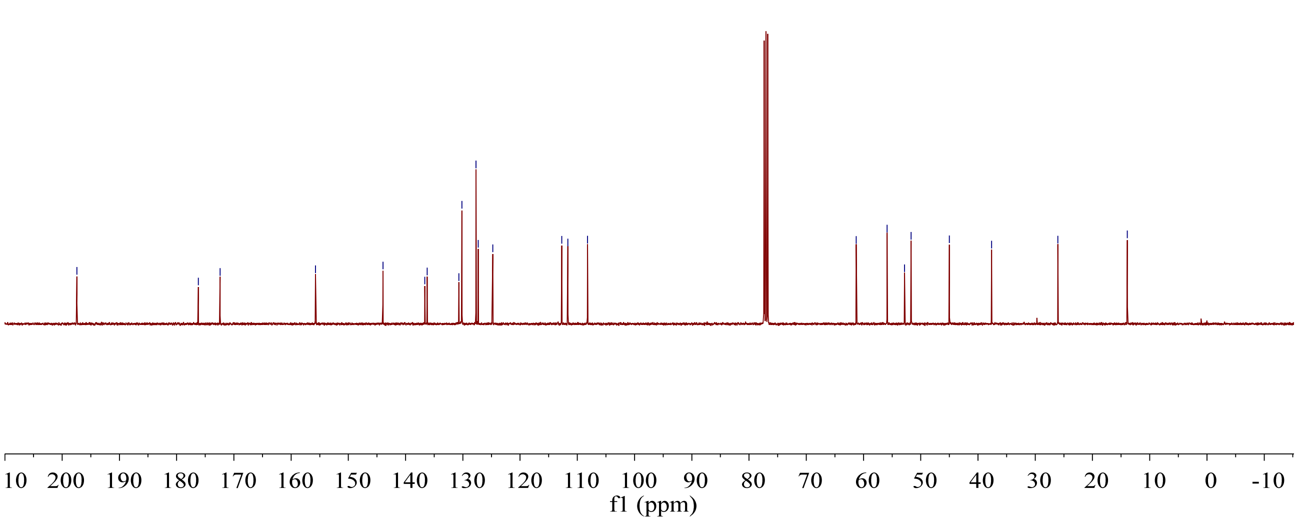

Daicel Chiral IE Column, *i*PrOH/*n*-hexane = 40/60, 1.0 mL/min

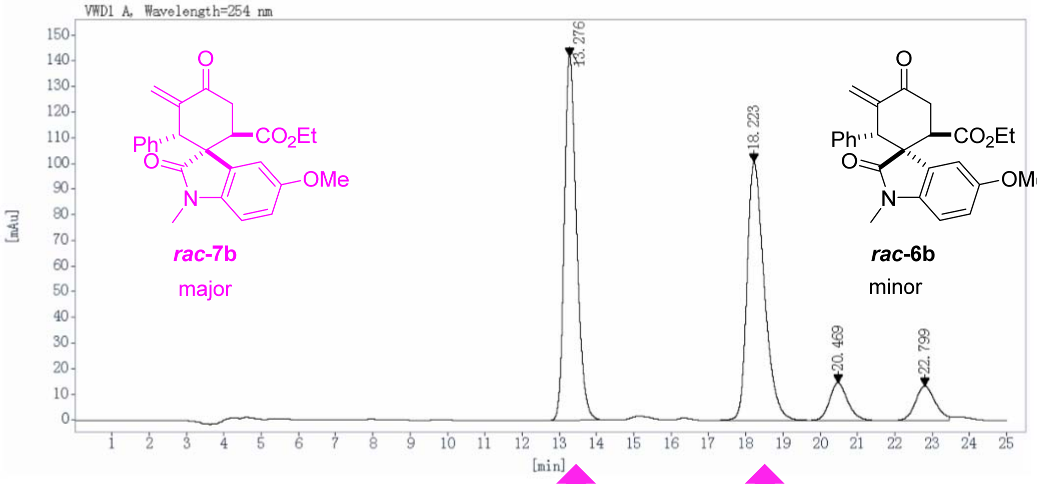

Ret Time [min]	Peak Type	Width [min]	Height [mAU]	Area [mAU*s]	Area [%]
13.276	BB	0.35	141.9306	3213.4878	43.2908
18.223	BB	0.49	100.6586	3228.1821	43.4888
20.469	BB	0.52	14.4664	486.4908	6.5538
22.799	MF	0.62	13.3415	494.8615	6.6666
			Totals:	7423.0222	100.0000

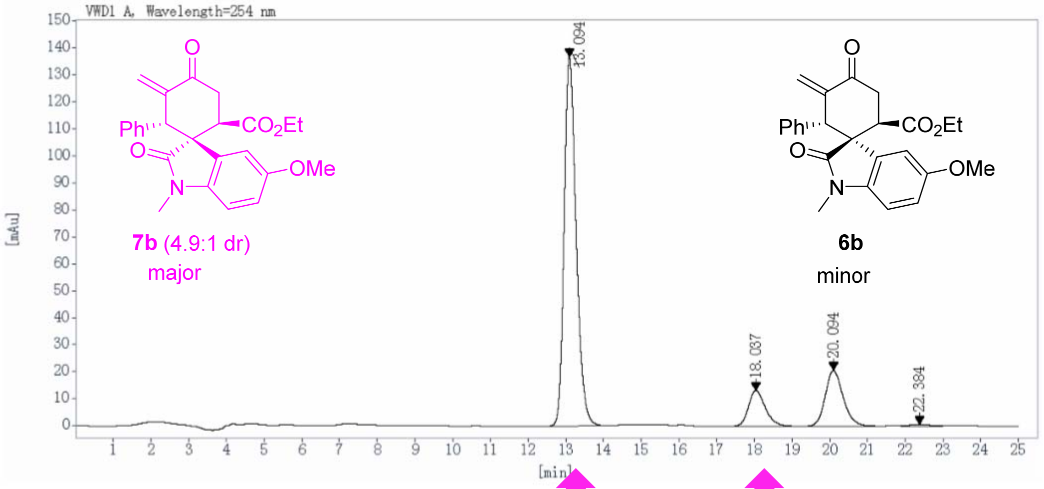

Ret Time [min]	Peak Type	Width [min]	Height [mAU]	Area [mAU*s]	Area [%]
13.094	BB	0.34	136.8103	3010.9121	73.1189
18.037	BB	0.47	13.1937	406.1255	9.8626
20.094	BB	0.50	20.5746	676.8121	16.4361
22.384	MM	0.63	0.6321	23.9815	0.5824
			Totals:	4117.8313	100.0000

HRMS (ESI-TOF) m/z: $[M + Na]^+$ Calcd for $C_{25}H_{25}O_5NNa^+$ 442.1625; Found 442.1622.

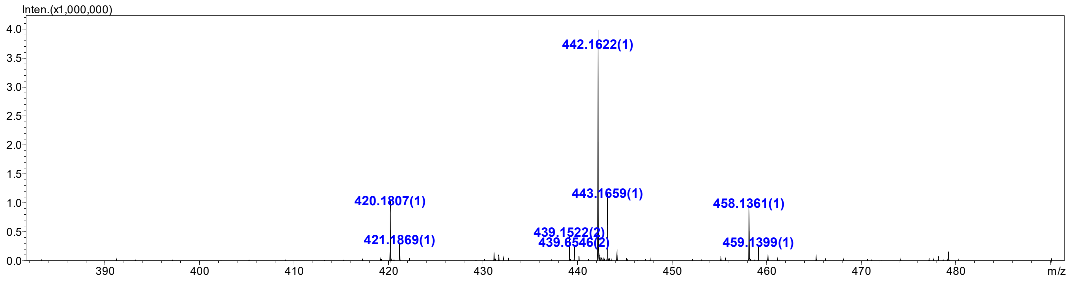

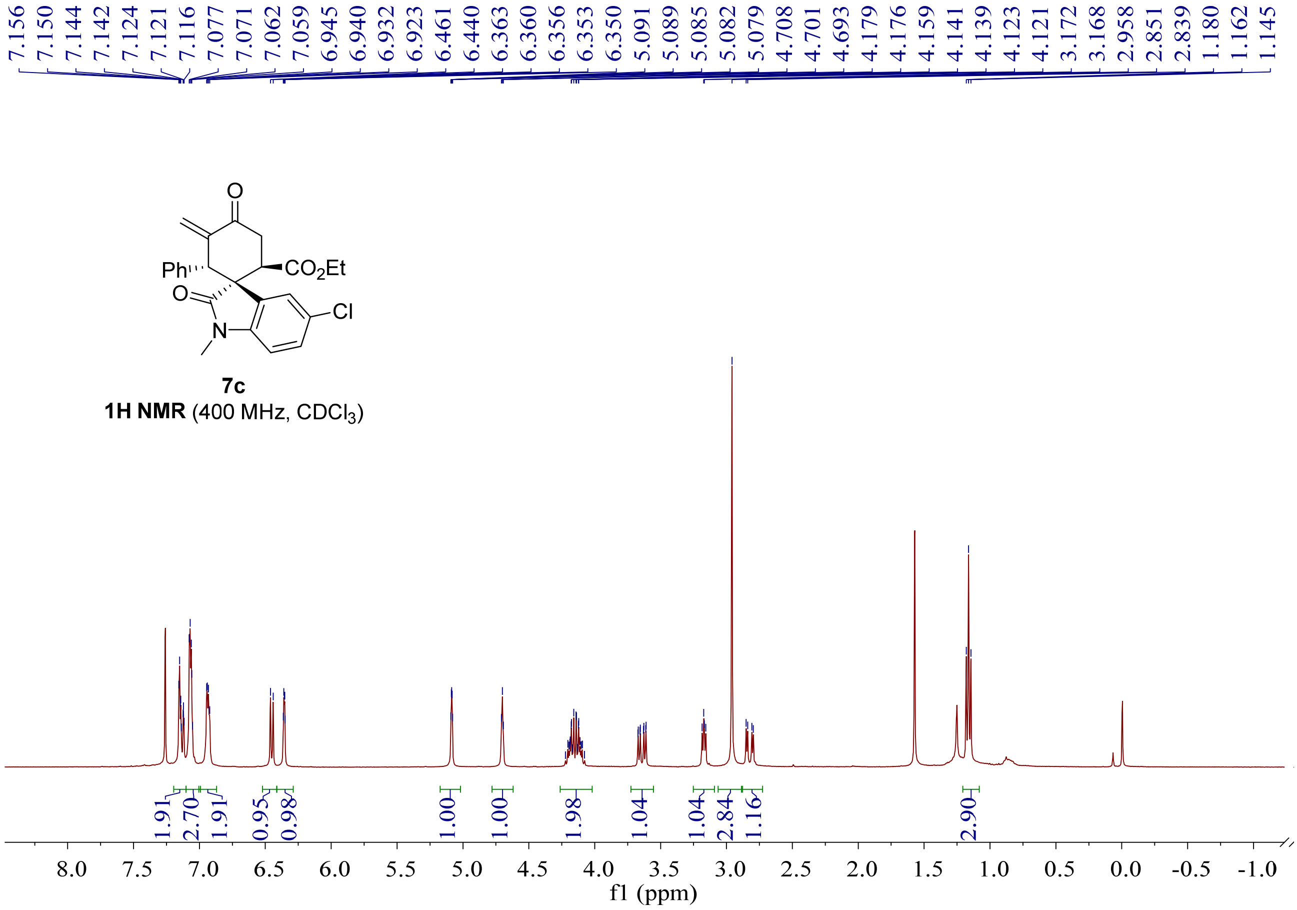
7.156 7.150 7.144 7.142 7.124 7.121 7.116 7.077 7.071 7.062 7.059 6.945 6.940 6.932 6.923 6.461 6.440 6.363 6.360 6.356 6.353 6.350 5.091 5.089 5.085 5.082 5.079 4.708 4.701 4.693 4.179 4.176 4.159 4.141 4.139 4.123 4.121 3.172 3.168 2.958 2.851 2.839 1.180 1.162 1.145
O
Ph
CO_2Et
O
N
Cl
7c
1H NMR (400 MHz, $CDCl_3$)
1.91 2.70 1.91 0.95 0.98 1.00 1.00 1.98 1.04 1.04 2.84 1.16 2.90
8.0 7.5 7.0 6.5 6.0 5.5 5.0 4.5 4.0 3.5 3.0 2.5 2.0 1.5 1.0 0.5 0.0 -0.5 -1.0
f1 (ppm)

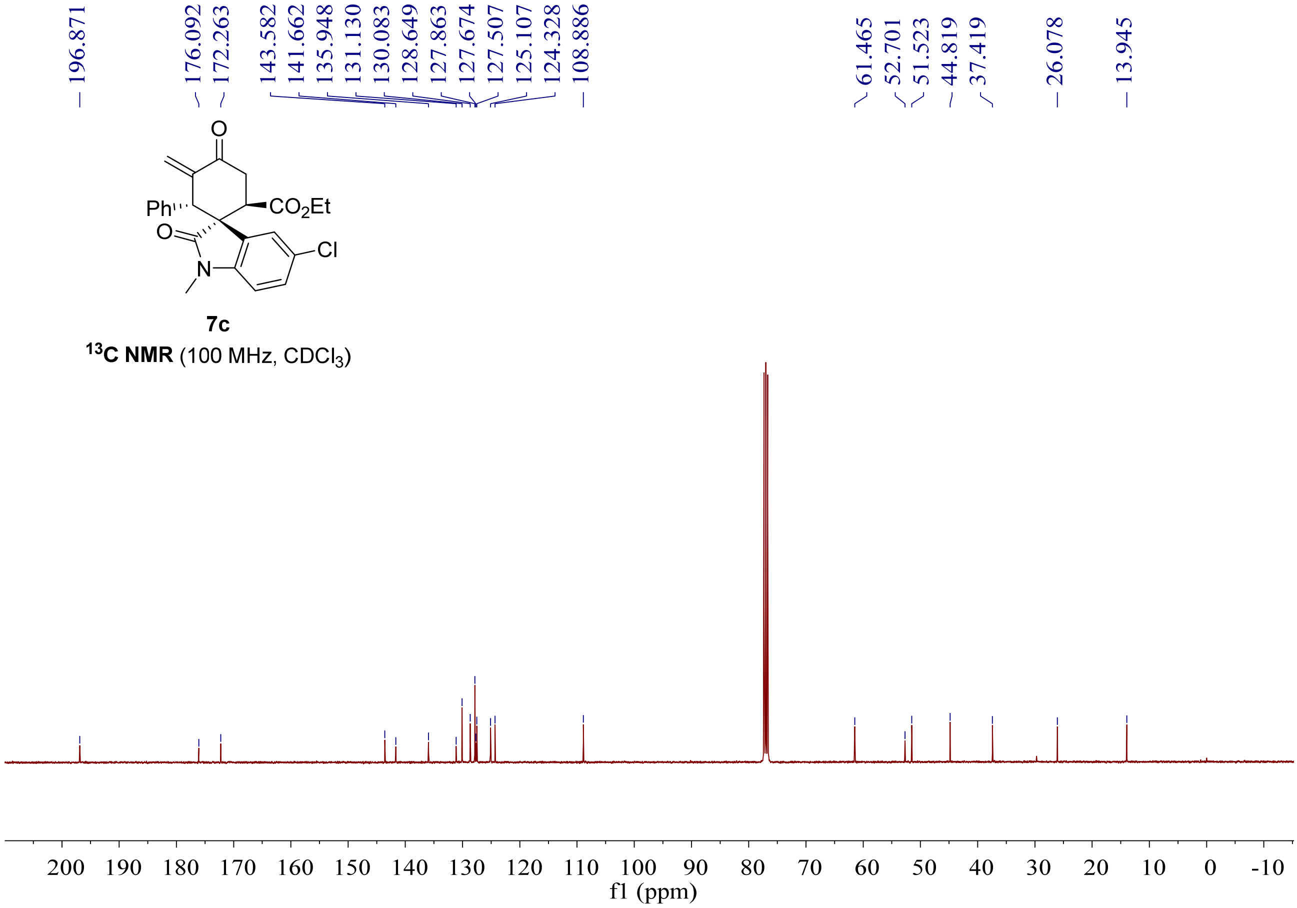
196.871 176.092 172.263 143.582 141.662 135.948 131.130 130.083 128.649 127.863 127.674 127.507 125.107 124.328 108.886 61.465 52.701 51.523 44.819 37.419 26.078 13.945
O
Ph
CO_2Et
O
N
Cl
7c
^{13}C NMR (100 MHz, $CDCl_3$)
200 190 180 170 160 150 140 130 120 110 100 90 80 70 60 50 40 30 20 10 0 -10
f1 (ppm)

Daicel Chiral IE Column, *i*PrOH/*n*-hexane = 40/60, 1.0 mL/min.

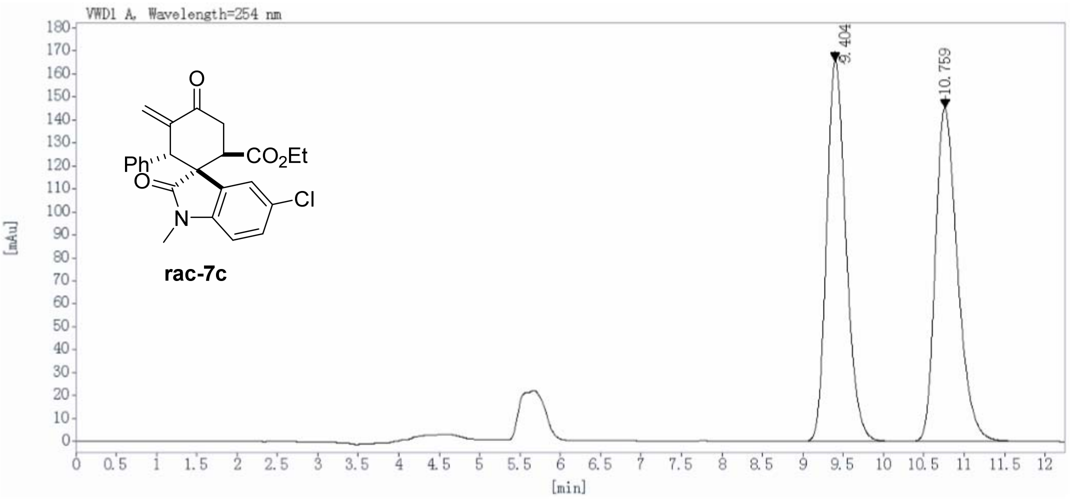

Ret Time [min]	Peak Type	Width [min]	Height [mAU]	Area [mAU*s]	Area [%]
9.404	BB	0.26	165.7225	2779.1226	50.0545
10.759	BB	0.29	145.1131	2773.0681	49.9455
			Totals:	5552.1907	100.0000

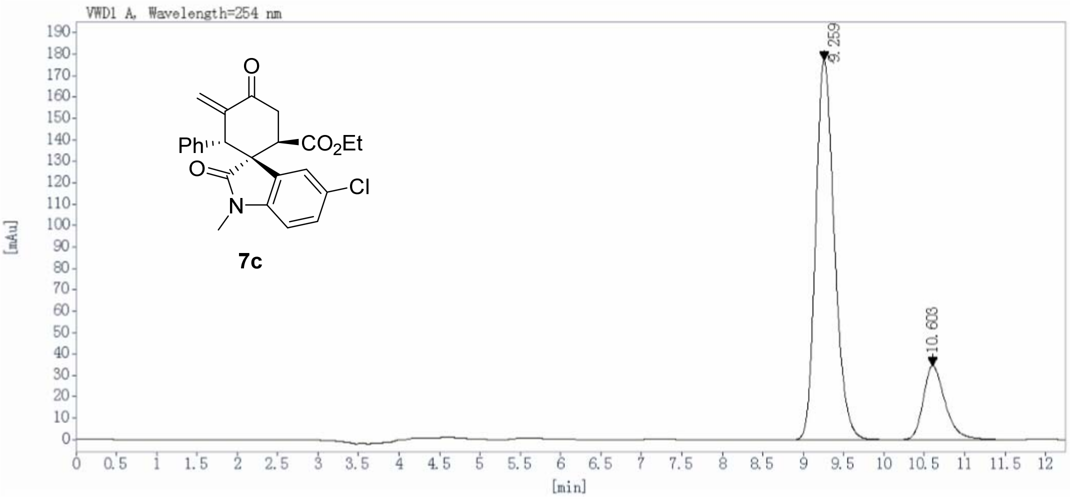

Ret Time [min]	Peak Type	Width [min]	Height [mAU]	Area [mAU*s]	Area [%]
9.259	BB	0.25	177.3208	2865.6379	81.6188
10.603	BB	0.29	34.4214	645.3641	18.3812
			Totals:	3511.0020	100.0000

HRMS (ESI-TOF) m/z: $[M + Na]^+$ Calcd for $C_{24}H_{22}O_4NClNa^+$ 446.1130 (^{35}Cl) and 448.1100 (^{37}Cl); Found 446.1129 (^{35}Cl) and 448.1109 (^{37}Cl).

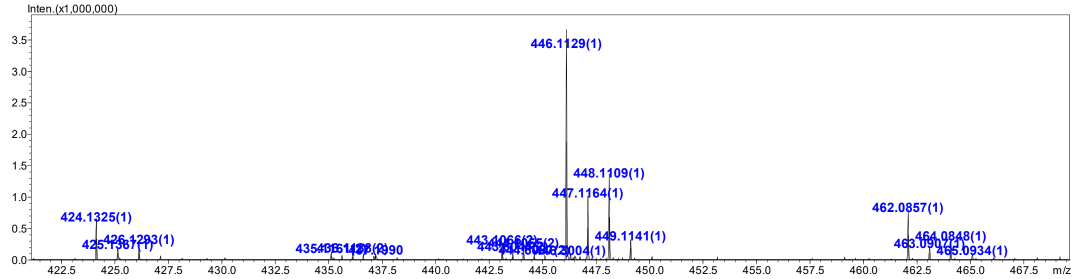

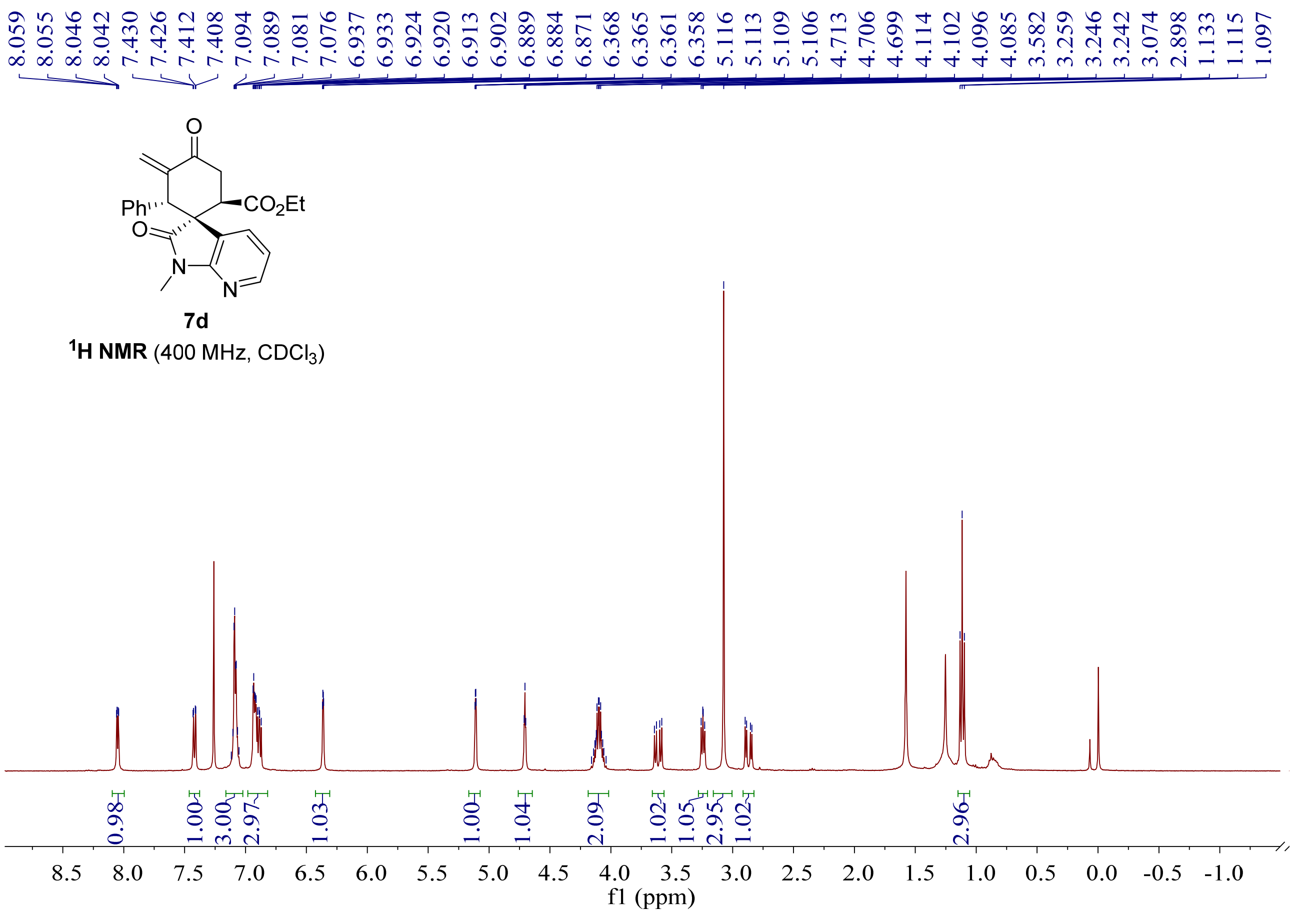
8.059
8.055
8.046
8.042
7.430
7.426
7.412
7.408
7.094
7.089
7.081
7.076
6.937
6.933
6.924
6.920
6.913
6.902
6.889
6.884
6.871
6.368
6.365
6.361
6.358
5.116
5.113
5.109
5.106
4.713
4.706
4.699
4.114
4.102
4.096
4.085
3.582
3.259
3.246
3.242
3.074
2.898
1.133
1.115
1.097
O
Ph
CO2Et
O
N
N
7d
1H NMR (400 MHz, CDCl3)
0.98
1.00
3.00
2.97
1.03
1.00
1.04
2.09
1.02
1.05
2.95
1.02
2.96
8.5
8.0
7.5
7.0
6.5
6.0
5.5
5.0
4.5
4.0
3.5
3.0
2.5
2.0
1.5
1.0
0.5
0.0
-0.5
-1.0
f1 (ppm)

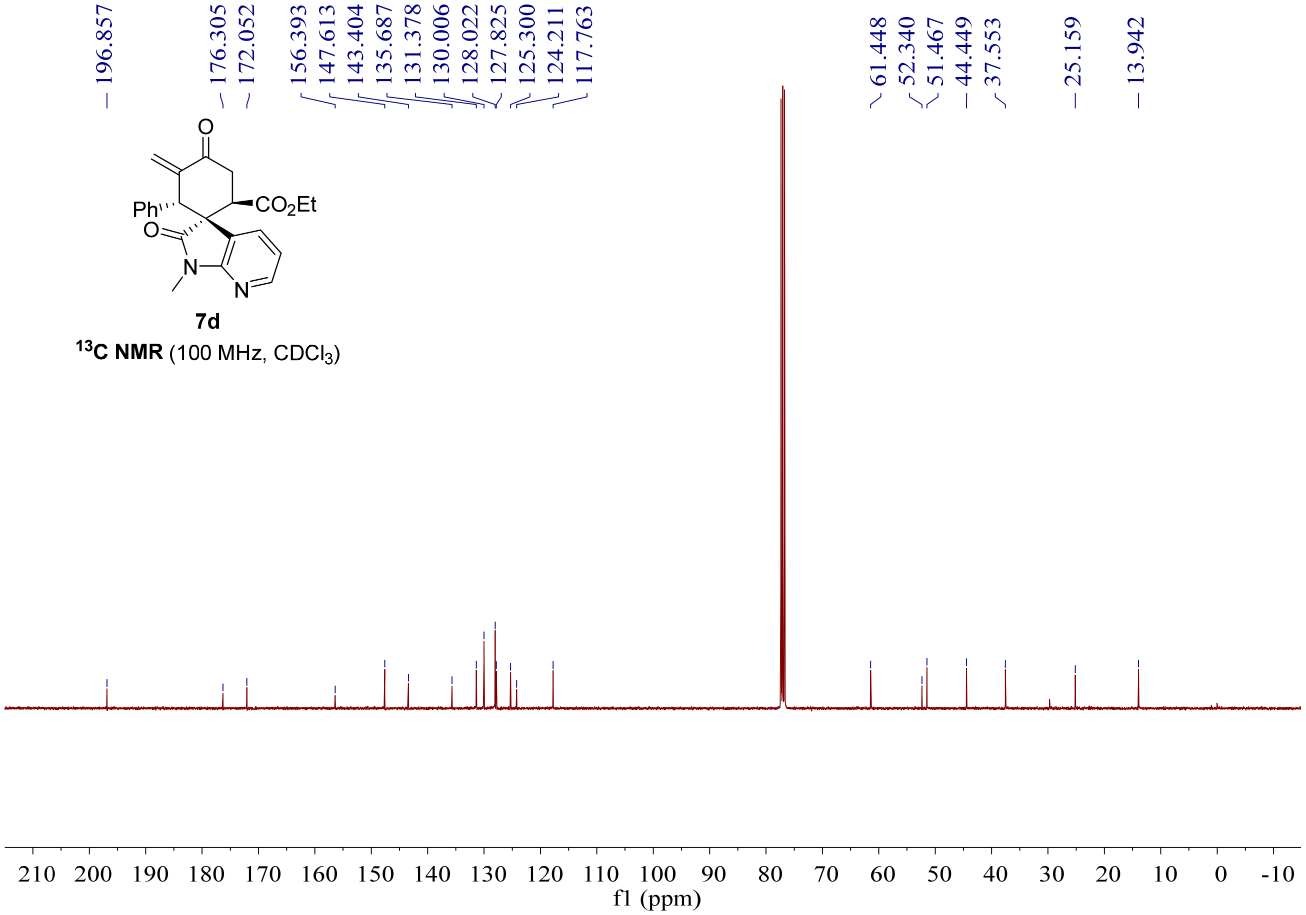
196.857
176.305
172.052
156.393
147.613
143.404
135.687
131.378
130.006
128.022
127.825
125.300
124.211
117.763
61.448
52.340
51.467
44.449
37.553
25.159
13.942
O
Ph
CO2Et
O
N
N
7d
13C NMR (100 MHz, CDCl3)
210
200
190
180
170
160
150
140
130
120
110
100
90
80
70
60
50
40
30
20
10
0
-10
f1 (ppm)

Daicel Chiral IE Column, *i*PrOH/*n*-hexane = 40/60, 1.0 mL/min.

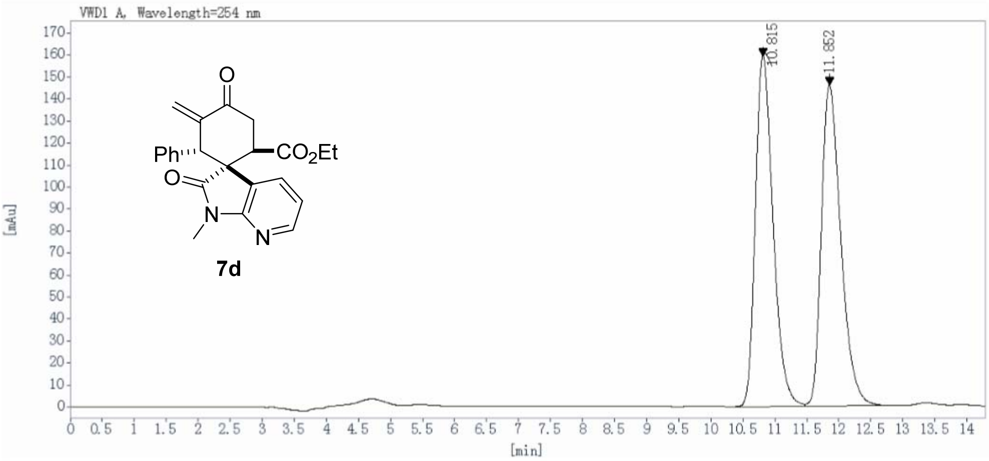

Ret Time [min]	Peak Type	Width [min]	Height [mAU]	Area [mAU*s]	Area [%]
10.815	BV	0.30	159.1865	3060.4221	49.9145
11.852	VB	0.32	145.7001	3070.9124	50.0855
			Totals:	6131.3345	100.0000

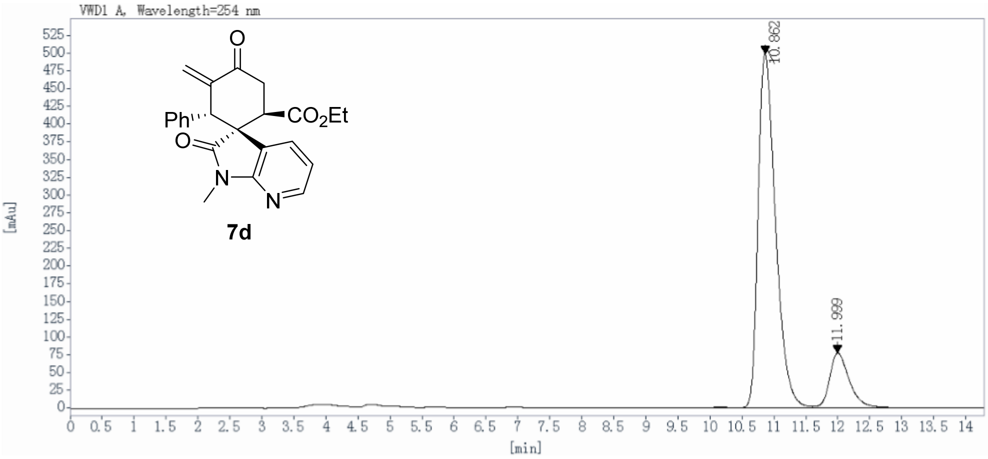

Ret Time [min]	Peak Type	Width [min]	Height [mAU]	Area [mAU*s]	Area [%]
10.862	VV R	0.29	498.4571	9463.8027	84.9830
11.999	VB	0.33	77.3378	1672.3156	15.0170
			Totals:	11136.1183	100.0000

HRMS (ESI-TOF) m/z: $[M + Na]^+$ Calcd for $C_{23}H_{22}O_4N_2Na^+$ 413.1472; Found 413.1471.

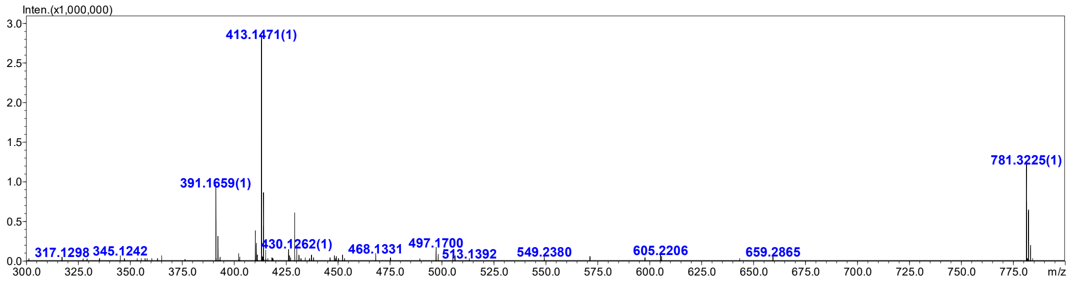

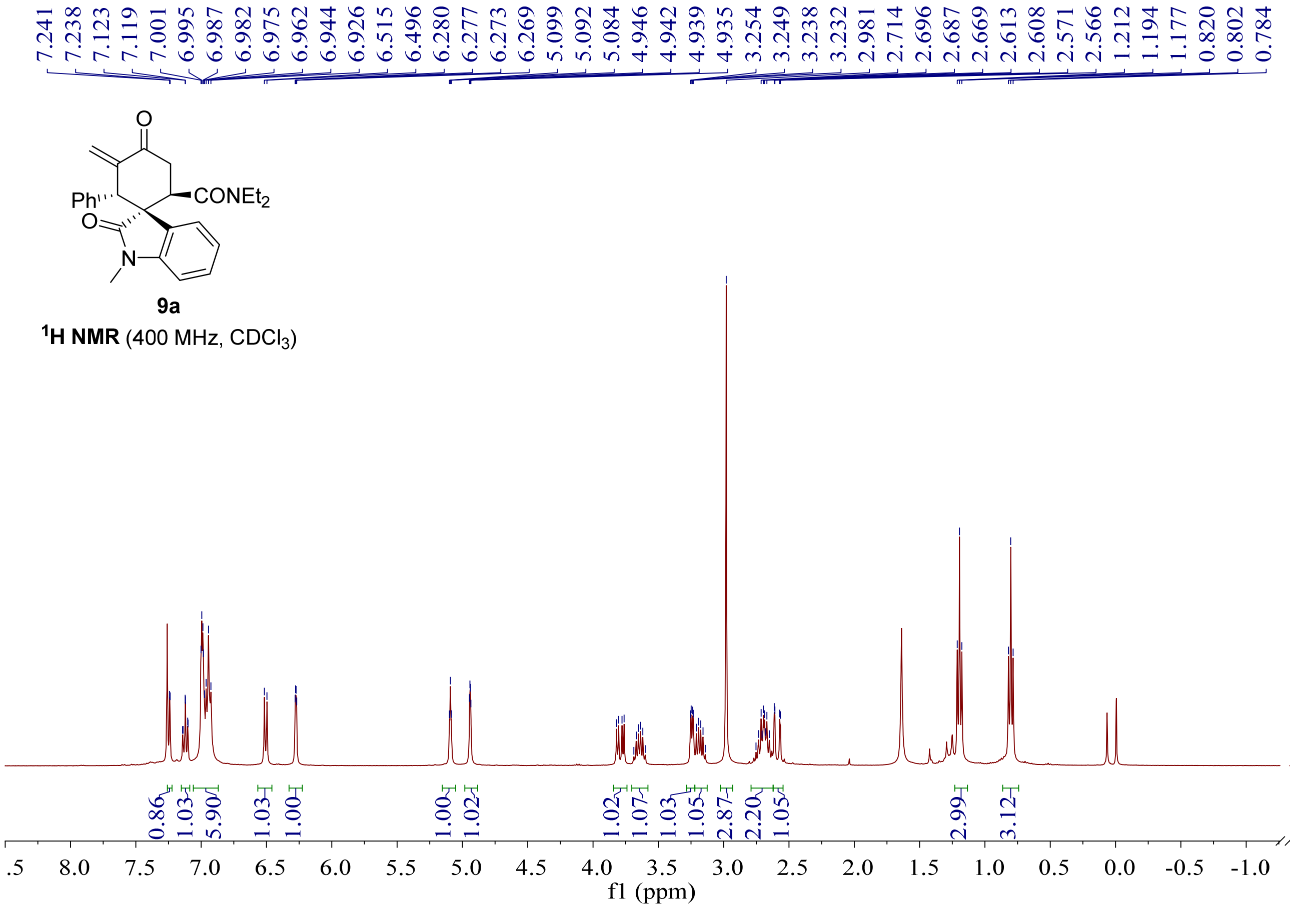
7.241
7.238
7.123
7.119
7.001
6.995
6.987
6.982
6.975
6.962
6.944
6.926
6.515
6.496
6.280
6.277
6.273
6.269
5.099
5.092
5.084
4.946
4.942
4.939
4.935
3.254
3.249
3.238
3.232
2.981
2.714
2.696
2.687
2.669
2.613
2.608
2.571
2.566
1.212
1.194
1.177
0.820
0.802
0.784
O
Ph
CONEt2
O
N
9a
1H NMR (400 MHz, CDCl3)
0.86
1.03
5.90
1.03
1.00
1.00
1.02
1.02
1.07
1.03
1.05
2.87
2.20
1.05
2.99
3.12
.5
8.0
7.5
7.0
6.5
6.0
5.5
5.0
4.5
4.0
3.5
3.0
2.5
2.0
1.5
1.0
0.5
0.0
-0.5
-1.0
f1 (ppm)

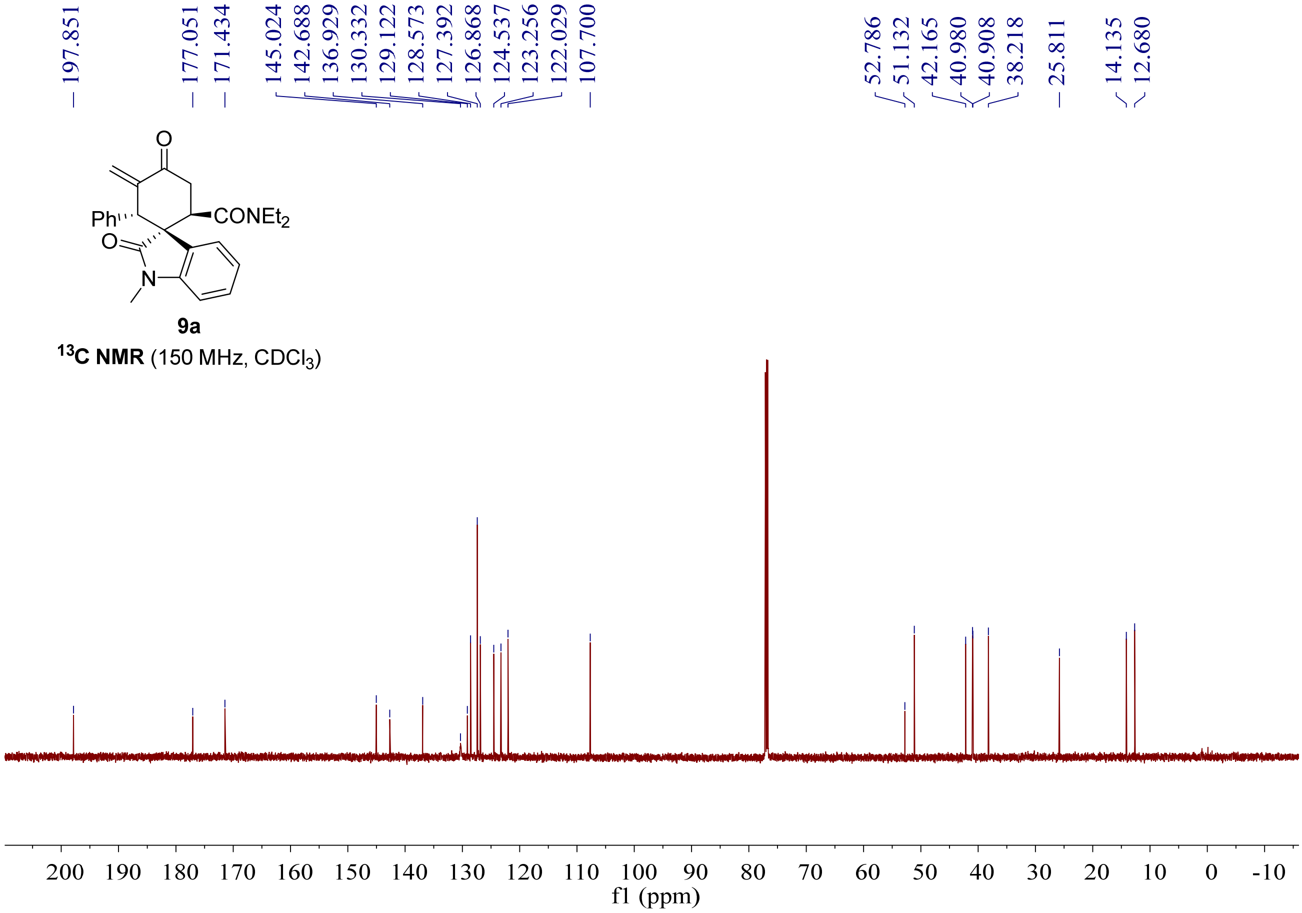
197.851
177.051
171.434
145.024
142.688
136.929
130.332
129.122
128.573
127.392
126.868
124.537
123.256
122.029
107.700
52.786
51.132
42.165
40.980
40.908
38.218
25.811
14.135
12.680
O
Ph
CONEt2
O
N
9a
13C NMR (150 MHz, CDCl3)
200
190
180
170
160
150
140
130
120
110
100
90
80
70
60
50
40
30
20
10
0
-10
f1 (ppm)

Daicel Chiral AD-H Column, *i*PrOH/*n*-hexane = 20/80, 1.0 mL/min.

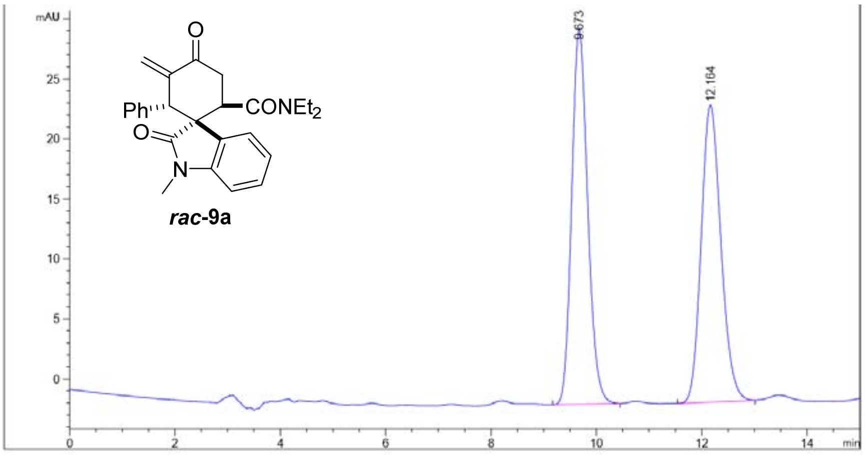

Time (min)	Area (%)
9.67	50.6
12.16	49.4

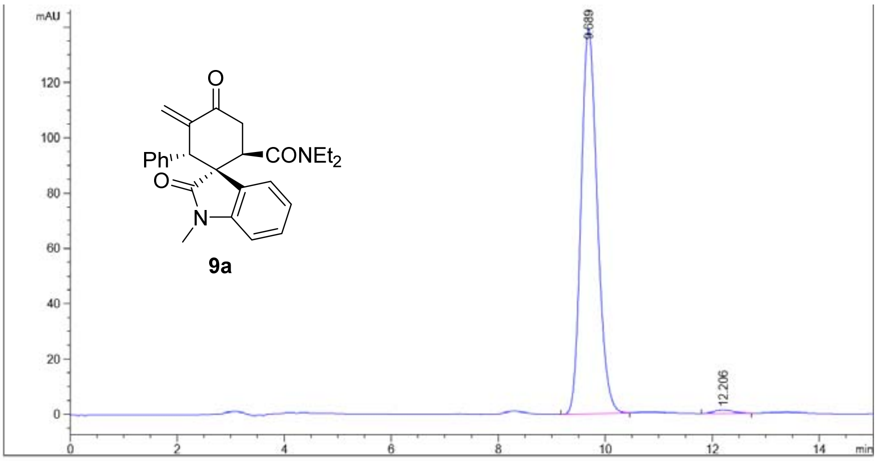

Time (min)	Area (%)
9.69	99.0
12.21	1.0

HRMS (ESI-TOF) m/z: $[M + Na]^+$ Calcd for $C_{26}H_{28}O_3N_2Na^+$ 439.1992; Found 439.1993.

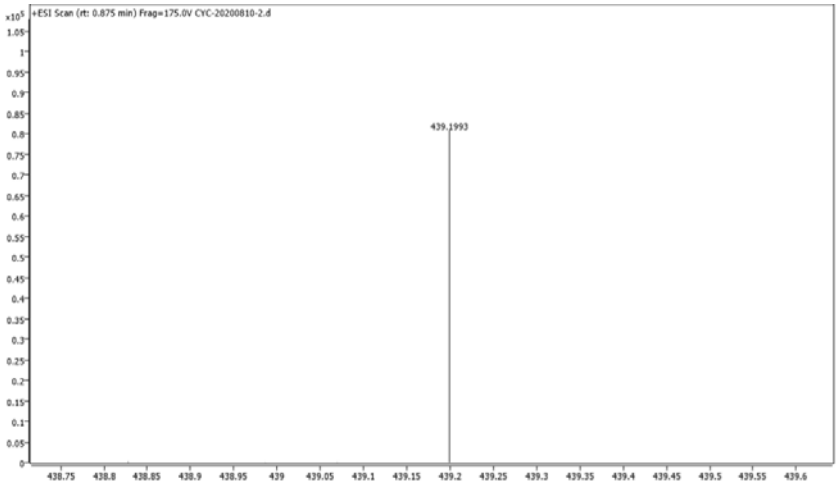

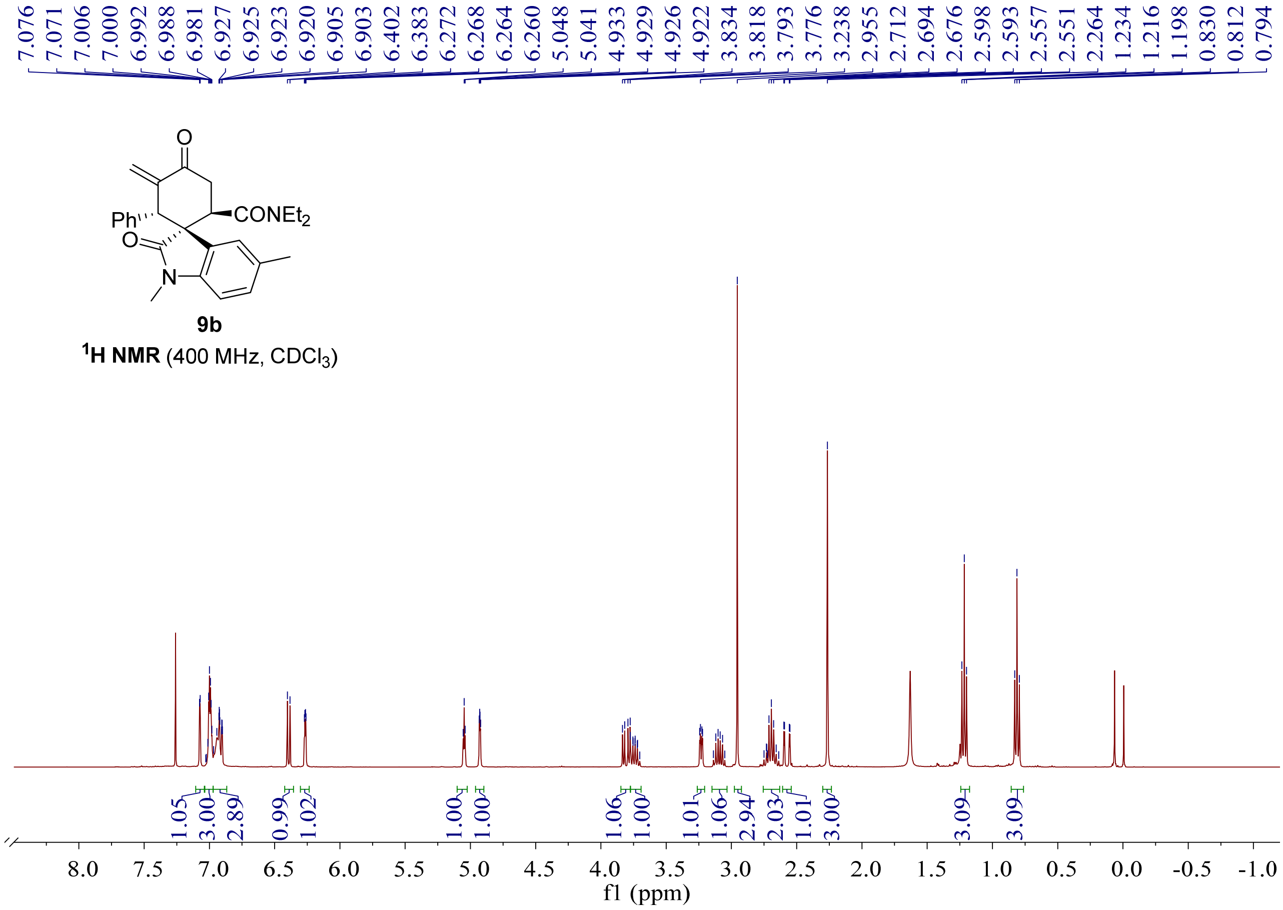
7.076 7.071 7.006 7.000 6.992 6.988 6.981 6.927 6.925 6.923 6.920 6.905 6.903 6.402 6.383 6.272 6.268 6.264 6.260 5.048 5.041 4.933 4.929 4.926 4.922 3.834 3.818 3.793 3.776 3.238 2.955 2.712 2.694 2.676 2.598 2.593 2.557 2.551 2.264 1.234 1.216 1.198 0.830 0.812 0.794
O
CONEt2
Ph
O
N
9b
1H NMR (400 MHz, CDCl3)
1.05 3.00 2.89 0.99 1.02 1.00 1.00 1.06 1.00 1.01 1.06 2.94 2.03 1.01 3.00 3.09 3.09
8.0 7.5 7.0 6.5 6.0 5.5 5.0 4.5 4.0 3.5 3.0 2.5 2.0 1.5 1.0 0.5 0.0 -0.5 -1.0
f1 (ppm)

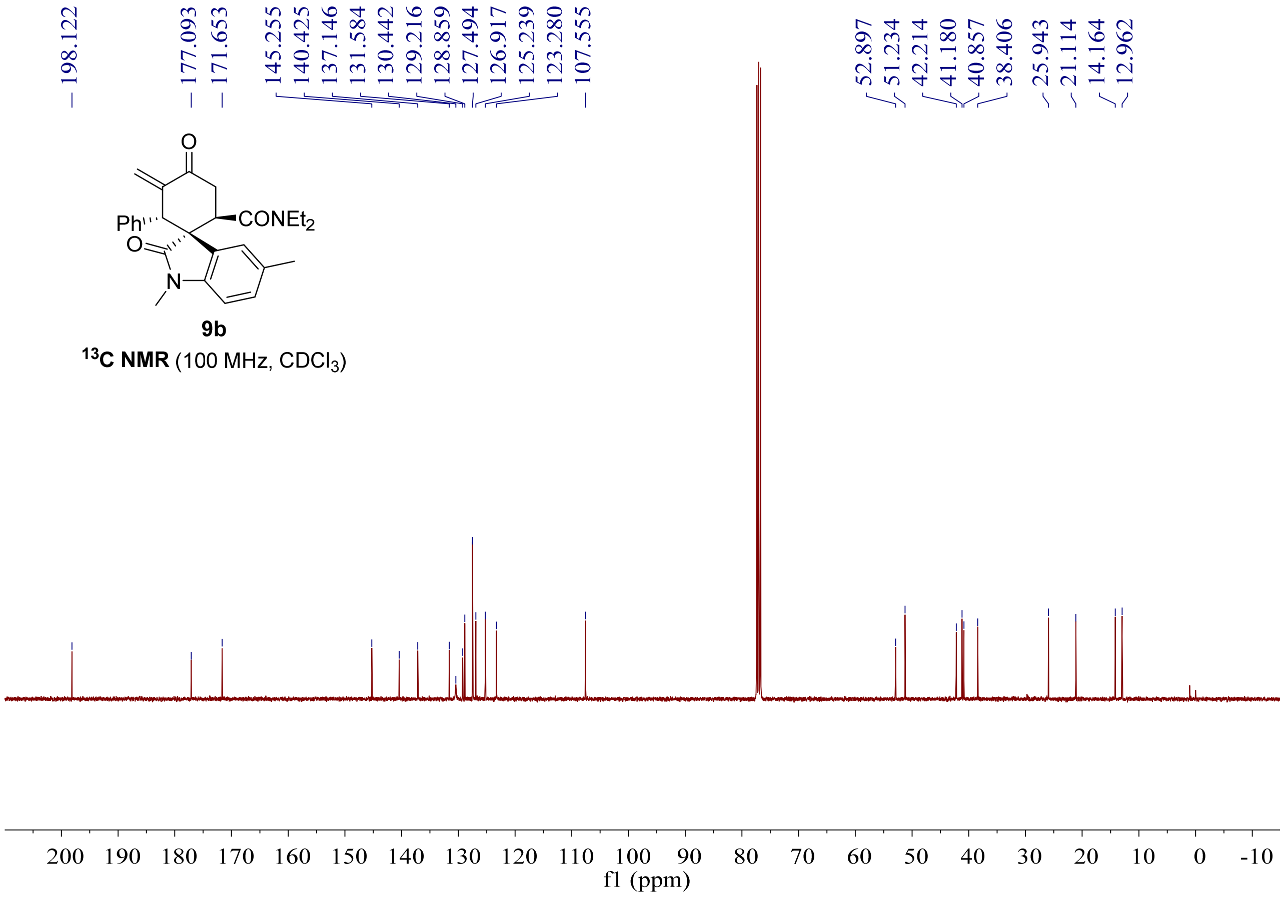
198.122 177.093 171.653 145.255 140.425 137.146 131.584 130.442 129.216 128.859 127.494 126.917 125.239 123.280 107.555 52.897 51.234 42.214 41.180 40.857 38.406 25.943 21.114 14.164 12.962
O
CONEt2
Ph
O
N
9b
13C NMR (100 MHz, CDCl3)
200 190 180 170 160 150 140 130 120 110 100 90 80 70 60 50 40 30 20 10 0 -10
f1 (ppm)

Daicel Chiral AD-H Column, *i*PrOH/*n*-hexane = 10/90, 1.0 mL/min.

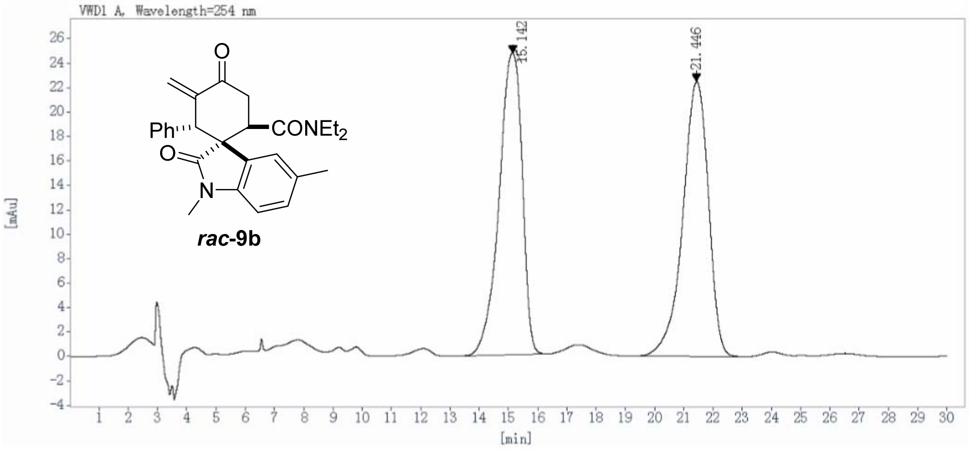

Ret Time [min]	Peak Type	Width [min]	Height [mAU]	Area [mAU*s]	Area [%]
15.142	BB	0.87	24.6910	1358.6678	49.7144
21.446	BB	0.95	22.5011	1374.2805	50.2856
			Totals:	2732.9484	100.0000

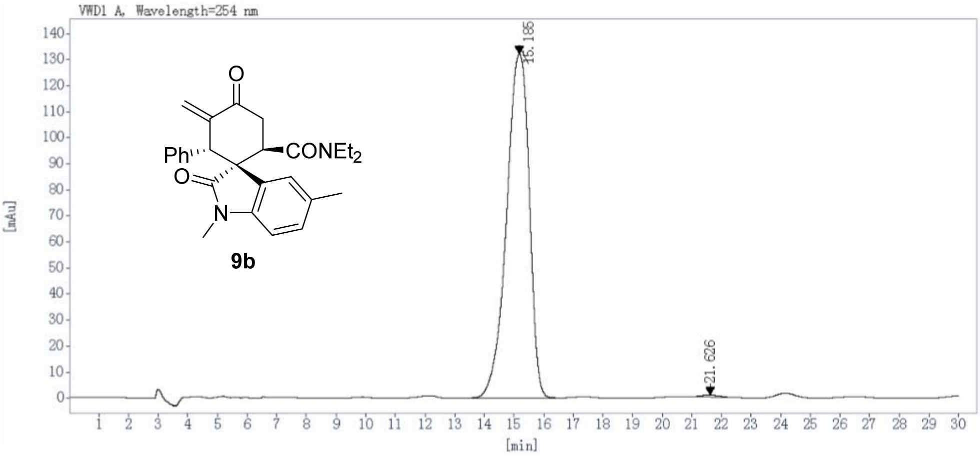

Ret Time [min]	Peak Type	Width [min]	Height [mAU]	Area [mAU*s]	Area [%]
15.185	BB	0.84	132.1888	7047.9160	99.5155
21.626	MM	0.75	0.7616	34.3117	0.4845
			Totals:	7082.2277	100.0000

HRMS (ESI-TOF) m/z: $[M + Na]^+$ Calcd for $C_{27}H_{30}O_3N_2Na^+$ 453.2149; Found 453.2150.

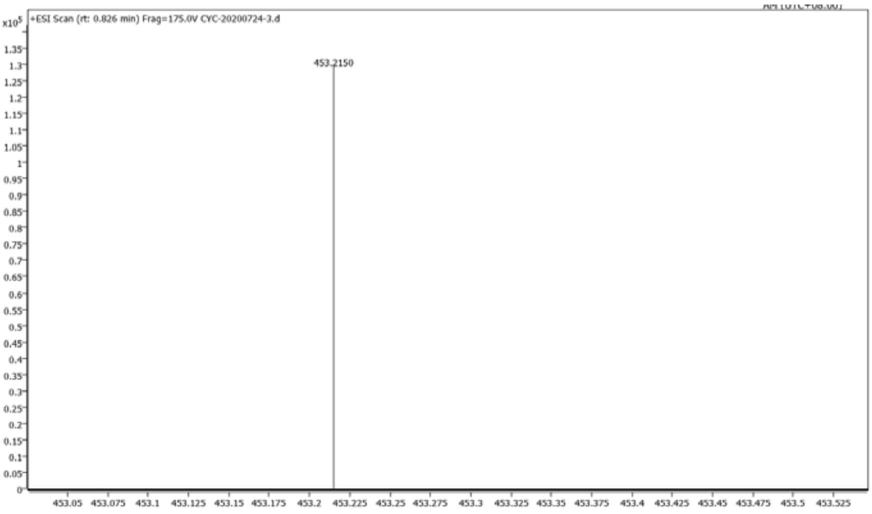

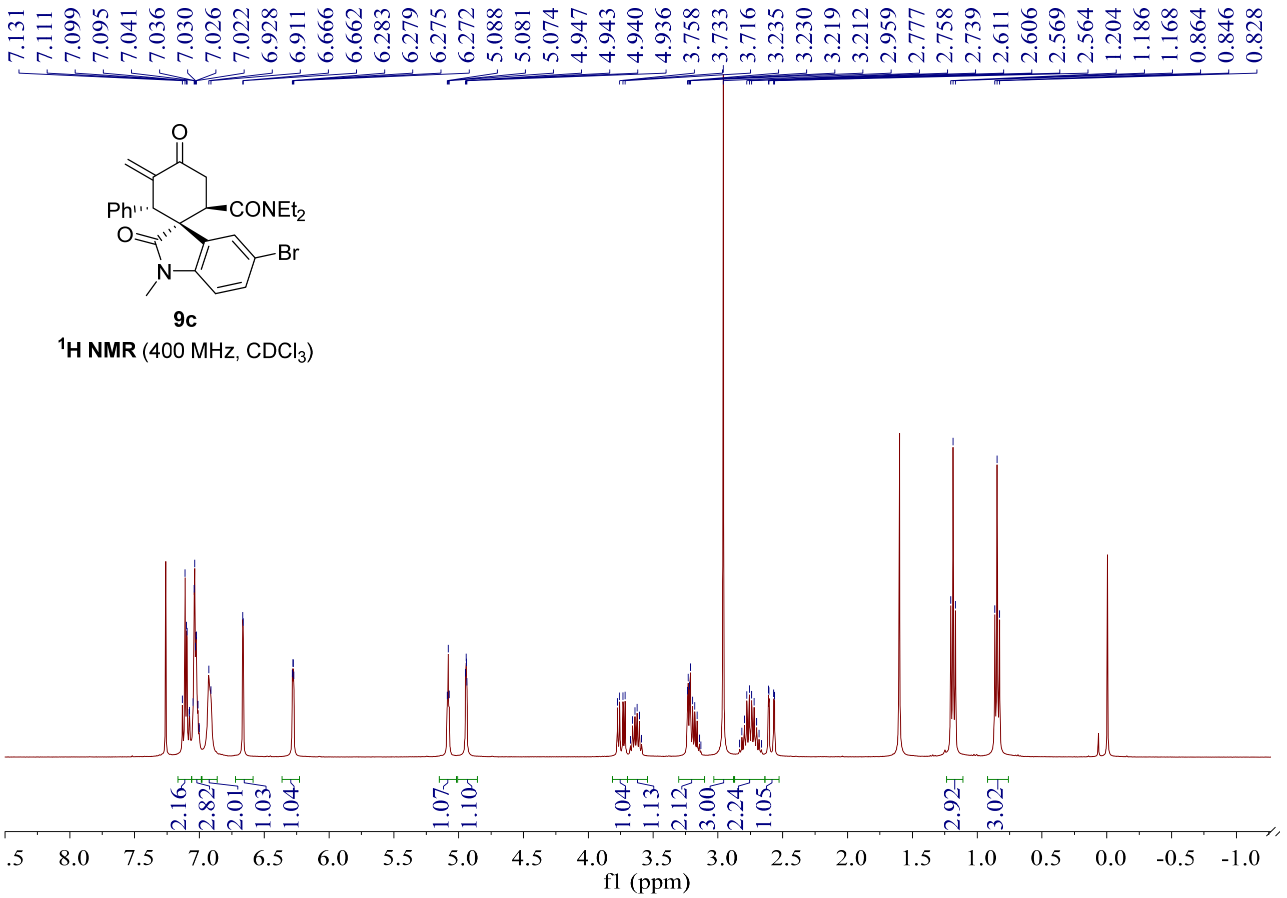
7.131 7.111 7.099 7.095 7.041 7.036 7.030 7.026 7.022 6.928 6.911 6.666 6.662 6.283 6.279 6.275 6.272 5.088 5.081 5.074 4.947 4.943 4.940 4.936 3.758 3.733 3.716 3.235 3.230 3.219 3.212 2.959 2.777 2.758 2.739 2.611 2.606 2.569 2.564 1.204 1.186 1.168 0.864 0.846 0.828
Ph
CONEt2
O
Br
N
9c
1H NMR (400 MHz, CDCl3)
2.16 2.82 2.01 1.03 1.04 1.07 1.10 1.04 1.13 2.12 3.00 2.24 1.05 2.92 3.02
.5 8.0 7.5 7.0 6.5 6.0 5.5 5.0 4.5 4.0 3.5 3.0 2.5 2.0 1.5 1.0 0.5 0.0 -0.5 -1.0
f1 (ppm)

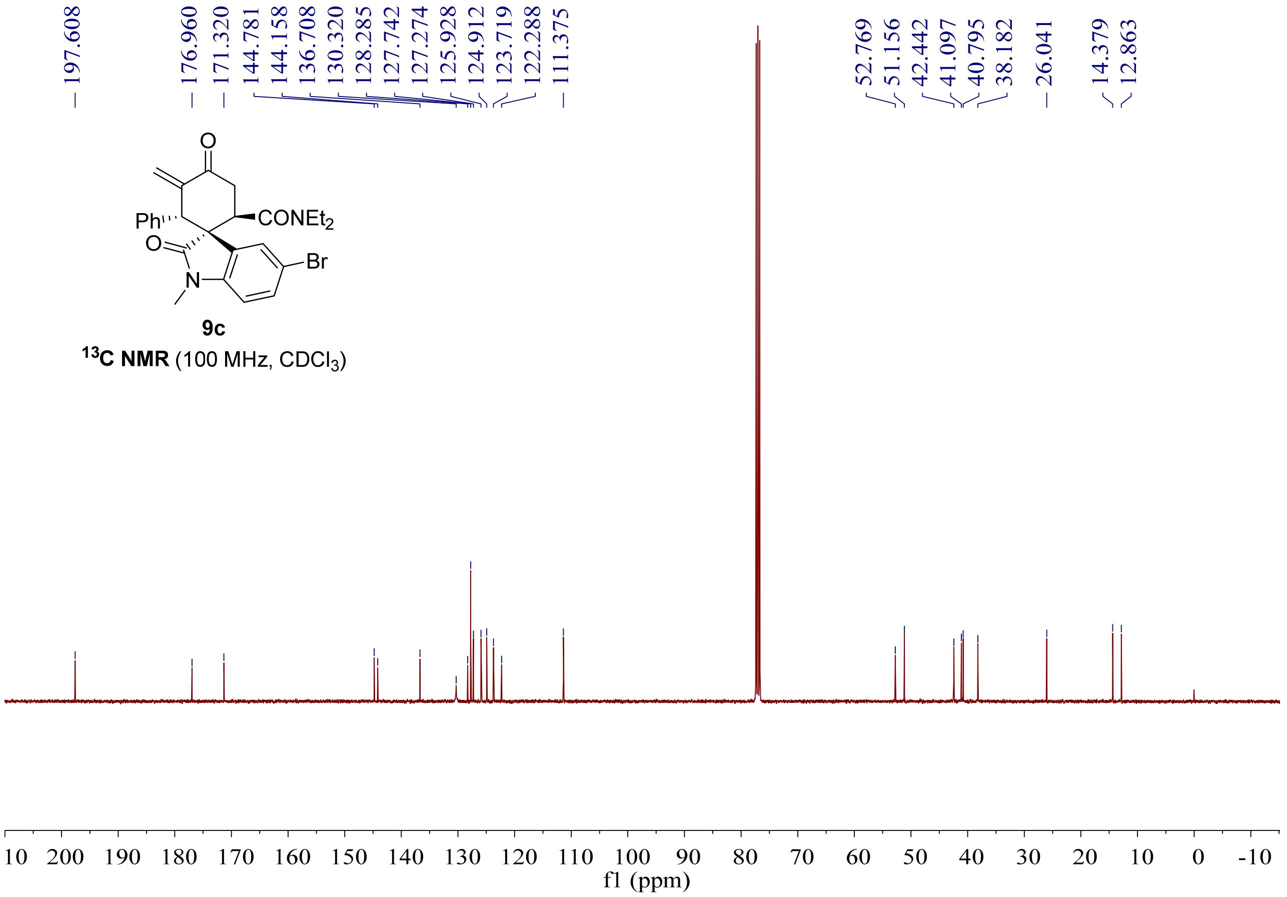
197.608 176.960 171.320 144.781 144.158 136.708 130.320 128.285 127.742 127.274 125.928 124.912 123.719 122.288 111.375 52.769 51.156 42.442 41.097 40.795 38.182 26.041 14.379 12.863
Ph
CONEt2
O
Br
N
9c
13C NMR (100 MHz, CDCl3)
10 200 190 180 170 160 150 140 130 120 110 100 90 80 70 60 50 40 30 20 10 0 -10
f1 (ppm)

Daicel Chiral IE Column, *i*PrOH/*n*-hexane = 40/60, 1.0 mL/min.

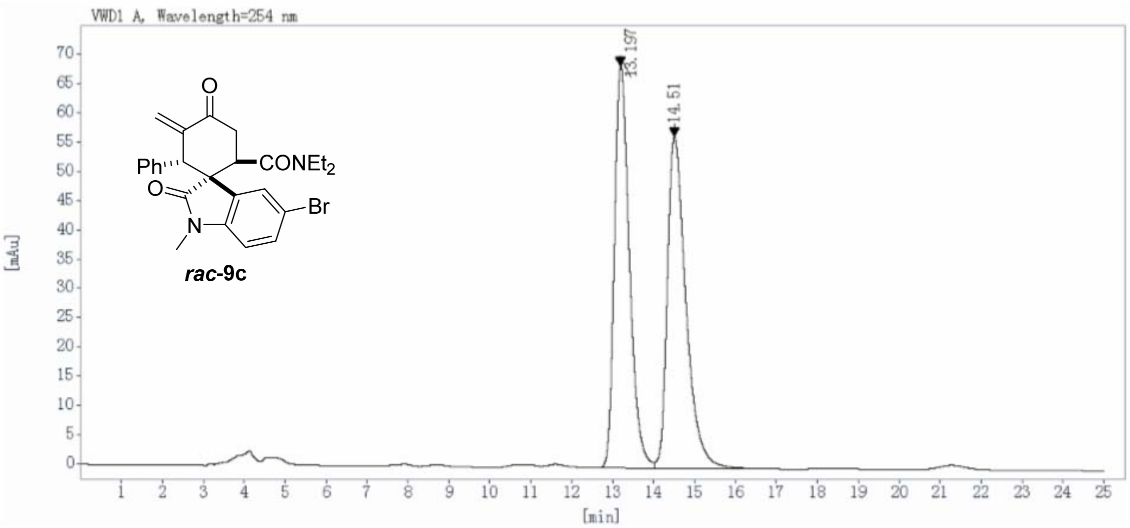

Ret Time [min]	Peak Type	Width [min]	Height [mAU]	Area [mAU*s]	Area [%]
13.197	BV	0.39	68.7610	1753.9299	49.5131
14.510	VB	0.48	56.6560	1788.4222	50.4869
			Totals:	3542.3522	100.0000

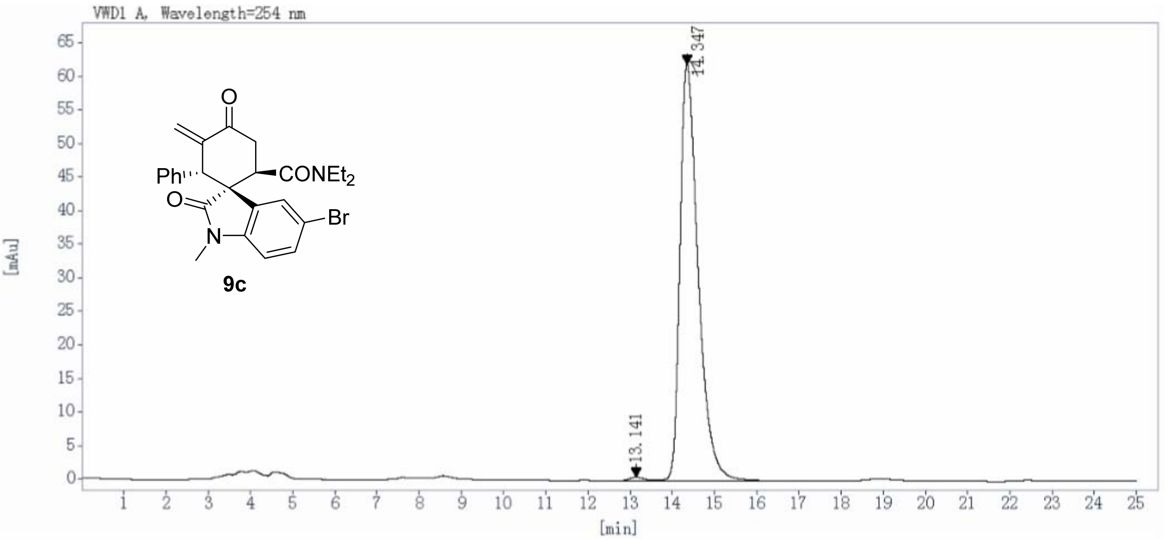

Ret Time [min]	Peak Type	Width [min]	Height [mAU]	Area [mAU*s]	Area [%]
13.141	MM	0.40	0.5282	12.6088	0.6746
14.347	BB	0.46	62.0473	1856.5323	99.3254
			Totals:	1869.1411	100.0000

HRMS (ESI-TOF) m/z: $[M + Na]^+$Calcd for $C_{27}H_{30}O_3N_2Na^+$ 517.1097 (^{79}Br) and 519.1077 (^{81}Br); Found 517.1096 (^{79}Br) and 519.1078 (^{81}Br).

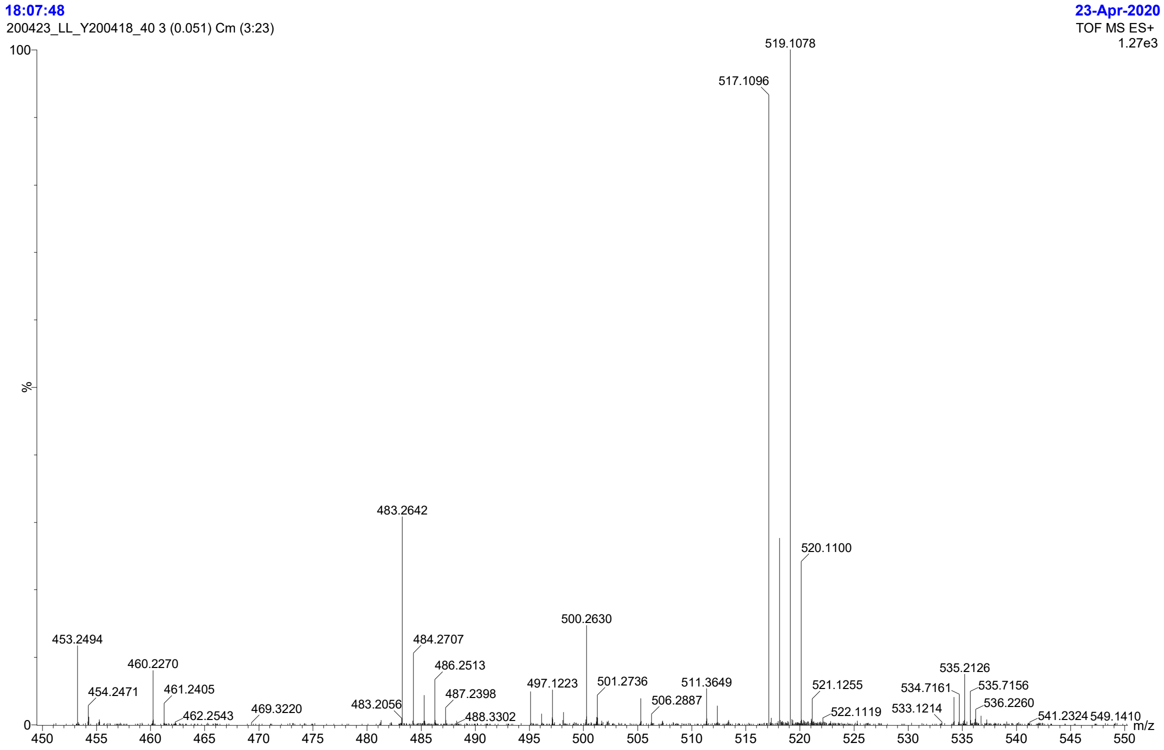

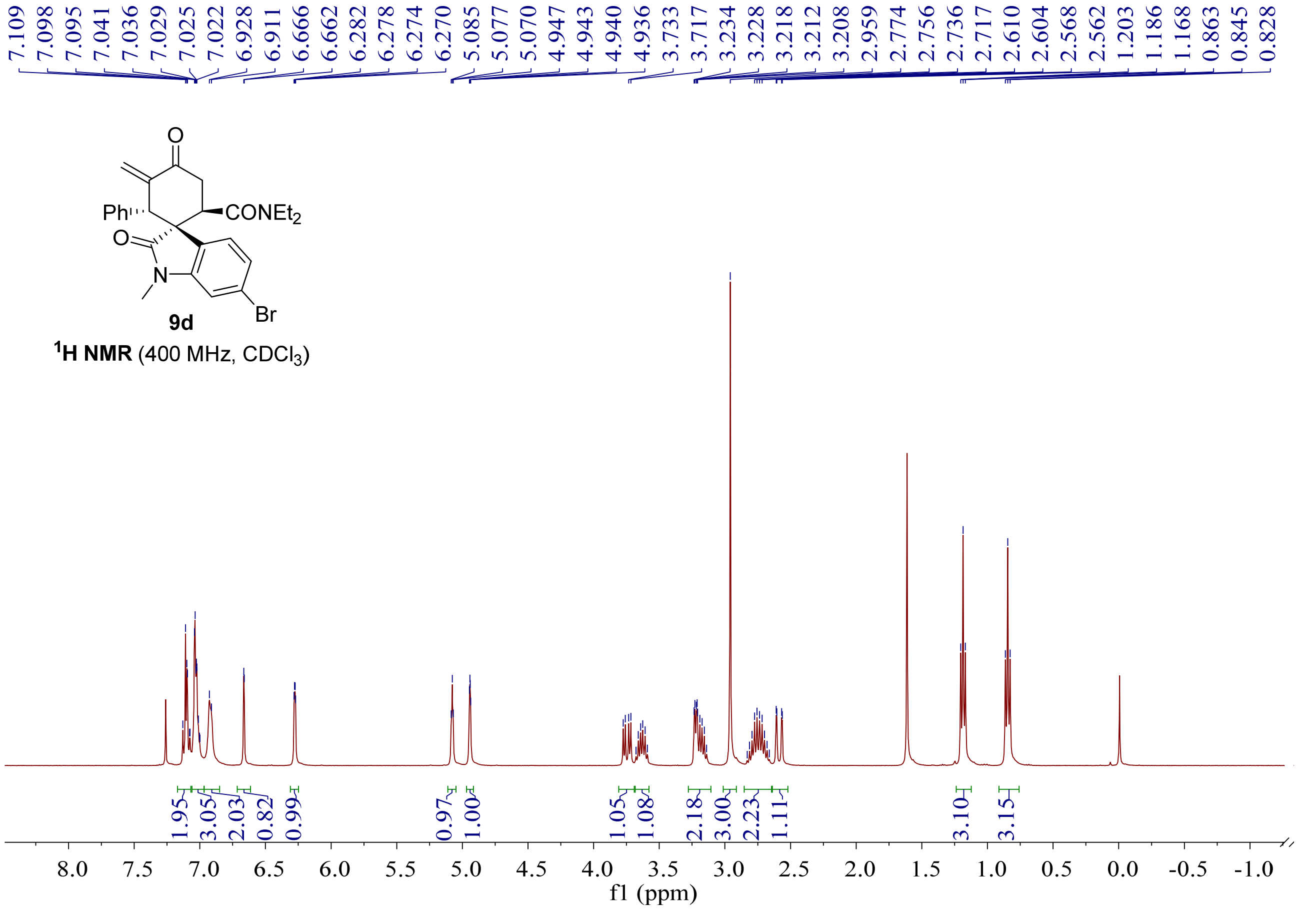

7.109 7.098 7.095 7.041 7.036 7.029 7.025 7.022 6.928 6.911 6.666 6.662 6.282 6.278 6.274 6.270 5.085 5.077 5.070 4.947 4.943 4.940 4.936 3.733 3.717 3.234 3.228 3.218 3.212 3.208 2.959 2.774 2.756 2.736 2.717 2.610 2.604 2.568 2.562 1.203 1.186 1.168 0.863 0.845 0.828
O
Ph
CONEt$_2$
O
N
Br
9d
^{1}H NMR (400 MHz, CDCl$_3$)
1.95 3.05 2.03 0.82 0.99 0.97 1.00 1.05 1.08 2.18 3.00 2.23 1.11 3.10 3.15
8.0 7.5 7.0 6.5 6.0 5.5 5.0 4.5 4.0 3.5 3.0 2.5 2.0 1.5 1.0 0.5 0.0 -0.5 -1.0
f1 (ppm)

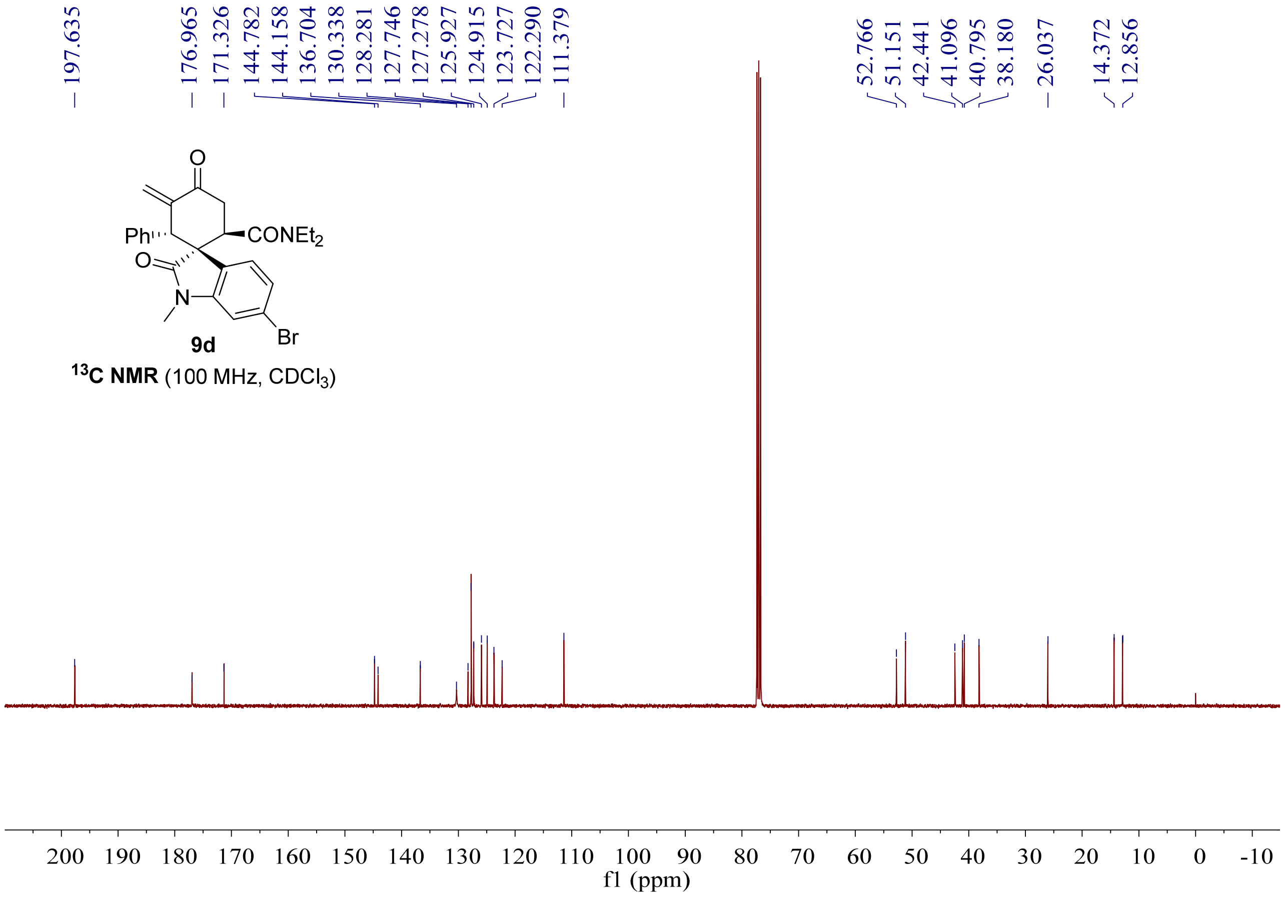

197.635 176.965 171.326 144.782 144.158 136.704 130.338 128.281 127.746 127.278 125.927 124.915 123.727 122.290 111.379 52.766 51.151 42.441 41.096 40.795 38.180 26.037 14.372 12.856
O
Ph
CONEt$_2$
O
N
Br
9d
^{13}C NMR (100 MHz, CDCl$_3$)
200 190 180 170 160 150 140 130 120 110 100 90 80 70 60 50 40 30 20 10 0 -10
f1 (ppm)

Daicel Chiral IE Column, *i*PrOH/*n*-hexane = 40/60, 1.0 mL/min.

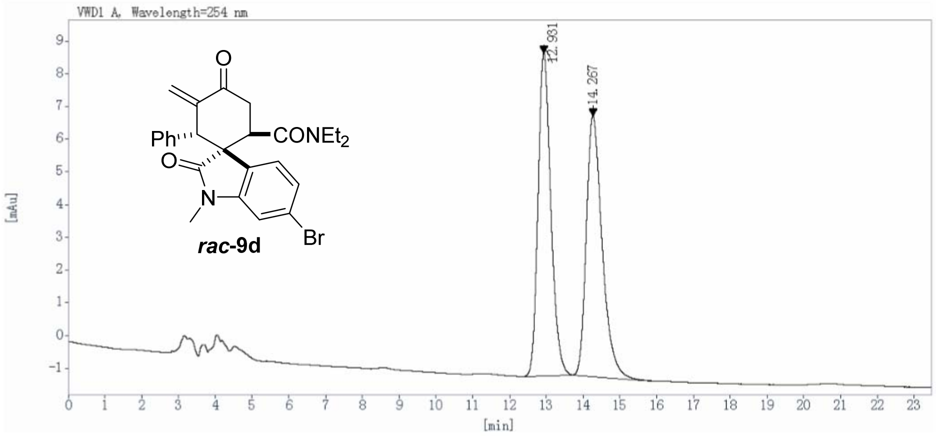

Ret Time [min]	Peak Type	Width [min]	Height [mAU]	Area [mAU*s]	Area [%]
12.931	BB	0.37	9.8647	238.7316	50.0640
14.267	BB	0.45	7.9525	238.1217	49.9360
			Totals:	476.8533	100.0000

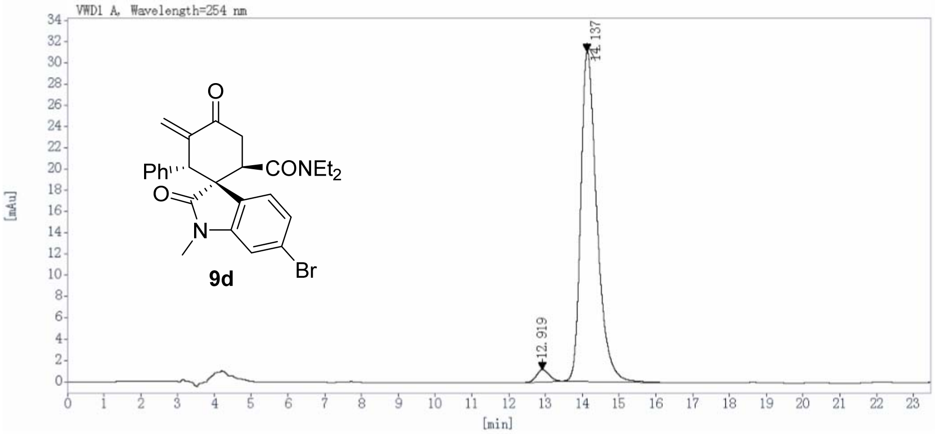

Ret Time [min]	Peak Type	Width [min]	Height [mAU]	Area [mAU*s]	Area [%]
12.919	BB	0.34	1.1345	26.0829	2.6941
14.137	BB	0.46	31.0549	942.0718	97.3059
			Totals:	968.1547	100.0000

HRMS (ESI-TOF) m/z: $[M + Na]^+$ Calcd for $C_{27}H_{30}O_3N_2Na^+$ 517.1097 (^{79}Br) and 519.1077 (^{81}Br); Found 517.1105 (^{79}Br) and 519.1089 (^{81}Br).

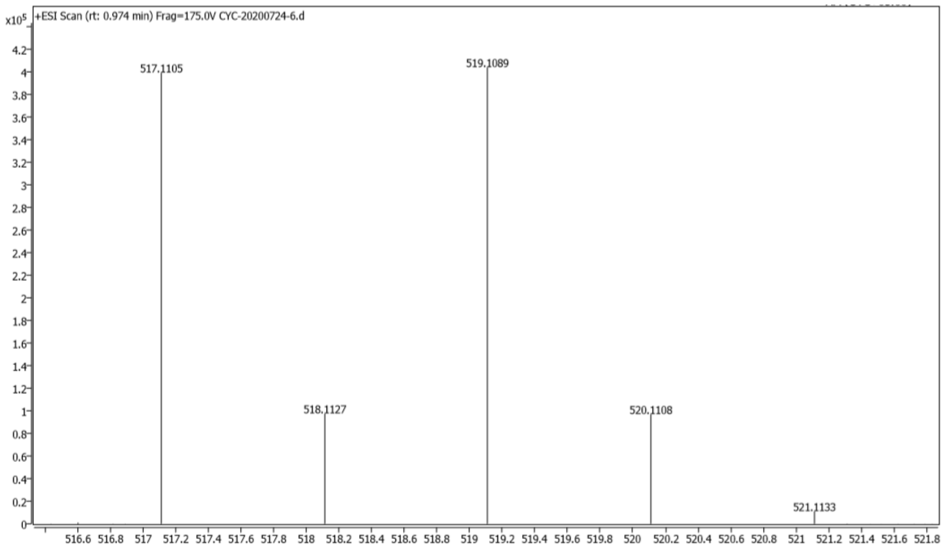

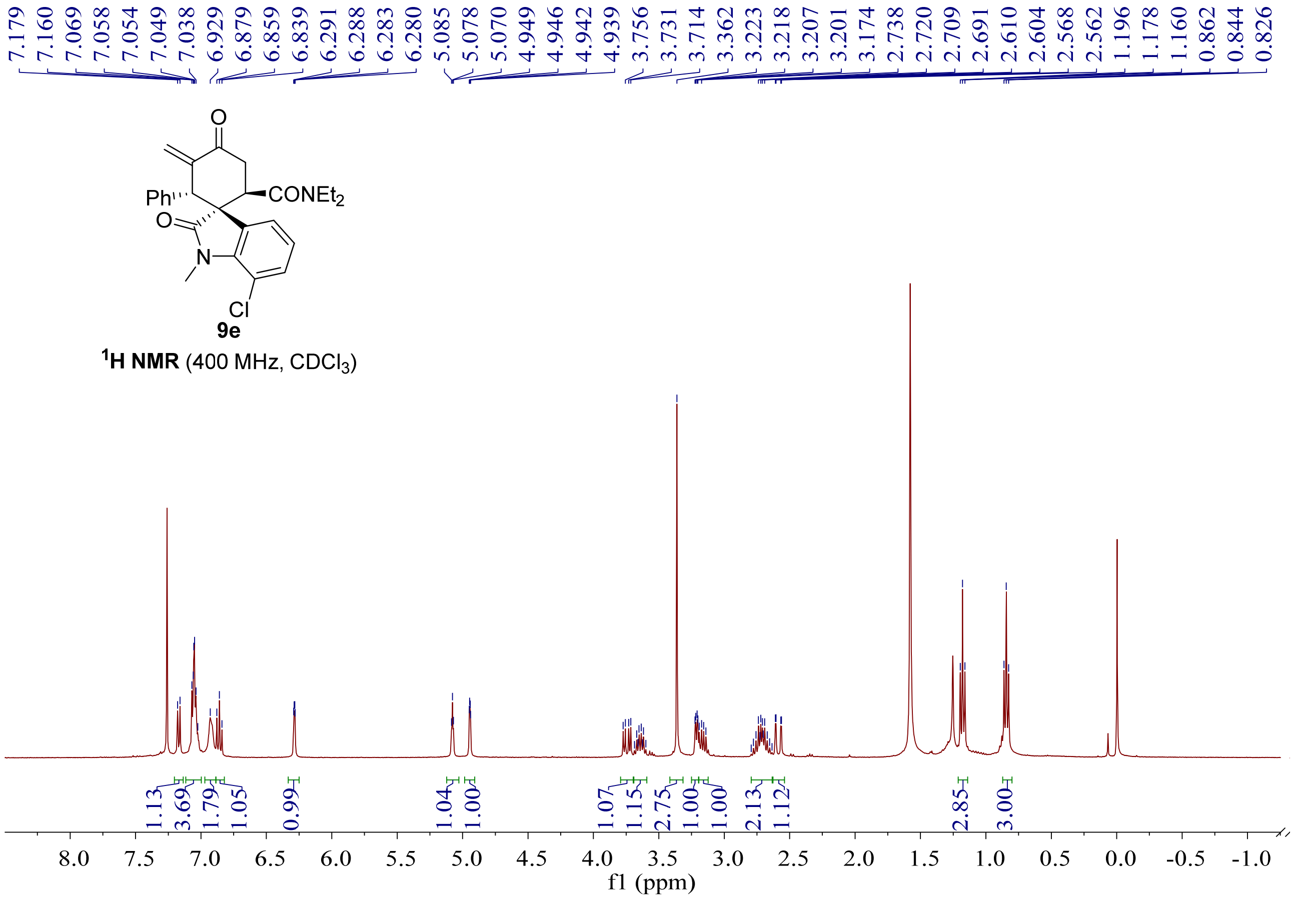

9e

^{1}H NMR (400 MHz, $CDCl_3$)

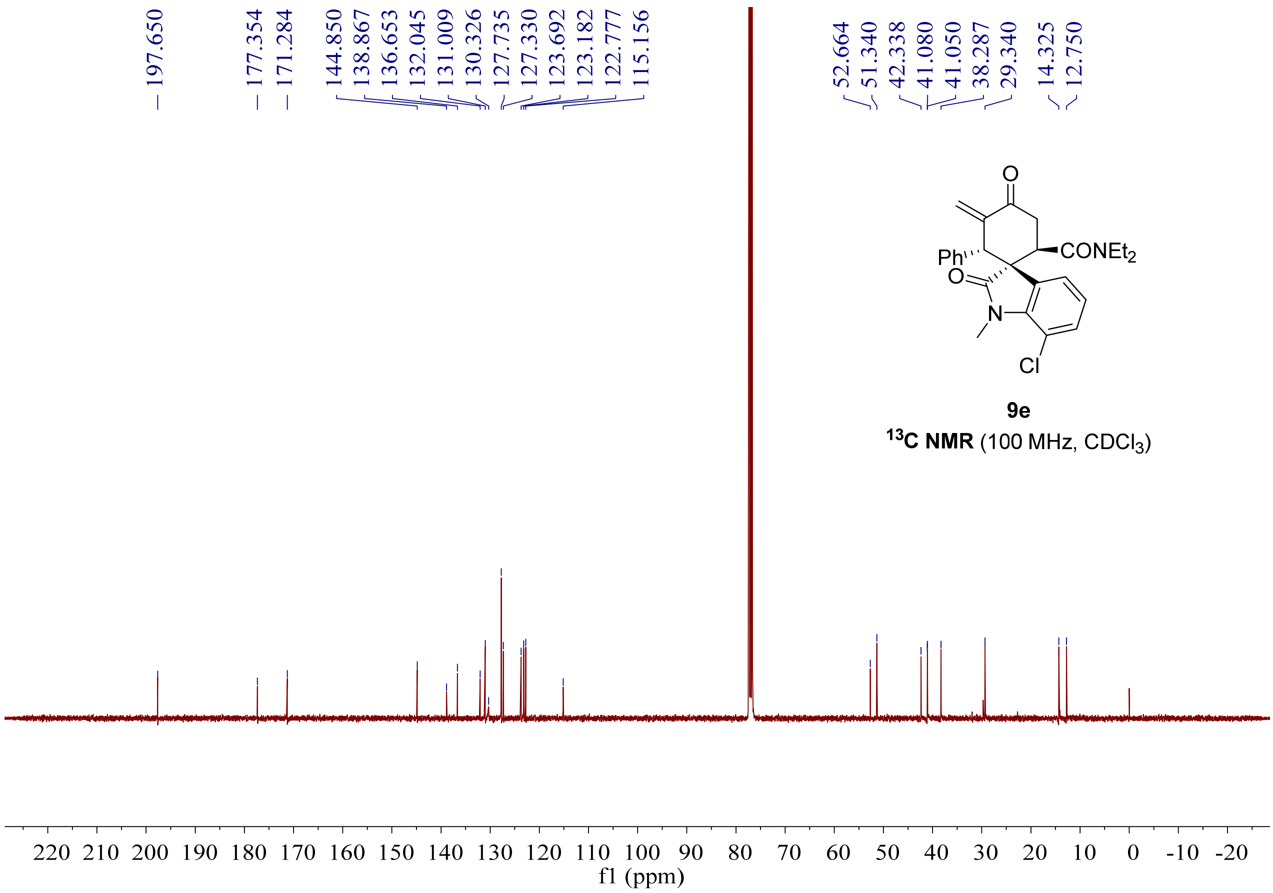

9e

^{13}C NMR (100 MHz, $CDCl_3$)

Daicel Chiral AD-H Column, *i*PrOH/*n*-hexane = 10/90, 1.0 mL/min.

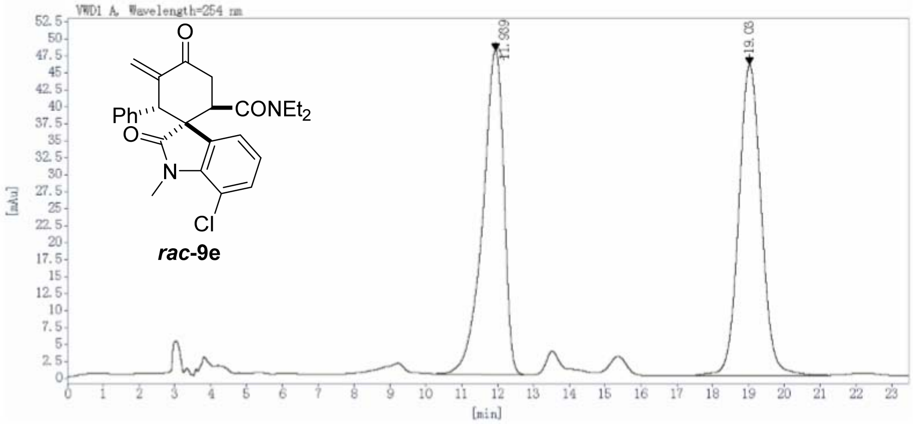

Ret Time [min]	Peak Type	Width [min]	Height [mAU]	Area [mAU*s]	Area [%]
11.939	BB	0.62	47.7095	1954.5952	49.4542
19.030	BB	0.68	45.7661	1997.7424	50.5458
			Totals:	3952.3376	100.0000

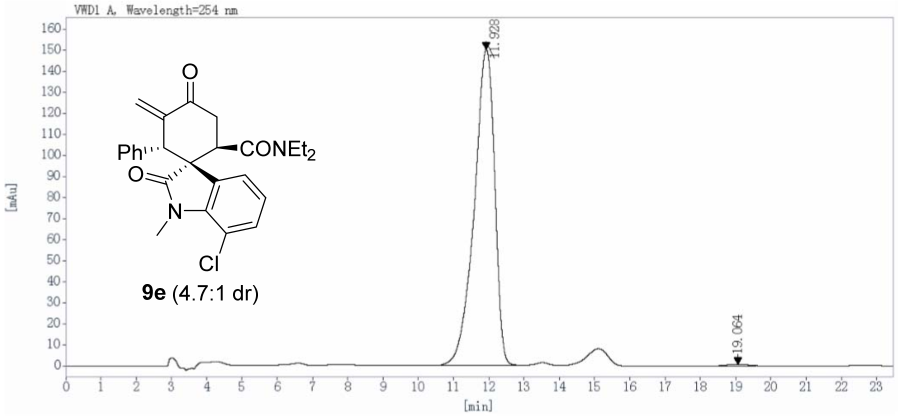

Ret Time [min]	Peak Type	Width [min]	Height [mAU]	Area [mAU*s]	Area [%]
11.928	BBA	0.62	150.3018	6050.2354	99.4332
19.064	MM	0.69	0.8342	34.4856	0.5668
			Totals:	6084.7210	100.0000

HRMS (ESI-TOF) m/z: $[M + H]^+$ Calcd for $C_{26}H_{28}ClO_3N_2^+$ 451.1783 (^{35}Cl) and 453.1753 (^{37}Cl); Found 451.1776 (^{35}Cl) and 453.1767 (^{37}Cl).

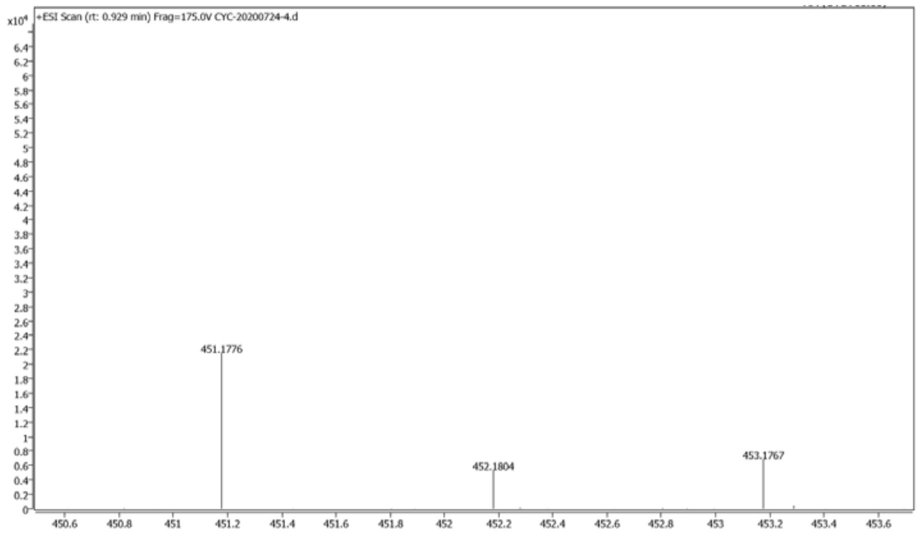

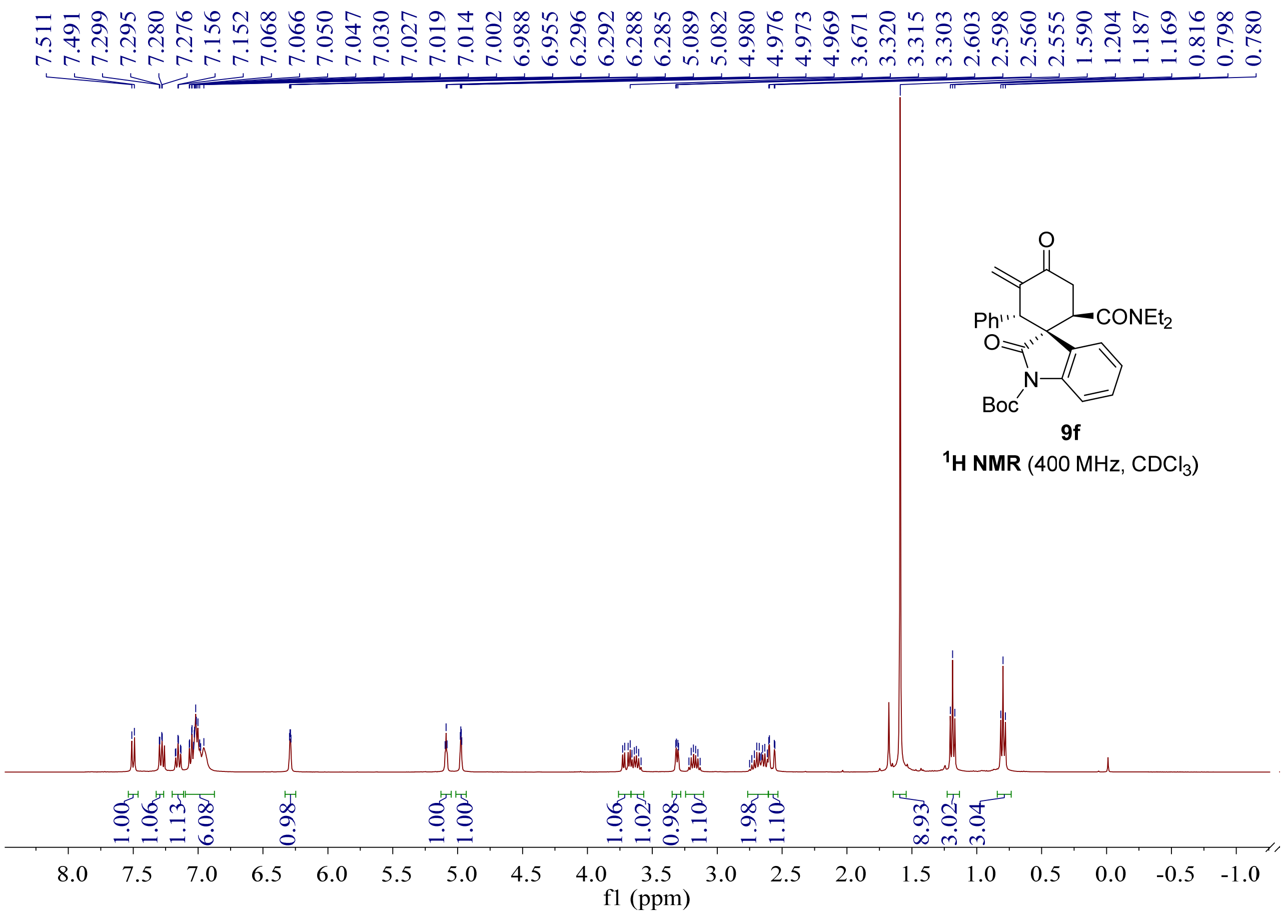
7.511 7.491 7.299 7.295 7.280 7.276 7.156 7.152 7.068 7.066 7.050 7.047 7.030 7.027 7.019 7.014 7.002 6.988 6.955 6.296 6.292 6.288 6.285 5.089 5.082 4.980 4.976 4.973 4.969 3.671 3.320 3.315 3.303 2.603 2.598 2.560 2.555 1.590 1.204 1.187 1.169 0.816 0.798 0.780
O
Ph
CONEt2
O
N
Boc
9f
1H NMR (400 MHz, CDCl3)
1.00 1.06 1.13 6.08 0.98 1.00 1.00 1.06 1.02 0.98 1.10 1.98 1.10 8.93 3.02 3.04
8.0 7.5 7.0 6.5 6.0 5.5 5.0 4.5 4.0 3.5 3.0 2.5 2.0 1.5 1.0 0.5 0.0 -0.5 -1.0
f1 (ppm)

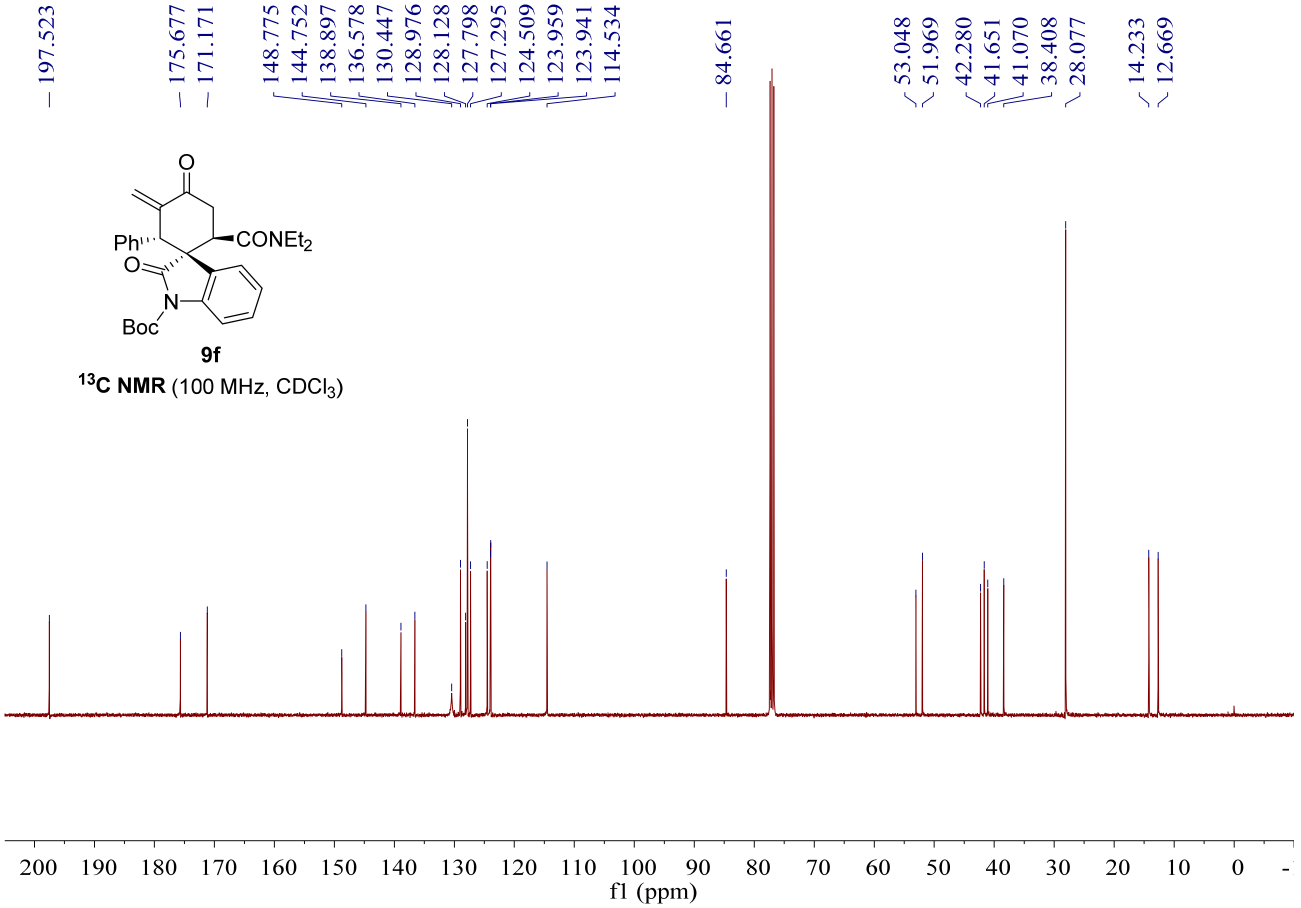
197.523
175.677 171.171
148.775 144.752 138.897 136.578 130.447 128.976 128.128 127.798 127.295 124.509 123.959 123.941 114.534
84.661
53.048 51.969 42.280 41.651 41.070 38.408 28.077 14.233 12.669
O
Ph
CONEt2
O
N
Boc
9f
13C NMR (100 MHz, CDCl3)
200 190 180 170 160 150 140 130 120 110 100 90 80 70 60 50 40 30 20 10 0
f1 (ppm)

Daicel Chiral IB Column, *i*PrOH/*n*-hexane = 20/80, 1.0 mL/min.

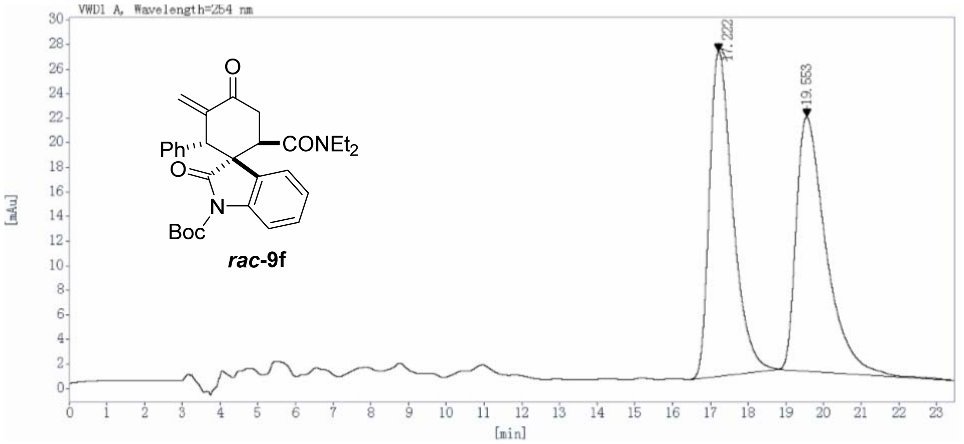

Ret Time [min]	Peak Type	Width [min]	Height [mAU]	Area [mAU*s]	Area [%]
17.222	BB	0.66	26.4285	1136.3441	50.0799
19.553	BB	0.82	20.6629	1132.7168	49.9201
			Totals:	2269.0609	100.0000

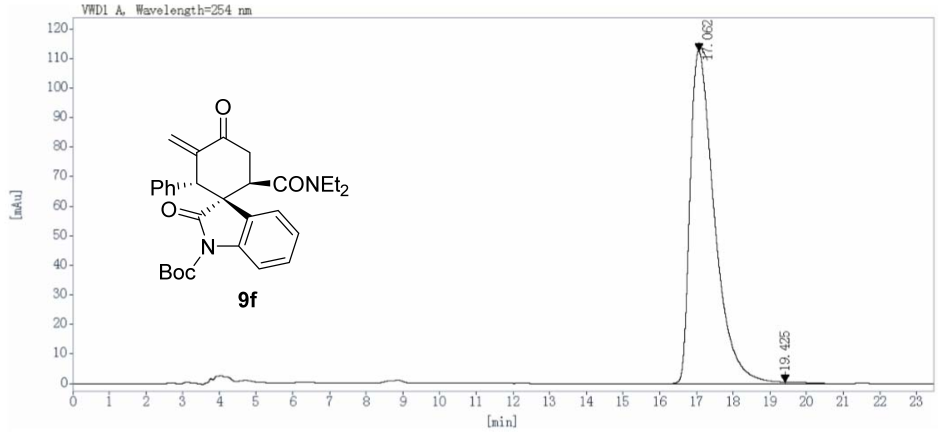

Ret Time [min]	Peak Type	Width [min]	Height [mAU]	Area [mAU*s]	Area [%]
17.062	MF	0.77	112.7366	5231.2627	99.5229
19.425	FM	0.72	0.5798	25.0806	0.4771
			Totals:	5256.3433	100.0000

HRMS (ESI-TOF) m/z: $[M + Na]^+$ Calcd for $C_{30}H_{34}O_5N_2Na^+$ 525.2360; Found 525.2360.

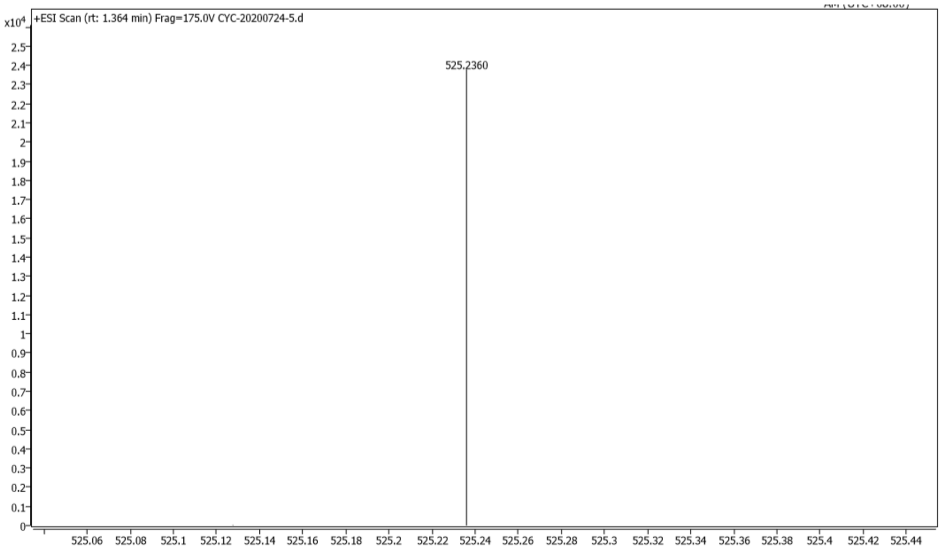

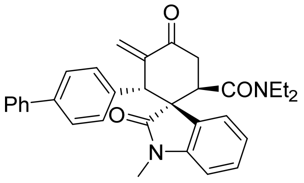

9g

^{1}H NMR (600 MHz, $CDCl_3$)

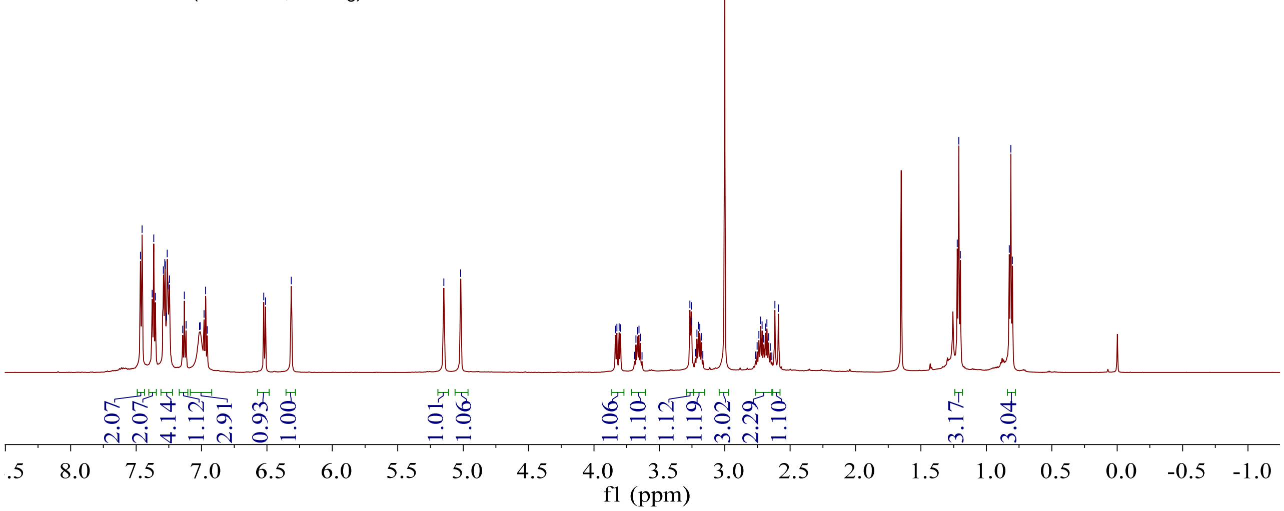

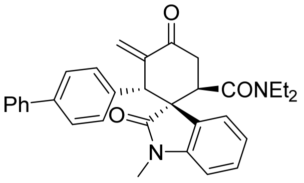

9g

^{13}C NMR (100 MHz, $CDCl_3$)

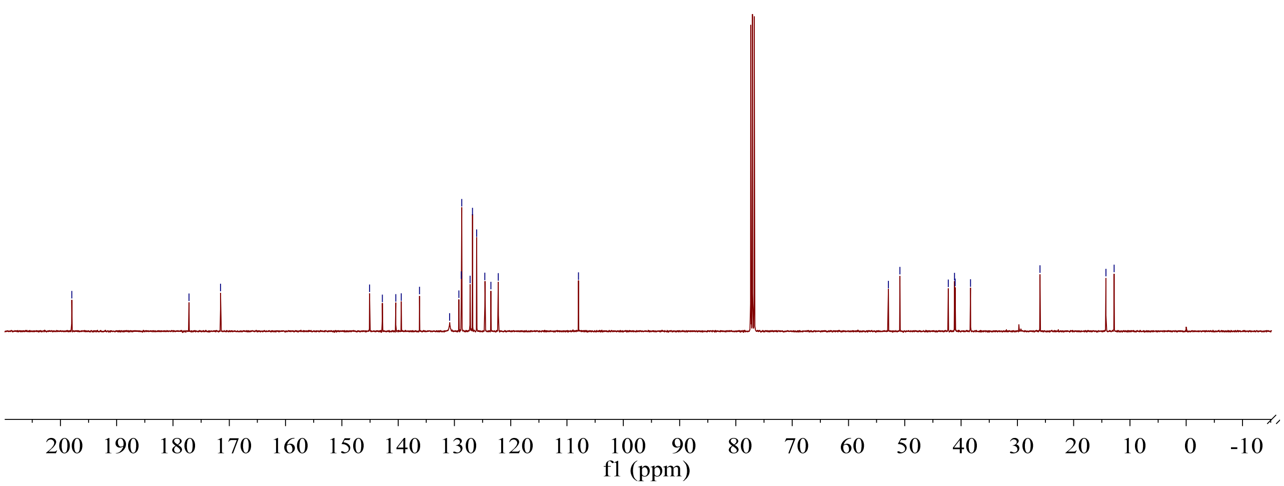

Daicel Chiral AD-H Column, *i*PrOH/*n*-hexane = 20/80, 1.0 mL/min

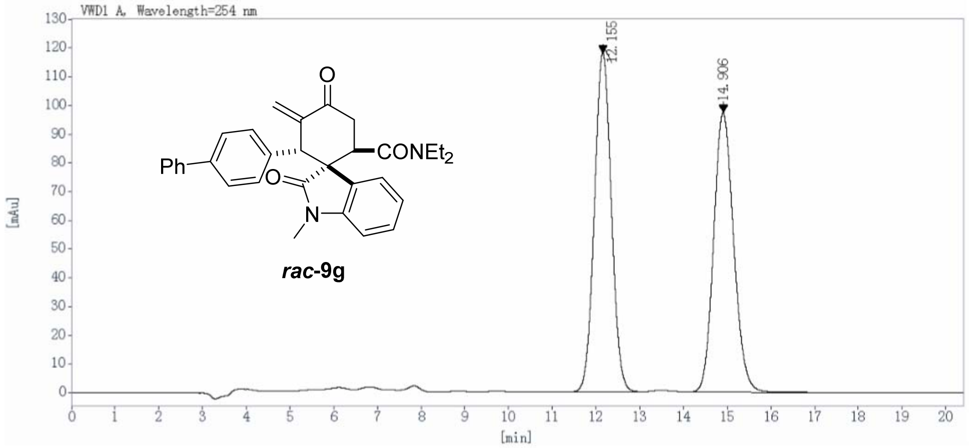

Ret Time [min]	Peak Type	Width [min]	Height [mAU]	Area [mAU*s]	Area [%]
12.155	BB	0.42	117.8931	3171.8823	50.6359
14.906	BB	0.49	97.2725	3092.2192	49.3641
			Totals:	6264.1016	100.0000

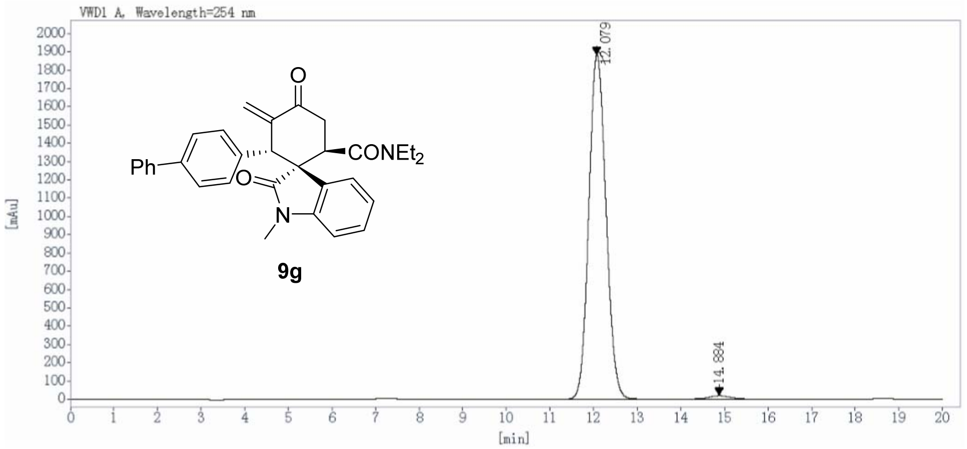

Ret Time [min]	Peak Type	Width [min]	Height [mAU]	Area [mAU*s]	Area [%]
12.079	BB	0.43	1883.7683	51446.0313	98.8894
14.884	MM	0.53	18.0919	577.7576	1.1106
			Totals:	52023.7888	100.0000

HRMS (ESI-TOF) m/z: $[M + Na]^+$ Calcd for $C_{32}H_{32}O_3N_2Na^+$ 515.2305; Found 515.2300.

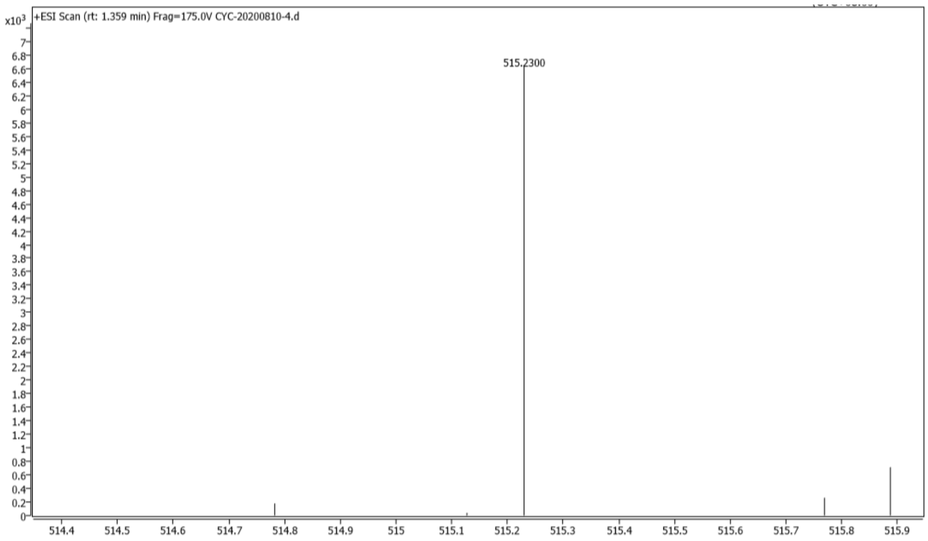

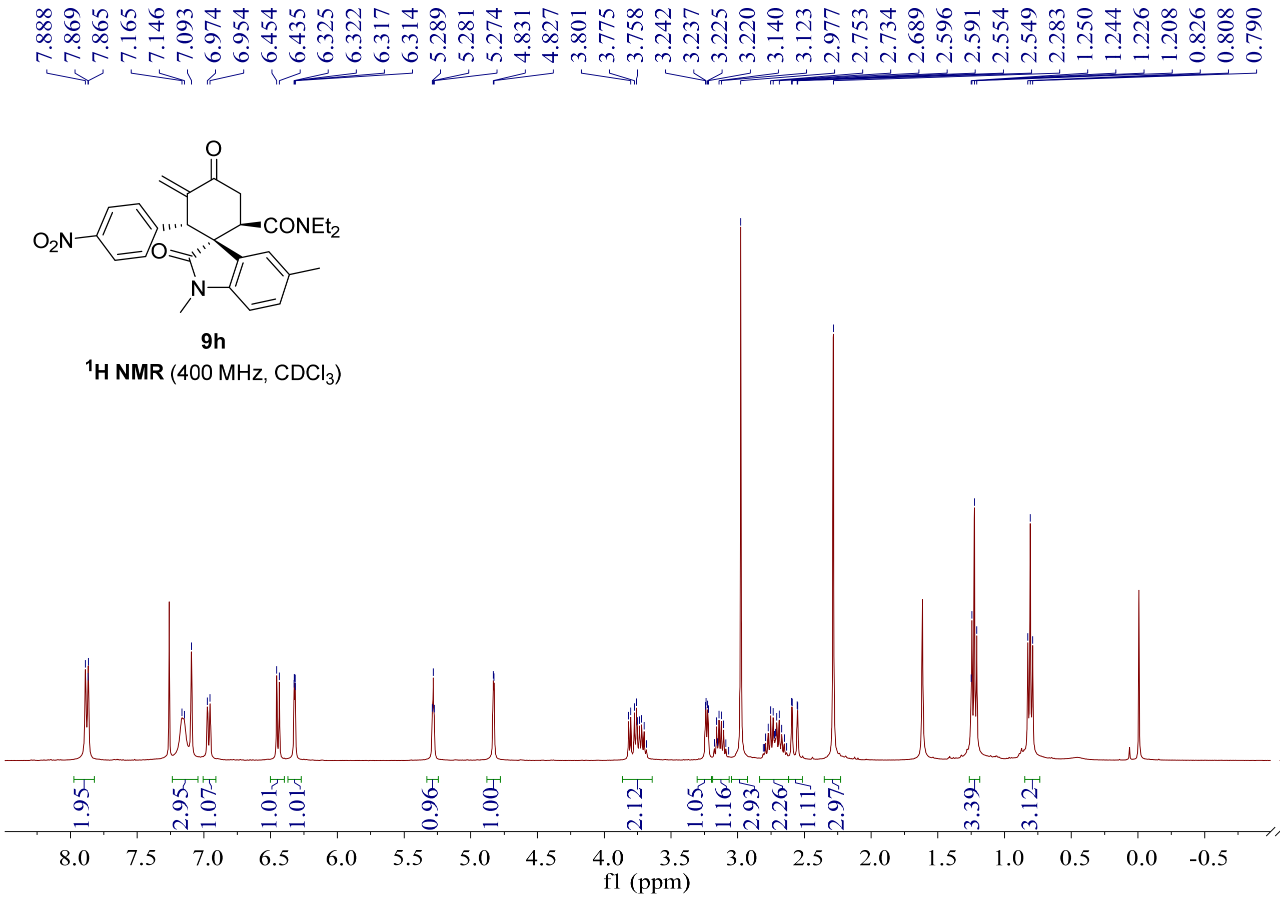
7.888 7.869 7.865 7.165 7.146 7.093 6.974 6.954 6.454 6.435 6.325 6.322 6.317 6.314 5.289 5.281 5.274 4.831 4.827 3.801 3.775 3.758 3.242 3.237 3.225 3.220 3.140 3.123 2.977 2.753 2.734 2.689 2.596 2.591 2.554 2.549 2.283 1.250 1.244 1.226 1.208 0.826 0.808 0.790
O
O_2N
$CONEt_2$
N
9h
^{1}H NMR (400 MHz, $CDCl_3$)
1.95 2.95 1.07 1.01 1.01 0.96 1.00 2.12 1.05 1.16 2.93 2.26 1.11 2.97 3.39 3.12
8.0 7.5 7.0 6.5 6.0 5.5 5.0 4.5 4.0 3.5 3.0 2.5 2.0 1.5 1.0 0.5 0.0 -0.5
f1 (ppm)

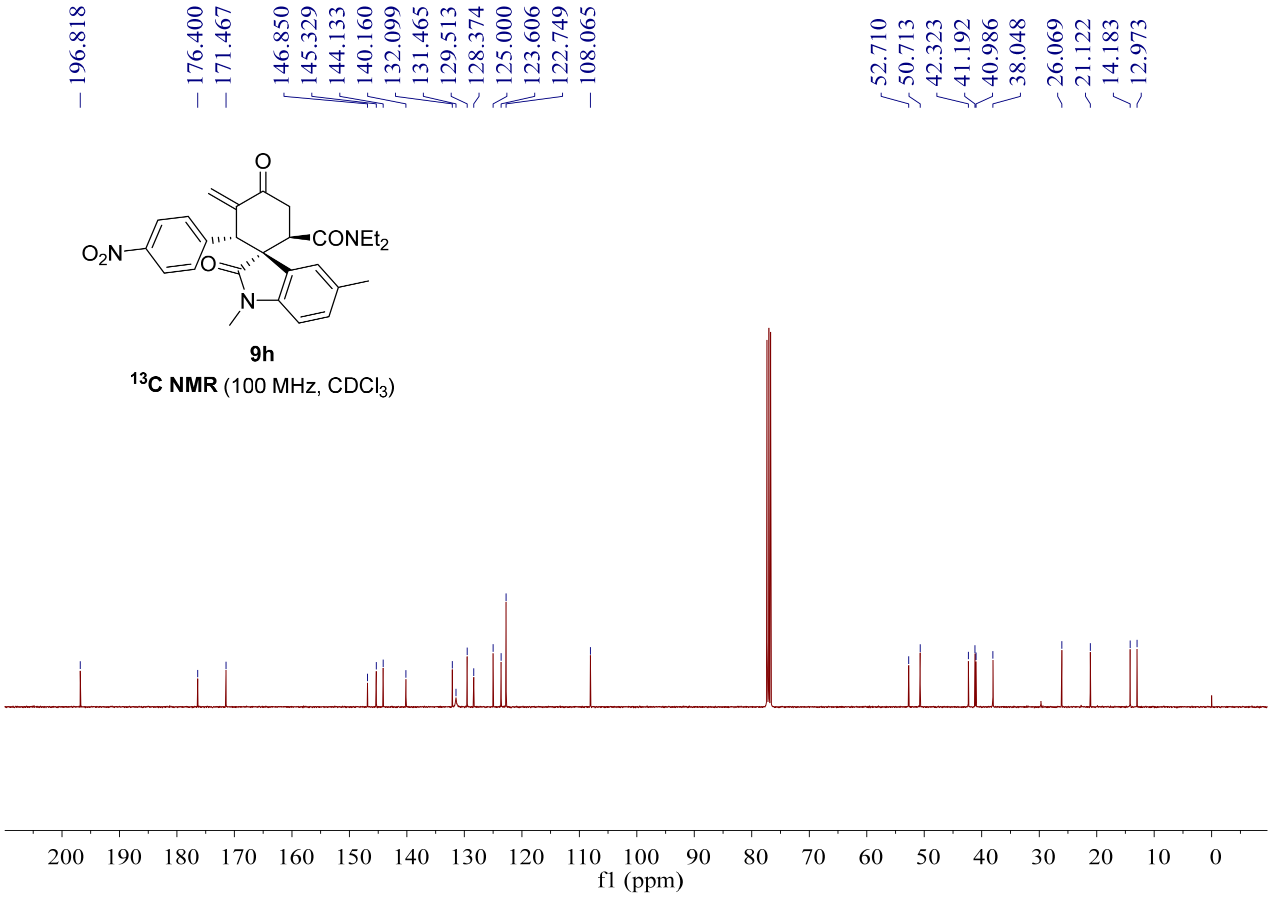
196.818 176.400 171.467 146.850 145.329 144.133 140.160 132.099 131.465 129.513 128.374 125.000 123.606 122.749 108.065 52.710 50.713 42.323 41.192 40.986 38.048 26.069 21.122 14.183 12.973
O
O_2N
$CONEt_2$
N
9h
^{13}C NMR (100 MHz, $CDCl_3$)
200 190 180 170 160 150 140 130 120 110 100 90 80 70 60 50 40 30 20 10 0
f1 (ppm)

Daicel Chiral IE Column, *i*PrOH/*n*-hexane = 40/60, 1.0 mL/min

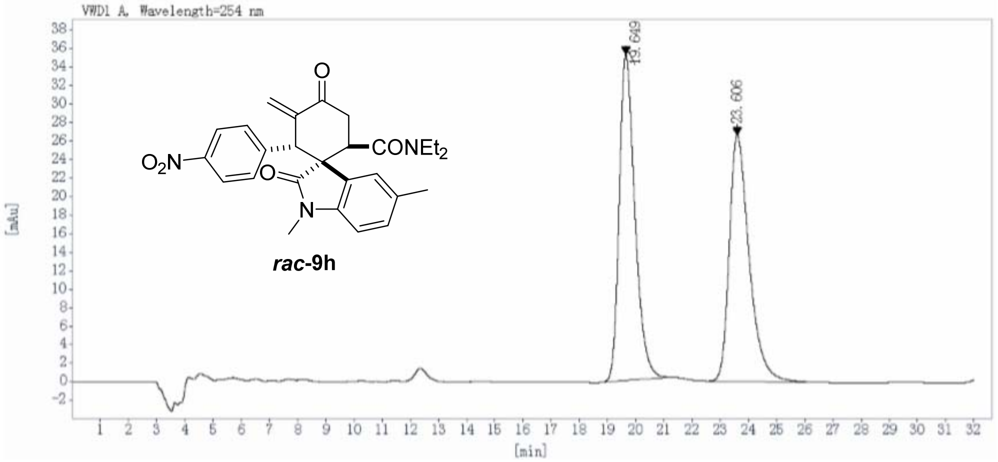

Ret Time [min]	Peak Type	Width [min]	Height [mAU]	Area [mAU*s]	Area [%]
19.649	BB	0.59	35.1576	1354.2555	50.9757
23.606	BB	0.74	26.5599	1302.4154	49.0243
			Totals:	2656.6709	100.0000

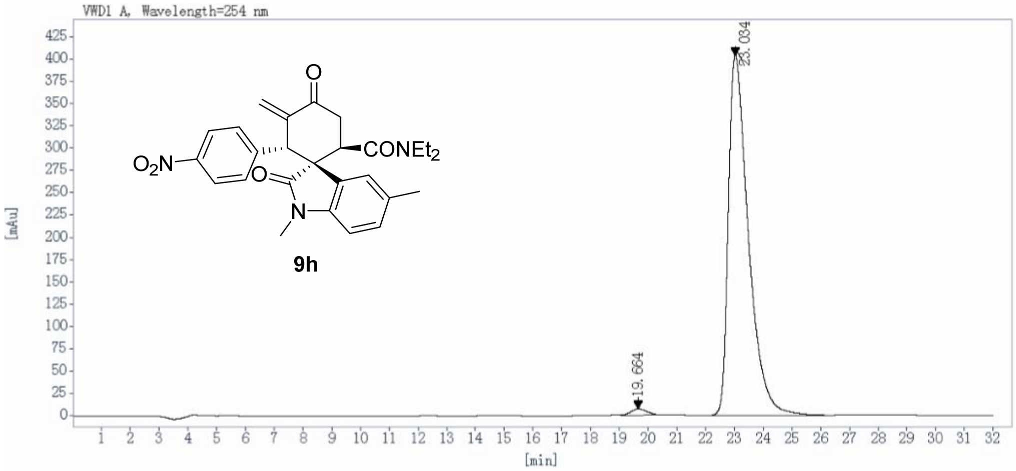

Ret Time [min]	Peak Type	Width [min]	Height [mAU]	Area [mAU*s]	Area [%]
19.664	FM	0.66	7.2431	285.7182	1.4422
23.034	BB	0.73	403.4261	19525.7051	98.5578
			Totals:	19811.4232	100.0000

HRMS (ESI-TOF) m/z: [M + Na]$^+$ Calcd for $C_{27}H_{29}O_5N_3Na^+$ 498.1999; Found 498.1999.

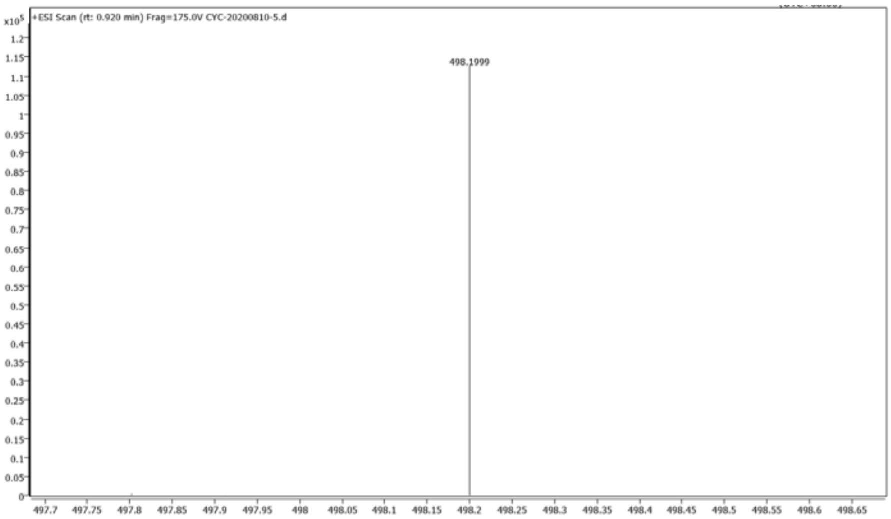

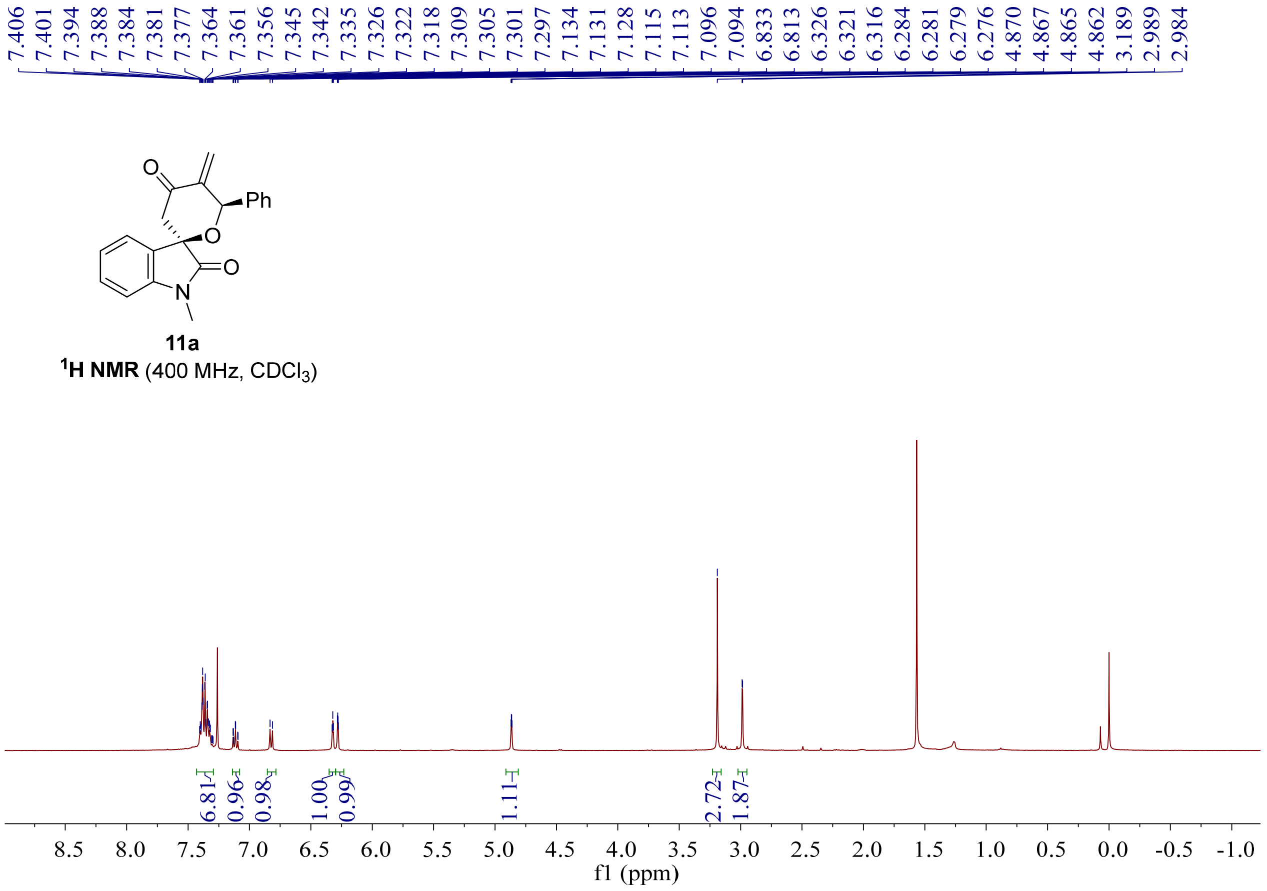
7.406 7.401 7.394 7.388 7.384 7.381 7.377 7.364 7.361 7.356 7.345 7.342 7.335 7.326 7.322 7.318 7.309 7.305 7.301 7.297 7.134 7.131 7.128 7.115 7.113 7.096 7.094 6.833 6.813 6.326 6.321 6.316 6.284 6.281 6.279 6.276 4.870 4.867 4.865 4.862 3.189 2.989 2.984
O
Ph
O
N
O
11a
^{1}H NMR (400 MHz, $CDCl_3$)
6.81 0.96 0.98 1.00 0.99 1.11 2.72 1.87
8.5 8.0 7.5 7.0 6.5 6.0 5.5 5.0 4.5 4.0 3.5 3.0 2.5 2.0 1.5 1.0 0.5 0.0 -0.5 -1.0
f1 (ppm)

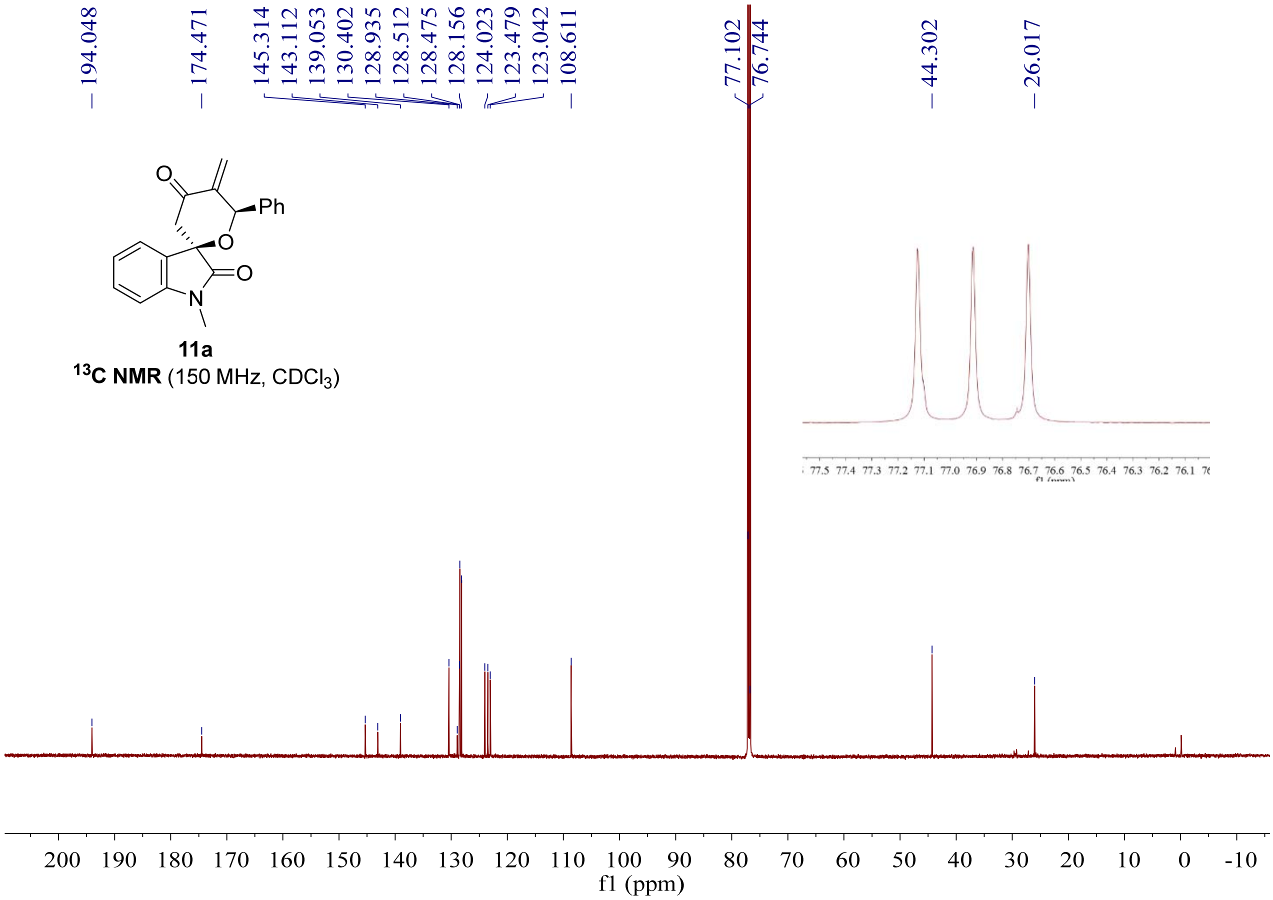
194.048
174.471
145.314 143.112 139.053 130.402 128.935 128.512 128.475 128.156 124.023 123.479 123.042 108.611
77.102 76.744
44.302
26.017
O
Ph
O
N
O
11a
^{13}C NMR (150 MHz, $CDCl_3$)
77.5 77.4 77.3 77.2 77.1 77.0 76.9 76.8 76.7 76.6 76.5 76.4 76.3 76.2 76.1
200 190 180 170 160 150 140 130 120 110 100 90 80 70 60 50 40 30 20 10 0 -10
f1 (ppm)

Daicel Chiral IB Column, (*i*PrOH/*n*-hexane = 10/90, 1.0 mL/min)

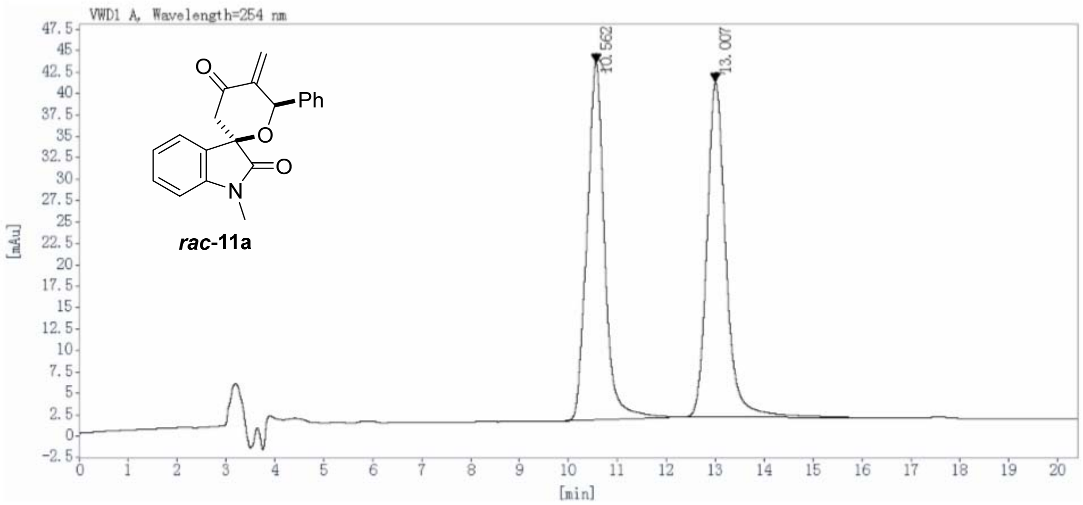

Ret Time [min]	Peak Type	Width [min]	Height [mAU]	Area [mAU*s]	Area [%]
10.562	BB	0.37	41.7363	1043.8326	49.5263
13.007	BB	0.41	39.0947	1063.8005	50.4737
			Totals:	2107.6332	100.0000

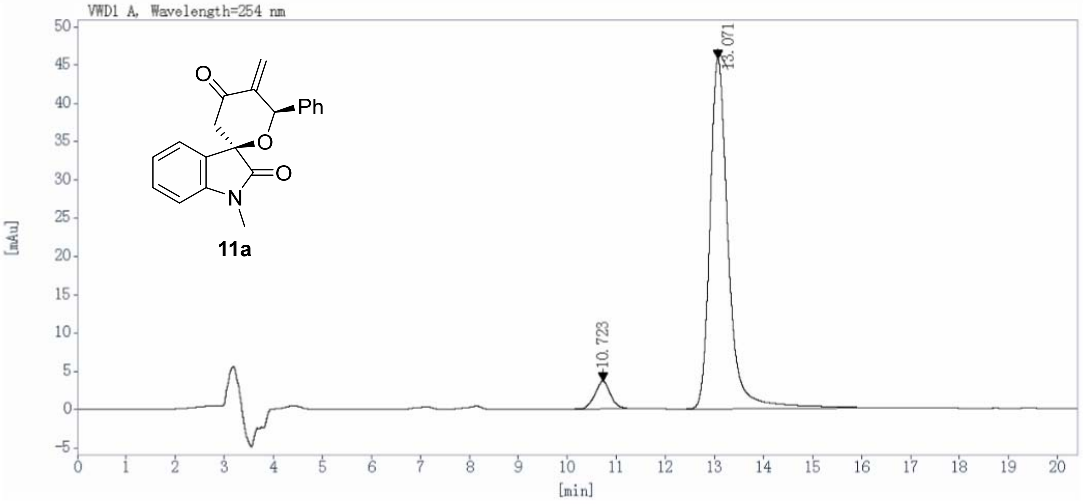

Ret Time [min]	Peak Type	Width [min]	Height [mAU]	Area [mAU*s]	Area [%]
10.723	BB	0.34	3.6328	81.8187	6.3976
13.071	BB	0.40	45.7734	1197.0728	93.6024
			Totals:	1278.8915	100.0000

HRMS (ESI-TOF) m/z: $[M + H]^+$ Calcd for $C_{20}H_{18}O_3N^+$ 320.1281; Found 320.1276.

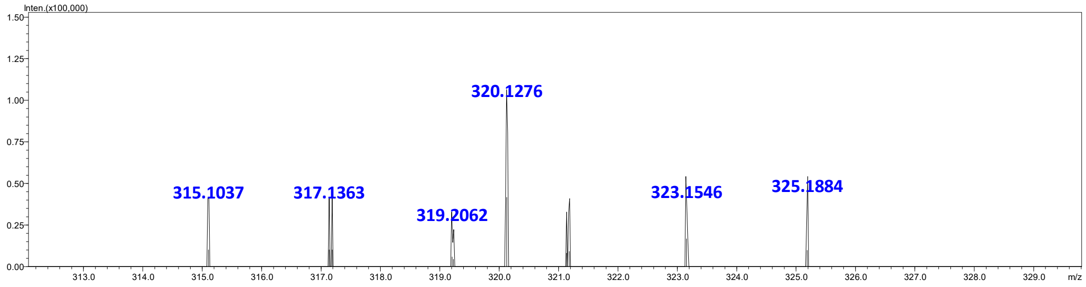

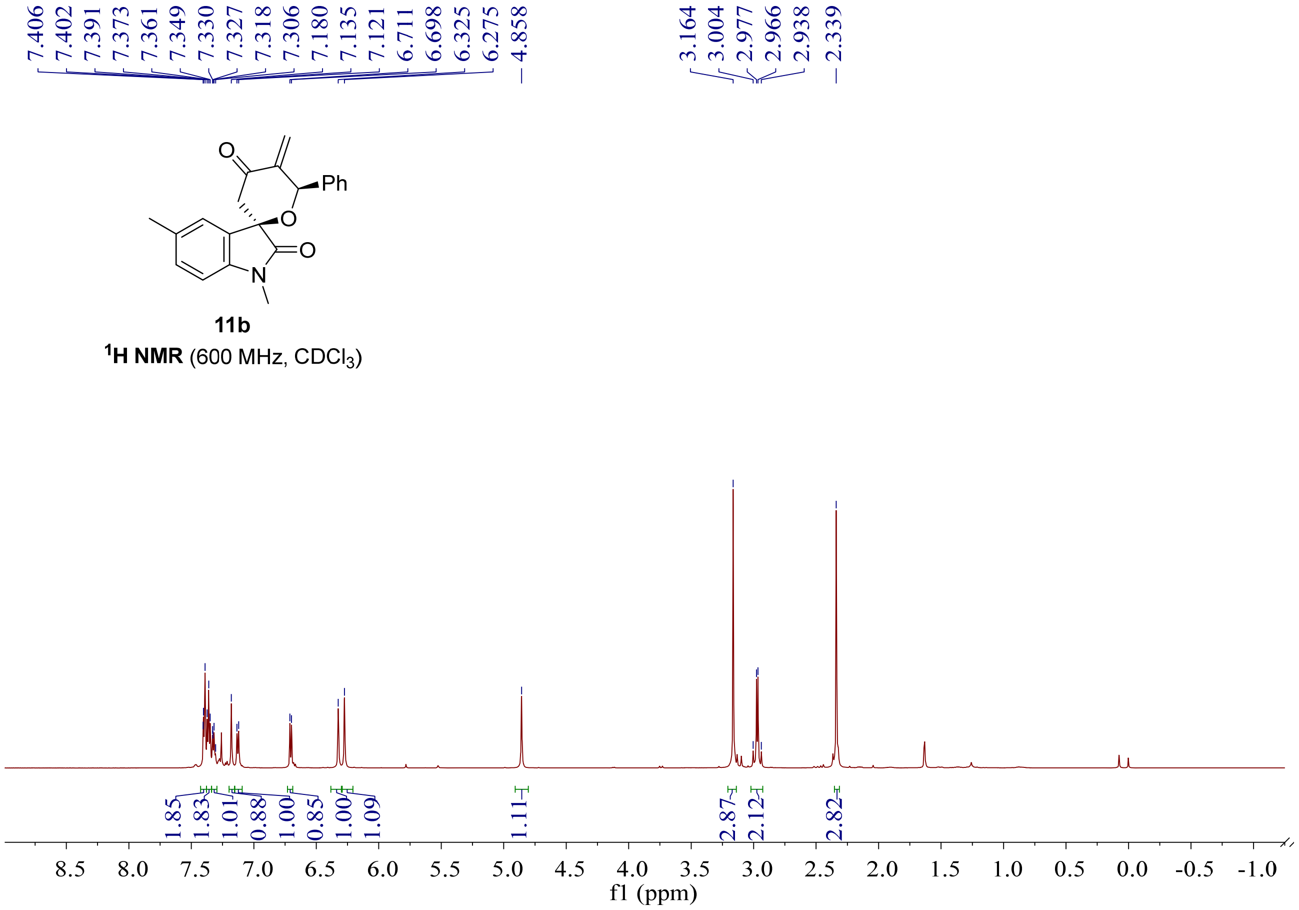

11b

^{1}H NMR (600 MHz, $CDCl_3$)

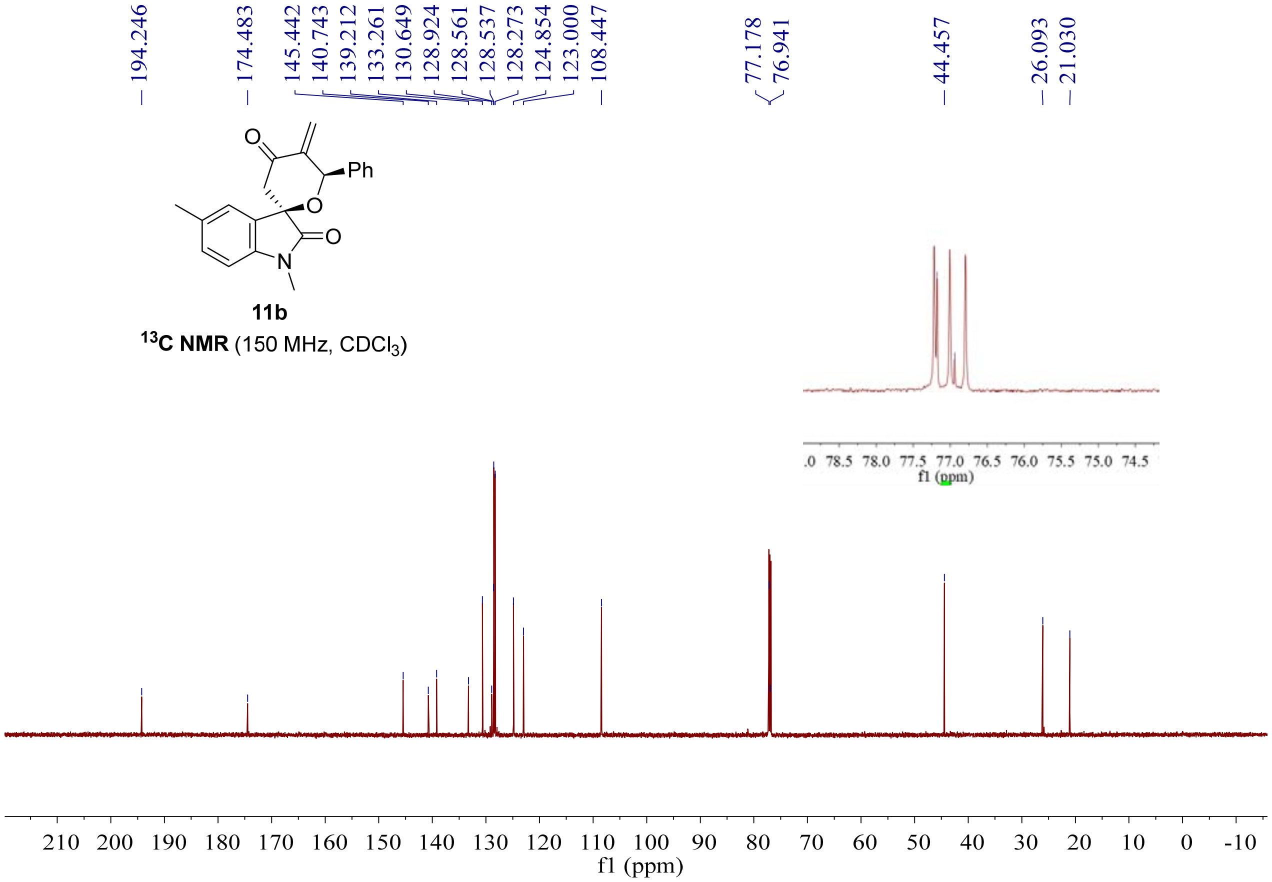

11b

^{13}C NMR (150 MHz, $CDCl_3$)

Daicel Chiral IE Column, (iPrOH/n-hexane = 40/60, 1.0 mL/min)

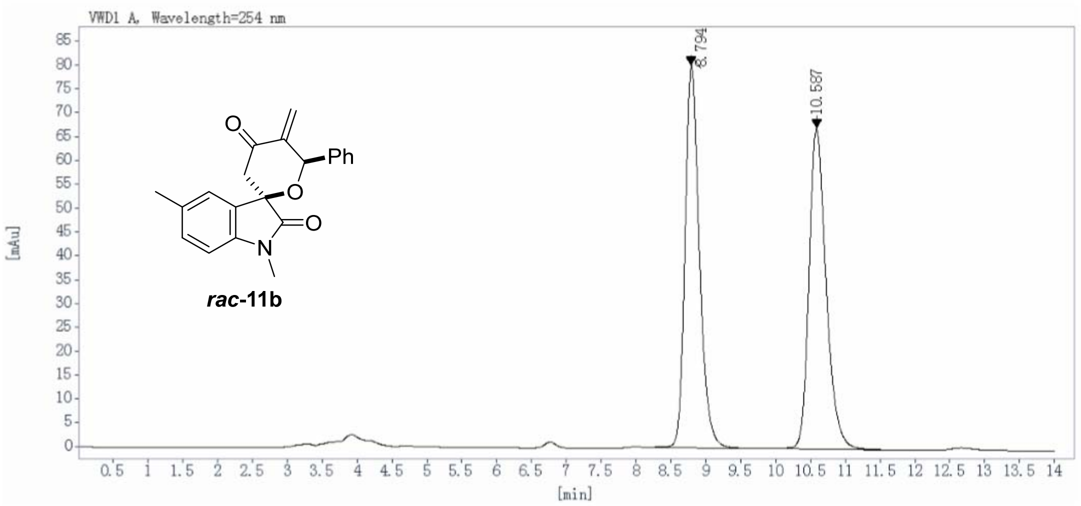

Ret Time [min]	Peak Type	Width [min]	Height [mAU]	Area [mAU*s]	Area [%]
8.794	BB	0.22	80.2525	1146.9333	49.9513
10.587	BB	0.26	67.2146	1149.1676	50.0487
			Totals:	2296.1010	100.0000

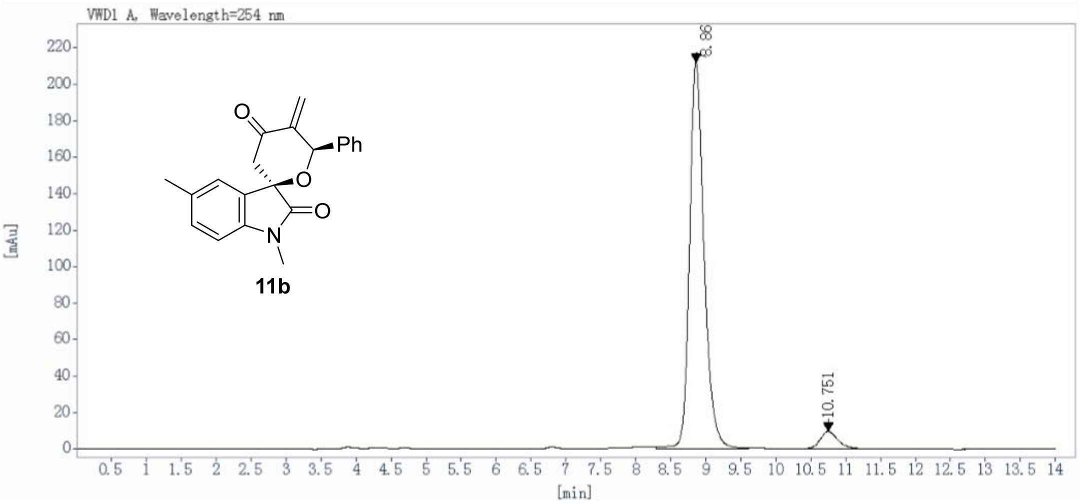

Ret Time [min]	Peak Type	Width [min]	Height [mAU]	Area [mAU*s]	Area [%]
8.860	MM	0.24	212.2242	3083.4087	95.0262
10.751	MM	0.28	9.4420	161.3902	4.9738
			Totals:	3244.7989	100.0000

HRMS (ESI-TOF) m/z: $[M + H]^+$ Calcd for $C_{21}H_{20}O_3N^+$ 334.1438; Found 334.1442; $[M + Na]^+$ Calcd. for $C_{21}H_{19}O_3NNa^+$ 356.1257; Found 356.1243.

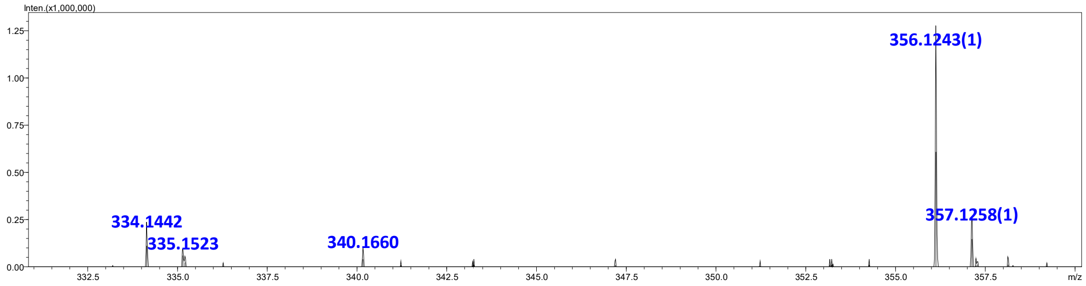

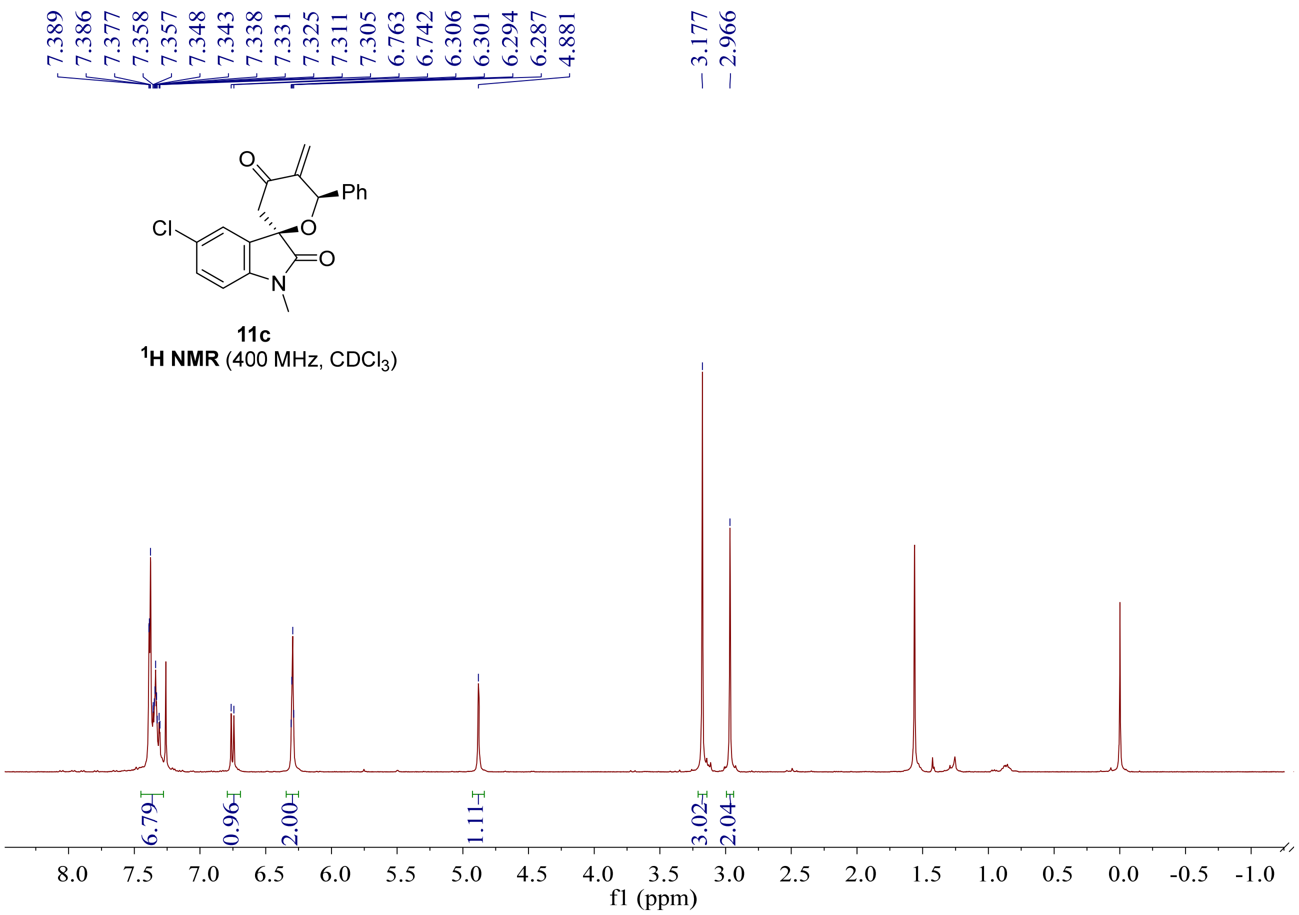
7.389 7.386 7.377 7.358 7.357 7.348 7.343 7.338 7.331 7.325 7.311 7.305 6.763 6.742 6.306 6.301 6.294 6.287 4.881
3.177
2.966
O
Ph
O
Cl
O
N
11c
¹H NMR (400 MHz, CDCl₃)
6.79
0.96
2.00
1.11
3.02
2.04
8.0 7.5 7.0 6.5 6.0 5.5 5.0 4.5 4.0 3.5 3.0 2.5 2.0 1.5 1.0 0.5 0.0 -0.5 -1.0
f1 (ppm)

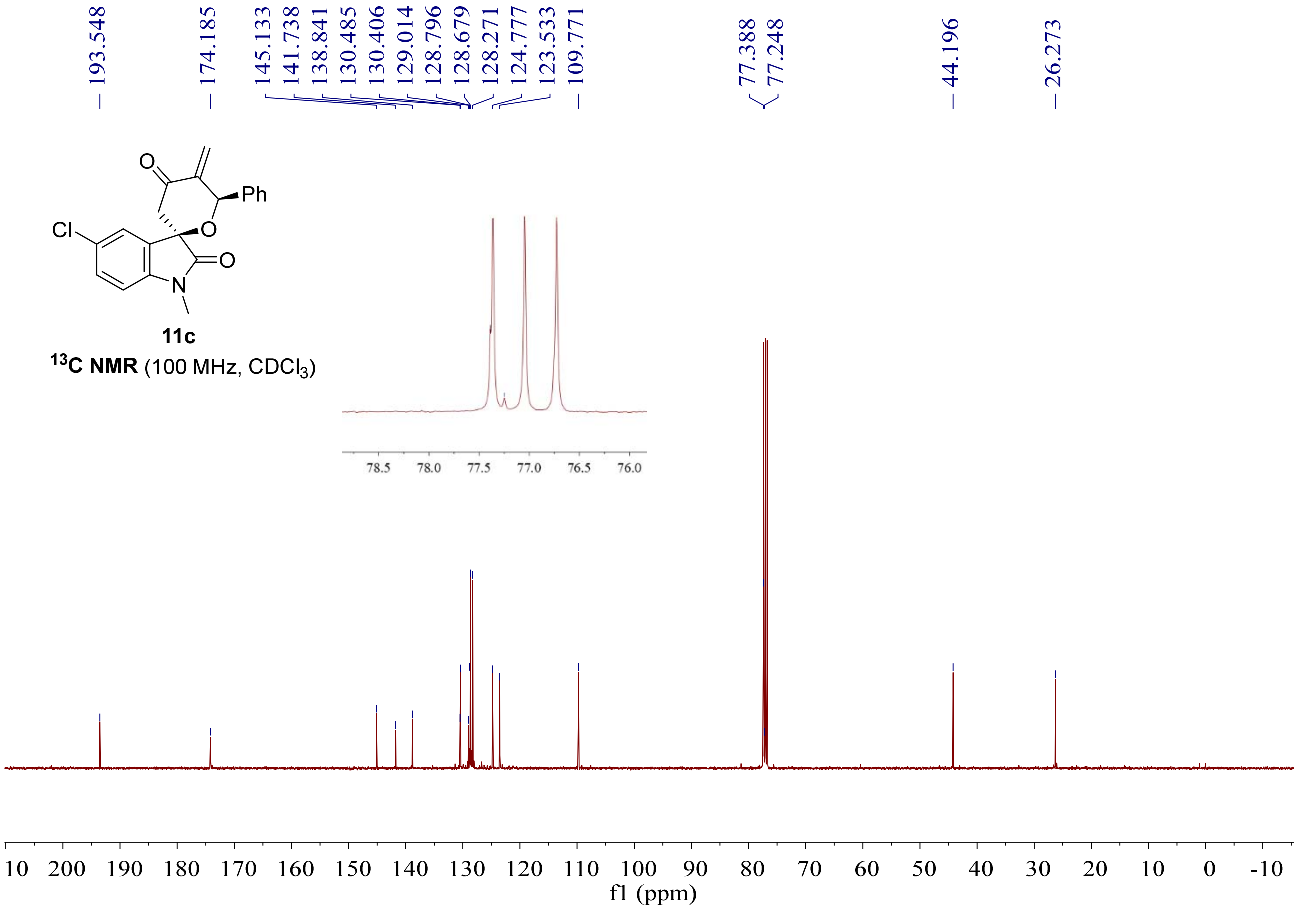
193.548
174.185
145.133 141.738 138.841 130.485 130.406 129.014 128.796 128.679 128.271 124.777 123.533 109.771
77.388 77.248
44.196
26.273
O
Ph
O
Cl
O
N
11c
¹³C NMR (100 MHz, CDCl₃)
78.5 78.0 77.5 77.0 76.5 76.0
10 200 190 180 170 160 150 140 130 120 110 100 90 80 70 60 50 40 30 20 10 0 -10
f1 (ppm)

Daicel Chiral ID Column, (*i*PrOH/*n*-hexane = 20/80, 1.0 mL/min)

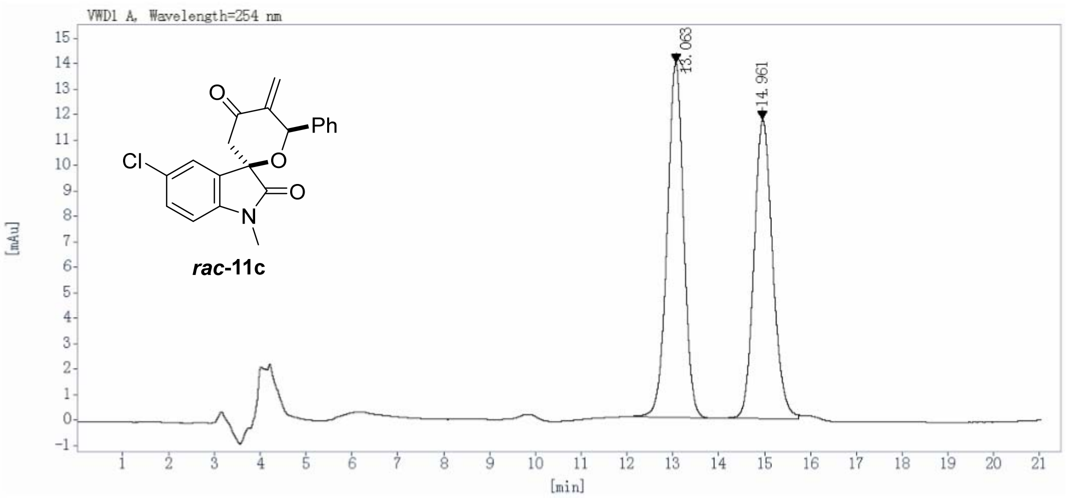

Ret Time [min]	Peak Type	Width [min]	Height [mAU]	Area [mAU*s]	Area [%]
13.063	BB	0.38	13.9365	347.6921	51.0265
14.961	MM	0.47	11.7429	333.7032	48.9735
			Totals:	681.3953	100.0000

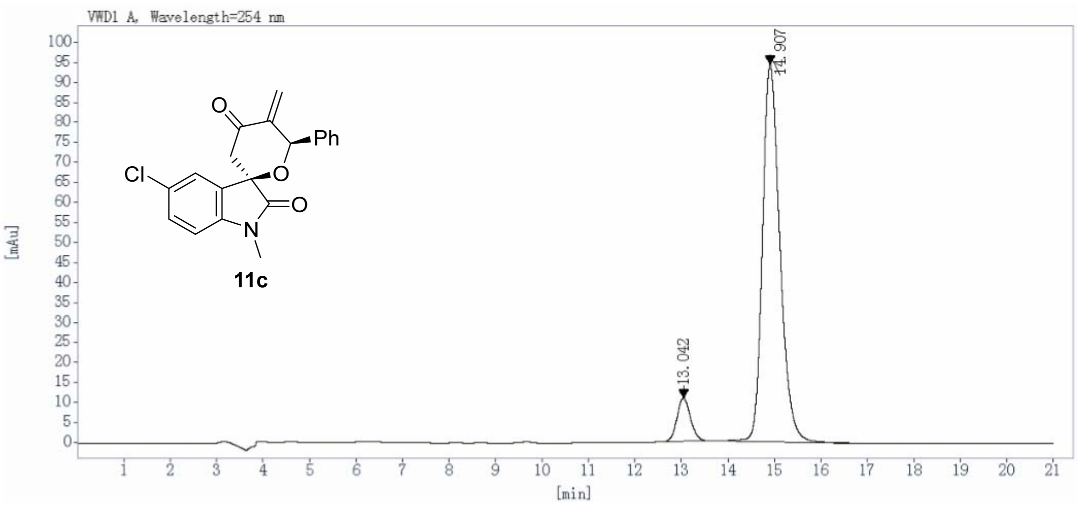

Ret Time [min]	Peak Type	Width [min]	Height [mAU]	Area [mAU*s]	Area [%]
13.042	MM	0.33	10.8674	217.7142	8.1315
14.907	BB	0.40	94.4187	2459.6973	91.8685
			Totals:	2677.4115	100.0000

HRMS (ESI-TOF) m/z: $[M + Na]^+$ Calcd for $C_{20}H_{16}O_3NClNa^+$ 376.0711 (^{35}Cl) and 378.0681 (^{37}Cl); Found 376.0708 (^{35}Cl) and 378.0655 (^{37}Cl).

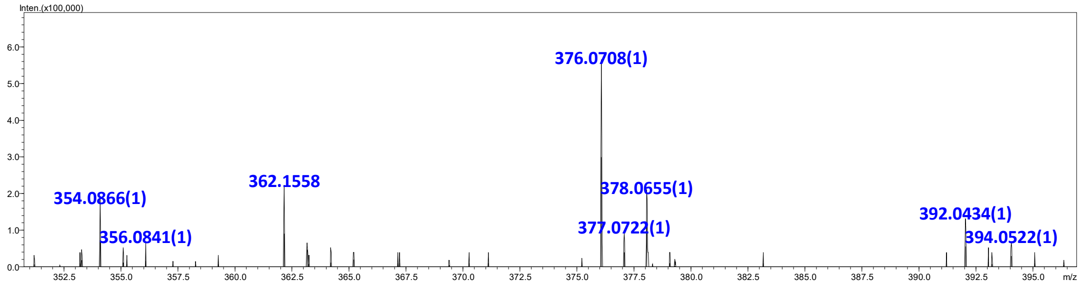

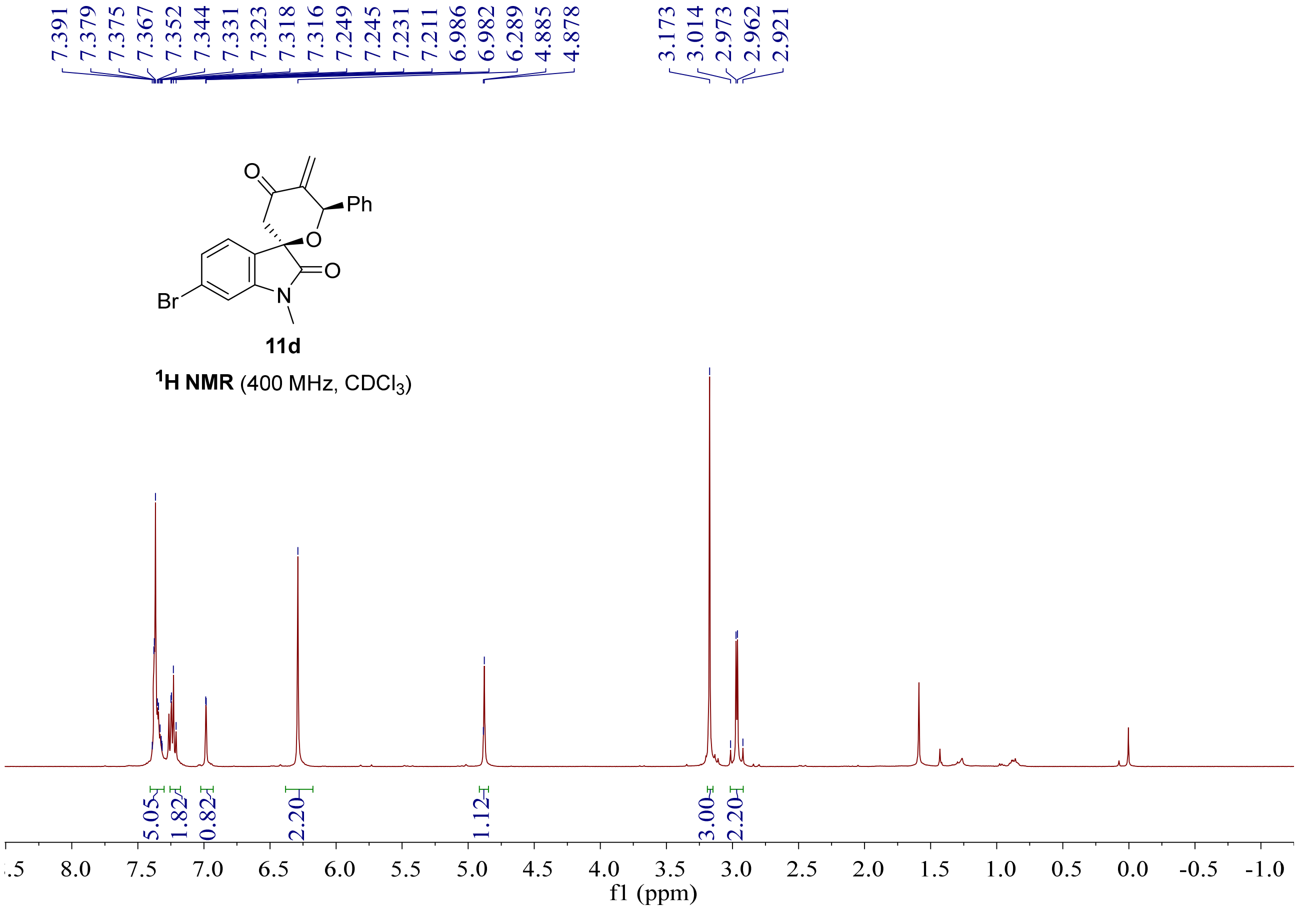
7.391 7.379 7.375 7.367 7.352 7.344 7.331 7.323 7.318 7.316 7.249 7.245 7.231 7.211 6.986 6.982 6.289 4.885 4.878
3.173 3.014 2.973 2.962 2.921
O
Ph
O
O
Br
N
11d
1H NMR (400 MHz, CDCl3)
5.05
1.82
0.82
2.20
1.12
3.00
2.20
.5 8.0 7.5 7.0 6.5 6.0 5.5 5.0 4.5 4.0 3.5 3.0 2.5 2.0 1.5 1.0 0.5 0.0 -0.5 -1.0
f1 (ppm)

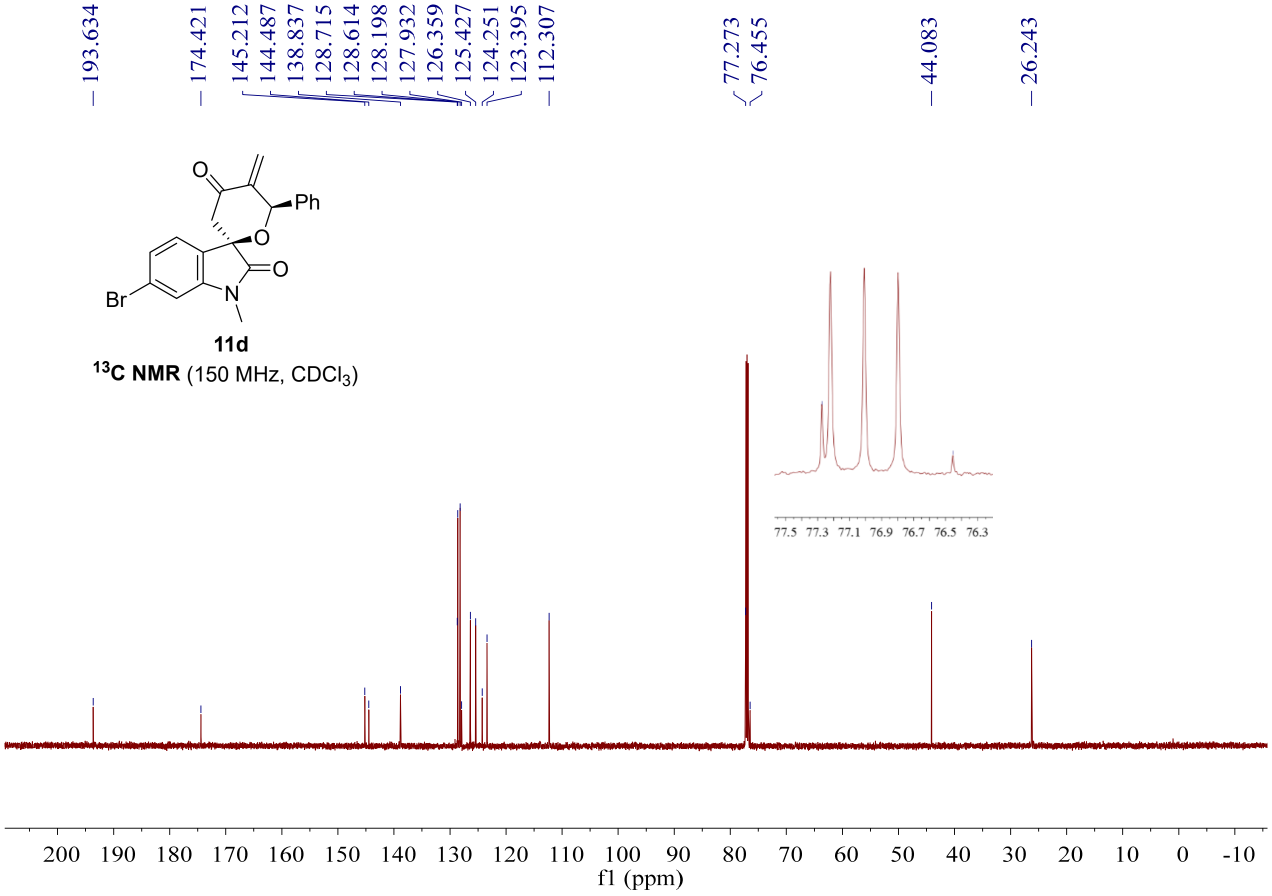
193.634
174.421
145.212 144.487 138.837 128.715 128.614 128.198 127.932 126.359 125.427 124.251 123.395 112.307
77.273 76.455
44.083
26.243
O
Ph
O
O
Br
N
11d
13C NMR (150 MHz, CDCl3)
77.5 77.3 77.1 76.9 76.7 76.5 76.3
200 190 180 170 160 150 140 130 120 110 100 90 80 70 60 50 40 30 20 10 0 -10
f1 (ppm)

Daicel Chiral IF Column, *i*PrOH/*n*-hexane = 40/60, 1.0 mL/min.

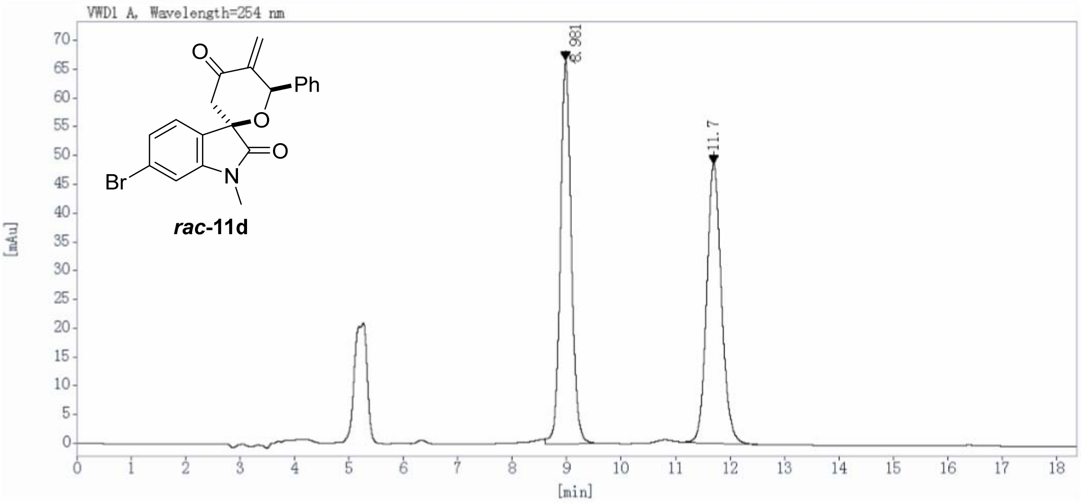

Ret Time [min]	Peak Type	Width [min]	Height [mAU]	Area [mAU*s]	Area [%]
8.981	FM	0.23	66.6191	927.3884	50.3936
11.700	BB	0.29	48.4221	912.9031	49.6064
			Totals:	1840.2916	100.0000

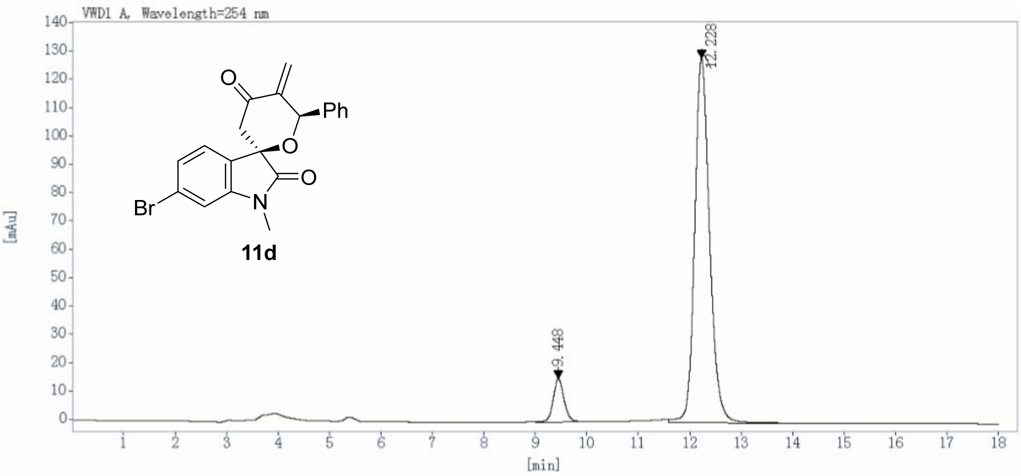

Ret Time [min]	Peak Type	Width [min]	Height [mAU]	Area [mAU*s]	Area [%]
9.448	FM	0.24	15.2788	222.8707	7.9543
12.228	FM	0.33	128.5995	2579.0198	92.0457
			Totals:	2801.8905	100.0000

HRMS (ESI-TOF) m/z: $[M + Na]^+$ Calcd for $C_{20}H_{16}O_3NBrNa^+$ 398.0386 (^{79}Br) and 400.0366 (^{81}Br), Found 398.0385 (^{79}Br) and 400.0350 (^{81}Br).

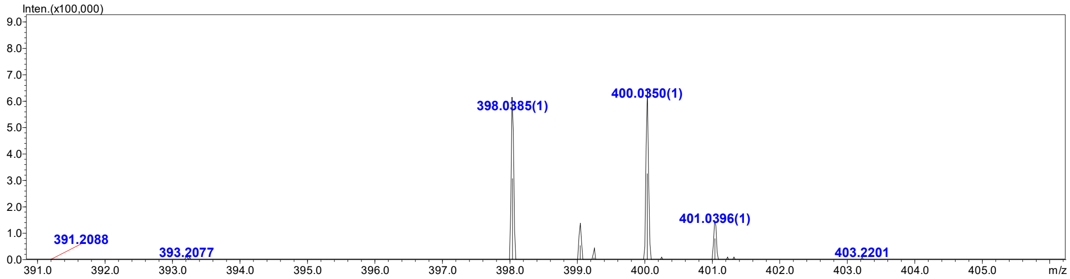

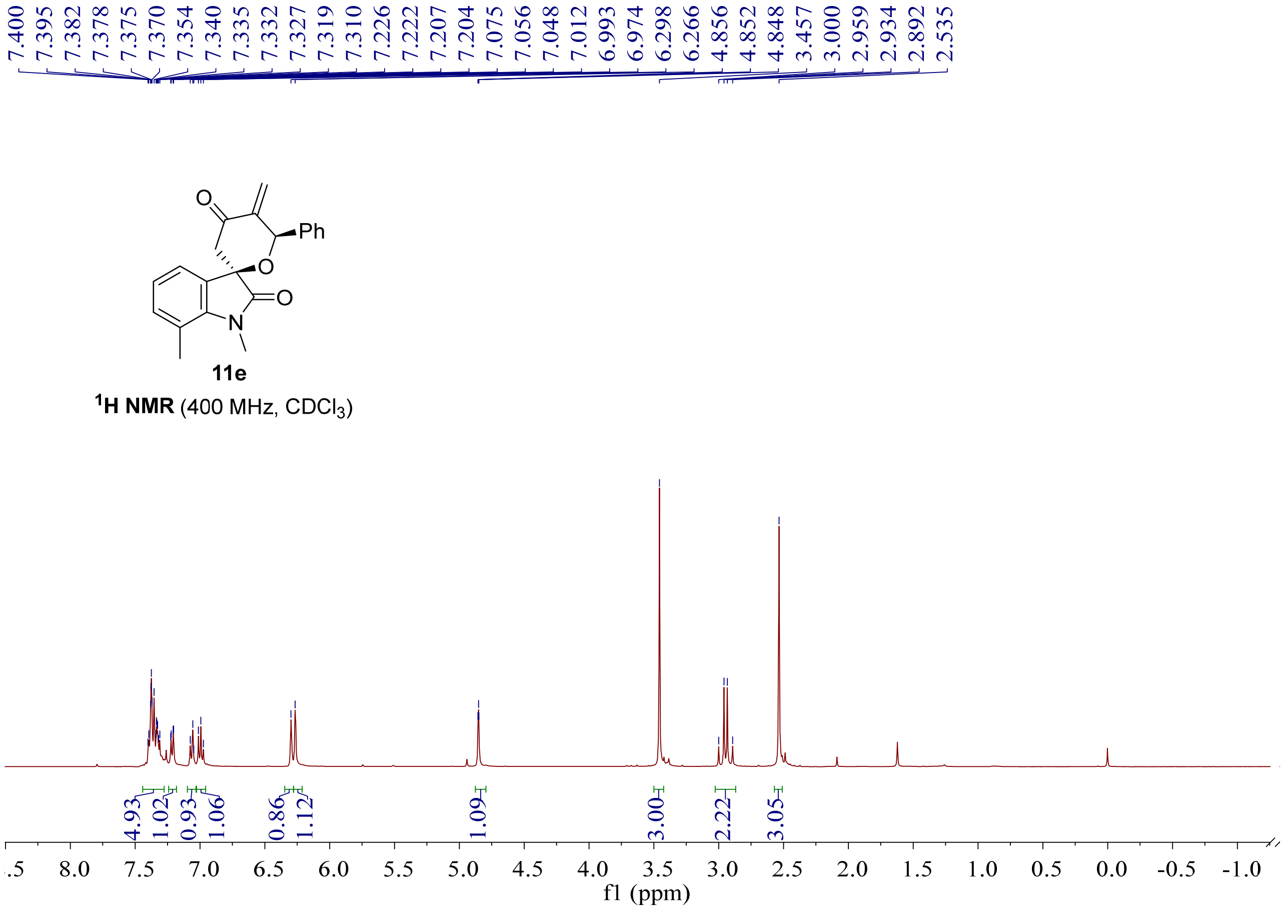
7.400 7.395 7.382 7.378 7.375 7.370 7.354 7.340 7.335 7.332 7.327 7.319 7.310 7.226 7.222 7.207 7.204 7.075 7.056 7.048 7.012 6.993 6.974 6.298 6.266 4.856 4.852 4.848 3.457 3.000 2.959 2.934 2.892 2.535
O
Ph
O
O
N
11e
1H NMR (400 MHz, CDCl3)
4.93 1.02 0.93 1.06 0.86 1.12 1.09 3.00 2.22 3.05
.5 8.0 7.5 7.0 6.5 6.0 5.5 5.0 4.5 4.0 3.5 3.0 2.5 2.0 1.5 1.0 0.5 0.0 -0.5 -1.0
f1 (ppm)

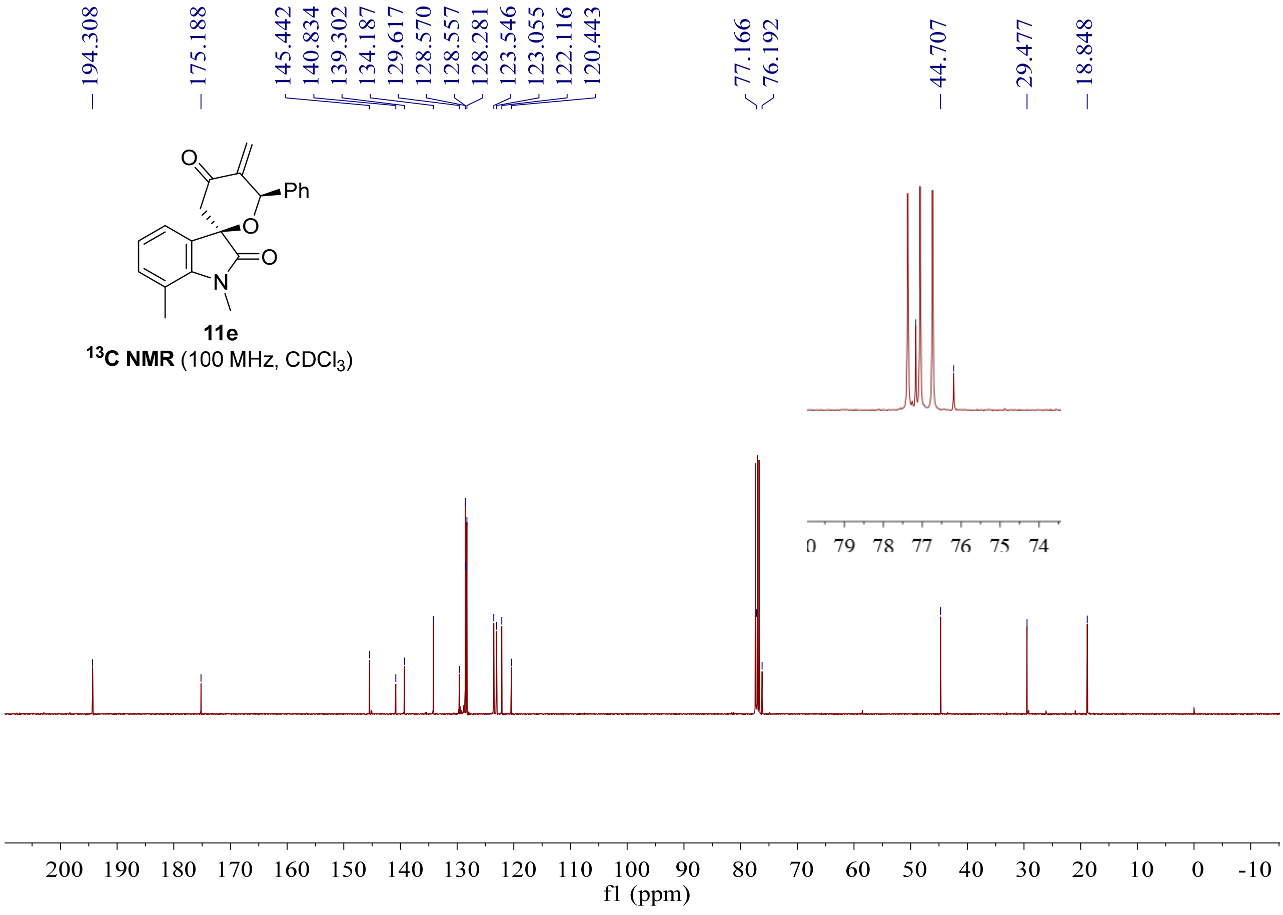
194.308 175.188 145.442 140.834 139.302 134.187 129.617 128.570 128.557 128.281 123.546 123.055 122.116 120.443 77.166 76.192 44.707 29.477 18.848
O
Ph
O
O
N
11e
13C NMR (100 MHz, CDCl3)
0 79 78 77 76 75 74
200 190 180 170 160 150 140 130 120 110 100 90 80 70 60 50 40 30 20 10 0 -10
f1 (ppm)

Daicel Chiral IF Column, *i*PrOH/*n*-hexane = 40/60, 1.0 mL/min.

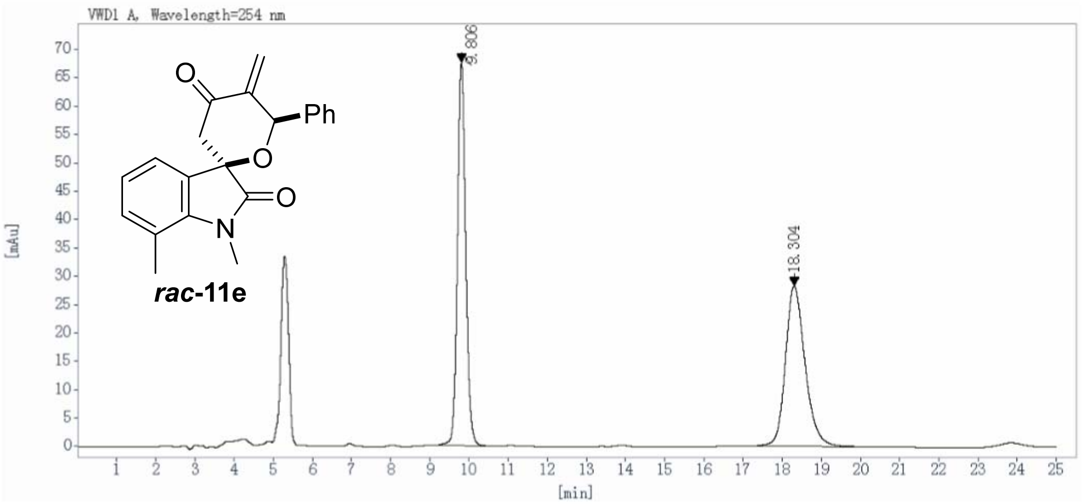

Ret Time [min]	Peak Type	Width [min]	Height [mAU]	Area [mAU*s]	Area [%]
9.806	BB	0.22	67.6106	994.0990	49.8888
18.304	BB	0.54	28.3010	998.5316	50.1112
			Totals:	1992.6306	100.0000

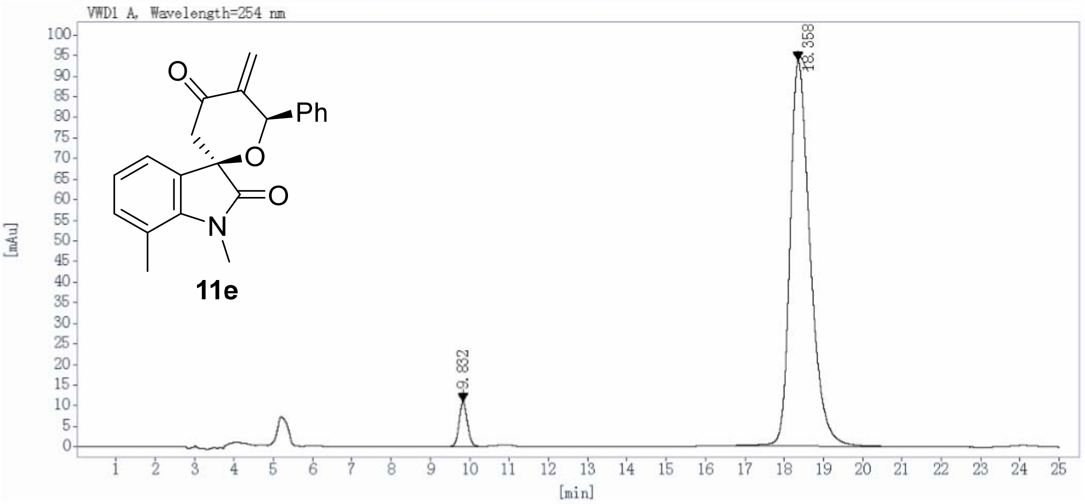

Ret Time [min]	Peak Type	Width [min]	Height [mAU]	Area [mAU*s]	Area [%]
9.832	BB	0.23	10.8507	159.3224	4.5663
18.358	BB	0.54	93.5735	3329.7952	95.4337
			Totals:	3489.1176	100.0000

HRMS (ESI-TOF) m/z: $[M + Na]^+$ Calcd for $C_{21}H_{19}O_3NNa^+$ 356.1257; Found 356.1248.

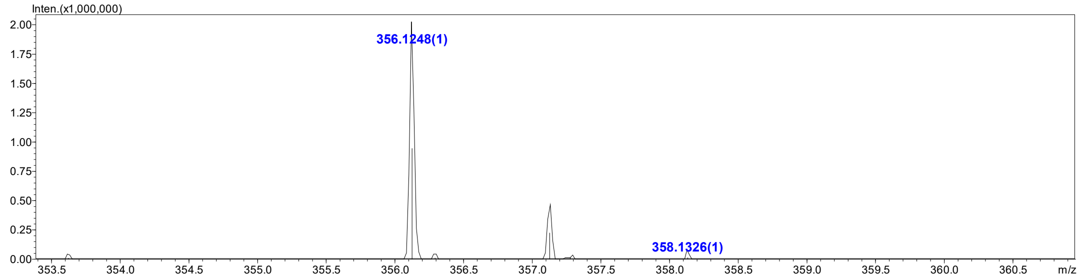

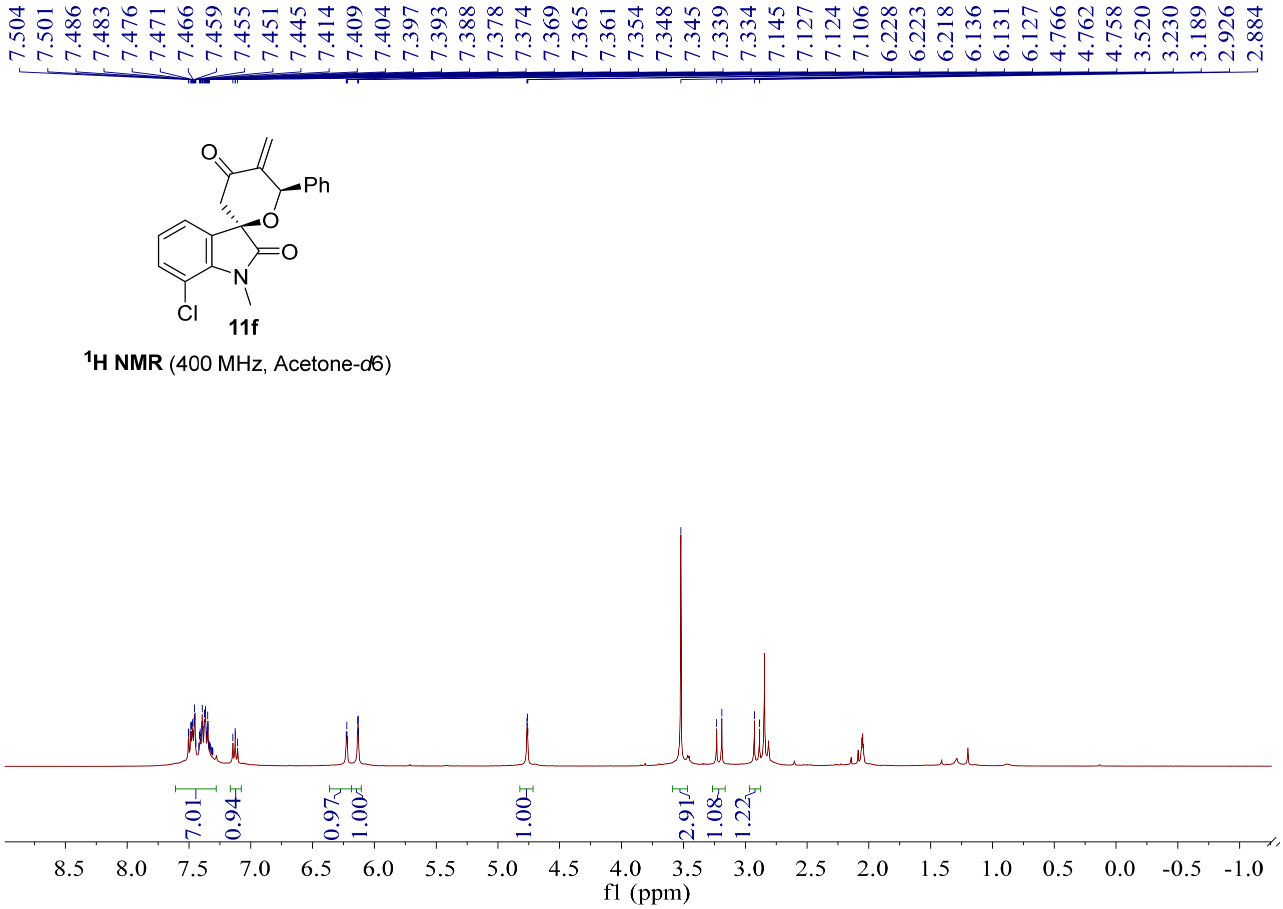
7.504 7.501 7.486 7.483 7.476 7.471 7.466 7.459 7.455 7.451 7.445 7.414 7.409 7.404 7.397 7.393 7.388 7.378 7.374 7.369 7.365 7.361 7.354 7.348 7.345 7.339 7.334 7.145 7.127 7.124 7.106 6.228 6.223 6.218 6.136 6.131 6.127 4.766 4.762 4.758 3.520 3.230 3.189 2.926 2.884
O
Ph
O
O
N
Cl
11f
1H NMR (400 MHz, Acetone-d6)
7.01
0.94
0.97
1.00
1.00
2.91
1.08
1.22
8.5 8.0 7.5 7.0 6.5 6.0 5.5 5.0 4.5 4.0 3.5 3.0 2.5 2.0 1.5 1.0 0.5 0.0 -0.5 -1.0
f1 (ppm)

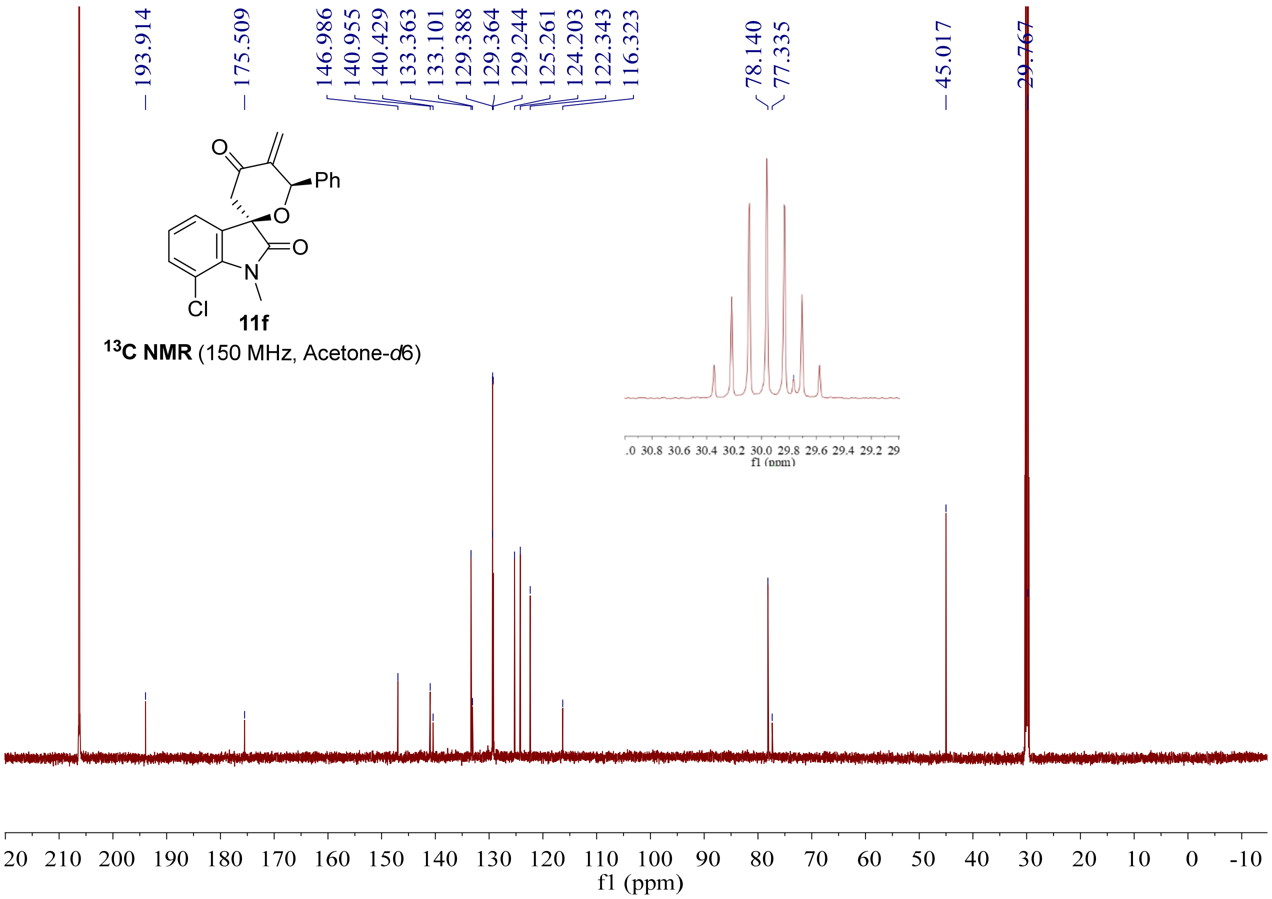
193.914
175.509
146.986 140.955 140.429 133.363 133.101 129.388 129.364 129.244 125.261 124.203 122.343 116.323
78.140 77.335
45.017
29.767
O
Ph
O
O
N
Cl
11f
13C NMR (150 MHz, Acetone-d6)
.0 30.8 30.6 30.4 30.2 30.0 29.8 29.6 29.4 29.2 29
f1 (ppm)
20 210 200 190 180 170 160 150 140 130 120 110 100 90 80 70 60 50 40 30 20 10 0 -10
f1 (ppm)

Daicel Chiral IF Column, *i*PrOH/*n*-hexane = 40/60, 1.0 mL/min

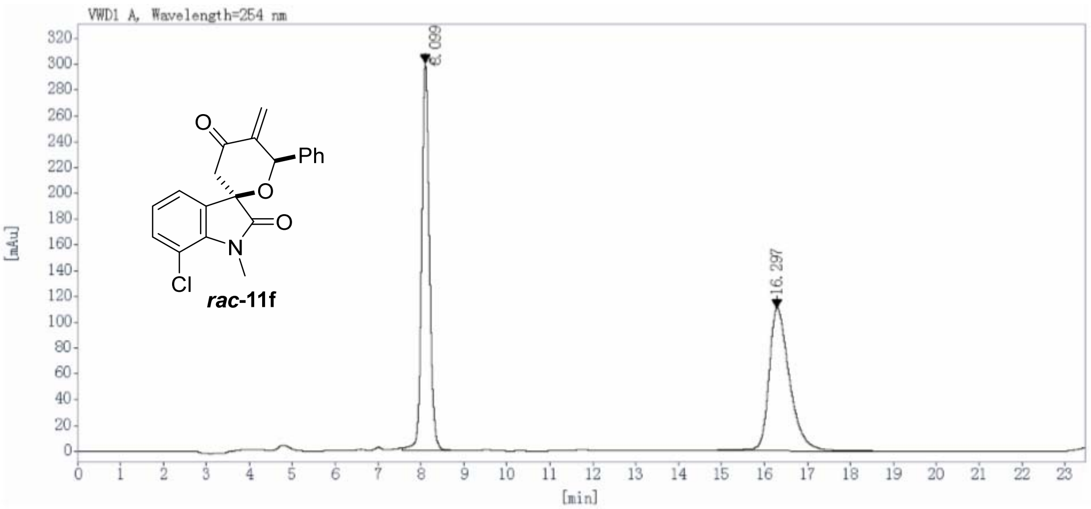

Ret Time [min]	Peak Type	Width [min]	Height [mAU]	Area [mAU*s]	Area [%]
8.099	FM	0.21	300.6503	3780.6301	50.2221
16.297	BB	0.51	110.9613	3747.1873	49.7779
			Totals:	7527.8174	100.0000

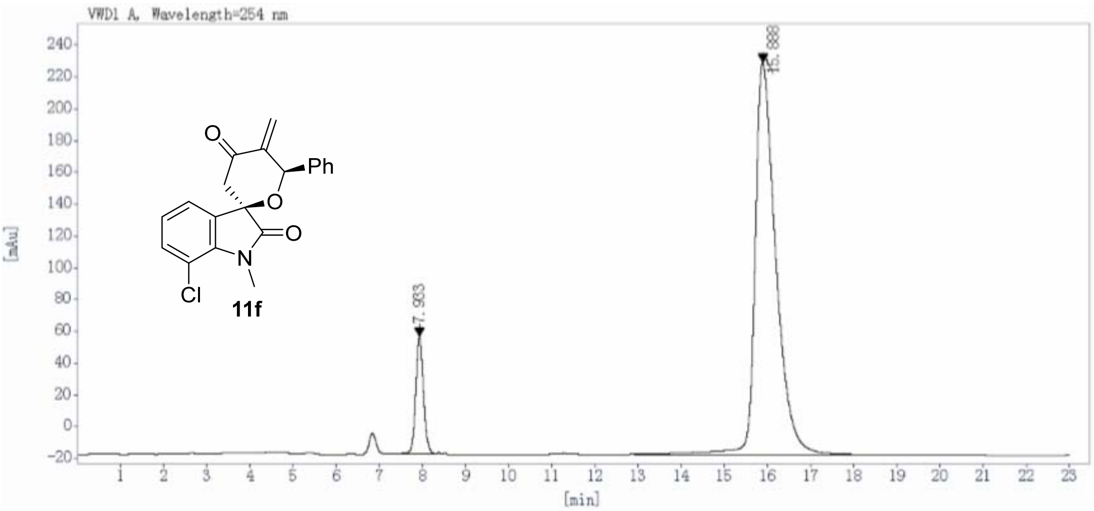

Ret Time [min]	Peak Type	Width [min]	Height [mAU]	Area [mAU*s]	Area [%]
7.933	MM	0.21	73.7646	920.5530	10.0085
15.888	MM	0.56	246.7279	8277.1953	89.9915
			Totals:	9197.7483	100.0000

HRMS (ESI-TOF) m/z: $[M + Na]^+$ Calcd for $C_{20}H_{16}O_3NClNa^+$ 376.0711 (^{35}Cl) and 378.0681 (^{37}Cl); Found 376.0706 (^{35}Cl) and 378.0667 (^{37}Cl).

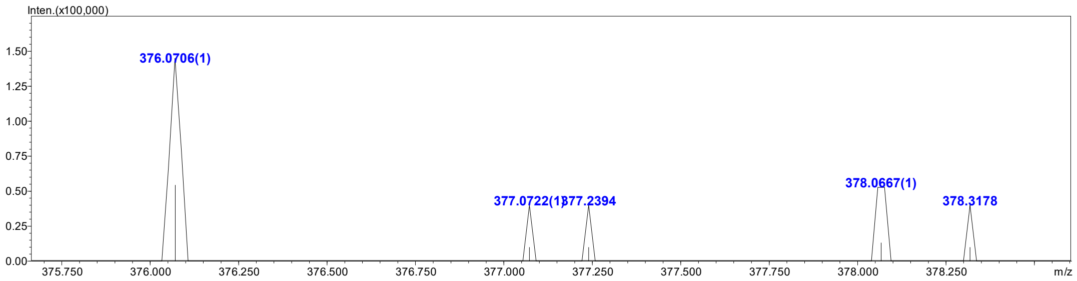

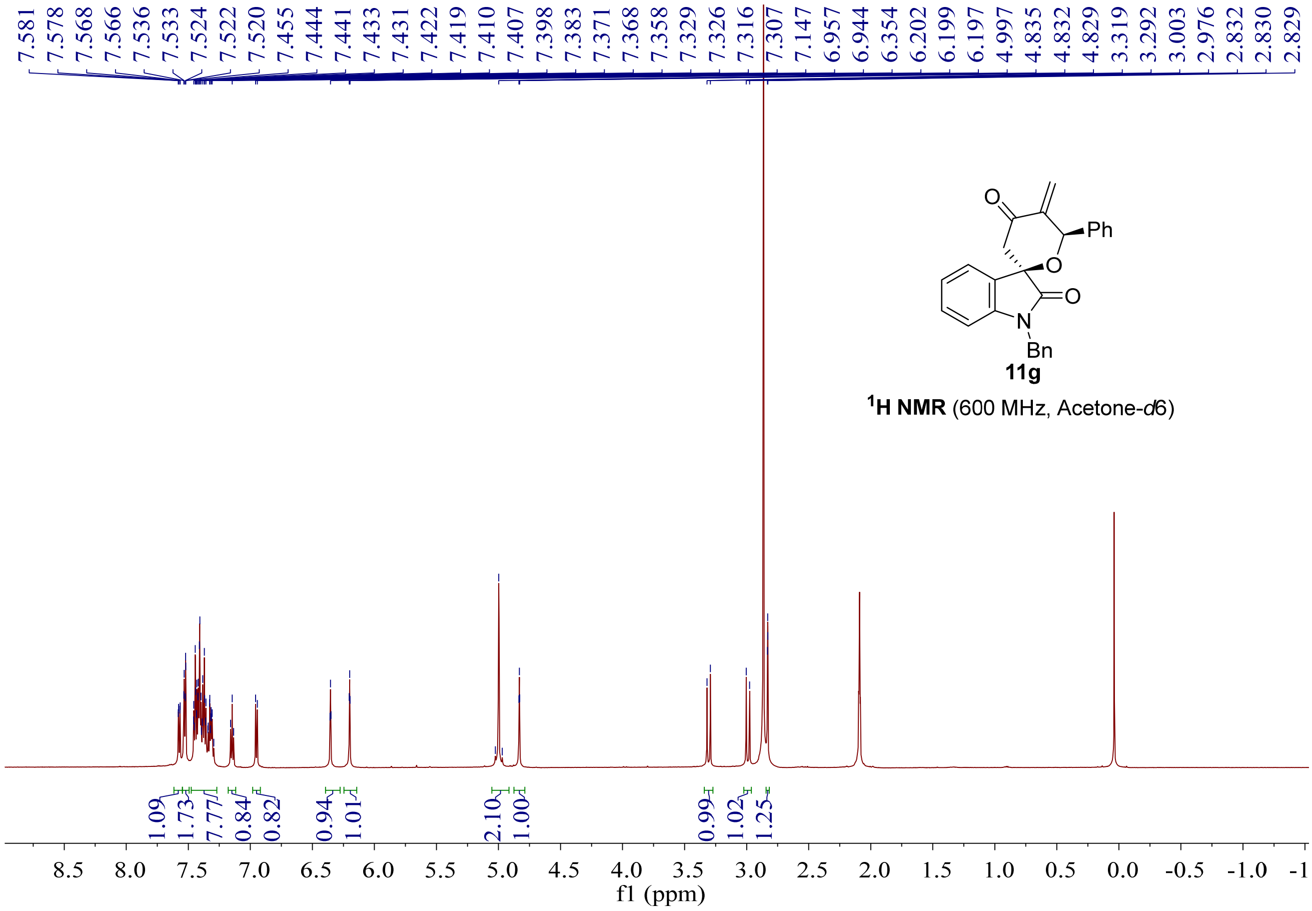
7.581
7.578
7.568
7.566
7.536
7.533
7.524
7.522
7.520
7.455
7.444
7.441
7.433
7.431
7.422
7.419
7.410
7.407
7.398
7.383
7.371
7.368
7.358
7.329
7.326
7.316
7.307
7.147
6.957
6.944
6.354
6.202
6.199
6.197
4.997
4.835
4.832
4.829
3.319
3.292
3.003
2.976
2.832
2.830
2.829
O
Ph
O
O
N
Bn
11g
1H NMR (600 MHz, Acetone-d6)
1.09
1.73
7.77
0.84
0.82
0.94
1.01
2.10
1.00
0.99
1.02
1.25
8.5 8.0 7.5 7.0 6.5 6.0 5.5 5.0 4.5 4.0 3.5 3.0 2.5 2.0 1.5 1.0 0.5 0.0 -0.5 -1.0 -1
f1 (ppm)

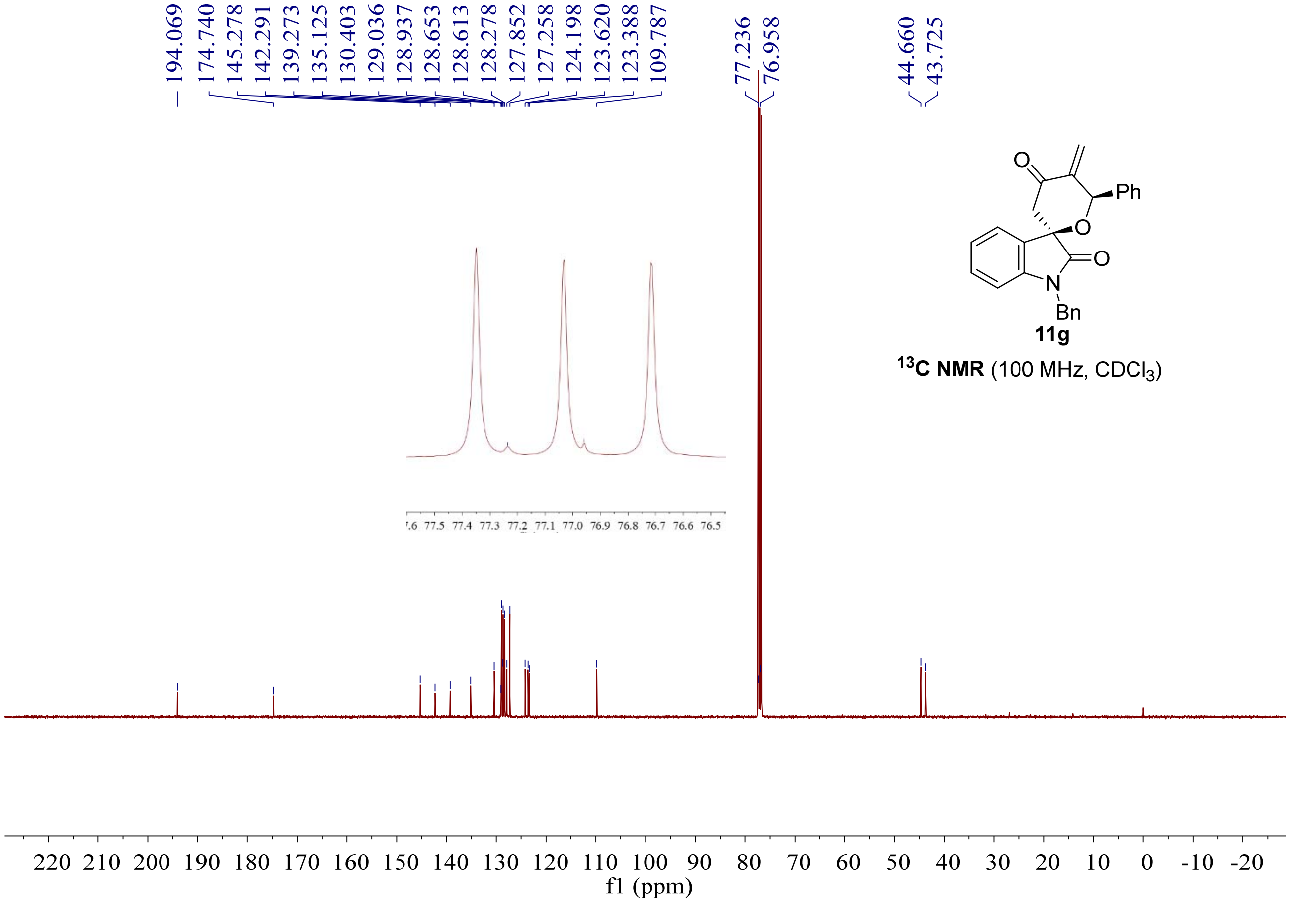
194.069
174.740
145.278
142.291
139.273
135.125
130.403
129.036
128.937
128.653
128.613
128.278
127.852
127.258
124.198
123.620
123.388
109.787
77.236
76.958
44.660
43.725
O
Ph
O
O
N
Bn
11g
13C NMR (100 MHz, CDCl3)
77.5 77.4 77.3 77.2 77.1 77.0 76.9 76.8 76.7 76.6 76.5
220 210 200 190 180 170 160 150 140 130 120 110 100 90 80 70 60 50 40 30 20 10 0 -10 -20
f1 (ppm)

Daicel Chiral IE Column, *i*PrOH/*n*-hexane = 40/60, 1.0 mL/min

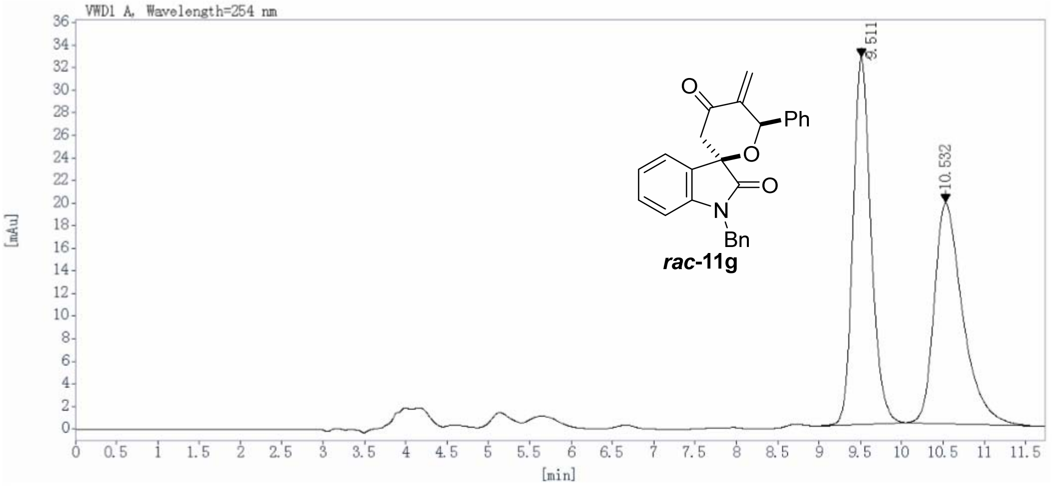

Ret Time [min]	Peak Type	Width [min]	Height [mAU]	Area [mAU*s]	Area [%]
9.511	BB	0.23	32.5346	493.3185	51.1522
10.532	BB	0.36	19.5491	471.0941	48.8478
			Totals:	964.4126	100.0000

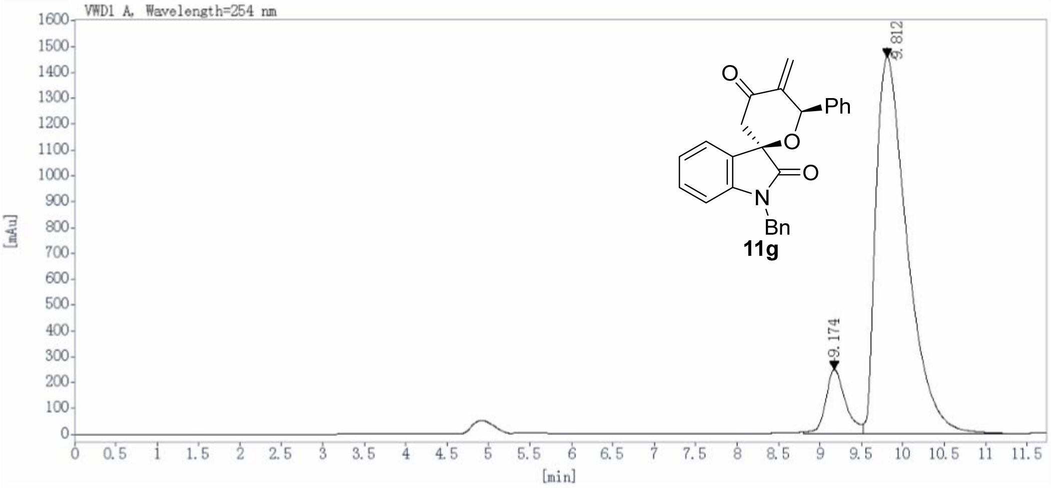

Ret Time [min]	Peak Type	Width [min]	Height [mAU]	Area [mAU*s]	Area [%]
9.174	FM	0.28	248.4929	4184.3003	9.8315
9.812	FM	0.44	1459.1060	38375.8711	90.1685
			Totals:	42560.1714	100.0000

HRMS (ESI-TOF) m/z: $[M + H]^+$ Calcd for $C_{26}H_{22}O_3N^+$ 396.1594; Found 396.1590.

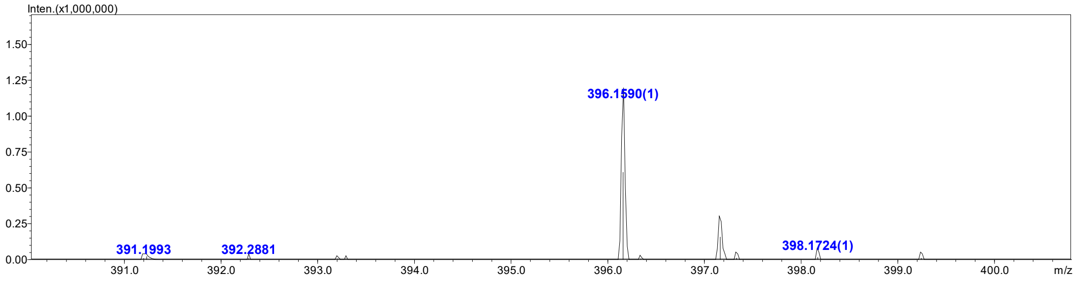

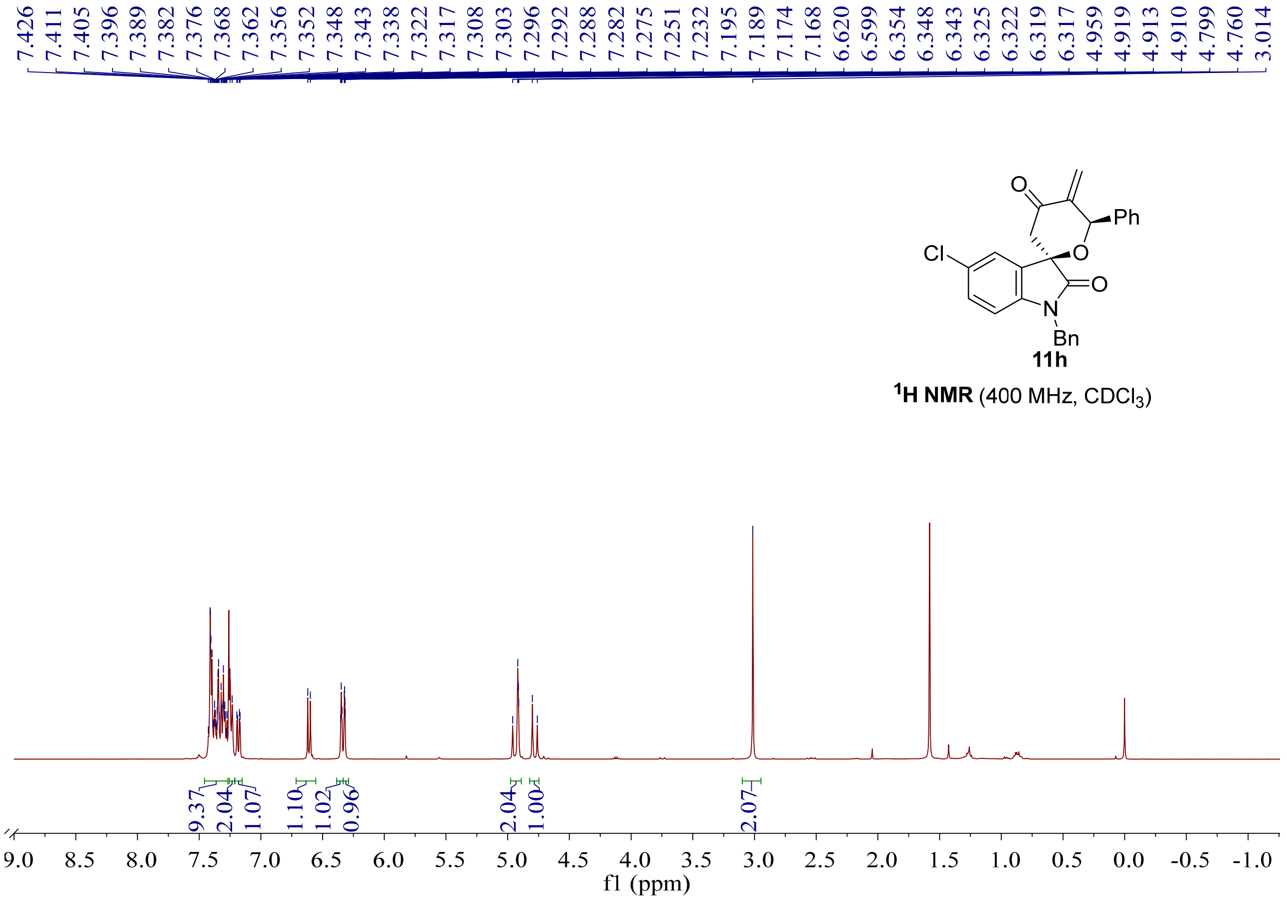
7.426 7.411 7.405 7.396 7.389 7.382 7.376 7.368 7.362 7.356 7.352 7.348 7.343 7.338 7.322 7.317 7.308 7.303 7.296 7.292 7.288 7.282 7.275 7.251 7.232 7.195 7.189 7.174 7.168 6.620 6.599 6.354 6.348 6.343 6.325 6.322 6.319 6.317 4.959 4.919 4.913 4.910 4.799 4.760 3.014
O
Ph
Cl
O
O
N
Bn
11h
1H NMR (400 MHz, CDCl3)
9.37 2.04 1.07 1.10 1.02 0.96 2.04 1.00 2.07
9.0 8.5 8.0 7.5 7.0 6.5 6.0 5.5 5.0 4.5 4.0 3.5 3.0 2.5 2.0 1.5 1.0 0.5 0.0 -0.5 -1.0
f1 (ppm)

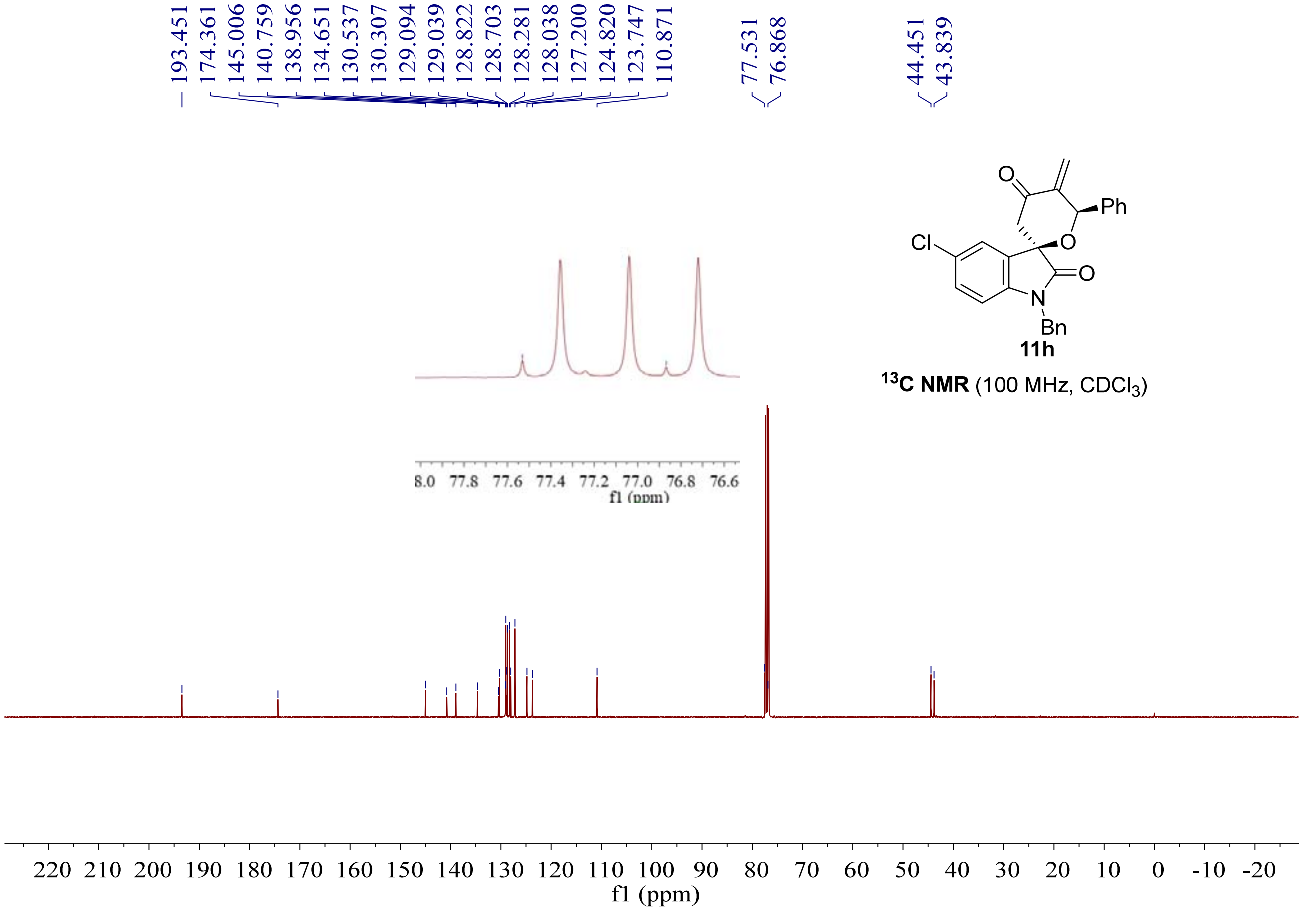
193.451 174.361 145.006 140.759 138.956 134.651 130.537 130.307 129.094 129.039 128.822 128.703 128.281 128.038 127.200 124.820 123.747 110.871 77.531 76.868 44.451 43.839
O
Ph
Cl
O
O
N
Bn
11h
13C NMR (100 MHz, CDCl3)
8.0 77.8 77.6 77.4 77.2 77.0 76.8 76.6
f1 (ppm)
220 210 200 190 180 170 160 150 140 130 120 110 100 90 80 70 60 50 40 30 20 10 0 -10 -20
f1 (ppm)

Daicel Chiral IE Column, *i*PrOH/*n*-hexane = 40/60, 1.0 mL/min

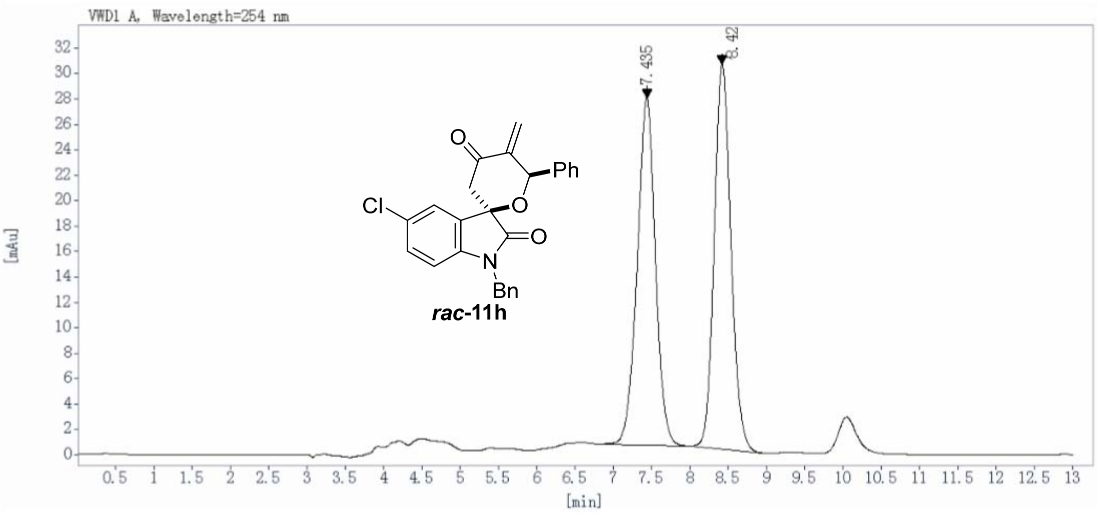

Ret Time [min]	Peak Type	Width [min]	Height [mAU]	Area [mAU*s]	Area [%]
7.435	BB	0.25	27.2954	453.8254	49.5680
8.420	BB	0.23	30.2786	461.7361	50.4320
			Totals:	915.5615	100.0000

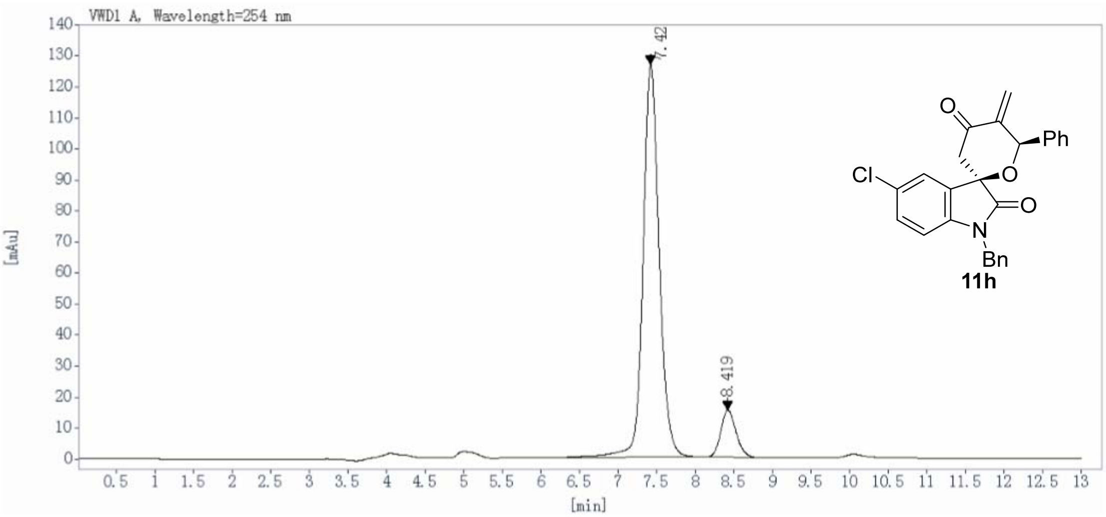

Ret Time [min]	Peak Type	Width [min]	Height [mAU]	Area [mAU*s]	Area [%]
7.420	BB	0.22	126.7034	1858.7831	89.8411
8.419	BB	0.21	15.1997	210.1843	10.1589
			Totals:	2068.9674	100.0000

HRMS (ESI-TOF) m/z: $[M + Na]^+$ Calcd for $C_{26}H_{20}O_3NClNa^+$ 452.1024 (^{35}Cl) and 454.0994 (^{37}Cl); Found 452.1008 (^{35}Cl) and 454.0995 (^{37}Cl).

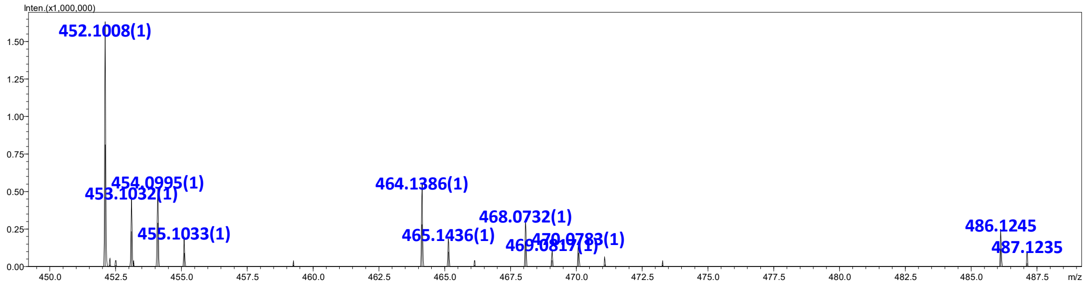

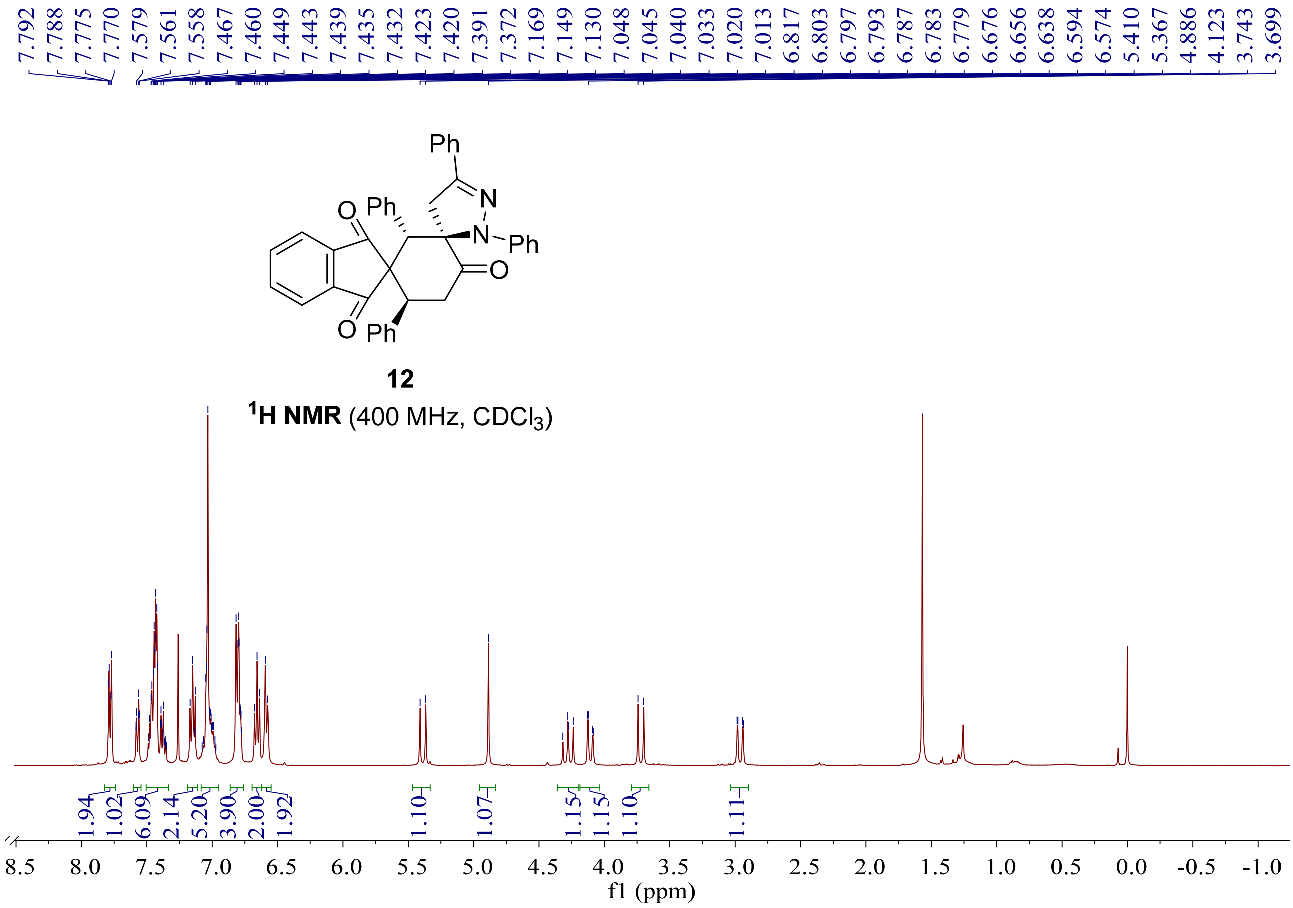
7.792
7.788
7.775
7.770
7.579
7.561
7.558
7.467
7.460
7.449
7.443
7.439
7.435
7.432
7.423
7.420
7.391
7.372
7.169
7.149
7.130
7.048
7.045
7.040
7.033
7.020
7.013
6.817
6.803
6.797
6.793
6.787
6.783
6.779
6.676
6.656
6.638
6.594
6.574
5.410
5.367
4.886
4.123
3.743
3.699
Ph
O
Ph
N
N
Ph
O
O
Ph
12
1H NMR (400 MHz, CDCl3)
1.94
1.02
6.09
2.14
5.20
3.90
2.00
1.92
1.10
1.07
1.15
1.15
1.10
1.11
f1 (ppm)

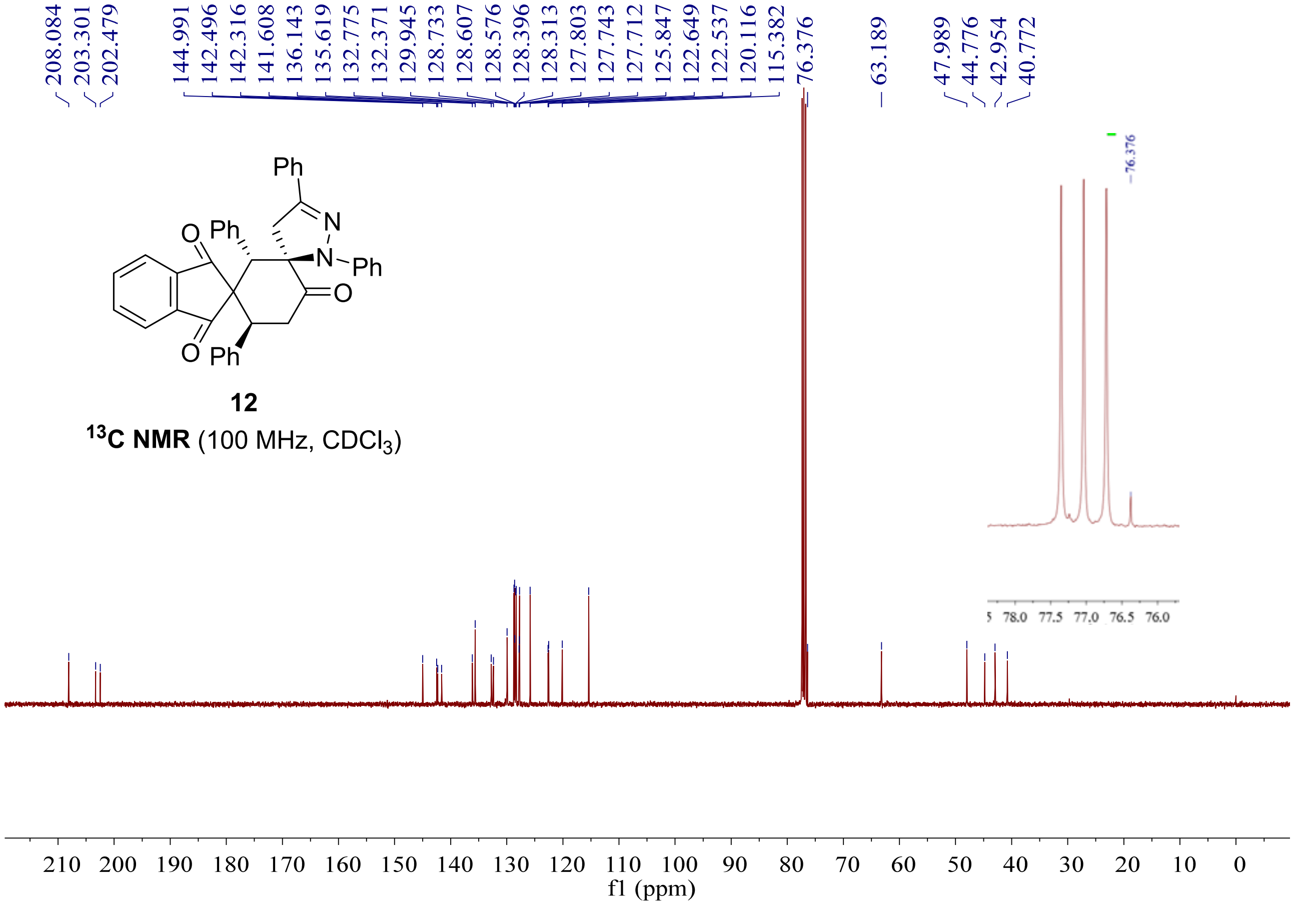
208.084
203.301
202.479
144.991
142.496
142.316
141.608
136.143
135.619
132.775
132.371
129.945
128.733
128.607
128.576
128.396
128.313
127.803
127.743
127.712
125.847
122.649
122.537
120.116
115.382
76.376
63.189
47.989
44.776
42.954
40.772
Ph
O
Ph
N
N
Ph
O
O
Ph
12
13C NMR (100 MHz, CDCl3)
f1 (ppm)

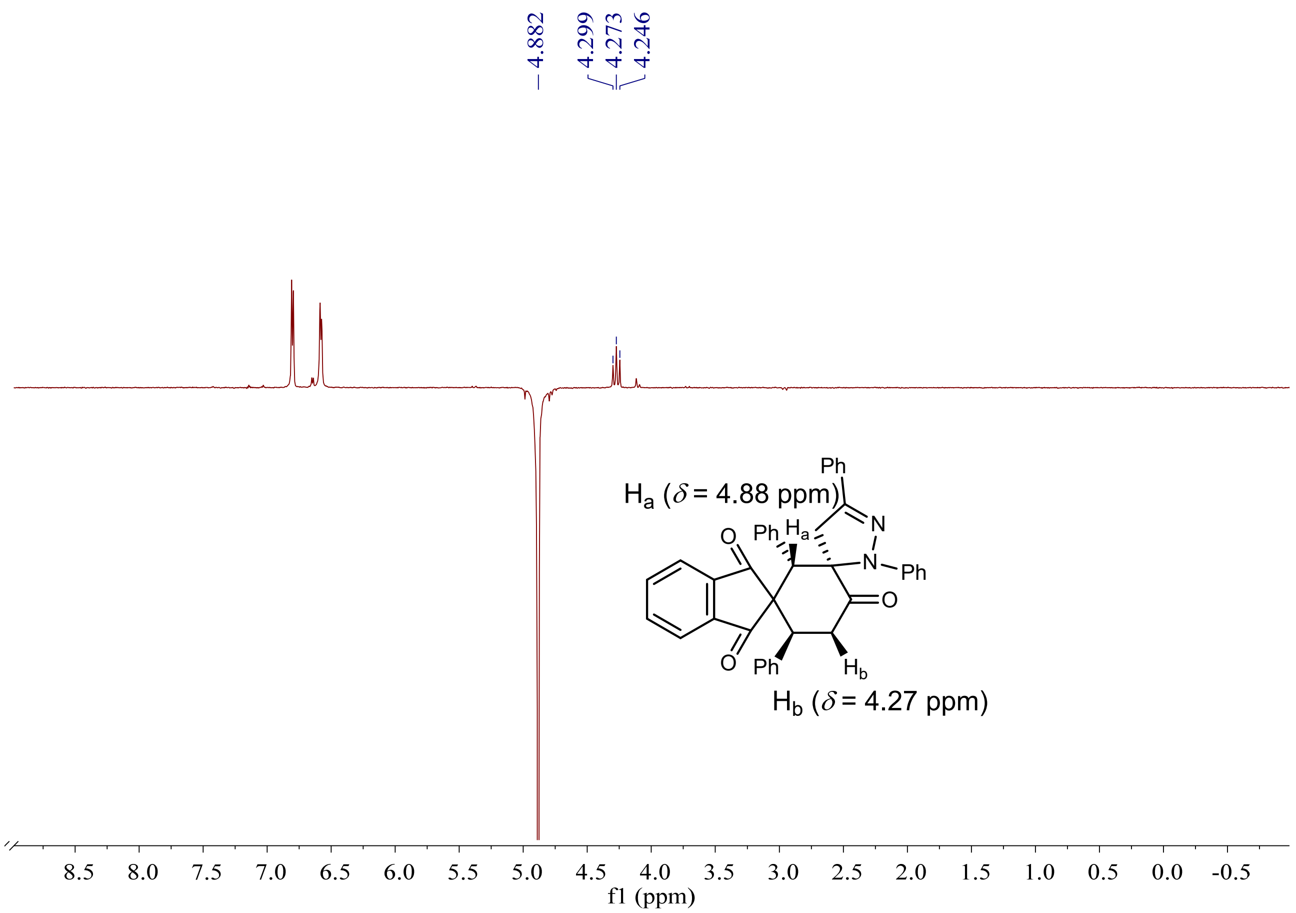

To confirm the stereoselectivity of this dipolar cycloaddition between **3a** and (*Z*)-N-phenylbenzohydrazonoyl chloride, the NOESY of H_a (δ = 4.88 ppm) was detected. The H_a showed sole relevant with H_b.

Daicel Chiral IE Column, *i*PrOH/*n*-hexane = 40/60, 1.0 mL/min.

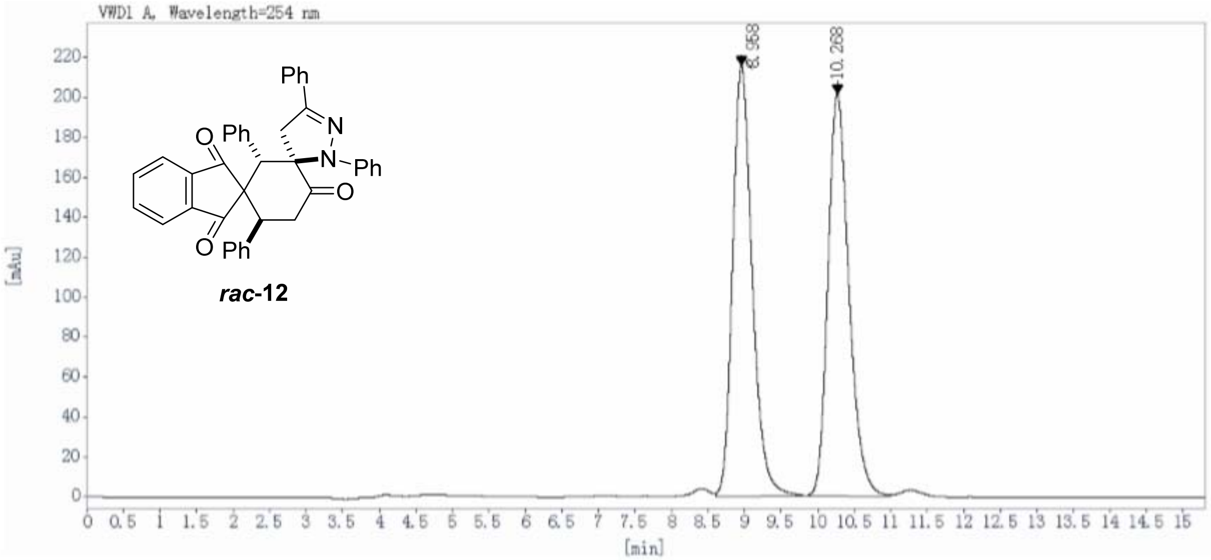

Ret Time [min]	Peak Type	Width [min]	Height [mAU]	Area [mAU*s]	Area [%]
8.958	MM	0.31	215.5126	3993.7952	49.8086
10.268	MF	0.33	201.0861	4024.4883	50.1914
			Totals:	8018.2834	100.0000

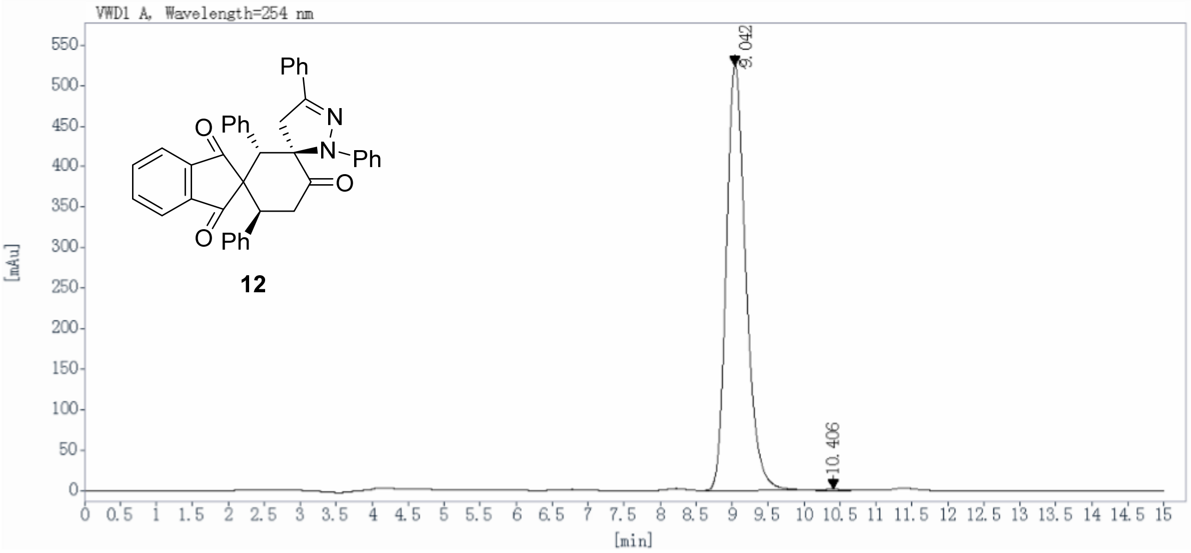

Ret Time [min]	Peak Type	Width [min]	Height [mAU]	Area [mAU*s]	Area [%]
9.042	BB	0.29	524.5488	9963.3438	99.6629
10.406	MM	0.33	1.6906	33.7049	0.3371
			Totals:	9997.0487	100.0000

HRMS (ESI-TOF) m/z: [M + Na]$^+$ Calcd for $C_{40}H_{30}N_2NaO_3^+$ 609.2149; Found 609.2136.

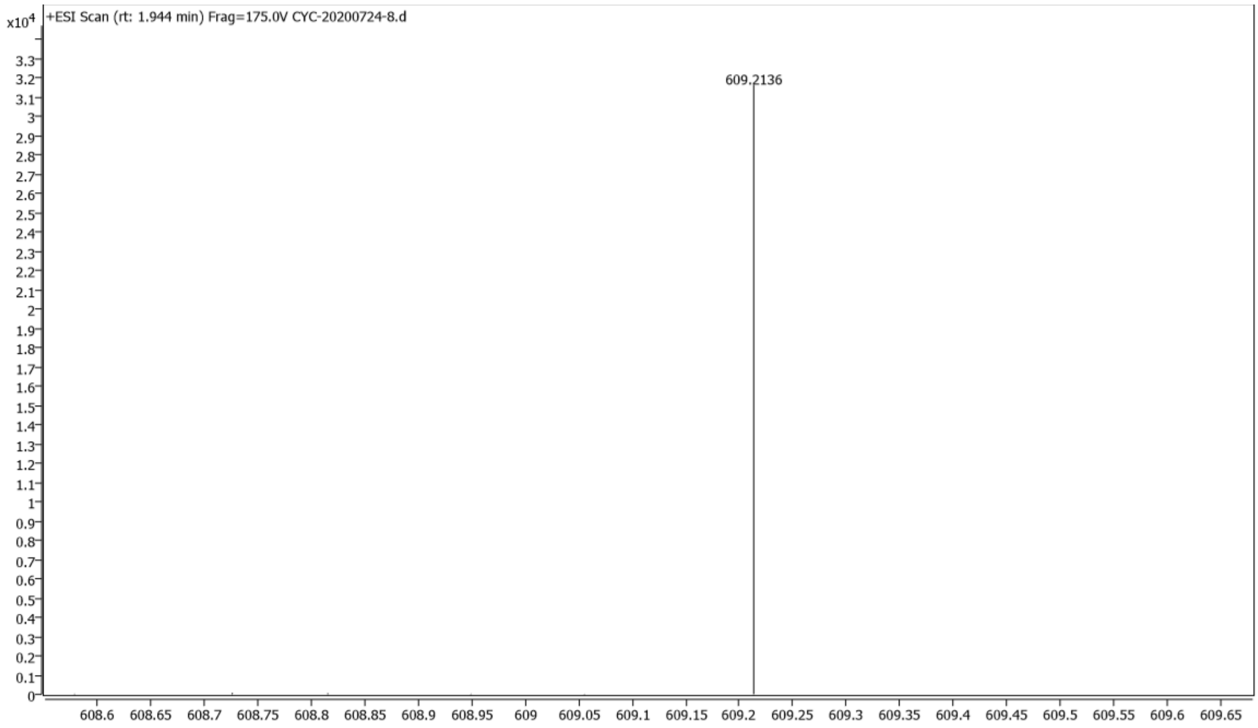

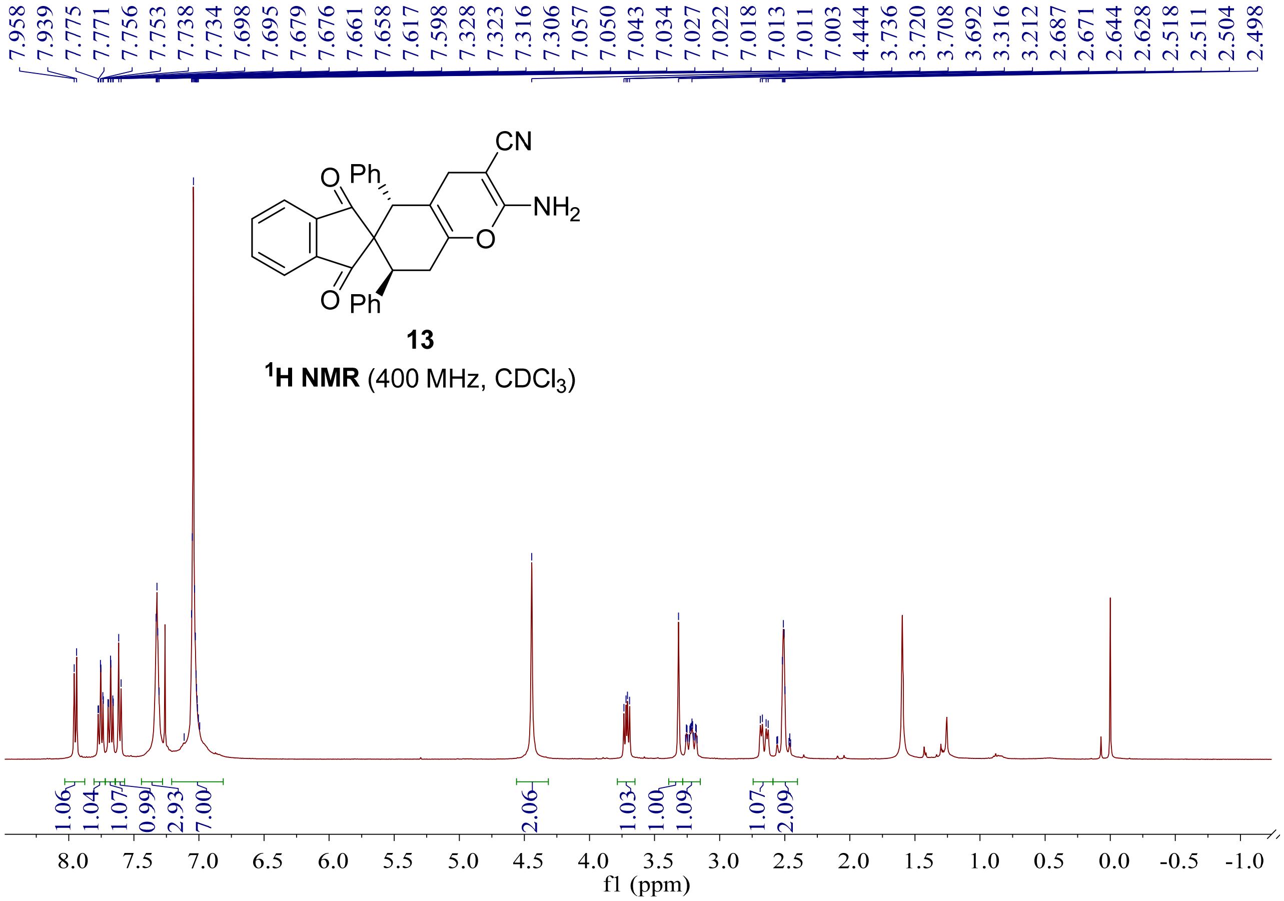
7.958 7.939 7.775 7.771 7.756 7.753 7.738 7.734 7.698 7.695 7.679 7.676 7.661 7.658 7.617 7.598 7.328 7.323 7.316 7.306 7.057 7.050 7.043 7.034 7.027 7.022 7.018 7.013 7.011 7.003 4.444 3.736 3.720 3.708 3.692 3.316 3.212 2.687 2.671 2.644 2.628 2.518 2.511 2.504 2.498
CN
Ph
O
NH2
O
Ph
O
13
1H NMR (400 MHz, CDCl3)
1.06 1.04 1.07 0.99 2.93 7.00 2.06 1.03 1.00 1.09 1.07 2.09
8.0 7.5 7.0 6.5 6.0 5.5 5.0 4.5 4.0 3.5 3.0 2.5 2.0 1.5 1.0 0.5 0.0 -0.5 -1.0
f1 (ppm)

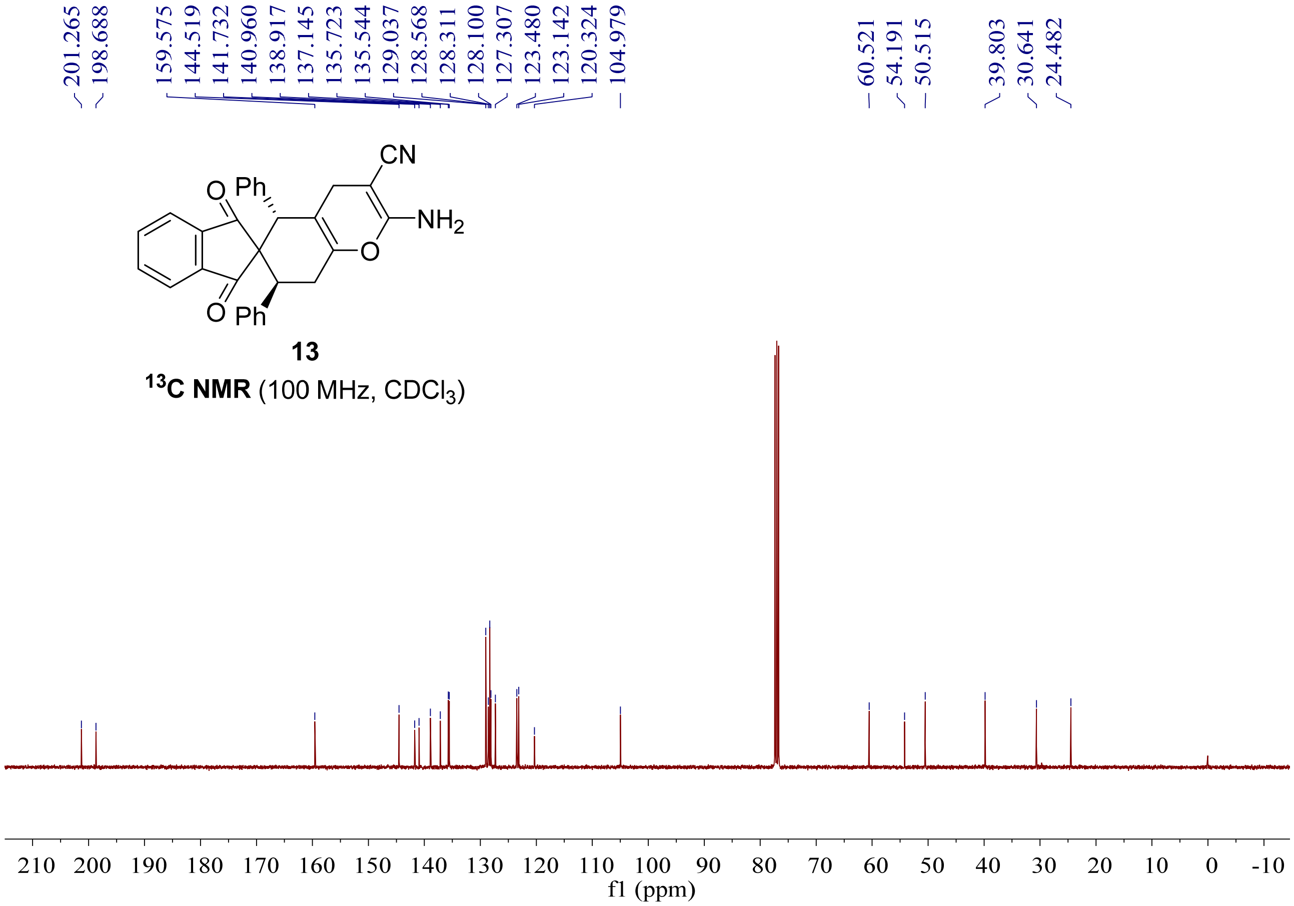
201.265 198.688 159.575 144.519 141.732 140.960 138.917 137.145 135.723 135.544 129.037 128.568 128.311 128.100 127.307 123.480 123.142 120.324 104.979 60.521 54.191 50.515 39.803 30.641 24.482
CN
Ph
O
NH2
O
Ph
O
13
13C NMR (100 MHz, CDCl3)
210 200 190 180 170 160 150 140 130 120 110 100 90 80 70 60 50 40 30 20 10 0 -10
f1 (ppm)

Daicel Chiral IE Column, *i*PrOH/*n*-hexane = 40/60, 1.0 mL/min.

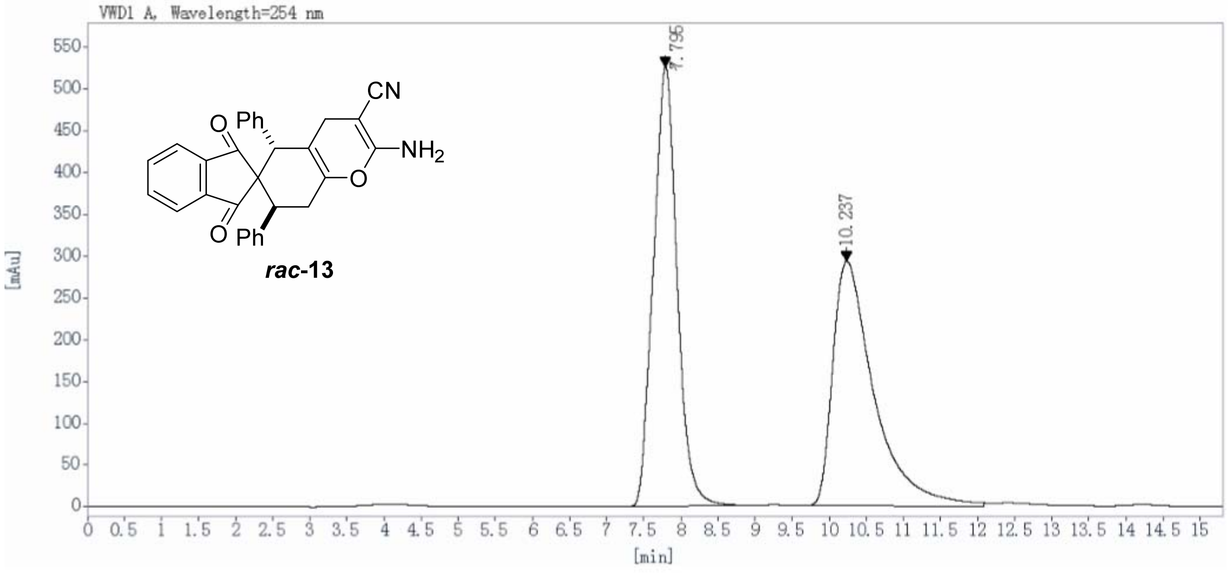

Ret Time [min]	Peak Type	Width [min]	Height [mAU]	Area [mAU*s]	Area [%]
7.795	BB	0.33	526.2322	11673.5518	50.7215
10.237	MF	0.65	292.9804	11341.4561	49.2785
			Totals:	23015.0078	100.0000

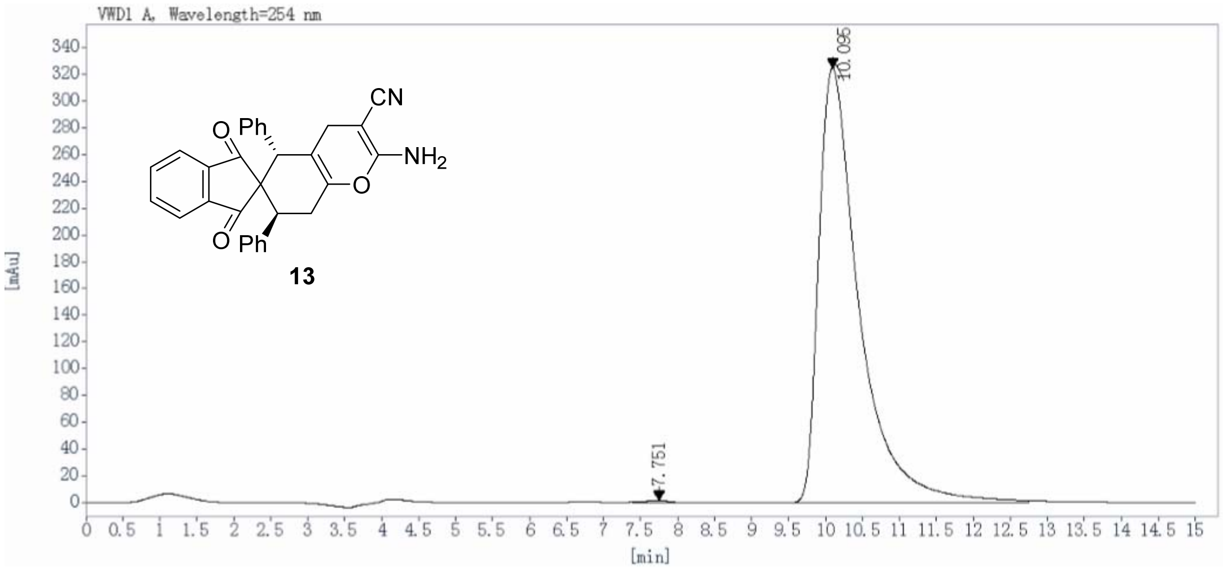

Ret Time [min]	Peak Type	Width [min]	Height [mAU]	Area [mAU*s]	Area [%]
7.751	MM	0.36	1.6782	36.1109	0.2922
10.095	MF	0.63	324.8903	12320.1855	99.7078
			Totals:	12356.2965	100.0000

HRMS (ESI-TOF) m/z: $[M + Na]^+$ Calcd for $C_{30}H_{22}NaN_2O_3^+$ 481.1523; Found 481.1520.

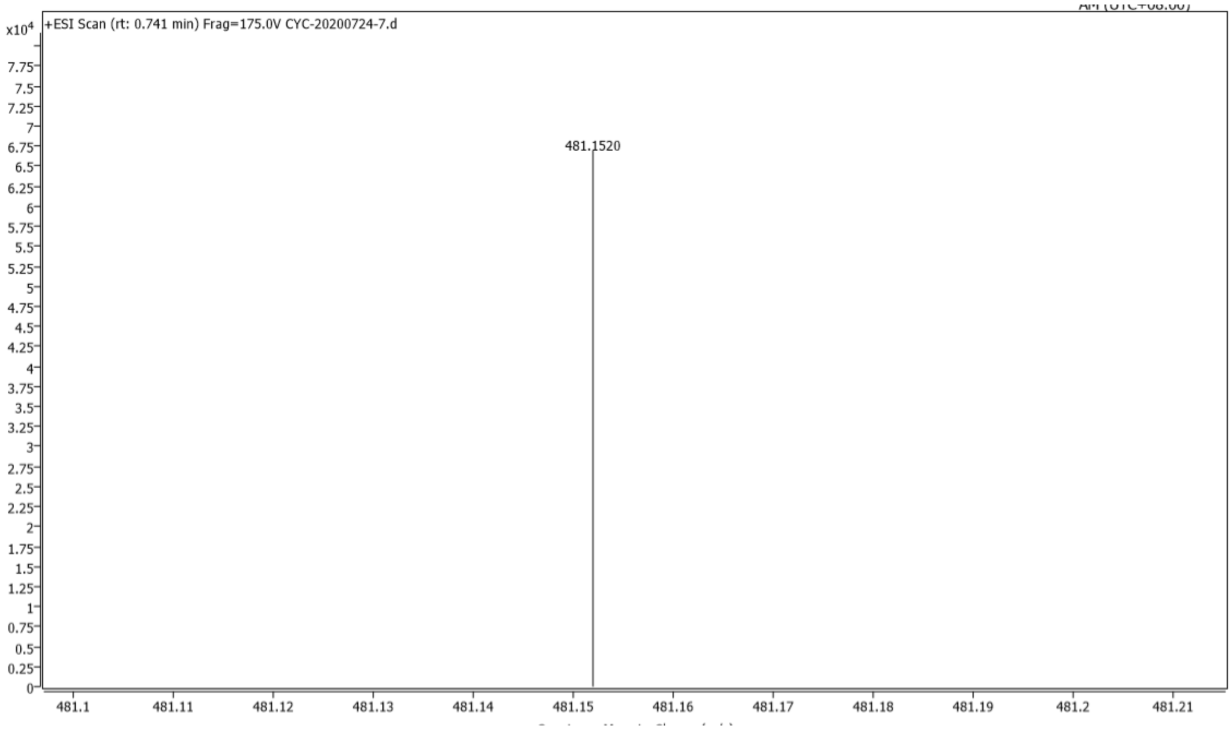

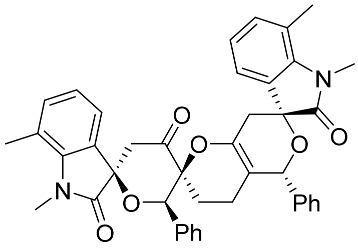

14

^{1}H NMR (400 MHz, $CDCl_3$)

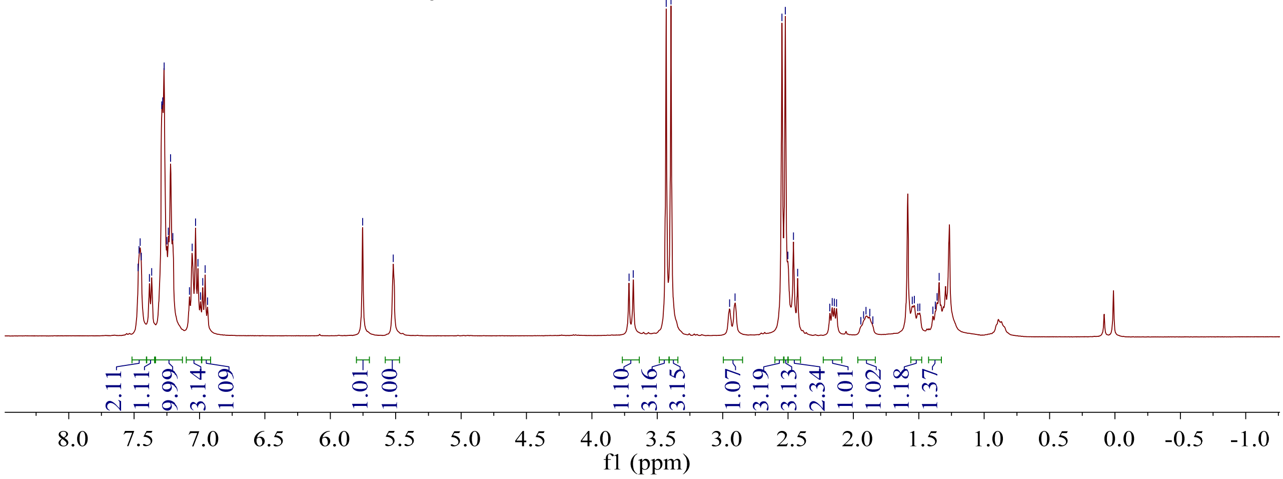

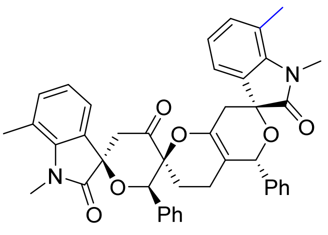

14

^{13}C NMR (150 MHz, $CDCl_3$)

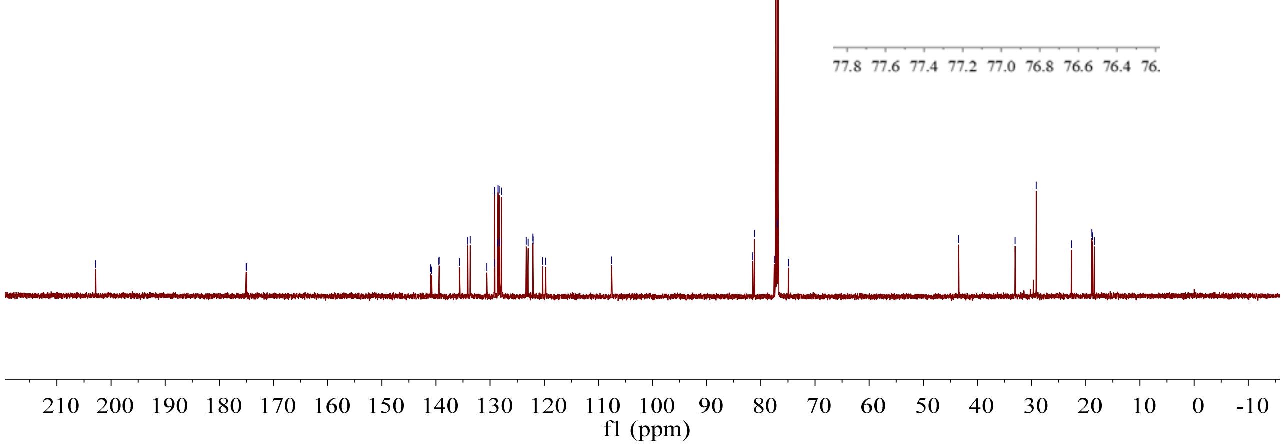

Daicel Chiral IE Column, *i*PrOH/*n*-hexane = 40/60, 1.0 mL/min

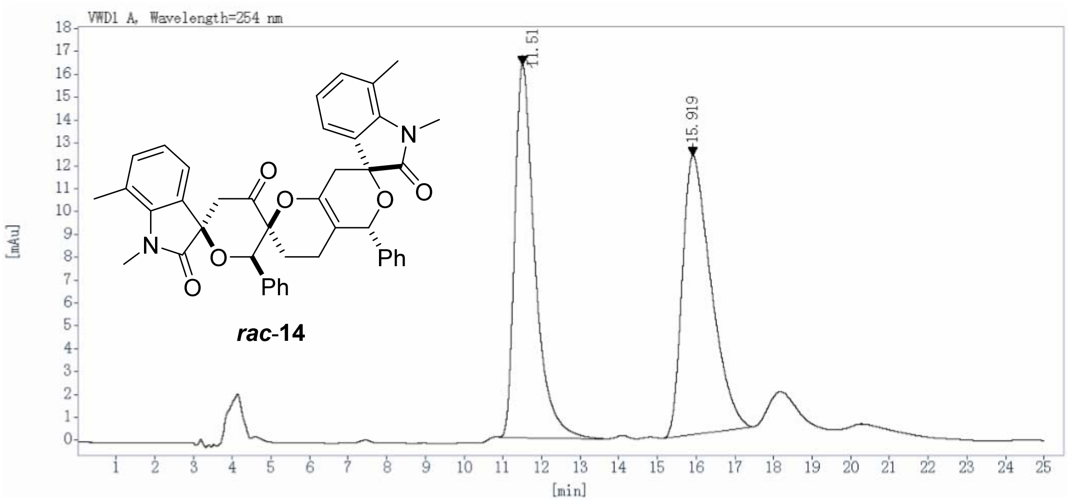

Ret Time [min]	Peak Type	Width [min]	Height [mAU]	Area [mAU*s]	Area [%]
11.510	BB	0.54	16.2930	589.8343	48.4264
15.919	BB	0.76	12.1743	628.1683	51.5736
			Totals:	1218.0026	100.0000

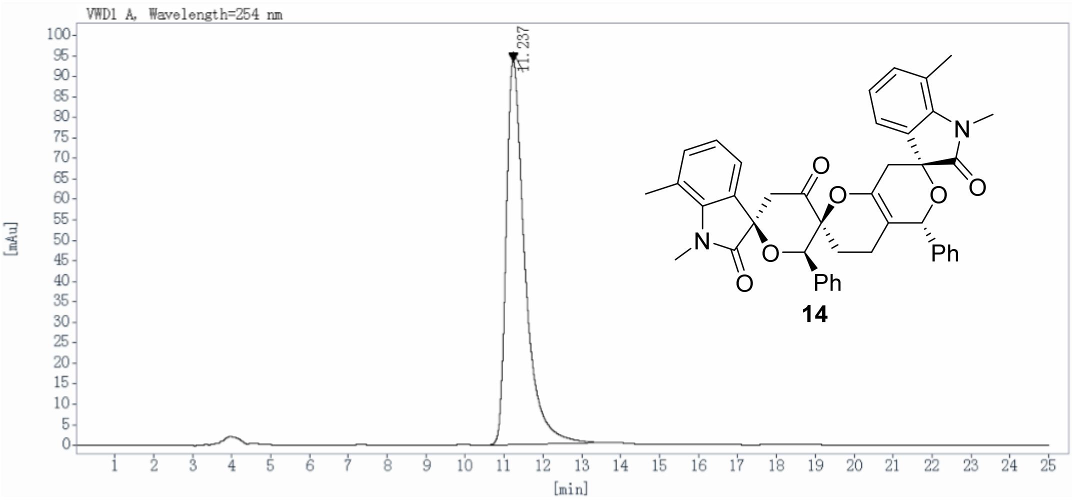

Ret Time [min]	Peak Type	Width [min]	Height [mAU]	Area [mAU*s]	Area [%]
11.237	BB	0.50	93.5078	3144.7483	100.0000
			Totals:	3144.7483	100.0000

HRMS (ESI-TOF) m/z: $[M + H]^+$ Calcd for $C_{42}H_{39}O_6N_2^+$ 667.2803; Found 667.2802.

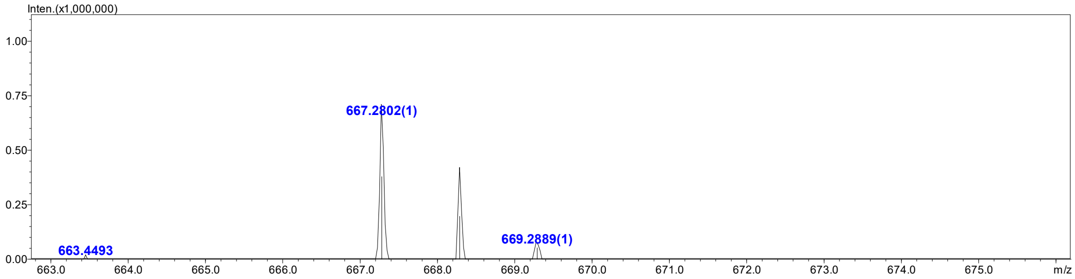

13.Computational methods and data

All calculations were carried out with the GAUSSIAN 09 packages.[7] The geometries of all intermediates and transition states were optimized using B3LYP-D3[8] functional together with SDD basis set for Pd atom and the standard 6-31G(d) basis set for the others.[9]All the optimized structures were calculated after considering various conformations and confirmed by frequency calculations to be either minima or transition states using the same level of theory. For transition states, intrinsic reaction coordinate analysis (IRC) was done to verify that they connect the corresponding reactants and products.[10] To take solvent effects into account, solution-phase single-point calculations were performed on the gas-phase geometries. The solution-phase single point energy calculations were done using B3LYP-D3 at SDD for Pd atom and 6-31++G(d, p) level for the others. Solvent effect was accounted for using self-consistent reaction field (SCRF) method, using SMD model.[11] Toluene was used as the solvent. Solution-phase single-point energies corrected by the gas-phase Gibbs free energy corrections were used to describe all the reaction energetics. All of these energies correspond to the reference state of 1 mol/L, 298 K. All energetics reported throughout the text are in kcal/mol, and the bond lengths are in angstroms (Å). Structures were generated using Gauss View 5.0.8 and CYL view.

Computational data:

INT1-A

Zero-point correction= 0.467626 (Hartree/Particle)
Thermal correction to Energy= 0.499184
Thermal correction to Enthalpy= 0.500128
Thermal correction to Gibbs Free Energy= 0.401058
E(solv) = -1890.93300443 A.U.

C	-3.158523	-1.316678	-0.674901
C	-3.565168	-2.677665	-0.237962
C	-4.844624	-3.100407	-0.203017
H	-5.652902	-2.446195	-0.506776
H	-5.088180	-4.105360	0.124048
C	-3.805722	-0.187277	-0.291335
H	-4.648724	-0.304920	0.386793
C	-3.475981	1.185700	-0.689766
C	-3.524390	2.224374	0.257953
C	-3.148974	1.517725	-2.018667

C	-3.225194	3.536065	-0.098353
H	-3.777115	1.986457	1.285879
C	-2.858965	2.833139	-2.378852
H	-3.160557	0.739747	-2.775431
C	-2.893877	3.847903	-1.420170
H	-3.253609	4.318162	0.655246
H	-2.615897	3.067190	-3.411446
H	-2.678238	4.874497	-1.703175
O	-2.504783	-3.442320	0.093366
Pd	-0.755347	-2.448891	-0.054244
C	0.630028	1.834867	2.173642
C	0.330265	0.489761	1.972234
C	-1.794744	0.762834	3.058936
C	-1.513539	2.113917	3.265309
C	-0.299751	2.645172	2.823275
H	1.570078	2.230737	1.807194
H	-2.734433	0.338260	3.399149
H	-2.235644	2.749813	3.768952
H	-0.073971	3.696300	2.976171
C	1.475271	1.320998	-1.018721
C	0.637198	3.565772	-0.875260
C	2.783640	1.770488	-0.860931
C	1.938997	4.037687	-0.698607
H	-0.205143	4.248764	-0.877977
C	3.007927	3.138731	-0.695913
H	3.605915	1.068066	-0.866218
H	2.121124	5.100324	-0.567668
H	4.025043	3.497153	-0.566747
O	1.314247	-0.299640	1.360012
O	1.210263	-0.046649	-1.200295
P	1.059728	-1.165854	-0.015463
C	-1.861532	-1.336200	-1.389931
H	-1.808747	-2.021217	-2.240800
H	-1.411179	-0.373240	-1.612848
C	-0.870639	-0.062347	2.415431
H	-1.085289	-1.113376	2.252627
C	0.397705	2.201451	-1.035276
H	-0.611637	1.826138	-1.149983
C	3.725934	-1.431079	-0.100645
C	4.390693	-1.430731	-1.324281
C	4.303947	-0.894431	1.047534
C	5.663973	-0.864398	-1.401233
H	3.903226	-1.858319	-2.193611
C	5.576086	-0.328043	0.955355

H	3.754548	-0.909103	1.980660
C	6.255699	-0.307495	-0.264937
H	6.189079	-0.854421	-2.351619
H	6.034696	0.099679	1.841916
H	7.244538	0.136319	-0.329247
O	2.453853	-2.010596	-0.046010

INT1-B

Zero-point correction= 0.467544 (Hartree/Particle)
Thermal correction to Energy= 0.499022
Thermal correction to Enthalpy= 0.499967
Thermal correction to Gibbs Free Energy= 0.401798
E(solv) = -1890.94210698 A.U.

C	3.257234	1.387567	-0.797840
C	3.515851	2.808111	-0.399761
C	2.284861	3.713276	-0.373303
H	2.102196	4.246574	-1.312701
H	2.459444	4.440628	0.425346
C	4.236001	0.475252	-0.668409
H	5.184844	0.867612	-0.303269
C	4.153022	-0.960520	-0.857480
C	5.063491	-1.778721	-0.168928
C	3.199859	-1.583697	-1.685352
C	4.996659	-3.161741	-0.266460
H	5.819156	-1.310106	0.455310
C	3.139585	-2.968407	-1.787435
H	2.528067	-0.980049	-2.277535
C	4.028697	-3.763876	-1.071895
H	5.703033	-3.773347	0.282427
H	2.400376	-3.426936	-2.436767
H	3.975917	-4.844682	-1.148928
O	4.607806	3.167718	0.004768
Pd	0.729674	2.478751	-0.066852
C	-0.372108	-2.190846	2.152769
C	-0.091704	-0.840286	1.965427
C	2.202461	-1.215259	2.598279
C	1.939596	-2.568185	2.772064
C	0.646793	-3.049508	2.558476
H	-1.379698	-2.543167	1.963842
H	3.201206	-0.824772	2.764665
H	2.733168	-3.243925	3.074294
H	0.429376	-4.101834	2.703519
C	-2.060612	-1.079568	-1.176611
C	-2.349191	-3.446821	-0.954404

C	-3.225837	-0.991732	-1.940111
C	-3.536009	-3.371189	-1.682995
H	-1.995823	-4.400987	-0.577609
C	-3.966405	-2.142221	-2.182997
H	-3.538900	-0.028458	-2.320092
H	-4.120444	-4.265473	-1.872091
H	-4.881760	-2.077054	-2.763753
O	-1.162954	-0.038896	1.569325
O	-1.293859	0.067952	-0.992138
P	-1.101481	1.025158	0.293977
C	1.842737	1.121827	-1.126165
H	1.590524	1.340132	-2.170501
H	1.503358	0.115221	-0.875066
C	1.185708	-0.337601	2.212739
H	1.396653	0.717719	2.096529
C	-1.598263	-2.299062	-0.702279
H	-0.663636	-2.347505	-0.159760
C	-3.784830	1.192753	0.486110
C	-4.638225	1.677202	-0.493897
C	-4.184228	0.208195	1.378686
C	-5.919746	1.146484	-0.598683
H	-4.288341	2.454841	-1.162095
C	-5.466916	-0.317300	1.257706
H	-3.490544	-0.136869	2.129842
C	-6.334829	0.142640	0.270279
H	-6.591404	1.516851	-1.364011
H	-5.790824	-1.092293	1.941634
H	-7.332548	-0.273957	0.184419
O	-2.523256	1.769642	0.553385

INT1-C

Zero-point correction= 0.465157 (Hartree/Particle)
Thermal correction to Energy= 0.497327
Thermal correction to Enthalpy= 0.498271
Thermal correction to Gibbs Free Energy= 0.397595
E(solv) = -1890.89657920 A.U.

C	2.936030	0.826875	-1.338490
C	2.867021	2.342734	-1.273719
C	4.097096	2.950105	-1.127945
H	5.017180	2.375430	-1.118792
H	4.171520	4.033155	-1.111573
C	3.592572	0.124362	-0.288070
H	3.990442	0.757455	0.501470
C	4.022821	-1.282645	-0.265583

C	4.161869	-1.925884	0.980993
C	4.320025	-2.013709	-1.430606
C	4.542146	-3.262073	1.058679
H	3.955752	-1.362013	1.887379
C	4.702066	-3.350457	-1.350734
H	4.278947	-1.517445	-2.394451
C	4.806058	-3.982780	-0.109149
H	4.636143	-3.741899	2.028663
H	4.933619	-3.897790	-2.260103
H	5.106468	-5.024806	-0.051083
O	1.709624	2.867919	-1.344365
Pd	1.386878	-0.076053	-0.042253
C	0.091548	2.908439	1.156438
C	-0.124412	1.623175	1.621793
C	2.101962	1.547004	2.561921
C	2.332780	2.839453	2.087152
C	1.329097	3.514368	1.388538
H	-0.659849	3.389294	0.542031
H	2.871640	1.019070	3.117726
H	3.300143	3.307478	2.230166
H	1.523430	4.494384	0.971159
C	-3.039376	-1.569886	0.901016
C	-4.765955	-1.148220	2.511115
C	-3.948184	-2.196380	0.053634
C	-5.698165	-1.749481	1.664164
H	-5.079886	-0.744250	3.469152
C	-5.285083	-2.276923	0.438605
H	-3.608622	-2.581628	-0.900699
H	-6.741499	-1.810239	1.958663
H	-6.004376	-2.746073	-0.225611
O	-1.284265	0.929818	1.222899
O	-1.697289	-1.498739	0.492430
P	-0.899163	-0.156375	0.012584
C	1.978515	0.116880	-2.113067
H	1.291579	0.711824	-2.707117
H	2.117950	-0.918909	-2.402973
C	0.869868	0.912282	2.324755
H	0.639302	-0.056404	2.760424
C	-3.424292	-1.057705	2.137039
H	-2.690771	-0.580070	2.774464
C	-3.081216	0.530973	-1.462623
C	-3.623318	-0.034736	-2.613077
C	-3.872256	1.212587	-0.539959
C	-4.997900	0.057957	-2.830179

H	-2.967802	-0.543925	-3.311681
C	-5.246737	1.284560	-0.764401
H	-3.422714	1.648115	0.343110
C	-5.813894	0.707653	-1.902246
H	-5.429484	-0.383849	-3.723555
H	-5.875245	1.794882	-0.040899
H	-6.885130	0.769828	-2.067931
O	-1.698289	0.396163	-1.292091

INT1-D

Zero-point correction= 0.465557 (Hartree/Particle)
Thermal correction to Energy= 0.497586
Thermal correction to Enthalpy= 0.498530
Thermal correction to Gibbs Free Energy= 0.398152
E(solv) = -1890.90070457 A.U.

C	-2.869947	-0.977513	-1.388811
C	-2.746491	-2.487066	-1.191921
C	-2.029778	-3.187007	-2.139604
H	-1.595704	-2.721275	-3.015461
H	-1.948547	-4.265123	-2.051580
C	-3.574122	-0.294154	-0.361905
H	-3.895658	-0.968185	0.431043
C	-4.109781	1.070129	-0.362973
C	-4.285341	1.725408	0.873941
C	-4.495775	1.749534	-1.534734
C	-4.780256	3.023623	0.933788
H	-4.013258	1.198872	1.784553
C	-4.995913	3.047978	-1.471661
H	-4.436981	1.239170	-2.490230
C	-5.130650	3.695180	-0.241270
H	-4.898753	3.512523	1.896696
H	-5.295593	3.552012	-2.386263
H	-5.522098	4.707213	-0.196274
O	-3.317081	-2.950488	-0.152403
Pd	-1.349969	0.062863	-0.193005
C	-0.374260	-2.710432	1.447158
C	0.043508	-1.396848	1.625118
C	-1.961672	-0.890819	2.883185
C	-2.389403	-2.198731	2.703320
C	-1.607370	-3.102528	1.971302
H	0.226792	-3.383061	0.846478
H	-2.562125	-0.184961	3.449499
H	-3.348790	-2.518372	3.095274
H	-1.969483	-4.105630	1.790105

C	3.035095	1.525616	0.906220
C	4.627060	1.034981	2.632202
C	4.015377	2.160304	0.149325
C	5.629936	1.643643	1.876240
H	4.860231	0.601653	3.600391
C	5.319412	2.210163	0.637858
H	3.755551	2.576359	-0.816920
H	6.648034	1.680691	2.251791
H	6.093671	2.686689	0.044444
O	1.241319	-0.971728	1.024613
O	1.727655	1.490045	0.394857
P	0.927224	0.171734	-0.153694
C	-2.019834	-0.203497	-2.226624
H	-1.306139	-0.701348	-2.872245
H	-2.279169	0.811016	-2.513235
C	-0.743683	-0.456849	2.321111
H	-0.345919	0.529794	2.539584
C	3.317031	0.975663	2.154053
H	2.531229	0.495887	2.724058
C	3.176900	-0.489482	-1.526304
C	3.781371	0.129773	-2.616229
C	3.915617	-1.209645	-0.589885
C	5.166981	0.050504	-2.756119
H	3.165573	0.669940	-3.327641
C	5.301177	-1.268125	-0.736209
H	3.416110	-1.685970	0.244221
C	5.930372	-0.638648	-1.812032
H	5.648120	0.534140	-3.601178
H	5.889275	-1.808517	-0.000762
H	7.009779	-0.690124	-1.917168
O	1.784442	-0.370083	-1.426665

INT1-A′

Zero-point correction= 0.467522 (Hartree/Particle)
Thermal correction to Energy= 0.499073
Thermal correction to Enthalpy= 0.500017
Thermal correction to Gibbs Free Energy= 0.400829
E(solv) = -1890.91943092 A.U.

C	-2.526928	-0.792464	-0.802278
C	-2.860149	-2.162429	-0.310323
C	-3.900648	-2.500553	0.479847
H	-4.679184	-1.802182	0.747287
H	-3.959824	-3.512063	0.867637
C	-3.166410	0.399224	-0.682455

H	-2.552044	1.272709	-0.902231
C	-4.554032	0.737622	-0.356638
C	-4.824194	1.983809	0.239074
C	-5.644684	-0.077893	-0.708547
C	-6.128884	2.376656	0.527459
H	-3.993237	2.641181	0.478755
C	-6.949487	0.318442	-0.429247
H	-5.457306	-1.023161	-1.205406
C	-7.198704	1.541652	0.199226
H	-6.311970	3.337874	1.000079
H	-7.777072	-0.326775	-0.710709
H	-8.218105	1.846788	0.417718
O	-1.907776	-3.063348	-0.639997
Pd	-0.084027	-2.199851	-0.514434
C	-0.030671	1.820971	1.359696
C	0.323361	0.488981	1.556097
C	-1.784325	0.034900	2.620170
C	-2.156062	1.367675	2.437563
C	-1.276481	2.256987	1.817385
H	0.659531	2.494174	0.864962
H	-2.471372	-0.670026	3.076024
H	-3.134475	1.705772	2.762570
H	-1.559821	3.296345	1.676332
C	2.195417	1.461777	-1.319194
C	1.272494	3.538072	-2.111787
C	3.091326	2.146385	-0.499225
C	2.149033	4.240759	-1.282210
H	0.568499	4.072745	-2.742481
C	3.058071	3.541698	-0.484736
H	3.786972	1.598405	0.122967
H	2.128337	5.326144	-1.261978
H	3.747778	4.081520	0.157458
O	1.584328	0.056605	1.116635
O	2.188635	0.068358	-1.376743
P	1.720114	-0.954792	-0.175320
C	-1.195784	-0.759241	-1.472393
H	-1.184396	-1.235249	-2.458116
H	-0.739481	0.229962	-1.508845
C	-0.532564	-0.408087	2.193815
H	-0.227018	-1.438637	2.338070
C	1.293903	2.143335	-2.134547
H	0.623991	1.577606	-2.773003
C	4.309265	-1.026365	0.412295
C	5.180204	-0.826818	-0.655510

C	4.597949	-0.557607	1.691605
C	6.370791	-0.133915	-0.433312
H	4.912948	-1.199810	-1.637702
C	5.792480	0.134242	1.899885
H	3.889486	-0.724471	2.494459
C	6.677284	0.349824	0.840607
H	7.055460	0.031749	-1.259630
H	6.028114	0.506599	2.892419
H	7.603525	0.891111	1.008024
O	3.114156	-1.722755	0.187002

INT1-B′

Zero-point correction= 0.467661 (Hartree/Particle)
Thermal correction to Energy= 0.499131
Thermal correction to Enthalpy= 0.500075
Thermal correction to Gibbs Free Energy= 0.401016
E(solv) = -1890.93217244 A.U.

C	-2.395918	-1.192605	-1.166966
C	-2.834763	-2.387510	-0.351664
C	-1.900027	-3.541079	-0.525702
H	-1.942882	-3.986961	-1.525733
H	-2.051077	-4.305772	0.242393
C	-2.854205	0.087537	-1.180794
H	-2.215866	0.762174	-1.753327
C	-3.981867	0.804132	-0.592231
C	-3.976327	2.206387	-0.751593
C	-5.047136	0.227586	0.126421
C	-4.970043	3.006146	-0.197704
H	-3.160327	2.668551	-1.302058
C	-6.044941	1.030352	0.673232
H	-5.054655	-0.842406	0.279212
C	-6.012666	2.419390	0.523016
H	-4.932910	4.084351	-0.327930
H	-6.854532	0.564967	1.229138
H	-6.792552	3.037281	0.959503
O	-3.737876	-2.379022	0.478945
Pd	-0.061755	-2.530729	-0.397273
C	-0.265991	1.717586	1.382662
C	0.098722	0.385389	1.558868
C	-2.084910	-0.141875	2.420296
C	-2.469216	1.187538	2.245107
C	-1.556137	2.113419	1.739370
H	0.447517	2.421944	0.973625
H	-2.796546	-0.875737	2.782346

H	-3.484491	1.494319	2.472370
H	-1.856033	3.146505	1.593418
C	1.807613	1.438619	-1.273288
C	0.618844	3.405361	-1.991520
C	2.703297	2.220222	-0.545595
C	1.495341	4.202764	-1.252315
H	-0.190014	3.859039	-2.556742
C	2.536899	3.606396	-0.538565
H	3.501924	1.753972	0.015322
H	1.370765	5.281236	-1.237063
H	3.227280	4.219251	0.033539
O	1.403781	-0.008122	1.206694
O	1.928015	0.048998	-1.318171
P	1.647461	-1.007355	-0.080993
C	-1.113345	-1.499972	-1.855166
H	-1.170243	-2.238443	-2.658982
H	-0.541938	-0.629915	-2.175466
C	-0.788951	-0.546068	2.094980
H	-0.473198	-1.573647	2.244437
C	0.773855	2.019746	-2.005543
H	0.101349	1.385734	-2.572217
C	4.245429	-0.719561	0.463458
C	5.090413	-0.473635	-0.615913
C	4.475262	-0.156002	1.716515
C	6.190492	0.365291	-0.434423
H	4.870917	-0.925579	-1.576725
C	5.578925	0.682180	1.884029
H	3.788871	-0.362400	2.529096
C	6.434548	0.946795	0.811852
H	6.852613	0.568173	-1.270798
H	5.766232	1.130759	2.855122
H	7.289646	1.601979	0.947853
O	3.144808	-1.562189	0.273057

INT1-C′

Zero-point correction= 0.465481 (Hartree/Particle)
Thermal correction to Energy= 0.497680
Thermal correction to Enthalpy= 0.498624
Thermal correction to Gibbs Free Energy= 0.397855
E(solv)= -1890.9010301 A.U.

C	-2.577099	-2.027372	-0.906980
C	-2.580930	-2.562728	0.516406
C	-3.202122	-3.745248	0.752569
H	-3.674804	-4.296856	-0.054094

H	-3.235128	-4.164966	1.753206
C	-3.280226	-0.858871	-1.290597
H	-3.228572	-0.602585	-2.351187
C	-4.318570	-0.129177	-0.561915
C	-5.118872	0.764417	-1.300534
C	-4.550592	-0.256792	0.823237
C	-6.138329	1.489610	-0.692176
H	-4.939495	0.876484	-2.367559
C	-5.585642	0.458455	1.421612
H	-3.882717	-0.867523	1.418191
C	-6.382607	1.329233	0.675037
H	-6.747596	2.168561	-1.282037
H	-5.759868	0.344511	2.488109
H	-7.184289	1.884844	1.154027
O	-1.963724	-1.744161	1.314268
Pd	-1.083481	-0.423760	-0.842364
C	-0.905850	1.319292	2.169126
C	-0.209970	1.446325	0.970571
C	-2.102791	2.568802	-0.045739
C	-2.803059	2.446138	1.148028
C	-2.199404	1.828711	2.250423
H	-0.451583	0.779445	2.991194
H	-2.561704	3.044179	-0.906551
H	-3.824796	2.800784	1.215710
H	-2.758710	1.707598	3.172507
C	3.378329	1.144658	-1.145087
C	4.613423	2.974165	-0.204652
C	4.556986	0.531838	-1.560087
C	5.807315	2.361833	-0.588470
H	4.631505	3.926565	0.317056
C	5.775079	1.143648	-1.271078
H	4.509355	-0.422631	-2.070842
H	6.758564	2.833371	-0.361059
H	6.699386	0.661530	-1.574179
O	1.071934	0.896244	0.867102
O	2.162157	0.512737	-1.448973
P	1.147040	-0.215699	-0.386941
C	-1.475250	-2.345287	-1.749133
H	-0.801121	-3.139789	-1.444638
H	-1.497592	-2.108674	-2.813103
C	-0.792453	2.069993	-0.152049
H	-0.195109	2.266244	-1.037894
C	3.386333	2.371132	-0.485480
H	2.452802	2.830267	-0.184588

C	3.317765	-1.421464	0.708621
C	4.158341	-2.400818	0.188205
C	3.769130	-0.483745	1.634731
C	5.496074	-2.422113	0.582792
H	3.759486	-3.120555	-0.518770
C	5.112166	-0.510223	2.009076
H	3.088430	0.260320	2.028347
C	5.978280	-1.471508	1.484439
H	6.160880	-3.180287	0.179408
H	5.480320	0.228546	2.714419
H	7.022286	-1.483962	1.782384
O	1.987367	-1.436622	0.270034

INT1-D′

Zero-point correction= 0.465431 (Hartree/Particle)
Thermal correction to Energy= 0.497626
Thermal correction to Enthalpy= 0.498570
Thermal correction to Gibbs Free Energy= 0.396690
E(solv) = -1890.90025307 A.U.

C	2.697685	1.976833	-1.008161
C	3.135603	2.707772	0.256337
C	2.102659	3.074698	1.116849
H	1.054448	3.000934	0.853275
H	2.352716	3.518067	2.075478
C	3.316797	0.823289	-1.548925
H	3.010479	0.565215	-2.563362
C	4.324112	-0.071723	-1.007620
C	4.638086	-1.213434	-1.782110
C	4.938368	0.070860	0.256944
C	5.513417	-2.184863	-1.314453
H	4.170221	-1.332739	-2.756820
C	5.829640	-0.899753	0.708573
H	4.746508	0.964105	0.838378
C	6.113399	-2.032001	-0.058503
H	5.734230	-3.055956	-1.924794
H	6.306161	-0.769550	1.676489
H	6.799467	-2.788250	0.312892
O	4.373498	2.872537	0.423409
Pd	1.093877	0.525009	-0.585169
C	0.341042	-1.060765	2.732762
C	0.095042	-1.368025	1.398205
C	2.401160	-2.035428	1.092594
C	2.657459	-1.719834	2.425100
C	1.625087	-1.254743	3.244769

H	-0.459616	-0.654119	3.340927
H	3.196454	-2.402583	0.453375
H	3.661878	-1.830087	2.818849
H	1.826105	-1.007110	4.282585
C	-3.330652	-1.108481	-1.326720
C	-4.729111	-2.925449	-0.624340
C	-4.433602	-0.416157	-1.817147
C	-5.851386	-2.235408	-1.084947
H	-4.840176	-3.903890	-0.166193
C	-5.699483	-0.983622	-1.685743
H	-4.293912	0.561782	-2.262720
H	-6.839819	-2.672529	-0.980825
H	-6.567572	-0.441341	-2.047662
O	-1.172607	-1.116979	0.862656
O	-2.064135	-0.518017	-1.471755
P	-1.124408	0.104418	-0.290127
C	1.462515	2.317707	-1.649746
H	0.894875	3.165841	-1.279898
H	1.312054	2.076817	-2.702158
C	1.110658	-1.873381	0.566297
H	0.871519	-2.215076	-0.437264
C	-3.455304	-2.367951	-0.746843
H	-2.575339	-2.883768	-0.383224
C	-3.366196	1.288643	0.717635
C	-4.129782	2.342381	0.223715
C	-3.925407	0.290237	1.512888
C	-5.494701	2.380995	0.508394
H	-3.650677	3.105951	-0.380045
C	-5.293860	0.335204	1.777125
H	-3.306897	-0.516362	1.885160
C	-6.081560	1.373419	1.276103
H	-6.098572	3.197566	0.123652
H	-5.743968	-0.450246	2.376558
H	-7.146138	1.399859	1.487664
O	-2.003849	1.295539	0.395089

PB-INT1-A1

Zero-point correction= 0.467960 (Hartree/Particle)
Thermal correction to Energy= 0.501562
Thermal correction to Enthalpy= 0.502506
Thermal correction to Gibbs Free Energy= 0.398224
E(solv) = -4465.27360820 A.U.

C	-1.065025	-2.510987	-1.573138
C	-0.170014	-3.698290	-1.426645

C	-0.411373	-4.895398	-2.009516
H	-1.291256	-5.043277	-2.625674
H	0.264349	-5.731978	-1.861656
C	-2.407179	-2.610435	-1.403017
H	-2.804215	-3.620971	-1.314282
C	-3.407343	-1.542726	-1.341591
C	-4.707980	-1.790023	-1.827646
C	-3.163783	-0.283284	-0.757214
C	-5.704679	-0.819416	-1.772242
H	-4.924604	-2.763508	-2.262237
C	-4.163100	0.686613	-0.697367
H	-2.202297	-0.083292	-0.302216
C	-5.436274	0.432318	-1.211887
H	-6.694858	-1.040489	-2.165317
H	-3.944590	1.639103	-0.223939
H	-6.212990	1.191585	-1.161307
O	0.882677	-3.419189	-0.657424
Pd	0.990711	-1.423445	-0.117787
C	4.174614	0.492372	-0.396676
C	3.258418	1.542866	-0.394184
C	4.299636	2.525198	-2.325534
C	5.228617	1.482513	-2.345817
C	5.160772	0.471318	-1.384050
H	4.084160	-0.289578	0.351726
H	4.343139	3.312142	-3.073906
H	5.999494	1.455856	-3.111416
H	5.874528	-0.347541	-1.401245
C	-0.957064	0.642865	2.224149
C	-3.288701	1.022583	2.667192
C	-1.091868	-0.712587	2.526292
C	-3.451743	-0.335569	2.944951
H	-4.139624	1.696500	2.715654
C	-2.353859	-1.195275	2.875977
H	-0.224549	-1.362720	2.475995
H	-4.432277	-0.723272	3.206074
H	-2.472642	-2.253708	3.088189
O	2.279979	1.609679	0.600063
O	0.283661	1.168553	1.851178
P	0.918190	0.701977	0.407662
C	-0.257933	-1.285846	-1.761506
H	0.477425	-1.407588	-2.565951
H	-0.812705	-0.360290	-1.888594
C	3.302914	2.557659	-1.348776
H	2.563585	3.351436	-1.317158

C	-2.038433	1.519847	2.296569
H	-1.891192	2.565895	2.051340
C	-0.485669	2.878707	-0.483528
C	-1.655115	3.155347	-1.196481
C	0.095642	3.849618	0.338264
C	-2.255220	4.406904	-1.077640
H	-2.086409	2.372984	-1.810931
C	-0.520530	5.098321	0.448141
H	0.998757	3.623872	0.889535
C	-1.692925	5.384878	-0.252742
H	-3.169242	4.613247	-1.628044
H	-0.074064	5.850795	1.092867
H	-2.164160	6.359079	-0.157104
O	0.036607	1.615137	-0.665154
Br	2.534813	-1.847698	1.921929

PB-INT1-A2

Zero-point correction= 0.467460 (Hartree/Particle)
Thermal correction to Energy= 0.501155
Thermal correction to Enthalpy= 0.502099
Thermal correction to Gibbs Free Energy= 0.396840
E(solv) = -4465.26568173 A.U.

C	2.792690	0.842589	-1.576925
C	1.848684	1.830740	-2.190290
C	2.177869	3.093891	-2.540380
H	3.191607	3.456726	-2.415227
H	1.439371	3.764142	-2.970353
C	3.572442	1.200721	-0.521414
H	3.590434	2.263537	-0.285780
C	4.358730	0.365362	0.380618
C	5.402261	0.965043	1.117682
C	4.084102	-0.996159	0.638500
C	6.155066	0.244590	2.040592
H	5.613972	2.019720	0.954640
C	4.839196	-1.714446	1.563274
H	3.239728	-1.483385	0.166848
C	5.882456	-1.107208	2.267421
H	6.953941	0.739946	2.588628
H	4.591395	-2.757338	1.746228
H	6.464308	-1.673313	2.990681
O	0.630006	1.296964	-2.338139
Pd	0.580962	-0.627784	-1.495557
C	2.550234	-0.501101	-2.128970
H	2.417511	-0.497679	-3.215340

H	3.206609	-1.304791	-1.809161
P	-1.536039	-0.438709	-0.578523
O	-2.540556	-1.737984	-0.610419
O	-1.569340	-0.110483	1.044777
O	-2.639669	0.690722	-1.123043
C	-2.641825	1.995842	-0.646194
C	-1.594752	2.864003	-0.952898
C	-3.722298	2.400759	0.137907
C	-1.637003	4.164488	-0.448476
H	-0.760048	2.498787	-1.551240
C	-3.754667	3.708872	0.624879
H	-4.504579	1.684431	0.367538
C	-2.710333	4.592293	0.337291
H	-0.813855	4.838786	-0.667817
H	-4.591116	4.032116	1.239654
H	-2.732351	5.606370	0.728141
C	-3.706081	-1.801463	0.138761
C	-4.899125	-1.303287	-0.385294
C	-3.667294	-2.407403	1.393977
C	-6.070558	-1.416073	0.365697
H	-4.889267	-0.830965	-1.360375
C	-4.844519	-2.516194	2.133476
H	-2.718234	-2.775677	1.767026
C	-6.047751	-2.020671	1.624479
H	-7.003160	-1.029483	-0.037138
H	-4.819287	-2.986013	3.113084
H	-6.962137	-2.105603	2.205500
C	-0.514416	0.525175	1.706075
C	0.697445	-0.134003	1.907390
C	-0.732836	1.812206	2.194896
C	1.714968	0.523989	2.601496
H	0.845210	-1.129224	1.502342
C	0.291695	2.454495	2.890357
H	-1.685563	2.296259	2.016610
C	1.517217	1.815535	3.091411
H	2.668334	0.025627	2.737778
H	0.130099	3.462722	3.263174
H	2.319531	2.323745	3.619568
Br	0.915165	-2.942572	-0.617113

PB-INT1-B1

Zero-point correction= 0.467905 (Hartree/Particle)
Thermal correction to Energy= 0.501373
Thermal correction to Enthalpy= 0.502317

Thermal correction to Gibbs Free Energy= 0.399203
E(solv) = -4465.28420826 A.U.

C	-1.167944	-2.203206	-1.524692
C	-0.390791	-3.418823	-1.055439
C	1.060908	-3.290758	-1.313714
H	1.317478	-3.181726	-2.373289
H	1.667347	-4.048750	-0.819057
C	-2.489295	-2.134717	-1.242854
H	-2.895904	-2.973087	-0.679993
C	-3.399986	-1.024964	-1.518546
C	-4.461540	-0.755889	-0.631147
C	-3.294426	-0.212739	-2.667156
C	-5.353162	0.288847	-0.860546
H	-4.555417	-1.365358	0.262044
C	-4.191744	0.827223	-2.901828
H	-2.508096	-0.418880	-3.385147
C	-5.225211	1.090944	-1.998019
H	-6.150614	0.481355	-0.146316
H	-4.084273	1.435831	-3.796675
H	-5.922280	1.904869	-2.180854
O	-0.916498	-4.319637	-0.398283
Pd	1.292261	-1.315993	-0.582752
C	4.090031	1.388601	-0.346139
C	3.011245	2.209359	-0.016236
C	3.807510	3.928162	-1.501254
C	4.896972	3.122835	-1.841045
C	5.030658	1.857138	-1.264675
H	4.163186	0.402372	0.104589
H	3.692579	4.911508	-1.950021
H	5.635496	3.478781	-2.554663
H	5.871375	1.222115	-1.530726
C	-0.779318	-0.311918	2.164291
C	-3.097714	-0.621127	2.715906
C	-0.591864	-1.691653	2.080547
C	-2.937139	-2.003702	2.599326
H	-4.069004	-0.200122	2.962650
C	-1.686381	-2.534661	2.277300
H	0.388966	-2.089827	1.845484
H	-3.785842	-2.665803	2.751198
H	-1.554569	-3.602528	2.142376
O	2.075991	1.789202	0.925337
O	0.286995	0.568756	1.946353
P	0.934802	0.683781	0.435128
C	-0.267988	-1.113767	-1.975067

H	0.230894	-1.298887	-2.934102
H	-0.725046	-0.126111	-1.978716
C	2.854441	3.471303	-0.589688
H	1.992734	4.073311	-0.320077
C	-2.019905	0.235561	2.488442
H	-2.130203	1.311862	2.548364
C	-1.051958	2.538767	0.247678
C	-2.375748	2.479242	-0.189361
C	-0.659564	3.451117	1.230473
C	-3.319113	3.341053	0.365012
H	-2.652580	1.744391	-0.934608
C	-1.617953	4.304808	1.780937
H	0.367237	3.463677	1.574180
C	-2.946776	4.255973	1.353831
H	-4.349280	3.277133	0.026938
H	-1.319470	5.008258	2.554062
H	-3.685887	4.921787	1.791456
O	-0.168444	1.655357	-0.345806
Br	3.223238	-1.936420	1.002335

PB-INT1-B2

Zero-point correction= 0.467811 (Hartree/Particle)
Thermal correction to Energy= 0.501332
Thermal correction to Enthalpy= 0.502277
Thermal correction to Gibbs Free Energy= 0.398378
E(solv) = -4465.27813399 A.U.

C	2.751770	0.268108	-1.623661
C	1.554436	0.909521	-2.296387
C	0.564788	-0.087456	-2.756405
H	0.948852	-0.782080	-3.510952
H	-0.381386	0.352333	-3.072545
C	3.603452	1.102303	-0.977192
H	3.333873	2.155965	-1.033498
C	4.759705	0.797289	-0.143911
C	5.194483	1.770593	0.782347
C	5.501655	-0.400697	-0.214155
C	6.286577	1.549918	1.616675
H	4.643001	2.705665	0.845082
C	6.596515	-0.619404	0.618042
H	5.222583	-1.154817	-0.939953
C	6.995177	0.348013	1.544227
H	6.585976	2.317687	2.326795
H	7.145783	-1.554886	0.540518
H	7.847022	0.169694	2.195562

O	1.363569	2.129584	-2.302913
Pd	0.572973	-1.329631	-1.054732
C	2.618837	-1.207740	-1.544065
H	2.726374	-1.733383	-2.500092
H	3.210951	-1.693801	-0.771934
P	-1.508485	-0.772783	-0.317678
O	-2.665605	-1.886877	0.080713
O	-1.504919	0.080746	1.103922
O	-2.425747	0.231916	-1.284881
C	-2.685170	1.572244	-1.029221
C	-1.776777	2.543396	-1.452114
C	-3.869431	1.923255	-0.378749
C	-2.068738	3.887037	-1.213874
H	-0.837070	2.265333	-1.921061
C	-4.145845	3.271672	-0.147938
H	-4.549746	1.145196	-0.053882
C	-3.247582	4.258012	-0.563772
H	-1.352816	4.641680	-1.526957
H	-5.064477	3.547858	0.363737
H	-3.463253	5.306765	-0.376212
C	-3.934364	-1.530738	0.502845
C	-4.984189	-1.577108	-0.415362
C	-4.157646	-1.153972	1.828369
C	-6.272396	-1.236575	-0.001937
H	-4.770920	-1.862395	-1.439578
C	-5.449111	-0.811308	2.229101
H	-3.319170	-1.118667	2.512760
C	-6.508789	-0.848315	1.319064
H	-7.090110	-1.265920	-0.717197
H	-5.625560	-0.509954	3.258214
H	-7.511738	-0.577110	1.637293
C	-0.472065	0.948900	1.475823
C	0.818495	0.473891	1.705723
C	-0.793868	2.293223	1.656204
C	1.814469	1.381082	2.071953
H	1.038135	-0.581506	1.590343
C	0.208562	3.184330	2.039269
H	-1.809377	2.627106	1.480038
C	1.516303	2.734473	2.235565
H	2.828296	1.021683	2.216044
H	-0.035808	4.235808	2.166855
H	2.299623	3.434784	2.514147
Br	0.959931	-3.121283	0.729308

PB-INT1-C1

Zero-point correction= 0.465952 (Hartree/Particle)
Thermal correction to Energy= 0.500259
Thermal correction to Enthalpy= 0.501203
Thermal correction to Gibbs Free Energy= 0.394289
E(solv) = -4465.25278250 A.U.

C	-3.140224	-1.083363	1.452666
C	-3.138803	-2.060723	2.629912
C	-3.111651	-1.447581	3.871461
H	-3.115354	-0.368770	3.987541
H	-3.194813	-2.052033	4.770524
C	-2.428240	0.162791	1.483222
H	-1.844817	0.334760	2.383307
C	-2.768826	1.405952	0.747098
C	-1.844377	2.467858	0.745874
C	-3.993767	1.601896	0.088560
C	-2.115261	3.659494	0.083640
H	-0.897456	2.332529	1.259340
C	-4.263214	2.794937	-0.581893
H	-4.743860	0.819962	0.121629
C	-3.325065	3.827405	-0.594689
H	-1.371673	4.451062	0.076439
H	-5.218172	2.920245	-1.086944
H	-3.533566	4.754025	-1.123157
O	-3.232989	-3.290343	2.340851
Pd	-1.366618	-1.125478	0.098575
C	-3.501677	-1.599061	0.186706
H	-3.760298	-2.653605	0.175843
H	-3.811648	-0.980403	-0.648864
P	0.711191	-0.279186	0.330525
O	2.002055	-1.275463	0.469280
O	1.356698	0.716774	-0.827030
O	0.895349	0.622562	1.716838
C	2.127975	1.059597	2.174899
C	2.625864	2.292185	1.755192
C	2.835648	0.267317	3.079222
C	3.858468	2.730924	2.240244
H	2.043634	2.887241	1.061749
C	4.065661	0.716792	3.559699
H	2.418292	-0.686978	3.380128
C	4.583370	1.944755	3.138352
H	4.252400	3.689223	1.912715
H	4.622046	0.102036	4.261782
H	5.543930	2.288431	3.511804

C	3.054903	-1.384425	-0.438970
C	4.300677	-0.883187	-0.068799
C	2.854366	-2.029568	-1.656930
C	5.376796	-1.040305	-0.943844
H	4.412084	-0.380126	0.884989
C	3.939245	-2.171729	-2.522146
H	1.861118	-2.393951	-1.907978
C	5.200232	-1.682552	-2.171040
H	6.353457	-0.653626	-0.664067
H	3.793409	-2.670097	-3.476715
H	6.039986	-1.799326	-2.851081
C	0.609642	1.607508	-1.586930
C	-0.528602	1.209306	-2.289837
C	1.086552	2.915721	-1.669564
C	-1.214915	2.158349	-3.046489
H	-0.860769	0.177436	-2.250060
C	0.399004	3.848539	-2.446664
H	1.997372	3.177667	-1.141947
C	-0.760767	3.475871	-3.127422
H	-2.114369	1.856989	-3.574957
H	0.770556	4.867901	-2.512996
H	-1.306267	4.206294	-3.718243
Br	-0.828732	-2.601245	-1.899194

PB-INT1-C2

Zero-point correction= 0.466150 (Hartree/Particle)
Thermal correction to Energy= 0.500276
Thermal correction to Enthalpy= 0.501220
Thermal correction to Gibbs Free Energy= 0.395204
E(solv) = -4465.24916867 A.U.

C	2.967148	1.378882	0.738967
C	2.624149	2.242380	1.941792
C	3.570294	2.214209	2.941227
H	4.461125	1.599163	2.869121
H	3.457812	2.848590	3.816077
C	3.506088	0.059158	0.851785
H	3.609519	-0.328361	1.862023
C	4.347556	-0.604638	-0.175091
C	4.385654	-2.008380	-0.259508
C	5.163124	0.137118	-1.046419
C	5.195580	-2.642690	-1.197263
H	3.732115	-2.584101	0.388890
C	5.970695	-0.500529	-1.988543
H	5.171876	1.219568	-0.965591

C	5.990167	-1.893436	-2.070518
H	5.198686	-3.728535	-1.254624
H	6.594329	0.094161	-2.652124
H	6.620551	-2.391235	-2.803379
O	1.552522	2.936746	1.864404
Pd	1.410253	-0.097640	0.229275
C	2.374411	1.699819	-0.511283
H	1.794151	2.613577	-0.552610
H	2.782656	1.324467	-1.445611
P	-0.756510	0.077948	-0.433409
O	-1.703441	0.066406	0.902544
O	-1.443608	1.268573	-1.366052
O	-1.254030	-1.156229	-1.389010
C	-2.429905	-1.881241	-1.265348
C	-3.616895	-1.386883	-1.803265
C	-2.373190	-3.117447	-0.625020
C	-4.780718	-2.144090	-1.675241
H	-3.617083	-0.412962	-2.279576
C	-3.543895	-3.867764	-0.514146
H	-1.426716	-3.434520	-0.196869
C	-4.747735	-3.384044	-1.032927
H	-5.716480	-1.757990	-2.069689
H	-3.515749	-4.829234	-0.008183
H	-5.658517	-3.968340	-0.932556
C	-3.086275	0.105465	1.023204
C	-3.700129	-0.992777	1.624849
C	-3.826997	1.217246	0.624080
C	-5.083268	-0.999072	1.782026
H	-3.084681	-1.831161	1.928050
C	-5.213284	1.196680	0.789240
H	-3.330604	2.080834	0.201459
C	-5.847298	0.091207	1.357224
H	-5.564661	-1.863662	2.230397
H	-5.796156	2.057852	0.473127
H	-6.927382	0.081523	1.476258
C	-1.312250	2.634897	-1.149960
C	-0.648546	3.182785	-0.053388
C	-1.914321	3.450427	-2.112853
C	-0.557643	4.573400	0.047521
H	-0.123603	2.600299	0.703069
C	-1.837026	4.836025	-1.981187
H	-2.428106	2.985224	-2.948476
C	-1.153135	5.404174	-0.901742
H	0.012581	4.969895	0.881320

H	-2.305316	5.470181	-2.730090
H	-1.081626	6.484767	-0.809428
Br	0.967760	-2.425916	1.157975

PB-INT1-D1

Zero-point correction= 0.466194 (Hartree/Particle)
Thermal correction to Energy= 0.500324
Thermal correction to Enthalpy= 0.501269
Thermal correction to Gibbs Free Energy= 0.395281
E(solv) = -4465.25642461 A.U.

C	-2.504833	-2.028079	-0.911675
C	-2.002329	-3.447369	-1.153089
C	-2.636710	-4.121429	-2.169918
H	-3.421491	-3.663572	-2.761851
H	-2.390787	-5.161090	-2.366015
C	-2.195051	-1.465380	0.367913
H	-1.618271	-2.150676	0.985799
C	-2.924003	-0.404618	1.094582
C	-2.300540	0.211949	2.196777
C	-4.237276	-0.017538	0.779139
C	-2.952777	1.192125	2.934956
H	-1.284287	-0.075292	2.450724
C	-4.888174	0.972337	1.515715
H	-4.756316	-0.515797	-0.032308
C	-4.249772	1.585518	2.593648
H	-2.440101	1.666237	3.766018
H	-5.904627	1.254791	1.251332
H	-4.756296	2.359041	3.165247
O	-1.107440	-3.873958	-0.343447
Pd	-0.967860	-0.449023	-1.120098
C	-2.862696	-1.132357	-1.948828
H	-2.834088	-1.480312	-2.975064
H	-3.453197	-0.238587	-1.765015
P	0.892956	0.094678	0.056665
O	2.276999	-0.249085	-0.731231
O	1.142962	1.689074	0.381644
O	1.202522	-0.444427	1.598916
C	1.689731	-1.722596	1.874737
C	2.703802	-1.792942	2.832543
C	1.187862	-2.868291	1.260017
C	3.225451	-3.037681	3.187029
H	3.063759	-0.874027	3.285211
C	1.725357	-4.104629	1.626173
H	0.376100	-2.865763	0.528106

C	2.738917	-4.199149	2.581501
H	4.012930	-3.095488	3.934596
H	1.318184	-4.990329	1.148062
H	3.145879	-5.168766	2.856806
C	3.428188	0.529026	-0.712420
C	4.452771	0.219379	0.177634
C	3.542696	1.566435	-1.636096
C	5.625988	0.976035	0.139913
H	4.321290	-0.600795	0.875693
C	4.719439	2.313451	-1.659835
H	2.710245	1.759067	-2.306454
C	5.761897	2.022496	-0.774436
H	6.434583	0.743954	0.828256
H	4.821918	3.126277	-2.373875
H	6.676689	2.608813	-0.798705
C	0.129051	2.504537	0.872967
C	-0.943908	2.878099	0.063488
C	0.256856	2.979649	2.177058
C	-1.910330	3.736239	0.588567
H	-1.005574	2.498673	-0.951295
C	-0.707223	3.853951	2.679866
H	1.106918	2.660217	2.770528
C	-1.795150	4.229534	1.889401
H	-2.759598	4.016263	-0.027870
H	-0.611650	4.231870	3.694617
H	-2.554294	4.896620	2.288433
Br	-0.218699	0.935673	-3.109927

PB-INT1-D2

Zero-point correction= 0.466221 (Hartree/Particle)
Thermal correction to Energy= 0.500321
Thermal correction to Enthalpy= 0.501265
Thermal correction to Gibbs Free Energy= 0.394800
E(solv) = -4465.25239709 A.U.

C	-2.166035	-1.985075	-0.350462
C	-1.634075	-3.058623	0.605452
C	-0.874020	-4.061925	0.025072
H	-0.767225	-4.161138	-1.049472
H	-0.538792	-4.891776	0.641318
C	-3.064886	-1.015328	0.183490
H	-3.203631	-1.112115	1.259036
C	-4.097678	-0.279590	-0.575924
C	-4.567132	0.961577	-0.105888
C	-4.680980	-0.811659	-1.739789

C	-5.562848	1.651786	-0.790221
H	-4.098957	1.391865	0.773640
C	-5.677467	-0.116509	-2.425193
H	-4.363807	-1.788916	-2.090051
C	-6.122175	1.120297	-1.956639
H	-5.898694	2.615881	-0.416096
H	-6.115031	-0.550298	-3.321485
H	-6.899938	1.661966	-2.489409
O	-1.948579	-2.918123	1.826736
Pd	-1.107327	-0.050772	-0.221901
C	2.107863	0.547093	2.826254
C	1.704567	-0.288449	1.789032
C	0.463844	-1.669986	3.308790
C	0.860901	-0.848888	4.363965
C	1.689049	0.252081	4.122658
H	2.712111	1.419518	2.600027
H	-0.225632	-2.496520	3.440653
H	0.512715	-1.053500	5.373090
H	1.990153	0.900465	4.941789
C	2.315102	2.508983	-1.178906
C	2.179614	4.400102	0.296069
C	3.361009	3.119186	-1.874864
C	3.225607	5.023095	-0.386924
H	1.706158	4.891619	1.141526
C	3.811966	4.376974	-1.477479
H	3.804309	2.598073	-2.717611
H	3.578906	6.002328	-0.075448
H	4.626360	4.848913	-2.021449
O	2.133843	0.051011	0.499276
O	1.922353	1.269413	-1.653153
P	1.098552	0.124312	-0.769495
C	-1.558278	-1.655859	-1.590886
H	-0.717728	-2.240196	-1.942720
H	-2.107738	-1.109011	-2.354573
C	0.905470	-1.403694	2.011114
H	0.604054	-2.056822	1.198951
C	1.712781	3.142908	-0.090421
H	0.880552	2.689395	0.442251
C	2.727197	-1.876227	-1.490595
C	3.946559	-1.363124	-1.927983
C	2.631582	-3.126504	-0.882576
C	5.100429	-2.127669	-1.750115
H	3.976270	-0.381312	-2.386874
C	3.794201	-3.879240	-0.714887

H	1.656517	-3.480041	-0.547369
C	5.028021	-3.385044	-1.146026
H	6.058406	-1.736301	-2.082694
H	3.732314	-4.853811	-0.238224
H	5.930233	-3.975235	-1.008672
O	1.545913	-1.165348	-1.685284
Br	-1.341785	1.843944	1.437783

PB-INT1-A1'

Zero-point correction= 0.467619 (Hartree/Particle)
Thermal correction to Energy= 0.501285
Thermal correction to Enthalpy= 0.502229
Thermal correction to Gibbs Free Energy= 0.397428
E(solv) = -4465.26824855 A.U.

C	-2.157255	-0.409651	-1.813146
C	-2.500106	-1.863374	-1.773472
C	-3.525045	-2.406409	-2.471633
H	-4.223635	-1.774442	-3.008134
H	-3.689531	-3.479669	-2.468368
C	-2.938217	0.673521	-1.564025
H	-2.430931	1.635837	-1.646890
C	-4.317131	0.799564	-1.098084
C	-4.910696	2.078376	-1.130579
C	-5.064599	-0.243244	-0.511569
C	-6.180215	2.313297	-0.607704
H	-4.344888	2.902691	-1.559433
C	-6.328788	-0.006267	0.018291
H	-4.636648	-1.237044	-0.472061
C	-6.901036	1.270304	-0.023014
H	-6.603636	3.314723	-0.649504
H	-6.873757	-0.828878	0.476229
H	-7.890135	1.446586	0.392612
O	-1.625925	-2.557649	-1.047525
Pd	-0.059478	-1.378099	-0.372581
C	3.676282	-2.040134	-0.091959
C	3.771290	-0.686622	0.232162
C	5.855685	-0.499875	-0.952297
C	5.774764	-1.850612	-1.298189
C	4.687289	-2.613139	-0.865272
H	2.830711	-2.616176	0.272827
H	6.699491	0.100621	-1.282267
H	6.556737	-2.306978	-1.899493
H	4.620012	-3.666018	-1.125167
C	-0.636430	1.167318	1.838414

C	-2.463215	2.697426	1.533957
C	-1.510636	0.157625	2.235824
C	-3.357680	1.698827	1.920879
H	-2.833666	3.676721	1.243292
C	-2.877761	0.434866	2.268515
H	-1.118490	-0.827354	2.467020
H	-4.425666	1.891073	1.911377
H	-3.573204	-0.354534	2.536254
O	2.799916	-0.080341	1.029731
O	0.742890	0.909446	1.777818
P	1.291273	0.155784	0.418490
C	-0.695050	-0.255270	-1.997058
H	-0.286512	-0.844976	-2.825639
H	-0.350974	0.778253	-2.039443
C	4.850122	0.091947	-0.186507
H	4.888051	1.140161	0.087722
C	-1.092445	2.434118	1.483363
H	-0.379217	3.187536	1.163656
C	2.437772	2.476210	-0.464913
C	2.995584	2.987576	-1.639841
C	2.761448	3.034199	0.776303
C	3.884989	4.057630	-1.575534
H	2.727473	2.524490	-2.583667
C	3.659756	4.102096	0.824484
H	2.327417	2.628968	1.680576
C	4.225467	4.618982	-0.342070
H	4.317900	4.448488	-2.492596
H	3.916579	4.530577	1.789692
H	4.923375	5.450014	-0.291524
O	1.561221	1.424385	-0.626218
Br	0.532868	-2.958495	1.593197

PB-INT1-A2'

Zero-point correction= 0.466901 (Hartree/Particle)
Thermal correction to Energy= 0.500854
Thermal correction to Enthalpy= 0.501798
Thermal correction to Gibbs Free Energy= 0.394840
E(solv) = -4465.25936280 A.U.

C	3.229984	-0.986565	-1.289773
C	2.776578	0.025885	-2.294024
C	3.578568	0.589711	-3.227272
H	4.647992	0.410254	-3.220281
H	3.164247	1.234847	-3.996455
C	4.068482	-0.828831	-0.229124

H	4.129176	-1.696821	0.428857
C	4.793210	0.325407	0.291658
C	5.346009	0.209644	1.587079
C	4.946876	1.569669	-0.359237
C	5.992600	1.272656	2.210196
H	5.232965	-0.733418	2.116583
C	5.589734	2.633569	0.268736
H	4.532737	1.697054	-1.351573
C	6.117715	2.500909	1.556408
H	6.394698	1.143754	3.212896
H	5.679330	3.581417	-0.257961
H	6.620127	3.335935	2.038301
O	1.460479	0.215880	-2.222352
Pd	0.535301	-1.339521	-1.172895
C	-0.181559	0.447684	2.879984
C	-0.189936	0.787445	1.528224
C	2.031178	1.686204	1.707009
C	2.056796	1.353810	3.061757
C	0.947977	0.735599	3.645494
H	-1.052086	-0.046188	3.298975
H	2.896332	2.146232	1.244328
H	2.946349	1.561596	3.648862
H	0.962863	0.464548	4.698215
C	-3.712242	-0.774592	0.444515
C	-4.299769	-1.936806	2.460301
C	-4.778873	0.105847	0.614780
C	-5.378601	-1.068026	2.647854
H	-4.107152	-2.730388	3.177649
C	-5.614788	-0.050032	1.721935
H	-4.935418	0.899373	-0.107133
H	-6.028613	-1.182076	3.511581
H	-6.448015	0.634116	1.861899
O	-1.357400	0.514105	0.828935
O	-2.916553	-0.643752	-0.688944
P	-1.359684	-0.093538	-0.710381
C	2.400850	-2.202907	-1.424950
H	2.324994	-2.582115	-2.450303
H	2.612186	-2.998089	-0.709171
C	0.906395	1.408981	0.927319
H	0.905714	1.628688	-0.135492
C	-3.459673	-1.799243	1.355478
H	-2.609565	-2.455396	1.186446
C	-2.585383	2.178539	-1.388815
C	-3.696374	2.167505	-2.233641

C	-2.495761	3.092133	-0.336623
C	-4.731140	3.078364	-2.019177
H	-3.734820	1.438636	-3.035834
C	-3.539597	3.993610	-0.127847
H	-1.621652	3.082096	0.303508
C	-4.659504	3.989820	-0.962623
H	-5.597662	3.069529	-2.675174
H	-3.475807	4.700702	0.694815
H	-5.469394	4.694091	-0.793087
O	-1.562579	1.278813	-1.627049
Br	-0.284233	-3.320961	0.099999

PB-INT1-B1'

Zero-point correction= 0.467529 (Hartree/Particle)
Thermal correction to Energy= 0.501167
Thermal correction to Enthalpy= 0.502111
Thermal correction to Gibbs Free Energy= 0.396183
E(solv) = -4465.27340629 A.U.

C	-2.624518	-0.353033	-1.342251
C	-2.796193	-1.800894	-0.928471
C	-1.815470	-2.690770	-1.585274
H	-1.856681	-2.679634	-2.679930
H	-1.817716	-3.701958	-1.181070
C	-3.348573	0.745401	-1.000372
H	-2.929289	1.669927	-1.402271
C	-4.546519	1.017844	-0.205494
C	-4.928641	2.372813	-0.089511
C	-5.341469	0.065562	0.465448
C	-6.031838	2.766108	0.662264
H	-4.329900	3.127793	-0.594477
C	-6.448277	0.461451	1.212685
H	-5.044047	-0.973714	0.415434
C	-6.803832	1.808734	1.323628
H	-6.287753	3.821149	0.733478
H	-7.037366	-0.296612	1.724675
H	-7.665380	2.107410	1.916129
O	-3.554856	-2.185566	-0.032987
Pd	-0.056690	-1.584004	-1.203398
C	4.346656	0.825128	1.388618
C	3.648749	-0.280077	0.902905
C	4.156992	-1.519800	2.892426
C	4.867968	-0.427297	3.394391
C	4.960837	0.743774	2.638567
H	4.390672	1.728650	0.790308

H	4.074624	-2.432537	3.476644
H	5.342132	-0.485251	4.370782
H	5.505346	1.602016	3.024481
C	-0.132161	0.845869	1.282068
C	-2.024553	2.112039	2.059904
C	-0.733947	-0.344350	1.690790
C	-2.655940	0.928607	2.443334
H	-2.530344	3.064596	2.190829
C	-2.010706	-0.293805	2.252894
H	-0.227954	-1.292580	1.543181
H	-3.660682	0.956686	2.851847
H	-2.509960	-1.222379	2.507364
O	3.093735	-0.202527	-0.371189
O	1.135372	0.853414	0.700761
P	1.479460	-0.002971	-0.675601
C	-1.342629	-0.189642	-2.097646
H	-1.392948	-0.550909	-3.133352
H	-0.984239	0.842065	-2.102145
C	3.543423	-1.457258	1.640317
H	2.977273	-2.290584	1.230014
C	-0.761528	2.075251	1.467475
H	-0.252549	2.980085	1.152325
C	2.273022	2.397868	-1.582726
C	1.535599	3.514393	-1.186537
C	3.649720	2.488917	-1.794865
C	2.185681	4.733037	-0.994065
H	0.466726	3.408604	-1.037315
C	4.290563	3.713300	-1.597821
H	4.194927	1.602663	-2.097241
C	3.564280	4.836447	-1.195048
H	1.612674	5.602594	-0.683365
H	5.362778	3.786883	-1.759709
H	4.068690	5.786417	-1.041349
O	1.610594	1.206748	-1.812303
Br	1.160225	-3.577225	-0.151570

PB-INT1-B2'

Zero-point correction= 0.467179 (Hartree/Particle)
Thermal correction to Energy= 0.500971
Thermal correction to Enthalpy= 0.501915
Thermal correction to Gibbs Free Energy= 0.395111
E(solv) = -4465.27129930 A.U.

C	-2.660920	-1.248468	-1.015998
C	-1.993825	-1.902528	0.167669

C	-0.547431	-2.186369	-0.103179
H	-0.423519	-3.098619	-0.701317
H	-0.007236	-2.304409	0.837456
C	-3.751339	-0.428772	-1.025015
H	-3.930404	0.031321	-1.998308
C	-4.665393	0.085803	-0.007282
C	-5.377474	1.263271	-0.330493
C	-4.916233	-0.489744	1.256275
C	-6.259525	1.859462	0.565886
H	-5.208416	1.723129	-1.301620
C	-5.807325	0.103242	2.147165
H	-4.372166	-1.383230	1.528990
C	-6.480898	1.283485	1.819708
H	-6.775534	2.775265	0.285338
H	-5.973378	-0.362588	3.116521
H	-7.169614	1.742934	2.524815
O	-2.517379	-2.125044	1.257644
Pd	0.030962	-0.690044	-1.468863
C	-1.803179	-1.371721	-2.223588
H	-1.606338	-2.406838	-2.529395
H	-2.130554	-0.771820	-3.073033
P	1.778141	0.038721	-0.217821
O	2.690757	1.330769	-0.671196
O	1.278693	0.502553	1.302704
O	3.051559	-0.915898	0.286591
C	2.790073	-2.198392	0.740500
C	2.730068	-3.252085	-0.170801
C	2.605714	-2.414154	2.106068
C	2.478047	-4.541576	0.296825
H	2.851181	-3.042150	-1.228140
C	2.356991	-3.708741	2.562529
H	2.641492	-1.565005	2.779312
C	2.290679	-4.773770	1.661293
H	2.415941	-5.364432	-0.410037
H	2.202700	-3.882556	3.624019
H	2.085981	-5.778684	2.019782
C	3.531131	2.009433	0.196715
C	4.881144	1.664519	0.254689
C	3.025102	3.062532	0.959065
C	5.735510	2.390521	1.084849
H	5.236446	0.834071	-0.344598
C	3.888322	3.779528	1.787212
H	1.968870	3.300299	0.899132
C	5.243870	3.448528	1.852828

H	6.788586	2.125415	1.132230
H	3.497090	4.598946	2.384332
H	5.912490	4.010899	2.499088
C	-0.017470	0.978545	1.483686
C	-0.599602	1.881405	0.589838
C	-0.716905	0.516413	2.595755
C	-1.907984	2.309831	0.820692
H	-0.065821	2.205480	-0.297818
C	-2.017840	0.965074	2.818269
H	-0.247252	-0.213847	3.246117
C	-2.618711	1.862144	1.933815
H	-2.379671	2.976095	0.104811
H	-2.579285	0.579416	3.663983
H	-3.646627	2.172967	2.086451
Br	0.294345	1.035303	-3.324825

PB-INT1-C1′

Zero-point correction= 0.466076 (Hartree/Particle)
Thermal correction to Energy= 0.500273
Thermal correction to Enthalpy= 0.501217
Thermal correction to Gibbs Free Energy= 0.395575
E(solv) = -4465.24984875 A.U.

C	3.245758	0.460749	-1.072980
C	3.187914	0.581012	-2.590598
C	4.355002	0.985765	-3.178749
H	5.249216	1.161090	-2.587077
H	4.411257	1.128667	-4.254780
C	2.398332	1.185276	-0.183849
H	2.672064	1.114244	0.871110
C	1.600363	2.390006	-0.469045
C	1.421634	3.309264	0.582753
C	1.030704	2.681611	-1.723861
C	0.729094	4.503808	0.387352
H	1.835051	3.078557	1.561124
C	0.335449	3.875222	-1.908154
H	1.138934	1.945156	-2.519674
C	0.186784	4.794878	-0.866138
H	0.611277	5.201459	1.213505
H	-0.104572	4.085448	-2.879743
H	-0.361149	5.720677	-1.024585
O	2.048551	0.292708	-3.110020
Pd	1.715993	-0.932204	-0.286205
C	3.866113	-0.666761	-0.480596
H	4.374527	-1.382759	-1.116568

H	4.152190	-0.665210	0.571569
P	-0.519356	-0.732807	-0.034125
O	-1.378796	-2.127588	-0.177504
O	-1.056603	-0.209747	1.459299
O	-1.370401	0.271196	-1.018667
C	-2.365300	1.178663	-0.680952
C	-2.032657	2.348483	-0.002669
C	-3.671969	0.936161	-1.100711
C	-3.030395	3.281090	0.276161
H	-1.004831	2.526897	0.280441
C	-4.661824	1.877916	-0.816658
H	-3.901482	0.020994	-1.632485
C	-4.348440	3.049546	-0.124327
H	-2.763637	4.198004	0.794603
H	-5.682552	1.687788	-1.137852
H	-5.123448	3.779595	0.093837
C	-2.753363	-2.198495	-0.303579
C	-3.271319	-2.635007	-1.523501
C	-3.595936	-1.876404	0.761679
C	-4.653548	-2.736202	-1.682006
H	-2.582135	-2.875241	-2.325945
C	-4.976290	-1.975189	0.587695
H	-3.164686	-1.531389	1.693275
C	-5.510498	-2.401062	-0.630165
H	-5.060228	-3.070645	-2.632591
H	-5.636451	-1.710916	1.409288
H	-6.586932	-2.473474	-0.758879
C	-0.165893	0.200942	2.441771
C	0.877460	-0.625311	2.867481
C	-0.368829	1.458144	3.009219
C	1.745848	-0.156123	3.854901
H	1.021963	-1.604004	2.420482
C	0.498121	1.905985	4.006471
H	-1.190201	2.068605	2.651951
C	1.564446	1.106192	4.424535
H	2.568638	-0.789340	4.174656
H	0.345000	2.888176	4.445906
H	2.245888	1.462257	5.192343
Br	1.875096	-3.398418	0.250848

PB-INT1-C2′

Zero-point correction= 0.465483 (Hartree/Particle)
Thermal correction to Energy= 0.499868
Thermal correction to Enthalpy= 0.500813

Thermal correction to Gibbs Free Energy= 0.392811
E(solv) = -4465.24791288 A.U.

C	2.891084	1.338254	-1.252279
C	3.141503	2.362255	-0.149867
C	4.181156	3.217823	-0.362357
H	4.800279	3.138643	-1.251582
H	4.428684	3.978690	0.373115
C	3.481636	0.048064	-1.284424
H	3.408594	-0.503131	-2.225002
C	4.505939	-0.462386	-0.361126
C	5.423091	-1.414801	-0.830721
C	4.581758	-0.057331	0.985174
C	6.411876	-1.935065	0.003240
H	5.355754	-1.747663	-1.864316
C	5.569257	-0.580556	1.813880
H	3.845378	0.639494	1.372399
C	6.491095	-1.514765	1.330916
H	7.113564	-2.670994	-0.382600
H	5.612954	-0.262961	2.852739
H	7.256040	-1.920784	1.988946
O	2.325729	2.276497	0.846301
Pd	1.308879	-0.135914	-0.765074
C	1.746204	1.501606	-2.077271
H	1.170798	2.418426	-1.987188
H	1.681993	0.997482	-3.043008
P	-0.898060	0.156046	-0.503429
O	-1.428045	-0.034938	1.037686
O	-1.602831	1.611650	-0.873833
O	-1.826865	-0.816194	-1.448455
C	-3.213490	-0.876458	-1.534391
C	-3.969871	0.205859	-1.986586
C	-3.818447	-2.092334	-1.217437
C	-5.357479	0.072818	-2.069374
H	-3.473762	1.129246	-2.252211
C	-5.202919	-2.210770	-1.305456
H	-3.198617	-2.910109	-0.871258
C	-5.979768	-1.127371	-1.723558
H	-5.951534	0.916470	-2.411281
H	-5.674757	-3.150964	-1.034428
H	-7.060669	-1.220225	-1.784025
C	-2.449602	-0.856335	1.484762
C	-2.180785	-2.203021	1.723298
C	-3.706916	-0.303025	1.720350
C	-3.215597	-3.015777	2.187303

H	-1.180526	-2.578107	1.520167
C	-4.730311	-1.129967	2.181812
H	-3.864375	0.752754	1.526408
C	-4.489896	-2.486976	2.411123
H	-3.022891	-4.069521	2.371269
H	-5.719020	-0.713419	2.354177
H	-5.291805	-3.128788	2.766505
C	-1.677250	2.672940	0.030263
C	-0.547925	3.150698	0.692317
C	-2.935252	3.249730	0.208603
C	-0.703676	4.241707	1.550021
H	0.445320	2.700026	0.578377
C	-3.066944	4.344913	1.064156
H	-3.788324	2.834854	-0.319556
C	-1.950072	4.843851	1.738199
H	0.175865	4.612028	2.068683
H	-4.043708	4.801329	1.205111
H	-2.053038	5.693808	2.408002
Br	1.347885	-2.400654	0.356241

PB-INT1-D1′

Zero-point correction= 0.465560 (Hartree/Particle)
Thermal correction to Energy= 0.499798
Thermal correction to Enthalpy= 0.500743
Thermal correction to Gibbs Free Energy= 0.393825
E(solv) = -4465.24911178 A.U.

C	-2.209043	-2.372547	-0.736838
C	-2.251878	-3.108412	0.606580
C	-1.068313	-3.218279	1.306358
H	-0.126959	-2.857017	0.907121
H	-1.046351	-3.780619	2.236178
C	-3.053221	-1.294668	-1.145399
H	-3.172938	-1.167228	-2.223507
C	-4.024879	-0.519198	-0.358569
C	-4.616974	0.592114	-0.988505
C	-4.367871	-0.790363	0.979857
C	-5.508930	1.416552	-0.310716
H	-4.337704	0.824418	-2.011891
C	-5.263013	0.040435	1.652647
H	-3.986130	-1.690040	1.448198
C	-5.833482	1.147335	1.020559
H	-5.939602	2.276501	-0.817866
H	-5.526365	-0.190961	2.682540
H	-6.527951	1.790364	1.556743

O	-3.403867	-3.567008	0.887646
Pd	-0.933321	-0.581522	-1.107889
C	1.020078	2.000274	2.049800
C	0.650924	0.703720	1.705136
C	-1.459370	0.952916	2.809375
C	-1.120374	2.262074	3.152992
C	0.124214	2.777754	2.784164
H	1.978688	2.389732	1.727866
H	-2.431023	0.545727	3.071821
H	-1.825115	2.881393	3.701643
H	0.395847	3.796442	3.049066
C	2.893322	1.632907	-0.912931
C	2.704879	4.011605	-0.651991
C	4.107064	1.632711	-0.225951
C	3.922151	4.034162	0.033194
H	2.145264	4.930619	-0.802520
C	4.620064	2.842268	0.244897
H	4.621189	0.692671	-0.058239
H	4.320496	4.972941	0.409002
H	5.562444	2.849215	0.786530
O	1.563753	-0.056542	0.952272
O	2.401027	0.428192	-1.403750
P	1.264964	-0.489598	-0.605651
C	-1.164607	-2.649140	-1.656149
H	-0.405769	-3.370548	-1.374018
H	-1.307107	-2.501130	-2.729160
C	-0.567254	0.153572	2.093202
H	-0.822991	-0.874837	1.839263
C	2.181456	2.810545	-1.131969
H	1.207769	2.761695	-1.609844
C	3.310700	-2.122790	-0.100165
C	4.440594	-1.972802	-0.903496
C	3.419838	-2.488129	1.241308
C	5.703135	-2.193087	-0.349502
H	4.315411	-1.676278	-1.938672
C	4.686398	-2.705401	1.782736
H	2.518054	-2.589062	1.834313
C	5.829669	-2.557054	0.992851
H	6.587855	-2.076529	-0.969822
H	4.778735	-2.987991	2.827767
H	6.813830	-2.726018	1.421234
O	2.050474	-1.940181	-0.654270
Br	-1.352972	1.909724	-1.230633

PB-INT1-D2′

Zero-point correction= 0.465972 (Hartree/Particle)
Thermal correction to Energy= 0.500041
Thermal correction to Enthalpy= 0.500986
Thermal correction to Gibbs Free Energy= 0.397009
E(solv) = -4465.25233194 A.U.

C	-3.307881	-0.771575	-0.949530
C	-4.208900	-0.290855	0.193664
C	-4.170855	-1.020604	1.363785
H	-3.546753	-1.901663	1.469818
H	-4.823795	-0.747049	2.189156
C	-2.416782	0.086152	-1.669240
H	-2.198484	-0.200101	-2.700882
C	-2.253370	1.529442	-1.414315
C	-2.039501	2.390086	-2.501658
C	-2.325404	2.086333	-0.124794
C	-1.938204	3.771690	-2.321364
H	-1.972065	1.969404	-3.502690
C	-2.198750	3.458729	0.057737
H	-2.477170	1.437695	0.727057
C	-2.020369	4.311595	-1.037218
H	-1.786356	4.419617	-3.181261
H	-2.245829	3.862659	1.064667
H	-1.938386	5.385858	-0.888422
O	-4.893918	0.728593	-0.122652
Pd	-1.213156	-1.485756	-0.664332
C	0.661132	1.812097	2.376222
C	0.359505	0.501599	2.005874
C	-1.726665	0.598541	3.190194
C	-1.443954	1.910490	3.572652
C	-0.246800	2.510850	3.170640
H	1.581098	2.268984	2.032665
H	-2.675001	0.133781	3.435655
H	-2.160338	2.468954	4.169012
H	-0.023886	3.535600	3.456554
C	1.963104	1.810002	-0.739925
C	1.620259	4.168595	-0.416696
C	3.226690	1.893145	-0.153072
C	2.877330	4.271124	0.183453
H	0.978827	5.039393	-0.515022
C	3.676408	3.131805	0.308460
H	3.836048	1.006387	-0.044818
H	3.231890	5.229638	0.553164
H	4.656174	3.199516	0.774175

O	1.285944	-0.186140	1.206882
O	1.469656	0.606102	-1.230128
P	0.874679	-0.654000	-0.322366
C	-3.189197	-2.135056	-1.287624
H	-3.697832	-2.875423	-0.680859
H	-2.908341	-2.446982	-2.295366
C	-0.815835	-0.122453	2.414994
H	-1.034127	-1.137406	2.102165
C	1.160293	2.938176	-0.881642
H	0.184805	2.835487	-1.334978
C	3.393346	-1.538069	-0.429591
C	4.166136	-1.247233	-1.553762
C	3.967606	-1.618838	0.839748
C	5.534796	-1.023785	-1.401546
H	3.681164	-1.183628	-2.521472
C	5.336650	-1.389990	0.979207
H	3.333944	-1.841493	1.689645
C	6.123006	-1.089355	-0.136342
H	6.139481	-0.788794	-2.273218
H	5.788854	-1.444139	1.965775
H	7.188048	-0.908728	-0.020080
O	2.041404	-1.776639	-0.592056
Br	-0.635269	-3.706684	0.394231

INT1-B1-TMA1

Zero-point correction= 0.636558 (Hartree/Particle)
Thermal correction to Energy= 0.677718
Thermal correction to Enthalpy= 0.678662
Thermal correction to Gibbs Free Energy= 0.559200
E(solv) = -4679.65815787 A.U.

C	-1.014620	1.033591	-2.567123
C	-2.333343	0.291666	-2.573952
C	-2.174893	-1.161585	-2.474214
H	-1.581992	-1.615533	-3.272884
H	-3.099688	-1.709389	-2.297362
C	-1.040218	2.384232	-2.473429
H	-2.031793	2.834792	-2.465201
C	0.081558	3.302477	-2.281015
C	-0.143751	4.514707	-1.596509
C	1.386406	3.059506	-2.755852
C	0.890124	5.413649	-1.345720
H	-1.151252	4.739024	-1.253783
C	2.419385	3.964270	-2.515096
H	1.581931	2.169570	-3.342512

C	2.183751	5.138286	-1.795348
H	0.685960	6.334031	-0.804801
H	3.413899	3.753515	-2.899837
H	2.992180	5.838466	-1.605686
O	-3.416299	0.894993	-2.465733
Pd	-0.682885	-1.169960	-0.930140
C	1.273352	-4.268922	-0.159245
C	2.179665	-3.266371	0.177221
C	3.839660	-4.199829	-1.285010
C	2.947798	-5.215843	-1.635623
C	1.669430	-5.246842	-1.073140
H	0.279739	-4.255084	0.275297
H	4.834196	-4.170642	-1.720734
H	3.248211	-5.980638	-2.345895
H	0.970891	-6.032005	-1.347213
C	0.078365	0.837258	2.246895
C	-0.499334	3.118463	1.730082
C	-0.673706	0.905600	3.421991
C	-1.237294	3.207209	2.911870
H	-0.417700	3.968885	1.061348
C	-1.325591	2.092974	3.753632
H	-0.721008	0.029385	4.059540
H	-1.732963	4.136021	3.179147
H	-1.892422	2.150056	4.679241
O	1.807922	-2.287990	1.112508
O	0.760215	-0.343171	2.030981
P	1.042506	-0.958178	0.527231
C	0.133613	0.117707	-2.354841
H	0.361035	-0.534584	-3.205267
H	1.042946	0.601997	-2.011124
C	3.457260	-3.212354	-0.375250
H	4.131028	-2.410778	-0.091341
C	0.159119	1.936161	1.385802
H	0.729501	1.886490	0.467221
C	3.265508	0.610812	0.725886
C	3.620466	1.894971	0.313988
C	3.888685	-0.000588	1.814315
C	4.620449	2.579043	1.002519
H	3.108498	2.341542	-0.531367
C	4.885843	0.700981	2.495310
H	3.593369	-0.994617	2.125169
C	5.256477	1.985741	2.095398
H	4.895373	3.580426	0.683556
H	5.373652	0.233515	3.345881

H	6.033610	2.521165	2.632645
O	2.272831	-0.012599	-0.026894
Br	-1.972616	-2.655277	0.769571
C	-3.025780	1.044678	0.598555
H	-2.745874	1.753105	1.375307
H	-2.315950	0.219228	0.557126
H	-3.093097	1.518236	-0.379137
C	-4.295170	-0.180090	2.291101
H	-5.280537	-0.571415	2.552047
H	-3.565308	-0.990104	2.215930
H	-3.968101	0.561754	3.021482
C	-5.372755	1.594189	0.967933
H	-5.412726	2.036161	-0.028642
H	-6.351275	1.196427	1.243367
H	-5.049910	2.338972	1.697758
C	-4.793620	-0.541414	-0.088072
H	-5.815607	-0.853266	0.138600
H	-4.710581	-0.080439	-1.074745
H	-4.104321	-1.384624	-0.011860
N	-4.379377	0.478571	0.945212

INT1-B1-TMA2

Zero-point correction= 0.634772 (Hartree/Particle)
Thermal correction to Energy= 0.676383
Thermal correction to Enthalpy= 0.677327
Thermal correction to Gibbs Free Energy= 0.557055
E(solv) = -4679.64810249 A.U.

C	1.248874	-2.487796	1.109236
C	0.375434	-3.453485	0.339419
C	-1.068148	-3.101649	0.444490
H	-1.434144	-3.161394	1.477213
H	-1.695957	-3.672181	-0.242808
C	2.590252	-2.618302	1.037634
H	2.954889	-3.437970	0.421024
C	3.598130	-1.728010	1.618025
C	4.806760	-1.507436	0.931024
C	3.424918	-1.089652	2.861916
C	5.784677	-0.659348	1.444805
H	4.955942	-1.991574	-0.028428
C	4.407866	-0.249383	3.382069
H	2.520476	-1.278419	3.430432
C	5.591134	-0.023236	2.673926
H	6.702398	-0.495513	0.885858
H	4.251976	0.227853	4.346062

H	6.356514	0.632256	3.080338
O	0.832093	-4.322696	-0.398262
Pd	-0.865046	-1.033152	0.024541
C	-3.341789	2.633293	-0.954183
C	-2.100295	2.651694	-0.315554
C	-3.118427	3.524021	1.688996
C	-4.363457	3.533131	1.054633
C	-4.468029	3.086849	-0.267894
H	-3.397417	2.256378	-1.968672
H	-3.024222	3.876190	2.712518
H	-5.241048	3.901216	1.578965
H	-5.430498	3.099808	-0.772815
C	1.808783	0.043419	-2.164855
C	4.124010	-0.501021	-2.477997
C	1.445717	-1.291850	-2.328952
C	3.783701	-1.849329	-2.613310
H	5.162938	-0.189481	-2.538723
C	2.446758	-2.242842	-2.532226
H	0.401646	-1.581360	-2.287188
H	4.559009	-2.592227	-2.778942
H	2.169736	-3.289107	-2.602163
O	-0.991612	2.287534	-1.051488
O	0.831095	1.029768	-1.946367
P	-0.044851	1.008525	-0.565827
C	0.468937	-1.324039	1.610208
H	-0.171570	-1.547070	2.473276
H	1.069826	-0.441274	1.820415
C	-1.979828	3.085107	1.008176
H	-1.003739	3.094465	1.480418
C	3.136789	0.456182	-2.242197
H	3.380900	1.504143	-2.114613
C	2.074766	2.489076	0.252861
C	3.297186	2.080651	0.779937
C	1.961113	3.639743	-0.525321
C	4.434502	2.842366	0.517693
H	3.349859	1.170236	1.363504
C	3.110880	4.388051	-0.786416
H	0.998500	3.925753	-0.932800
C	4.346998	3.993985	-0.268702
H	5.388398	2.517772	0.921672
H	3.036384	5.281026	-1.400503
H	5.236417	4.580889	-0.478909
O	0.968473	1.689704	0.545463
Br	-2.632129	-0.949310	-1.894149

C	-4.388446	-2.285518	1.004453
H	-3.730552	-2.228764	0.134566
H	-3.845338	-2.677658	1.864428
H	-5.266813	-2.898197	0.792659
C	-3.669933	-0.030096	1.684812
H	-4.017396	0.985675	1.871341
H	-3.192496	-0.451624	2.570110
H	-2.966619	-0.048964	0.849569
C	-5.539743	-0.306960	0.120961
H	-6.407223	-0.926637	-0.113629
H	-5.842591	0.713080	0.357092
H	-4.819249	-0.315293	-0.702244
C	-5.806507	-0.925412	2.482243
H	-6.123160	0.094058	2.708722
H	-6.670300	-1.535549	2.212733
H	-5.297985	-1.358880	3.344958
N	-4.855716	-0.889488	1.329149

INT1-B2-TMA1

Zero-point correction= 0.635379 (Hartree/Particle)
Thermal correction to Energy= 0.676999
Thermal correction to Enthalpy= 0.677944
Thermal correction to Gibbs Free Energy= 0.555712
E(solv) = -4679.65199497 A.U.

C	2.572071	1.747166	1.429629
C	1.702037	1.375376	2.616821
C	0.280838	1.757784	2.427106
H	0.118752	2.840675	2.391440
H	-0.377553	1.297142	3.165370
C	3.803153	1.176405	1.389041
H	4.053867	0.583261	2.268116
C	4.746545	1.077933	0.280524
C	5.627822	-0.027947	0.259097
C	4.815911	1.975664	-0.805566
C	6.489918	-0.258011	-0.811624
H	5.622268	-0.712625	1.104731
C	5.679280	1.746598	-1.874909
H	4.200659	2.866434	-0.800341
C	6.511523	0.623399	-1.896226
H	7.153840	-1.119015	-0.794673
H	5.706853	2.456445	-2.697289
H	7.181278	0.450491	-2.733654
O	2.106189	0.665778	3.538035
Pd	0.016160	1.278568	0.391304

C	-0.768779	-3.315264	1.345362
C	-0.712449	-2.120454	2.068535
C	1.367438	-2.794963	3.073069
C	1.323270	-3.999419	2.365321
C	0.253341	-4.255570	1.501407
H	-1.611290	-3.506102	0.690218
H	2.198010	-2.570709	3.734985
H	2.111395	-4.736623	2.490917
H	0.201102	-5.195839	0.958581
C	-3.211858	-1.718428	-0.971300
C	-4.635218	-2.345267	-2.806187
C	-4.073678	-2.332380	-0.061290
C	-5.509499	-2.967998	-1.911608
H	-4.852890	-2.343019	-3.870409
C	-5.226377	-2.954729	-0.545066
H	-3.853756	-2.309271	0.999039
H	-6.408545	-3.454992	-2.277170
H	-5.905907	-3.429846	0.156623
O	-1.754875	-1.204293	1.963563
O	-2.023804	-1.109824	-0.589954
P	-1.762692	-0.123095	0.701740
C	1.792772	2.411208	0.363177
H	1.455082	3.422904	0.613533
H	2.233881	2.401291	-0.630069
C	0.350192	-1.849439	2.929590
H	0.404614	-0.909040	3.463896
C	-3.483379	-1.714176	-2.339190
H	-2.792155	-1.205575	-3.003791
C	-3.667024	1.697311	0.623148
C	-4.388228	2.480175	1.521667
C	-3.393208	2.137190	-0.671386
C	-4.841414	3.734782	1.114771
H	-4.579647	2.099492	2.519397
C	-3.840430	3.402010	-1.058225
H	-2.821882	1.524974	-1.360925
C	-4.565375	4.200940	-0.173295
H	-5.404583	4.350621	1.809999
H	-3.614689	3.755959	-2.059593
H	-4.912975	5.181801	-0.483562
O	-3.256153	0.441802	1.074229
Br	-0.046679	1.160733	-2.182553
N	2.355760	-1.934735	-1.448763
C	2.206464	-1.379590	-0.059137
H	1.546363	-0.510245	-0.098654

H	1.778699	-2.151408	0.574882
H	3.187508	-1.080378	0.299026
C	3.256958	-3.126142	-1.392737
H	4.233456	-2.799870	-1.030614
H	2.824527	-3.856986	-0.707180
H	3.349586	-3.551436	-2.393765
C	2.957007	-0.884819	-2.346717
H	2.266205	-0.039326	-2.383081
H	3.921367	-0.586819	-1.936566
H	3.079669	-1.318944	-3.341276
C	0.998563	-2.330527	-1.963782
H	0.384413	-1.428925	-2.030970
H	1.122890	-2.785507	-2.948599
H	0.560124	-3.039799	-1.261043

INT1-D1-TMA1

Zero-point correction= 0.634614 (Hartree/Particle)
Thermal correction to Energy= 0.676378
Thermal correction to Enthalpy= 0.677322
Thermal correction to Gibbs Free Energy= 0.555723
E(solv) = -4679.63785337 A.U.

C	-0.747061	-2.973845	0.510526
C	0.539329	-3.557501	1.061981
C	0.726734	-4.894929	0.843221
H	-0.023430	-5.499464	0.346023
H	1.585368	-5.404121	1.272237
C	-1.369618	-1.894212	1.218979
H	-0.841713	-1.589044	2.118564
C	-2.806376	-1.533226	1.187761
C	-3.198281	-0.266367	1.657518
C	-3.804156	-2.421794	0.756212
C	-4.536727	0.108734	1.665202
H	-2.437753	0.429717	1.997616
C	-5.146073	-2.041721	0.759667
H	-3.529811	-3.423923	0.445146
C	-5.517872	-0.774196	1.207300
H	-4.813368	1.097763	2.015190
H	-5.902883	-2.745696	0.424221
H	-6.563237	-0.478670	1.208847
O	1.338786	-2.735113	1.665681
Pd	-0.453370	-1.078705	-0.571825
C	-1.108232	-3.166951	-0.839020
H	-0.495494	-3.810944	-1.459538
H	-2.106298	-2.953910	-1.210253

P	0.008690	1.055671	0.071704
O	1.540804	1.495112	-0.259749
O	-0.822441	2.239859	-0.685554
O	-0.213722	1.598857	1.620761
C	0.689306	1.366858	2.659286
C	0.951905	2.453583	3.494286
C	1.284157	0.124513	2.875748
C	1.828337	2.294271	4.568105
H	0.466469	3.402673	3.291353
C	2.163094	-0.014397	3.952757
H	1.084925	-0.744727	2.252905
C	2.440876	1.059988	4.798870
H	2.031245	3.137650	5.222165
H	2.615806	-0.986857	4.123273
H	3.122349	0.937718	5.635701
C	1.954185	2.778574	-0.632361
C	2.450819	3.644976	0.335657
C	1.912040	3.118737	-1.981818
C	2.918149	4.898010	-0.067218
H	2.466715	3.336425	1.375497
C	2.380861	4.374246	-2.367457
H	1.514182	2.401646	-2.693025
C	2.883079	5.264514	-1.414000
H	3.308988	5.587145	0.675994
H	2.351627	4.657204	-3.415696
H	3.245600	6.241286	-1.720650
C	-2.210112	2.142050	-0.848503
C	-2.750472	1.234083	-1.757172
C	-3.011843	3.007472	-0.109894
C	-4.137415	1.184846	-1.905919
H	-2.092795	0.589437	-2.330454
C	-4.395134	2.961038	-0.286933
H	-2.545506	3.699313	0.583132
C	-4.959955	2.045166	-1.176912
H	-4.571543	0.471262	-2.599713
H	-5.030681	3.635730	0.279658
H	-6.037814	2.001593	-1.301037
Br	0.436943	-0.682947	-2.923120
N	3.762144	-2.331526	-0.670695
C	2.816076	-3.196064	-1.466652
H	3.396700	-4.008591	-1.909319
H	2.062039	-3.607402	-0.792363
H	2.343245	-2.581077	-2.234661
C	3.060444	-1.061627	-0.259821

H	2.226637	-1.362978	0.378877
H	3.767588	-0.442973	0.295738
H	2.707523	-0.550224	-1.152732
C	4.945976	-1.992059	-1.514867
H	5.470947	-2.911394	-1.781328
H	4.595641	-1.484246	-2.415116
H	5.607668	-1.333884	-0.949276
C	4.179952	-3.071385	0.570870
H	4.602148	-4.034179	0.275534
H	4.933152	-2.472444	1.087709
H	3.279048	-3.188648	1.190716

INT1-D1-TMA2

Zero-point correction= 0.633619 (Hartree/Particle)
Thermal correction to Energy= 0.675680
Thermal correction to Enthalpy= 0.676625
Thermal correction to Gibbs Free Energy= 0.554598
E(solv) = -4679.61618325 A.U.

C	3.148423	1.926000	-0.410151
C	2.900179	3.343509	-0.907716
C	3.798899	3.794905	-1.845339
H	4.614984	3.178728	-2.204513
H	3.741462	4.818780	-2.200034
C	2.437221	1.574554	0.783223
H	1.830007	2.403620	1.143216
C	2.763463	0.534233	1.775912
C	1.789870	0.200857	2.740990
C	4.012889	-0.107295	1.857337
C	2.039567	-0.758147	3.718283
H	0.832542	0.713250	2.706603
C	4.262030	-1.071402	2.835795
H	4.803401	0.180983	1.173839
C	3.276620	-1.410739	3.766421
H	1.272959	-0.990416	4.453439
H	5.241834	-1.539655	2.887028
H	3.478769	-2.149540	4.536944
O	1.931305	3.967562	-0.354122
Pd	1.482785	0.569438	-0.913396
C	3.616704	0.865684	-1.227067
H	3.908657	1.076796	-2.249565
H	4.015975	-0.052667	-0.803333
P	-0.679398	0.496824	-0.210814
O	-1.748004	0.974488	-1.348859
O	-1.098845	-1.057881	0.158191

O	-1.226112	1.198275	1.167738
C	-1.625498	2.544264	1.247597
C	-2.819144	2.780955	1.926959
C	-0.857789	3.571691	0.708604
C	-3.262836	4.096474	2.070586
H	-3.372973	1.941197	2.335470
C	-1.323587	4.880682	0.860306
H	0.098942	3.413614	0.202143
C	-2.517132	5.148586	1.533080
H	-4.190049	4.294831	2.601029
H	-0.724449	5.685759	0.446547
H	-2.864327	6.171895	1.643303
C	-2.964438	0.348768	-1.607861
C	-4.107958	0.733810	-0.914357
C	-2.992256	-0.643595	-2.586389
C	-5.313478	0.091433	-1.203025
H	-4.042541	1.519028	-0.168882
C	-4.205645	-1.273507	-2.866323
H	-2.067757	-0.902376	-3.093930
C	-5.365157	-0.912716	-2.172359
H	-6.213426	0.378652	-0.667159
H	-4.244898	-2.046076	-3.629133
H	-6.306873	-1.406564	-2.393483
C	-2.021507	-1.526819	1.087080
C	-1.739562	-1.443800	2.451754
C	-3.163944	-2.177899	0.624407
C	-2.628149	-2.012024	3.365129
H	-0.845346	-0.928936	2.780788
C	-4.041593	-2.745440	1.549447
H	-3.361417	-2.218615	-0.439873
C	-3.780088	-2.663630	2.918606
H	-2.418612	-1.940427	4.428590
H	-4.937630	-3.244830	1.192612
H	-4.470333	-3.101367	3.633456
Br	1.042250	-0.975235	-2.890060
N	1.463552	-3.763018	-0.033255
C	2.582017	-3.709827	-1.039481
H	2.412905	-2.844441	-1.687346
H	3.525828	-3.618255	-0.499504
H	2.566113	-4.631544	-1.624103
C	1.600622	-4.981089	0.823507
H	2.560022	-4.941190	1.341787
H	0.785737	-4.991024	1.549124
H	1.550966	-5.869432	0.191573

C	0.142901	-3.797898	-0.759571
H	0.083273	-2.914283	-1.398737
H	0.110767	-4.710569	-1.357924
H	-0.659128	-3.792523	-0.021267
C	1.531776	-2.536901	0.838304
H	1.467489	-1.656403	0.198026
H	0.692421	-2.557696	1.530475
H	2.476674	-2.542052	1.380397

INT1-C1-TMA1

Zero-point correction= 0.634518 (Hartree/Particle)
Thermal correction to Energy= 0.676460
Thermal correction to Enthalpy= 0.677404
Thermal correction to Gibbs Free Energy= 0.554417
E(solv) = -4679.63518712 A.U.

C	-1.249301	-2.497279	1.467091
C	-0.035722	-2.874986	2.299511
C	0.042420	-2.347199	3.562317
H	-0.756348	-1.756511	3.994736
H	0.863392	-2.635661	4.211809
C	-1.895469	-1.225122	1.624649
H	-1.454329	-0.557803	2.357955
C	-3.296859	-0.903580	1.260743
C	-3.651406	0.436950	1.022957
C	-4.299034	-1.883730	1.178108
C	-4.953369	0.780461	0.674986
H	-2.889468	1.206478	1.092689
C	-5.604870	-1.538170	0.831940
H	-4.054676	-2.915726	1.406567
C	-5.935726	-0.207806	0.570284
H	-5.195626	1.818917	0.474042
H	-6.366980	-2.310657	0.776019
H	-6.952928	0.058812	0.297317
O	0.813354	-3.671643	1.737744
Pd	-0.770425	-1.163809	-0.219995
C	-1.508713	-3.190471	0.263172
H	-0.866874	-4.028698	0.020978
H	-2.468595	-3.138361	-0.237785
P	0.079456	0.917146	0.060015
O	1.710019	0.949040	0.052420
O	-0.218594	2.191915	-0.944397
O	-0.233195	1.528445	1.559572
C	0.668934	2.334371	2.253978
C	0.843861	3.671360	1.906455

C	1.375821	1.750609	3.303500
C	1.752440	4.442718	2.634157
H	0.294866	4.086372	1.069203
C	2.278973	2.533229	4.022831
H	1.210040	0.700542	3.529870
C	2.470846	3.877664	3.689777
H	1.899990	5.485731	2.369632
H	2.835560	2.090201	4.843635
H	3.175784	4.482250	4.252733
C	2.568021	1.661330	-0.784810
C	3.458900	2.547850	-0.185785
C	2.577585	1.416382	-2.156049
C	4.383697	3.214539	-0.991083
H	3.414022	2.707086	0.885686
C	3.504768	2.096960	-2.946233
H	1.877403	0.701902	-2.579341
C	4.407274	2.994305	-2.370053
H	5.083331	3.910440	-0.536811
H	3.519636	1.920572	-4.018013
H	5.125521	3.519577	-2.992772
C	-1.533762	2.365740	-1.397000
C	-2.056110	1.511104	-2.366082
C	-2.283856	3.409355	-0.859760
C	-3.368521	1.711736	-2.796743
H	-1.448029	0.701307	-2.756058
C	-3.589853	3.606405	-1.311659
H	-1.845113	4.050331	-0.102448
C	-4.135368	2.755650	-2.275683
H	-3.789007	1.043991	-3.542589
H	-4.180268	4.422084	-0.904108
H	-5.154919	2.905435	-2.618168
Br	0.211368	-1.545668	-2.540964
N	3.334981	-3.195677	-0.124737
C	4.511034	-3.038975	-1.028640
H	5.257837	-2.412772	-0.536735
H	4.930229	-4.023970	-1.242790
H	4.177510	-2.563641	-1.952597
C	2.284010	-4.050633	-0.792502
H	2.738578	-5.014681	-1.032224
H	1.467817	-4.145366	-0.070150
H	1.939742	-3.535121	-1.689965
C	2.745680	-1.840366	0.172710
H	3.515124	-1.222353	0.638195
H	2.405683	-1.401812	-0.762485

H	1.904238	-2.011902	0.843643
C	3.733810	-3.840869	1.174092
H	4.492281	-3.214692	1.649258
H	2.820844	-3.906299	1.780726
H	4.145274	-4.829099	0.957015

TS1

Zero-point correction= 0.851000 (Hartree/Particle)
Thermal correction to Energy= 0.907215
Thermal correction to Enthalpy= 0.908159
Thermal correction to Gibbs Free Energy= 0.753597
E(solv) = -5446.10480663 A.U.
Imaginary frequency = 459.66

C	-1.053649	1.070729	-1.576598
C	-2.143976	0.093354	-1.195901
C	-3.410727	0.180808	-1.847199
H	-3.477278	0.815766	-2.726147
H	-3.966873	-0.747240	-1.913842
C	-0.213846	1.601423	-0.544951
H	-0.470827	1.294050	0.463622
C	0.512438	2.886229	-0.631848
C	1.610941	3.117649	0.211563
C	0.065735	3.922127	-1.467052
C	2.265894	4.343942	0.198713
H	1.948052	2.329640	0.875878
C	0.725245	5.150981	-1.475780
H	-0.842946	3.788418	-2.041023
C	1.829695	5.364728	-0.649834
H	3.119530	4.501693	0.849897
H	0.358497	5.949737	-2.114345
H	2.338689	6.324643	-0.655751
O	-1.936734	-0.670899	-0.226944
Pd	0.862344	0.031319	-1.606095
C	-0.596383	1.102126	-2.902710
H	-1.111075	0.527243	-3.664825
H	0.072585	1.880568	-3.255513
P	2.283315	-0.678793	0.029707
O	2.223101	-2.283676	0.301703
O	3.873389	-0.456948	-0.290384
O	2.284536	-0.026000	1.543189
C	1.392026	-0.374932	2.561270
C	1.927725	-0.420702	3.846822
C	0.051289	-0.665588	2.321099

C	1.104120	-0.781494	4.911690
H	2.979530	-0.197159	3.988586
C	-0.764590	-1.025143	3.397650
H	-0.380309	-0.602142	1.329241
C	-0.240230	-1.092149	4.689370
H	1.517666	-0.823136	5.915400
H	-1.823615	-1.179853	3.217426
H	-0.882122	-1.363050	5.522544
C	2.992090	-2.972971	1.250405
C	2.352211	-3.451767	2.390994
C	4.340544	-3.219789	1.005978
C	3.089343	-4.193371	3.314529
H	1.306956	-3.218078	2.559445
C	5.064933	-3.961278	1.940621
H	4.800047	-2.832728	0.104636
C	4.444324	-4.448179	3.093184
H	2.601809	-4.562045	4.212104
H	6.118086	-4.158441	1.763343
H	5.015025	-5.022994	3.816452
C	4.291574	0.805772	-0.741027
C	4.061244	1.182777	-2.062498
C	4.948773	1.639292	0.159077
C	4.488359	2.445089	-2.479535
H	3.559822	0.497888	-2.738249
C	5.386764	2.889383	-0.279201
H	5.101001	1.303509	1.179000
C	5.148793	3.297545	-1.593473
H	4.304312	2.756975	-3.503232
H	5.904056	3.548471	0.412074
H	5.476998	4.277736	-1.925544
Br	1.645722	-1.669730	-3.311388
N	-1.571160	-3.843618	-1.299532
C	-1.714907	-3.086581	-2.593698
H	-2.518935	-3.545625	-3.172128
H	-1.960752	-2.055061	-2.357659
H	-0.764119	-3.112792	-3.128599
C	-0.360189	-3.331377	-0.558112
H	-0.539574	-2.285119	-0.321840
H	-0.241223	-3.918275	0.353772
H	0.512331	-3.421197	-1.202591
C	-1.387287	-5.296963	-1.599030
H	-2.269650	-5.667330	-2.123820
H	-0.500467	-5.414011	-2.223824
H	-1.256497	-5.836830	-0.659945

C	-2.801711	-3.631989	-0.455213
H	-3.675992	-3.959029	-1.020939
H	-2.698964	-4.224316	0.455875
H	-2.864789	-2.567443	-0.214866
C	-2.241929	3.153148	1.559018
C	-2.447246	2.139210	2.501132
C	-1.785355	2.152528	3.717866
C	-0.888332	3.203182	3.970137
C	-0.678211	4.210296	3.023300
C	-1.362617	4.197329	1.797870
C	-3.050975	2.815857	0.341643
C	-3.387881	1.099189	1.928249
H	-1.944879	1.359734	4.440148
H	-0.343921	3.229626	4.910483
H	0.030135	5.006928	3.234170
H	-1.194875	4.961986	1.046339
C	-3.778128	1.587823	0.621033
O	-3.029126	3.441100	-0.728596
O	-3.670896	0.061293	2.548931
C	-4.554654	1.057388	-0.464024
H	-4.831764	1.864114	-1.141662
C	-5.631272	0.036798	-0.335455
C	-5.564024	-1.046764	0.556611
C	-6.729484	0.118452	-1.207868
C	-6.569215	-2.013951	0.563631
H	-4.745705	-1.085540	1.267221
C	-7.734574	-0.847106	-1.195296
H	-6.789813	0.950852	-1.905089
C	-7.655179	-1.924311	-0.310630
H	-6.510959	-2.837978	1.271176
H	-8.578805	-0.757489	-1.873899
H	-8.437201	-2.679035	-0.295767

INT2

Zero-point correction= 0.853081 (Hartree/Particle)
Thermal correction to Energy= 0.909566
Thermal correction to Enthalpy= 0.910510
Thermal correction to Gibbs Free Energy= 0.755490
E(solv) = -5446.13014894 A.U.

C	-1.425480	-0.022789	-0.906909
C	-2.011923	-1.111389	-0.033148
C	-3.362007	-1.714010	-0.380581
H	-3.286919	-2.399868	-1.239232
H	-3.652590	-2.300475	0.493699

C	-0.627115	1.001008	-0.299044
H	-0.582163	0.950081	0.783358
C	-0.306527	2.335641	-0.849494
C	0.660509	3.100444	-0.172128
C	-0.945794	2.898112	-1.966165
C	1.001262	4.373091	-0.611857
H	1.137457	2.691421	0.710593
C	-0.601878	4.176099	-2.402768
H	-1.780311	2.387475	-2.427028
C	0.376747	4.915164	-1.737317
H	1.756865	4.940685	-0.077956
H	-1.124720	4.603342	-3.253715
H	0.639551	5.911522	-2.081631
O	-1.348356	-1.578274	0.896922
Pd	0.717139	-0.400796	-1.284798
C	-1.284750	-0.284532	-2.280132
H	-1.709696	-1.188503	-2.703772
H	-1.041068	0.492692	-2.993640
P	2.603630	-0.197404	-0.008689
O	3.129855	-1.646229	0.535860
O	3.941375	0.340432	-0.774920
O	2.759639	0.760801	1.323469
C	1.878934	0.849277	2.394910
C	2.129173	1.881429	3.298323
C	0.797896	-0.013888	2.570687
C	1.274946	2.052320	4.387023
H	2.979887	2.533750	3.131863
C	-0.073978	0.192736	3.642180
H	0.577523	-0.793658	1.854973
C	0.170123	1.215507	4.558785
H	1.466419	2.855776	5.092278
H	-0.967136	-0.420436	3.716862
H	-0.508286	1.373776	5.390947
C	4.257846	-1.798806	1.352431
C	4.083142	-1.851737	2.732464
C	5.509521	-1.948300	0.762150
C	5.198961	-2.065470	3.543286
H	3.093318	-1.709440	3.153583
C	6.615970	-2.161972	1.585172
H	5.599334	-1.889864	-0.316558
C	6.463916	-2.221730	2.972688
H	5.077050	-2.103395	4.621785
H	7.599082	-2.279132	1.138880
H	7.329542	-2.386246	3.607330

C	3.851642	1.567227	-1.456725
C	3.209826	1.628000	-2.691813
C	4.430568	2.688241	-0.870539
C	3.137978	2.859087	-3.345645
H	2.781108	0.727977	-3.119900
C	4.368069	3.907320	-1.546712
H	4.914602	2.591585	0.095068
C	3.716455	3.995704	-2.778424
H	2.625629	2.925364	-4.300414
H	4.819734	4.790024	-1.103172
H	3.655084	4.949731	-3.293079
Br	1.776808	-2.064461	-2.886802
N	0.042877	-4.652569	0.015083
C	-0.659509	-4.218174	-1.243687
H	-1.347697	-5.009681	-1.546029
H	-1.203841	-3.306489	-1.019461
H	0.086023	-4.006212	-2.012285
C	1.083162	-3.623068	0.382625
H	0.566752	-2.683111	0.563792
H	1.600959	-3.960935	1.281444
H	1.771208	-3.503723	-0.451934
C	0.714131	-5.967970	-0.225064
H	-0.041495	-6.712527	-0.481491
H	1.421391	-5.849550	-1.047185
H	1.241578	-6.265649	0.682460
C	-0.957915	-4.766482	1.131868
H	-1.726667	-5.484994	0.841367
H	-0.438644	-5.112698	2.027304
H	-1.390389	-3.777168	1.299714
C	-3.218081	2.753058	0.788725
C	-3.157897	1.979488	1.955683
C	-2.656984	2.503970	3.133821
C	-2.188427	3.828713	3.123234
C	-2.238965	4.595304	1.956077
C	-2.766374	4.061284	0.768556
C	-3.761975	1.885134	-0.318822
C	-3.644798	0.578650	1.646194
H	-2.622653	1.895886	4.031597
H	-1.775631	4.261100	4.031204
H	-1.862639	5.614865	1.966997
H	-2.796437	4.639646	-0.149443
C	-3.961495	0.573938	0.246149
O	-3.920995	2.244004	-1.495118
O	-3.672787	-0.342783	2.485204

C	-4.380074	-0.580415	-0.624159
H	-4.269589	-0.219685	-1.654028
C	-5.807573	-1.068770	-0.454026
C	-6.292231	-1.424354	0.813878
C	-6.655113	-1.191205	-1.559346
C	-7.595913	-1.895066	0.962404
H	-5.639061	-1.314058	1.675686
C	-7.962027	-1.661049	-1.411088
H	-6.290466	-0.903816	-2.542997
C	-8.435740	-2.016992	-0.148361
H	-7.961848	-2.161133	1.951010
H	-8.609427	-1.742951	-2.280543
H	-9.453257	-2.379788	-0.028298

INT3

Zero-point correction= 0.855376 (Hartree/Particle)
Thermal correction to Energy= 0.911146
Thermal correction to Enthalpy= 0.912091
Thermal correction to Gibbs Free Energy= 0.759467
E(solv) = -5446.14538204 A.U.

C	1.06906500	-0.95874600	-1.03220700
C	1.71246000	0.36949800	-1.34312500
C	1.39145700	1.58121300	-0.50986800
C	0.25392200	-1.10490300	0.13048200
H	0.11005600	-0.23592700	0.75721600
C	-0.02144400	-2.37085300	0.83272000
C	-1.14891600	-2.45065200	1.66764700
C	0.86477000	-3.45802100	0.78356600
C	-1.39892800	-3.60174800	2.40656900
H	-1.82476300	-1.60530900	1.72604700
C	0.62048700	-4.60353300	1.53673700
H	1.77509400	-3.37915300	0.19895600
C	-0.51604300	-4.68305400	2.34336700
H	-2.28420700	-3.65238600	3.03214500
H	1.32923400	-5.42604100	1.50969200
H	-0.70535600	-5.57708700	2.93088500
O	2.50945200	0.43755800	-2.27710300
Pd	-0.97811700	-0.93218600	-1.66519200
C	0.94996500	-1.90796500	-2.07645400
H	1.38948900	-1.67929800	-3.04019300
H	0.71127400	-2.94604900	-1.87681800
P	-2.63137300	0.37137500	-0.77255800
O	-2.97467200	1.78678100	-1.51008600

O	-4.18032100	-0.15496200	-0.71128900
O	-2.27456100	0.77732500	0.77159100
C	-2.95892600	1.61468900	1.65755000
C	-4.15608700	2.25954100	1.34020100
C	-2.35084000	1.77145600	2.90289200
C	-4.74228000	3.08806700	2.30128900
H	-4.61994900	2.12923200	0.37185700
C	-2.95211100	2.60551800	3.84459800
H	-1.42005300	1.25253900	3.10221600
C	-4.14667700	3.26774200	3.54963100
H	-5.67207200	3.59571900	2.06121300
H	-2.47928500	2.73467200	4.81400100
H	-4.60986400	3.91698900	4.28670000
C	-1.89920300	2.63516000	-1.83019000
C	-1.24644200	2.46903700	-3.04981700
C	-1.52867200	3.62505900	-0.92258500
C	-0.20502300	3.34247900	-3.37078600
H	-1.55040800	1.66603900	-3.71333600
C	-0.48135500	4.48650600	-1.25452600
H	-2.04583400	3.70950000	0.02640500
C	0.17504000	4.35016700	-2.47993100
H	0.31382800	3.22472900	-4.31757400
H	-0.16372600	5.24030600	-0.54153600
H	0.99343200	5.01859800	-2.73093800
C	-4.36712300	-1.42045300	-0.12928000
C	-4.12162700	-2.56712800	-0.88186100
C	-4.78975200	-1.48344500	1.19637900
C	-4.30454100	-3.81210900	-0.27732000
H	-3.78650100	-2.47196800	-1.90971900
C	-4.97859500	-2.73664500	1.78191400
H	-4.96285100	-0.56479100	1.74667900
C	-4.73120800	-3.90003400	1.04933600
H	-4.10934900	-4.71418200	-0.84946400
H	-5.31374100	-2.80000300	2.81325500
H	-4.87080600	-4.87276000	1.51166800
Br	-2.06693400	-1.28222700	-3.88309400
C	3.55652600	-1.23797200	2.51140000
C	2.29591900	-1.06800300	3.09153600
C	1.76859300	-2.00185000	3.96052500
C	2.54385900	-3.14054900	4.25111000
C	3.80389900	-3.31104700	3.67826800
C	4.32844000	-2.34988700	2.79146100
C	3.78120600	-0.09049800	1.56135300
C	1.68261100	0.18685900	2.51639600

H	0.77556500	-1.86818900	4.37773800
H	2.15243500	-3.90021800	4.92216000
H	4.38531100	-4.19829900	3.91585100
H	5.30762000	-2.47748600	2.33690200
C	2.62782900	0.73511500	1.58285400
O	4.82339800	0.00891200	0.85363900
O	0.52457400	0.54811400	2.79777100
C	2.44972900	1.87441900	0.61099200
H	3.41371200	1.97713400	0.09952500
C	2.09285500	3.24609000	1.17148700
C	0.93436400	3.44790800	1.93508000
C	2.85740500	4.36174700	0.81262500
C	0.55541600	4.73089300	2.32670600
H	0.34284900	2.58757700	2.22288100
C	2.48124000	5.64891400	1.20302500
H	3.75337200	4.22202600	0.21134600
C	1.32406000	5.83909000	1.96013500
H	-0.34941200	4.86122100	2.91529000
H	3.09091800	6.50133800	0.91410900
H	1.02700800	6.83936700	2.26415800
H	0.40351700	1.51479300	-0.05602700
H	1.37976200	2.43004800	-1.19496800

INT3-iso

Zero-point correction= 0.854254 (Hartree/Particle)
Thermal correction to Energy= 0.910258
Thermal correction to Enthalpy= 0.911202
Thermal correction to Gibbs Free Energy= 0.756633
E(solv) = -5446.13992387 A.U.

C	-0.91539900	0.44279400	-0.11169500
C	-1.37006100	0.46238800	-1.55782200
C	-2.38728300	1.48127000	-1.99877000
H	-2.16611400	2.46296500	-1.57347300
H	-2.31552100	1.56721200	-3.08612000
C	-0.01480300	-0.60782800	0.24742100
H	0.28284500	-1.23656400	-0.58552200
C	0.15089400	-1.21902200	1.58151700
C	1.27120800	-2.03696200	1.81845900
C	-0.80072800	-1.07186500	2.60391800
C	1.44022700	-2.67835100	3.04175800
H	2.02617400	-2.13288200	1.04587200
C	-0.62966300	-1.71684900	3.82767800
H	-1.69685500	-0.49367400	2.42761500
C	0.48960400	-2.52048500	4.05430400

H	2.32565500	-3.28270300	3.21036700
H	-1.38037600	-1.58807700	4.60268000
H	0.62302100	-3.01694100	5.01139500
O	-0.95242800	-0.39745600	-2.32965100
Pd	1.06411900	1.26250700	0.22581900
C	-1.05847400	1.55638600	0.74264900
H	-1.53825000	2.46153600	0.39703700
H	-0.98347200	1.45930500	1.81890700
P	3.06438000	0.51642200	-0.55036800
O	2.98261300	-1.06320300	-1.04437700
O	3.65164700	1.31839100	-1.84846600
O	4.44579200	0.54979200	0.32287500
C	4.38592800	0.01599200	1.62197500
C	3.71780900	0.71271200	2.62768900
C	5.02199600	-1.19928600	1.86329000
C	3.68017500	0.16001100	3.90868400
H	3.24349400	1.66267000	2.40140900
C	4.98641700	-1.73020400	3.15420900
H	5.53444200	-1.70551100	1.05307100
C	4.31120400	-1.05682200	4.17480500
H	3.15509900	0.68825900	4.69870600
H	5.48700300	-2.67265800	3.35813100
H	4.27946900	-1.47775200	5.17547500
C	4.05129300	-1.89119500	-1.37395400
C	3.90417800	-3.23872400	-1.04346300
C	5.20660600	-1.42664900	-2.00367200
C	4.93099400	-4.13416900	-1.33840900
H	2.99259400	-3.56374600	-0.55282000
C	6.22891400	-2.33433200	-2.28637800
H	5.30524000	-0.37956600	-2.26009900
C	6.09973500	-3.68452900	-1.95666000
H	4.81829700	-5.18292000	-1.07868200
H	7.13284400	-1.97653600	-2.77046000
H	6.90187000	-4.38081300	-2.18135600
C	2.71360200	1.94787500	-2.68620800
C	1.70937800	1.20252900	-3.29930400
C	2.81180800	3.32472400	-2.85204800
C	0.77144500	1.86352800	-4.09544800
H	1.64509500	0.13188200	-3.14183000
C	1.87828500	3.96940000	-3.66273800
H	3.58299100	3.87005000	-2.32123200
C	0.85552500	3.24373500	-4.28051200
H	-0.02606400	1.28923000	-4.55575200
H	1.93767100	5.04579000	-3.79165800

H	0.12366900	3.75575700	-4.89819000
Br	1.87736400	3.56033800	0.73863100
N	-2.81153100	-3.98796500	-0.38045500
C	-2.39963900	-3.80300100	1.05799800
H	-2.71612000	-2.80244500	1.35851300
H	-2.88902500	-4.57487700	1.65564800
H	-1.31483000	-3.89807100	1.12793000
C	-2.45960000	-5.36188000	-0.84472100
H	-2.98715500	-6.09350100	-0.22991500
H	-2.75685200	-5.46780700	-1.88928600
H	-1.38144200	-5.50158400	-0.74851600
C	-2.10441100	-2.96015800	-1.22793700
H	-2.40487400	-1.98650000	-0.84593700
H	-1.02988900	-3.11405200	-1.13428800
H	-2.42693900	-3.07771500	-2.26159100
C	-4.29682800	-3.76131900	-0.50331500
H	-4.49685700	-2.74604600	-0.15625600
H	-4.57793800	-3.85884500	-1.55211700
H	-4.80967000	-4.50647000	0.10834600
C	-4.41284300	2.05555500	2.08168000
C	-4.15052500	0.68316500	2.20113300
C	-4.12986900	0.06564300	3.43936600
C	-4.36925700	0.85714400	4.57953700
C	-4.62088200	2.22348300	4.45875300
C	-4.64655600	2.84149500	3.19286200
C	-4.35759000	2.40481100	0.60812000
C	-3.89218500	0.12806100	0.82317600
H	-3.93380200	-1.00019800	3.52525800
H	-4.35766400	0.39904600	5.56525200
H	-4.79812300	2.81708900	5.35148100
H	-4.83926700	3.90482700	3.08410500
C	-4.06565900	1.17372600	-0.09418300
O	-4.49853300	3.53634700	0.13721800
O	-3.52982200	-1.07165300	0.62438200
C	-3.86428400	1.14559400	-1.57811000
H	-4.42393100	2.01417100	-1.95605900
C	-4.46720000	-0.08710300	-2.25027800
C	-5.72869900	-0.53253800	-1.82016600
C	-3.86877200	-0.76145600	-3.32213900
C	-6.36949700	-1.60459700	-2.43562000
H	-6.20189600	-0.02157500	-0.98745800
C	-4.50507700	-1.84541300	-3.93764500
H	-2.88751400	-0.46650000	-3.67097300
C	-5.75876800	-2.27309600	-3.50198200

H	-7.35061400	-1.91651500	-2.08625400
H	-4.01803900	-2.34391300	-4.77270600
H	-6.26073500	-3.10339200	-3.99238400

TS2

Zero-point correction= 0.861609 (Hartree/Particle)
Thermal correction to Energy= 0.915292
Thermal correction to Enthalpy= 0.916236
Thermal correction to Gibbs Free Energy= 0.771262
E(solv) = -5442.62904608 A.U.
Imaginary frequency = 318.42

C	-0.88678300	-1.29216500	0.33457900
C	-1.21547000	-0.73617800	1.66035000
C	-1.14637600	0.76916900	1.77224800
C	-0.69276400	-0.30959900	-0.73302300
H	-0.07818100	0.53965200	-0.46643100
C	-0.51570300	-0.68582500	-2.14729800
C	0.50014100	-0.05128000	-2.88139600
C	-1.35275700	-1.60345500	-2.80159100
C	0.68391300	-0.33988100	-4.23063800
H	1.15337000	0.65483700	-2.37999500
C	-1.17801700	-1.87948900	-4.15420700
H	-2.16112200	-2.07395400	-2.25286000
C	-0.15783700	-1.25007100	-4.87188500
H	1.48954300	0.14194300	-4.77393000
H	-1.84402200	-2.57833800	-4.65196900
H	-0.02156100	-1.46888700	-5.92735700
O	-1.60685100	-1.43000800	2.61157900
Pd	1.17095100	-1.90056100	0.49630800
C	-0.75651600	-2.70855700	0.17098800
H	-1.04660200	-3.35151400	0.99376600
H	-0.76464200	-3.16391800	-0.81201600
P	2.72695200	-0.25592900	0.70293600
O	3.33239000	0.14895200	2.18855900
O	4.20079700	-0.27783200	-0.03369300
O	2.08332100	1.18319600	0.17308600
C	2.59581200	2.47503200	0.24098000
C	3.89113100	2.77663000	0.67198900
C	1.71169600	3.48126000	-0.14964300
C	4.28482700	4.11652300	0.72498600
H	4.57304600	1.99034700	0.96500900
C	2.12315700	4.81147800	-0.08598500

H	0.72043100	3.21742700	-0.50175000
C	3.40806600	5.13718600	0.35445100
H	5.28928600	4.35602400	1.06278700
H	1.42662100	5.59076800	-0.38169200
H	3.72552800	6.17472700	0.40375400
C	2.37716400	0.34375800	3.19057500
C	1.69768400	-0.74759500	3.73224800
C	2.11982300	1.64719200	3.61536100
C	0.73545000	-0.51920100	4.71715100
H	1.90975400	-1.74762900	3.36776400
C	1.16394700	1.85843700	4.61056000
H	2.66263400	2.46998100	3.16370300
C	0.46789600	0.77808100	5.15898900
H	0.18525400	-1.36107800	5.12414800
H	0.95908700	2.87017000	4.94921800
H	-0.28151600	0.94803200	5.92689200
C	4.18754400	-0.50671900	-1.41790600
C	3.98794500	-1.79562000	-1.90985800
C	4.38021600	0.58195200	-2.26684000
C	3.98871000	-1.98890200	-3.29230600
H	3.81782400	-2.61718800	-1.22049900
C	4.38966100	0.36915800	-3.64668800
H	4.52152400	1.57064800	-1.84371100
C	4.19268200	-0.91469000	-4.16103700
H	3.82568400	-2.98773100	-3.68618300
H	4.54850400	1.20980700	-4.31650000
H	4.19659300	-1.07660200	-5.23536900
Br	2.50749600	-4.02142900	0.74349300
C	-3.63808000	0.46664600	-2.46439300
C	-2.70020400	1.41630300	-2.88597900
C	-2.56932500	1.75450600	-4.22334200
C	-3.42374500	1.12958000	-5.14116800
C	-4.37751200	0.19443500	-4.71806200
C	-4.49303400	-0.15194300	-3.36674700
C	-3.46998500	0.25017100	-1.00120300
C	-1.90253500	1.87150700	-1.70035200
H	-1.81848000	2.47233300	-4.53708300
H	-3.34789400	1.37115600	-6.19748900
H	-5.02756200	-0.27292500	-5.45240300
H	-5.21280300	-0.89180400	-3.02928600
C	-2.31027100	1.01790700	-0.55508600
O	-4.19384200	-0.48923100	-0.30784700
O	-1.02499700	2.72388100	-1.73364900
C	-2.24192400	1.45800600	0.91107800

H	-3.19743500	1.12904600	1.33957300
C	-2.21529300	2.97782000	1.05213300
C	-1.15026600	3.67192800	1.63144300
C	-3.31189200	3.71311100	0.58007200
C	-1.16988300	5.06632800	1.71604300
H	-0.28690500	3.13884700	2.01131100
C	-3.33806200	5.10218300	0.66637500
H	-4.14791700	3.18512400	0.12570500
C	-2.26023800	5.78732500	1.23390000
H	-0.32046100	5.58370300	2.15323500
H	-4.19653600	5.65128700	0.28882100
H	-2.27353900	6.87177900	1.29830400
H	-0.15537200	1.11083800	1.46545600
H	-1.28082600	1.04176000	2.82038000

TS2-new-iso

Zero-point correction= 0.860042 (Hartree/Particle)
Thermal correction to Energy= 0.914108
Thermal correction to Enthalpy= 0.915053
Thermal correction to Gibbs Free Energy= 0.767107
E(solv) = -5442.61941956 A.U.
Imaginary frequency = 310.01

C	-0.95307700	-0.69109400	0.17383000
C	-1.26527400	-1.39180800	1.43850500
C	-2.34303100	-2.44686500	1.32914500
H	-2.04036700	-3.21461200	0.61059800
H	-2.46288200	-2.93789700	2.29680400
C	-0.05297700	0.43179700	0.26817700
H	0.29058000	0.58546600	1.28688700
C	0.00316300	1.66297900	-0.54930200
C	0.99075000	2.61263400	-0.21446000
C	-0.87463200	1.98252800	-1.59809300
C	1.09466400	3.82353500	-0.88977000
H	1.70343200	2.35863700	0.56206100
C	-0.77757500	3.20155800	-2.27000200
H	-1.66499800	1.30498700	-1.87995000
C	0.20196300	4.13136300	-1.92146800
H	1.88659300	4.51846100	-0.62578800
H	-1.47814800	3.41743000	-3.07260300
H	0.28149500	5.07451000	-2.45448100
O	-0.79516600	-1.05158400	2.53068800

Pd	1.09019500	-1.07550100	-0.62418800
C	-1.60657800	-1.18149100	-1.00006300
H	-1.68531300	-2.25146300	-1.14511400
H	-1.53167000	-0.63989900	-1.93456600
P	3.16051200	-0.58365200	0.03110100
O	3.23906200	0.58276400	1.22080800
O	3.97861300	-1.84163000	0.70033100
O	4.36565000	-0.10576200	-0.98311200
C	4.03367500	0.95569800	-1.83868400
C	3.11605100	0.75735200	-2.87088300
C	4.63867000	2.19129200	-1.62151000
C	2.78927800	1.83815600	-3.69081600
H	2.66917800	-0.22180700	-3.01400400
C	4.31390900	3.25702200	-2.46268700
H	5.34566100	2.30264600	-0.80697000
C	3.38376700	3.08563100	-3.48997900
H	2.06526300	1.69924400	-4.48803700
H	4.78490400	4.22373600	-2.30681400
H	3.12343800	3.92125500	-4.13313200
C	4.38189400	1.21607600	1.68967300
C	4.22408700	2.53886700	2.10840000
C	5.62906300	0.59100600	1.75947400
C	5.32296600	3.24695400	2.59205200
H	3.24323600	2.99832100	2.04566200
C	6.72169300	1.31493300	2.23915400
H	5.74102600	-0.43695700	1.44052500
C	6.57878800	2.63986100	2.65456400
H	5.19650800	4.27663100	2.91501500
H	7.69321200	0.83161300	2.28782300
H	7.43644100	3.19267500	3.02578000
C	3.21618900	-2.87059100	1.27305100
C	2.22243200	-2.58454600	2.20852000
C	3.46784800	-4.17127300	0.85169400
C	1.45086100	-3.63144800	2.71649500
H	2.03350500	-1.56377600	2.52062400
C	2.70607400	-5.21028200	1.38445900
H	4.22156600	-4.34587200	0.09271100
C	1.69326000	-4.94374600	2.31001000
H	0.65449700	-3.40263800	3.41762500
H	2.88811400	-6.22832200	1.05329800
H	1.09184800	-5.75698000	2.70591500
Br	1.52294400	-3.00012700	-2.18222900
N	-2.58809400	3.21524600	2.44990000
C	-2.27290300	3.79341100	1.09292000

H	-2.64652500	3.09864600	0.34209500
H	-2.76690700	4.76414100	1.01594900
H	-1.19208800	3.90303800	0.99540000
C	-2.14302300	4.16103500	3.51733000
H	-2.67577200	5.10664000	3.40180900
H	-2.36451100	3.72316200	4.49189800
H	-1.06859000	4.32284600	3.41729200
C	-1.87110600	1.89701500	2.61147200
H	-2.21682200	1.24094600	1.81687800
H	-0.80062100	2.07685100	2.53106200
H	-2.12195900	1.48073400	3.58584600
C	-4.07123800	2.97715400	2.55859700
H	-4.35372200	2.27111400	1.77856900
H	-4.28604700	2.54889900	3.53708800
H	-4.58282400	3.93363300	2.43399500
C	-4.49356300	-0.71446500	-2.70108600
C	-4.24298900	0.52349200	-2.09431100
C	-4.38202800	1.71051300	-2.79890300
C	-4.78546000	1.63393000	-4.14051800
C	-5.03483500	0.39726300	-4.74530400
C	-4.88789000	-0.79942200	-4.02653500
C	-4.24216200	-1.79550700	-1.68593300
C	-3.81125100	0.29215000	-0.67777900
H	-4.17913600	2.66456400	-2.32118000
H	-4.90578200	2.54518500	-4.71977900
H	-5.34450000	0.36510800	-5.78595100
H	-5.07259900	-1.76592600	-4.48509200
C	-3.67606700	-1.13602000	-0.48792800
O	-4.44952500	-2.99237000	-1.83920400
O	-3.55096500	1.20313400	0.12837200
C	-3.71718000	-1.86856300	0.84896200
H	-4.32430100	-2.76086200	0.64308000
C	-4.46230300	-1.08397100	1.93048000
C	-5.75692500	-0.61614400	1.65218500
C	-3.94603200	-0.85759200	3.21265500
C	-6.50941900	0.05488300	2.61267700
H	-6.17831200	-0.78469600	0.66466000
C	-4.70338500	-0.19305300	4.18378500
H	-2.94437500	-1.18520600	3.46380000
C	-5.98662600	0.26695700	3.89230100
H	-7.51116500	0.39816600	2.36825400
H	-4.28509500	-0.04973400	5.17766100
H	-6.57891900	0.77148700	4.65123400

INT4

Zero-point correction= 0.856380 (Hartree/Particle)
Thermal correction to Energy= 0.911713
Thermal correction to Enthalpy= 0.912657
Thermal correction to Gibbs Free Energy= 0.760996
E(solv) = -5446.14831156 A.U.

C	0.67758400	-1.36106900	0.22448600
C	0.92976000	-1.44919100	-1.21147400
C	1.06134500	-0.10238600	-1.89811500
C	0.86450500	0.04139100	0.80213600
H	0.11210600	0.69557900	0.35949200
C	0.67147100	0.14080700	2.29947100
C	-0.42398300	0.86296000	2.78729000
C	1.53413500	-0.47306900	3.21687400
C	-0.64957000	0.97781200	4.15885900
H	-1.11153200	1.32543900	2.08592100
C	1.31616600	-0.35452200	4.58891500
H	2.37220800	-1.06355900	2.86004600
C	0.22315400	0.37323200	5.06374500
H	-1.51600900	1.52804300	4.50966400
H	1.99617100	-0.83623900	5.28655900
H	0.04994300	0.46095700	6.13284900
O	1.13242500	-2.50648200	-1.84204300
Pd	-1.43834100	-1.80625700	0.29385100
C	0.42514600	-2.53928800	0.98325400
H	0.57177700	-3.50998900	0.52327000
H	0.45197000	-2.52052400	2.06650900
P	-2.78811100	-0.24080600	-0.59045700
O	-3.40248200	-0.37011400	-2.13117200
O	-4.23468800	0.22788600	0.05705600
O	-1.97596500	1.22116600	-0.67543100
C	-2.39120100	2.44791900	-1.17119100
C	-3.62647200	2.66876100	-1.79001400
C	-1.46891000	3.48661000	-1.02539000
C	-3.91646600	3.94584100	-2.27772100
H	-4.34305700	1.86594000	-1.89045900
C	-1.77479000	4.75188500	-1.52348600
H	-0.52922600	3.29065200	-0.52296300
C	-2.99789600	4.98914100	-2.15503700
H	-4.87511300	4.11802200	-2.75932100
H	-1.04900500	5.55268200	-1.41053600
H	-3.23557000	5.97604400	-2.54133600
C	-2.47716800	-0.69055500	-3.12374800

C	-1.93883500	-1.97558300	-3.19520000
C	-2.10278200	0.30468000	-4.02761300
C	-1.00677200	-2.26157700	-4.19342300
H	-2.23086900	-2.72085900	-2.46264300
C	-1.17911500	0.00051400	-5.02863100
H	-2.53427000	1.29517600	-3.93428600
C	-0.62768700	-1.28066800	-5.11183000
H	-0.56664600	-3.25222200	-4.23664100
H	-0.88682800	0.76935900	-5.73861500
H	0.09599100	-1.51172200	-5.88871600
C	-4.23475900	0.57485600	1.41375200
C	-4.19361000	-0.41887200	2.39067900
C	-4.28970100	1.92792700	1.74733900
C	-4.21549400	-0.03707600	3.73289300
H	-4.12010200	-1.46263300	2.09978200
C	-4.31875100	2.29262700	3.09482900
H	-4.31357300	2.67149100	0.95833800
C	-4.28326500	1.31194600	4.08931900
H	-4.17471700	-0.80494500	4.49984700
H	-4.36913800	3.34424500	3.36341900
H	-4.30794400	1.59863700	5.13731700
Br	-2.93744500	-3.66769300	1.14906300
N	4.31911400	-3.86049900	-1.15620700
C	3.17154400	-3.90195800	-0.17276100
H	2.24082200	-3.89864400	-0.73997700
H	3.28592500	-4.79446800	0.44492700
H	3.21033200	-2.99474900	0.42589700
C	5.60821700	-3.73027000	-0.40312000
H	5.72161500	-4.59564000	0.25233300
H	6.43434100	-3.68845300	-1.11583800
H	5.56004900	-2.81202000	0.18364000
C	4.15869000	-2.67061800	-2.06931900
H	3.16549900	-2.70921900	-2.52002400
H	4.23078700	-1.77920500	-1.44940400
H	4.95656600	-2.70303700	-2.81396400
C	4.31020500	-5.11475700	-1.97238500
H	3.35826200	-5.17110300	-2.50162200
H	5.13734700	-5.08461500	-2.68404300
H	4.41781900	-5.97224600	-1.30615900
C	4.03397100	1.13407800	1.84989000
C	3.27525200	2.30816500	1.82958300
C	3.58802600	3.38453400	2.65392200
C	4.69071400	3.25580400	3.50008800
C	5.45695800	2.07709000	3.51893100

C	5.13698100	1.00085800	2.69143900
C	3.47421400	0.16411100	0.88519000
C	2.15510300	2.16666600	0.86433200
H	2.98315200	4.28501600	2.63050000
H	4.96401400	4.07573500	4.15780800
H	6.30714400	2.00746800	4.19117500
H	5.71497900	0.08213300	2.70121700
C	2.20095700	0.74080400	0.27668200
O	3.99228900	-0.91501200	0.59994700
O	1.31156800	3.01162800	0.64321800
C	2.26239800	0.66262500	-1.30178200
H	3.14769800	0.05643600	-1.52114900
C	2.50410000	2.01883700	-1.94098500
C	1.49384500	2.73765700	-2.58556900
C	3.77795700	2.59554800	-1.84266200
C	1.74477200	4.00976000	-3.10127200
H	0.49825700	2.32060800	-2.67638500
C	4.03403500	3.86482000	-2.35845300
H	4.57626500	2.04481500	-1.34793700
C	3.01289900	4.57951000	-2.98841900
H	0.93811500	4.55606400	-3.58177100
H	5.02814000	4.29475400	-2.26986400
H	3.20609300	5.57086100	-3.38849900
H	0.13913900	0.46577600	-1.76139300
H	1.20677700	-0.25561400	-2.96942900

1q-TS2

Zero-point correction= 0.851408 (Hartree/Particle)
Thermal correction to Energy= 0.906145
Thermal correction to Enthalpy= 0.907090
Thermal correction to Gibbs Free Energy= 0.760289
E(solv) = -5478.75061370 A.U.
Imaginary frequency = 313.54

C	1.24011400	1.13814800	-0.39296600
C	1.79831800	1.31081100	0.96453900
C	1.61330100	0.13396500	1.89007300
C	0.65440400	-0.15891200	-0.66362000
H	0.03969700	-0.61239800	0.09753500
O	2.43934900	2.31199400	1.30613000
Pd	-0.70420900	2.04299500	-0.55464000
C	1.26013800	2.20943400	-1.33758700
H	1.74398100	3.13384200	-1.04496900

H	1.19348800	2.00401200	-2.39930000
P	-2.45021600	1.10975200	0.58751900
O	-2.86948600	1.74209600	2.05743500
O	-3.99729900	1.00346800	0.04809900
O	-2.06852200	-0.45810800	0.96324500
C	-2.87658200	-1.47490600	1.45700300
C	-4.02611100	-1.23847800	2.21448700
C	-2.44759400	-2.77171800	1.17140900
C	-4.75610900	-2.33375100	2.68352600
H	-4.34603800	-0.22637600	2.42531900
C	-3.18330700	-3.85086200	1.65922600
H	-1.54413400	-2.91980100	0.59046700
C	-4.34120900	-3.63849500	2.41214800
H	-5.65419000	-2.15762300	3.26892000
H	-2.84516500	-4.86045600	1.44411000
H	-4.91410900	-4.48205000	2.78596800
C	-1.78624700	1.94295000	2.92185700
C	-0.94299200	3.03833300	2.73451100
C	-1.56110600	1.01115900	3.93378300
C	0.14918900	3.19720800	3.58896800
H	-1.13440800	3.72965100	1.92004000
C	-0.47178500	1.19003300	4.78817300
H	-2.22810700	0.16161000	4.03444900
C	0.38500100	2.27956400	4.61509400
H	0.82466800	4.03262600	3.43627700
H	-0.28665400	0.46731200	5.57757800
H	1.23842300	2.40921700	5.27449600
C	-4.20124700	0.50422000	-1.24542600
C	-3.80259700	1.24394000	-2.35823700
C	-4.83645100	-0.72960100	-1.37217600
C	-4.04423700	0.71814100	-3.62937000
H	-3.31778900	2.20668800	-2.22670800
C	-5.08311000	-1.23331700	-2.65041000
H	-5.12542100	-1.27360600	-0.47976800
C	-4.68280900	-0.51536400	-3.78026400
H	-3.73474900	1.28530000	-4.50244700
H	-5.58388400	-2.19103200	-2.76007300
H	-4.87324800	-0.91299400	-4.77310500
Br	-1.67271800	4.23246500	-1.31989700
N	5.75482200	2.27874900	-0.12697100
C	4.50649200	2.64940400	-0.89297000
H	3.82684700	3.16217100	-0.21490200
H	4.80031200	3.27798500	-1.73552700
H	4.03783800	1.72724800	-1.23119900

C	6.62702100	1.42709400	-1.00445200
H	6.89862300	2.00458000	-1.89010300
H	7.52433900	1.15158300	-0.44684400
H	6.05102800	0.54236800	-1.28052600
C	5.38024200	1.49204600	1.10567000
H	4.67093300	2.07551100	1.69171000
H	4.89632400	0.57762000	0.76410400
H	6.29580700	1.28254600	1.66249700
C	6.47728500	3.52408900	0.27736000
H	5.81397300	4.12012000	0.90552900
H	7.37601100	3.25099000	0.83327500
H	6.74661900	4.08594100	-0.61855900
C	3.03963400	-2.21738600	-2.17236500
C	2.03351500	-3.14467000	-1.87961800
C	1.68000700	-4.13236600	-2.78567300
C	2.36473200	-4.17472000	-4.00858900
C	3.36929900	-3.24463600	-4.30240100
C	3.71976700	-2.24952100	-3.38076700
C	3.17978100	-1.29522600	-1.01100900
C	1.44909300	-2.82906900	-0.53135700
H	0.89776200	-4.84540300	-2.54534100
H	2.11434300	-4.93786200	-4.74003100
H	3.87954500	-3.29573000	-5.26000700
H	4.48980700	-1.51730700	-3.60313300
C	2.11122800	-1.58129500	-0.06145200
O	4.06171200	-0.42453000	-0.89323400
O	0.54948700	-3.47083400	-0.00445100
C	2.31282200	-1.18243700	1.40423400
H	3.38802800	-0.98137400	1.48700700
C	2.00228800	-2.30900400	2.38071200
C	0.69903600	-2.56515200	2.82277500
C	3.03797400	-3.12274600	2.84892500
C	0.43841400	-3.60949700	3.70590400
H	-0.12768500	-1.96519100	2.46212100
C	2.78404600	-4.16921400	3.73773400
H	4.05523000	-2.93657300	2.51024100
C	1.48080000	-4.41540000	4.16941500
H	-0.58483000	-3.79901800	4.01801400
H	3.60380500	-4.78867300	4.09182800
H	1.27797600	-5.22982600	4.85947100
H	0.54175500	-0.04011600	2.01655500
H	2.01201700	0.39690900	2.87135700
C	0.23194600	-0.55826900	-2.03943500
O	0.75349000	-0.21195500	-3.08211200

O	-0.78698000	-1.42806800	-1.94697500
C	-1.27064300	-2.02707300	-3.16814900
C	-2.06174500	-3.25340300	-2.75721100
H	-0.41195300	-2.27344400	-3.79837800
H	-1.89158400	-1.29067900	-3.68594300
H	-2.45421200	-3.76192900	-3.64478400
H	-1.42451400	-3.94643300	-2.19926500
H	-2.90231100	-2.96828700	-2.11933700

1q-TS2'

Zero-point correction= 0.850726 (Hartree/Particle)
Thermal correction to Energy= 0.905625
Thermal correction to Enthalpy= 0.906569
Thermal correction to Gibbs Free Energy= 0.758182
E(solv) = -5454.37801054 A.U.
Imaginary frequency = 184.65

C	-0.99141100	-0.32269900	-0.86641900
C	-1.70575600	-1.50276700	-1.43014900
C	-2.79801300	-1.17620700	-2.42043900
H	-2.39834700	-0.56522200	-3.23435100
H	-3.16579400	-2.10369800	-2.86312700
C	-0.10115000	-0.55471700	0.25519000
H	-0.03450700	-1.59460200	0.55243800
O	-1.51562200	-2.64309100	-1.00932200
Pd	1.14802400	-0.10480600	-1.36152300
C	-1.27446500	0.94558400	-1.39897700
H	-1.56143900	1.06685900	-2.43243900
H	-0.95414600	1.84010800	-0.88124400
P	3.15502200	-0.43783000	-0.45077600
O	2.98529400	-0.73648000	1.15629100
O	4.07663800	-1.67767200	-1.00208300
O	4.35550600	0.66801100	-0.52216100
C	4.06561400	2.01278900	-0.25227700
C	3.49123800	2.80573600	-1.24353400
C	4.41385200	2.52791700	0.99485500
C	3.27376500	4.15789700	-0.97233600
H	3.21657600	2.36314100	-2.19568200
C	4.19094800	3.88267800	1.24870500
H	4.86178400	1.87750800	1.73756800
C	3.62561000	4.69955800	0.26667300
H	2.82837300	4.78663500	-1.73772400

H	4.46618400	4.29794100	2.21401800
H	3.45670800	5.75357100	0.46747200
C	3.95520400	-0.71864000	2.15017100
C	3.51010800	-0.32355600	3.41226900
C	5.28482100	-1.07800900	1.92524500
C	4.41943300	-0.28046900	4.46780600
H	2.46439600	-0.05888800	3.53234200
C	6.18225700	-1.02334800	2.99437500
H	5.61101800	-1.38067900	0.93814300
C	5.75824000	-0.62590600	4.26321100
H	4.08071900	0.02877300	5.45281200
H	7.22074900	-1.29412200	2.82691600
H	6.46512000	-0.58581000	5.08657600
C	3.39510700	-2.87810900	-1.26217100
C	3.41244000	-3.87504600	-0.29069500
C	2.71872100	-3.03542200	-2.46998900
C	2.73904900	-5.07170900	-0.54326100
H	3.94615700	-3.70599900	0.63892700
C	2.04232400	-4.23395700	-2.70313000
H	2.70851400	-2.22453700	-3.19082700
C	2.05124400	-5.25066400	-1.74564400
H	2.74990100	-5.86084400	0.20330700
H	1.50233800	-4.36575600	-3.63585900
H	1.52197000	-6.17984400	-1.93537800
Br	1.79818000	0.51281000	-3.70390300
N	-3.04290500	-2.18116800	3.25812900
C	-2.44779000	-0.93573300	3.86371300
H	-2.63431300	-0.11628000	3.16971400
H	-2.93265500	-0.76547000	4.82744500
H	-1.37377500	-1.07556700	3.97649100
C	-2.89477100	-3.32464500	4.20726000
H	-3.42435900	-3.09199500	5.13298100
H	-3.31766100	-4.22037500	3.74957600
H	-1.83352000	-3.47547400	4.41122100
C	-2.33117700	-2.50084400	1.96541100
H	-2.48549500	-1.65723000	1.29799900
H	-1.27263000	-2.63597800	2.18263200
H	-2.76521700	-3.40641500	1.54369300
C	-4.49808300	-1.93776900	2.96283100
H	-4.55866300	-1.11840900	2.24673200
H	-4.92544900	-2.83900300	2.52612500
H	-5.00198100	-1.68543500	3.89805900
C	-3.74415000	3.26177400	-0.66868500
C	-3.48511600	2.61606800	0.54769700

C	-3.31762200	3.33424900	1.71949100
C	-3.40894200	4.73507600	1.65259600
C	-3.65413700	5.37903700	0.43695500
C	-3.82514300	4.64096800	-0.74715800
C	-3.90228900	2.20547900	-1.73503400
C	-3.44292300	1.13404400	0.30730300
H	-3.12071000	2.82528600	2.65884500
H	-3.28287900	5.32662900	2.55530800
H	-3.71666500	6.46326800	0.40938500
H	-4.02062600	5.12777000	-1.69786600
C	-3.63252400	0.91692900	-1.09343300
O	-4.18931500	2.40936800	-2.91175400
O	-3.23260500	0.29085800	1.20765600
C	-3.99238200	-0.38346200	-1.77936400
H	-4.58710400	-0.06762400	-2.64818600
C	-4.91657800	-1.25877100	-0.92850100
C	-6.06639100	-0.67398200	-0.37286600
C	-4.72342500	-2.63309800	-0.73625000
C	-6.99750100	-1.43138900	0.33400400
H	-6.23144100	0.39134200	-0.50746500
C	-5.66280000	-3.39902600	-0.03704500
H	-3.83497800	-3.11930500	-1.12017100
C	-6.80588400	-2.80684400	0.49802500
H	-7.88346400	-0.95119200	0.74106200
H	-5.49790900	-4.46815000	0.07638700
H	-7.54167800	-3.40512600	1.02901700
C	0.07437800	0.34502000	1.41575700
O	0.21697800	-0.05845400	2.56331300
O	0.08836200	1.65409000	1.10115000
C	0.32106000	2.58515200	2.17772600
C	0.28306800	3.97544300	1.57749900
H	1.29467100	2.35902200	2.62377000
H	-0.44988200	2.43860500	2.94117600
H	0.47511800	4.72020200	2.35822100
H	1.05240500	4.07806500	0.80874600
H	-0.69600300	4.18195700	1.13741900

TS1B

Zero-point correction= 0.681880 (Hartree/Particle)
Thermal correction to Energy= 0.728513
Thermal correction to Enthalpy= 0.729457

Thermal correction to Gibbs Free Energy= 0.596031
E(solv) = -2657.36860569 A.U.
Imaginary frequency=62.66

C	1.334915	-0.426093	-2.198757
C	1.629272	0.952036	-2.795877
C	1.679852	2.028241	-1.907510
H	1.344259	1.967453	-0.884983
H	1.853874	3.018612	-2.310103
C	1.143186	-0.647971	-0.792514
H	1.066756	0.234906	-0.168127
C	1.421933	-1.866960	-0.037974
C	1.010264	-1.905224	1.309164
C	2.118328	-2.971261	-0.565083
C	1.222862	-3.037600	2.083386
H	0.530240	-1.033371	1.737147
C	2.336744	-4.099731	0.216887
H	2.538861	-2.916714	-1.561635
C	1.878496	-4.143734	1.535658
H	0.897497	-3.052113	3.119306
H	2.897926	-4.933682	-0.191462
H	2.062706	-5.024094	2.144158
O	1.808854	0.980441	-4.034164
Pd	-0.808038	-0.780719	-1.854844
C	-5.853686	-2.269103	-0.694862
C	-4.904913	-1.692748	0.144993
C	-6.583702	-1.233923	1.792691
C	-7.554566	-1.790095	0.957800
C	-7.184815	-2.316373	-0.281267
H	-5.541630	-2.658714	-1.658201
H	-6.864520	-0.825329	2.758616
H	-8.592988	-1.817847	1.273523
H	-7.932184	-2.759493	-0.932794
C	-4.648527	1.402267	-0.464425
C	-7.032374	1.675440	-0.468305
C	-4.527358	2.219013	0.655995
C	-6.937321	2.475730	0.672693
H	-8.001839	1.459950	-0.906729
C	-5.685936	2.751156	1.225000
H	-3.551127	2.432674	1.073720
H	-7.834457	2.888416	1.124067
H	-5.603735	3.384719	2.103234
O	-3.585950	-1.667316	-0.329515

O	-3.506012	0.846765	-1.065191
P	-2.597141	-0.385856	-0.488806
C	0.974096	-1.423467	-3.104075
H	0.996906	-1.140638	-4.152526
H	0.884152	-2.471393	-2.839557
C	-5.246337	-1.191752	1.399305
H	-4.486546	-0.754639	2.033377
C	-5.884331	1.139774	-1.048086
H	-5.935990	0.501899	-1.922600
C	-1.423077	0.834017	1.600420
C	-0.998889	0.581998	2.905475
C	-0.887856	1.885392	0.855097
C	-0.001707	1.379698	3.463211
H	-1.441987	-0.244046	3.452245
C	0.117485	2.671845	1.427339
H	-1.228177	2.084020	-0.155213
C	0.564019	2.419780	2.722966
H	0.346174	1.175498	4.470911
H	0.561547	3.474407	0.848091
H	1.356257	3.023808	3.149552
O	-2.407081	-0.017949	1.107399
C	5.018559	-1.753750	0.073645
C	4.550979	-1.362715	1.331771
C	4.671713	-2.199214	2.433103
C	5.298591	-3.437295	2.249441
C	5.779649	-3.822175	0.990120
C	5.637328	-2.981409	-0.119661
C	4.715771	-0.665875	-0.906964
C	3.952814	0.011823	1.250300
H	4.290073	-1.888933	3.400675
H	5.416745	-4.111525	3.093360
H	6.265630	-4.787528	0.877513
H	5.993150	-3.267588	-1.104798
C	4.172161	0.476228	-0.129359
O	4.883816	-0.731637	-2.118609
O	3.369895	0.548266	2.194806
C	4.034707	1.691772	-0.771916
H	4.330650	1.645457	-1.817485
C	3.889721	3.045185	-0.245948
C	3.550284	3.344351	1.087204
C	4.139434	4.116075	-1.130464
C	3.458021	4.669522	1.508056
H	3.356180	2.528947	1.772639
C	4.048513	5.435489	-0.705036

H	4.395830	3.894238	-2.162707
C	3.702397	5.719308	0.619870
H	3.197258	4.885372	2.541295
H	4.244220	6.243692	-1.403890
H	3.626797	6.749889	0.955468

TS1C

Zero-point correction= 0.681447 (Hartree/Particle)
Thermal correction to Energy= 0.727972
Thermal correction to Enthalpy= 0.728916
Thermal correction to Gibbs Free Energy= 0.594583
E(solv) = -2657.36591119 A.U.
Imaginary frequency= 156.32

C	1.237397	0.074908	-1.589334
C	1.359590	1.573200	-1.341736
C	2.144796	2.264046	-2.261621
H	2.479696	1.768764	-3.166105
H	2.200728	3.343548	-2.216403
C	1.642408	-0.808852	-0.548606
H	1.957422	-0.337207	0.377257
C	1.934051	-2.235270	-0.699157
C	1.909107	-3.062684	0.439415
C	2.297003	-2.799042	-1.937820
C	2.183482	-4.421324	0.335630
H	1.681293	-2.620968	1.402823
C	2.569149	-4.159371	-2.037426
H	2.437550	-2.152175	-2.795639
C	2.500852	-4.976730	-0.906379
H	2.165527	-5.045138	1.224086
H	2.863164	-4.578620	-2.995118
H	2.723354	-6.036778	-0.986789
O	0.817799	1.997141	-0.287325
Pd	-0.571729	-0.604147	-0.454848
C	-1.077323	1.793175	2.156757
C	-1.692930	0.574662	1.901052
C	0.211957	-0.610128	2.806766
C	0.852173	0.604930	3.037874
C	0.196943	1.799243	2.722348
H	-1.561874	2.710426	1.843503
H	0.694948	-1.543911	3.077273
H	1.868399	0.625558	3.413985
H	0.704923	2.746313	2.868151
C	-5.125446	-1.446650	0.488365
C	-6.578569	-1.256107	2.387092

C	-6.214242	-1.640482	-0.356217
C	-7.685078	-1.422098	1.552962
H	-6.716390	-1.112401	3.454710
C	-7.499070	-1.619705	0.182836
H	-6.047323	-1.773087	-1.418646
H	-8.687874	-1.402295	1.968748
H	-8.354696	-1.751074	-0.472342
O	-2.921436	0.561089	1.223571
O	-3.839661	-1.474227	-0.076941
P	-2.824841	-0.204700	-0.259648
C	0.340802	-0.392408	-2.566008
H	-0.115694	0.324722	-3.240854
H	0.252322	-1.441249	-2.828950
C	-1.068160	-0.643939	2.226745
H	-1.608128	-1.580581	2.116420
C	-5.286015	-1.272414	1.860419
H	-4.418985	-1.133691	2.494156
C	-4.932831	1.216312	-1.216025
C	-5.648493	1.070307	-2.400495
C	-5.521173	1.728103	-0.061635
C	-6.999046	1.418702	-2.421143
H	-5.145834	0.680119	-3.279185
C	-6.875467	2.059174	-0.094834
H	-4.936451	1.836173	0.842985
C	-7.617586	1.903231	-1.267155
H	-7.567079	1.304797	-3.339733
H	-7.350144	2.439289	0.804574
H	-8.671360	2.164180	-1.282853
O	-3.582347	0.840033	-1.246896
C	5.039288	-1.493676	-0.299341
C	4.774878	-1.090956	1.012264
C	4.940487	-1.959653	2.080882
C	5.390589	-3.258233	1.805985
C	5.654375	-3.662018	0.491440
C	5.478198	-2.778308	-0.581841
C	4.725285	-0.348269	-1.216089
C	4.270025	0.326577	1.017751
H	4.726335	-1.629505	3.093197
H	5.534498	-3.963753	2.620043
H	5.995275	-4.676359	0.303048
H	5.664706	-3.082202	-1.606874
C	4.321564	0.785481	-0.371909
O	4.763176	-0.389838	-2.446405
O	3.878818	0.880604	2.050542

C	4.153274	2.042369	-0.969600
H	4.581807	2.086691	-1.966866
C	3.982516	3.340358	-0.318029
C	3.277933	3.518225	0.885877
C	4.514087	4.476046	-0.961699
C	3.129531	4.793857	1.427099
H	2.870527	2.656054	1.388766
C	4.371860	5.744684	-0.412376
H	5.042310	4.348731	-1.903590
C	3.674251	5.908355	0.788723
H	2.583768	4.917863	2.359142
H	4.799113	6.605998	-0.918588
H	3.557002	6.898852	1.220223

14. References

(1) (*a*) S. Chandrasekhar, Ch. Narsihmulu, B. Saritha and S. S. Sultana, *Tetrahedron Lett.,* 2004, **45**, 5865–5867; (*b*) P. R. Krishna, E. R. Sekhar and V. Kannan, *Tetrahedron Lett.,* 2003, **44**, 4973–4975; (*c*) S. Xue, L. He, K. Han, Y. Liu and Q. Guo, *Synlett,* 2005, **8**, 1247–1250; (*d*) Y. M. Chung, Y. J. Im and J. N. Kim, *Bull. Korean Chem. Soc.,* 2002, **23**, 1651–1654.

(2) C. J. Lee, C. N. Sheu, C. C. Tsai, Z. Z. Wu and W. Lin, *Chem. Commun.,* 2014*,* **50**, 5304–5306.

(3) (*a*) K. S. Halskov, T. K. Johansen, R. L. Davis, M. Steurer, F. Jensen and K. A. Jørgensen, *J. Am. Chem. Soc.,* 2012*,* **134**, 12943–12946; (*b*) R. Sriram, C. N. S. S. P. Kumar, N. Raghunandan, V. Ramesh, M. Sarangapani and V. J. Rao, *Synth. Commun.,* 2012, **42**, 3419–3428; (*c*) Y. Liu, Z.-J. Cai, S.-Y. Wang and S.-J. Ji, *Asian J. Org. Chem.,* 2016, **5**, 43–47.

(4) (*a*) H. J. Davis, M. E. Kavanagh, T.Balan, C. Abell and A. G. Coyne, *Bioorg. Med. Chem. Lett.,* 2016, **26**, 3735–3740; (*b*) M. A. Jinks, H. Sun and C. A. Hunter, *Org. Biomol. Chem.,* 2014*,* **12***,* 1440–1447.

(5) Q.-Q. Yang, X. Yin, X.-L. He, W. Du and Y.-C. Chen, *ACS Catal.,* 2019, **9**, 1258–1263.

(6) B.-X. Xiao, B. Jiang, X. Song, W. Du and Y.-C. Chen, *Chem. Commun.,* **2019**, *55*, 3097–3100.

(7) M. J. Frisch, G. W. Trucks, H. B. Schlegel, G. E. Scuseria, M. A. Robb, J. R. Cheeseman, G. Scalmani, V. Barone, B. Mennucci, G. A. Petersson, H. Nakatsuji, M. Caricato, X. Li, H. P. Hratchian, A. F. Izmaylov, J. Bloino, G. Zheng, J. L. Sonnenberg, M. Hada, M. Ehara, K. Toyota, R. Fukuda, J. Hasegawa, M. Ishida, T. Nakajima, Y. Honda, O. Kitao, H. Nakai, T. Vreven, J. A. Montgomery Jr., J. E.Peralta, F. Ogliaro, M. J. Bearpark, J. Heyd, E. N. Brothers, K. N. Kudin, V. N. Staroverov, R. Kobayashi, J. Normand, K. Raghavachari, A. P. Rendell, J. C. Burant, S. S. Iyengar, J. Tomasi, M. Cossi, N. Rega, N. J. Millam, M. Klene, J. E. Knox, J. B. Cross, V. Bakken, C. Adamo, J. Jaramillo, R. Gomperts, R. E. Stratmann, O. Yazyev, A. J. Austin, R. Cammi, C. Pomelli, J. W. Ochterski, R. L. Martin, K. Morokuma, V. G. Zakrzewski, G. A. Voth, P. Salvador, J. J. Dannenberg, S. Dapprich, A. D. Daniels, Ö. Farkas, J. B. Foresman, J. V. Ortiz, J. Cioslowski and D. J. Fox, Gaussian 09, Gaussian, Inc.: Wallingford, CT, USA, **2009**.

(8) (*a*) A. D. Becke, *J. Chem. Phys.,* **1993**, *98*, 5648–5652; (*b*) C. Lee, W. Yang, R. G. Parr, *Phys. Rev. B.*, **1988**, *37*, 785–789.

(9) K. Fukui, *Acc. Chem. Res.,* **1981**, *14*, 363–368.

(10) J. M. Um, D. A. DiRocco, E. L. Noey, T. Rovis and K. N. Houk, *J. Am. Chem. Soc.,* **2011**, *133*, 11249–11254.

(11) R. F. Ribeiro, A. V. Marenich, C. J. Cramer and D. G. Truhlar, *J. Phys. Chem. B.,* **2011**, *115*, 14556-14562.
